# Genome-wide characterization of the aldehyde dehydrogenase gene superfamily in soybean and its potential role in drought stress response

**DOI:** 10.1186/s12864-017-3908-y

**Published:** 2017-07-07

**Authors:** Wei Wang, Wei Jiang, Juge Liu, Yang Li, Junyi Gai, Yan Li

**Affiliations:** National Key Laboratory of Crop Genetics and Germplasm Enhancement / National Center for Soybean Improvement / Key Laboratory for Biology and Genetic Improvement of Soybean (General, Ministry of Agriculture) / Jiangsu Collaborative Innovation Center for Modern Crop Production, Nanjing Agricultural University, Nanjing, Jiangsu China

**Keywords:** ALDH, *cis*-element, Drought stress, Gene network, Phylogenetic analysis, Soybean

## Abstract

**Background:**

Aldehyde dehydrogenases (ALDHs) represent a group of enzymes that detoxify aldehydes by facilitating their oxidation to carboxylic acids, and have been shown to play roles in plant response to abiotic stresses. However, the comprehensive analysis of ALDH superfamily in soybean (*Glycine max*) has been limited.

**Results:**

In present study, a total of 53 GmALDHs were identified in soybean, and grouped into 10 ALDH families according to the ALDH Gene Nomenclature Committee and phylogenetic analysis. These groupings were supported by their gene structures and conserved motifs. Soybean ALDH superfamily expanded mainly by whole genome duplication/segmental duplications. Gene network analysis identified 1146 putative co-functional genes of 51 *GmALDHs*. Gene Ontology (GO) enrichment analysis suggested the co-functional genes of these 51 *GmALDHs* were enriched (FDR < 1e-3) in the process of lipid metabolism, photosynthesis, proline catabolism, and small molecule catabolism. In addition, 22 co-functional genes of *GmALDHs* are related to plant response to water deprivation/water transport. *GmALDH*s exhibited various expression patterns in different soybean tissues. The expression levels of 13 *GmALDH*s were significantly up-regulated and 14 down-regulated in response to water deficit. The occurrence frequencies of three drought-responsive *cis*-elements (ABRE, CRT/DRE, and GTGCnTGC/G) were compared in *GmALDH* genes that were up-, down-, or non-regulated by water deficit*.* Higher frequency of these three *cis*-elements was observed for the group of up-regulated *GmALDH* genes as compared to the group of down- or non- regulated *GmALDH*s by drought stress, implying their potential roles in the regulation of soybean response to drought stress.

**Conclusions:**

A total of 53 *ALDH* genes were identified in soybean genome and their phylogenetic relationships and duplication patterns were analyzed. The potential functions of *GmALDHs* were predicted by analyses of their co-functional gene networks, gene expression profiles, and *cis*-regulatory elements. Three *GmALDH* genes, including *GmALDH3H2*, *GmALDH12A2* and *GmALDH18B3*, were highly induced by drought stress in soybean leaves. Our study provides a foundation for future investigations of *GmALDH* gene function in soybean.

**Electronic supplementary material:**

The online version of this article (doi:10.1186/s12864-017-3908-y) contains supplementary material, which is available to authorized users.

## Background

Due to the unpredictability of environmental conditions and the inability of plants to move in order to avoid unfavorable conditions, the growth and productivity of plants are adversely affected by various environmental stresses, including drought, high temperature, and salinity [1–3]. Among abiotic stresses, drought is considered to have the harshest, negative effects on plant growth and development, which can lead to significant losses in productivity [4, 5]. Rising global temperatures and decreases in available water resources in the world will exacerbate the negative impact of the drought stress on plants [6, 7]. Aldehydes are both intermediates in and byproducts of some fundamental metabolic pathways associated with carbohydrate, amino acid, and lipid metabolism [8, 9]. Furthermore, abiotic stresses, such as drought, high salinity, and high temperature, also lead to the accumulation of reactive oxygen species (ROS) which further promote endogenous aldehyde production via a lipid peroxidation chain reaction [10–12]. Excessive amounts of endogenous aldehydes have detrimental effects on cellular metabolism due to their chemical reactivity and toxicity, and consequently can have a negative impact on the growth and development of plants [10, 13]. Therefore, excessive aldehydes need to be eliminated in order to maintain non-harmful levels. Aldehyde dehydrogenases (ALDHs) play an essential role in the detoxification of aldehyde molecules by facilitating the irreversible oxidation of a series of endogenous and exogenous aromatic and aliphatic aldehydes into their corresponding non-toxic carboxylic acids [10, 11, 14].

Aldehyde dehydrogenases (ALDHs) comprise a superfamily of genes containing several gene families encoding NAD(P)^+^-dependent enzymes that are widely distributed in prokaryotes and eukaryotes [8, 15]. ALDHs have been divided into 24 distinct protein families [15–17]. Among the 24 families, fourteen families (ALDH2, ALDH3, ALDH5, ALDH6, ALDH7, ALDH10, ALDH11, ALDH12, ALDH18, ALDH19, ALDH21, ALDH22, ALDH23, and ALDH24) contain members from plant species, and seven families (ALDH11, ALDH12, ALDH19, ALDH21, ALDH22, ALDH23 and ALDH24) are unique to plants [17]. The number of sequenced plant genomes is greatly increasing. Therefore, the occurrence and composition of *ALDHs* have been extensively studied in several plant species, including *Physcomitrella patens* [15], *Chlamydomonas reinhardtii* [15], *Arabidopsis thaliana* [14], *Oryza sativa* [18, 19], *Zea mays* [20], *Gossypium raimondii* [21], *Sorghum bicolor* [15], and *Foxtail Millet* [22].

The potential functions of some *ALDHs* have been investigated in previous studies. Specifically, *ALDHs* have been found to respond to several abiotic stresses, including drought, high temperature, salinity, and oxidative stress, suggesting the potential roles of *ALDHs* in stress tolerance [16, 18]. For example, *ALDH22A1* in maize was induced by dehydration, high salinity, and ABA treatment. Overexpression of this gene in tobacco significantly improved stress tolerance as evidenced by a reduction in malondialdehyde (MDA) accumulation [23]. The expression of *TraeALDH7B1-5A* was up-regulated in roots, leaves, culms, and spikelets of wheat subjected to drought and salt stresses. Transgenic Arabidopsis over-expressing *TraeALDH7B1-5A* had significantly enhanced tolerance to drought stress and exhibited the up-regulation of stress responsive genes [11]. Recently, CaALDH1*,* an aldehyde dehydrogenase in pepper, was demonstrated to interact with AvrBsT (*Xanthomonas* type III effector) to promote AvrBsT-triggered cell death in tobacco (*Nicotiana benthamiana*)*,* and over-expression of *CaALDH1* gene in Arabidopsis enhanced the defense response to *Pseudomonas syringae* pv. *tomato* and *Hyaloperonospora arabidopsidis* infection [24]*.* Some ALDH members have also been reported to play major roles in controlling or influencing the growth and development of plants [25, 26].

Soybean (*Glycine max*) is the most widely grown seed legume worldwide and provides an inexpensive source of protein and oil [27, 28]. The complete genome sequence of the palaeopolyploid soybean was made available in 2010 [29] and thus has provided an opportunity to identify and characterize the *ALDH* gene superfamily in soybean. Kotchoni et al. previously identified 18 *ALDH* genes in soybean, encoding members of five different ALDH protein families [30]. A more comprehensive analysis of the *ALDH* gene superfamily in soybean, however, is lacking, and its role in abiotic stress response is unknown. In the present study, a genome-wide search of the soybean genome sequence identified 53 *GmALDHs.* Subsequently, the phylogenetic relationships, gene structures, protein motifs, duplication patterns, co-functional gene networks, tissue expression patterns of the soybean *ALDHs* and their response to drought stress were all analyzed. Lastly, the *cis*-elements related to hormone and stress-response in the promoter regions of all 53 soybean *ALDHs* were also investigated. The information generated in this study provides a foundation to further investigate the functions of *ALDH* genes in soybean.

## Results

### Identification and characteristics of soybean ALDH superfamily

In order to comprehensively identify ALDH members in soybean, we used the keywords of “ALDH” and “Aldehyde dehydrogenases”, and the Hidden Markov Model (HMM) profile of the ALDH domain (PF00171) as queries, to search the latest version of the soybean genome (Wm82.a2.v1) in the Phytozome v11.0 database. Subsequently, BLAST searches using all of the Arabidopsis ALDH sequences as queries were performed to re-check the obtained sequences. As a result, 63 putative soybean ALDH candidates were identified. The conserved ALDH domain (PF00171) was confirmed with SMART, and the presence of the ALDH cysteine active site (PS00070) and the ALDH glutamic acid active site (PS00687) were examined using PROSITE. After removing false-positive sequences, a total of 53 soybean ALDHs*,* grouped into 10 families, were identified (Additional file 1: Table S1). Each soybean *ALDH* gene was given an ALDH family name based on criteria established by the ALDH Gene Nomenclature Committee (AGNC) [31], and denominated according to soybean nomenclature, with the ALDH family name followed by a number based on the physical location of the gene on soybean chromosomes (Additional file 1: Table S1). All 53 putative soybean ALDH proteins possess a conserved ALDH (PF00171) domain (Additional file 2: Table S2), which is a basic characteristic of ALDH families. All 10 ALDH families are represented by more than one member in soybean (Additional file 1: Table S1): ALDH2 is represented by 18 members; ALDH3 by 11 members; ALDH5, ALDH7, and ALDH10 by two members; ALDH6, ALDH11, and ALDH12 by three members; ALDH18 by five members; and ALDH22 by four members. These soybean *ALDH* genes encode proteins that range from 444 (*GmALDH2B3*) to 756 (*GmALDH18B3*) amino acids (aa) in length, with molecular weights (kDa) that range from 48.70 (*GmALDH2B3*) to 82.18 kD (*GmALDH18B3*), and predicted isoelectric points (pI) in the range of 5.23 (*GmALDH10A2*) to 9.11 (*GmALDH3H3*). The prediction of subcellular localization using WoLF PSORT reveals that 60.4% (32 out of 53) of GmALDHs locate to the cytoplasm.

### Distribution and phylogenetic analysis of the ALDH superfamily among different organisms

In order to investigate the distribution of the ALDH superfamily, members of the ALDH superfamily in 19 different organisms whose *ALDH* genes have been previously reported were analyzed (Additional file 3: Figure S1). Results indicated that the ALDH superfamily in soybean has the most members with 53 ALDHs, which is more than in other species, such as 39 ALDHs in *M. domestica*, 30 ALDHs in *G. raimondii*, 26 ALDHs in *P. trichocarpa*, and 23 ALDHs in *Z. mays* (Additional file 3: Figure S1). The ALDH2 family was found to be the largest ALDH family in plants, with 18 members in soybean, 13 members in apple, and 8 members in cotton. In addition, soybean was found to contain four ALDH22 members. This represents a significant difference from other species, where ALDH22 is represented by a single gene member, with the exception of apple.

**Fig. 1:**
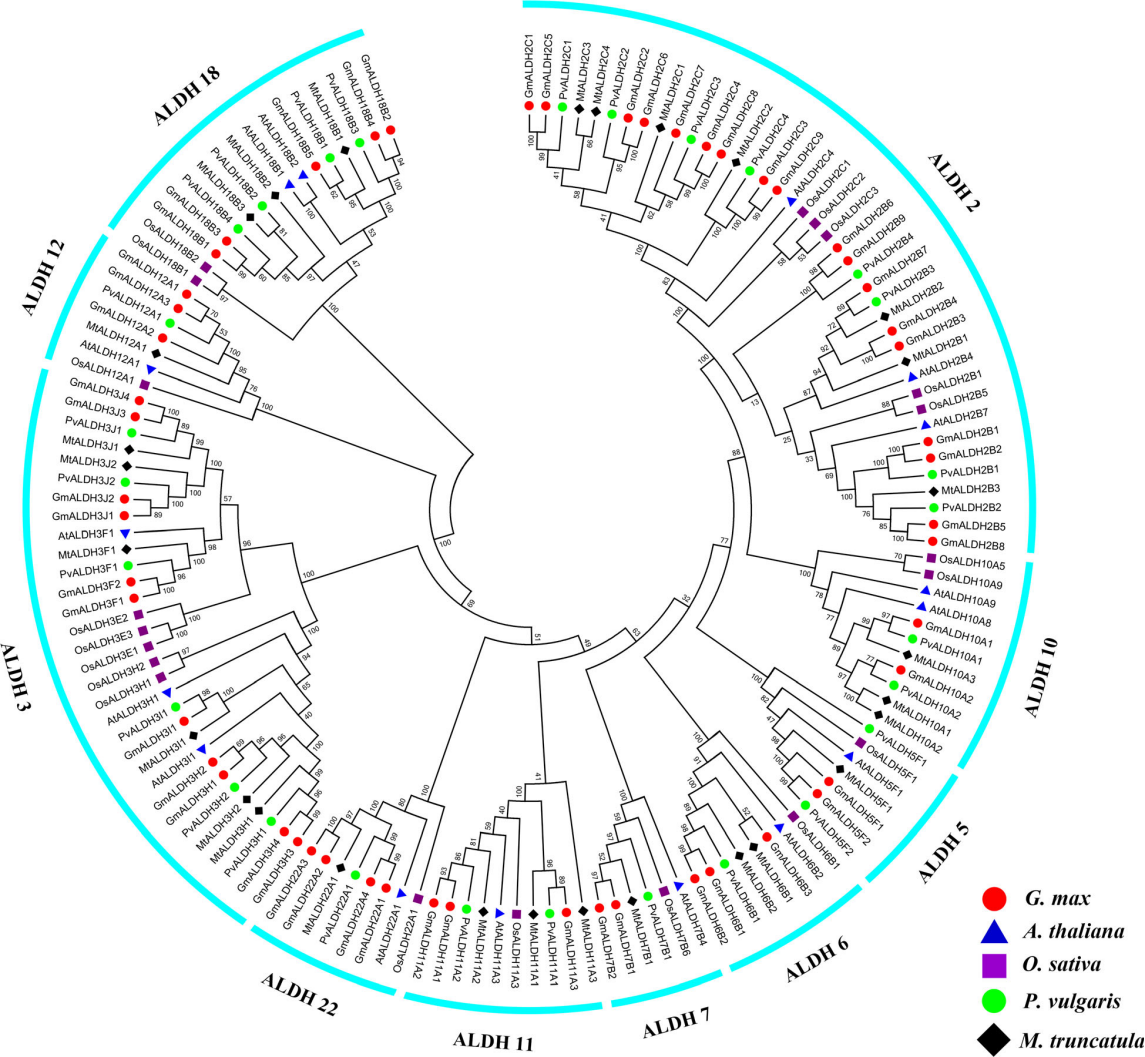
Phylogenetic tree of ALDH proteins from *G. max* (Gm), *A. thaliana* (At), *O. sativa* (Os), *P. vulgaris* (Pv), and *M. truncatula* (Mt). Alignment of 145 ALDH protein sequences from five plant species was conducted with ClustalW2, and the phylogenetic tree was constructed using MEGA 5.2 based on the Neighbor-joining (NJ) method. Bootstrap values in percentage (1000 replicates) are labeled on the nodes

A neighbor-joining method was used to construct a phylogenetic tree with the full amino acid sequences of 145 ALDH proteins (Fig. 1 and Additional file 4: Table S3) from three legume species including common bean (*Phaseolus vulgaris* L.), Medicago (*Medicago truncatula*), and soybean (*G. max*), as well as Arabidopsis (*A. thaliana*) and rice (*O. sativa*). The resulting dendrogram illustrates that the 53 GmALDHs were grouped into 10 distinct families, together with their orthologous ALDHs from common bean, Medicago, Arabidopsis, and rice (Fig. 1), with every family containing both monocotyledons and dicotyledons members. Generally, the putative ALDH protein sequences from the same family or subfamily were clustered together. Overall, ALDHs from soybean have a closer relationship with ALDHs from common bean and Medicago than those from Arabidopsis and rice (Fig. 1), which is consistent with the fact that common bean, Medicago and soybean are all legume species. Lastly, in relative comparison to the other ALDH families, the ALDH18 family is the most distantly related family.

### Exon–intron structures and conserved motifs of soybean ALDH superfamily

**Fig. 2:**
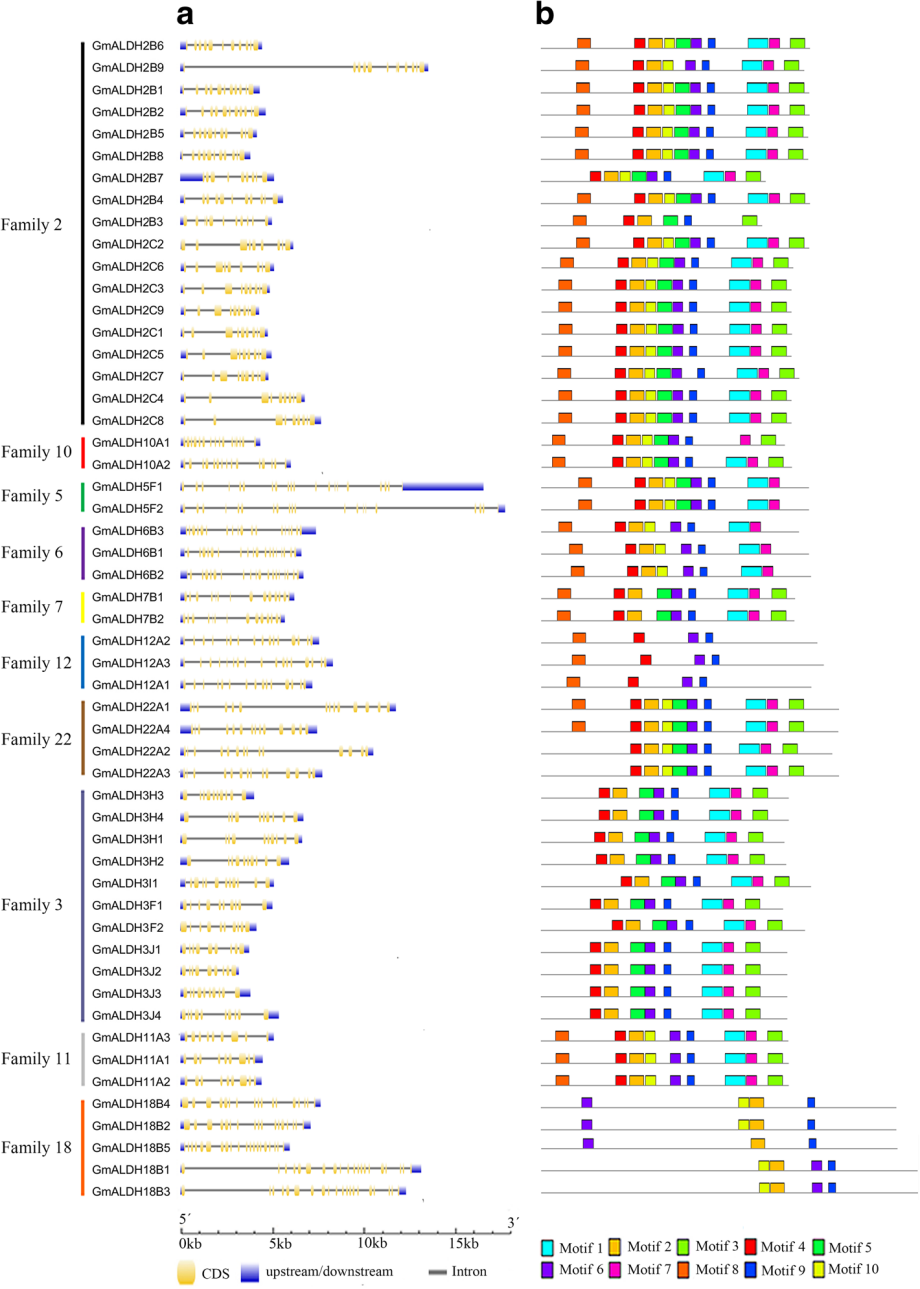
Gene structures and protein motifs of the soybean ALDH superfamily. **a**. The exon/intron structures of soybean *ALDH* genes. The relative position is proportionally displayed based on the kilobase scale at the bottom of the figure. Yellow boxes, gray lines, and blue boxes represent exons, introns, and upstream/downstream regions, respectively. **b**. The conserved motifs in soybean ALDHs. Different motifs and their relative positions are represented by the colored boxes

In order to obtain additional information pertaining to the conservation and diversification of *GmALDH* genes, their structures were analyzed using the GSDS online suite [32] and schematically illustrated (Fig. 2a) based on their evolutionary relationships (Additional file 5: Figure S2). The numbers of exons and introns in *GmALDH* genes range from 9 to 20, and 8 to 19, respectively. All *GmALDH* genes within the ALDH 5, 7, 11, and 22 families contain 20, 14, 9, or 14 exons, respectively, indicating that the majority of *GmALDH* genes within the same family or subfamily share a highly conserved gene structure. In contrast, a greater diversification in gene structure was observed in the remaining ALDH families (2, 3, 6, 10, 12 and 18), which have several exon/intron structure variants. For example, in ALDH18 family, *GmALDH18B2* and *GmALDH18B4* contain 17 exons while the remaining members (*GmALDH18B1, GmALDH18B3* and *GmALDH18B5*) possess 20 exons.

The putative amino acid sequences of the soybean ALDHs were further analyzed for the conserved motifs using MEME suite 4.11.1 [33]. A total of 10 conserved motifs were identified in the collective putative GmALDH proteins and designated as motif 1 to motif 10 based on the E-value of each motif (Fig. 2b and Additional file 6: Figure S3A). The most common motif at the N-terminal is motif 4, which was found in 48 out of 53 (90.6%) soybean ALDHs, and motif 8 is another common motif at the N-terminal. Motif 7 was found at the C-terminal in 44 out of 53 (83.0%) GmALDHs. The results also showed that the type and number of conversed motifs were highly similar within each of the ALDH 3, 5, 6, 7, 11 and 12 family, suggesting that there might be functional similarities of GmALDH proteins within the same family. This premise is consistent with a similar statement reported in apple [34]. Some motifs are missing in certain families, for example: ALDH3 family does not have motifs 8 and 10, ALDH5 family does not possess motif 3; ALDH6 family does not have motifs 3 and 5; ALDH12 family does not have motifs 1, 2, 3, 5, 7 and 10; while ALDH18 family only has motifs 2, 6, 9 and 10. These results suggest that there might be a functional divergence in GmALDHs among the different ALDH families. In addition, these 10 conserved motifs of GmALDHs were also observed in putative ALDH proteins from the other four species**,** including common bean, Medicago, Arabidopsis, and rice, suggesting that these putative ALDH proteins were conserved among different plant species (Additional file 6: Figure S3B).

### Chromosomal distribution and expansion patterns of soybean *ALDH* genes

MapChart [35] was used to illustrate the physical positions of *GmALDH* genes on their corresponding chromosomes. Results indicate that the 53 *GmALDH* genes are distributed across 18 chromosomes and their distribution is uneven (Additional file 7: Figure S4). Among the 20 chromosomes in soybean, chromosomes 10 and 20 carry no *GmALDH* genes, while chromosomes 3, 11, 12, 16 and 19 contain only one *GmALDH* gene. Chromosome 8 possesses six *GmALDH* genes, and all of the other remaining chromosomes contain two to five *GmALDH* genes. The results also revealed that most *GmALDH* genes are located on chromosome arms, which is in agreement with the genome-wide gene distribution pattern, where approximately 78% of all soybean genes locate on the chromosome arms [29].

**Fig. 3:**
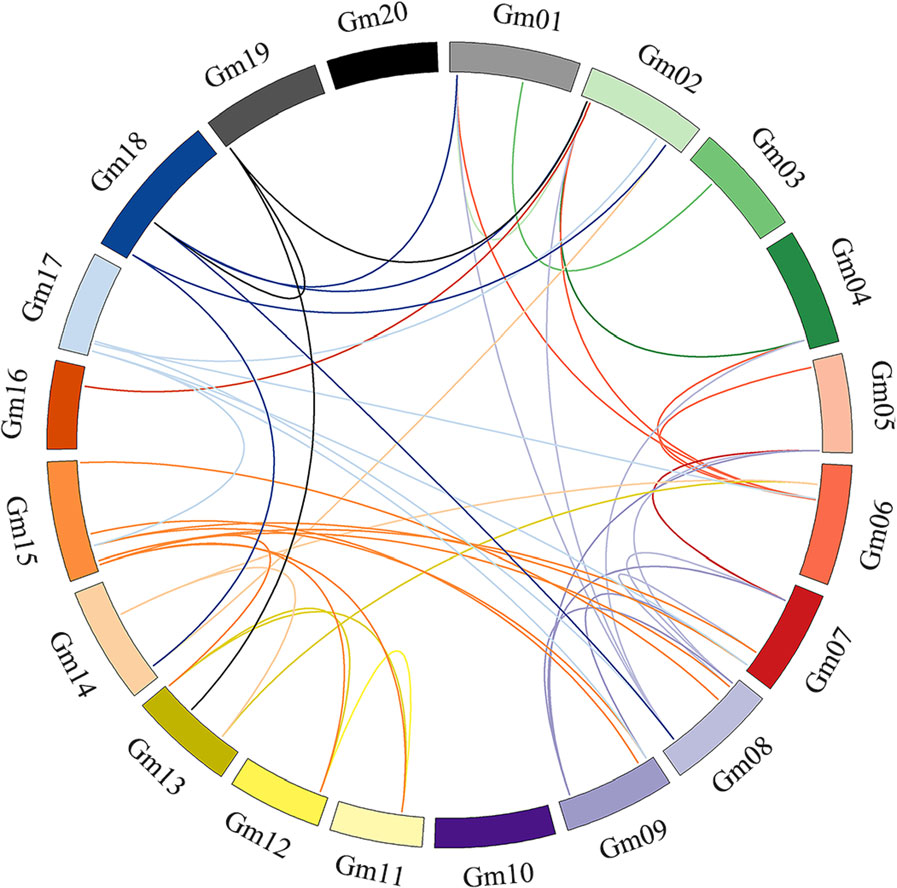
WGD/segmental duplications of soybean *ALDH* genes. Twenty soybean chromosomes are displayed in different colors. WGD/segmentally duplicated *GmALDH* gene pairs are linked by different colored lines. The illustration was generated using CIRCOS software

Gene duplication events that occurred during the evolution of genomes are considered as the major mechanisms that contributed to the complexities of genomes and the expansion of gene families in plants [36]. The occurrence of whole genome duplication (WGD)/segmental duplication and small-scale (local) tandem duplication events in *GmALDH* genes was examined (Fig. 3, Additional file 7: Figure S4 and Additional file 8: Table S4), in order to get more information about the mechanisms that contributed to the expansion of the soybean *ALDH* genes. The results indicated that one pair of *GmALDHs* (*GmALDH12A1* and *GmALDH12A2*) was the result of a single tandem duplication (2 out of 53 genes, 3.8%), four pairs of *GmALDHs* (8 out of 53 genes, 15.9%) expanded through both tandem and WGD/segmental duplication (including *GmALDH2C1*, *GmALDH2C2*, *GmALDH2C3*, *GmALDH2C4, GmALDH2C5*, *GmALDH2C6*, *GmALDH2C7*, *GmALDH2C8*), and 47 out of the 53 (88.7%, including the 8 genes from both tandem and WGD/segmental duplication) *GmALDH* genes underwent WGD/segmental duplication (Additional file 8: Table S4). Interestingly, most of *GmALDH* genes (32 out of 53 genes, 60.4%) have more than one-pair of duplicates, for example, *GmALDH2B1* has four duplicated genes (Additional file 8: Table S4).

Since 88.7% of the *GmALDH* genes underwent WGD/segmental duplication, we further estimated the possible duplication time according to their pairwise synonymous distances (*K*s values) based on the previous study in soybean [29]: *K*s values of 0.06–0.39 correspond to the 13-Ma ago (Mya) *Glycine*-lineage-specific genome duplication, and *K*s values of 0.40–0.80 correspond to the 59-Mya early-legume WGD, while the *K*s values greater than 1.5 probably correspond to the more ancient ‘gamma’ event [37]. Based on the *K*s values (Additional file 8: Table S4), 21 duplicated *GmALDH* pairs (with *K*s values of 0.07–0.32) were associated with the 13 Mya *Glycine*-lineage-specific genome duplication, and 25 duplicated *GmALDH* pairs (with *K*s values of 0.41–0.80) were associated with the 59 Mya early-legume WGD, while seven duplicated *GmALDH* pairs (with *K*s values of 0.82–1.56) might be associated with more ancient genome duplication, which are consistent with the previous study [29].

### Potential co-functional genes of *GmALDHs*

To identify the potential co-functional genes of *GmALDHs*, we constructed the co-functional gene networks of *GmALDHs* (Additional file 9: Figure S5) using the SoyNet database [38]. A total of 1146 gene pairs of co-functional links were identified for 51 out of 53 *GmALDH* genes (96.2%), with the average of 22.5 co-functional genes per *GmALDH* in soybean (Additional file 10: Table S5). A great variation in the number of co-functional genes is present among the *GmALDH* genes. For example, *GmALDH10A2* and *GmALDH7B1* have 101 and 86 putative co-functional genes, respectively, whereas *GmALDH18B1* has only one putative co-functional gene. The Gene Ontology (GO) enrichment analysis (Additional file 11: Figure S6) suggests that the co-functional genes of *GmALDHs* are enriched (FDR < 1e-3) in the following biological processes: lipid metabolism (GO: 0006629, FDR **=** 0.000132), metabolic process (GO: 0008152, FDR = 0.000216), photosynthesis (GO: 0015979, FDR **=** 1.24e-05), proline catabolism (GO: 0006562, FDR **=** 9.37e-05) and small molecule catabolism (GO: 0044282, FDR **=** 3.48e-05). In addition, there are 22 co-functional genes that are related to response to water deprivation/water transport (Additional file 10: Table S5), suggesting the potential roles of these genes and their co-functional *GmALDHs* in plant response to drought stress.

### Expression profiles of *GmALDHs* in different soybean tissues

**Fig. 4:**
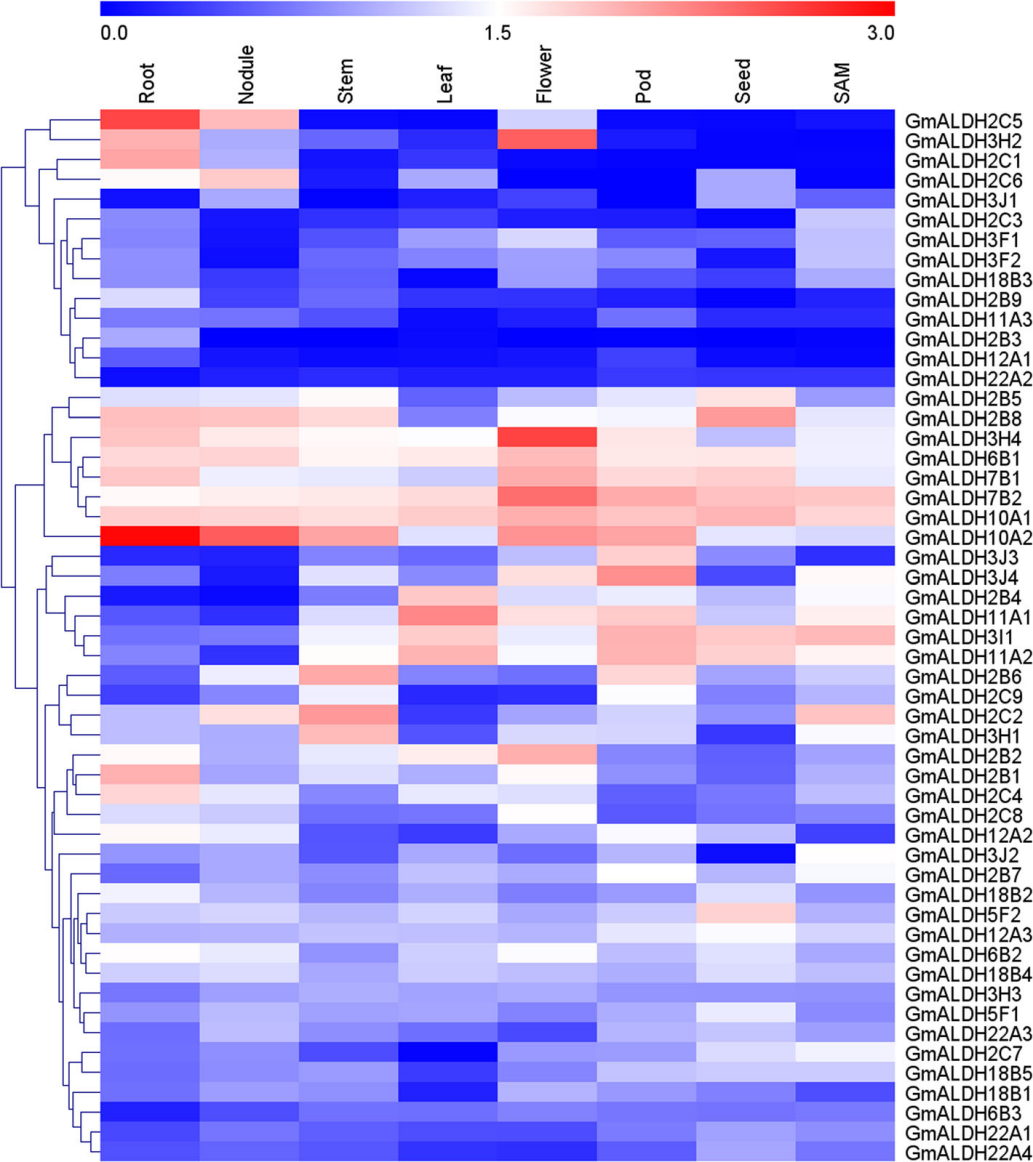
Heat map of the expression profiles of *GmALDHs* in eight different soybean tissues. The color scale represents the relative transcript abundance of the *GmALDH* genes in eight soybean tissues. The heat map with hierarchical clustering of *GmALDH* genes was constructed based on log10 (FPKM + 1) values using MeV 4.9 software by average linage with Euclidean distance. The FPKM values were obtained from the RNA-seq data at Phytozome v11.0

Since RNA-seq data of *G. max* is available [39], it is possible to investigate the in silico expression profiles of the soybean *ALDH* genes in different soybean tissues and gain an elementary understanding of the potential functions of the different *GmALDHs*. RNA-seq data (FPKM values, fragments per kilobase per million mapped fragments) for all of the *GmALDHs* in eight tissues, including root, nodule, stem, leaf, flower, pod, seed and shoot apical meristem (SAM), were obtained from Phytozome11.0 [40]. Transcript abundance for all 53 *GmALDH* genes were identified (Fig. 4 and Additional file 12: Table S6) and showed variation in tissue expression patterns. Some *GmALDH* genes exhibited tissue-specific expression, such as *GmALDH2C1* and *GmALDH2C5*, which showed a higher expression level in root than other tissues; while *GmALDH3H2* and *GmALDH3H4* were highly expressed in flower. Many *GmALDH* genes expressed at high levels in multiple tissues. For example, *GmALDH3I1* and *GmALDH11A2* exhibited high levels of transcript abundance in leaf/pod/seed/SAM. Some *GmALDH* genes exhibited high levels of expression in all of the tissues for which expression data was available. This included *GmALDH7B2* and *GmALDH10A1*. In contrast, the expression levels of some *GmALDH* genes, such as *GmALDH12A1* and *GmALDH22A2,* were very low in all of the examined tissues. In addition, some of the tandem and WGD/ segmental duplicated genes exhibited distinct expression patterns, such as *GmALDH2C1*/*GmALDH2C2* and *GmALDH12A1*/*GmALDH12A2*.

**Fig. 5:**
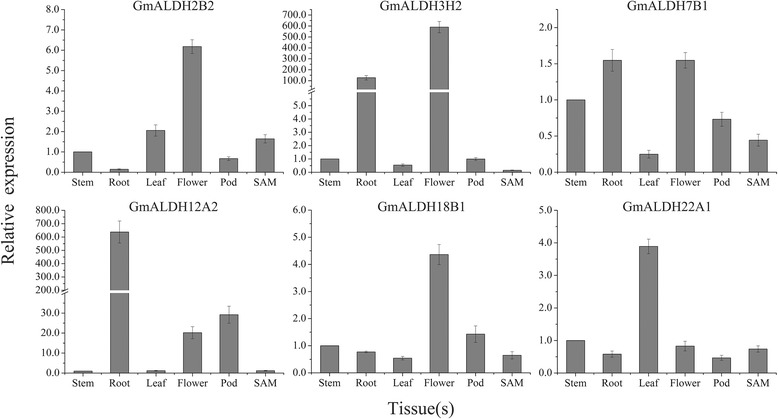
Relative expression levels of six *GmALDHs* in different soybean tissues by qRT-PCR. For each gene, the relative expression level in stem was set as one, and the soybean *60S* gene was used as the internal control. The data represent the mean ± SD of three replicates

In order to validate the expression levels obtained from the RNA-Seq data, six *GmALDH* genes were randomly selected from six different soybean ALDH families for quantitative reverse transcription polymerase chain reaction (qRT-PCR) analysis. Transcript levels were analyzed in six different tissues, including stem, root, leaf, flower, pod, and SAM (Fig. 5). The qRT-PCR analysis demonstrated that *GmALDH2B2* and *GmALDH18B1* were highly expressed in flower whereas the relative expression level of *GmALDH3H2* was higher in flower and root. *GmALDH7B1* expressed at a relatively consistent level in all tissues except leaf. *GmALDH12A2* was highly expressed in root and pod. The relative expression level of *GmALDH22A1* was higher in leaf than other tissues. In general, the expression levels obtained by qRT-PCR for these genes are similar to the results obtained from the in silico analysis of the RNA-seq data.

### Expression analysis of *GmALDHs* in response to drought stress

**Fig. 6:**
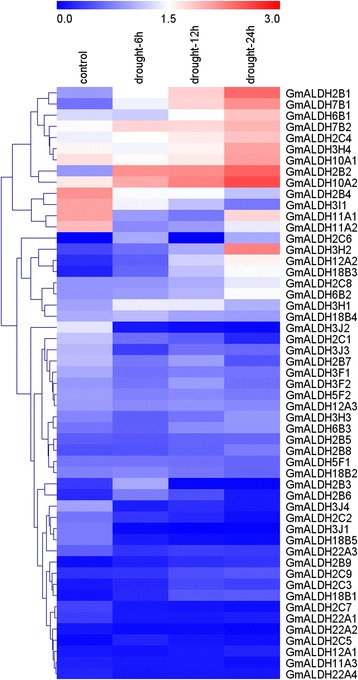
Heap map of the expression profiles of *GmALDHs* in soybean plants subjected to drought stress for 0, 6, 12 and 24 h. The color scale is based on the log10 (FPKM + 1) values. FPKM values of 53 *GmALDHs* were obtained from the RNA-seq data using leaf samples of the drought-tolerant soybean cultivar, PI416937. The heat map with hierarchical clustering of *GmALDH* genes was constructed using MeV 4.9 software by average linage with Euclidean distance

Soybean *ALDH* genes were also analyzed in silico for their response to drought stress using publicly available soybean leaf transcriptome data [41]. FPKM values for the 53 *GmALDH* genes (Additional file 13: Table S7) were retrieved from RNA-seq data from the leaves of the soybean drought-tolerant cultivar PI416937 that had been subjected to a drought treatment for 0, 6, 12 and 24 h [41]. A heat map was constructed based on the log10 (FPKM + 1) values for the 53 soybean *ALDHs* (Fig. 6). The criteria of *P* value < 0.05 and │log_2_ fold change│ ≥ 2 was used to identify soybean *ALDHs* that were differentially expressed between drought stress and control [27, 42]. The results indicated that the expression levels of most *GmALDH* genes were significantly altered by the drought stress for at least one time point (Additional file 14: Table S8). Compared to the well-watered control, 13 (24.5%) *GmALDH* genes were significantly up-regulated and 14 (26.4%) down-regulated in soybean leaves in response to water deficit (Additional file 14: Table S8). For example, *GmALDH2B2* was highly up-regulated in response to the drought treatment at 6, 12 and 24 h, while the expression level of *GmALDH3J2* and *GmALDH3J4* decreased quickly in response to the drought treatment.

**Fig. 7:**
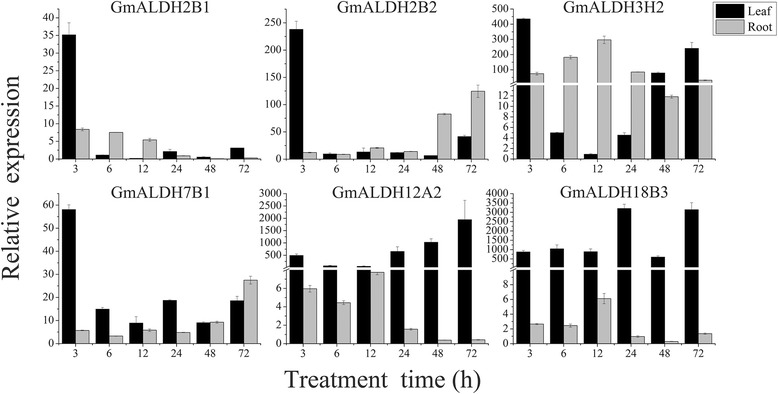
Relative expression levels of six soybean *ALDHs* in response to a simulated drought stress. The drought tolerant soybean cultivar, KF-1, was grown hydroponically for two weeks and then exposed to 20% PEG treatment. The relative expression levels of six *ALDH* genes at 3, 6, 12, 24, 48, and 72 h were determined by qRT-PCR in comparison with 0 h. The soybean *60S* gene was used as the internal control. The data represent the mean ± SD of three replicates

Six out of 13 of the up-regulated *GmALDH* genes exhibited a significant fold change ≥ 5.0 for at least one time point. These genes were selected for further analysis of their response to drought stress using qRT-PCR. After grown under well-watered condition for two weeks, the drought-tolerant soybean cultivar (KF-1) was treated with 20% PEG and sampled at 0, 3, 6, 12, 24, 48, and 72 h. The relative expression levels of the six *GmALDH* genes in the leaves and root tips of soybean plants are shown in Fig. 7. The expression levels of all six genes were up-regulated in response to 20% PEG treatment. These results are in agreement with the transcriptome data. The transcript levels of *GmALDH2B1*, *GmALDH2B2* and *GmALDH7B1* in soybean leaves were rapidly up-regulated at 3 h but then decreased at later time points. The expression level of *GmALDH2B2* and *GmALDH7B1* in soybean roots increased as the duration of PEG treatment progressed. In contrast, the relative expression level of *GmALDH2B1* in roots was highest at 3 h and subsequently decreased to lower levels of expression at later time points. The transcript levels of *GmALDH12A2* and *GmALDH18B3* in soybean leaves were significantly up-regulated by the 20% PEG treatment at all time points (3–72 h), but their expression levels in roots did not exhibit any dramatic changes. In comparison to the other five up-regulated *GmALDH* genes, *GmALDH3H2* showed the greatest induced expression in response to drought stress in roots (Fig. 7).

### Characterization of putative *cis*-regulatory elements in the promoter regions of drought-responsive *GmALDHs*

**Fig. 8:**
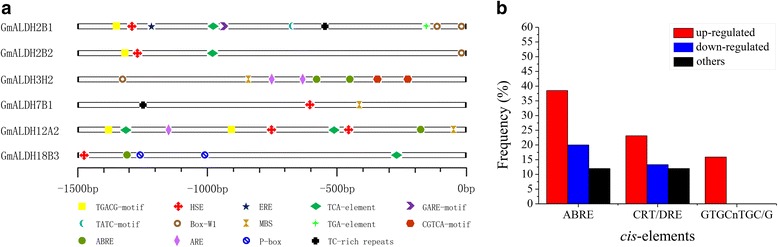
Analysis of the *cis*-elements in the promoter regions of soybean *ALDH* genes. **a**. Predicted *cis*-elements in the 1500 bp upstream regions of six *GmALDH* genes. The names of the *cis*-elements are indicated at the bottom of the figure. The scale indicates the relative position of each *cis*-element relative to the translation start codon, ATG. **b**. Frequency of three drought-responsive *cis*-elements present in the promoter regions of different groups of soybean *ALDHs*. The frequencies of ABRE, CRT/DRE, and GTGCnTGC/G *cis*-elements in up-regulated, down-regulated and non-responsive *GmALDH* genes under drought stress are shown in different colored bars. Changes in expression were determined by the criteria of *P* value < 0.05 and │log_2_ fold change│ ≥ 2 comparing drought-stressed soybean plants with control

*Cis*-acting regulatory elements in the promoter regions of genes play important roles in the transcriptional regulation of genes associated with abiotic stress responses, such as drought and heat stress [43]. In addition, phytohormones, such as salicylic acid (SA), jasmonic acid (JA), ethylene (ET), and abscisic acid (ABA), also play essential roles in plant adaptation to stresses by inducing an interaction between transcription factors and their corresponding *cis*-elements [44, 45]. In order to gain insight into the transcriptional regulation of *GmALDH* genes, putative stress- or hormone- responsive *cis*-acting elements were identified using the PlantCARE database [46], in the 1500 bp region upstream of the translation start codon of the six *GmALDH* genes that showed greatly increased expression levels (fold change ≥ 5.0) in response to drought stress (Fig. 8a). Fourteen putative *cis*-acting elements were identified (Additional file 15), including five stress-responsive (ARE, Box-W1, HSE, MBS and TC-rich repeats) and nine hormone-responsive (ABRE, CGTCA-motif, ERE, GARE-motif, P-box, TATC-motif, TCA-element, TGACG-motif and TGA-element) *cis*-acting elements. The *GmALDH7B1* promoter region contains only three stress responsive *cis*-elements, while the other five genes contain both stress and hormone-responsive *cis*-elements in their promoter regions. The stress- and hormone-responsive *cis*-elements in the promoter regions of the remaining 47 *GmALDHs* were also identified. The analysis indicates that promoter regions of the majority of *GmALDHs* contain both hormone- and stress- responsive *cis*-elements (Additional file 16: Table S9). A great variation in the number of *cis*-elements is present in the promoter regions of the *GmALDH* genes. For example, the promoter regions of *GmALDH2B1* and *GmALDH2B3* have 10 *cis*-elements, whereas the promoter region of *GmALDH18B2* only contains a single *cis*-element (Additional file 16: Table S9).

A recent study reported that ABRE, CRT/DRE, and a novel GTGCnTGC/G element are present in the promoter regions of the 50 soybean genes that responded most strongly to drought stress, and these *cis*-elements could be used as components of synthetic drought-inducible promoters to drive the expression of transgenes [47]. Therefore, the numbers of these three types of *cis*-elements present in the promoter regions of soybean *ALDHs* were counted (Fig. 8b and Additional file 16: Table S9). Based on the criteria used to identify the differentially expressed genes (DEGs) in drought-stressed vs. control plants, the 53 soybean *ALDHs* were divided into three groups: up-regulated; down-regulated; and others (non-DEGs). Five (38.5%) ABRE, three (23.1%) CRT/DRE, and two (15.9%) GTGCnTGC/G *cis*-elements are present in the regulatory region of the 13 up-regulated *GmALDH* genes, while only three (20%) ABRE, two (13.3%) CRT/DRE, and zero (0%) GTGCnTGC/G *cis*-elements are present in the 14 down-regulated *GmALDH* genes. The frequency of the occurrence of these three *cis*-elements in genes that exhibited no change in expression in response to drought stress is much lower than it is in the DEGs. These observations are consistent with the previous study [47], which implies that these *cis*-elements, and the genes that they are associated with, play important roles in regulating the response of soybean to drought stress*.*

## Discussion

ALDHs have been characterized and analyzed in both prokaryotes and eukaryotes [8, 15]. However, a genome-wide identification and a comprehensive analysis of this gene family in soybean have not been previously reported. In the present study, a total of 53 *ALDH* genes were identified in the soybean genome that were clustered into 10 ALDH families (including family 2, 3, 5, 6, 7, 10, 11, 12, 18, and 22) based on criteria defined by the ALDH Gene Nomenclature Committee (AGNC) [31]. While plant ALDHs can be grouped into 14 families, only 10 ALDH families are present in soybean and other magnoliophyta plants. These data suggest that these 10 families may have evolved before the divergence of monocots and eudicots. To date, members belonging to ALDH21 and ALDH23 have only been found in *S. mollendorffii* and *P. patens*, while ALDH24 appears to be unique to *C. reinhardtii*. These observations imply that these three families may have played an important role in the evolution of the transition of aquatic plants to terrestrial plants. Additionally, it is plausible that these were then subsequently lost during the evolution of magnoliophyta plants.

Gene duplication is considered as a major driving force for the expansion of gene families and evolution of novel gene functions [36]. Five mechanisms of gene duplications were proposed, including WGD (or polyploidization), tandem duplication, segmental duplication, transposon-mediated duplication, and retroduplication [48]. Among these, tandem duplication and WGD/segmental duplications have been considered as the main duplication patterns for the expansion of gene families [36]. WGD/segmental duplications occur more frequently in plants because most plants retain numerous duplicated chromosomal blocks within their genomes through polyploidy followed by chromosome rearrangements [36, 49]. The present study revealed that both tandem duplication and WGD/segmental duplication have contributed to the expansion of the soybean ALDH gene superfamily, with 47 *GmALDH* genes underwent WGD/segmental duplication (Additional file 8: Table S4), suggesting that WGD/segmental duplication played a major role in the expansion of the *ALDH* gene family in soybean. The soybean genome has undergone two rounds of WGD in its evolutionary history at approximately 59 and 13 million years ago, resulting in a highly duplicated genome with nearly 75% of the genes present in multiple copies [29]. The estimated time of the WGD/segmental duplication events (Additional file 8: Table S4) indicated that the expansion of soybean *ALDH* gene family is probably occurred along with the two key WGD events, and that these genes were retained during evolution. In addition, three pairs (*GmALDH3J1/GmALDH3J4*, *GmALDH2C2/GmALDH2C8* and *GmALDH3J2/GmALDH3J4*) have *K*s values greater than 1.5, indicating that these duplicated pairs might originate from the ‘gamma’ event [29]. This premise is consistent with *ALDH* duplication events in other plant species [16, 22]. Although we found that most soybean *ALDHs* originated from duplication events, we could not predict their functions based solely upon their common ancestors. This was largely due to the diversification of the duplicated *ALDH* gene pairs during evolution. For example, the duplicated gene pairs, *GmALDH2C1*/*GmALDH2C2* and *GmALDH12A1*/*GmALDH12A2*, exhibited distinctly different tissue expression patterns (Fig. 4). These results suggest that the functions of these duplicated gene pairs may have diverged during evolution.

Co-functional networks can provide useful information in identifying genes that are involved in a particular pathway or phenotype with various network algorithms [38, 50, 51]. Investigation of potential co-functional networks associated with a gene family would help understand their functions [52]. In present study, we identified 1146 co-functional genes of 51 *GmALDHs* based on the SoyNet database of co-functional networks for soybean (Additional file 10: Table S5), suggesting *GmALDHs* are widely involved in the co-functional gene networks. Gene Ontology (GO) enrichment analysis (Additional file 11: Figure S6) indicated that the co-functional genes of *GmALDHs* are enriched in proline catabolism (GO: 0006562, FDR **=** 9.37e-05). As a compatible solute, proline is believed to play a role in plant adaptation to drought and salt stresses [53]. Meanwhile, proline may act as a signaling/regulatory molecule to activate multiple adaptive responses [54]. The overexpression of an aldehyde dehydrogenase gene *ALDH21* in tobacco increased the proline accumulation in transgenic plants, and enhanced drought and salt tolerance [55]. In addition, 22 co-functional genes of *GmALDHs* are related to response to water deprivation/water transport (Additional file 10: Table S5). For example, *AtDRIP2*, the orthologous gene of *Glyma.02G141500* in Arabidopsis*,* has been reported to encode a RING E3 ligase and function as a novel negative regulator in drought-responsive gene expression by interacting with AtDREB2A protein [56]. These results suggest the potential role of the *GmALDHs* and their co-functional genes in the regulation of plant drought tolerance.

The functions of *ALDHs* have been extensively studied in other plant species and have been reported to participate in many catabolic and biosynthetic pathways. For example, ALDH2 family members metabolize acetaldehyde generated as a consequence of ethanolic, and ALDH6 family members, functioning as methylmalonyl semialdehyde dehydrogenases, facilitate reactions associated with both valine and pyrimidine catabolism [15]. In addition to their important roles in various metabolic pathways, many plant *ALDHs* have also been reported to respond to a variety of abiotic stresses, including dehydration, high salinity, heat, cold, oxidative stress, and ABA treatment [11, 23, 57]. In our study, the expression patterns of 53 *GmALDHs* in soybean plants subjected to drought stress were analyzed in silico (Fig. 6), using the available RNA-seq data [41]. Results indicated that 13 (24.5%) *GmALDH* genes were significantly up-regulated and 14 (26.4%) were down-regulated in response to water deficit. A large number of the up-regulated *GmALDH* genes were members of the ALDH2 family (46.2%), while most of down-regulated *GmALDH* genes were members of the ALDH2 (42.9%) and ALDH3 (35.7%) families. These results suggest that members of the ALDH2 and ALDH3 families in soybean play roles in the response to drought stress. A wheat ALDH gene, *TraeALDH7B1-5A*, was induced in response to both drought and salt stress, and transgenic Arabidopsis lines over-expressing *TraeALDH7B1-5A* significantly exhibited enhanced tolerance to drought stress and up-regulation of stress-responsive genes [11]. In our study, the expression of *GmALDH7B1* in the roots and leaves was significantly up-regulated in soybean plants subjected to 20% PEG treatment (Fig. 7), which is consistent with the biological function of its corresponding orthologous gene in wheat, *TraeALDH7B1-5A*. Moreover, the gene expression levels of *GmALDH2B1*, *GmALDH2B2*, *GmALDH3H2, GmALDH12A2,* and *GmALDH18B3* were also significantly up-regulated in the leaves of soybean plants treated with 20% PEG (Fig. 7), suggesting that these *ALDH* genes might play potential roles in soybean response to drought stress.

*Cis*-acting regulatory elements play important roles in the transcriptional regulation of genes involved in the response of plants to abiotic stress and phytohormones [43, 58]. Many abiotic stress and phytohormone responsive *cis*-elements, including ABRE, HSE, CRT/DRE, MBS, and TGA-elements, play important roles in plant response to various abiotic stresses [47, 59]. Each *GmALDH* contained at least one *cis*-element related to phytohormone or stress responses. *GmALDH2B1* and *GmALDH2B3* have 10 of the stress and phytohormone responsive *cis*-elements, indicating that these two *GmALDHs* may play important roles in soybean response to stress. This premise is consistent with the expression profiles of these two genes in response to drought stress: *GmALDH2B1* and *GmALDH2B3* were differentially expressed in drought stressed and control plants (Additional file 14: Table S8). The number of drought-responsive *cis*-elements (ABRE, CRT/DRE, and GTGCnTGC/G) present in the promoter regions of soybean *ALDH* genes was also greater in genes that were differentially expressed in response to water deficit as compared to genes that did not exhibit a change in expression (Fig. 8b). Collectively, our results suggest that these three *cis*-elements play important roles in the regulation of soybean *ALDHs* expression in response to drought stress.

## Conclusion

In conclusion, a total of 53 putative *ALDH* genes were identified in the soybean genome and were grouped into 10 families based on a phylogenetic analysis. Gene structures and conserved motifs were more similar within a family than between different families. In comparison to reports on the ALDH superfamily in 18 other species, it appears that the members of the ALDH superfamily in soybean have been greatly expanded, with the majority of *GmALDH* genes underwent WGD/segmental duplications. The co-functional gene networks of 51 *GmALDHs* identified 1146 co-functional links, which are significantly enriched in the process of lipid metabolism, photosynthesis, proline catabolism, and small molecule catabolism. *GmALDH* genes exhibited various expression levels in different soybean tissues. Based upon published transcriptome data on the response of soybean to drought stress, the expression levels of 13 (24.5%) *GmALDH* genes were found to be significantly up-regulated and 14 (26.4%) were down-regulated in response to water deficit. The qRT-PCR analysis revealed that three *GmALDH* genes (*GmALDH3H2, GmALDH12A2* and *GmALDH18B3*) were highly induced by drought stress in soybean leaves. Finally, the numbers of three drought-responsive *cis*-elements (ABRE, CRT/DRE, GTGCnTGC/G) in the promoter regions of up-, down-, and non-regulated *GmALDH* genes were compared. The comparison revealed that a greater number of these three *cis*-elements are present in the promoter regions of *GmALDH* genes that were differentially expressed in response to drought stress than in *ALDH* genes that exhibited no changes in expression. The present study broadens the knowledge base on soybean *ALDHs* and provides a foundation for further investigations pertaining to the functional roles of soybean *ALDH* genes in the response to drought stress.

## Methods

### Identification and characterization of ALDH superfamily in soybean

The soybean (*G. max*) genome (Wm82.a2.v1) from Phytozome v11.0 (http://phytozome.jgi.doe.gov/pz/portal.html) [40] was used to identify *GmALDH* genes. In addition to using the keywords of “ALDH” and “Aldehyde dehydrogenases” to search the Proteome database at Phytozome, the Hidden Markov Model (HMM) profile of the ALDH family domain PF00171 (http://pfam.xfam.org/family/PF00171) [19, 60] was used as a query to perform the blast. All hits with E-values below 0.01 were kept and treated as candidate ALDH and ALDH-like sequences in soybean [22, 61]. Subsequently, BLAST searches using all Arabidopsis ALDHs as queries were also performed. Finally, all candidate sequences were examined to confirm the presence of the conserved ALDH domain (PF00171) using SMART (http://smart.embl-heidelberg.de/) [62, 63], Pfam (http://pfam.xfam.org/) [60], and CDD (Conserved Domain Database) (http://www.ncbi.nlm.nih.gov/Structure/cdd/cdd.shtml) [64]. Likewise, confirmation of the presence of the ALDH cysteine active site (PS00070) and the ALDH glutamic acid active site (PS00687) was also conducted using PROSITE scan (http://prosite.expasy.org/) [65]. The resulting soybean ALDHs were named according to the nomenclature established by the ALDH Gene Nomenclature Committee (AGNC) [31]. Briefly, the protein sequences having a similarity of more than 40% with other previously identified ALDHs were grouped in the same family, while ALDH sequences with more than 60% identity were considered to comprise a subfamily. Sequences sharing less than 40% identity with previously identified ALDHs were grouped into novel families. The identity and similarity of putative soybean ALDH proteins compared to each other and with ALDH proteins from Arabidopsis were calculated by MatGAT v2.02 [66], and the results of these comparisons are presented in Additional file 17: Table S10. The genomic, coding, and putative protein sequences of 53 GmALDHs (Additional file 18) were obtained from Phytozome v11.0 [40]. The molecular weight (Mw) and theoretical isoelectric point (pl) of GmALDH proteins were estimated by ExPASy Compute pI/Mw tool (http://www.expasy.ch/tools/pi_tool.html) [67]. The subcellular localization of each GmALDH was predicted using WoLF PSORT (http://www.genscript.com/wolf-psort.html) [68]. The ALDH sequences from common bean and Medicago were also identified using the same methods as soybean.

### Sequence alignments and phylogenetic analysis of ALDH proteins

The putative amino acid sequences of previously identified ALDHs from *A. thaliana* [14, 15] and *O. sativa* [18, 19], as well as ALDHs from *G. max*, *M. truncatula*, and *P. vulgaris* identified in this study, were obtained from the Phytozome v11.0 database [40]. Multiple sequence alignments were performed using the ClustalW program in BioEditV7.0.5.3 [69]. The alignment results were used to construct a phylogenetic tree with MEGA 5.2 [70] based on the neighbor-joining (NJ) method [71] using 1000 bootstraps and the pair-wise option.

### Analysis of gene structures and protein motifs

The diagrams of exon-intron structures for *GmALDH* genes were constructed using the Gene Structure Display Server (GSDS: http://gsds.cbi.pku.edu.cn/) online tool [32]. Conserved motifs of putative GmALDH proteins were predicted with Multiple EM for Motif Elicitation (MEME: http://meme-suite.org/) [33] using the default settings for motif width (between 6 and 50 wide) and site distribution (zero or one occurrence per sequence). The maximum number of motifs was set as 10. The presence of the conserved GmALDH motifs in the other four plant species was analyzed using Motif Alignment & Search Tool (MAST: http://meme-suite.org/tools/mast) [33] with the default setting of *p*-value less than 0.0001.

### Chromosomal distribution and duplication analysis of soybean *ALDH* genes

The physical positions of *GmALDH* genes on soybean chromosomes were obtained from SoyBase (http://soybase.org/) [72]. The distribution of the 53 *GmALDH* genes on 20 soybean chromosomes was visualized using MapChart [35]. The Multiple Collinearity Scan toolkit (MCScanX) [73] was used to identify the duplication events that occurred in *GmALDH* genes in soybean genome. In brief, BLASTP was preformed to identify the intra-species paralogous pairs using protein sequences with the following parameters settings: alignment significance: E_VALUE (default: 1e-05); MATCH_SCORE: final score (default: 50); MATCH_SIZE: number of genes required to call a collinear block (default: 5) and the maximum gaps (default: 25). Soybean *ALDH* genes falling in the identified collinear blocks were considered as WGD/ segmental duplication events, while closely adjacent (no more than one gene separating them) homologous *ALDH* genes were considered to represent tandem duplication events, based on the identification standards in MCScanX [73]. Moreover, the WGD/segmental duplications identified by MCScanX were future verified by the PGDD (http://chibba.agtec.uga.edu/duplication/) [74]. The number of synonymous substitutions (*K*s) per site for the *GmALDH* gene pairs from WGD/segmental duplication was calculated using the MCScanX program [73]. The *K*s values were used to estimate the duplication time, which was calculated as *K*s/(2 × 6.1 × 10^−9^) × 10^−6^ Mya, based on a rate of 6.1 × 10^−9^ substitutions per site per year [75]. Finally, the syntenic relationships of soybean *ALDH* genes were illustrated with CIRCOS [76].

### Co-functional gene network analysis

The putative co-functional genes of *GmALDHs* were investigated using SoyNet (http://www.inetbio.org/soynet/), which is a database of co-functional gene networks for soybean [38]. The network was developed by wiring 40,812 soybean genes (~73% of the coding genome) with 1,940,284 co-functional links, which were inferred by Bayesian statistics framework [38, 77]. The putative co-functional gene pairs were obtained by estimating the log likelihood score (*LLS*) between each pair of genes, and scores greater than zero indicate the paired genes might be co-functional, with higher scores indicating more confident linkages and stronger support for the co-functional relationship. The co-functional networks were drawn using the Cytoscape software [78]. The putative co-functional genes were subjected to Gene Ontology (GO) functional analysis using Singular Enrichment Analysis (SEA) method by agriGO tool (http://bioinfo.cau.edu.cn/agriGO/analysis.php) [79]. The newest soybean genome Wm82.a2.v1 was set as background, and the significantly enriched GO terms for the putative co-functional genes of *GmALDHs* were determined using hypergeometric tests, with the Bonferroni-corrected *P*-value ≤0.01 and FDR ≤ 0.01 as the thresholds, respectively [80, 81].

### Putative *cis*-elements in the promoter regions of soybean *ALDHs*

The 1500 bp sequences (Additional file 19) upstream from the translation start codon of all of the *GmALDH* genes were obtained from Phytozome v11.0 [40]. The putative stress or hormone responsive *cis*-acting regulatory elements in these sequences were predicted using the Plant CARE online database (http://bioinformatics.psb.ugent.be/webtools/plantcare/html/) [46].

### Analysis of *GmALDH* gene expression in different soybean tissues

The expression levels of all 53 *GmALDH* genes in eight soybean tissues were obtained from the RNA-seq data (FPKM values) at Phytozome v11.0 [40]. A heat map with hierarchical clustering of *GmALDH* genes was constructed using MeV 4.9 software [82, 83] by average linkage with Euclidean distance method, to visualize the expression levels in eight tissues based on the log10 (FPKM + 1) values of *GmALDH* genes.

The tissue expression patterns of six randomly selected *GmALDH* genes were confirmed using qRT-PCR. Seeds of the soybean variety, Kefeng-1 (KF-1), provided by the National Center for Soybean Improvement, Nanjing, China, were germinated and grown for three days in moist sterile sand. Root tips (0–2 cm) were then harvested while other seedlings were transferred to a growth chamber under a 14/10 h photoperiod (26/24 °C) and 60% relative humidity. Shoot apical meristems (SAM) from V2 stage plants, the youngest trifoliate leaves, stems, flowers from the R2 stage plants, and pods from R4 stage plants were harvested and immediately frozen in liquid nitrogen and stored at −80 °C. The experiment was performed in triplicates.

### RNA-Seq data analysis, drought stress treatment, and qRT-PCR

For the in silico analysis of the response of *GmALDHs* to drought stress, RNA-Seq clean reads (three biological replicates) from a drought-tolerant soybean cultivar, PI416937, that had been subjected to drought treatment (exposed to air) and sampled at 0, 6, 12 and 24 h, were downloaded from the SRA database (BioProject accession: PRJNA259941) [41]. Mapping of the obtained transcriptome reads to the reference soybean genome (Wm82.a2.v1) was conducted using TopHat2 (v2.0.13) [84]. FPKM values were estimated using Cufflinks software [42], and Cuffdiff (FDR ≤ 0.05) was used to find differentially expressed genes (DEGs) for the different sample comparisons. The log10 (FPKM + 1) values of the 53 *GmALDH* genes were used to analyze their expression patterns in soybean plants subjected to drought stress.

To experimentally investigate the expression of *GmALDH* genes in soybean plants subjected to drought treatment, the soybean cultivar KF-1 was used. Soybean seeds sterilized in 1% NaClO for 1 min, followed by three washes in distilled water, were germinated in the dark for 4 days at 24 °C and 60% humidity. The seedlings were then transferred to plastic boxes and cultured hydroponically in half-strength Hoagland nutrient solution (pH = 5.8) in a growth chamber at 26 °C/24 °C (day/night), 60% relative humidity, and 14 h/10 h (light/dark) photoperiod. The half-strength Hoagland solution was replaced every two days. In order to simulate a drought stress, two-week-old soybean seedlings (V2) were placed in plastic boxes containing half-strength Hoagland solution containing 20% PEG6000. Both root-tips (0–2 cm) and youngest trifoliate leaves were collected at 0, 3, 6, 12, 24, 48 and 72 h, and immediately frozen in liquid nitrogen and stored at −80 °C until further use. Three biological replicates were obtained for each time point.

Total RNA was extracted using TRIzol reagent (Invitrogen, USA) according to the manufacturer’s protocol. First-strand cDNA was synthesized from 1 μg of total RNA using a PrimeScript 1st Strand cDNA Synthesis Kit (TaKaRa, Japan) following the manufacturer’s instruction. The gene specific primers were designed using primer premier 5.0 (Premier Biosoft International, USA) software and synthesized by Invitrogen (Shanghai, China). The amplification efficiencies (E) of qRT-PCR were estimated by the calibration curves using a series of dilutions of cDNA, according to the equation: E = [10^–1/slope^]-1 [85]. The primer sequences and amplification efficiencies of qRT-PCR reactions were shown in Additional file 20: Table S11. The specificity of the amplification was verified by the melting curve (Additional file 21: Figure S7). qRT-PCR was performed on a Roche 480 real time detection system (Roche Diagnostics, Switzerland) following the manufacturer’s instruction. Each qRT-PCR reaction contained 2 μl cDNA, 7.5 μl 2X SYBR Premix Ex Taq (TaKaRa, Japan), and 0.4 μl of each forward and reverse primers in a final volume of 15 μl. The amplification program was set as follows: initial denaturation at 95 °C for 5 min, 40 cycles of denaturation at 95 °C for 10 s, annealing at 58 °C for 30 s and extension at 72 °C for 20 s. The relative gene expression level was calculated using the 2^-ΔΔCT^ method [86]. To determine the relative expression levels of *ALDH* genes in different soybean tissues, the expression level of each gene in stem was set as “1”. The relative expression levels of *ALDH* genes in the leaves and roots of soybean plants at 3, 6, 12, 24, 48 and 72 h after exposed to 20% PEG treatment were compared with the gene expression at 0 h. The soybean *60S* gene was used as the internal control. Origin v8.6 software was used to graph the results of the qRT-PCR analyses.

## Additional files

Additional file 1: Table S1. Soybean *ALDH* genes and their encoded proteins. (XLSX 15 kb)

Additional file 2: Table S2. Identification of PF00171, PS00687 and PS00070 domains in soybean ALDHs using HMMER 3.0 software. (XLSX 13 kb)

Additional file 3: Figure S1. Distribution of ALDH families (1–24) in 19 species. The phylogenetic tree on the left, based on the taxonomic identifications of the species, was generated using the Taxonomy Common Tree Tools on the NCBI website (http://www.ncbi.nlm.nih.gov/guide/taxonomy/). The names of the ALDH families are listed on the top of the table. The references are as follows: A, He et al. [21]; B, Brocker et al. [15]; C, Zhang et al. [17]; D, Chen et al. [22]; and E, Li et al. [34]. + and − represent presence or absence, respectively. (TIFF 3236 kb)

Additional file 4: Table S3. Multiple alignments of ALDH protein sequences from *P. vulgaris* (Pv), *M. truncatula* (Mt), *A. thaliana* (At), and *O. sativa* (Os). (XLSX 36 kb)

Additional file 5: Figure S2. Phylogenetic tree of soybean ALDH superfamily. The tree was constructed using MEGA 5.2 based on the Neighbor-joining (NJ) method. Bootstrap values in percentage (1000 replicates) are labeled on the nodes. (TIFF 424 kb)

Additional file 6: Figure S3. A. Sequence logos of the conserved motifs identified in GmALDH proteins. B. Presence of the conserved motifs in the ALDH proteins from soybean, common bean, Medicago, Arabidopsis and rice. (TIFF 4489 kb)

Additional file 7: Figure S4. Chromosomal distribution and tandem duplications of soybean *ALDHs*. The 53 *ALDHs* were mapped onto soybean chromosomes based on their physical positions. Five tandemly duplicated gene-pairs are labeled by orange boxes. The scale on the left is in megabase (Mb). (TIFF 2182 kb)

Additional file 8: Table S4. Estimated time of the WGD/segmental duplication events among soybean *GmALDH* genes. (XLSX 13 kb)

Additional file 9: Figure S5. The co-functional gene networks of *GmALDHs* in soybean. The putative co-functional genes of *GmALDHs* and their co-functional links were identified using SoyNet. The gene networks were drawn using the Cytoscape software. *GmALDHs* were marked in red and non-*GmALDH* soybean genes were marked in blue (except that the 22 non-*GmALDH* genes related to response to water deprivation/water transport were marked in green). (TIFF 7097 kb)

Additional file 10: Table S5. Detailed information of putative co-functional genes of *GmALDHs*. (XLSX 92 kb)

Additional file 11: Figure S6. Gene Ontology (GO) enrichment analysis of the co-functional genes of *GmALDHs*. (PNG 244 kb)

Additional file 12: Table S6. FPKM values of *GmALDH* genes in eight different tissues of soybean. Values obtained from Phytozome v11.0. (XLSX 15 kb)

Additional file 13: Table S7. FPKM values of *GmALDH* genes in the drought-tolerant soybean variety PI416937 under control and drought stress conditions. (XLSX 14 kb)

Additional file 14: Table S8. Fold change and *p*-value of *GmALDH* genes in the drought-tolerant soybean variety PI416937 under drought stress compared with control. (XLSX 13 kb)

Additional file 15: Details of the *cis*-elements identified in this study. (DOCX 14 kb)

Additional file 16: Table S9. Hormone- and stress-responsive *cis*-elements present in the 1500 bp upstream region of the 53 *GmALDH* genes. (XLSX 27 kb)

Additional file 17: Table S10. Identity and similarity values of GmALDH proteins compared to each other and to ALDHs from Arabidopsis and three other ALDH proteins. (XLSX 39 kb)

Additional file 18: Genomic, coding, and protein sequences of the 53 soybean ALDHs. (DOCX 226 kb)

Additional file 19: The 1500 bp upstream sequences of the 53 *GmALDH* genes. (DOC 111 kb)

Additional file 20: Table S11. The primer sequences and amplification efficiencies for qRT-PCR in this study. (XLSX 10 kb)

Additional file 21: Figure S7. The specificity of the amplification for qRT-PCR in this study. (TIFF 7637 kb)

## Abbreviations

ABRE: ABA-responsive element
ALDHs: Aldehyde dehydrogenases
CRT/DRE: C-repeat (CRT)/dehydration-responsive element (DRE)
FPKM: Fragments per kilobase per million mapped fragments
GO: Gene Ontology
SAM: Shoot apical meristem
