# Supplementary figures and images for: Genome-wide characterization of the aldehyde dehydrogenase gene superfamily in soybean and its potential role in drought stress response
Source: BMC Genomics. 2017 Jul 7;18:518. doi: 10.1186/s12864-017-3908-y (PMC5501352; doi:10.1186/s12864-017-3908-y)

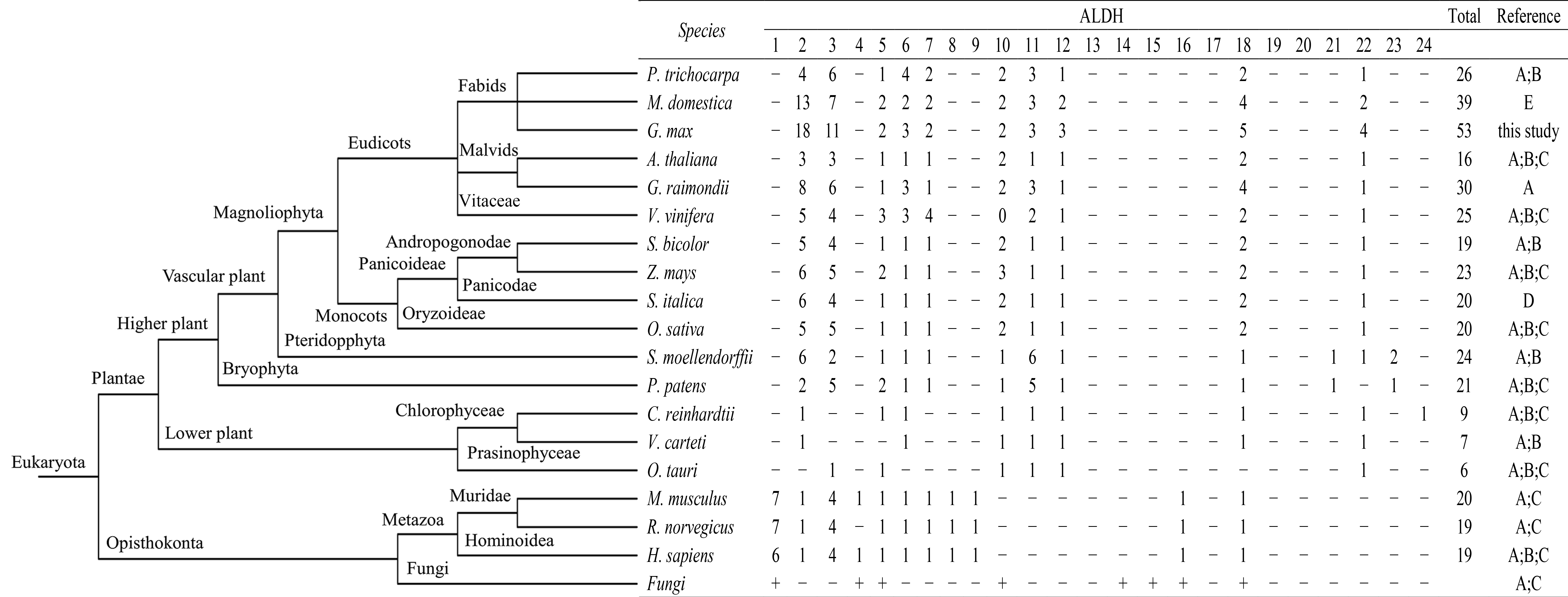

Supplement: Supplementary file 3 — Distribution of ALDH families (1–24) in 19 species. The phylogenetic tree on the left, based on the taxonomic identifications of the species, was generated using the Taxonomy Common Tree Tools on the NCBI website (http://www.ncbi.nlm.nih.gov/guide/taxonomy/). The names of the ALDH families are listed on the top of the table. The references are as follows: A, He et al. [21]; B, Brocker et al. [15]; C, Zhang et al. [17]; D, Chen et al. [22]; and E, Li et al. [34]. + and − represent presence or absence, respectively. (TIFF 3236 kb) [file 12864_2017_3908_MOESM3_ESM.tiff]

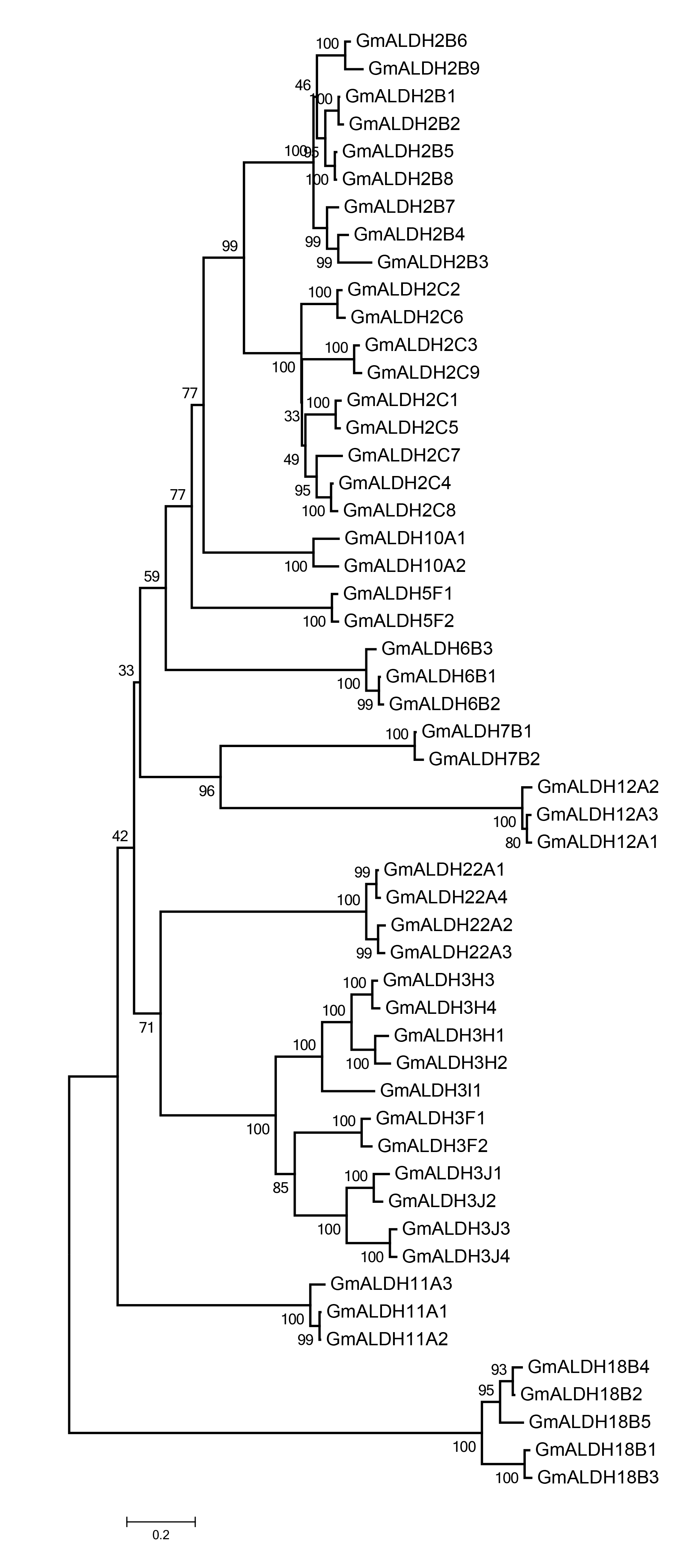

Supplement: Supplementary file 5 — Phylogenetic tree of soybean ALDH superfamily. The tree was constructed using MEGA 5.2 based on the Neighbor-joining (NJ) method. Bootstrap values in percentage (1000 replicates) are labeled on the nodes. (TIFF 424 kb) [file 12864_2017_3908_MOESM5_ESM.tiff]

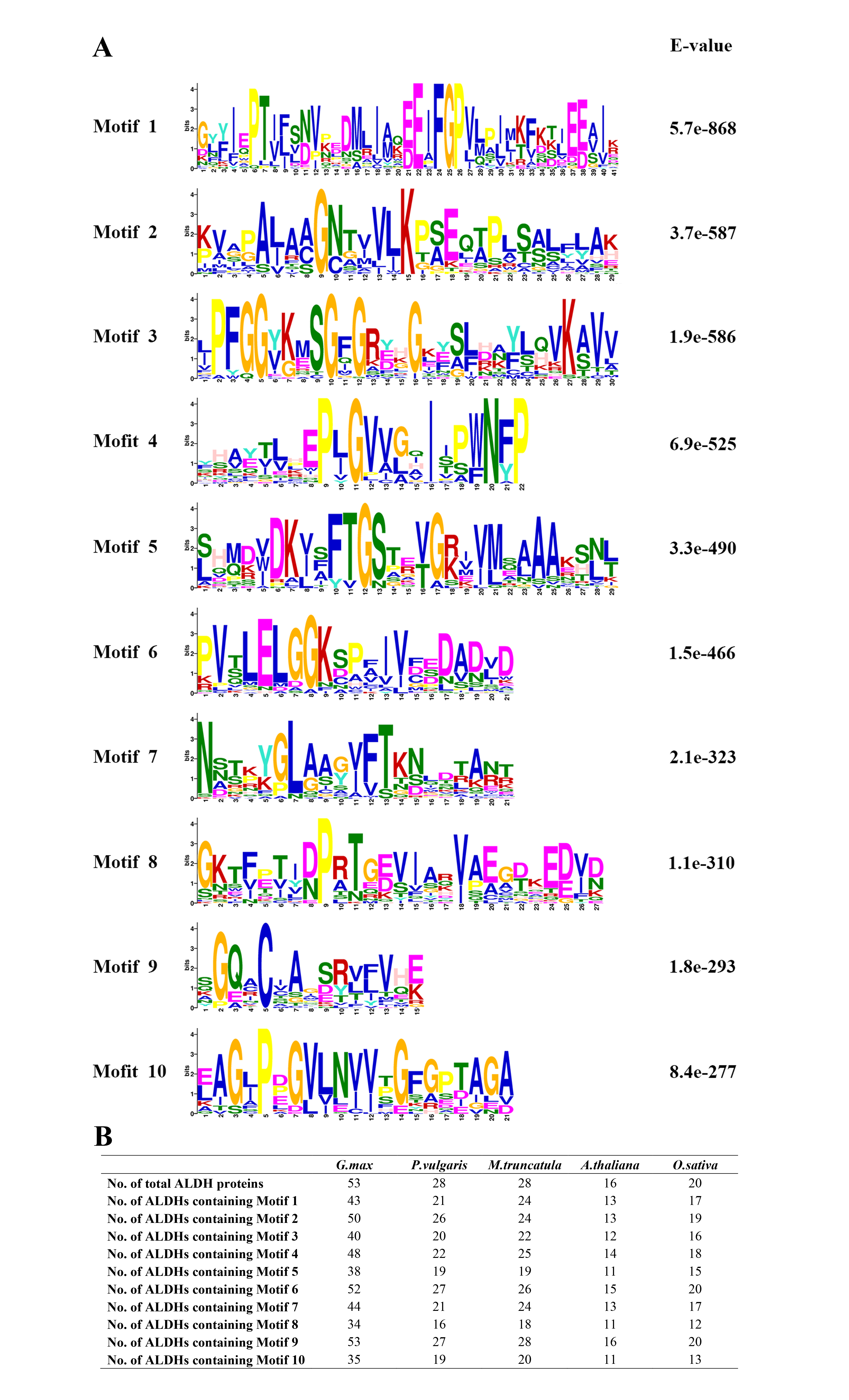

Supplement: Supplementary file 6 — A. Sequence logos of the conserved motifs identified in GmALDH proteins. B. Presence of the conserved motifs in the ALDH proteins from soybean, common bean, Medicago, Arabidopsis and rice. (TIFF 4489 kb) [file 12864_2017_3908_MOESM6_ESM.tiff]

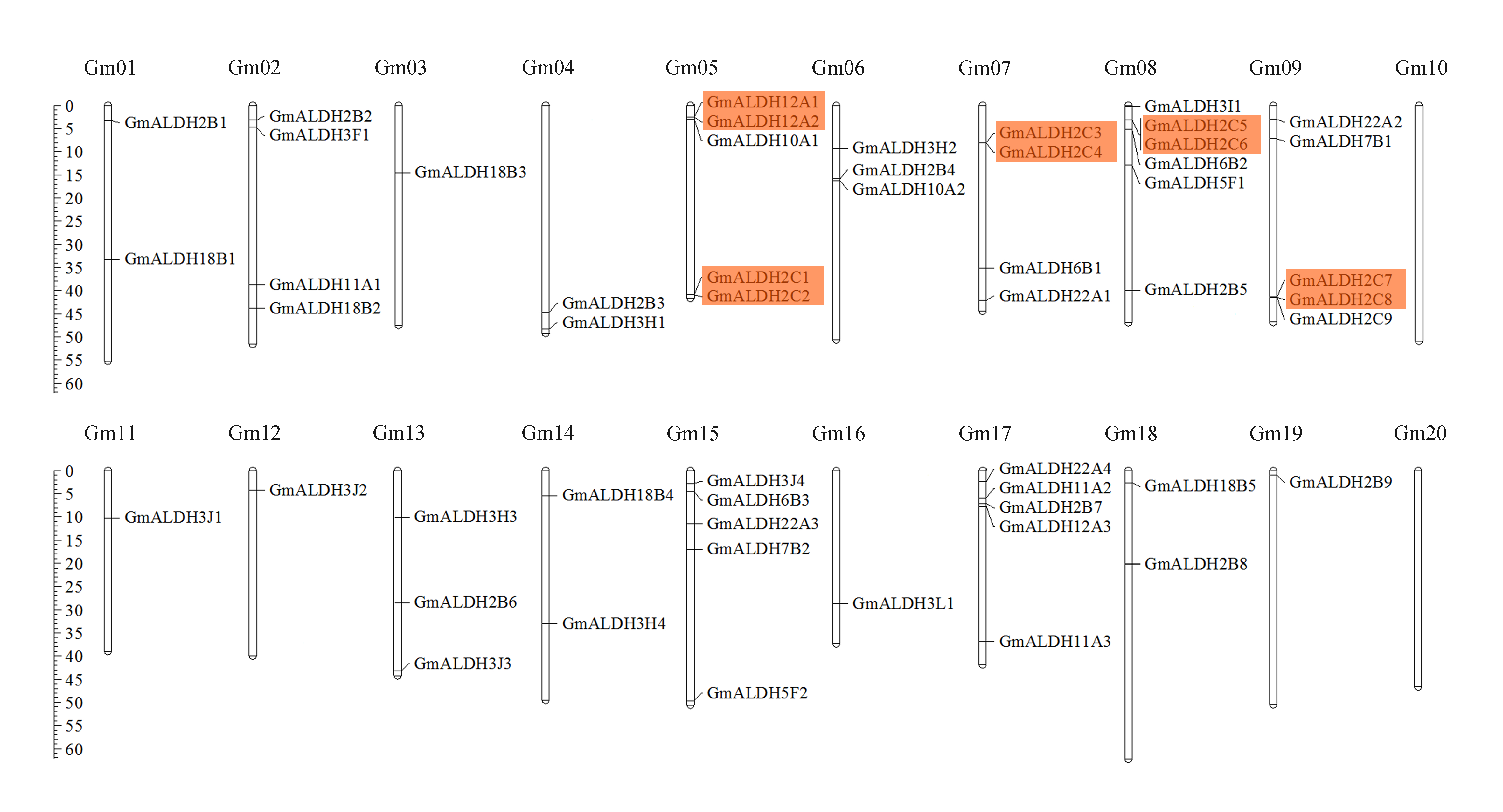

Supplement: Supplementary file 7 — Chromosomal distribution and tandem duplications of soybean ALDHs. The 53 ALDHs were mapped onto soybean chromosomes based on their physical positions. Five tandemly duplicated gene-pairs are labeled by orange boxes. The scale on the left is in megabase (Mb). (TIFF 2182 kb) [file 12864_2017_3908_MOESM7_ESM.tiff]

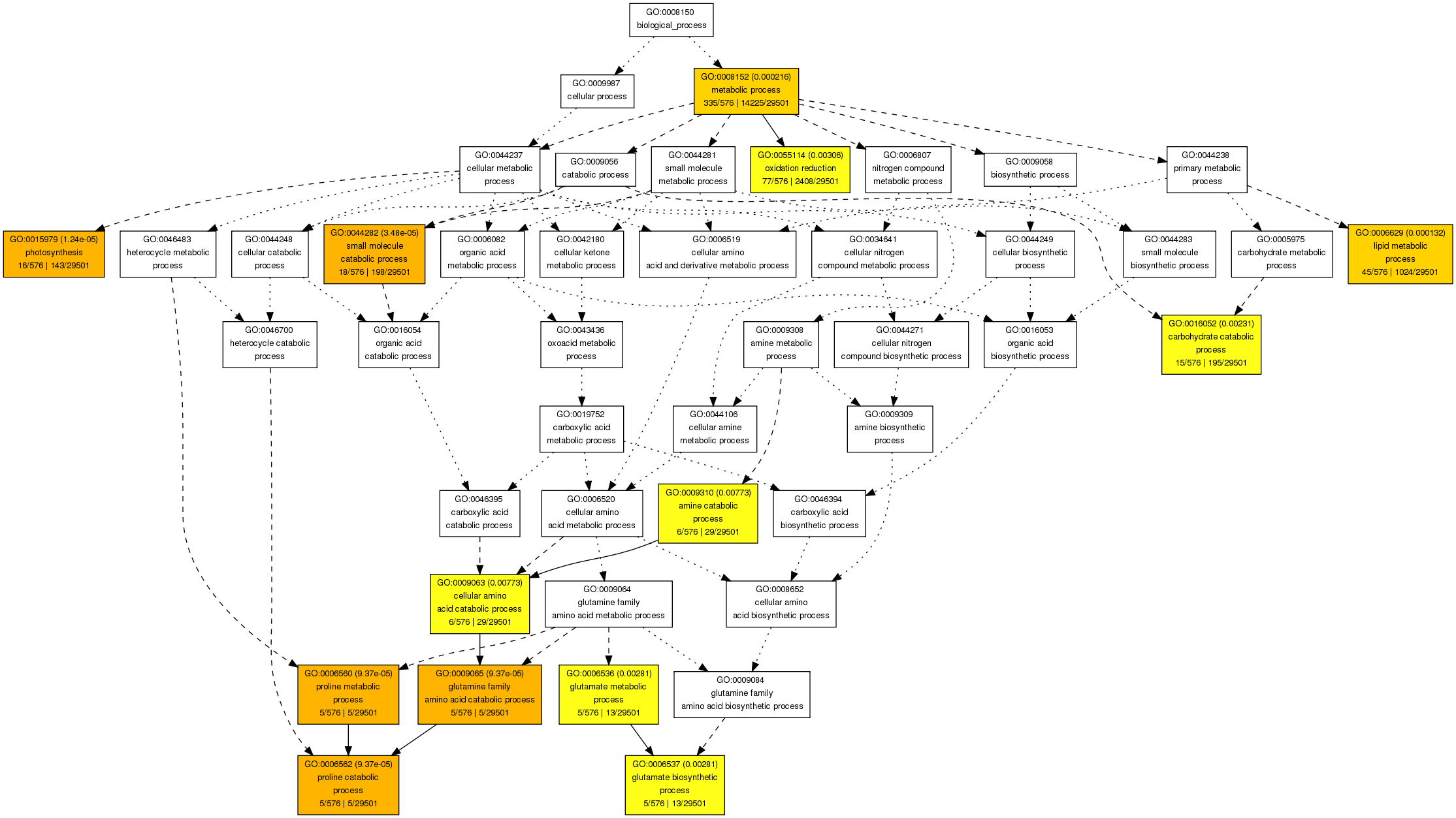

Supplement: Supplementary file 11 — Gene Ontology (GO) enrichment analysis of the co-functional genes of GmALDHs. (PNG 244 kb) [file 12864_2017_3908_MOESM11_ESM.png]
