# Supplementary material for: Genome-wide characterization of the aldehyde dehydrogenase gene superfamily in soybean and its potential role in drought stress response
Source: BMC Genomics. 2017 Jul 7;18:518. doi: 10.1186/s12864-017-3908-y (PMC5501352; doi:10.1186/s12864-017-3908-y)
Supplement: Supplementary file 15 — Details of the cis-elements identified in this study. (DOCX 14 kb) [file 12864_2017_3908_MOESM15_ESM.docx]

**Additional file 15. Details of the *cis*-elements identified in this study.**

| Stress-responsive | ARE | *cis*-acting regulatory element essential for the anaerobic induction |
| --- | --- | --- |
|  | Box-W1 | fungal elicitor responsive element |
|  | HSE | *cis*-acting element involved in heat stress responsiveness |
|  | MBS | MYB binding site involved in drought-inducibility |
|  | TC-rich repeats | *cis*-acting element involved in defense and stress responsiveness |
| Hormone-responsive | ABRE | *cis*-acting element involved in the abscisic acid responsiveness |
|  | CGTCA-motif | *cis*-acting regulatory element involved in the MeJA-responsiveness |
|  | ERE | ethylene-responsive element |
|  | GARE-motif | gibberellin-responsive element |
|  | P-box | gibberellin-responsive element |
|  | TATC-box | *cis*-acting element involved in gibberellin-responsiveness |
|  | TCA-element | *cis*-acting element involved in salicylic acid responsiveness |
|  | TGACG-motif | *cis*-acting regulatory element involved in the MeJA-responsiveness |
|  | TGA-element | auxin-responsive element |
