# Supplementary material for: Genome-wide characterization of the aldehyde dehydrogenase gene superfamily in soybean and its potential role in drought stress response
Source: BMC Genomics. 2017 Jul 7;18:518. doi: 10.1186/s12864-017-3908-y (PMC5501352; doi:10.1186/s12864-017-3908-y)
Supplement: Supplementary file 18 — Genomic, coding, and protein sequences of the 53 soybean ALDHs. (DOCX 226 kb) [file 12864_2017_3908_MOESM18_ESM.docx]

**Additional file 18. Genomic, coding, and protein sequences of the 53 soybean ALDHs.**

**Note:** The genomic sequences are listed on pages 1-162, the coding sequences are listed on pages 162-203, and the protein sequences are listed on pages 203-219

**The genomic sequences of 53 soybean *ALDH* genes**

>Glyma.01G031500 | Chr01:3278502..3282846 reverse

TTGGTTAGAGGGTTGATTTCCCATTTGACCGTAACTATATAATCTTGGAAGAATTTCACAAAGTTCATAAAACTGGACTATAAACAGTAGTGGAGAGAGG

GAGAGAAACCAAAGAAGAAGATTGACCATGGCTTCATCAATGAGGATCTCAAGGCTGCTCTCTCGTTCTTTTCTTTCTGCTTCCACTACTCCTTTGTTCT

CTAGAGGTACTTATTGCTTATCATCACTTCCAAGATCCTGGGGTGATTGATGATTTGATTACTTTTTCTGCTTGGTTCTGAGAACATGTAGAGCATGAAA

ATGAATTGATTTTCAATTTTGTATTTTTCTTATTTGATTAACTGGGTTTGTGGGGGATTTGGATTATAGGATCATTTTCTGATCCTTAGGATATAATAGT

TACCTATTTGTTTGGAGATGGTTGGATTGATCAGCTGTTATTTTTTTTGTAAAGAGAGAAAATGAAATGTGTTATAAAAAATTATATGATCATTGACCAA

GTGGGGGAGAGAGGATATAAAAAGTTTGAAACTTTAAAAGGATCCACAAGTGAAAGTGCTATTTGTGGTGAGGCATTCTGAACTGTCCTTTTTTGTCTGG

CCCCTGTTCCTTTTCAGCTACCACCTGTTTCTTTCACAGTTTCACTGCCCCTTCAAGTACATGTTATTACTTTAGTGTATAAATTGGAGGCTACTTCCTG

GGACCATTTCAATTTATGTCACATGCCAAGATGATGAATGATCATACCTTTTGATTGGTGTTTTTAGGCCTTGGTTTTGCTTTGGGGCTTTCATTAATGG

TCTTATTTATTTTTAATAAATTGGCCCCTTTTTTCTTCTTCTGATATTCAGGTGGAAGTGGTGCTCTTGGTGCAGGGCTCAGTAAATTCAGCACTGCTGC

TGCAATTGAAGAACCAATCAAACCACCAGTTAAAGTAGAACACACCCAGCTCTTAATCGATGGAAAATTCGTTGATGCTGCTACTGGTTAGTCCTTTTTT

TTTCACCCTAGTTTTATCCCCATCTATAAAGTGTGTGGTTGGGTTAGCATTTGCAAATTTAATTTTGAATGAAGTTGATTTTACAAAATTGACTTTGATT

GAAATTGAGTTTGATGGGACATGATTTATATTTGAATGTTTTTATTCTGAAAGTAAGTTAGTAGAAAAATTCAACATACATTTTTGTTATGTAATGCAAA

AATCAATTTTGGATCCTCAATTGTTTGCCTAAGATCATTTTAGCTAGTATTTCAACATGACTTTGGATAATCTATGCGAATCCAAACACCATTTGCACAC

AAATTAAGCATTTTAAGGTGGTCATAACTTACTGTTGTGATTCCCCTGACATCAGGCAAAACTTTTCCAACGTTGGACCCCAGGACAGGAGATGTGATTT

CTCATGTTGCTGAGGGTGACCATGAAGATGTTGATCGAGCAGTTGCAGCTGCTCGCAAAGCCTTCGACCATGGACCTTGGCCAAAGATGACAGCCTATGT

ATTTTCACCCTTCTAATTGTTCTTGAACAATGTACAAAATCCATGGTCTTTGGTCCCATGATCACTCAACTAATTCCTTGTTTTGTTTGCTTGTTGTGTA

ACCATGTAGGAAAGACAAAGGATATTGCTGCGTGCTGCTGATCTGTTTGAAAAGCACAATGATGAGCTTGCTGCTCTTGAGACTTGGGATAATGGGAAGC

CATATGAACAATCTGCTCAAATCGAAATTCCAATGCTGGTTCGCCTATTTCGATACTATGCTGGTATGTCGAAATGAGAAACACTAACAACACACTCTTA

TAAATAAATTCTTTGTTATTGATTGAAATTTATTGAAAATCACAAATTTTTTGTAGGTTTCACTTCTCATTTAATGAGTTCCACTCAATTTTATAATTGT

AGATAGTATGTTAGAACAAGTGTGTTATAGAGTGTGTAATTAACGTTACTCATGCAGAAATAACTTGTCTGATTGTCTCTTTGGTGTTCAATGAAACTAG

TGCTAACACTTCTAATTCCGAATTGCTCAGGTTGGGCAGATAAGATTCATGGTCTCACAGTTCCAGCTGATGGGCCATATCATGTGCAAACATTGCATGA

ACCCATTGGTGTTGCTGGTCAGATCATTCCGTGGAACTTCCCTCTTGTCATGTTTGCCTGGAAGGTTGGGCCAGCATTGGCCTGTGGTAACACCATTGTT

CTCAAAACAGCTGAGCAGACTCCATTATCTGCTTTGTATGCATCAAAGTTGCTTCATGAGGTTTGTCACATTCCATGCTTTATTTTAAAAGGAGATTGCT

TTTAGTTTGTTTTGTTGAAAGAAATGGGAAGATTTGTATATATATGTTGAATGGTCATACCAAATTTATCATGTCTGGCAGGCTGGACTTCCTCCTGGCG

TTCTGAATGTAATATCTGGCTTTGGTCCTACCGCTGGTGCTGCCATTGCTAGTCACATGGACATAGATAAGGTATTGAGATTTTTAGTTTTCCATTAGTA

TTCATTTTCACCCCTCGTCGATCCTTTTTACCCTCTGCTATTCACTTGTTTCCTCAACATTGGCAAATCCCTTTTAGTGAAACAGTGTGAGGATTTTTTG

ATGGTGGAACCATGGAAGAAATAAGTTTCTTGTGTAGTCACAAAATAGAAGCTTAGAAAGTGTGTGTATATACTGAAGTCTTTGCTTTGTTGAACTTTAT

TGCAGCTTGCTTTCACTGGTTCAACCGAAACTGGAAAAGTTGTCCTTGAATTGGCAGCTAGAAGTAATCTTAAGCCTGTTACTCTGGAGCTTGGTGGGAA

ATCTCCTTTTATAGTATGCGAAGATGCTGATGTAGATGAGGCTGTTGAGCTAGCACACTTTGCATTATTCTTTAATCAGGTACATCCAAATATAAAGTTT

GTCTTGTAAAATAATTGTCTTTCATAAGGTTCAGAAACAAGCTAATTGATGTTTTTTGTGACATTCAGGGACAATGCTGCTGTGCTGGGTCTAGAACATT

TGTACACGAACGTGTGTACGATGAATTTATTGAGAAAGCAAAGGCACGTGCTTTGAAGCGTGCTGTTGGTGATCCATTCAAGGGGGGAATAGAGCAAGGT

CCTCAGGTAACATACCCATACGTTTAGTATCAAAATGCAGAGAAAATATTGCACTAATTTGATATTTCCTTACATGTTTCAAAATGTCTGCTCTATTGAT

GATTTTGTGTATGATCCAAAAATCAGATTGATTCAGAACAATTTCAGAAAATACTGAAGTATATTAGGTCCGGTGTTGAAAGTGGAGCTACTCTTGAAAC

TGGAGGAGATAGATTTGGCAACTCAGGCTTCTATATTCAGCCTACTGTCTTCTCAAATGTTAAGGTATAATTATAATAAGAGGAATGCTAGCAACACACT

CCCTACTAGAAACTTTTTGCAATTTTCGATATATTGTAGTGAATAATAAAGTGTGTTTAAAAGATTGTTATGTAGTTCTTGCTAGCATTTCTCTAATAAT

AATAATTTTTAATTGCACTCATTGTATCAAGGATGCTTAAAATGTGAATTCTTGTGTTACTAATTGTAAAAACAACCAGGATGACATGCTGATTGCAAAG

GAAGAGATCTTTGGTCCAGTGCAAACCATATTAAAATTCAAGTGAGAACAGTGACTCCTATTGGCAAATAATATCATTATCTTTGCACTTTTGCTTCAAA

CACTAATTATCTTTTGTGTTTGATCACAGGGACCTTGATGATGTGATTCAAAGAGCAAATAACACACACTATGGCCTTGCAGCAGGGGTGTTCACAAAAA

ACATTAACACAGCTAACACTTTGACTAGAGCATTGCGAGTTGGAACAGTGTGGATTAACTGCTTTGATACATTTGATGCAGCAATTCCCTTTGGAGGGTA

CAAAATGAGTGGCCAAGGAAGAGAAAAAGGAGAATACAGTCTCAAGAACTACTTGCAAGTGAAGGCTGTTGTTACATCCTTGAAGAACCCTGCTTGGCTT

TGAACATCATTAGCTTTAGATTTATTTGATGAAAAAGATTAATAAATAGGCTCCAATAATAAAGATAATTAAATTGGGTTTATTCCATTCAAAGTATCTG

ATAATGATGAATATGATCTATTTTCTTTTTCTGTTTTTGCCCCTGCATTTTATGGGTAGCGCGAAATATACTATGGTGTAAAATTCATCGTAGAGGTGAA

TTTCTGGTTGGAATGGGAATCAAATTTGCATAATGATGGATGTAAACTTCATATTACTTGTTCTCAGAGGACATTGTTCGTTTTTAGTAAATGGCAGTGT

TATGATTGAACTGTGATTCAACTAACAACGATTCAGTATCATATT

>Glyma.02G034000 | Chr02:3157712..3162381 forward

TTCATAAAGTGACATATATTTTGTACCTATAACTTGATTGGAAAAAGAACACCTATTATGGAAGGTGTGCTGAGCAGAGATGTCAACGAATTATGGTTGC

TCATATGTTAATGTCACATTATTTCTGGGGTGATTGATAACAACATTTAAGACTTCGTTCACAATGGGGTAAAGGGTCAGTTTCCCATTTGACTGCAACT

ATATAACACTCGTAACAATTTCACAAAGTTCATAAAACTTTACTATAAACAGTAGTGGAGAGAGGGAGAAAAACCAAAGAAGATTGACCATGGCTTCTTC

ATTGAGGATCTCAAGGCTCCTCTCTCGTTCTTTTCTTTCTGCTTCCACTACTACTCCTTTGTTTTCTAGAGGTACTTGCTGTTTATCATCACTTCCAAGA

TTTTGGGGTGGTGATCTTGTTATTTTTTCTGCTTGGTTCTTAAGGACACGTAGAACATGAAAATGAAGTGACTCAATTTTGCACTTTTTTTTATTTGATT

AACTTGGTTTGTGAGGGATTTGAATGATAGGATCATTTTTTATTTTAGGAATGTTTTTTATATTGGCAAATGTTACTTTGTTATTTTTGTTAAAAATATC

ACTGAGATATTCTGAGAATTCAAACTCGCAACCTTTCTTCCTTTGTTTTTAAGCTTCTTTTCCATCCATTAGACCAACCTTATATCTACTCTTATCCTTA

GGAATGATAGTTACCAATTTGTTTGGAGATGGTTGGATTGATCAGTTTTTTTTTTTTTTTGTGAAGAGAGAAAATGAGGGGTGCCATAAAAAATTATATG

ATCATTGACCAAGTGAGGGGGAGAGGGATATAAAAAGCTTGGAACTTTAAAAGGATCCATAACTAAGTGCTATTTACGGTGAGGCATTATGAAGTCTGAA

CTGTACTTTATTGGCTGGCCCCTGTTCCTTTTCAGCTACCACCTGTTTTTTTCACAGTTTCACTGCCCTTTCAAGTACATGTTTTTTCTTTCACAATTGG

AGGCTACTTTCTGGGACCATTTCAATATATGCCCCCTGCCAAGATGATGAATGATCATGCCTTTTGATTGGTGTTTTTAGGCTTTGCTTTTGCTTTGGGG

CTTTCATTAATGGTCTTATTTTTTTTAATAAATTGACCCTTTCTTCTGATATTCAGGCGGAAGTGGTGCTCTTGGTGCAGGGCTCAGTAAATTCAGCACT

GCTGCTGCCATTGAAGAACCAATTAAACCACCACTTAAAGTTGAACACACCCAGCTCTTAATTGATGGAAAATTTGTTGATGCTGCTACTGGTTAGTCCT

TTTTTTTTCTCCCTTCATAGTTTTATCCCCATCTATAAAGTGTGTGGTTGGGTTAGCATTTGCAAATTTAATTTTGAATGAAATTAATTTTACAAAATTG

ACTTTGATGGAAATTGAGTTTGAAGTGATGCAATTTATATTTGGATGTTTTTATTCCAAAAGTCAACATAATTTTTTGTTATTTAATGCAAAAATCAATT

CTGGATCCTCAATTGTTTTACCTAAAATCACGTTAGCTAGTATTTCAACATGACTTGGGATAATCTATGCAAATCCAAACACGTTATTCATCATTTGCAC

AAAAATTAAGCATTTTAAGGTGGTCATAACTTGCTGTTGACATCAGGCAAAACTTTTCCAACGTTGGACCCCAGGACAGGAGATGTGATTTCTCATGTTG

CTGAGGGTGACCATGAAGATGTTGATCGAGCAGTTGCAGCTGCTCGCAAAGCCTTCGACCGCGGACCTTGGCCAAAGATGACAGCCTATGTATTTCCACC

CTTCTAATTGTTCTTGAATAATGTGCAAAGTCCATGGTCTTTGGTCCCTTGATTACTCAACTAAATCCTTGTTTTGTTTGCTTGTTGTGTAACCGTGTAG

GAAAGACAAAGGATATTGCTGCGCGCTGCTGATCTGTTTGAAAAGCACAATGATGACCTTGCTGCACTCGAGACTTGGGATAATGGGAAGCCATATGAAC

AGTCTGCTCAAATCGAAATTCCAATGCTGGTTCGCCTATTTCGATACTATGCTGGTATGTCGAAATGATAAACATTAACAACAGTCTTTCAAATAGATTC

TTTAATTATTGATGAAAATATTGAAAATCACAATTATTGGGGGTGTCACTTCTTATTTAACGAGTTTCACTCAATTTTATAATTGTAGACGGTATGTTAG

AACAAGTGTGTTAGAGAGTGTGCTGTTAGCATTACTCATGTAGAAATAACTTGTCCGATTATCTCTTTGTTGTTCAATGAAACCAGTACCAACACTTCTA

ATTCCAAATTGCTCAGGTTGGGCAGATAAGATTCATGGTCTCACAGTTCCAGCTGATGGGCCATATCATGTGCAAACATTGCATGAACCCATTGGTGTTG

CCGGTCAGATCATTCCGTGGAACTTCCCTCTTGTCATGTTTGCCTGGAAGGTTGGGCCAGCATTGGCCTGTGGTAACACCATTGTTCTCAAAACAGCTGA

GCAGACTCCATTATCTGCTTTGTATGCATCAAAGTTGCTTCATGAGGTGGGTCACATTCCATACTTTTTCAAAAAAAAGATTGGTTTTAGTTTATTTTGT

TGGAAGAAATGAAAAGATTTGTATATATATGTTTAATGCTTGTACCAAATTTATCATTTCTGACAGGCTGGACTGCCTCCTGGTGTTCTGAATATAATAT

CTGGCTTTGGTCCAACTGCCGGTGCTGCTATTGCTAGTCACATGGACATAGATAAGGTATTAAGATATTTAGTCTTTCAATAGTATTCATTTTCACCCTC

TCCACCCTTTTGACAGTAAGCTATTCACTTGTTTTCTCAACATTGGCAAATCCCTTTAGTGAAAGTGTGAGGATTTTGATTGTGGTAGGAATGAGTGTCT

TGTGTATTCACAAACAAGAAGCTTATAAAAGTGTGTGTATAAACTTAAGTCTTTGCTTTGTTGCATTTTGTTGCAGCTTGCTTTCACTGGTTCAACCGAA

ACTGGAAAAATTGTCCTTGAACTGGCGGCTAGAAGTAACCTTAAGCCTGTAACTTTGGAGCTTGGTGGGAAATCCCCATTCATTGTATGCGAAGATGCTG

ATGTAGATGAGGCTGTTGAGTTAGCACACTTTGCATTATTCTTTAATCAGGTATATGAGTATATCCCAAATATCAAGTTGTTTGTAAAATTGTCTTTCAT

AGAGTTCAGAAAAAAATTAATTGGTGGTTTTTGTGACATTCAGGGACAATGCTGCTGTGCCGGGTCTAGAACATTTGTACACGAACGTGTGTACGATGAA

TTTATTGAGAAAGCAAAGGCACGCGCTTTGAAGCGTGCTGTTGGTGATCCATTCAAGGGGGGAATAGAGCAAGGTCCTCAGGTAACATACCCATACGTTT

AGTATCAAAATGCATAGGAAATATTACACTAATTTTATATTTCCTTACTTTTTTTTAAATGTCCGACTCTATTGACGAATTTTGTGTATGATCCAAAAAT

CAGATTGATTCAGAACAATTTCAGAAAATACTGAAGTATATTAGATCCGGTGTTGAAAGTGGAGCTACTCTTGAAACCGGAGGAGATAGATTTGGCAACT

CGGGCTTCTATATTCAGCCTACTGTCTTCTCAAATGTTAAGGTATAATTATAATAAGAGGAATGTTAGCAACACATTCTCTGATAGATACTCTGCTAGAA

AATTTTTGTAATTTTTGATATATTTTTGTAAATAATAGAAAGTTTGTTTAAAAGATTGTGTTATAGAGTTCTTGCTAGCATTTCTCTAATGATAATATCT

TTTAATTTCACTCTCATTGTATCAAGGATGCTTAAAATGTGAATTCTTTGTGTTACTAATTGTAAAAACAACCAGGATGACATGCTGATTGCGAAGGAAG

AGATCTTTGGCCCAGTGCAAAGCATATTAAAATTCAAGTGAGAACATTGATTCCTATTGGCAAATAATATCATTATCTTTGCACTTTTGCTTCATACACT

AATTGTCTTTTGTGTTTGATCACAGGGACCTTGATGATGTGATTCAAAGAGCAAATAACACACACTATGGCCTTGCAGCAGGGGTGTTCACAAAAAACAT

TAACACAGCTAATACTTTGACTAGAGCATTGAGAGCTGGAACAGTGTGGGTGAACTGCTTTGATACATTTGATGCAGCAATTCCCTTTGGAGGGTACAAA

ATGAGTGGTCAAGGAAGAGAAAAAGGAGAATACAGTCTCAAGAATTACTTGCAAGTGAAGGCTGTTGTTACATCCTTGAAGAACCCAGCTTGGCTTTGAA

CATCATTAGCTTTAGATTTATTTGATGAAAAGATTAATAAATAGGCTCCAATAATAAAGATCATTAAATTGGGTTTATTCCATTCATAGTTTCTGATAAT

GATGAAAATAATCTAGTTTCTTTTTCTGTTTCCCCCCCTGCATTTTATGGGTAGAGCGAAATATACTATGGAGTCTATGGTATAAAATTCAACGTAGAGA

TGAATTTCTGGTTGGGACTGGAATCAAATTTGTATAATGATGGATGTAAATTTCATATTACCTGTTCTCAAAGGACATTGTTCGTTTTTAGTAAATGGCA

ATGTTATGATTTGAACTGTGATTCAACTAGTAACGACTTAATATATATTTTTAATATCATATAATCGAAC

>Glyma.06G183900 | Chr06:15873310..15878929 reverse

ATCATCCACAAAATGAAACGTCAAATGTTGGGCTCACTGACAGTCCATTTATTTGTTTCGTAAAATTTTGGCAAAAAGGACAGTTGACGGTTACAGACAG

AACTTGAAGCATTGTTATTGTGTGAAGCTCCTAATCACAAAGTAGAAGAAATAATAAACACCCTTTGGAGATATTTGCTTAGGCCATTGGTCATGGCAAC

TCGAAGACTTTCATTGCTCCTCTCTCGCTCTCTATCTTCCACTTCTTTTCAAGCTGCTTCTCTACTTCACTCACTAGGTAACCAACCTATTCAGTTCTTT

TTTTTATCCACAAATGTTAGTTTGTTAATTTTTGTTAAAAATGTTAGTGGTAGGAATTCGAACTCATGACCTATTCTCTTCCTCATATTCTTCAAGCTTC

TTCTTCTTGCTTTTTGGTCACTATTAGTTTATGGGATTTGGCATATTTAAAGTGGTGGATATAACCAAGTGTTTTTCAGTTTGCATTTTCTGCTTCTCTG

CCTTCATCAAAGTACAGCACAGGAACTGTGTGACTCTAGTACTGTTAGGCTTAGCATATGCACATATAGTTAGCTTCCATTTTTAGACCCAACTAAAGTT

TTTTTTTTGGTATGTTTGAATAAGAGGAAAGAAAACACATATAATGGTATTTTTAGTTGTTTGGTTGGAGAGGAAGTGAAAAGAAGAAAGAAAAATTGAC

TTTTAAAAACAAAAATTCAATTTCTTTTCTCTACTTCTATTCCATCATTTCAACTTTCTATCTTAATTTTTGTCTGGCATACCAATGAAACCAGTTTTGT

TGGTTAGAATATGGATAGAAAAAGAGGGAAAAGAAAGAAAAAAAGTTCAATATTTGAATTAGCTAGAGAATGAGAAGGACAAAATTTCTGAGTTGCTGTT

CTTAAAAATAAATTTTCTCTCTATTTTCTTTTCACTTTTTTCCAACTAAACAAAAAAAAATATTTGGTATCTTTTTCTTCTATATTTTATTTCTCTTTCT

TTCCAAACATTCCAAGTGGTTATTAACTTACCATCCCCTGCTTTTAATTTTTTTTCCTTTTTTCTTTTATAATTTATCTTAGCTTTAGTATGTTAACTTT

TTTTTTTGTTCCCTAACCAACATAGGGAGTGCTTTGAAGTTACTATCACTTATCAGAATGCTATGTTCAATTCATTGCATAAAACTTCCACATGATTTTT

GTTCCCACAGGGAGAAACTCTGGCAAGTGGGGAAACTTTAACAGATTCAGCACTGCAGCAGCAGTTGAGGATTTAATCACTCCACAAGTTCCAATAACAT

ACACTAAGCATCTGATAAATGGACAATTTGTGGATGCTGCTTCAGGTTTGTCTAGTTTGAATTTAACAAGGTTTTTTTACTTTGCCACTTTACAAAATTC

TACAACGGTTTCATTTGCCATTTCTTATTGTCTCAGGGAAAACTTTTCCAACATATGACCCACGCACAGGAGAAGTGATTGCTCAAGTTGCTGAAGGTGA

TGCAGAAGATATCAACCGTGCAGTATCGGCAGCTCGCAAGGCCTTTGATGAAGGACCTTGGCCAAAATTGACTGCTTATGTAGGCATTTAAATTTTCCAA

TCTTCTCCCTTTGCTTGAAGAGAAAAGAATTAGTGTAGAGTAAATAATTTATGTACTGACAATGTAAAAGGTTATTACATTATCATTAGATCACAAATTT

ACATGTAGGATAAAGTGATTGAGAGTAAATAATCTATATGCACTGACAATATAAAAGGTTATTGCACTCACAGATTGTCACTTACGATAAAATAGTTGAC

TTTTATTATAGTTACCTTAAAAGTCAAACTTACTATGATTTTTAATTGATTGATATTTTATACTGATAGTACATAAATATTAAACTGTTAAATATGACAA

TGATCTGTTAACAACTTCTTCAGGAAAGATGCAAGATAATACTGCGCTTTGCTGATTTGGTTGAGAAACACGGTGATGAACTTGCAGCTCTGGAGACATG

GAACAATGGAAAGCCTTATGAACAGTCTGCCACTGCTGAATTACCAACGTTTGTGCGTTTGTTTCGTTACTATGCTGGTAGGTTCAGTACTAACAGCAAC

CAAAAGTTAAGTGTTTGTTTTATTTTTTCAAGACAACTTGCTGATATGTTGCAAGCAATTTTCTTTTGCAGGTTGGGCAGATAAAATCCATGGTCTGACA

GTGCCTGCTGATGGAAATTATCATGTGGAGACATTGCATGAACCAATTGGTGTTGCAGGACAAATCATACCTTGGAACTTTCCTCTCCTCATGTTTGCTT

GGAAAGTTGGACCAGCTCTTGCATGTGGAAATACTGTCATCCTTAAGACTGCTGAACAAACACCTTTAACAGCTCTCTATGTAGCAAAACTTTTTCATGA

GGTAAGTTCATAAGCTTTGCTCTCTCCCCTCCTTTTTAATAGACAATTAGACACAACTAGACTGCTAAATGCTAATCATCAAGACATCAGCCATGTAGTT

GCTACTAGCTAGTTTAGTCCATTGTTGCATCTATATCTCCCAAAAACATTGCTCTGGTGATACTTCATTTTATTTCTACAATTTTACCTACTTATGGAAA

AATGAATAAATAAATGAAAAATTAAACGGAATTCTACTTCTTTAGTACATCTTATTGACTTAATTTGGTCAAACTATCACGAGTAATTGATAAATAATGT

GTTCAAGTGTTCTTCCTCTTTAGATTCTGAAGGAATATTTCAGTTACATTTGTGTTTTCTACCTAGTTTAAACTTAATGTGAACCCTTTTGGGCAGGCTG

GTCTTCCACCAGGTGTTTTGAATGTAGTTTCTGGCTATGGTCCAACTGCTGGTGCAGCTCTAGCAAGTCATATGGATGTTGACAAGGTATTTTGGAGAAC

CAAGCTTCTTACACTTCTCTTGCACAGTATGAGACATTGTAGAGCAACAAGAATGAACCTGGTTTGATATCTCTAGAATTTCAGTTGCTAAAAATATTTT

GGAGAACCAATCTTCTTACACTTCTCTTCCATATGCATAATATTTGCCTGTATGATTGGTCAGTAAACCAACATAAGGTACTGTCCTGTTTTGACAGGGA

CGAGTAGTACTTATCTGTGCACTGGTGTATATAATGTATGGTCAATTTGGTGTACCCTTAAATTAGGAAAAAAAATTCGAGATAAATTTTATATGAATAA

AATTGTTTGAAAAATCCTTCACCCCAATTTAAGGGAAAATGAAAAAGACTGTCTTCACCTTGAAATTGATCAATGCGAAAGAAGGTAAGACACACAATCA

TATTCTACCTTTTTCTTTTCACTTTTAAGATTCATAATTTGATTCATCTTCCTAAAATTCTAACTATTTAGTTCCTCTTAATAGCTTAGATATTAAGGCA

TCTTATGCAAGATAGTGCCGGCATGCCTTTGCTTATCATCAAATTACCTTTTCCTTTACATAATGATATTCAGATAAAATTTAACCAATTATGTCAAAAT

ATCATATCAATATTTAACATCTTTTCTTCTTTCTTTGCAGCTAGCATTCACTGGATCTACCGAGACTGGAAAAGTTGTGCTTGGATTGGCTGCGCAAAGC

AATTTGAAGCCTGTGACATTGGAACTTGGAGGGAAATCACCTTTCATAGTGTGTGAGGATGCTGATGTTGACCAGGCTGTAGAACTTGCGCACTTTGCTT

TGTTCTTTAATCAGGTTTGTTCAACCAACTCTCATCAACTTTGAAGTTGAATCTTGATATGCATAGCATATGTGATGAACATGCTTATGATTTATGTTGG

ATTTTTTACAGGGGCAATGTTGCTGTGCTGGGTCTCGTACCTTTGTACATGAGCATATCTATGATGAGTTCTTGGAGAAAGCAAAGGCTCGTGCTTTGAA

ACGTGTTGTTGGGGATCCCTTCAAAAAGGGTGTCGAACAAGGACCTCAGGTGCTTTTTCTCTTCAAAGTAGACAACACACAAAATATTAGCAACATACTC

TCTAATATTCTTTCAAAAACACTCTTGTTGTGTGAAATTTATAGTTACTCTACATATGTAAGTTTCATTCCCCATTGATGGGTATGATTCCCAAACTAGT

GAAATTCACATGACTTTTAACCAATTAGAGATAATGTGCCAAAAGAAAACTAATTAAAGGAGTGTTTTGCTAGTACTCTTAGGCACCATATTATTGGTTG

TGTTCTTTCTTATTTTGTTTGTTCCTTTTACAGTTACTAGTGAGTAGTATTTGAGTTCAATGTATTTGAAAATGAGAAATTTACGTTTGATAGAACACCT

ACTTTGTTGTTCATTAATCAATACATTCTTGATCTTACTCTATATAACAAACTAACATTGTATTTCTGCCTTTGGGAAAAATGCCAGATTGATGTGGAAC

AATTCCAGAAGGTCCTTAGGTACATAAAGTCCGGTATTGAAAGCAAGGCCACCCTTGAATGTGGGGGTGACCAAATTGGCTCAAAGGGCTTCTTTGTCCA

GCCCACTGTCTTCTCAAATGTTCAGGTATTGACAAATTAGCACAAGTGATTCGCAGATATTTCAATAATTTGAAACATTTACTAACCCGTGCTAATAATC

AGGATGACATGTTGATAGCAAAAGATGAAATATTTGGCCCTGTACAAACCATCTTGAAGTTCAAGTGAGTGAAACTTCAATTCTCAATGGCTTGTTTTCC

ATGTTAATGCACTCTATAACACTTATCATTGGGTGATATTCATGAAGATTTGATTAAGTATATGTAGCCTGTAGGTAACCTTCCTCATTTGGTGGGTCCC

TTTTTCAAACTAATGAGACTCGCATGAATTTTAAACAATAAAAAAAAAAACAATGTGTTGGAAAAAGAAAATATTAGCGAATGTGTTAATGACACTTTGT

TTTACTATGTTGCAACATTTGATTAAAACTTCCCTGAATCACTTGTTTAATGTAGACCAAGGCAAAATGTTACTAGACATTATTGAGGAACAACAATGAA

TATTTCATTTGAAAATTATTCTAATTTTGTTCAAATATTTTTTGCGTTATACTAGGGACATCGACGAAGTAATTCGAAGGTCAAACGCAACGCACTATGG

TCTAGCTGCAGGAGTTTTCACTAAGAATGTGCACACAGCCAACACTTTGATGCGAGCACTGAGGGTTGGAACAGTATGGATCAATTGCTTTGATGTTTTT

GATGCTGCAATTCCCTTTGGAGGGTACAAAATGAGCGGCATTGGCAGGGAGAAGGGTATCTACAGTCTCAACAATTACCTGCAAGTGAAAGCTGTGGTTT

CACCAGTGAAGAAACCTGCATGGTTGTGATTTGTGAATTGCTCAAACTGCTATGACCGAAATCCTACGTGGCTATCATCTTTTTCCGCTGAGGCAATGGC

ATGCAATAAATAATGTGATGTTACTAAGATAAAACCTGATTAGGTACCTGAACATTATACCAGTTGAATTTATGTATTTTTCACTTGTTAATTGCTCAAA

CACAAACTTGGATACTGTAATGACACTGTTGCTGTAGGATATAAATTTTGCACCATTCACACTTGTGTGGATTACTGTCAAGTGAAAAAGTAATTAACAT

AAATTAATTAGCATAAAAAA

>Glyma.08G288000 | Chr08:39917358..39921545 reverse

CATGCATAGATTAGCGGCATATAAACAATTCTTAAAAGTAAATAAATCAGCCTGATCGCGTGGTGTGACAAACAGTAACACATTGGGTTGATCATGCGTT

TTCTTATATAATGGGGAACCAATAGCTATTTAATAGAACTTAATTAGTGAAGAGAGAGAGAAGACGAACACTATCATGGCTTCTTCACTCAGAATCTCAA

GACTTATCTCTCGCTCCTTTTCTTCTACTTCTTTCTTTTCACGAGGTAGAGCATGTGTTCTTTGTGATGGTGGTCTCATCATTTTTCTTAGTTGAAAAAC

AATAATTATTAAGTGATATGTTCTCCTTTTAGTTAATTTCATTTTTTTTTTCTTGAACTGATTTTTTTTTTCTGGGTTTGATTTCATTGATGATTGTTTG

AAAGAGAAAATTGTAGAAATTGAAAAAAAAAATGAAACATTTTTTGTGTACGGATCAGTTCATTCAATGTTTGGTAGCTGTCAACTACTACATGTCTGGT

TGTCCTATGTTTTCTTAACTGTACTTCTAGATTCTCTCGTTTTTTACCTAGTATTTTGTGTTTCTTGTTGAGTGTGAGTGTGTGACCATGTGAATTAAAA

CTCATGTTTGAAATTGATGTTTAATTGGATGTTTTGATTTGGATTTTTCATTGCCCTTTTGATGCCATAAGTAACTCTGTGCATGCATCAAAATTGACAA

AAAAAAACTTTCAGGTGGGAATGGTTTCCTTGGTTCAAGACAAAGTAAATTCAGCACTTCTGCTGCCATTGAGGAAGAACCCATTAAACCATCAATACAA

GTAGAACACACCCAACTCTTAATTGATGGAAAATTTGTAGATGCTGCTTCTGGTGAGTGTTTATTTTGTTAATCTTAAGCCTTTCGATCCTTCCTCTCCA

GGATTTTGGTCATAAAGTTGAGTTTTTGACTTAATAATAAGATGAAAAAGCAAAAGAAGAGAGAGAGATTTTGATGTGTTTTTTTGGGGTATCTTACTAT

TGGGATTCTTGTGGATCAGGTAAAACTTTTCAAACTTTAGATCCTAGGACAGGTGAAGTGATTGCTCATGTTGCTGAGGGTCACTCTGAAGATGTTGATC

GAGCAGTTTCAGCTGCACGAAAAGCATTTGATCATGGACCATGGCCTAAGATGACAGCTTATGTATTTGATCATAACCTCTCTATAATTTTGTTTTTCCT

GCAGTTCACAAAGATGTGATCTTTGGTCTCATGATTGAGTTGAAATCCCTTTTTGTTGCTTCCTTGTTACTGTAATTTTATAGGAACGGCAAAGGATCTT

GTTGCGTGTGGCCGATCTGATCGAGAAGCATAATGATGAGCTTGCAGCACTTGAGACTTGGGATAATGGAAAGCCTTATGAACAAGCTGCTAAGATTGAA

GTTCCAATGCTTGTTCGCCTGATTCGATACTATGCTGGTGTGTGGGATGCAAAATGTGGAACTTAGAATGCTCTTTGTTTTGTGAAAATTTGGTGACACT

TGTGTTTTCATTCTCCCCAGGTTGGGCAGATAAGATTCATGGTCTAACAGTTCCAGCTGATGGACCTTATCATGTGCAAACATTGCACGAACCGATTGGT

GTTGCCGGTCAGATCATTCCATGGAACTTCCCTCTTCTCATGTTTGCCTGGAAGGTTGGACCGGCATTGGCCTGTGGCAACACCATTGTTCTCAAAACAG

CTGAGCAGACACCATTATCTGCTTTATATGCAGCAAAACTATTTCATGAGGTGTGTGATAGTAATATAGACCAATCTTCATTTTGTTAGAAGCATGATTT

TTGTGGAGTTTAATTTACTCATGGATATCTACTATCCTCATCATTCCCAATTTGTAATGTGTCTGGCAGGCTGGACTTCCTGCTGGTGTTTTGAATGTAG

TTTCCGGCTTTGGTCCAACTGCTGGTGCTGCTCTTGCTAGTCACATGGAGGTTGATAAGGTACTAAATAACTATAATGTTCAGCCTCAGCAGTAGTGGAA

CAATGTAATGGTTTTAGTGTTGCCTTGCATGAATTTCTGTAAATTTTATGGCCATTGATTTCAGTTTCCTTATCTTGTGGAGAAGTTTCAATTACTTATA

TGATGTTTATTTTCATTCTAATTAATTATTAGACATGTGACATGATAGTAGTGTCTTAACAGCATGAATTTTTTACATTATCCCGCAGCTTGCTTTTACC

GGTTCCACTGATACTGGGAAAGTTGTCCTTGAACTGGCGGCTAAAAGCAATCTTAAGCCTGTTACTTTGGAGCTTGGTGGGAAATCTCCCTTTATAGTGT

GTGAAGACGCTGATGTAGATCAGGCTGTTGAGCTCGCACACTTTGCCTTATTCTTTAATCAGGTATTTTAAAGTATAGAGCTCTGGTAAAACAATTCTGT

CATAGAATTCTGAAAACTTGATTGGTGTTGCTTGTGACATTCAGGGACAATGTTGCTGTGCTGGGTCTCGAACATTTGTACATGAAAATGTATACGAGGA

GTTTGTTCAGAAAGCAAAGGCTCGTGCTTTGAGACGTGTTGTTGGTGATCCATTCAAGGGGGGAATAGAGCAAGGTCCTCAGGTAAATTGCAATGAAAAT

CCTATACACTGCATCCTCATGTTTGATCTTTGAACCCTGTTTTCCAAAAGTTGTCTGAGGGTATAATACGTCCAACAATATGGCATTGTGTCTGTGCTTC

ACTAACTCAATGCTGATTGCTGATGACCGGAGATATGTTACCATCCTTATGATCAAAGATGGATGGGCAGTGGCCCGAGTGCCCCTCCCTTGGTACTTCT

CATGTATATAGAAATATATTTTTAAGTTAGTAGATGTAAAATTAATTATTTATGTTATTATTATAATAATCATTAAGGTTAGTTAGTGTAAAATTAGTTG

TGTTTGTTATTATTACTGTAATGGTTAAGGTGATAACTAGAATAAAATTATGTAATACTAATTGTGTGTTTGTTGTTTTTATGATAACGGTTAAGGTTAT

TATCATTTAAAATAAAAATTAGTTTCAATTTTTTGTAAAAATAATAAATATTTTTTTAAAAAAAATTATTACTTATTAAAAGTTAGACATTTTAAACAAT

TACAAAGGAAGAACCCCCTTTAATAGGTTTCTGGATCCGTCCTTGCTTATGATTTTCAGATACTAGAGGCTTCACTGTACTAAAATGCCCAGAAAGAATG

TTACATTGCTACTACTTTGACATTCCCTTTTCTTAATGTATGACTCTTTTTAAAATGTCTAAATACACTGATTATTTCTTGTATGATCCAAACAATCAGA

TTGATTCAGACCAATTTGAGAAAATACTGAGGTACATCAGATCCGGTGTTGAAAGCGGGGCAACCCTTGAAACAGGAGGAGACAAGCTTGGCAACAAGGG

CTTCTACATTCAACCTACAGTCTTCTCAAATGTTAAGGTACATCTTTCTAGTTGCATTTTCACCTTATCAGAAGAGACTCTGAAAAGTTAAAATACAACT

CATAATGCTTTCAATTGAATTTGTAACAATCACCAGGATGGCATGCTGATTGCAAAGGATGAGATCTTTGGTCCAGTCCAATCCATATTAAAATTCAAGT

AAGAAAACAACTAGTGTTAGTTAATTACTTTGCAGAGAATGGTATTATACTACCATACATGTGTTGTGCTTTGTGCATTAATTTTTGTGTTGATGACTCC

AGGGACCTTGGTGAGGTAGTTCAAAGAGCGAACAACACACGTTACGGGCTTGCGGCAGGAGTGTTCACAAAGAACATGGACACTGCAAACACTTTGACGC

GGGCACTGAGAGTTGGAACAGTTTGGATAAACTGCTTTGACACATTTGATGCTGCAATTCCCTTTGGAGGGTACAAAATGAGTGGTCAAGGCAGAGAAAA

AGGAGAGTATAGTCTCAAAAACTACTTGCAAGTGAAGGCTGTCGTTAATCCCTTGAAGAACCCAGCATGGCTTTGAGAAACACTAGTGCCTTCCTTGGAT

GATCAACTATCAGTGCCTCAATGATAATGATGATAAAAACAAAATCCTTTTTTTTTTTTGTTACACGAGTTGAGTTATGACATTGTAAACATAATAGTAA

AGAATCTTGTCAATGAAGTAAGTGTCTATGCTGTGCGTGCTACTATATATATATATCATAAATTCATAATTAAATAAAGACTCTTAAG

>Glyma.13G170600 | Chr13:28436396..28440865 reverse

TTAGAATGTGACTCTTTTTAACTTGTTCATTGGCTCACTCTTAGAATAGTAATTTTGATCCATAAGCAAATTAGACATGATATGCTCATTTACGTTAATG

ATAACTGACTATTGCTAACTATGTTTTGCTGTCTACCTTCTCTTCCATTCGGGTGACACAGTACTGAGATGCTTACTGGGTAACACTTTATTAAGCTCTT

AAAAGAAGGGTGGTAAGAAAATTAATTTCACGAAGATTTGTATAGTTCTAGAAATCTCTGAGGGTAGGATTACAAAGAAAGGGAAGAAAAAAAAATCCTC

AACATTAAAGAATCTTCATGCAGGTCATGGCCTCTAGAATTCTTTCAACTCTACATTATGTGTGTTCTTCTTCTGCCTCAGCCACAAAACGATGTTTAGG

TAATAGCAGTATCTCTCCCTTCTATAACGTGCATTTTGAGTAGAAGATTGAAATCTTCAATATCTTCCTGTTGTGTTATATGCTGACATATGGAAAAGGG

AAAGAATATATATATTCTTTATAAATTGCATTGGAGAAAACAATTTAAAACTTGAAGGGAGGTTTTGAACTTTTTTTTTTTTGCCTTTTGGTTATATACT

AACCACACATGCACGATGGTGCTGCTAATATGCTGTCTGTCACATACATACACATCTTAATTTCTTTAACAATAAAGTATAGAGAAGTGTAACATATATT

TCTGATCATATCTTAACATTATTAAATTCGTTAGTCTGAAAATTTGGATAACTATTGACTAAATTTTCAGGCCTATATAGTCATTGGCAGAGAAGTATAA

GTGGAATTGCTGCGTCTGTTGTTGCTGATGTGGAGCCTAGCATAGCACCAGTGCAAATTGATCAAAGTCAGCTTCTAATTGATGGGAAATTTGTTGATGC

AGCTTCTGGTTAATATCTTACCTTCATGTACTTAAAACGTTGTTTCATTCATATGATTACTCATAACAATCTATGCAACAGAAACATAATTTAACTATTT

TGTATTTGTTAGGAAAAACTTTTCCAACTTTTGATCCTAGGACTGGAGATGTAATTGCCAATGTTGCTGAAGGAGATGCTGAAGATGTAAATCGCGCTGT

CCATGCTGCTCGCAAGGCTTTTGATGAGGGACCATGGCCAAAGATGACAGCTTATGTATTAAAACATTATCTAATACTTATTTGTTCTATAAATTTCAAT

ATGGAGCTGTTTTGATGCTGCACTTTTTTCTTTTGGACTACAAGATGCTACATGTTTTTTATATACAAGTTTAATCATTATCTTTGATGAACTTCTCTAG

GAAAGATCACGTATAATTTTGCGCTTTGCTGATTTATTGGAGAAACACAATGATGAAGTTGCAGCCATAGAAACATGGGATAGTGGCAAGACTTATGAAC

AGGCTGCAAATGTTGAAATACCCATGGTAGTACGATTGTTCCGATATTATGCAGGTAATTCCATTATCTTTGAGTTCTTATATCACGTAGTTATAATATT

GTATAACAAGTTGATGTTCTTTGGACTTCATTCAATTTTCTTCAGGTTGGGCGGATAAAATTCACGGTCTAACAGTTCCAGCTGATGGACCATACCATGT

CCAAACTTTGCATGAACCAATTGGTGTAGCAGGGCAGATTGTTCCTTGGAATTTCCCACTTCTTATTTTTTCATGGAAGGTTGCACCTGCATTAGCTTGT

GGTAATACAGTTGTAATGAAAACTGCAGAGCAGACACCCCTTTCAGCCCTTTATGTGTCAAAGCTATTTCTTGAGGTAGCCTTATACAGTTTTACCACAC

CTAAAAATGTTTCCATTTATGTGTATACTGGCTTGGATTTCACTTTTGTGACCATCTTCTTATTCTTTTATAGAGCTGCAATCCCTCTTAAATTCCATAC

CTTCCCTATCGTATATGATGCCTTGACTTCGAAATCTCTACCCTTCAAAATGAACCGAATCAATAAGATTCTTGACCAAGATGAAGGCTACAGAAACTTT

TTTTTTTCTTCAAGCTTATTCGTTAATTGTTACTAATACTAGATCAATAACCTAAAATTAACAAAAAAATAAAATTACCTCTTCCATTAAAGAGGAAAAT

GTGTACAAGTCTTAAATGTTACAATTGTCAGTTTGTCTCCATTATTAACTGGACCTCTAGAAATTAAAAGCTTCAATTTGAGATGAACTTAAGCCTGCTA

TCAAAATTGCTTACAACAAATAGATTGTGGTCCTGCATGTGTCTATAACTTTGTAATTGCCCTCTCTGATGGATTTGATGCAATAGGCAGGACTTCCTCC

TGGAGTTCTGAATGTTATCTCTGGCTTTGGTCCTACTGCTGGTGCAGCACTGTGTAGTCATATGGACGTTGACAAGGTACTTCAAAAGATTTAGAGGAAG

AATTCTCTTGAGACTTAAAAAAAAAGGGTAAAATGTGTTTAATTGGTCAAAATTGGTTTTAGTCTCTATATTTTAAAAAGAATGATTTCGGCCCTTGTAT

TTATGAAATTTTGTGAATTTTGTCACTGCTCAAGTCAAAATAAAGTATAGGGTTAGGGACAAAATTTACCACATTTTAGAAATGAGGACCAAAAACATTG

TTTTTAAAATGTAGGGATTAAAACCAATTTTGACCAAACTAAAATACATTTTATTCTAAAATATATATCTTCATTTTTCTTATTCTCTCTAAGTCTACAA

CACAAATATGCTTTAATTTATGCTGCCTAATGGCCTAATAGCTACTAGTAAACTAAAAATTGGCTGATTTATTTTTTGTAGCTTGCTTTCACAGGATCCA

CTAGCACTGGTAAACGTGTACTTGAACTGTCTGCACACAGCAACCTGAAGCCAGTAACTCTGGAGCTGGGTGGAAAATCTCCATTCATTGTGTGCAAGGA

TGCTGATGTTGATGCAGCCGTTGAGGCTTCACATTTTGCTTTATTTTTTAATCAGGTTTGATGACTATAGTTTATGTTATGAGGAGTAATAACAATATGC

TCTACTTAAAATTTATTGAAAACTACAAAAACATAACAGATTCATTAAATAAAAAGTGAGATCCACAAAATTTGTGATTTCTAATAAATTTCAGCCAACA

ATAAAGTGTGTTATAAAAATAGTATGTTAAAGAATGTGTTGGTAACATTTCTCTTACGTTATTATTAATAATAATGAGAACGGTGCATTGCTTGTATACA

GGGGCAATGTTGCTGTGCTGGTTCTCGCACATTTGTCCATGAAAGCATATATGGTGAATTTGTTGAAAAGGCTAAAGCCCGTGCTCTTAAACGAGTAGTT

GGAGACCCTTTCAAAAATGGGGTTGAGCAAGGTCCTCAGGTATCTGTGACTACCTCCTATCTTCTATTTCCTAATTAACCTTAAATCAAAGAGAACCTTT

GGTTCAAATACGAATAACATTGTAAGTTTTTCAATATCTTGGTTTTAGCATGTAAAAGAAACTACTTTTGCAATTTCTCATTCATACTAACAGAGTTTTC

TTTTTTCTTTTTAACTTATGGTAGATAAATGCTAAGTAGGTGGCATCTTAAAGGGGGATGGATTGATAATTGTATGTGTTTCAGATTGATTCTGTGCAAT

TCGAGAAGATCATGAAATACATAAGATCAGGCGTTGAAAGTGGTGCTCAACTGGAATCTGGTGGTCAAAGAATTGGCTCCAAAGGCTACTACATCCAACC

CACTGTTTTCTCAAATGTTCAGGCATGATACTTCCAATTAATATGGTTCTATTTCTGAACAAGTAGTAACATAACTAAGAGACAGAAGTAAATAGCTAAA

TTGGTTTCTTTGTTATTATTACAGGACAACATGTTGATAGCAAAAGATGAGATATTTGGTCCAGTACAATCTATCTTGAAATTCAAGTATGCTACAAATC

ACTAGATAAAAGTGCCATCATGTTTGTACTTTGACATTCTTCAGTAGATCCTCCTCTTAAATCCTACTTATTCTGTTTTGAAATAAATCATAGGGATCTA

GAGGAAGTGATAAGAAGAGCCAATGCAACTTCCTATGGCCTGGCTGCTGGAGTTTTTACTAAAAATATGGACACTGCCAACACCTTGATGCGAGCATTGC

AAGCTGGAACGGTATGGATCAACTGCTATGATGTCTTTGATGCAGCAATACCTTTTGGTGGATATAAGATGAGTGGTCAAGGGAGAGTAAGGGGAATCTA

CAGCCTTAGAAGCTACCTTCAAGTTAAAGCTGTTGTCACTGCTTTGAAGAATCCCGCATGGCTGTAGTTTTCCCACTACCTGAGTGTTCATGTTCATAAT

GTCTTATCTAGAAAAAAAACTACATCTTTCATGGAACATTCCTAAGGCATTGAATTAGCCAAAGTTATTACACAACTAAAGCTTTTCTGTGCCAATCTTT

GCTAACTCAACTTACCTTCTGTTAGAATATCACCCTTGGTGTCAAAAAAACTTCTCTTTACTAAGTTAAA

>Glyma.17G091000 | Chr17:7090659..7095786 reverse

ATCAGCACTTTAATATCCTTTTCAGACCCCACAAGTATGGACAAAATGTCATCCATAAAATGACACTTTCTTGCATTGCTTTCTTTTTTACTACATAATA

TTGATTGATGGTGATAACGTTTAATGACACAAGGTACAACCATTTTTTATTTTGAAAAGACTCTTTTATCATTCATAAGAATAAGAACAAGGAGGAGAAG

AAAAAGAAGCAGGGAAAAATATTTGACTTTGATGATGGCAGCTCGCAGTCTTTGCAGGCTACTCTCTCGCTCTCTCTCTTCTTCCAATTCTTCTGGAGCT

GCTTCTCTGCTTCACTCACTAGGTGATCAACTTAGTTTCATTCTCATCTTTTATTTGTTAATGCTTAATTTGTTTCCTAACCCTTTATGAATGTTGATGA

TGGCCATGAGTTTCCCAGATTGCAAAGCATATGAATAATTTGACTAGCACTATTCACTTTTGTTTAGCACACCAAAACTATTGTGATATAATGAATCTTT

TTCATTACTTTCAAGACCAAATTTTAAAGTATCTTCTGATAGCGGTTATTATCCACCACTTGTATTTCTTTATATATTCATTTTGGCAACTATATTATCA

GAATACAATCTTCAATTTGTTGAATAAAACTGCCACATGATTTCTTTGTTTATCCCAGGAAGAAACCCTGGAGGGTGGAGAAACATTAACAGATTTAGCA

CGGCTGCAGCAGTTGAGGAACTAATCACTCCACAAGTTTCAATACGTTACACACAGAACCTGATAAATGGGCAATTTGTGGATGCTGCATCAGGTGTGTC

TAGCTTGAACAAGCTTTGTTATAACATGTTTGACTTCTAGTTCGAACACCCCAAACCTCACTTTGTTATAACATGTTTGGATTAAAATTGGGAAAGTATT

TTTGTGAATGCTAATGCACATTTGCAAATTTCAATACACAAAATGAAGAGGACTAATTTGTTGCTTTGGGAGTAAAAAATACTTTTGAACTCTCTGAACT

GTAATGCAAACATGCACTAACTTGTTTAACAAAATCATGGTTAGGTTGTGTACTTGTGTTTGAATTTGAACATGAATGTTGCGTCTCAATTCATGGATTG

AGTGGAACTGAAAAGAAGTTAGCTTTTCCCATTTGTAAAATCAATTGGTGGACTTTATCTACAATTTCTTTTGGTCTCAGGGAAAACATTTCCAACATAT

GATCCGCGCTCAGGAGAAGTGATTGCTAATGTTGCTGAAGGGGATGTTGAAGATATCAACCCGTGCAGTATCAGCAGCTCGCAAGGCCTTTGATGAGGGA

CCTTGGCCTAAAATGACTGCATATGTAGGCATTTCCTGAGTGTCCTCCCTTTCTTGCACATCCTTCAATTAATATTTGGTAATAATTTATCAATAACTCC

TTCAGGAAAGAAGTCGGATATTGCTGCGCTTTGCTGATTTGGTTGAGAAACACAGCGATGAGCTTGCTGCTCTCGAGACATGGAATAACGGAAAGACTTA

TGAACAGGCTGCCAAGACTGAATTACCAATGTTCGTGCGTTTATTTCACTATTATGCTGGTAAGATCAGTAACAACAGTTAGTTGTTAATTATGTTAATC

CTTCATTTTTTTAAAAGAAATTTTATCTTGACACCCTCATCACAACAATTATTGTTTTACAGGTTGGGCAGACAAAATTCATGGGTTGACAGTGCCAGCT

GATGGAGATTATCATGTGCAGACATTGCATGAACCAATTGGTGTAGCAGGACAAATTATACCTTGGAACTTTCCTCTAGTTATGTTTGCTTGGAAAGTTG

GACCAGCTCTAGCATGTGGTAATACCATTGTTCTGAAGACTGCTGAGCAAACTCCACTGACAGCTCTCTTTGTAGCAAAACTATTTCATGAGGTCAGCTC

ATATCTCATAATCTATGCTTTCTCTCTCTCTCTCTCCCTATCTCTTAAGCATGGATACAGTCAAATTTAGTCATCAAAACATCACCTGAACACTTGTTAG

TTGAGCCTGTCATTGCTTATTCCCCTGAAACAGTGCTCTAGTGCTACTTCATTCTACTTCTACATACCTAAAAAATGGAAAAATAATGATGCAAACATCA

CGTTTTCTAGTGGTGGAAACAGCCTCTTCACCTACATGGGCAGAATGGAGGGAGCCTCATGCACCGGTTTACCCTTATTAAATTAAAAAAATTGTACCTA

ATTTGATACATCTTATGAACTTAAAAATCTACCTTTAAGCCACAGTTTGCCTTGTCAAAGTCTACTGCATATATGAAGTTTGCACAATGTTCATCGATGT

CACCAAAAAGTGTACATGGGATATTTGTTCTATAATTTGTCATGTGGTGTCATTATCTCAGTAGTTTTGGAATAATGTTGCCGAAGTTATAAAGAAATAC

AGACTGTTTTGATATGTGATGTCAATTGTCAAATAATAACTCTAGCCTTTTGTTTTTTACTTCTTTAGTTAACCCCAATTTGTGCTGACATGCAGATTTT

CTTATTTAGATTTCGATGACTATTTCAATTGCATTTGTGTTTCCCTAGATAATTTGTAACCTTCACCCTTTTGTGCAGGCTGGTCTTCCTGATGGTGTTC

TGAATGTAGTTTCTGGGTACGGTCCAACTGCTGGCGCAGCTCTTGCAAGTCATATGGATGTGGACAAGGTATTTTGGGAACCAAGAATGTTAGACTCTTA

TATATAGTTTTGTATGAGAGTCAAGAACGAATCTGGCTTGATGTTAGACATTATTTTGTTCTAACTAATCTTGAGGGTGTAGATTGCTAAACATGTTAAG

GATACTGTCGTATCATAGTGCATTATATTTGCCAGCATGATTGGTCCAGATCCCATGTAAGTCCATGTCAGGTTTTATCAGATGACAATAGTTCTTATTT

ATACCCTATTGTATGAGACTTAGCCTTTAGAGTATAAAAATTGGTTAGTGGAAAAGTAATGAAAAATACATTATCTTGAAGGTGGTTAGCCTGTTTCTGT

ATCAAAGAACAGGTTGGTTGGCAGTCTGTCAAGGTATTTTGGAGCCAATAACAATTTTTTCTGCAATATTTATTTGTTTATTGCCTGATACACATGACAA

ATGACTTTATTATCTTTGAGGGACATTAAATCAAAACTGCTCCTACATATTAGGGAAAATTGGTTCTTTGATACTGTCTAGTGTTAATGTAAGAACAATG

GCATTCATTTGTTTATTGTCTGTATCCTTAAGCTTTGAATTTTCCCTTTGCATAGTAATTGTCGGGTAAAACCAAACCATTTTTTATCAACATATAAAGT

TCTTTTTTTCTATTCTATTTTTGCAGCTAGCATTTACTGGCTCAACAGATACCGGAAAAGTTGTACTCGAACTGGCTGCAAGAAGCAATCTTAAGCCAGT

GACATTGGAGCTTGGAGGGAAATCGCCTTTCATAATATGTGAGGATGCTGATGTTGACAAGGCTGTTGAACTTGCACACTTTGCTCTGTTCTTTAATCAG

GTTTATTCAATCAGATTCAATCACTTCATTGAACCTCAATATGCATGTGTCAAGAAGAAGCTTATGCCTAATGTTGGATCCTTTACAGGGGCAATGTTGC

TGTGCCGGCTCACGTACCTTTGTACATGAGCGTGTCTATGATGAGTTCTTGGAGAAATCAAAGAAACGGGCTTTGAGACGTGTTGTTGGTGATCCATTCA

AGAAAGGTGTTGAACAAGGTCCTCAGGTAATTTTCTTTTCAAAGTAACCACTGTGTTATTGTACTTTACAAGTTGTTCTTTTCTTTTTGTTTCATCCTTA

ACAATCATGAGTTCATGGGAAAAGTTAAAGTTTTACAGAAGACCACATTCTTGATCATACTCTTATGTAACTAAATAACACTATTTCATGTCTCTTGCAA

AAACCCTAGATTGACGTGGAACAATTTGAGAAAGTCCTTAGGTACATAAGGTCTGGAATTGAAAGCCATGCCACCCTTGAATGTGGGGGTGATAGATTGG

GCTCAAAAGGCTTCTTTGTCCAGCCAACTGTCTTCTCAAATGTTCAGGTATTGAAATTATTTACCACAAGTAATTTGCATAAATGCAGCAAGATTCTAAA

ATATTGAAGCTTTTTTCTAACACGTGCTAATGATTAGGATGACATGTTGATAGCACAAGACGAAATCTTTGGCCCAGTTCAGTCCATCTTGAAATTCAAG

TGAGTGAAATTTGATTCCTCACTTGCTTATGCAATTCTATTACAAAATAAAAATTTTAGTCAAATAAATCCTACAAATGTGTGGTGATATATCATCCTAT

TGTCGGTGGATCCTTCCAACTCAACTTATTTCATCATCAGTAATTAATATAGCAAATGGCCAACGTAGAAAAAAGACCACCATTTTTGTAAAATCATAGT

GAATACAACAAAAAGCATTCATATTGATTTTAATGTGGCATTATGAGATTTTTCTAATAGCTTTTGGTTTATATAGGGACATTGATGAAGTAATACGACG

GGCAAATAAAACACGCTATGGTCTAGCAGCAGGTGTGTTTACTAAGAACGTGAGCACAGCCAACACATTGATGCGGGCACTGAGAGCTGGAACTGTGTGG

ATTAATTGCTTTGATGTTTTTGATGCTGCAATTCCTTTTGGTGGATACAAGATGAGTGGTATTGGCAGGGAGAAAGGAATCTACAGTCTTCACAACTACC

TGCAGGTGAAAGCTGTTGTCTCACCAGTGAAGAATCCTGCGTGGCTATAAAATGTCCACACTTGTGTCCCTTGTGTCTTCAACAATAAAAATCGATTGCA

CACAAGAGCTGAATTTTAATCATGAATTAATCACCTCGAATTTGATGTGATCTTAGCTAACAAACGTGCTTCGTATATTTATTTATCCAAAATAAAAAGT

TCCATGCATCTACTACTAGTTTACTACTTGGCAAAAATATACATAACTACACAATTACCACTACATATATGATACCTTAATGGATTAGTTGAGTGGAGCA

AGACAAGGTTTCACATGTGACAAATCAAAGCTTCGAATTTCTACTTGTCTTTTTGAGACAAGGTAAATGCAAACTAAATTCCTCTGAAAGTATTTTTTGA

GCTCGGAGGCTTGGCTTTGTGTGTCTTG

>Glyma.18G137300 | Chr18:20178242..20182076 forward

AACAGTAACACATGTTTGATCATGTGTTTTTATAAAATGGAACTGAGTTAGGGAAGAGAGAGAAGAGGAACACTGACATGGCTTCTTCACTCAGAATTTC

AAGACTCATCTCTCGCTCCTTTTCTTCTACCTCCTTCTTTTCACGAGGTAGAGATGCATGTGTACTTTGAGATGGTGGTCTCATCATTTTCCTATGTTAT

AAAACAATAATTAAGTGATTTGTTTCTCTTTTTTGTTGATTTCCATTTTTTTTTCTTGAACCGATTTTTTTCTGGGTTTGGTTTCGTCGATGATTGCTTG

AAATAAAAGGATGACCAATTGGAAAAAGGGTGGGATCCAGAAATTTTTTTGTGTACGGATTAGTTCATTCAATGCTTGGTAGCTGTCAACCACTACATGT

CTGGTTGTCCTATGTTTTCTTAGTTGTACTTATAGATTCTCTCGTTTTTCACCTATTATTTTGTGTTTCTTGTTGAGTGTGACCATGTGAAAGCTATGTC

TATGTAAATTAAACTCATGTTTGTATCACTTTGAAATTGATGTTTAATTGGGTATTTTGATTTGGACTTTTCACTGCCCTTTCAAGAATAATTAACTCTG

TACATGGATCAAAATTAAAATAAATAAAAAACTTTCAGGTGGGAATGGTTTCCTTGGTTCAAGACACTGCAAATACAGCACTTCTTCTGCCATTGAAGAA

GAACCCGTTAAACCATCAGTGCAAGTAGAACACACCCAACTCTTAATTGATGGAAAATTTGTAGATGCTGCTTCTGGTGAGTGTTTATTTATTTGTTTAT

TTTGTTAATCTTGTTTTGTTTTTGAATCCTTCGCCTTCACCCTTTTTGACATAAAGTTGAGTTTTGACTAAACAGAAAGATGGGGGAAAAAAAAGAAAGA

AAAGAGAGGGATTTTTTTAAGGTTTCTTTTTTCTGGGTATCTTACTATTGGGATTCTTGTGGGATCAGGTAAAACTTTTCCAACTTTAGATCCTAGGACA

GGTGAAGTAATTGCTCATGTTGCTGAGGGTCACTCTGAGGATGTTGATCGAGCAGTTGCAGCTGCACGAAAAGCATTTGATCATGGACCATGGCCTAAGA

TGACGGCTTATGTATTTGATCATAACCTCTCTATAATTTTACTTTTCCTGCTGTTCACAAAGATGTAAACTTTGGTCTCATGATTCTGAGTTGAAATCCC

TTTTTTGCTTTCTTGTTACTATGATTTTATAGGAACGGCAAAGGATCTTGTTGCGCGCAGCTGATCTGCTTGAGAAGCATAATGATGAGCTTGCAGCACT

TGAGACTTGGGATAATGGAAAGCCTTATGAACAAGCTGCTAAGATTGAAGTTCCAATGCTTGTTCGCCTGATTCGATACTATGCTGGTATGTGGGACACA

AAGTGTGGAACTTTGAATATTCTTCTTTGTTTTGCAAAATTTTAGTGACACCTGTGTTTTCATGTTCCCCAGGTTGGGCAGATAAGATCCATGGTCTAAC

AGTTCCAGCTGATGGACCATATCATGTGCAAACATTGCATGAACCAATTGGTGTTGCCGGTCAGATCATTCCTTGGAACTTCCCTCTTCTCATGTTTGCC

TGGAAGGTTGGACCGGCATTGGCCTGTGGCAACACCATTGTTCTCAAAACAGCTGAGCAGACACCGTTATCTGCTTTATATGCAGCAAAACTATTTCATG

AGGTGTGTGATAGTGGTATAGACCAATCTTCATTTTGTTAGAAGCATGGTTTTTGTGGAGTTTAATTTACTCATGAATATCTTCAATCCTCATCATTCCC

AATTTGTAATCTGTCTGGCAGGCTGGACTTCCTGCTGGTGTTTTAAATGTAGTATCTGGCTTTGGTCCAACTGCTGGTGCTGCTCTTGCTAGCCACATGG

AAGTTGACAAGGTACTAAAATATTTAGCATCTGCAGTAGTGGAGAAGTGTAGTGATTTTATTGTTGGCATGCAATAGTTTCTGTAAATTCTATGGCAATT

AATTTCAGTTTCCTTATCTTGTGAAGAAGTTTGAATGTTTGATATGATCATTTTCATTCTAATGGATTATTAGACATCTAGACTTGATAGTGTCTTAACA

GCATGAATTTTTTACATTATCTCGCAGCTTGCTTTTACTGGTTCCACTGATACTGGGAAAGTTGTCCTTGAACTGGCGGCTAAAAGCAATCTTAAGCCTG

TTACTTTGGAGCTTGGTGGGAAATCCCCCTTTATTGTGTGTGAAGATGCTGATGTAGATCAGGCTGTTGAGCTAGCCCATTTTGCTTTATTCTTTAATCA

GGTATATAAACTATAAAGTATAGTGCTCTTGTTGTAAAACAATTAGATTCAGAAAATTTGATTGGTGTTGTTTGTGACATTCAGGGACAATGTTGCTGTG

CTGGGTCTCGAACATTTGTACATGAAAGTGTATACGATGAGTTTGTTGAGAAAGCAAAGGCTCGTGCTTTGAAGCGTGTTGTTGGTGATCCATTCAAGGG

GGGAATAGAGCAAGGTCCTCAGGTAAATTGCAAAGAATCCTTTACATTGCATCCTCATGTTTGATCTTTGAGCTTTGTTTTCCAAAGTTGTCTGAGGATA

TAATATGTCAGTCAATTTGGCATTACGTCTGTGCTCTCATTAATTCAATGCTGATTGCTGATGACTGGAGATATGTTACAACCCTTAATTCCTTATGAAG

TTCTGGTTCTCGAGGCTTCACTATACTAAAATGCACAAAAAGAATGTTATATTGCTACTACTTTGACATTCCCATTTCTTAAAGTATGATTCTTTTAAAA

TGTCTAAATACTCTGATTATTTCTTCTATGATCCAAACAATCAGATTGATTCAGATCAATTTGAGAAAATACTGAGGTACATCAGATCTGGTGTTGAAAG

TGGGGCAACCCTTGAAACCGGAGGAGACAAGCTTGGCAACAAGGGCTTCTACATTCAACCTACAGTCTTCTCAAATGTTAAGGTATATCTTTTTAGTTTC

ATTTTTGCACTTTATCGAATGAGTCTGAAAAATGAAAATACAACTCCTAAAGAAGTGTTTTCTATTGAATTTGTAACAATCACCAGGATGGCATGCTGAT

TGCAAGGGATGAGATCTTTGGTCCAGTCCAATCCATATTAAAATTCAAGTAAGAAAACAATGAGTCTTAAATACCTTGCTGAGAATGGTATTATACTATT

ATGTATGTGTGAACTAATTTTTGTGATGATGACCCCAGGGATCTTGGTGAGGTAGTTCAAAGAGCAAATAACACACGTTACGGGCTTGCGGCCGGAGTGT

TCACAACGAACATGGACACTGCCTACACTTTGACGCGGGCACTGAGAGTTGGAACAGTGTGGATAAACTGCTTTGACACATTTGATGCTGCAATTCCCTT

TGGAGGGTACAAGATGAGTGGTCAAGGCAGAGAAAAAGGAGAGTATAGTCTCAAAAACTACTTGCAAGTGAAGGCTGTTGTTAATCCCCTGAAAAACCCA

GCATGGCTTTGAGCAAACACTAGCCTTCTTTGGATAATCAATAACTATCAGTGCCTCAATGATAATGATGATAAAAATAAAATCCTGCTTTTTTTTTTTT

TTTGGCGTTACACGAGATATGAGTTATGACGTTGTTGATATTTTCCTGGTCTTCATCCTTTTATCTCTTTTGTCAATTTCTATTCCTTGCATTTGTATAA

GTCAGAGTGACACACTTGTTACTCTTATGGTCTAAATTGAGTTTTTAATTATACGTGTTTGAATTGGATTATTTTAAAATTATATTTTTTTTTGTAAGTT

TTTTCAAACGCGATCTAATGGCTAATTGTTTGTAG

>Glyma.19G010800 | Chr19:1020788..1033952 reverse

ATGACTAGTATTCGACAATGTGAAAGTGATGAATCAAGTTTAAAATCTGCATTTGAAGAAGTATCAACATTTAGTTAGTGCCTGACACAAAAAGAGGATA

CTTGTTAAAAAACAATATAATAAATATATAATTTCAAAATAACTGCAATAAAAATTCAATACAATATTAATTATAATGTCAATATTAACCTGTTAGATCT

AATCAGCTCTTAGATATTTATAAGCTTCCACAGGACACAATGAATGAGAAGAATAAGGTGCCCTACTCTCCTTGCAGTATAAATCTGACAAACTGAGGAT

AAGGAAGCTACACTACAAAGAATGGATCAACAATCAACATCAAGAAAACAAGATATTTTACACTTGTTTTTACAATATTCATAATTAGCTACATATAAAT

TATCAGAGGGAGAGACAAATTAACTAGTGTTAATCATTAGTGTTCTTTGGATACATTTTTCATAAGTATTTATAGAAATAGAAAATAAAATAAAAAAGAT

TAAAATTTTCTCTAAATTAAAATTAATTTAATTATATGAACTTAATTTTTATTGAAGTTCTCTCAATTTCTCTAAAAGCTAAAATATATAAATTAATTTT

AATTTATGAAAGAAACTTAATTAATTCACTTTACTTTTCTATTTTCTTTTCTTACATACATAAGAACTTATTAAAAAAAAAAGTTATCTGAACAGAATGG

TTAGATTTAAATTGTAGAATACCGTTAAATATATTGATGAATGATAATATACTTCTTCAGTTCTTTGAAATGATATGTTTTTATTAATGATGAATGTAAA

ACAAGTACTATAAAAGATCTGTTTCTCTTGATCTTGATGATTCTCTCCTTATTTGTGTTGTGATGCTTCCTTAATTATCCTGTGCAAGATCTATAGTGAG

GAGAATACGCCAGGGTACCATATATTCTTCCCATACATCATCATTTATAAGGTTAAACTTAATTAAGCACATGAGATTAATTAAGGCCATTTTATGGGAT

GGACATGCAAAACTTTGTTGGGTTACAAGTGTGAGGTAAAGTCTCACATCGGATAGAAGTGAAAAGATTGAACACCATATAAGTGAGGAGTATACCCACA

AAAGGTTTTGAGTTAGAGTGTAATGTCAGACGACTCCCTTATAAGTGACTCGTGGTTTATAGGTGTACATCTCGAATCTCCCCAACAAAACCTGTCATAG

CCCATTGTCTAGCACTTCAACGTGGTTAACAATTAGTGAAAGTTTCAGTTCCAACACATGGAAATTTCTCCTTGCACGATCTGACAACCTGATGAGTGGT

GTGGTGCCTATTAAATTCAAACCTGCAATTGAGGTTATCTATGCACTCTCTAAAACTGAAGGAAAGCAAGGCAAATGTTTTTTATTTATTTTTTTATGTA

TGGTGTCCTTCTATTATTCAACCGTGTAAATTTAAAATATCAATGAAATTAAAATTTTAAAAAAAAACTCTTGTTTTTTAGGGAAACACGAGAAAAAAAT

AAATTAAAACGATTCAGTAAGTTAGTAACAAAAGTTAAAAAATAAAAAAACTATACTGTACATCAATTCAACACCCTTCCTGAGGATTTGTAAAATGTTG

CTTAATTAATTGCTTTGGATTGTATTGAATAAAACTTGTATATATCTATAATCAAATTAATTAGATAGTAAAATGAAAAAAAAAATTAAAGTGAATTGGA

TGATCGAGATTCTTATAAAAAAAATTCCATCCAATTGCAATTGCAAACATCCATTTCTACAAGGATGTTGATCTAGTGTTTGATTTAAGGAGAATTTAGC

TTAACAAAGTGATCAAGAATTTAGAGTTAACTCTCATTTTTTTTATAGACGTGAGTTCAATTTTACTTAATATATATTATTAAATTTTTGTTTGGTGGGA

AGTTCTCTTTAGAGAGATTTCATATTGCCCAAGGTTTAATTGAATTTTTTTTATTAGCAAACGTTAATTATTGATTTGTTAGTTTTAGGTTTGCATAGTA

AATTTCCGGCTAAGCTTATGATAGCTATCTCAACATTTCGTTAACATCATGCTTCATAGTACTATATGCCTCCTTATTCTAGTACTTATATTTTAGGGGT

AAAGGAAAATGCAAGAAAATAAAAGCTGGGAAGTTTTCATTAAAAATGAAATCTTGTTTAGTTTAGAAGTTTAGATAAAGAGAGAAATATGAGGAGGTCA

ATACTTGTTTTTTCTAGGTCAGAAATTACGGATGTTTTATCTCACCCATGTTGTATGATTGAAATTTTACAAGTTGAAAATCAGAGTTTAACTTTTGATT

TTCTTCTCACTTGTTTGTATTAAAAAATTGTTTTCTAAAACAATTTCACATTACTTAACAATGAATACTTAACAATGAATGTATCATTTAAAGTCTATTA

AGTTTTGAAAATTGTTTCCAAAAATAAGTATTAAAAAACTAAAAAACAATTGGAAGATGTTTTTTTCCCCATAATCTTCTGCTGCGGTCTTACCTCAAGG

GTGTGAGTAATTATTGGAGTGGTGTGTGCTGAGGCATGAATTCTGTTGGTTTTGTGAAGATTTATTCTTGATTTAAAAAACTAAAGAATTTTTTAAAATA

GAAAGCAAATACAACTTAAAATTCCTCCTATTAGTTTCTAGTCACTTTTCTAATATTGAAAATTCAAAAAAACTTAGAAAATTTGTAAATTAGGATTAAG

AATTTAATTAAAATGCCCAAAATTAAACAAAATTTTAAACCGTTACTCAATTACATTTCACAATATATCATAAATTGATTATTTTTACAATAATTTATCT

TAAAAGTTATATATATTTATAATTATTTCTAATTAATTAATTAGTTACCATTGAAAAATTGTTTACGATAGCATTGCATGTGAAAGTTAAACTCTGTATA

ACTAGACTGTACTTGACATGTTCGATTGTAATTATGTTGATATGATAAAAGTAGCAAGTTATTTTTTGTTTTCCTTCCTCTTTTCTCTCTTCCGTTAAAA

TTCAGATTTTTTAAATTAAGCAAAGATTGCGTGAAGGAAAAATAGCAGATATTTATGAAAGGAATGAGGGAAATAGAGCAAACATAAAAAAATATTCTAC

TTTCTGATCAATATAAATTAAAATTCTGTATTCTCTAAAAGCAGTAGGATCAAACCCTCCTAACATGCTCAGCCCAAGTTAATGCAACTTGCACAGATTT

TGCCAAAACAAAACTATGTACTCTTGAAATTGGTTCTCAGTAACATCGATCAAACGCTTCAACATGTTTTTCAAGTTATGTTTCCTAGTTTTGAATTGCT

TAACTTTGTCTAGGTTTCCTCCAATAGGTTAAGAAATCATTGTTGTCAGGTACGATCCAACTCGTCAATTAAATTCTCATCTTTCCATGTATATTCTCTA

TTTTTGTTTGAAATGTAAGATGAAAAAATTAAATATATAATTTTTCTCAAATATAAAAAGAAATTCAACTAATTTTTATCTTATTTAATGTTATTACTCC

TAAAATATTTTTTTATTTAATTGATGTGCTAGTTTTAATGACTATATTTTATTTTTAATATCAGGTTTCAATTAAATAAGTTGAAAAAAAAATTAAAATT

AAAATTGATGTAATGAAATATGATTAATTAACTGTGTTTAGGTGATGTTTCATTTTTATTTTCCTTACATTTGTTACCAAAATAAGATAAATTTTCCAAA

TTCCACTTTCTCTTTCTTATCTTTTCTTAAATTATACAAGTTTGTAAATTTTTTTCCAATATTCACCATATTTCTATGTTGGCTGAAAAATCGGGTTTGG

TTACCCCGACTTCTGAATAAGTCTCTTTTTTTTTTTACTTTAAATTATTAAAATAATACAAATAAGAATCAAAACTTTTAATAATAAAAATTATTAAAAT

TGTCTAATAATATATTTGTTTTATTAAAAAAATTAATTTTTTAAAATAATTATTTTCTCCAATTTTTTATTAAAATAATTTTTATAATTTTTTTAAGAGA

TATACAAGTTTCTCTTTCTTATCTTTTCTCAAATTATACAAGTTTATAAAAAAAATTTCCAATATTCACCATATTTCTATGTTAGCTGGAAAATCGGGTT

TGGTCACCCCGACTTCTGAATAAGCCTTTTTTTTTTACTTTAAATTATTAAAATAATACAAATAAAAATCAAAACTTTTAATTATAAAAATTATTAAAAT

TGTCTAATAATATATTTGTTTTATTAAAAAAATTAATTTTTTAAAATAATTATTTTCTCCAATTTTTTATTAAAATAGTTTTTATAATTTTTTTAAGAGA

TATATATAGATAAAGAAAAATCAAAAAATTTGAGAAAATTATAGTATAATTTTTTAGTTACATGTATATATTTTTTTAGCTAAATTTATGTTCACGGGAA

GGTTTAGAGTAATGTTATAATTCTAAAAATGTGATTGTCATTCTTGATCCCATCAATGTCGCTGTTATTCACTTACAACTCCTTTGGTTTACTGCCACAC

GAATTAATGTGACAATGGAGATGAGTGGGGTTCTGATTCAAGAGAAAGAGGACTACAAAGGGTCATCAGGTTTTTCTATGAGATGGACGAAGACGAAAAT

GAACGGGAAAGTAGAAAAAATATATGAAATTTATCGGCAACATGATCATAACAAAAATAATTGTTCCAGCATACCTTAATCTTAAGTCTTTCCTTGACCA

AATATAATTGTTTAAGTACTTTATTTATAATATAATATAATGTTCTTCTATAAATAAATTGTTAAACAAAAAGTATAATCTTATGACATAAACAAACAAA

TTATAATTAGTAAGATAATGATATTAAAAGTTTTAATTTTTTCACTAAGCAAATATATTATTAGATAAAATTCATATTTTAAAAAAATGTTCACCTACAA

AATATCTTATATAAATTAAATAATACAAGATAAGTAATTATATTTAATAATATTATTTTCTTACTAAAAAATTATTTTAATAAAAAAGTATTTTTAAAAT

CAATTCAAAATATATTTATATAATATAATATTTGTATTATTTTAATAATTTAAATTAAAATAAAAATATAAACATATAAATTAATTTTAAAAAAAGAGGT

TCATACGGATGTCGGGATAGTAAACATAAAAATGTAGTGTACATTGAGAATTTTTTTACAAACTTTGTATAATTTGAAAAAAAAGATAAGAGAGAGAAAG

CGTAGTTTAAGAATTTTTTTTCCTAAAATAACATCTATTGGTCGTTCATTGTAGCACTCATTATTATTAGTTGAAATTTACATGAGTAGTACTAAATTGG

AAGCACAATCCTCTACGAATAATCATCTTATTTAGCATGACCCACATGATTTGAATCTAATAATAATAAGTGTGACCAAATGGAATAGGTTGGTCGCATG

TTTTATTATTAGAATATAATATTAATCCTTAATTTTGGCGACAATTTTAGTGTCCCTTAATCTTGTGATTGCATTCTTAGTATTCTAGAATGCTAGTGAC

TATATTATATCATAATTTTTTTTAGATTAACTTCTAGATATCTTCTAGTTGTTAATAAAAAATTAACATATTAAGAGATTCATTTAAGAAGCTCTCACAA

TAAATAGTTGTCCATATACAAATTTAGGTATCAAAACTCTTTTTTTTTTCCTATGTGAAAGACTTGTGAGTGTCCTATGATTAACTCATTGGGCCAGAGA

CTAATCCTACCCACGCCTTGTGATTGTCGTTAGCTCAATAAAATTTTATACACAAAGAAAATCAAACACTAGTTCTCTATCTTAAATCATTACACCAATC

ATATGAGTTCAGGAATAGAGACTTTAATCACATGCTTAAAAAAAATTATTTCACAATCATACTACACCATCTTAATTGCTAATGTTACATTAAGTATGGT

AATATTTGCCAAGCTTTAGATAATTTGGTGCACAAAATCCAAAGAAAAAATGAATTGTTGTATATTGAGAAGAATATGATCAGATATGGTAGATAGAAAT

GGAATCATTCCTTTTTGGGTTCCACGGAGTGGCTTAAAAATAATCATCTGACCCCACCGATGACTGGAAAAATGGCCCAATAATGGCCCAGTGGCCCACA

GCCTTCATAGCATTATGCAGGTCCATACTAACGGAAGGTGGCCCAAGAAAATAATAAGGTACAACCAGAAATTCTAAATTCTACCGAAGTCCATCGAACC

AACCAAAGGCAACTAATCTTAAAGGATTCAGCCCCAGAGAACGTGCAGAAACCAAAATAGTACGATATAGTTTAAAAGTAAAATTGTTTGATGAAATGTG

TATATCAAATAAGATAAATTTTATTATTAATAATTATAAATTATCTTAATAATTATGTTTACCAATGTTTTTTAAAATAATATTGATCTAAAACTGTTTA

CATTAATAATTCATGAAAATTAAACTCGTATAGAAAAAGAAAAGAAATAGTTTTTATTAATTATATTAAAAGTTTTGTTTACCATAATATCTAATTGATT

GAAAATATAAAACTTGTTATATTAATAATGTGTAGAAGTTTAACTCGTATAAAAAGAAATAAAAAATAAAAATAAAGATATTAAAAATTATGTTTATCAC

AATTTTTTTATTGAATGACTATTTAAATTTTTTTACATTAACAGTAGTACATGAAAATTAATATATATATATATATATATATATATATATCACTTTTAAA

ATAAAAAAATTAAAATGTTATGATAATTTATGATAAGAAGAAAAAGCTAATATATTTTTTTAAAAAAATTACATATTTTATTGATAACGTTGTCATTAAT

TATTATATCTTTATGTTTTTTCTCCCAGATTGCATAGCAATAATTCGTTCCTATTTCTATTTAAAGATTTTTTTGTATTCTTGTCCCGTGTTGGTGCACG

GATTCTGACTCAGTGATTGCTAAAATCCAAAACGTGTTATTTGACGATTCATGTCATGGCCTACGTGTAAACATGTTGGTTGACAAAAGTATGGCGTATA

AGGTACGTAAATCCAAAGAATTAATGACATGTACAAGATTTTATTGTATTAGAAAATATTGTTGATTTATAACCTAGGAAAACAAATATTTTTGTTTCAC

TTTTTCAACGCATTACTCACTTTTTAATTAGGGGTAGATTTAGATCAGGTTGTCTGACTAAACGCATTTGTTGCATTTATTTTAATATCTTGATATAGTC

TTCTTGTTATCATTTTAGACTTAATATAATTTATATTTTTTCCAAAAAAAAAAATTTATTATCACTTATTCCTTATTTTTTAGTCACCTTTCGTCATCCA

AAAAGATAAGTGCATTAAGATCTCTTCTCTAAAGTTCTAGACCTGGATCAATATTATTAGGATTCACTACTCCACTTCATTAAGAAGTACTGCCAATTTA

TCACTTTGTGCATGAATTAATTAATACAATTTACAAGATTATAGAAAAGGTAAATAGTAAAATTTGTTTCGAAGATAAATTAATTTATGAAAGATGAAAA

TTTTAAATTTAATTTTTCTAAATATGTAGAAACTGTGAAAAATTAGTCTTTATATAATTTAATGATCAAATCGGTCCCTAAATTTTGCATCTTAATGTCA

AATTGATCAATAAAAAAATATAACTTATTGTTAAATTAATCCTTGAACATTATAATTTAATTAATAAAATAATCTTAAAAATTTAACAGCATAATTAATT

TGTCACATTTTTTATATATTTAAGAATTAAATTTATATTTTTTATCTTTTAATAATTAATTTTACCAGATCACATTTTTGGAAATGAGTGACTATTTACC

TTAATAGGAAATAATGTTTGGGGAATGGGGTGTGACCTAAAGAGATAAAGGGAAATAAATGAAATAATAGGGTAAATGGGCTTTCTACTTAGTAGCAACA

CCTTTTTGAGATATTATTATGGAAATTGCAAGCAATCAAGCATTAAGGGGATGCCACGGCCAATTTTATTTATTTTTTATCGATAAATATTAATTATTGG

TTGTTAATTTTTTGTTAAAAGAGAAATTAAATTCTTGATCTTTTCTCCTTCTCTGTTCTTTTAACTGCCCAACCAACATTTTATAACTCCACTAGCAACT

TTCCCCCCCCCCCCCCTGTACTGCAAATATCATTGCTAGCTTAATGAAAATAAAATATCTTTTATAAATGAAAACTCAAAATTTCTTTTCCATGAAAATA

GCGTTTGTGGCCAGCAATAACGTCAAATTTTAACTCAATTTCATGACATCAGACATGGTACCCACTACTAGTATCAAGTAAATATCTTTTTCATTGATCT

GTTCTTGTTGGCTCCAAATGATGGCAGAAATCACTTACTAATCGATAGTGAAAAACAAAAGTTTGGTGCAAATTGGCAAATTGCTGAATGATAAGTGGTG

ACTTGCTTGTGAGAGTAGCATGGTATATTAATTTAATCATATAACTAAGACTTTGCGATTGACCATTCTCCACTCAATGAATAAAATTGTACAAAATGAA

CATCATTATCTTCTTTTCTCATTCTCAACTAAAAAATAAAGTTAAAAATTGAAGGATTAAATATTTTATAAACTCAACAAATATGAAAAATTTCGCATTT

AGTCCCATGACAATAATGTCATTTTGATTTTTCATTTTTTGAAATTTTGTCAAGTGTAGTTTGTGTTTTTTCGCCACCTCAATCGATAATGTTAGTAACA

GCGACAAGACTTGACACAGTCACATAAATGAAAGACTGGAAAGAGATTATTATTTTTTTATTTGAAAGGGGAAAGAGAGATTATTTTTTGAAAGGATTAA

AAGTGAACATTTTCTTAGTTTAAAAGGGTTTTGATATTTTTTTATTTAGCCAATGAAATTATAACATATATGCGTCCATATTACTTAAATATAATGGTAA

TATAATATTTCTATTTCAAGCACTTCAACTATAGAGATCTTACTTGCATAGTTTTCTTTTTATTTTATTTTACATATATTGATCCATGTTTGTGTTTGAA

AGATAATAATAAAGTTTGGGATGCAATATTTTTAAAATATCATACGGCATCACACTTTAGTTAATGAATCTTTATATAATTGGTTAGAATTTATTGAAAA

TCAGGTTTCAGCTTCTCATTCAATATAAATTAGGAGAGAATCATTAAATGAGAAGCAAAACCTACTAAAAATGGCAATTTCCAATAAATTTCAACCAATC

ATAGAGAGAGTTTTAGAGAGAGAGTGTCACTATCATCTCTCATTGCTGATCATATCTTAACATTATTGACTAAACTTTCAGGCCTATGTTCTCACTGGCA

TAGAAGTATAAGTGGAATTGGTGCTTCTGCTGCTGCTGATGTGGAGCCTAGCATAGCACCAGTGCAAATTGATCATAGTCAGCTTCTAATTGATGGACAG

TTTGTTGATGCAGCTTCTGGTTAATATCTTACCTTTATGTACTTAAAACATTGTTGTTTCATTCATATGAAAACATTACAATCTATTCTATAGAAACTTA

ATATAACATTTTAACTATTTTACCTTTGTTAGGAAAAACTTTTCCAACCTTTGATCCTAGAACTGGAGATGTAATTGCCAATGTTGCTGAAGGTGACACT

GAAGACGTAAATCGTGCTGTTCGTGCTGCTCGCAAGGCTTTTGATGAGGGACCATGGCCAAAGATGACAGCTTATGTATTGAAACATCATCTAATACTTA

TTTGTTCTACAAATTTCAATATGGAGCTGTTTTGATGCTGCATTTTTTCTTTTGGACTACAAGATGCTACACGTTTTTTATATTCAACAAATTTTAATCA

TTATCTTTGATGACCTTCTCTAGGAAAGATCGCGTATAATTTTGCGCTTTGCTGATTTATTGGAGAAACACAATGATGAAGTTGCCGCAATTGAAACATG

GGATAGTGGGAAGACTTATGAACAGGCTGCTAAGGTTGAAATACCTATGGTGGTACGCTTATTCCGATATTATGCAGGTAATTCCATTATCTTTGAATTC

TTCTATTTTGTAGTTATAGTATTGTATAACAAGTTGATGTTCTTTGGTATTCTTTCTGTTTTCTTCAGGTTGGGTGGATAAAATTCATGGTCTAACAGTT

CCGGCTGATGGACCATACCATGTCCAAACTTTGCATGAACCAATTGGTGTAGCAGGGCAGATTGTTCCTTGGAATTTCCCACTTCTTATTTTTTCATGGA

TGGCTGCACCTGCATTAGCTTGTGGTAATACAGTTGTAATAAAAACTTCAGAGCAGGCACCACTTTCTGCCCTTTATGTGTCAAAGCCATTTCTTGAGGT

AGCCTTATGTACCACACCTATAAATGTTTCCATTTATGTGTATACTGGCTTGGATTTCACTTTTGTGACCATCTTCTTATTCTTTTATAGGGCTGCAATC

ACAATTAAATTCCATACCTTCGCTATCGTATATGATGACTAGTTCCTCTTTTCTGTTCTTGTATGGCTTGACTGTTAGGAGCAGAGGACTAGCTAACTAC

ACTAACCTATATTTGATAATTCTTCTCTGATTCTTATTTCCTTTCCATGGTATATTTATTGTGATCATACAACGGATAAGGCTTGACACTCATGACCCAT

AACAGAAAAAGATAATAATGATAATAGAATTGCAAATAATAGAATAGTGGAAACATGCTATTTTGTTATAATTGTGACAGTGCTGTCGGACAAGGGATAT

GCTGGTGTCTTCTGCACCCTCTTGGGCCTACTCTCCCTTTGGACTGTATTGCTATCATTGACTTTGAAATCTCTACCCTCCAAAAATGAACCGAATATTC

TTGACCGAGATGAAGGCTACAGAAACATTTTCTTCTTCAAGCTTAATTGTTAGCTGTTACCAATGCTAGATCAATAACCTAAGCCTAGCCAAAATAAAAT

TAACAAAAAAAATACCTCTTCCATTAAAGAGAATGTGTATAAGTCTTCAATGTTACAATTGTCACCATTATTAACTGGACCTCTAGGAAATCGAAAGCTT

CAATTGAAATGAACTTAAGCCTGCAATCTCAAAATTGCTTACCACAAATAGATTGTGGTCCTGCATGTGTCTATACCTTTTTAATGGTCCTCACTGATGG

ATTTGATTCAATAGGCAGGACTTCCTCCTGGTGTTCTGAATGTTATCACTGGCTTTGGTGCTACTGCTGGTGCATCGCTGTGTAGTCATATGGACGTTGA

TAAGGTACTTCAAAAGATTTAGAGGAAGAATTCTTGAGACTTAAAAAAATGGGTAAAATGTGTTGTATGTATTTCAAGAATTGGTCAAAATTGGTTTTAG

TCTCTATATTGTAAAAACAATGGCTTTGGCCCTTGTATTTATGAAATGTTGTGAATTTTGTCCCTGCTCAAGTCAAAATAAAGTATAGGGTTAGCGACAA

AATTCACCACATTTTATAAATATGAGGATCAAATTTTTTTTTTTTAAATTTAGGGGTTAAAACCAATTTTGAGGGAACTAAAACACATTTCATCCAAAAA

AAATATATCTTCATTTGCCTTATTCTCTCTACAGTCTACAATACAAATATGCATTAATTTATGCCGCCTACATGGCCTAATAGCTAATAGTAAACTAAAA

GTTGACTGATTTTGTTTTTGTAGCTTGCTTTCACAGGATCTACTAGCACTGGTAAACGTGTACTAGAACTGTCTGCACACAGCAATCTGAAGTAACTCTG

GAGCTGGGTGGAAAATCTCCATTCATTGTGTGCGAGGATGCTGATGTTGATGCAGCTGTTGAGGCTGCACATTTTGCTTTATTTTTTAATCAGGTTTGAT

GATCCTAGTTTATTTTATGAGGAGTGATAACAATACACTCTACTTAAAATTTATTGAGAACTACAAAAGCATTAGCGAGACTCATTAAATAAAAAGTGAG

ATCCACAAAATTTGTGATTTCTAATAAATTTCAGCCAACAGTAAAGAGTGTGTTCAAAATAGTATGTTAAAGACTGTGTTGGTAGCATTTCTCTTACGTT

ATTATTCACAAAAATGAGAACAGTGCATTCTCAATTTTTATTTATTTATTGCATGATGATCCAATATGGTTCAAGCTGTTTTGTTTTGCTTGTCTACAGG

GGCAATGTTGTTGTGCTGGTTCTCGCACATTTGTCCATGAAAGCATATATGATGAATTTGTGGAAAAGGCTAAAGCTCGTGCTCTTAAACGTGTAGTTGG

AGACCCTTTCAAAAATGGAGTTGAGCAAGGTCCTCAGGTATCTATGACTACCTCCTATCTTCTATTACCTAATGAACCTTAAATCAAAGAGAACCTTTGG

TTGAAATAAGAATAACATCCTAAGTTTTTCAATATCTTTGGTTATAGCAAGTAAGAGAACCTTATGGTTTTGCAATTTCTCATTCATACTAACAGAGTTT

TCATTTTTTTTAACTATGTAGATAAATGCTAAGTAGGTGGCATCTTAAAGGGTGATGAATTGATAATTGTATGTGTTTCAGATTGATTCTGCGCAATTCG

AGAAGATCATGAAGTACATAAGATCAGGCGTTGAAAATGGTGCTACACTGGAATCTGGTGGTCAAAGAATTGGCTCCAAAGGCTATTACATCCAACCCAC

TGTTTTCTCAAATGTTCAGGCAAGATACTTACAATTAACAATGGTTTTATTTCTGAACAAGTAGTAACATAACTACAAAACAGAAGTAAATAACTAAACT

GATTTCCTTGTTATTATTACAGGACAACATGTTGATAGCAAAAGATGAGATATTTGGTCCAGTACAATCTATCTTGAAATTCAAGTATGCTATAAATCAT

TCGATAAAAGTGCTATCATGTTTGTACTTTGACATTCTTCAGTAGCTCCTTCTCTTAAATCCTACTTATTCTGTTTTGAAATAAATCATAGGGATCTAGA

GGAAGTGATAAGAAGAGCCAATGCAACTTCCTATGGCCTGGCTTCTGGAGTTTTTACTCAAAATATGGACACTGCCAACACCTTGATGCGCGCATTGCGA

GTTGGAACAGTATGGATCAACTGCTATGATGTCTTTGATGCAGCAATACCTTTTGGTGGATATAAGATGAGTGGACAAGGGAGAGTAAGGGGAATCTACA

GCCTTAGAAGCTACCTTCAAGTTAAAGCTGTTGTCACTGCTTTGAAGAATCCCGCATGGTTGTAG

>Glyma.05G231800 | Chr05:40924505..40929251 forward

CTAGTTCTGTTTGCATATTATTACAAGTCTCAGTCCCCAACGACTGGTTCTGCTTTCTCTCTGTTGCTTCGATTCATTCACACAACTCAACCATGACTTT

CAATAATGGCGATGCAGCTGCTGCCTCCCTCAACAAGGTCCCCACGGTCAACTTCACCAAGCTCTTCATTGACGGACACTTCGTTCACTCTGTGTCAGGT

TCATTTTTTTTATACCTATGCACTTTCTGTTGCCCCATCTAATTATCTGCTTCTTCTACCTTTCTATGCTCCTCTAATTTCTATTCCTTTTTCTGTATTT

TTCTTTCCATTGATGTACATGGATCAAAACTAAATACTCCTATTATTATGGTGTGACATAATTCGCATATATATAATTTTGACCTTAAGCATATGGTTAG

AACCTCTCCCATTAAGGAGGGATCCCAACAGATTGTCCCTTGTAAGTTTATGTTCAAACTGTAATTTAGAGGTTAATTAATTATCTATACTTGTGTCAAC

AGTACTACTATGGTGTCTCAACTCTCAACTAAGTCTTATATACTAATATATATGACTTTTGCTAAAACTCTTGTTGAAAAAATAGGAAAGACATTTGAGA

CAATAGATCCAAGAACAGGAGATGTTATAGCGAGGATCAGCGAGGGAGATAAAGAAGACATTGACATTGCTGTTAAAGCAGCACGTCATGCATTTGACAA

TGGTCCATGGCCTCGCCTTCCCGGTTCTGTACGTTATTTTTTCCCTTCTCTTTTTTTATACTATGCACAAGGAGATTAATATATGATAACTATAAATATA

AACAACATAATTAAGATTACTGCGGACTGCAGTAACTTCACTTGGCCGAATGATGAGGAATGGATTGACTTTATGCATTTGGGTAACTCTTTTATATTTA

ATTTTAATATCAACGTAAAAAAAATTTAACATTTGTATTTAATAGTATATTTTCACAATATTTCACAAAACTGATATTTTCTAATTGGTAATATGATTTA

AATTATTAAATAATATGCTCATGTCGATATTAATTATGTATGTTATAATTTATAAAAGAACTGACATTAAAGTTATATTAAAAGATTACGATAATCTTTG

AAATAAATATTATTAAGACTCGAGCATGCAAACGGCCATGTGATCCAGATTGCTATATGAATAATTAACATCAACGTTCTTACGTGATTTCTGATTATTT

AATGCCAAACCCATGTGATCCAGTTTGTAATGTCGATGTTATCAATTTCAATTTTTTTTTCAACCATCGATTCAATTTTATTTAAAACACAACTATGCAT

AAGTTACAAGTCACTCAAATGAGTTGGATTTATGAAATTTAAATATGACTTAAATTTTTTTACATTTAATTTTAATCGCGCAAAAAAGTTAACACTTCTT

TTATCGTATATTGCCGTATTATTTCATAATTTTGATACAATTTATACTTGAAATATAATTAAATTTGTCAAATGTTATTATTAGAATAAATATTACATGA

AAAATATATTGAAATTTTAATATATTATTACTCCTACGTGCAAGCGTGACTACGGATGTCGGTTTGGTATAAAACATAAAAAACACACCTAACGAGACTT

TTTTTTTTTAAAAGAAGTTTCTTAGATTCAAACGGCCGTTTGATGATTTTAAATATTTGAAACACACAAACTATTAATAATTAGCTGACCCTCCCCTAAA

ATTGGAAGAAGAAGAAGATCGAAGAAGATGTTATAGTAATGAATTTACATTGAAATTGTTGTATAAATATCATTTAGCAAGGGAGTCATATTACGGCTTG

AGGTAAGTAGTGATGGATACTTTCGATAAATATAGTTGGCTATAATCTCCAAAGTAATTCGTTGGTTGCTGTTAAATATTGATAAATTAAGAATTTGAGT

TTTGTTTAATATGATCATGTTTATATTTTTCGGGACTTTATTGATGTTTTAACTTAGTTATTCCAATAAATATTTTTTAATTAGCCACTGGTAATCTTGC

ATCATCAAAGTTTGTTTGTAATTTTCGAGTATCATGCGTCCAGAATGTACAGTAATTTTATTTGTACAATATTTTTTTTTATATTACGTTAGTTTTGTTT

GAGTGATATATAAAACAAGAGAATTACACGTTCAAAAGCCATCAAAGTTGTATAAATTTTTCAATACAATTTTATATATAGAGTTCATTAATTTTTTATG

TAGTATCATATATGGTAAAATTCTATATATTATCAACCAATTAAAAAGCATTCTAATTAAATCTTTAAGGTGCTTTTTATTTAAACTCTTGCATATATAT

TCAAAGCTTAGTTAAAGCATGCATCTCATCAAAATAAAAAATAAAGACAGTTTAGTTAAAGGGTACTGCACTTTTTTTTATGAGACAAAATTCTTTTGCG

ACGTATGAATTGAAACTTGACATGACGTACGTGAATGTGATAATCAGGAGAGAGGAAGAATTTTGCTGAAATGGGCAGAGCTAATAGAGGAAAATGCAGA

AGAACTTGCGGCACTAGATGCCATTGATGCGGGGAAGTTGTACCATATGTGTAGGAATTTGGAAGTTCCAGCAGCAGCAAACACTCTTCGTTACTATGCA

GGTGCTGCCGATAAGATTCATGGCGAGGTGTTGAAAATGTCCCGAGACTTCCATGCCTATACATTGCTTGAACCACTTGGTGTTGTGGGACACATTACTC

CCTGGAATTTCCCCAATACCATGTTCTACATCAAGGTTGCTCCTTCTTTAGCTGCTGGCTGCACCATGGTCCTCAAGCCCGCCGAGCAAACACCCCTCTC

TGCTTTGTTTAATGCTCATCTTGCTAAATTGGTATGTGCATGTGATCCATTTTCAACAATTAAATGATTCATTCATATCTTTATCAACCATGCATGCATG

GCTTGGAGATATAAGGTTGGTTGGATGATTAAGAAAGAAGAAAAGGAGGAAAAAGTCATGGGTTCGATCCCTCCTATTAACAATTAACGTTTGTCGATTA

AAAACAAATGCATACATGGCTCTTCATCACAAGTTACTTTAGCATAAAAGCTTATAAATTAGATTATTTTCCTTATTTTGAAGGCTGGAATCCCAGATGG

AGTGATCAATGTAGTGCCTGGATTTGGCCCAACTGCTGGTGCTGCATTAAGCTCACACATGGATGTTGATAAGGTAATTAAATTTACATGTCAAGCATAT

AGAAGTACCATAAAATCTTAAATAAGACTAGTTAAATATTATATTAATCTATATATTCGAGTATATGCTTCTTTACTTGGTGGTATGTTCAGGTTAGCTT

TACTGGTTCAACACAAACAGGCCGTGAGATAATGCAGGCTGCAGCTAAGAGTAACTTGAAACAAGTTTCACTTGAATTAGGAGGCAAGTCACCCCTCATA

ATTTTTGATGATGCTGATATAGACAAAGCTGCTGAGCTAGCTCTACTAGGCATCCTATATAACAAGGTGAAATTGCTTTCAGGAAATCCTTTCTTTTCAA

ATAGCATCTAGTTAATTATGTCTCTGCTAACATTTAATTTCTTCCCATGTGACTAAAGGGAGAAGTTTGTGTTGCAAGTTCCCGTGTGCTTGTTCAAGAA

GGGATCTATGATGAATTTGAGAAAAAACTGGTGGAGAAGGCAAAAGCTTGGGTCGTTGGGGATCCCTTTGATCCTAAAGTTCAACAAGGCCCTCAGGTAA

GAAAAATAGGTAGTGGCACAATTATTTCTACATTAGTTCTAATATATATATATCGGAGATTTTCTTGTAGCAACATAACTTAATATTTCAAAAATTGCTT

AGCAGAATTTACAAACAGAAAAAGGAGAAATGACCGGACCAAAAAAACAGAAATGAGAAATAATTAAGATCATCGTCTATTGGCTAACAATTTTTCTGTG

TATATTGAAGGTTGACAAGGAACAATTTGAAAAGGTCCTTTCATATATTGAGCATGGAAAGAAAGAAGGAGCTACCCTTTTGACCGGGGGTAAAACAGTG

GGAAACAAGGGCTACTTTATTGAGCCAACAATTTTCTCCAATATAAGGGTAAATTCCAAGTGCACAAAGTTATTAATTAATTGAGTATCTTCAGCATAAT

TAGATAACTAATCATCATCTTTCCTTTGTAAAATTTGTTAACTACCTGTTTAGGAGGATATGCTTATAGCACAGGATGAAATATTTGGCCCAGTAATGGC

ACTGAAGAAGTTTAAGTAAGTTAATTTTAATGTTATTGTGTCATGTTTCTTGTTAATTAACATGTTGAAATCCTCATATTATTATGTTAATTATCATGAT

ATATGAACAATAGGACCATTGAGGAAGCAATTAAGAGTGCTAACAATACCAAGTATGGCCTAGCAGCAGGCATAGTGACCAAGAATTTGGATACGGCAAA

CACAGTGTCAAGGTCCATTCGTGCAGGCACTATTTGGATCAACTGCTATTTTGCCTTTGGCGATGATGTTCCCTTTGGAGGGTATAAGATGAGTGGATTT

GGAAAAGATCATGGATTGGAAGCCCTTCACAAGTACCTACAAGTTAAATCTGTTGTTACTCCCCTTTACAATTCACCCTGGCTTTGAATGTCTACTGCAT

ATATCATATATGGATCTTGGAAAATAGTCCATTCTGGCCTTCTGGCCACTACCTATCAATTTGCATCTGCCATGAGCCATGGAAATTCAGTTCTTGAATA

GTGGTGTCTGTTATATTATCAAAAAGTTGGCCTTTTCCTACTATTCT

>Glyma.05G231900 | Chr05:40934314..40940453 forward

AAAAGTCCTAAAGTTGCGCCAATAATTCATGTTCTTGTCTTTGCATATAGTCCTAATTAATCATATTGCAACATTCCACTTACCGACACCATCTCTACGA

CAACCTCCTTTCTCTCTATCTCTAGCTCGGATGAGTGCTCTCTCTAACTCCAGTAGTAGCCACGGCAATTCCTTCCTCAAGATGCCCGCCATCAAGTTTA

CCAAGCTCTTCATCAATGGAGATTTTGTTGATTCCATATCAGGTTCTTGAATTTTAATATTACAACTATTGTTAATGCTATTTTTAATATTTTATAAAAA

AATGTTACTTTTAATTAAAACTAGCTTTATAAATAATTACCGTCAATTTTCAATTATCAAAATAATAGTTATTAAAATAAAATGCCAATTTTGATAAATA

AATAAATAAACAAGAATAACAGTAATTATGAAATGTTGATGGGGGTTGACATTGATGAAAGATCTAAAACGAATTTTTATCTCTTCTCCATAAGTATGAG

TTTGAATCCTCTTATCGTCATTTATTTTGCTTGGTAGACAATTTTTCTTAAAGAGATCTTGTGATGCCTAAGAGATAAGTTGAGTAGTTAAATTGAATGA

AACTACTCATTTGCAATGGAGTTAAATTGTGATTTAGGTTTATGTAGTAATCATCTAGGAGTTTAAAAATGGTGTATTTTTTTTAATAAATATATAGTGT

ACGGTTAATCATTTAGATTTCAAATATTTTTAAAAAGTATAATTTATTTGGATTTCTTCTAAATACAAAATAGTCGGATATATTTCTAACAAACTTCTTT

ATGATTATTTGACTTGAATTAAAAAGGAAGGACATTTGAGACTATAGACCCCAGAAAAGAAGAGGTAATTGCAAGAGTTAGTGAGGGAGATAAAGAAGAC

ATTGATATTGCTGTTAAAGCAGCACGTCAGGCATTTGACTCGGGTCCATGGCCTCGCTTGCCAGGCTCTGTAAGTTATTCTTTTCCTTTTCTTTAATTAG

ATATTTTTTAAATTAAAATATACTTTTTTGTCTTTATAAATATCAAAAAATTCAGATTTTATTCTTATAAAATTTTCGATATATTTTTTGTTTTCGTAAA

ATTAAAATGTGTTATTTTTTTACCATCAAGTTTGGACAAATCCATACCTATATAACATCTTGTAAAACTTAAATGTTAAGCTAAAATATGTTTCTGATTC

ATATAAATATTAAAATGTTCAGAATTTATCTCTACAAAATTTTGAATTTTTTTTTCATTCTCTAAAAATTGAAATATGTTACTTTTTGACTGAACGTAGG

TTCAGGTATGTCCGAACATATGTGGTACTCTTTCATTAGTTTCATTGATGTTTATGGGAGTGGGTGATTCCTGAAAATTAAATATCGTCACAAAATATAG

AAAAAAAACCCTCCCACAAATTTATAAATTCTTCTTTCTTTTCTAATTTTCTTCATCTTCTTCAAATTTAAATAAATAATTTTTTATGTATTTTGTAGCG

GTTTTTAGCCTCAACAAATCACTCACTCTCTTAGTTATTAATGGAATCAATGAAAGAGTGACACATAGGTTCGGATACCTAAACCTACATCCGGACCAAA

AATTAATAGATTTTAATTTTTTGAAAATGAAAAAAAGACTCAAAATTTTATAAAAATGAATTCCAATTATTTTGATATTTATAGAGATAAAAAATATATT

TTACCTTATAATTTAGGTTTTAAAAAATGTCAGGTAAATTCTGATTCCTCCAAGCTTGACTTCAAGTCAAAAGCAATATATTTTAATTTTGCGATGATGA

AAAACATATCAAAAATTTTACAATGACAGATTCTAAACTTTTTAATATTTATAGAAACGAAAAATATATTTTAATGTATTTTTTAATATGTGCAAAACCA

TCCCTAATTTCTTACATTATAAACTTCAACGTGTATTTTTAAATTAACGAAAAAATGGAAATTATGATCATTAATTTAAAATTAATAATTAAAATTACGA

TAATATATGTTTACTCAAAACTTTCGAAAGCCGGTACCTGTATACACATACCATTATCAATGACCTATTCGGCTATTCTAATCGAGGCCAAGCAGTCCAA

TGTTGAACAAGATACTTGTGAGCTCATGTTTGACTTAGACGGANNNNNNNNNNNNNNNNNNNNNNNNNNNNNNNNNNNNNNNNNNNNNNNNNNNNNNNNN

NNNNNNNNNNNNNNNNNNNNNNNNNNNNNNNNNNNNNNNNNNNNNNNNNNNNNNNNNNNNNNNNNNNNNNNNNNNNNNNNNNNNNNNNNNNNNNNNNNNN

NNNNNNNNNNNNNNNNNNNNNNNNNNNNNNNNNNNNNNNNNNNNNNNNNNNNNNNNNNNNNNNNNNNNNNNNNNNNNNNNNNNNNNNNNNNNNNNNNNNN

NNNNNNNNNNNNNNNNNNNNNNNNNNNNNNNNNNNNNNNNNNNNNNNNNNNNNNNNNNNNNNNNNNNNNNNNNNNNNNNNNNNNNNNNNNNNNNNNNNNN

NNNNNNNNNNNNNNNNNNNNNNNNNNNNNNNNNNNNNNNNNNNNNNNNNNNNNNNNNNNNNNNNNNNNNNNNNNNNNNNNNNNNNNNNNNNNNNNNNNNN

NNNNNNNNNNNNNNNNNNNNNNNNNNNNNNNNNNNNNNNNNNNNNNNNNNNNNNNNNNNNNNNNNNNNNNNNNNNNNNNNNNNNNNNNNNNNNNNNNNNN

NNNNNNNNNNNNNNNNNNNNNNNNNNNNNNNNNNNNNNNNNNNNNNNNNNNNNNNNNNNNNNNNNNNNNNNNNNNNNNNNNNNNNNNNNNNNNNNNNNNN

NNNNNNNNNNNNNNNNNNNNNNNNNNNNNNNNNNNNNNNNNNNNNNNNNNNNNNNNNNNNNNNNNNNNNNNNNNNNNNNNNNNNNNNNNNNNNNNNNNNN

NNNNNNNNNNNNNNNNNNNNNNNNNNNNNNNNNNNNNNNNNNNNNNNNNNNNNNNNNNNNNNNNNNNNNNNNNNNNNNNNNNNNNNNNNNNNNNNNNNNN

NAGTAAATATCGGAATGACAAATGTAAGTAAAAAAAAATGAATATTATTTAAGAATGAAAAAGTTGAAGAACTTTTAAATAGAAAAGGTAAAAATAAACT

TACATGTGTGGTTTATGTTTATTGACGAAGATCTTTATCTGAAGTTTTATTTTTCTTGTTTGAATTTTTCCTTCTATCTATCACTTCTAATCCTCTTCAA

ATATCTTAATTAATATTGACATCGTATGGTATACACGCAGGAAAGGGCAAAAATTATGATGAAATGGGCAGACCTAGTTGATGAAAATATAGAAGAACTA

GCAGCATTAGATACCATTGATGCTGGAAAGCTATACTATATTAATAAGGTAGCGGAAATTCCTTCAGCTACAAATGCGTTACGGTACTATGCAGGTGCTG

CTGATAAAATTCACGGTGACGTATTAAAAATGAACGGGGATTTCCATGCATATACACTTTTGGAACCAATTGGTGTTGTGGGACACATAATTCCATGGAA

TGCCCCCAGCCTCTCATTTTTCATCAAGGTTAGCCCTTCCTTAGCTGCAGGCTGTACTATGGTCCTCAAACCTGCTGAACAAACACCCCTCTCTGCTTTG

TTTTATGCTCATCTAGCTAAGTTGGTATGTATCAAGACATAGTTTCATTTCCTTATCAAAGATGACCCTCACAAAGTTTACTTGAATTTAGGCTAATTCT

ATTGGGTTTGTCCCTTTTAAAAGGCTGGAATCCCAGATGGTGTGCTTAATATAGTACCTGGATTTGGCCCAACTGCTGGCGCAGCAATAAGCTCACACAT

GGACATAGATGCGGTAATAAGAAGTTACATGTCAAATATTTACAAGTACTAGTCTCTTAATTGATTTGACTTGTAAAATTTCAATTTCCTTGATTGTGTT

GATCTTCAGGTCAGTTTTACTGGTTCAATTGAAGTAGGGCGTGAAGTGTTGCAGGCTGCAGCTTGGAGCAATTTAAAACCGGTTTCACTTGAATTAGGAG

GCAAGTCTCCTCTCATTATTTTCAATGATGCGGATATAGACAAAGCTTCCGAGCTTGCTCTCTTTGGCATCATGTCTAACAAGGTGAAATTCTTTCCTTT

CAAGGAACATCTAGTAATACATACACATACACACGTATAATATACGAAGCATATCAAATTAAGTGAGTATTCTTCTTGTTGGAAAAATGTGAACTACAAA

TGTAAATTATTAAATCTTATTTGAATAATCAAAATTAAAAGAAAAATACAAAAGAGTTTTCAAGTGGTTTCCTCCTTATCAACTAACTTTTAATTTTGAT

TATTAATATATGGTTGAATGAGCCATATTTATAGATGTAGCCAATAAATTTTGTATTTCTTCTTTGTGCTTTCAGGGAGAAATTTGTGTGGCAGGTTCTC

GTGTGTTTGTCCAGGAAGAGATCTATGATGAATTTGAGAAGAAGTTGGTGGAGAAGGCAAAATCTTGGGTCGTTGGGGATCCTTTTGATCCCAAATCGCT

GCAAGGGCCTCAGGTAGGGACAATATATATAGTAGCACGATTATGCTTACAATAGTTCTAAAACGTATTTGATCCTTTGTTTTTACTAGTAACTCTCTAA

ATAGGAAAATGAATTTTAGTAATTAGTACAAATTTATTTGTTCAAATAATACCAAATGACATGGTGTTAATTTTTTTTTTTATCAACATTCCCATATATT

TCTAGGAGCATTTGTTTCGCGTCATCTTCTCTTTTTTATATCAGGAATGTGAGAATATAAATGAGTATTCTCATATTTATTAGAAGGCTATGATAATGTA

TTCAGGAATATATTTTTATTTTCAAGAAAATTGTGCTCTCACATTGTAAGTAGATTTTATTCCTATGAGTAGGAGGTGAATATCTATATTCTTATGGGAA

CTCTCTTCTACTCTCTTTCACTTTTAGTTTTTAAATTTTTTTAATTATATAACTCTTTTCAAAATTACTCAATTCTAACCAAACATATTCGTGTAAATAA

CATTTCTGGTATATTACTCGTAGAAATATGATTCCAGAGAATATAAAATTATAATGTGAAACAAATATCTTTTGGTCATCATCTAATGTATCTTGTCTTG

TTTGGTGATGGTTGGATGCACACTATTTTTTTACTATATTGTTCGTCTAAAATAACAAAGTTATTCCAATTATATAGTTCAAACATTTGAATAAATATAC

GTTTACTTAGTCTTTTATCCCATTTTCACTATATTTTTTGGTGAAATTTATCATAACTTGTTTTCTATTATTATAGGCTGACAGGAACCAATTGGAGAAA

ATACTTTCATATATTGAGCACGGAAAGAGAGAAGGAGCCACCCTTTTGACAGGAGGTAATACAGTGGGCAACAAAGGTTACTACATAGAACCTACAATTT

TCTCCAATGTAAAGGTAAATTTTAATTCCATCAAAGTCTCAACCTGGGTATCTTCAGCATAGTTTGATAACTAACCATCGTGTTTTCATTTTTCTTCTGG

AAATTAAAAAAAAAAAAATCCTGATTAGGAGGACATGCTAATAGCACGGGATGAAATATTTGGCCCTGTACTAGCGCTGATGAAGTTTAAGTAAGTTATA

GTCTAATTTAATGGTTTTTGTTGTAGTTGTTTAGTACAAAAAAAAAATGTGTTTGTTGGCTTGTTAACGAAGGTTGAATTAAAGTATATATATGACATGA

ACAGGACCATGGAGGAAGCAATTAAAAGTGCTAACAATACCAAGTATGGCCTAGCAGCAGGAATTGTAACCAAGAATTTGGATACTGCAAACACTATGTC

AAGGTCCATTCGTGCAGGCATTGTTTGGATCAACTGCTATTTTACCGTAGGGAGTGATGTTCCTTTTGGAGGGTATAAGATGAGCGGATTTGGAAGAGAT

TTGGGATTACAGGCCCTTCACAAGTACTTACAAGTTAAATCTGTTGTAACACCTATTCATAATTCTCCTTGGCTTTGAATAGTTGAATGTCTCCTACGTG

AGCACAAATGTGAGTCTTTTCATTTGGAATAAATTACACTTTAATTCCTTATAATGTGTGAATTGTCTCTTGTATTATTATGATTACTTTGTTTTATTAC

CACGTTGTTAACTTAAATATAATTTTTAGAGTGAAACAAC

>Glyma.07G087400 | Chr07:8068697..8073551 reverse

TTGTGCTACATATAAATAACTCTTGTTCTCCCTCTAAACACTCTCTACCCTGCCTCCAAAAACTTGTTGTTTGGCACTTCTTCACACTCAGCTAGCAGAT

CAGTCACTGAAGTGGATAAGAATGGAAAATCTCAGCAATGGTCACTTAGAATCCTTTGTCAAGATTCCAACAATAAAGTTTACCAAGCTTTTCATCAATG

GAGAATTCCTAGATTCTGTTTCAGGTTCCAATTACTTTCCTCATCATTTACCTCTATTCTACAACATTAGTTAAATGTTTCTTCCTTTATATGTGAAGCA

AATAGTTTATGCACTGAAAGTTGTAAAGGATTTTTTTTATGTTGTCTAGTTACAAATCATAATATGTTATAAATATTTTGTTTTTTTTTTGTAATAATTT

TTATAAAAGTCATATCAATACTGATTTTTTATTAGTTGGTTATAGTAGAAATTAAACTCGTATATATAAGAAACAATATTTTTACATAGAACCAAAATGG

ATTATACATGCACCTGATATAGAAATATATCTTTATTCAAGAAGTGGAAGCTTTATTTTGTAATCACATACAATTTGGAAAGTCCATTTAATAGGGAGGA

GGGGTTGGTTTGATAAGAATAGTAATGAATGATACAACACCATCCTCTTGACATGCATTCAAGTGAGAATTGTTAATGCAGGTTAAATTGTTAGTACTTT

ATTTTATTTCCAACTAATTTAAAATTATTCTTTTGCAAGTAAAAACGAATTTAAAACTATCAGATAACTGCAGTACACATTTGTTAATCTATATTATTTT

GTTTCTTTCATAGTTTCAAAATATTAAAGAGATCAAATTATATAAAAAATGCATAGAGATCAAATATCAATCAAGACTATATTAACCACAAATTAACTTT

TGTTTTCTAACTATAATACGAGCAAAGATTTCATACAGGGAACTATCTAAACTTAGCAAGAGGAAATTGAACTATAAATATGTGAGCATGAGCTTAAAGT

GATATAATTTTTGCTTGTAAGCTTGCCTAATCATAGCCAAGGGTCATGATTCCTTCTTTTTTTTTATTTTTGAATAATTAAATTGACTTATTAAAACTTA

TATGCATTTGGAATTTTTCTTTTCTGGAAAAACAAAAGGAAAAACGTTTGAGACCGTTGACCCAAGAACAGAGGAAGTGATTGCTGAAATAGCAGAGGCT

AACAAAGAAGATGTTGATATTGCTGTGAAGGCCGCACGTGAAGCATTTGATTGTGGTCCATGGCCACGCATGCCCGGTGCTGTAAGTTCATCTCTTCACC

TATCTTTTATTTCAATTATAACTATAATTAAATGAAAAGTTGTGTTGGAAAAAACCCAATTCCACATCCGCTATAAATAAAACCAAATTAAAGTACATAA

GTGAGGAATAATCCTCACCACTTGAGTTAATTTTTGGGATTGAGTTAGGTCAACTCACATTCTAAGATATTATCAAATAATATTCAAAATTAATCCAAAA

GACCACTCATCATTTATTATCGAGGCACCAAAAAGAAGTGGTGAACATGAAGGGATGTATAACCCAAATCACACATTAAATAAAATTAAAGTCAAATTAA

AATATATAAATTGTGCAATCATCACTGCCAAAGCAGAACTGAGTTTTCATTTTCAAGGTAAACAATTGTCTGTCCAACATTATTCATAACGAAGTTGAAA

AAAGAAATTGAATTGTTTGAAAACAAATGCTTTATAAACTATTCTAGTCTCTGTCCCAAAAAGAAAAAAACTATTCTAGTCACCACTTTTATTTATTTAA

TTAGTTGCTGTCACGAAGTTTTGTCAAATTTTTATGATCCCTCTTTTCTTTATCTCTATTAATATTCCTTAATCGTCTGAAAAACATTAGACTGGCACCT

TTATACATACATAAGAAATACATTATACCTTCTGTCCCTTGTAAGGGAGTTGCTTAAACGTTAACAAAAAAAAAAAGATTATGTGCAATTTAAAAAAAAT

CCCTAAGAGTTAGGGAGAGTTAATATAATTTGCTCTACTTATTTTCATATGGGGTCTATTTCAATTCGTTTCTCTCCATGGTTATCAAAATAAAAATAGT

TTAATTTGCATGTCCTTTCAATATATTTTTATACCTACATCACCATTTTACTATCTTATCATGTTTATTAATCTGATACGACAATAAGGTGAATTTGTAT

TGAATGTCAATATAAAAATGATAGTATAAAATTATATGAAATCGAGTTTGCAAAAGGTTCACACACTATCTAACGTACAACTTCTGAATAATAATAATAA

TAAAGAAGTCAAGTCTCAATAATTTTACACGCAAAAGATAGGCAATCAGGCAATGTTCTCAAGCTTTCAAAAGCAGTGAGCAGCATTAACTAACTTGTTA

TGATCATGGGATTTTGGACAGGAAAGAGCAAAGATTATGCTGAAATGGTCAGAGCTAATTGAACAGAACGCAGAAGAAATAGCAGCATTGGACACCATTG

ATGGGGGAAAGCTATTCAGTTGGTGTAAGGCTGTGGATGTTCCTGAAGCATCCAACATCCTACGTTACTATGCCGGCGCTGCTGATAAAATTCATGGAGA

TGTGTTCAAAACATCTCGTGACCTCCACTTGTATTCTCTGATGGAACCTGTTGGTGTTGTTGGACACATTATCCCTTGGAATTTCCCTACCGTCATGTTC

TTCGCCAAGGTTGCCCCGGCCTTGGCTGCTGGCTGCACCATGGTCATCAAGCCTGCTGAGCAAACACCTCTCTCATCACTCTTTTATGCTCATCTTGCTA

GGCTGGTATGGTTTAACACATTTGTTTATTAGTTGCTTATCAAAAGCAGCTAAACTAGCATTTGGTAGAGGTGTTAAAATAGGACAGAACATAACATGTT

GGAGATTTCACATTGTCTAATGATACGGCCCAAAATGGTGTATATAAGTGAAGAGACTCTCACTCCATATTACATTAAGTATTTTCGCCATATCAATAGT

CAATGTGTAACTTAATATATGTGTTCTGTTTTATTTTATGTTTGGCAAATCTTAAATATAGAACATGGTGTAAAAAAGTGGTACATTTTGGTTCCACCCT

TTTTAATTGAATCAAAGAAAATGGAACACAAGTCTTTTGAGTCAAACGCTACCTTAGGAACATTAGCATGTGACATATGTTGCTATCTTGGAAATTTATG

TTTGGTTTGTTAGTATCATTAATACATGGAAGATGGACATGAATATAAGTTGACTAGGTGTTTTTTGTTTTCCCTTTTCATCAAGGCTGGTATCCCAGAT

GGAGTGCTGAATGTAGTACCCGGATTTGGCTCAATTGCAGGGGCTGCAATAAGTTCACATATGGACATTGATGCGGTAATTTGAGTGTGAAACGTTCTAT

AGCATTGATCAAGTACAAGGGTATTATTGCATTTGTCACCAACTCCTAAGGTTTGCTGTGATTATGTTTTATGCTACAGGTCAGTTTTACAGGTTCAACA

GAAACAGGTCGTAAAATAATGCAGGCTGCGGCCTTGAGCAATTTGAAACCAGTTTCACTCGAATTAGGAGGAAAGTCACCAGTTTTGATTTTTGATGATG

CTGATGTAGACAAAGCTGTTGACCTTGCTCTCTTTGGCATCCTACATAACAAGGTATATTTTCTTCCCAGGAAATTTTATAACTTCGAAAAGAACAGACT

TATACTGTTTGAAATACTATAATTTTCAGGGAGAAATCTGTGTTGCATTCTCCAGAGTTTATGTCCAGGAAGGGATTTATGATGAATTTGAGAAGAAGGT

AGTGGAGAAGGCTAAAACTTGGGTAGTGGGAGACCCCTTTGATCCTAAAGTTCAGCAAGGACCCCAAGTAAGATTACTCTAAATTTGCACAATTGTACAA

CTATGTTCACACTAGAATTTAAGTGATGTACCTTATTACTGGAAACCCTCTTAACATATTTTCTTTCAATCTCCATGTAGCTGCTAGCATAAAAATAAAA

TTGTTAAATGTAATTCTTCTCTGACATGATTTTTTAATGCAGACTAGTAAGGCTCAATATGATAAAATTATTTCCTATATTGAGCATGGAAAGAGTGAAG

GAGCCACACTGTTGACTGGGGGTAAGCCAGCGGGCAACAAGGGATACTACATTGAGCCTACCATTTTTGTCAATGTTAAGGTGCATTTCAACTCTAAAAG

AAGCATGAAACCAGCTATCTTTATCATAATTGACAGTCGACCTTAATTTTTTTGGTTTCTTTTTGAGAAATTCATTTAGGAGGACATGCTAATTGCACAA

GAGGAAATATTTGGACCTGTGATGACACTTTCCAAGTTCAAGTAAGCCTATCTTATATTCCTTTTCGCTTAGTACAAGAAAAGAAGTTTGTTATTGTGCA

TTTGAATGTTGAATCAAGTATGGTATGCGTGCAACAGGACCATTGAGGATGCAATTAAGAAAGCCAACAATTCCAAATATGGCCTAGCAGCAGGGATTGT

GACCAAGAACTTGGATATTGCAAACACTGTATCAAGGTCCATCCGTGCAGGCATCATTTGGATCAATTGCTTCTTTGCCTTTGATATTGACTGCCCTTTT

GGAGGGTATAAGATGAGTGGATTTGGAAGAGATTATGGACTGGAAGCACTTCATAAGTTTCTCAAAGTTAAGTCTGTTGCAACTCCTATTTACGATTCTC

CTTGGCTTTGAAACTTCATTCTCCGAGGGTGTTTGTAAACACTAAACATATACATCATTTGGATCAACTACTTAATACAATGTTTTTTTTTTCTCTTTCT

TTGGATGCTAAGCAATAAGTTTTAAGCATCTTCTTAGTAATAATAATTTATATAC

>Glyma.07G087500 | Chr07:8093667..8100429 forward

GGCAGGCTCGTGAGTTGTATATGACATGTTTCTCTGTGCACCCATTGTTCTTGTCTCAAAGTAAAAGTGTGTGTATATAAAAGAGGTCCCTCTTCTCAGT

TCTTTGAAAACCTCAACAACTCAACACGTTGCCTCTACTCTCTGCAGCTTTTAATTTGTTTGCAGGCACACAACCATGGCTGCTCTCTCCAACGGCCACG

ACGCTTCCTTCTTCAAGATGCCCTCCATCAAGTTCACCAAGCTCTTCATCAATGGAGAATTTGTTGATTCCCTTTCAGGTTCTGATTTATTAACTAGTTA

GTTATGCGCCTCTCTTCTCTCCTTAATTTCCCTATATATATATATTCTTTTATTGTCTCACTTTTTTATTTTATGTGGAAAAAGTTATTCCTACACTGTA

TTTTTTTATGCATGTTGTTGGTGTAAAAAACCTATGGACTTTGTCTATAACTAATTTCTCTCGGAAAAACGTAGTTAATCACTCGGGTCTCCCATCTTGT

GTTTTACACTAGTTAAAATTAGAAAAAAACTTAAATTTTGATTAGAAAATAACACTAATATATAACACTTAAAAAAATGGATTTAAATTTTGTCATCAAG

TGAAACGGAAGAAAATTTGGATGGATAATGATAAAATCCTTTGTCTGCTAATCCTATTCATCCTTTCCTGTTCTGATTTTTTTATTTTTTTTGGTTTTTG

GACCCAATATCTTTTATGTGAAAAAGGAATAATATAGTACACCATACGAAAATACTAACGACAATTCCAAAGATAGGGGGTGCAAACTCGTTGTATAGGT

TATTTTTATTCAATGGTTTGTGTGAAGCAATGAATAGATAAGACTTGCCCCATTTGATCCAGCCTTTGCTCAACCAAGGAACATATTCATGAAAAAGGGA

ATTTATTAAAAATGAAAGGCCAATAAATAATTTCTATTTTAAATTTGACAATACAACAATAACGAGTACGCTTATCTCGAGGCATGAAATTTTATCTATG

ATTACGAATGTAATAATTTTATACTGTACTTATTATGAGTTTTAAAAAATATTTATAGGTGCGGACTATGTTAAAAATGTAATAATAATTTTTTAATGTA

TGATAACAGTACTAATATAGTATAATGTATTTTTGGTATACATTAGTCAAGGTTAATCTTGTATACATTAGACCAGCCATAGTGAAATATTGAGATACGA

ATTTAAGCTTTCATACTAATTATAAATATTCGATATATTTTACAGTTAAGTCAACTTTAGTTTGTCAGTTTATTTATATTCTCGTATTAAAAGTTGGGAA

AGTTAAATGTAACTGTTATTATAATATCTTGAAAAGCTCCCAAAGGTAGAAAATGCACCATCACAACTATCAAAGGAGTGGCTAATAATATATCAACCTA

TCAACCAAATATTCAAGTGGTGAATTTTTTTAAATTTTTTTTCTTTAAATAAATTGTGAAAACCTTCCTGAGTTTTTTTTTTTTTTGCTTACCTTCTTTA

TATATTTAATTTTTGTTTTATAAAATTGGAAAAAAAAAAAGGGAAGGAGTTTGAAACAATAGATCCCAGGACAGGAGAAGTGATTACAAGAATCGCAGAA

GGAGCAAAAGAAGACATTGATGTTGCTGTCAAAGCGGCACGTGACGCTTTCGACTATGGTCCATGGCCCCGTATGCCCGGTGCTGTACGTTATCATTTCT

CCATTTGTTTCTCTATAACTGTTATGAGTTATTAGTATGACAAATAATTTGCGGGTCTCATTAATTTTTTACTGCATTTTTGGTAAGGAATAAAAGTACG

TAAGAAAATTTTATTTAAAAATCCGTTAAAATACTAGTATACTCTCTCGTATTCTTATCTTAAGATCTAAATCAAAAGATTAACTTAGTCATTTTTATAA

AATTGAATATAAAATGTCAAAGCATTAATTATTTAATTCGTAAAATTTTAATTATCTTTGACTTATTTTCAGAATCAAATTTCCGAACTGAATTCTTAAA

GATGCCGGTCAACTTCATCTTGTTGTGCGGGCATATATTAATTTGATTTATTTTCTCTCATCAATCTTATCGTATTCATTAATGATGCCTTTTTCGTTTT

GACATTTTCTTCCCACTTGTGGCTTGTGTAGCATGCTTCACGGGTCATTCCCACACGTATGAGTGTGTGTAGGGTGGGTAAACGGGTCGGCCCGTCCCGC

GTATGGCCCACCCCATAAATACGCATTTGATAGTACCACCCCCATAAATTATTTTTGTCTTTAAGAGTATTTTTTCATCCATAAATCAAATATACAATTA

CTCTTTTTTAAAATAATTATTGTCCTAATTTGTTTACGTAAAAATATATTAAAAAATAATAATTTTATAAAATTAATTTTATTATTAATATTATATTAAA

AAGTTAAAGTGATACTCATTAAAAATATTAATAAAAAATAATTAATGTTATATTAAAAATTAACATTAAATTTATTTTAAGAGATTTTCATTAAAAATTT

AACAATTATTTTGAGATGGAAGAAATATTTGATTTCTAGAGTAACGGTGGCCATAGAATAAAAGCTAGTTCGTTTTTGGTAACAAGTCGATTACATATGT

TTACCCTGATTTTTAGTTTTTTACGTTAACAAAAAGACGTAGAAGCATATGTACGATGAACATTTATTTTAATTAAATTCATATATTTTGTAATGAAAAC

AGTAAGACGCAGAAGCATATACTAAAATACGTTGTAATATATAAATTTTTTACGCTGTCAATAAATTTTAGATGATCATATTATTAAAATTAAAATTTCA

CTATAACAAATTTAAAAATCTTATTTAAAATGTTAAATGTTTTCTGATTAGTTAATGGTATAAAAAATAACAATACATTAAAATTCATCTCAAAATAAAT

TGAAGAGCCCTCAATTATAATAGAAATACAAAGTATTGGTTCAGACCGTATAGTAAGTCAGTAGTATTTTATATTTTGGAAGTTGGAAATGGATTTTTCT

GAATATAGTATAAGCTTTGTCTGTCTGTATGTACTCATAATGCATCGGACCAATCAAGTGAACATAAACAAAAAACTAAAATAGACTACGTATTTTTTAT

ACTATTTAATTACAAAATTATTATCATAAACAAAAAGTTAAAATAGACTACGTATTTTTTATATAATTTAATTACAAAATTATTATAAAATTATCAATGT

ATAATAATTATCATAAAAGTTACATTTATGATAATTTTTTATTAGTTAACAATGTAAAATTATTTTACTAAACTCATTACATCATATTATAATTAATAAC

TTTGGTTTTTATTTATTTTGAGTTTTGGTTTTCATCTTTTTAACTTTTTACATTTTATTTATTTGTTATCTTTATCGTTGAGTTTTTATATTATTTTTAT

TTATAATTTTATATTTTAGTTCACAAATTTCATTTCTAATAAAACAGTTTACTAAGTAGAGAAGAAGAGCATACAACTTAAGCATGTGGGTTTAAACCTT

GCACAGCACTTGGCTCCTTTTCCATTCCTTGCTCCTTCCTTTTCTCAATGGGATCCTCTCTATTTTTTTTTTAAAAAAAATCCTAAAAATATGTATATTT

AGAAAAGAGATATGATTTGAACATTTTGGCTTTTTTGAATGAAATCAAAATTGGAAAATGTGGAAGAACCTTGTACTCGGTACACAAGGGTCTCGAACGT

ATTGCGTGATTTGAGTTTGAGTTTGACTTCAAATTGTGACCGTATCGTGTGTTTTGAGTTTGACTTAAAATAGGTGAACAAATAGTCTAATACATGTTAA

ATTAAAACCTAATACAACATATGATATTTTTGCAAGTTGCAATGTTGATCATTCTCACCCAGTCCGTGGGTTGATGGCGGTGATATGATACCATGATTTG

TTAAAAGTAGCAAGACAACCTAGACACGATTGTATACTGTCTTGATAAGTGTTTACTATATAAGCGTTTATCTATATATTGTTTGTAAAAAAAAAAAAAG

AAATTTGAGTTAAATTATTTTTAAACCAATTGCCAACTATTTTTATAAAATTCTTTTAAAAATTTATTGAAATAAGCTGAAAATCCTTTCAAGGTATCAT

AAAGTATATTCACATGTTTCTTCTATCTTTTGAATTGACTATAAAACTAACTAATGTTCATGACTTAGACAGACAATTGCATTTTAGATCCTGTAGTTAA

TAATCAACCAAGCTCTAGACATGGTTACTTACTTCGTGATAATGTTGATATTACATTTTAGTAATGAATTCTCGTATTCATAACAAATTGTCATTAGCAA

CCAAATGGATGTTGTGAGTTTAAAGAGAAGAGTTGGGGTTGTTATGAATTGTTAATGTTCATCCTCTTTTGCAACATAGTGTTGAACTTTGGCATGGTTT

GAAAACCAGGAAAGAGCAAAAATCATGATGAAATGGGCAGACTTAATTGATCAGAACATCGAGGAAATAGCAGCATTGGATGCCATTGATGCAGGGAAGT

TGTACCATTGGTGCAAGGCTGTTGACATTCCTGCTGCAGCAAATACTATTCGTTACTATGCCGGTGCTGCGGATAAAATTCATGGGGAGGTCTTAAAAGC

GTCTAGGGAGTTCCATGCATATACTTTGTTAGAACCAATTGGTGTTGTAGGACACATTATTCCGTGGAATTTCCCCAGCACCATGTTTGTTGCCAAGGTG

AGTCCTTCCTTGGCTGCTGGTTGCACAATGGTCCTCAAGCCTGCTGAACAAACACCTCTCTCGGCCTTGTTTTATGCTCATCTTGCTAAGCTGGTATGTG

TCTTCAAGAAATGGTTCATTTTCTTATCAAACATAAATTGACATCGACAAGTTAACCACTAAAAGGTGATTGATTTTCCTTGCTTTTATGTTTTGAAGGC

TGGAATTCCAGATGGAGTGCTTAATGTAGTACCCGGATTTGGCCAAACTGCAGGTGCTGCAATAAGCTCACACATGGACATTGATAAGGTAACTTAAATG

TCACGCATTTGGAAGTATGTTTGAGTCCTACATTGATTGAGTGGAATAAAAAGCTTAATGTGGTACTTAAGTTATGAGATTCTCTCATGTAATAGATTAG

TCTTTTGGGTTCAACTCTTCTTTTGTGCTTAAGTCAAAATAATTGGTGCTTTTGTTGAGAGTAGGGAGGAAAAGGCCTACTTATTTACAGGCTAAATTAA

GGTGCAATGTACTGAATACTGATAAGTGACCACCTAATAGACTAGTCATTTGGATTTGACTCTTCTTTGTGTTTTAGTCATAACAGATGACCAAAATTTA

AATATAGTAAGACCAATGTAATTGGCTGATTGTGAATTTGTGATTTGTGCATATGTAGGTAAGCTTTACGGGTTCAACAGAAGTGGGGCGTGAAGTAATG

CGTGCTGCAGCTAATAGTAATTTGAAACCAGTTTCACTTGAGCTAGGAGGCAAGTCACCCGTCATAGTTTTTGATGATGCTGATGTAGATAAAGCTGCTG

GACTTGCTCTCATGGGCATCCTATTTAATAAGGTGAAATTTCATTTCATGAAATCTAATTCCTTTTGACAAAGTAGTCTAATTGTGCATCTTCTAACATT

TAATTTTCTTGTTGATTTCAGGGAGAAATTTGTGTTGCAGGCTCCCGTGTGTTGGTTCAGGAAGGAATCTATGATGAATTTGAGAAGAAATTGGTGGAAA

AAGCAAATGCTTGGGTGGTTGGTGATCCTTTTGATCCTAAAGTTCAGCAAGGGCCTCAGGTATGAACTTGGTAGCACAATTATGTCTACATTTGTTTTCT

AGTAACCCTCTAACTATTGAAACACAAAATGAGAGTAGTAAAATATGAAAATCATTTTTCTCTATTGGCTAATTGTTTTCTTGTTGTAGGTTGACAAGAA

GCAATTTGAAAAGATTCTTTCCTATATCGAGCATGGAAAGAAAGAAGGTGCAACCCTTTTGACAGGGGGCAAAAGAGTGGGCAACAAGGGTTACTACATT

GAGCCTACAATTTTCTCTAATGTTAAGGTGAATTTTAACTCCACAAAAGTATGAAATAAGGTTCCATCAGAATAATTTTATAACTAACCATCGCATTGCC

TTTTGCATTTTATTCACAATATCTTGTTTAGGAGGACATGCTTATAGTACAAGATGAAATATTTGGCCCTGTGATGGCTTTGATGAAGTTTAAGTAAGTT

ATTACAAATCAATTTATTTATTCTAACTATTAGCTTAATTTGATGCTAGAAAACGTTGAGTTTGTTCATGAATATTGAATTCAAGAGTATTGTATAAATG

AACAACAGGACTATTGAAGATGCAATTAAGATTGCCAACAATACAAGGTATGGCCTAGCATCAGGCATTGTGACAAAGAGTTTGGACACAGCCAACACTG

TGTCAAGGTCCATTCGTGCAGGCATTGTTTGGATCAACTGTTATTTTGCCTTTGGGGATGACATTCCTTATGGGGGGTACAAGATGAGTGGATTTGGAAG

AGATTTTGGAATGGAAGCCCTACACAAGTATCTTCAAGTTAAATCTGTTGTAACTCCCATTTACAATTCTCCCTGGCTTTGATTTTTTTTATTTTTTTTT

GTGTTGTGTGGATATAAACAATGGTGAACTATAGAAGAGCTTCCTTAAACGTATACTTCATCAAATTAATAAGTGTTTAAATACATTTTTAGTCATTGTA

ATTTAATATTTTTTTATTTTTCATTTTTATAAAATTATTTTTTATTTTATTTTTAGGTCTTGT

>Glyma.08G039200 | Chr08:3067517..3072472 forward

AAACATTATATCCAAAAGATTCTCTGTATATTATACCTATGATGATTTAAGTAATAAATTTACTTATAAATTATAAAACTTACTTTATAAGTATTATAGA

CATGGGATTTTACATAATTGCCATCTAATGTCGATTAATACGTGAGCTCCTTGTTTGTTTTATCCTTTGATGATATTTTATCGCATATATAACAGTGGTG

CTAGCTTAGTCTGTTTGTATTACAATTAGTCTCAGTCCCCAACGACTGGTTCTGTTTTCTCTCTGCTGCTTCGATTCATTCACACAACCATGACTAGCCT

TACTAATGGCGATGCTGGCTCCCTCAACAAGGTCCCCACGATCAAGTTCACCAAGCTCTTCATTAATGGAGACTTCGTTGACTCTCTGTCAGGTTCATTT

TTCATACCTCTGCACTTTCTGTTGGTCTATCTTTTCTGCTTCTTCTAGCTTTCTATGCTCCTTTAATTTCTATTCCTTCTTCTGTATTTTTTTCCCCATT

GATGTACCTGGATCAAATGAAATACACATGTTATTGTGGGTGTGACATAATTGAGGTCTAACTTATTTTTAAAAAATGACATATAACTTTAATCTTAAGC

ATATGATTAGAAGTTTGATTTTAAGGTTGTTCTGTATGAAAAAATTTATCATAAAAGGTCAACTCTTTAAATTGATCTCAAGAATTAGTCTTTCACTTCT

TGTACTTGATTAAGCATTCAATTCTTATGTTCGTGTACATGAAAAAATATTTATATGAGACATCAAGTTAGATGAACAAGTATCTCGATAGATTAATATT

TGACTCTTAGTAGAAGGATAGCCTTGATTCAAATGAAAAGAAATGAAATACACTTACAAGAGACAATATATTACCTCTATTTCAGGTCACATGTAAAGTC

CTGAATTATGATTTAACTCTAAATAACGCGTATGTTTGGATTTTTGTCTGAACAAAAGTTTGTTTCTTGTAAGTTTGTGTTCAAACTATAATTTAGAGGT

TAATTATCTATACTTGTTATAAATGGTTGTGTCAACAGTACTACGTATGGTGTCTCAACTCTCAACTGAGTCTTATATATATATATATAACGAATTTGTG

TGTGTGTATATATATATATATATATATATACATACATACTAATATGACTTTGCTAAAACTCTTGTTGTTGTTGAAAAAACAGGAAAGACATTTGAGACAA

TAGATCCAAGAACAGGAGATGTTATAGCGAGGATAAGCGAGGGAGATAAAGAAGACATTGACATTGCTGTTAAAGCAGCACGTCATGCATTTGACAACGG

TCCATGGCCTCGCCTTCCCGGCTCTGTACGTTATTTTTTCCCTTCTCTTATTTAATTTATATGTACCAGGTGATTCATATATGATAATCATAAATATAAA

CAATAGATCTGTATTCCCCCTAATAAAAAAATAAAAATAAACCATAGATCATGTGTATAAATATAATTAAGATTACGGCGGAGTGCAGTAACTTCCCTTG

GCTGAATGATTTTCCAAAGATATGGGGAATGGATTGACTTTTTCGATCTTTTTATATTTAACTTTAATGAATTATTAACGTAAAAAAATTAACATTTGTA

TTTTAAATAGTATATTTTCACAATATTTCACAAAACTGATATGTTTTTTAATTGGTAATATGATTAAAATTATTAAATACCATGTTCGTGTTGATATATA

TAATTATATGTATTGTAAAAGAATTGACATTAAAGATACATAGAAAATTACGTAAATCTTTTAATAAATATTATTATTACTCAAGCATGCAAAACCCATG

TGATCCAGATTGTTATATGAATAATTAATATCAACGTTCTTATGTGATTTCTGATTATTTAATGCTAAACCCATGTGATCCAGATTTGTAATGCCGATTC

TATAAATTTCAATTTTATTTAAAACACGACTATGCATAAGTTACAAGTCACTCACAGTGAGTTGGATTTATGCATTTAAATATGACTTACATTTTTTACA

TTTAATTTTAATAGTGTAAAAAAATTAACACTTATTTTATCGTATATTGCCGTATTATTTCATAATCTTGATACAATTTATATTTGAAATATATATAAAT

TTGTCAAATAATATGTCGATGTCAGCTTATATGATACTGTGAGAAGAATCACATGAAAAATATATTGAAATTTAAATACATTTATTAGAATAAATATTAT

TACCCCTATGATGCAAGCGTGACTAGCTATGTCGGTTAGGTTTAAATGTTACCAAATGTAAACTTAAAAAACACACATAACGAGACATGAATTTTTCTTA

ACAAATTTTTTACGTTCAAACGGCCGTTTGATGATTTTAAATATTTGAAACACACAATTACAAACTGTTAATAATTGGTTGACCCAATGACCCCTCCCCT

AAAAATGGAAGAAGAAGAAGTAGAAGATGTTATAACGAATTTATATAGAAATTGTTGTATAAATATCATTTAGCAATGGAGTCATATTATGGCTTGAGGT

AACTAGTGATGGGTATATACTTAAGATAAATATAGTTGGGTATAATCAACAGCTGTGAAATATTGATAAATTAAGAATTTTGAGTTTTGTTTAATATGAT

CATGCTTATATTTTCGGGACTTTATCGATGTTTTGACTTAGTTATTCCAATAAATATTTTTTAATCAGCCACTGGTAATCTTGTATGATCAAGGTTTGTT

TGTAATAATCAGGAGAGAGCAAGAATATTGCTGAAATGGGCAGAAATAATTGAGGAAAACGCAGAAGAACTTGCAGCACTAGATGCGATTGATGCGGGGA

AGTTGTACCATATGTGTAGGAATGTGGAAGTTCCAGCAGCGGCAAACACTCTTCGTTACTATGCTGGTGCAGCCGATAAGATTCATGGAGAGGTGTTGAA

AATGTCTCGAGAGTTCCATGCCTATACATTACTTGAACCACTTGGTGTTGTGGGACACATTACTCCCTGGAATTTCCCCAATACCATGTTCTACATCAAA

GTCGCTCCTTCTTTAGCTGCTGGCTGCACCATGGTCCTCAAGCCTGCTGAGCAAACACCCCTCTCTGCTTTGTTTTCTGCTCATCTTGCTAAATTGGTAT

CTGTTTCCTCTACACAAGTTATTACTTGTGCACAAAAGCTTATTATTTATTGAGTTTTCCTTATTTTGAAGGCTGGAATCCCAGATGGAGTGATCAATGT

AGTGCCTGGATTTGGCCCAACTGCTGGTGCTGCATTAAGCTCACACATGGATGTTGATAAGGTAATTAAATTTACATGTCAAACATTTACAAGTGCCATG

ATCTTAAATAAGACTGGTTAAACGTTATATTAATCTATATATTTCATTATATGTTTCTTAACTTTGGTGGAATGTTCAGGTCAGCTTTACCGGTTCAACA

CAAACAGGCCGTGTGATAATGCAGGCTGCAGCAAAGAGTAACTTGAAACAAGTTTCACTTGAATTAGGAGGCAAGTCACCCCTCATAATTTTTGATGATG

CAGATATAGACAAAGCTACTGAGCTAGCTCTACTAGGCATCCTATATAACAAGGTGAATTTGCTTTCAGGAAATCCTTTACTTTCAAATAGCATATCCTG

TTAATTATGTCTCTGCTAACATTTAATTTCTTCCTATGTGACTATAGGGAGAAGTTTGTGTTGCAAGTTCCCGTGTGTTTGTTCAAGAAGGGATCTATGA

TGAATTTGAGAAAAAACTGGTGGAGAAGGCAAAAGCTTGGGTCGTTGGGGATCCCTTTGATCCTAAAGTTCAACAAGGCCCTCAGGTAAGAAAAATAGGT

ACTGCCACAATTATTTCTACACTAGTTCTCAACTATATATATATATATATATATATATATGTCGGTTATTTTCTTGTAACTCCATAATTTAATATTTCAA

AAATTGCTTAGAAGCTAGCTTTTTCAAACAGAAATGAAGAAATAATTAAGATCCTCGTCTATTGGCTAACAATTTTCTGTATATTGTAGGTTGACAAGGA

ACAATTTGAAAAAGTTCTTTCATATATTGAGCATGGAAAGAAAGAAGGAGCTACCCTTTTGACCGGGGGTAAAACAGTGGGGAACAAGGGCTACTTTATT

GAGCCGACAATTTTCTCCAATATAAGAGTAAATTCCAAGTGCATAAAGTTATTAATTAATCGGGTATCTTCAGCATAATTAGATAACTAATCATCATGTT

TCCTTTGTAAAATTTATTAACTACCCGTTTGTAGGAGGATATGCTTATAGCACAGGATGAAATATTTGGCCCAGTGATGGCACTGAAGAAGTTTAAGTAA

GTTTATTTTAATGTTATTGTGTCATGTTTCTTGTTAATTACCATGGTGAAATCCTCATATTTATTATGTTAACTATTATGATATATGAACAACAGGACCA

CTGAGGAAGCAATTAAGAGTGCTAACAATACCAAGTATGGCCTAGCAGCAGGCATAGTGACCAAGAATTTGGATACAGCAAACACAGTGTCAAGGTCCAT

TCGGGCAGGCACTATATGGATCAACTGCTATTTTGCCTTTGGCGATGATGTTCCCTTTGGAGGATATAAGATGAGTGGATTTGGAAAAGATCATGGATTG

GAAGCCCTTCACAAGTACCTACAAGTTAAATCTGTTGTTACTCCCCTTTACAATTCTCCCTGGCTTTGAATGTCTACTGCATGGATCTTGGAAAATGGTC

CATTCTGGCCATTTCCTATCAATTTGCATCTGCCATGAGGCATGGAAATTCAGTTCTTGAACAGTGGTGTCTATTATATTGTCAACAAGCTGGCCTTTTC

CTACTTTTCAGTCATGCTTTAATAGAAAATGTATGGAAGAAAAACAGTAATTTTATTTTTGTTTGGTTTGTAATTAAAAGGAAATATCAACACAATTTAA

CTTATTGCTTCAACAGAATATATAAATTGCTATAAATCTATATCTATTTTTTATTC

>Glyma.08G039300 | Chr08:3073843..3078936 forward

TTGCCTTTGCGAGAGCGCTGATTTCTCCACCTAAAGTCCTAAAGTTGCGCCAATTCATGTTCTTATCTTTGCATTGCATATATATATATGTTCTTCGTAA

TTAATCATATTGCAACATCCCGCGTACCGCCAGCGTCTCTACGACAATCTCCTTTCTCTCTAACTCATAACTCAGATGAGTTCCCTCTCTAATAACTCTA

GTAGTAGCCACGGCAATTCCTTCCTCCAGATGCCCCCCATCAAGTTTACCAAGCTCTTCATCAATGGAGATTTCGTTGATTCCTTATCTGGTACTTGAAT

ATTAAGACAAATTTAAATGTAATATTACAAGTATTGTTAATGCTATTTTTAATTTAAATTAGCCTTTTTTAATTATTACTGTCAATTTTCAATTATAAAA

AAATTGTCAATTTTGACAAATAAAAAAAAAAAAACAAGAATGACAGTAACTATGAAACGTTGATGCGGGTTTGCAGTAATTAAGGATCTGAAACTAACTT

TTATCTTCTCTTTAGTGTGAATTTGAATCCTCTTTATGATCACTTTTTTGTTTGATAGATAATTCTCCTTGAAGAGATCTTCTCACTTCTAAGGGATAAT

TTGAGTAGTTATAATTAAGTAAAATTACTTGTGATTTAGGTTTAGGTAGCAATAATCATCTAGGAGTTTAAAAATATAGTGTATTGTTAATTATTTAGAT

TTGAAATATTTTTAAAAATTATAATTTATTTTGATTTCATCTAAATACAAAATAGGCGGATATATTTCTAACAAAATTGTTTGTGATTATTTATATTGAA

ATAAAAAAGGAAGGACATTTGAGACTATAGACCCCAGAACAGAAGAGGTAATTGCAAGAGTTAGTGAGGGAGATAAAGAAGATATTGATATTGCTGTTAA

AGCAGCACGTCAGGCATTTGACTCAGGTCCATGGCCTCGCTTGCCCGCCTCTGTACGTTATTCTTTTTCCTTTCTTTAATTAAATATTTTTTTATTCTTC

CTTTCTCCAACTAAATCATCCCCAGTTTCTTACATTATAAACTTCAACATGTATTTTTTAATTTAACTAAATAATGAAAATTGTGATAATATATGTTTAC

TCAAAACTTTCGAAAGGCCATACTTTTATACATACCATTATCAATGACCTATTCGGCAATTCGATCTAATCGAGGCCAAGCAGTCCAATGTTGAACAAGA

TACTTATGAACTCATGTTTGACTTAGACGGAGTGTATATTGAGGAACAACAAACCAAACATCATTTGGTAGTCTAAGACTATTAACTGTTTGCCGGCTAG

ACATATATTTAATTTTTTGTATTTTACCATAAAAGCTAATAACACTTAATTATATGCATATAATCATGCATAATAACTGAGTAGATACAATGTGATATAA

GACTACCTAATAATTTCTCAAAATAAACATATGTTTTGAGTATGCTTTGGTGGGAAAAACTTCTAGTTAGCTCCTAATTAGAAGAAGCATGTTGAGTATC

TTAATTGAAATTTATATTTTAATTGTGCGATCATAAGTCTGACTTTTTTTTACAGCAAACATACGACTGACATTAAGCGGGCAATTAAGTGGAAAAATTC

AATTGTAAAGTAAATATTGGAATGACAAATGTGAATGGAAAAATTTAATATTATTTAAGAATGAAAAAATTAAACAACTTATAAATAAATAAATAAAGGC

TAAAATAAACTCACGTGTGTGGTTTATGTTTATTGATGAAGATTTCTATGATATTTTATTCTTTTATATTTAGTAATAGAATAAATTTATTGTACTTTTG

TGATTATTTATAATTTAATTGTTTATTTTCTTGTTTGAATTTTTCCTTCTATCACTTCTAATCCTCTTTAAAAATCTTAATTAATATTGACATCGTATGC

TATACCAGGAAAGGGCAAAAATTATGATGAAATGGGCAGACCTAATTGATGAAAACATAGAAGAACTAGCAGCATTAGATACCGTTGATGCTGGCAAGCT

GAACTATATTAATAAGGTTGTGGAAATTCCTTCAGCTACAAATGCGTTACGATACTATGCAGGTGCGGCTGATAAAATTCATGGTGAAGTATTAAAAATG

AATGGGGATTTTCATGCATATACACTTTTGGAACCAATTGGTGTTGTGGGACACATAATTCCATGGAATGCCCCCAGCCTCTCGTTTTTCATCAAGGTTA

GCCCTTCCTTAGCTGCAGGCTGCACCATGGTCCTCAAACCTGCTGAACAAACACCCCTCTCTGCCTTGTTTTATGCTCATCTAGCTAAGTTGGTATGTAT

CAACACATAGTTTCATTTCCTTATCAAACATATTTAGGCTAATTGTGTTGGGTTTGTCCCTTTTAAAGGCTGGAATCCCAGATGGTGTGCTTAATATAGT

ACCTGGATTTGGCCCAACCGCTGGTGCAGCAATAAGCTCACACATGGACATAGATGTGGTAATAAGAAGTTACATGTCAAATATTTACAAGTACTTACTA

TTCTCTTAATTGACTTGACTAATAAAATTTCAATTTCCTTGATTGTGTTGTGTATCTTCAGGTCAGTTTTACTGGTTCAATTGAAGTAGGGCGTGAAGTG

ATGCAGGCTGCAGCTAGGAGCAATTTAAAACCAGTTTCACTTGAATTAGGAGGCAAGTCTCCTCTCATTATTTTCAATGACGCGGATATAGACAAAGCTG

CCCAGCTTGCTCTCTTTGGCATCATGTCTAACAAGGTGAAATTCCTTCCTTTCAAGGGACATCTTGTAATATATATATATATATATATATATATATATAT

ATATATAGTATAACAATATAACAAGCATGCCAAACCAACTGAATATTCTTCTTATTCTTATTGGAAAAATGTGAACTACATATGTAAATTATTAAATCTT

ATTAATCAAAATTAAAAGAAAAATATAAATAATTTAATGTGCACAGTAAAATAATTTTATACTTCAATCATAAATCATTATTTAAATTATTTTAAGATAA

TTATCTTAAAACTTAACACATTTATATATATGATGAGTTATAATTGAATAATTGTGTAAAAGAATTTATATTATCAATGTGTAATCTTTTTTCTTAAATA

ATATTAAATGAGTTTTCAAGTGGTTTCCGGATATTAACTAACTTTTTATTTTCATCATCAATATGTGGTTGAATGAGCCATGTTTATAGTTGTAGCCAAT

AAATTATGTACTTCCTGTTTGTGCTTTCAGGGAGAAATTTGTGTGGCAAGTTCTCGTGTGTTTGTCCAGGAAGAGATCTATGATGAATTTGAGAAGAAGT

TGGTGGAGAAGGCAAAATCTTGGGTCGTTGGGGATCCTTTTGATCCCAAATCCCTGCAAGGGCCTCAGGTAGGGACAATATATATGATAGCATGATTTTG

TTTACACTCATTCTAAAATATATTTGGTCCGTCCTTTTCTTTTTTTACTAGTAACTCGCTAAATAGGAAAATGAATTTTAGTAATTAGTACAAATTTATT

TGGTCAAATATTATGAAATGACATTGTGTTATTTTTTTTTATAGATTTCCCATATATATTTTTAGGGGTGTTTGTTTTATGTCATCTTCTTTTATTTCTA

AGAATGTGAGAAGAGAATAGAAATGAATATTCTCATATTTATTAGAAGATTTATTAGAAGGTTATGATAATATATTTTATTTTTAGGAAAATTATGTTCT

CACGTTGTAAGTAGATTGTATTCCCATGAGTAGGTGGTGGGTATCTATATTTTCATTCGAACTAACTTTTAACTTTTTCTTTCACTTTTAGGTTTTTAAA

AATTTTAATTATCTAAAATTTAGCTGTTTTTTAAAATTACTCAATATTAACCAAACATATTCATGATCGAGTATAATATAACATTCCTAGATATATTCTT

GAAAATATGATTCCTGAAAATATAAAATTATACGGCGAAGCAAACGTTCCTTGATCATCATCCAGTGTATTTTGTCTCGTTTGGTCATGGTTGCAAATTC

TTGAACTCTATTTTTGTACCATATTCTTCGTCTGAGATAACACAATACTCCAAGTATATAGTTCAAACATTTGAATAAATATACGTTTACTTTCTCTTTT

ATCCCATCTTCACTATATTTTTTGGTGAAATTTATCATAACTTGTTTTCTATTATAGGCTGACAGGAACCAATTGGAGAAAATACTCTCCTATATTGAAC

ACGGAAAGAGAGAAGGAGCTACCCTTTTGACCGGAGGTAATACAGTGGGCAACAAAGGTTACTACATAGAACCTACAATTTTCTGTAATGTAAAGGTAAA

TTTTAATTTCATCAAAGTCTCAACCTCGGTATCTTCAGCATAGTTTGATAACCTCTAAAAAAAAGCATAGTTTGGTAACTAACCATCGTGCTTTTATTTT

GCTTATGAAAACAAAAAACCTGTTTAGGAGGACATGCTTATAGCACGAGATGAAATATTTGGCCCTGTACTAGCGCTGATGAAGTTTAAGTAAGTTATAG

TTTAATTTAATGATTTTTTTTATTGATGTTTAGTACCCATAAAAAAAGTGTATGCGGGTTTGTTCATGAACGTTGAAATTAAGTATGATATATGGTATGA

ACAGGACCATGGAGGAAGCAATTAAAAGTGCTAACAATACCAAGTATGGCCTAGCAGCAGGAATTGTGACCAAGAATTTGGATACTGCAAACACTATGTC

AAGGTCCATTCGTGCAGGCATTGTTTGGATCAACTGCTATTTAACCGTAGGGAGTGACGTTCCTTTTGGAGGGTATAAGATGAGTGGATTTGGAAGAGAT

TTGGGATTGCAGGCCCTTCATAAGTACTTACAAGTTAAATCTGTTGTAACACCTATTCACAATTCTCCTTGGCTTTGAATAATTGAATGTCTCCTACATG

AGCACATATGCGTGTCTTCTCTCATTTGAAATAAATTACACTTTATTTCCTTATGATGTATGACTTAAAAATACTTAGTCTCTTGTATTATGAGTTCTTT

GTTTTATTACAACGTTGTTAACTTAGTATAGTTTTTAGAGAGAAACAACAAATTATAACTATTACATTTTTTCTTCCCATTGACGTGAAAATGT

>Glyma.09G189200 | Chr09:41396279..41400810 reverse

ATGAACTCCAATGGTTACCCTGCTTCCTCCTTCAAAATCCCCACCGTCAAGTTCACCAAGCTCTTCATCAATGGACATTTTGTTGATTCACTTTCAGGTT

CATTACCTTTACTTATCGTTTCTCTTTCTACTTTCTTTTTTTTCCCCTTTATTGCTCTTCACTTCAGCTTCTTTCTTTATTTCTTTCTGGGTACGTTACA

CTGCTCCCTCATTCCTTGCATTTTCCTCCTCTGTTTTTCATATTAAAGTGAAATACTAATACACTAGATGAAACATCACTCCTCAACTCTATTTTGTTGA

GGAGGAAAATTTATGTTGTATAGTTTAAAGAGTATGTTTGACCCAGTTTATTTTTTTTTAAGGAAGAAGCTTGACCAAACAAATTTTAAAATAATTCTTA

TAAAAATTAAGTAATTAGAAGAACAGAACCAATAAAATAATAATATTGGATTCGATTTACTTCGGTAAAATTTAAGTTTCGAGTTTTGAGGATGAAAAAA

ATTTAATTAAACCAAAAAATCCACTAAAAGTTAGAAAAACAGAAACAATAATTAAAGAACTTTTAAAAAAGCTATCTTGAGGAGCTTTTTATTTACTTAA

TTTATATGGAGCTTTTGTTTTTCAAAAACCACACTTCTATACCTGGGCTGGCACAAGAATCCCCTTATCAATTCTAAGAATTACAATCTGATTATTTATT

GCAAGTTCTTTTATGACAATGGACCTGCTAGTATATATAATAACTAACAATTAATATTTTCATTTTAATAAAAAAATAACTTCATTCATATTTTTTTCCA

TCAACATCAATGCTTTTAAACATTTATATATTTTGCAAATAATATTTTCAAATTAAAAAGTTTAAATATTTGTATTTTTATCATTATTAAAATAACTTTA

TATACATTTTTTTTTCGTTGGACGAGATAATTATTTTCAAACTTGAAAGGAGCTTTTAATCTAAAGAAAATGAAAATTATTAAATAATTTCTAGTTATTT

TCTTAAGCTAGACCAAATAACCTAGTAAAGATTAAAGGAAAATAGTAAACTGATTTATCATTGATTAGATTTTAGGATGAGATTTGCCTTAACAATTATT

CTCAAACATGCCAACTATGCACACTAATAATTTGTATTTAATTTTTATTTTTTAACTTAAAAAAACTTCAAAATTAATCATGACATGGTTGTCTGTATTA

GTTCACTTATGCTGTGTTTTTTTTTTATTGTACATACTTACGCTGTTTTGTAATGGTGAAAAAAAATTAAGAAAAAGAAAAAAAAAATTGAATGAAAGAA

GAACTAGCATAAAAGAAAAATTAAATGTTACTTGTGGACTTAACTTTTTTCTATTTCCTTTCTTTATATATATATATGGGTGTGAAAAATGTTATACAGA

GACTTATGTAGTTATGTAAGGAGGTGTGGCGTGTGCTTGTAAGCTACTAGCTTTTCATTTGTTTTTCTTCTTTTTTTTTCCTTTAAAAGAATATAGCCAA

TTGACATTAACAAATATCAACCACTAAAGTTGTTTTTGTTTAGATAAATGAATAACAAATCATTTACATTGTTTGTGCCTAACTTTGTTTTTATTTCTTA

AAAAAATATGGTAAAACAGGAGGAGAATTTGAGACAATAGACCCAAGAACCGGAGAGGTGATTGCAAGGATTGCAGAGGGAACCAAAGAAGACATTGACC

TTGCCGTCAAAGCGTCTCGTCTGGCTTTCGACCATGGTCCATGGCCTCGTATGCCTGCCGTAGTACGTTATCATTTACTACCTCCGGTTCTCTTTTTACA

AGAAACAAGTTTTAATGTAAAATTTACAAGGGAGTATTACTTTTTAATACTTATATTCTATTAAATGATAAGATATAAATAGAGTAAGAAAATTATACCA

TTATTATTATTATTACAGATAATACTTATATTCTATTAAATGATAAGATATAATTTTTTTTCCGATTACAAAAAAGGAGCTCTATATTACTTTTTCCACT

TATTCACTCTTCTACTATTGCTTGTAACATGTTAATTAAACTTGACATGATTGTCCAATCAGGAAAGAGCAAGAATTATGATGAAATGGGCTGACCTAAT

TGACCAACACGTAGAAGAAATTGCAGCACTGGATGCCATTGATGCTGGAAAGTTGTACCATATGCTTAAGGCTATTGAAATTCCTGCAACAGCAAATACT

ATACGTTATTATGCTGGTGCTGCTGATAAAATTCATGGAGAGGTATTAAAGCCGGCACGGGAGTTCCATGCATATACTTTACTTGAACCAGTTGGTGTTG

TGGGACATATCATTCCTTGGAATTTCCCTAGCATCATGTTTGTCTCCAAGGTTAGCCCTTGCTTGGCTGCTGGTTGCACAATGGTCCTCAAGCCTGCTGA

ACAAACACCTCTTTCAGCTTTGTTTTATGCTCATCTAGCTAAACTGGTATGTATTCAACAAGTAGGAGTCTTAACAAAACACTTCCTAACATACTCCTTC

CAATGTCTTCTTTATTATTGGTTATAATTTAGAGTTTAATTTTTATGCATTGCCAGTATACAAAAAATTAAATTAAGAACAAATCAGAAATCATTAGTAG

ATGATTTTTAAGATTATTATTATAAAACACGATAGTTTTTTTTAATAAGTTCTTATGTGACGATTTGTAATTAGATGTATAACAGTGTACACACACTGCT

TTCTCTAAGATTGACCGGGAACTATAAGATCATAGCTGAGAGCTTTTTTTGGACTGAAATCATAGCTGAAACTTATTAAACAAAAAGCATCAACAACACA

ATTTTGTGATTTTTGATATATTTTAGTTAATAATAAAGATTGTTTAAAAAAAAAAAAAGTTAGAAAGTTTATTACTATCATATTTATTCAATATATCAGC

AAAAAATATCATTTTGATACAACAAATGGTTCATTTCCTTATAAAACATAATTGGCATTTACAAGTTAATTGCTAGAGGCTAAGCTGATTGTTGATTTTC

CTTGGTTATATGTTTTGAAGGCTGGAATTCCAGACGGAGTGCTCAATGTAGTCCCCGGATTTGGCGCAACTGCAGGTGCTGCAATATGCTCAGACATGGA

CATTGATAAGGTAACTTATATGTCAAGCATTTGGAAGTACCATGATTTAAATATAAATAAAGTAAGACTAATACAATTGGTTTGTTTCTCAATTGTGCAT

GTGTAGGTCAGTTTTACAGGTTCAACAGAAGTAGGGCGTGAAGTAATGCGTGCTGCAGCTAACAGTAATTTGAAACCAGTTTCACTTGAACTAGGAGGCA

AGTCACCATTCATAATTTTTGATGATGCAGATCTAGATAAAGCGGTTGAGCTTGCTCTCATGGCCGTAGTATATAACAAGGTGAAGTTTCTTTCAATAAT

CATATTCCTTTTTACATAGCAACAACACATCTTCATTAGTGACAACTATTTATTGTTGTTGTCTGGTTTTCAGGGAGAAGTTTGTGCTGCAGGCTCCCGC

GTATTTGTTCAGGAAGGGATCTATGATGAATTTGAGAAGAGGTTGGTGGAGAAGGCAAAAGCTTGGGTTGTTGGGGATCCCTTTGATCCTAATGTTCAGC

AAGGGCCTCAGGTATAAACTAGCACAACCCCATTATGTTTACTCTTGTTCTCCTACAACTTTATTTCAAGCATTGCCTTAGTAAAAAACAGAGAAATGAC

CCAAACAAGAAAAGTTAATTACTAATTAGGATCCTCCTCTAGTTACTTACATTACTACTTGCTATGGTAGGTTGACAAAAAGCAATTTGAAAAAATTCTT

TCCTACATCGAGCATGGAAAAAGAGAAGGGGCCACCCTTTTGACAGGGGGCAAAAGAGTGGGTAACAAGGGTTACTACATTGAGCCAACAATTTTCTCTA

ATGTTAAGGTGAATTCTAACTCCTGTGAAATTTTCTGCAAATCTGCATGAAATTCATGCAGAAAAATTTCACACAATTCATGAATTTATTGTATTGTACA

AAAATACAAAGTAGGGTTTCATTAAGAAGATTAAATAACTGAGTAACTAACCATCGTATTGTACTTTTACTTTTTATTTTTAACATGTATAGGAGGACAT

GCTTATAGCACAAGATGAAATATTCGGTCCCGTGATAGCACTGATGAAGTTTAAGTAAGTGAATACTTTTTATTTTTTTATTTTTTGTAGTTCCAGTATA

AAAAATATGTGCAATTGTTCATGAATATGAAATTTAAGTATATATGATATATGAACAGGACAATTGAGGAAGCAATTAAAAGTGCTAACAACAGCAGATA

TGGCCTAGTAGCAGGTGTTGTGACCAAGAGCTTGGACACAGCAAACACAATGTCAAGGTCAATTCGTGCAGGGGTTGTTTGGATCAACTGTTATTTTGCC

TTCGAAAATGACATTCCTTATGGAGGATGTAAGATGAGTGGATTTGGAAAAGATTCTGGATTGGAAGCCCTACACAAGTACCTACATGTTAAATCCGTTG

TAACACCCATTTACAATTCTCCTTGGCTTTGA

>Glyma.09G189300 | Chr09:41407454..41415106 reverse

GTACCTTTTCTCTGTCGACTGGTTGTTCTTGTCTCGGAGTTAAGTGTGCGTAACTCTGTATATATAAAAGAGGTCCCATTCTCACTTTTTCGAAAAACAC

GTTTGCCTTTGATCTTCTTGTCTCTACTGCTTTTATTTGCAGACACCCAATTAACGATGGCTGCTCTCTCCAACGGCCACGGCTCTTCCTTCTTCAAGAT

GCCCCCAATCAAGTTCACAAAGCTCTTCATCAATGGGGAATTTGTTGATTCCCTTTCAGGTTTTTAATTAATATACTTATGCCTCTTCTCTCCTTTAATT

AATATTCTTTTATTGCTCTTCATCTCACTTTTTCTTTTTATGTGGAAAAAGTTATTCCTACGCTGTATTTTTTGGTGCATAAGTGAAAAAAAAAAAGTTA

CAAGTATGACCAATGATATATAATGAAACATATAGAGACAGGGAAAATGGCATAAAGTGAAGGACTAAATTTAGATGTATGTTATTTTATGAAAAATCTA

GTTGATTACTCGGGGCGGGTCTCCCATCTTGTACTGTTCTGTATTTCAAATCAGAATTCCATCTCAATAATACATGCTCATGTTTAATGTAGATTTTTAT

TATACATGTTATGAGATAAAACTTCAATTTTGATTAGACAATAACACTAATAACACTTAAGAAATCGCATTTAAATTTTGTCTTCAAGTGAAACGGAAGA

AAATTTGGATGTATAATGATAAGATTCTTTCTCTGCTAATCCTATTCATCCTTCTCTGTTTTGCTCTCTCTCTTTTTTAGACTCAATATCTTTCGTGTAA

AAATGAAATAATTATGAAGGTCTTTGTTAATCAACTAGAGCATTGGTTAAAAAATTAAAAAATATATATTTATTATGAACAAATTTAATGTATTTTAATA

TTTTTTTTAATTTCTTAACCAACATCATACATACTTAAGCTACAAATCGAAAGATAGATGATATGGAAATTCCTTGTAGAGGTTAGGTTTATTCAATGGT

TTGTGTAAAGCTGTGAATAGATAAGAGTTTCCCACTTGGTCCAGCCTTTGCTCTCAACCATGCAACATACTGTAATATTCATGAAGAAAGGGAGTTTCAT

TAAATAATTTTTATTTTAATTCCGAAAATATAAAAACAAAAGGTCAATAAGTAGTTCCTACTTTAATTTTGATAATGTATACTTATAACAATAATGAATA

TGTTTATCTCGAGGTTTGAAAATCTAATCTATTTAAATAAAAATTTATGAATGTTATAATTTCATGCTTATTATGAGTTTTAAAAATTATTTATAGGTAT

TGACTATGTTATAAATGTAATGTTAACTTTTTTTGGTATACATTAATAAAAATAAAGTTAGAATTTAAGACCTCGAATACTATTCGAATTCTCTACAATT

AGATGAATCCTAGTGAATCATTGATGTACAAATTTAAGATTTCACAACAATTATGTATCAATATCTTTGACCGTAATGTCAACTTTGATGGGTGGGTCTA

TTTTTATTCTCGTTTTAAAAGGTGCTAAAGTTAAATTTAATAATGAAAGCATACGTAACATTATATCTTGAAAGCACCCAAAGGTGGAAAATGCACCAGC

ACAGGTATCAATTGGAAAGGCTGATATATTAAGTTATCAACCAAATATTCAAGTGATGAATTTTTTTTTCTTTAAATAAATTGTGAAAACCTTCCTGAGT

TTTTGTTCTCTTTTTATTTTTAATTTTTTTACTTACCTTTTTTAATCTAGTATTTGTTTAATTTTTGTGTATAAACATGGAAAAAACAGGGAGGGAGTTT

GAAACAAGAGACCCTAGGACAGGGGAGGTGATTACAAGAATCGCAGAGGGAGCAAAAGAAGACGTTGATGTTGCTGTCAAAGCGGCACGTGCGGCTTTCG

ACTATGGTCCATGGCCCCGTATGCCCGGCGCTGTACGTTATCATTTCTCCATTTCTTTCTCTATAACTGTTGTGTTGTGAGTTATGACAATTAATTTGTG

GGTCTCACTAATTAATCTCACTACATTTTAGGTAAGGAATCAAAGTACGTAAGAAAATTTATTAAAAAAAAAAATACGTCAAGATAGTACTCCCTCGTGT

CCTTTTTTTTTTGTAGTGTAAAAAAAGAAACTCGCTCGTGTTCTAATTATAAGGCCTAAATTTCCATAAAAAAAAGGTTTAAATTAAAACTGTAATTTGT

TTTTTTTTATTATAAAAAATTTAGGTGAGTTTGATTTTGAGAGCTGAGATTTATTTGGTCTCTTTTTAATAAAGTACGATCTAAGATATCTCAACATTAT

TTTTTATTACAAAATATCCTTCATTGACGTTAATTTTGAAATGATACGCCAATGTGACTGTGATAGAAACATAATTCATTTGGTTAGTTTTTTCACAAGA

ATAGTTTTGAGAAGACCAATTAAGATAAATTTAATGCTCTTAATTAAAACAATTAAATTTTTAATCTTGATAAATTAATTAATTAGACTTTATAATAAAT

ATCGGATAAAGTATTACTAAAAAATATAGTCACTACTATAATAAATAAAAAATATGAATTATTTTCAATATAGAAAAAAATGATCATATATAAAAATTCT

TAAGTTTTAATATCTTAACGAATTATCTTTGACCTGTTTTCTGAATTAGATTTTCGAACTGGATTCTTAAAGATGCTGGTTAACTTCATCTTGCTTCCTT

TCAGAAGAAAAAATAACTTAATCTTGCCACTCAGTATCATTTATTGATTTGACTTCTTTTCCCCAATAAACTTGTCATCAATCTTATCGTATTCATTAAT

GTATTGATATTTTCTTCCCACTTGTTTGATAGCTGATGCTAGAGCCAGCTTCGATCCTAGCATGATTCACAGGTCATCGCGTCCCTGTGAGTCTGTGACT

TTGATTGACACGTATGAGTGTGTTTGAGTTTTGAGAATGCGTTCAAATCATATTATTAAACGGAAAAAATGATTTTCTAATGCAAAAATTAAAGTGTTAA

TTTTGCGTTCTGTCAAAGAAAATGCAGACACAAACACCCACACACTTATATATATATTGTTTGTATTTATTAATTTTTTTTAACTAAATGTTGGCATGCA

TTGCATGGTTCATATTTGGGTTCGAGGGTAACGAAATAAAAGCTAGTACGTGTTTGGTAACAAGTCGATTAGCCTGATTTTTACGTTAATTTTAACAAAA

AGACGCAGAAGCATATATATGATAAATAAATTTAATTAAAATACATTGCAATATATATATATACATTGTCACATTACTAAAATTAAAATTTTACTATAAC

AACTTTAAAAATCCCATGTAAACCGTTGTCTGATTAGTTAATAGTATAAAAATAGCAATATATTAAAATTCATCTTGAAGAACCCTAAGCGGTTAATTAA

TCAGTGCAAATGATTGATTGTTTATTAATTATTTTTTTTATTTAAAAATTATTTAATTGTATTTTAGGATATTATTTAATTGCTAATTGATTGATTTTGA

GATTTCTTAATTCTATTAAATAATCATTCAAATAGATAATATTGGTCAGTTTATTTAAACTTATTTATTAAAAATTATAAAGATTTGTAATTAAAAAAAA

AACAAAACCATACGACCTAAGCGTTTTTTTTTCTTCCATTTATTAGTTAATCTCAGCTGACCAAGAAATTGAAAATTAAAACAATACCGGACCGCGGGAA

CCTCGGCCTTGTTTGGTTGTTGAGGAAAAAAAATGTAAATGTATTAAATTTTTTTAAAAAATTGACATGGATCTCACACTTTCATTCATACATTACTTAA

CTTAAAAAAAATCTCAATTTCATTTTAAAATATTTAAAATTTTAGCAAATTGTTTTATTTCAATATAGTTGTTATTTTAAAAATTATGAAAGTTATTTCT

GTTTACTTTTATCTTTTTAATAACTTTCTCGAATAAAATAGAGTATTAATTAATTTATAGTTTTCTGAATAGTTTATTAAAAAAATAGTTAATGAGAGTC

GCATTTTTTAACTTTAATTACTTTATAATTTTTCTTGAATTATATATGTATTTAAAATTGCTTATAAAAAAGATTATTAAAAATCAATATAAGAAATATT

CAGGCTAGTTGAATAGTAAATTTTATACTTTGTGAATCCTGTTAAAAATCACCTTATCAAATAGCACAAGGGGAAAAAGGGAGGGGACGGTAAAGCATTA

AACTATCTCTTACACGATGAATTTAAGTCTGTTAGAAATTTAAGATGTGTGAAAAAAACTAACAGTTCACATAAGTATTTGTGTATTAAGTTTTGTATGT

CTTTTACCGAAGTATGTAAAAACTTAGTGATAAAGATATAAAACAGTTTAATAAGTAGTAGAGTAGAAGAACTTGCAACTTAAGCATGTTGCTTTAAACC

GTGCACGGCACTTAGCTCCTTTTCCATTCTTCTCTCCTTCCTTTTATTAATGAGATCCTATCTAATTTTTGTTTTTTCTTTAAAAAATCATAAAAATATG

TATATCTAGAAAAGGGATATGATTTGAATATTTAGCCTTTATGAATTACATCAAAATTGGAAAATGTGGAAGAACCTTGTACTCGGTTATACACAAGAGT

CTCGAACGTATCGTGTGTTTTGAGTTTGACTTAACATGTGTGAACAAATAGTCTAATAGTACTTGTTAAATTAAAATCCAATACAACATAAATGTTTGGC

TAACTCCCACCATGTATACTGTCTAGATACTTTACTATATATATAAGCGTTTATCTATAAATTGTTTGTAAAAAAGAAATTGAGTTAAATTATTTTTAAA

CTAATTGCAAACTATTTTTATAATATTTTAAAAAAAATTATTGAAATAAGCTGAAAATTATTTTAAGGTACTATAAAGTATATTCACAAGTTTCTATCTT

TTGAAAAACCAATGTTCATGACTTAGTCAGATAATTGCATTTCAGATCCTGTAATTAATAACAAAGCTCTCGACCAAGCTCTAGACATGGTTACTTACTA

CGTGATAATGTTGATATTAAATGTAGTAATGAATTCTCGCATAAAAAATAAATTGTCATTAGCAACCAAATGGATATTTTGTGTTTAAAGGGGAGGGTTG

TGGATGTTATGACTTGTTAATGTTCATCCTCTTTTGCAACATAGTATCAAACTTTGACATGGTTTGAAAATCAGGAAAGAGCAAAAATTATGATGAAATG

GGCAGACTTAGTTGATCAGAACATTGAGGAAATAGCAGCATTGGATGCCATTGATGCTGGAAAGTTGTACCATTGGTGCAAGGCTGTTGATATTCCTGCT

GCAGCAAGTACTATCCGTTACTATGCAGGTGCTGCAGATAAAATTCATGGAGAGGTCTTAAAAGCTTCTAGGGAGTTCCATGCATATACTTTGCTAGAAC

CAATTGGTGTTGTGGGACACATTATTCCTTGGAACTTCCCTAGCACCATGTTTGTTGCGAAGGTTAGTCCTTCCTTGGCTGCTGGTTGCACAATGGTCCT

CAAGCCTGCTGAACAAACACCTCTCTCGGCCTTGTTTTATGCTCATCTTGCTAAGCTGGTATGTGTCTTCAACAAATGCTTCATTTTCTTATCAAATGTA

ATTGACATTGGCAAGTTATCTACTAAAGTCTAAGTTGATTGATGTTCCTTGCTTTTATGTTTTGAAGGCTGGAATTCCGGATGGAGTGCTTAATGTGGTA

CCTGGATTTGGCCAAACTGCAGGTGTTGCAATAAGCTTACACATGGACATTGATAAGGTAACTCAAATGTCAAGCATTTGGAAATTGGAAGTATGTTATG

AGTCCTACATTGATTGAATGGAATAAAAAACTTAATTTGGTACTTAAGTTATTAGTCTTTTGGGTTGAACTCTCTTTTTGTGCTTAAGTCAGAATAATTG

GTGCTTTTGTTGAGAGAGTTGGGACGAAAAGGCCTACTTATATACAGGCTAAATTAAGGTGCAATGTACTGAATTCTGATAAATGACCACCTAATGGACT

AGTCATTTTGGATTTATCTCTTCTTTGTGTTTAGGTCATAACAAAGTGCCATAACCAATGTAATTGGCTGATTGTGAATTTTTTATTTGTGCATATGTAG

GTAAGCTTTACGGGTTCGACAGAAGTGGGGCGTGAAGTAATGCGTGCTGCAGCTAATAGTAATTTGAAACCAGTTTCACTTGAACTAGGAGGCAAGTCAC

CCGTCATAGTTTTTGATGATGCTGATGTAGATAAAGCTGCTGAACTTGCTCTCCTGGGCATCCTATTTAATAAGGTGAAATTTCTTTCGTGAAATATGAT

TCCTTTTGACAAAGTAGTCCAATTGTGCATATTGCAACATTTAATTTTCTTGTTCATTTCAGGGAGAAATTTGTGTTGCGGGCTCCCGTGTGTTGGTTCA

AGAAGGAATCTATGATGAATTTGAGAAGAAATTGGTGGAGAAAGCAAAAGCTTGGGTGGTTGGTGATCCTTTTGATCCTAAAGTTCAACAAGGGCCTCAG

GTATGAACTTAGTAGCACAATCATGTCTACATTTGTTTTCTCTAGTGACCCTCTAACTATTGAAACAGAAATGAGAGTAGTAAAAATATGAAAACCATTT

TTCTCTATCGGCTAATTATCTTCATGTTGTAGGTTGACAAGAAGCAATTTGAAAAGATTCTTTCCTATATCGAACAAGGAAAGAAAGAAGGGGCAACTCT

TTTGACAGGGGGCAAAAGAGTGGGCAACAAGGGTTACTACATTGAGCCTACAATTTTCTCTAATGTTAAGGTGAATTTTAACGCCACAAAAGTATGAAAT

AAGGTTCCTGCAGAATAATTGGATAACTAACCATCCCATTGTCTTTTGCTTTTTATTCACAATATCTTGTTTAGGAGGACATGCTCATAGTACAAGATGA

AATATTTGGCCCTGTGATGGCTTTGATGAAGTTTAAGTAAGTTATTATAAATCTATTTATTTATTCTTACTATTGACTTAATGTCATCCTAGAAAATGTT

TTGAGAGTTTGGTCATGAATTGTATAAATGAACAACAGGACTATTGAGGATGCAATTAAGATTGCCAACAATACAAGATATGGCCTAGCATCAGGCATTG

TGACAAAGAGTTTGGACACAGCCAACACTGTGTCAAGGTCCATTCGTGCTGGCATTGTTTGGATCAACTGTTACTTTGCCTTTGGGAATGACATTCCTTA

TGGAGGGTACAAGATGAGTGGATTTGGAAGAGATTTTGGAATGGAAGCCCTACACAAGTATCTTCAAGTTAAATCTGTTGTAACTCCCATTTACAATTCT

CCCTGGCTTTGAATATCTCTTTGTGTTGTGTGGGTATAAACAATCGTGAACAATGCAGAAGCTTCTTTACACATATACTTCATCAGATTAATAAAATTGG

GGCAAGTTATCTTACTCTACCAGTTCTTTTTATGGAGTTTAATTTCTATGTAAAGTAAGTACATATTATTTTTTTTATTGTCAAGCCAATAAAAAAGTTA

TATGTAGTAGATATAAATTTTAAGGTAATTATTGTAAAAATCAACACACTTGTCATTATTGGATAATACAAAATCACTTCTTTACGGGTATGTCTTTCTT

AAGTTATCAAAGGCCACTGTTGAAACTCGAAATAAGATTGCTCTGTCGTATTT

>Glyma.09G189500 | Chr09:41438057..41442332 forward

CCAAATGATCAACCAGCTAGCTACCAATATATACCATCATATTCATATAATGCATATGCAGTGTTGTGTCGTGCTACATATAAATAACTCTTGTGCTCCC

TCTACAGTCTACACACACTCCCAGAAACTTGTTTGGTACTTGACACTCAGATTCACTCAAGTGGATAAGGATGGCAAATCTCAGCAATAGCCACTCAGAA

TCCTTTGTCAAGATTCCAACAGTAAAGTTTGCCAAGCTTTTCATCAATGGAGAATTCCTAGATTCTGTTTCAGGTTCCAATTACTTTCCTCATCATTTAC

CTCTATTCTACAACATTAGTAAATGTTTCTTCCCTTTATATGTAAAGCAAATAGTTTATTCACTTAAAGTTATAAATGATTCTTTTATATTGTTATCTAA

TCACAGATCATAATATATTATAAATATGTTGTGTTTTGTAATAACTATCATAAAAGTTATATCAATAATAAATTTTGATTAGTTGGCTACAGTAGAAATT

AAACTCCGTATATAAAAGAAACAATATTTTTCATAGAACCAAAATGGATTAGATACATGGACCTGGCATAGAAATATATCTTTATTCTAGAAGAGGAAGC

TTTATTTTGTAATTACATACAATTTGGAAAGTCCATTTAATAGGGAGGAGGGGTTGGTTTGATAAGAATAGTATTGATATGAATTATACAACGCCTTTAG

TAATTGATATGAATGATGCATTTATGTTAGAATTGTTAATACATGTTAAATTGTCAGTACTTTATTTTGTTTCTAACAAATTTGAAATTATTCTTTTGCA

AGTAAAAACGTATTTAATTAAAATGATCCCTTCTTTTTTATTTTTGAAAAGTTCAACTGACTTATTAAAACTTGTATGCACTTAGAATTTTCTTTTTTTT

TTTGGAAAAAAAAAGGAAAAACGTTTGAGACTGTTGACCCAAGAACAGAGGAAGTGATTGCTGAAATAGCAGAGGCTAACAAAGAAGATGTTGATATTGC

TGTGAAGGCCGCACGTGAAGCATTTGATTTTGGTCCATGGCCACGCATCCCCGGTGCTGTAAGTTCAGCTCTTCACCTATCTATTTATTTCAATTATAAT

TAACTGAAAAGTTGTGTACTCATCACTACCAAAAGCTGAGCTGTGAGTTTTCAAGGTAGAAAGTTGCCTTTTCACCATTATTCATAACAAAAGTTGAAAA

AAGAATTTAATTGTTTGAATACAAATGCTTTATAAACTATTCTAGTCTGTGTCAAAAAAAAAAAAAAACTATTCTAGTCACTGCTTTTATTTATTTAGCT

GTTGTCCTCAAGTTTTGTCAAATTTTTCCAATACCTCTTCTTTTTTATCTCTCTAGTCTCTACTGATATTCCTTAATCGTTTGAAAAACGTTAAAAGAAA

TAGAAGATCATGTGCAATTTTAATAAAATCTTAAGATAGGGAGAGTTAATATAATCTGCTCTACTTATTTTCATATGATTGCATTCTATTCCAATTGGTT

TTTTTGCATGGTTATCAAAATAAAAATAGTTTAATTTGCATGTCCTTTCAATATATTTTTACACCAACCTCACCATTTTACTATGTTATCATGTTTATTA

ATCTGATACGACAATAATGTGAATTTTTATTGAATGTCAATATAAAAGTGATAGTATAAAAGTATATGAAATAGAGTTTGCAAAAGGTTCACACATTATC

TAACGTACAACTTCTGAATAATAATAAAAAAGAAGTCAAGTGTCAACAATTTTACACATGCAGAAGATAGGCAATGTTCTCAAGCTGTCAAAAGCAGTGT

GCAGCATTAACTAACTTGGTATGATCATGGGATGTTGGACAGGAAAGAGCAAAGATTATGCTGAAATGGTCACAGCTAATTGAACAGAACGCAGAAGAAA

TAGCAGCATTGGACACCATTGATGGGGGAAAGCTTTTCAGTTGGTGTAAGGCTGTGGATGTTCCTGAAGCATCCAACATCCTACGTTACTATGCCGGTGC

TGCTGATAAAATTCATGGAGATGTGTTCAAAACATCTCGCAACCTCCACTTGTATTCTCTGATGGAACCTGTTGGTGTTGTTGGACACATTATCCCTTGG

AATTTCCCTACCGTCATGTTTTTCGCCAAGGTTGCCCCGGCCTTGGCTGCTGGCTGCACCGTGGTCATCAAGCCTTCTGAGCAAACACCTCTGTCATCAC

TCTTTTATGCTCATCTTTCTAAGTTGGTATGGTTAAACACATTTTGTTTATTAGTTGCTTATCAAACACAGCTAAAGTAGCATTTGGTAGATGTGTTAAG

ATGGGACAGAACATACCATGTTGGAGATTTCACATTGACAAATGATATGATCAAAATAGTGTATATAAGTGGAGAGATTCTCACTCCATATTACATTAAG

CATTTTAGCCATATCACCCGTGTGTACTGTTTTATTTTATGTTTGGTACATCTTAAAAATAGAACATCGAGTAAAAAAGTGGTACATTTTGGTTCTACCC

TTTTTAATTGAATCAAAGAAAATGAAACACGTCTTTAAGTCAAATGCTACCTTAGGAACATTAGCATGTGGCATATCTTGGTATCTTGGAAATTTATGTT

TGGTTTGTTAGTACCATTAATACATGGATGATGGACATGAATATATGCTGACAAGGTGTTTTCTGTTTTCCCTTTTGTTTAAGGCTGGTATCCCAGATGG

AGTGCTCAATGTAGTGCCCGGATTTGGCTCAATTGCAGGGGCTGCAATAAGTTCACATATGGACATTGACGCGGTAATTTGTGTGAAACGTTCTATGGCG

TTGATAAAGTTGAAGGGTATTGCATTTGTCACCAACTCCTAAGTTTTGCTGTGATTATGTTTTATGTTACAGGTCAGTTTTACAGGTTCAACAGAAACAG

GTCGTAAAATAATGCAGGCTGCAGCCTTAAGCAATTTGAAACCTGTTTCACTCGAATTAGGAGGAAAGTCACCACTTTTGATTTTTGATGATGCTGATGT

AGACAAAGCTGTTGATCTTGCTCTCTTTGGCATCCTACACAACAAGGTATATTTTCTTCCAAGGAAATTCTGCAACATAGAAAAGAACAGACTTATGCAG

TTTGAATAATTTTCAGGGAGAAATCTGTGTTGCATTCTCCAGAGTTTATGTCCAGAAAGGGATTTATGATGAATTTGAGAAGAAGGTAGTCGAGAAGGCT

AAAACTTGGGTTGTGGGGGACCCTTTTGATCCTAAAGTTCAGCAAGGACCCCAAGTAAGAATACTTAAAATTGCCAAGTGCACAACTATCTTCACACTAG

AATTAAAGTGATGTACCTCATTACTAGTAACCCTCTTAACATTTTCTCTTTCAATCTCCATGTAGCTGCTAGCATAAAAAGAAAATTCTTCGATGTAATT

CTGACATGATTTTCTAATGCAGACTAGTAAGGCACAATATGATAAAATTCTTTCCTATATTGAGCATGGAAAGAGTGAAGGAGCCACACTGTTGACTGGG

GGAAATCCAGCGGGCAACAAGGGATACTACATTGAGCCTACCATTTTTGCAAATGTTAAGGTGCATTCGAGCTCTAAAAAGAAGCGTGAAACCGGCTATC

TTTATCATAATTGAACAATTGACCTTAATTATTTTGCTTCTTTTTGAAAAATTCATTTAGGAGGACATGCTGATTGCACAAGAGGAAATATTTGGACCTG

TGATGACACTTTCCAAGTTCAAGTGAGTCCATCTTATATTCCTTTTCACTTAGTACAAGAAAAAAAGTTTGTTATTGTGCAACTGAATGTTGAACCAAGT

TTGATATACGTGCAACAGGACCATTGAGGATGGAATTAAGAAAGCCAACAGCTCCAAATATGGCCTAGCAGCAGGGATTGTGACCAAGAACTTGGATATT

GCAAACACTGTATCAAGGTCCATCCGTGCAGGCATCATTTGGATCAATTGCTTCTTTGCCTTTGATATTGATTGCCCTTTTGGAGGGTATAAGATGAGTG

GATTTGGAAGAGATTATGGACTGGAAGCACTTCATAAGTTTCTCAAAGTTAAATCTGTTGCAACTCCTATTTACAATTCTCCTTGGCTTTGAAACTTCAT

TTTCCCAGGGTGTTTGTAAACACTAAACATATACATCATTTGGATCAACTACTTAATACAACGTTTTTATCTCTTTTTTTGGGATGCTAAGCATTAAGTT

TTAGGCATCTTCTTAGTAATTATAATTTGTAAAGAGCTTGTGTGATGCCCAAGCACTTCAAAGAAAAGTAATGCAA

>Glyma.04G181100 | Chr04:44704411..44709036 forward

ATGGCTGTCTTCAATTCATTGCATAAAACTTCCACAAGATTTTTTGTTCCCACAGGGAGAAACTCTGGCAAGTGGGGAAACGTTAATAGATTCAGCACTG

CAGCAGCAGTTGAGGAATTAATCATTCCACAAGTTCCAATAACATACACTAAGCATTTGATAAATGGACAATTTGTGGATGCTGATGCTGCTTCAGGTTT

GTCTAGTTTGAATTTAACCAGGTTTGTTTACTTTGCTACTTGACAAAAATTTAATCCGACAATTTTAATTTGAGAAAAGTAATTTATCAGAGAATTTGAA

TTTCTGTAATCTAGAATTTATTGTTTGAATATTCTTTTGTGAAGAATTTAAATTTATGAAAAATGTGAAATTTCAATTTCTTTTTAAATGGTGAGAAATT

GAAAATTTTTTCTTACAAAAAGATCTTCTAAAACGTTTGCGTATTTTTTTTACTAACTTTATCATCTTTTCTATCTGAAAGTTCCCAAAATCTTTAAAAT

GTTAAAAATTCTTGAATTTAACTTTTGTTATCCAAACACAATTTTGAAAATAAAAGAATTTCTGTTGAAATATTTAAAATTCTTAGAATTTAAAATTTCT

CAAAAATTTTAAATTTTTTCATCCAAACAAACGGGATTTCATTTGCCATATTTCTTATGGTCTCAGGGAAAACTTTTCCAACATATGACCCGCGCACAGG

AGAAGTGATTGCTCGTGTTGCTGAAGGTGATGCAGAAGATATCAACCGTGCAGTATCAGCAGCTCGCAAAGCCTTTGATGAAGGACCTTGGCCAAAAATG

ACTGCTTATGTAGGCATTTAAACTTTCCAGTCTTTTCCCTTTGCTTGAAGAAAACTGAATTGGTGCAGAGTAAATAATCTATGCATTGACAAGTAAAAGG

TTATTACACTGTCCTTTAATCACAAATTGTCGTGGATGATAAAGTCATCAAGAGTAAATAACCTATGCACTAACAATGTAGAAGGTTATTACACTCTCAT

TCAATCACAAAGTGTCACCTTTGATAAGGTGATTGAATTTTGTCATAACTACCTTAAAAGTCAAACTTACCATATTTTTAATCGGTTGACATTTTATGCT

ACGGTACATAAATATTAAATTCATATGAAACATAACAATGATTTGTTAACAACTTCTTCAGGAAAGATGCCAGATAATACTGCGCTTTGCTGATTTGGTT

TAGAAACACAGTGATGAACTTGCAGCTCTGAAGACATGGAACAATGGAAAGCCTTATGAACAATGGGCCACTTCTGAATTACCAACGTTTGTGCGTTTGT

TTCGTTACTATGCTGGTAGGTTCAGTACTAACTGAAATCAAAAGTGTTTGTTTTATTTTTTTCAAGACAACTTGCTGATATGTTCCAAACAATTTTCTTT

TATTGCAGGTTGAGCTGATAAAATCCATGGTCTGACAGTGCCTGCTGATGGAAATTATCATGTGGAGACATTGCATGAACCAATTGGTGTTGCAGGACAA

ATCATACCTTGGAACTTTCCTCTCCTCATGTTTGCTTGGAAAGTTGGACCAGCTCTTGCATGTGGAAATACTGTCATCCTTAAGACTGCTGAACAAACAC

CTTTAACAGCTCTCTATGTGGCAAAAGTGAGCTCATAGATTTTTCTCTCTCACCCTCCTTTTCCTAATACACAATTAGACTGTTATCATCAAGATATCAA

CCATGTACTTGCTAGTCTAGTCCATTGTTGCATGTATATCTCCCAAAAACATTGCTCTTGTGATACTTCATTATATTTCTACAATTTTACCTACTCGTGG

AAAAATAAATAATCAAATAAAATTGGAATTCTACATATTTGGTACAGCTTATGGATTTAATTTGGTTATAAAAACCATCATAAGTTACTGATAAATAATG

CCTTCAAGAGGTCTTCATCTTCTAGAGTTGGCATTGCAGGATAATTTCTCATGGCAAAGTCTTTTTATTCTACCTTTAGGTTTTCGACTGTTATATTGTA

ATTTTGGGATATCGTTGCAGTAGTTTAAGAATTAACATCAATTTTTGCTGACATGATAATGTGGTACTTCAGATTCTAAAGGAATATTTCAGTTACATTT

GTGTTTTGTACTTGGTTTAACTCAGTGTGAACCCTTTTTGGCAGGCCGGTCTTCCACCAGGTGTTTTGAATGTAGTTTCTGGCTATGGTCCAACTGCTGG

TGCAGCTCTAGCAAGTCATATGGATGTTGACAAGGTATTTTGAAGAACCAAGCATCTATGTGGCACATTGTAGAGCATAAAAAATGAACCTGGTTTGATA

TCTCTAGGATTTAGGTTGCTAAAGTTATTTAGTGTGCTTCCATATGCATAATATTTGCCAGAATGATTGGTCTGAAACCCCACTTAAGGTACTATCCTTT

TTTGTCAGGGGAGAGTAGTACTTATCTGTGCACTGCTATATATGATGTATGGTCATTTTGGTATCCCCTTAAATTAGAATAAAAATTTCCACATAAATTT

TATATGAAAAAAATTGTTTAAAAATTCTTCACCCCAATTTAAGGGAAAATGAAAATGACTGAACCTTGGAATTGATCAGTGCGAAAGAAGGCAAGACAGA

CTATCATATTCAACCTTTTTTTTTTATCTTTTAGTATACATAATTTGATTCATCTTCCTAAAATCCTAATTAGTAAGTTCCTCTTAATAGCTTTAGATAT

TAAGGCATCTTATGCAAGAAAGTGCCCGCATGCATTTGCTTATCATTAGATTACCCTTTCCTTTACTTAATGATATTCTGGTAAAATTCAACCATTAATG

TCAAAATATTATATCAAAGTTTAACATCTTTTGTTCTTTTGCAGCTAGCATTCACCGGATCTACTGAGACTGGAAAAGTTGTACTTGAATTGGCCGCAAG

AAGCAATTTGAAGCCTGTGATATTGAACTTGGAGGGAAATCTCCTTTCATTGTGTGTGAGGATGCTGATGTTGACCAGGCTGTAGAACTTGCACACTTTG

CTCTGTTCTTTAATCAGGTTTGTTCAACCAACTCTCATCAACTTTCTTGAAGTTGAATCTAGATATGCATGTGTGAAGAACATGCTTATGATTTATGTTG

GATTTTTTATAGGGGGCAATGTTGCTGTGCTGGGTCTCGTACCTTTGTACATGAGCGTATCTATGATGAGTTCTTGGAGAAAGCAAAGGCTCGTGCTTTG

AAACGTGTTGTTGGGGATCCCTTCATAAAGGGTGTCGAACAAGGTCCTCAGGTACTTTTTCTCTTCGAAGTTATCAACATATGACAAATATGTTAGCAAC

ACATTCTCTAACATTCTCTTTCTAAAACTCTGTTTCTTGTTGAGTGAAATTTATGTGAGTTCCACTAAATATGTTAGTCTCATTCCCATTCTTGGGTCCT

ATTCCCAAACCACTGAAATTCAGATGACTTTTAATCAAGCATAGATAGTGTGTCGGAAAAAGAATATTAAAGGTGTGTTTTGCTAGTACTCTTAGGCAGA

ATATTATTGATTGTGTTCTTTCTTATCATTTTTGTTCCTTTTACAGTTACTAGTGAGTATTTGAGTTCAATGTATTTGAAAATTAGAAATTAACGTTTGA

CAGAACACCTTCTACTTTGTTGTTCATTAATCAATACATTCTTATGTTACTATGATGGAACAAACTAACACTGTTCCTGGCGTTTGGGGAAAATCTCAGA

TTGATGTGGAACAATTTTAGAAAGTACTTGGGTACATAAAGTCTGGTATTTAAAGCAAGGCCACCCTTGAATGTGGGGGTGACAGAATTGGCTCAAAGGG

CTTCTTTGTCCAGCCTACTGTCTTCTCAAATGTTCAGGTATTAACAAATTAGCACAAGTGATTCACAAAATCTACAATACCTTGAAGCATTTTCTAACCC

GTGCTAATAATCAGGATGACATGTTGATAGCAAAAGATGAAATTTTTGGCCCTCTATAAACCATCTTGAAGTTGAAGTGAGTGAAACTTCAATTCTCAAT

TTTTGTTTCCCATCTGAATGCACTCTACAACACTCGTTATTGGGTGATATTCATGAAGATTTGATTAAATATGTGGAGGTAACTTTCCTCGTTTTGTGGG

TCCAATTTTCAAACTAATAGGACTCACATGAATTTTAACCAATAAAAGATAATGTGTTGGAAAAAAACATATATTAGGGAGTGTTAATGACACTTTGTTT

TACAATGATGCAACATTTGTTTAAAACTTCCTGGTATCAATGTTTAATGTAGACCAATGCAAAACGTTATTAAACATTAATGAGGAACAACAATGAATCT

TTTAATTGAACATTATTCTAATTTTGTTCAAATAATTATTTTTGGATTATCCTAGGGATATTGACAAGTAATTCGAAGGTCAAATGCAACGCATTATGGT

CTAGTTGCAGGTGTTTTCACTAAGAACGTGCACACAGCCAACACTTTGATGCGTGCAGAGAGTTGGAACTGTGTGGATCAATTGCTTTGATGTTTTTGAT

GCTGCAATTCCTTTTGGAGGGTACAAAATGAGCGGCATTAGCAGGGAGAAGGGTATCTACAGCCTCAACAATTACCTGCAAGTGAAAGCTGTGGTTTCAC

CAGTGAAGAATCCTGCATGGCTGTGA

>Glyma.02G051500 | Chr02:4665410..4670463 forward

CAACTTTGCATTTAATGAGATCACCGCACTATAAATATCCATGTCCACATGTGTGGCAAGTAGAGTTGAGGAAAAATATTAAGAGAGAAAATAATCAATA

CTACAACTTTACAATTTTCGTGGTAGCTAGACCAAGGTAGTTCAATTGCCATGGATATTGGTGGTGGAGTGGAAGAGCCAGTGAGAGAGTTGAGGCAATA

CTTCAAAACTGGAAAAACCAAGAGTGTAACATGGAGGAAAAACCAGCTCACATCTCTCATAGACCTTGTTCATGAGAATGAAGATGCTATATTTAAGGCC

CTCCATAAAGATCTTGGGAAGCACCCTGTTGAGGCTTACCGTGATGAGGTTAATTATCACTAAACAACACCAAAATTTGTAGTTTTGATTACCTGAAACA

TAGTTACAAGTTGATTTACATTATATACACTTTCATCCACCATATAGCTTGTTTGTAACTTTTTATTTATTTATTTATTTTAATTTTACCCTTTAAATTG

TATTGCTGGATTCTTGACTAGTTGATTGCTTTTGTTAGGTTGGAGGGGTAGAAAAATCAGCCAGCAAAGCTTTGAGCTGCGTTGAGAAATGGATGGCCCC

TAAAAAGGTATATGATTCTATTTCCCTTTTTTCCTTATTAATTCCTCCCTTTCATTTTAAGAACTTTTCATACTATAACATGTGATTAGAACATCACTTT

TATCGTCAAAAGCTCCAAATCAGAATTGTATGCCATTAAGGTAATGAAATTTTTTATTCAAACTTGATCTAGAAGTAATATAATGTTGCATAAAGGATCA

TTTTTTAATTGGTTGATTATGTCCCATTACTCTACCAATGTATTCACATCCTTACATTTTGACTATAGCGTATAATGATTCGTTGACCATTGTTCTTGTT

CTGTTCAATGTTTTTTAGCTTCTTCAACAATTTTCTTGTGATGACATGTGTCAGCTGTTAACCTTTTTTCTTTGTTGGGGCTATTATTGCAGAGTGATAT

CCCTTTTCTTTTCTTCCCAGCTAAAGGAGAAGTGTTGTCAGAACCACTTGGTGTGGTTCTCATAATTTCTTCTTGGAACTTCCCAATCAGTGAGTAAAAA

TTTCCAATTCAATCCATGTTATGTTTACTTGTTAGTTGTTACTGTTAGATTTCAGAATTATTCTTGAATCAGTTTCCTAGATACTTTTTCATGTACATCT

CTTCATTTTTATTACAAAATCTTGACCAATAATATTTTAGTCTATAAAAAGTCTGTAAGTCATTCTAGATTTATGGAATTATCAGCGATCAAAGGTTAAG

GTACTATTTACCTCAAGAATTTTTTGTAACAGGATCAGGCTGAATATAAGTTCAGCAATGCTTCAATTGTTGTCATATTTTTGGTGTTTTAAACTTATCA

CAAAAATGTTAATATATTTACACTATTATGCAGTACTGGCATTGGATCCAATAATTGGGGCAATATCTGCTGGAAATGTTGTAGTCATTAAACCTTCAGA

GCAAGCTCCAGCATGCTCTTCTTTTCTTGCCAATACCATTCCTCGCTACTTGGACTCTAATGCCATCAAGGTGATTGAGGGTGGAGAAGATGTCTGTGAA

CAATTACTGCGACAAAAATGGGACAAAATATTCTTCACTGGTATTATTTTTCATACCAAGCAACTTCTTGCTAAATTGTGGTGTGTGTATATGTATATGT

TTACTGTATATATGAAACCTAATACATGTCTGGGGGAACATTTGCATACTTGATCTCAGATGAACATAGATTTGGAAAAATTGATTTTTGCTAAAAATAA

GTGTTAATTTAAGTGATTTATGTTTAGAAACTTTTATCTCCAGAGTAAGTTTTCAGTGAAACTTAACCTGAAATTTTCAACTCAACCCAACACTAAAGTA

AGTTCTGCTAGTGTTTGGACCTGCGTTTGAGGCATTCAAACCAAATCCAAAAATAGCATTCATTTGATTGGCTGAACACCATTTCCTATTACTTGGTGGT

TTGCTTATAATCTCATTTAAGAATAAAATATAATCCTACAAAGATGACTACAAACTGAATTCTGGTTCTAGAATTATTTCTCCATGGTGTATAAAATTTT

CAGGAAGTCCACGTGTGGCAAGCGTAGTGATGTCTGCTGCTGCAAAGAATTTAACTCCTGTTACTCTAGAGTTGGGTGGAAAATGCCCTGCCATACTGGA

TTCCCTTCCCAATCCTTCAGAGTTTGAGGTAGTTCACATGAGTTATTAGTATTATTAGTATGCTTGCCACATTCAATTTCAAGGTCTAATTCAGTTTTCT

TTTCTTTGTACATTTGTGGGCATCAAGTTGGCAGTGAAAAGAATTGTAGGAGGAAAGTGGGGACCTTGCAGTGGTCAAGCTTGCATAGGAATAGACTATT

TGCTTGTTGAGGAGAAGTTCTCATCTGCTGTGGTATTCATTACAATCATCAACAGCTATTTTTGCTAAAATATTATTGTGAATAAATAATCATTTTGATT

TCTGAATGTAAATCAATGATAATTTTGTTCTTGAAAGTTTAAAAAAAAAAAATTAGTCCACGTATATATAAAAAATGTAACAATTAAATCTTATTGTTAA

ATTTTGTAAGATCAATTTATCATTAAATTATAATATTTGAAGACTAATTTATCAATAAGTTAATTTTTACATTTTTTTTACACATTTTGGATAAATGTTC

ATTTACCCTTTTATTTTTCTATTAGTAAGTTGTTGTTTCTCTTCTAGATAAAACTATTAAAGAAGTTCATAAGAAGATTTTATGGTGAAAACCCAGTGGA

GTCAAAGGTAATTTCAAGAATAATAAACAAGCAGCACTTTGAGAGATTATGCAATCTTCTCAAAGACCCTCTTGTTGCGGCTTCCATCGTTCATGGTGGT

TCAGTCGACGAAGAAAACTTGTAAGTTCGGCCTGCAGGGAATAGAAATGGTTAGTTAAGTTGATTTGTTTAACTCGGCAATTTCTTCCAATCTTTTCAGG

TTCATTGAGCCTACAATTTTGTTAGATCCTCCACTAGATTCTGAGATAATGGCAGAAGAGATATTCGGCCCACTGCTTCCTATAATCACAGTGAGAATCA

ACAGTCCCATTTTATGTTTTCAATTCTCACATATATTGTGCATTGTCTCATTCAACTCTTGTTGCACTTGCAATCTGTGCAGTTGGATAAAATTCAGGAA

AGTATTGAGTTTATCAATGCAAAGCCAAAACCTCTTGCCATTTATGCCTTCACCAAAGATGAAACTTTCAAGAGAAAGATTCTATCAGAAACATCCTCAG

GAAGTGTCGTATTTAATGACACAATGGTTCAAGTAGTGCTTTTATCTTCCTTGTCTTCCCTAACTTAACTTTCTTTTCATAATAAGCTTTTATATATATA

TATATATATATATATATATAAAAGGATCTATTCTTGTTATCATGATTCTTTTTCTTAAGATTTAATAATTAAGATTATAATCATTAGATCTTAAGAAAAT

AAATGATAAACACGAGAAATAATTCTAAAAAAATTATTCTTTGAGTAATATATATATATATATATATATATATATATATATATATATATATATATATATA

TATTATTTTTGTTTGTTCTGAGTTTTGTTATAGACTTTTGTTGTAAATTAAGTGATGATATTATATCATGTTAAAATCCCTTTCAATGTATATCTGTCGT

TGGAATGGTATCATGTCATCACTAAAAATTTGGAACAAAACTTTAAATAATTTTAAAAAAATTATTAGGAACTTAAAATAAAAATCACGTATATATTAAA

CATCTAAAAGTTATTTAAGTTTATCAAGTATTAAACTCGGCTCGGATTGGGTGGGCTGGCCCGTTGGCCTGGGAACTGGGCACCCAATTGGTCTGAGCCA

CCTATTGGGTCGGTTTTGCATATGATTCGATCTGACTCGGCATGATTTGATCTCGAATCAGTGACTCGATGACTTGGTTGACTCAGTGAGTCATACATTT

TTAAGTTTTTTTTTCATTTAAATAATATCACTCGTATTAGAAAAATAAATTTATTTCTTACACTTGTACTTTTTATAGATTTGCTTATGTTTTTTATCAA

TCACATCTTAAATTTTGAGATGATAAAAGTGTTATTATTTTCAATTTTCTTTTGACTAATTTGTGTGTATATTAGCTTATGTTTACTATTATACAACAAT

AATATATATTTTAAATTCTAACTTAAGGTTATTTATATATACCGAATTCTAAAGAAATATGTTACATGCTCAAAATTTTAAACAAGTTCATAATTATACT

TCTTTAAAATTTATTAAACTTTAAATCTTAATTGTTTAAATTTACACAAATTTAATTATTTAACTGTTTTTAATATTAACTGGGTCATGCGAGTAATTGA

TGATTCATTTGATCAACTCCTAACCCAGTGATCTCCTATCCTGACGGAGTCAATGATTGATAACCGGGAGTAATACTTTCGTTGTGTGTGCAGTTTTTAT

GTGATACGCTGCCATTTGGAGGAGTTGGCCAGAGTGGTTTGGGAAGGTACCATGGGAAGTACTCTTTTGACACTTTCAGCCATGAAAAAGCTGTAATGCA

TAGAAAGCTGTTCCTTGAAATTGAGCCAAGGTACCCTCCTTGGAATAAGTTCAAGCTAGAGTTTATCAGATTAGCATACAGGCTGAACTACTTTGGACTA

GTACTGCACATGCTGGGCTTGAAAAGATACAACTAGATCAATACTCAGCACAATGTTCATCTATTCCTCAAATACATGTTGATTAAAAAATAATTAAGCT

TTATTGTGACTGAACATGAGTTGTGGGAAAATAGGAGCAAGAATCCGTATTTGCAGAAGTTGCACAAGTAGTGAACATCAACTTGGCGTTGTAATAAGCG

AACCTTTGTATCTCTTTTTTTTTTTGCTAGTAATAGGACGTTTTTACTTCAAATGTCTTAACAGTCCAAACATTGTCAAACATTTTTAACTTCTCTTTTC

ACCTGCATTTGTCCACCTCTATCTATGGCTGAACCATGAATATAGTGCCCTTGC

>Glyma.04G248500 | Chr04:51551718..51557683 forward

GCACCAACTTCTTCAAAACGACAAATATGAACCCTACTGAGCCACATTAGCGTTGCTCTCGCACCGCCAAGCCACTGCACAGATAATGTTCCCATGGTAT

GATTCAGAGAGTCACGTCCTTGATTATTGGAGCAAGCACGCACGCTATCCTTATCTATCTCTTCTCTTCTTATTGCTCATTATTACGCGTCTCTCTCGTG

TAGTATATATATACGTATGGTACTGTTCTTTCAATCATTAAACTCCTTTATATTTACACACACATATATACCTCTCCTCCTAATTTATATCCCAAGTTTC

CACCTATCCAATTTCACCGTTGCTAGCTCCTGTTTCTTTCTTTTTTGTCGGAGAAAATAGTGATAGTAATGTCGGGTGAGGAGACACAGAGGAATGTGTT

TGGCGCGGAAACGGCGTCGTCCTTGGTGAAGGAGCTGAGGGACAACTTCGGTAAAGGAACCACGCGAAGTTACGAATGGAGAGTGTCGCAAGTGAAGGCT

CTATTGAAGGCGGTGGTTGAAAACGAGGACCAGATCGTCGGTGCTCTTTGTTCCGACCTAGCCAAGCCTCCGCTCGAAACCGTCGTCTACGAGGTACTAA

TTAGTAATTAGTACTGTACTTGTCTTCATGTTTGGCACCGATCTTTAACTTAATTTCTGTTCTACGATTTATGTTTCACTTTTTTTTTAGTACATGTCAA

TCGAATACAGTGTGGGAATTTATAATAATTTATTATTTTTTTTTGAAAAATTTAATTGCTTGAATGTCTTTAAATGTCATAGATTCTTCGGACATGATTT

TCATTTTGTTTAAAACACAAGTGTACAAATCAAAATTTTGACAGAATATCAAAAGTTTTCTATCATGTGTAACGACAAATTTTTAATTTATATAAAAAAA

TGTAAAGATCTAAAATCCTACCATTTTATATAGAAATGTATACGGTATAGGACGGATTACTTATGTCTCTTTGTTAAAAGTCAGATATATTCAAATTACA

CCCTTAGTAAAAACTTGTATGAAAATTATCAATGAATCCACATTCTAATAACCCTATATTTTTTTAATAATATTTAGAAAGAGGAAGGAAGAATGGTCTG

TGATAAGAGATATGATGTGATAAAATAAAATAAAAAATAATGATAGGTGTTGATATATAGAATGTATAATACATATGTGAATCACTCAAAATTATAATAG

TTTCATCCATCATGAATATATTCAACTATAATACTATATTATTCAATTACACTCACAATATATCATTTAATTATTTAATAATTCAATCAAAAATTAAATT

TTCTAACGCCTCATCAAGCATTTCAAAATCTTAAAAGGATTTAATCGTGTGTGAATTGAGCTATAAGTTGTCAACCATCTTTACCTGATGAATGATTTTG

TCTGCTTATAATACGGACCGACAAAGTGTTGTGGCTGCTTGTTATATTTTTAATGGACCGACAAAGTGTTTTGGCTGCTTGTTAATTTTCCACGGACCAC

AAATTACTGAGATTGATTAACTGTTAGTTTTCTGATGGAAGATTAACATTCGATATATACATATATTAAAGTATTAGTATTTGTCTCTTTCCTGTAAATC

ACGTGGTGAAATAAACATTTAAACACGCAAAAGTTTTCTAATTTCTAACATGCATCGGCCAGTGGAATAATCCCTACTACTTCAATTTGTATGGTACATA

TAATAATTCTGTCGGAGGAATAGGTTTTTAAGTGAGAAGAGGCTAGGCAGTGACCACAAATGAGCATTAGATCTTATCTGAATTTCTTTATTAAATAAAT

TAAATTAATACAAGAGGAAAGTTCTCAACGGGATTTTATAAAAATTATGGGTTATAGTGTTTCAATTTTTTTATCATGGAATAAATTCTTCATAGCTCTT

TTTATCATTATTATTAAATTAGTTTCTTAAGTATTTAAAATCTAAACTTTTAAATATGTTAGTTTAATTCAATCCTTGACTAAAATCTTATATTTAAATA

TTATGATACTTTAAGGAAAGGTCTGAATATTTTATGGTTTAAGTATCATGTGAAATTCTAGTCTTTAAAATTAAATATAGTTCGTTTTCTGATGAAATAT

AAATGATAAATATGTTTTTTTAATGAATATAAATAATACTCCTCCTGTTATTTTTAAACTGCAAACATGATGATGCATAAAACTAAATAGATTATTGTAT

ACATATACTATATTTAATTTAGGTGCTGTAAGTATCCATAAAAAATGACATATTATAACTATATATATCGCCAATTTTTATATAATTAAATATCTGTATG

TATCAATGATATGGCAATGCCTTATTAATTAACTAGTACATGTTTTGATTTATTACTTTGAAAAAACAATCAAATAAGTTTAATCATAACACTAATTTAA

GCAAGAACCCGGAAAGCCATTTTTACACATTTATGATATTAATTATTCCACCTTTCTTCAGACTTGCAATATATTATCAACTAAACATATTTTGAAATTT

GGAGCTTCTTCGACAAGGACACGAAGCTGATCTAATGTGGGGCAAACAATTAGTTTTAACATACAACTGCGTTAAATATCATATTTTCAGTCTACTCTTT

ATTGATGTAGTTTGTCATGCAGATTGGTATGTTTCAAAACTCATGTGAAGTCATACTGAAGGAATTGAAACATTGGATGACGCCAGAAAAGGTACATTGT

ATCTGAATTTCTTCCGCTGAGAAGGAATTTGACAAATAATATTGCTTATATATTGTATTAGTAATTTGATTCACCTAATGTTCTTTTTAATTTCAGGTCA

AAACGTCAATCAGAACTTTTCCTTCTTCAGCTGAAATAGTACCTGAACCGCTGGGTGTCGTGTTAGTCATCTCTGCATGGAACTATCCAATCTGTATGTT

AATATTTACTTGACCTTGATTTTTATATATTTTTACCTGACATTTTCAGCCTTTCTTCTTTTTTCTTTTAAAATCTACATTAAGTTATTGATAACTACTA

ACTACATTATTACCTTTCTCTGTCCATCATTCCAGTGTTGTCCCTTGATCCAGTAGTTGGAGCTATAGCAGCTGGTAATGCTGTGGTTCTAAAGCCATCC

GAAATTGCTCCGGCCACTTCATCAGTACTAGCAAAGTTGATAGAGAAGTATATGGATAACTCATTTGTAAGAGTTGTTGAGGGGGCAGTTGATGAAACAA

CTGCACTATTGCAGCAAAAGTGGAACAAGATTTTCTACACAGGTTTTTCTTCTTTGTCATTAATTATTCTAGTTTTCTTTATAATTGGTCTGTTTTTATG

TATATATACACGTGTAATTTTGAAATCCCTCCTCAGGGAACCAAGTAAATGCATATATAATTTGAACATAAAATAGCAAGAGAATAATTTTTTTGTCTCA

TTCAAGTTATGTATACATTTTGTAGTATGAATAATTCTTGGATTTTTAGGTAATGGAAGAGTGGGAAAAATAGTGATGACTGCGGCTGCAAAACACCTTA

CACCAGTTGTACTGGAGCTTGGAGGAAAATCTCCTGTTGTTGTTGATTCAAACAACAATTTGCTGGTACTACTATTGCCTAAGCTAAGCCTTTTTGTTTT

CCCAATTACTTGCTATTACCTTAACCTCTTTCAGACTCATTCTTATGTTAATATGTATTTGCAGGTAGCAGCTAGACGAATAATTGCTGGCAAGTGGGGA

TTGAACAATGGACAAGCCTGCATTTCTCCCGATTATGTTATAACAACAAAAGACTATGCTCCCAAGTTGGTATGAAATTTTCCCTATGTGTAATTGGAGT

TATGAATCCATGTCTTTGTTTATTTATTGTTTGATTCCTCCTATTTCTAGGTGGATACCCTAAAGACTGAATTGGAGTCATTTTATGGAAGGAACCCGTT

GGAATCTGAAGATTTGTCTCGAATTGTGAGTTCCAACCACTTTGCTCGCTTGTCAAAGCTCTTGAATGATGATAAGGTTTCTGGCAAGATTGTTTATGGA

GGCGAAAAGGATGAAAAGAAATTGTAAGCTCCCTTTATCTGTTTTTGATTAAATTTGACCTTATAGAGAGACCTTTTATTAATATTAAAGAAATGCAATA

GATCTTGACACTGGCCTTTTTATTGCCCCAAAAATTTCAGAAGGATTGCTCCCACTATTTTATTGGATGTTCCGCAAGATTCTTCGATAATGGGTGAGGA

GATATTTGGTCCATTGCTTCCCATCATCACGGTATGAATAAAGGCACATGAAGAACTGTTTGTATGATGCATATTTTCTTCTTCAAATTTCATTTATGTT

CTTTGATTGCTTTTCCAATAGTCTAATAGGTACAATGTTCACATAGTTTTGCAACATGTTTTCAACCTGCAAGAGTAATTGATATGTGCTTCTTCAGGTG

TGATAAGAGTCACAAGTCCCAACTAGAAAAGCAGCAATAAGATATATTGGGGAAAACTTCAATTGATTATGTGTTACTTAGAGATGCTAGTCTTTTTGTG

GACATTTACAAAAGTTTTATGCTAAATGTTAAACTATATAACGTGGAAAGAGGTGATATAATTTAAATAGTTCTCAGCAATGAACTAAAGCAAAGGAAGA

TCAACTTTCATACTTTTTTTTTTTGAGTGGAACAGGTCAATAAGCTCGAAGAAAGTATAGATGTGATCAACTCAGGAGCAAAGCCCCTCGCTGCATATGT

ATTTACAACCGACAACAAGTTCAAGGAGCAGTTCGTAAAGAATGTTTCTGCCGGGGGTTTGCTCGTCAATGACACTGCCTTACATGTAATATTTGTTCTT

TTCAAAAAAATTCATGAATATGCATGAGTTTGCGTGTGCCTTTTGATGTGATTCTTATCCCTGAACAGATTGTTGACAACTTGTTGCTATGAAATCATTT

TTTTTCCTTGGATACCTTTAAACTTGAATGGATAGATAATTCTGTGTTAATGTCAAGAAAATAATTTTTGAATGAATATATATTTCAGTTTCTCTCAGGA

TAGAGAAGAGTTACTTTTTTTTTTAAAATGTGCGTGGTGCATGTGTGTGCAATAATCATATCTCTGTTTCTGTTGATTCACATTGAATTTGATGAATTCT

TCAATTTTAGATGTTTTGGTCATAGATTATGATTCATTTTAAGAACTTTTCCCCATCTATACATATGATATGGATATAGGCATTTTGCATTGTTATTTAA

AAATCCTTATTTTCTGTTTCATATCAACTTAAAATCTGAACATTTTTCATGTGTATTCTCCTTTTCCTGGTGCAGCTTGTGGTTGATACCTTGCCATTTG

GGGGAGTTGGGGAGAGTGGAATGGGTGCATACCATGGGAAGTTCTCTTTTGATGCTTTTACACACAAAAAAGCAGTTCTTTATCGCAGTTTTGCTGGGGA

TTCAGCAATAAGGTACCCACCATACACGGATACAAAACTGAGATTGATGAAGGCTCTCGTCGGTGGTCGTATCCTCGGAATAATTCGTGCTCTTTTCGGA

TGGTCCTAAGCTTAACTTGTTTTGAAACCTAGCTATTTTTTATGTATCTTTCGTTCTGGTTTTTTTGTACTTCATCATGCTGTGTAGCTTTATTATTATT

TTTGTTTTTTTAGAAGAAAACCAATAGGCGAGAGTTGCGCATGAAAAAAGCAAAATAAATTAAATAATCATTGCTACTTTTCTTCGTGATTGTGTTAGAT

GTCTTGCGGTTGTAATGTGGCTAATGCCTTTTAGAGGCTATGCAGGCTGTGATACTATAAAATACTGTGTATATATGCACATATTAACATGCCTTTCACG

GAAGGACAAGGCATGCATGCTCTTATGTATGTCATAAATTTATAATCCAGATTTTGCTAGCGATTTTGGTTTCTCATGGTGATAGTAGTGTTGTGAATCT

GACATGTATTTTGAAGAGCACAGTATTATTAATATTGGAATTGAAAGCTGTGTGTTTGGAGTCAAC

>Glyma.06G114300 | Chr06:9268543..9272590 reverse

TATTCACACACATGTATATATGTATATATAGAGAGCTCTCCTCTCTTCCCTAATGTATGTGCTTTAACTTTGAAACCCAACCCAATTTCACCGTTGCTAG

CTCTTGTTTCTTTTTTGTCGGAGAAAATAATAATAACAATATATAATGTCGGTTGAGGAGATGCAGTCACAGAAGAGGAATGTGTTTGACGCAGAAACGG

CGTCGTCCTTGGTGAAGGAGCTGAGGGACAACTTCGGTTCGGGAAGGACTCGGAGTTATGAATGGAGAGTGTCACAAGTGAAGGCTCTGTTGAAGGCGGT

GGTTGATAACGAGGAACAGATCGTCGACGCTCTTCGTTCCGACCTAGCCAAGCCTCCACTCGAAACCATCGTCTACGAGGTACTACTACTTCTACTTGTC

GTGATTTTGTCTTTTTTTCTTTCTCGGTGTCTTCTAATCACTAGATTAGAAACTAATCTTCTTGTGGTTCATGATTGTAAACAACTTTGTAGATAATGAG

AATCAAACACAAATCTTCACTAAATAAATTGATTTCACTTGTATGAACAGATCTTACATTACTTTTCCTGTTTGGCACCGATCTTTAATTTTCTTATCTA

CTCATGTTTTAACTACAACTTGCTTTGCTTGCGTTTTCGTGATTTTTTTGGTAGTGACGATGACCTGTTAGAGGATCAACCTCATGTGTGTTGATTAAGA

ATCGTGTCTAGTCAAATCATGTTTGTGATTCTATACGCTACACTGTTAGAATATTGACCTTCGAAATATTTTTTTTTCTTTTTTAAAAATTCGTACTCGC

TTATTATATGCATGTGCATTATGTTACGTACTCTTTCAATATAACTTTTTAACACCATATGAATTTAATAAAGAGCCTTTAGATTATATGATATTATCTG

AATGATTATTTATACTAAATTTAAATTAATTAAACATTCAGAAACTGATAAAATATTCGATGTGTTGAAATGAATTTGATAATTGAAGTATTTAAATTGT

ATATTAAGTAATTTATTTAATACAAAACTAATTATTATAATTCTAATTTTCATAATTAAATATTTGTGTTGCAGGTTGGTATGTTTAAAAACTCATGTGA

AGTCATACTGAAGGAATTGAAACAATGGATGAAGCCAGAAAAGGTATATTTTATCTGAATTTCCTCCACAGAAGAAGGAATTTGACCTAAATATAATGTT

GTGTATATAATTTATTTATTCATCACATGATGTTCCTTTTATTTCAGGTCAAAACATCAATCAGAACTTTTCCTTCGTCAGCTGAAATAGTACCTGAACC

ACTGGGTGTTGTGTTGGTCATCTCTGCATGGAACTATCCAATTTGTATGTTAATAAATATTTACTTAAACTTGAGTTTTTTTATATATACTTTGAGTTGA

CATTTTCAGCCATTTCTGATAAGTTATTCTCTGTCTGTATTGCCAGTGTTGTCTCTTGATCCAGTCGTTGGAGCTATAGCTGCTGGGAATGCTGTGGTTC

TAAAACCATCAGAAATTGCTCCTGCCTCATCATCATTATTATTAAAATTGATAGAGAAGTATTGTGATAACTCATTTATAAGAGTTGTGGAGGGGGCAGT

TGATGAAACAACCGCACTATTGCAGCAAAAGTGGGACAAGATTTTCTACACAGGTTTTTCTTCTTTCTTTATCATTATTCTAGTTTGAATCCCTTCTCAG

GGACTAAACGTACAAATTAATGCCTAATTTGAACATAAAACAGCAAGAAAATTAAATGAATAATTCTTGGATTGTTAGGCAATGGAAAAGTGGGAAGAAT

AGTGATGACTGCTGCTGCAAAACACCTTACACCAGTTGTGCTGGAGCTTGGAGGAAAATCTCCTGTTGTTGTTGATTCCAATGTCGATTTGCAGGTATTG

CCTAAGCCTCTTGGTTTTTCAATTACTTTTTATTACTTTATTCTCTTTCAATTTCATATTTTACTTAAAATTAATTTGTATTCACAGATAGCAGCTAGGC

GAATAATTTCTGGCAAGTGGGGATTGAATAATGGACAAGCCTGCATTTCTCCAGATTATGTTATAACAACAAAAGATTGTGCTCCCAAGTTGGTATGGAA

CTTTCCCTATGTGTAATTAGAGTTCTGAATCTATGTCTTTGTTTATTTATTGTTTGATATCTCCAATTTCTAGGTGGATGCCCTAAAGACTGAATTGGAG

AAATGTTATGGAAAGAACCCGTTGGAATCAGAAGATTTGTCTCGGATTGTGACTTCCAACCACTTTGCTCGATTGTCAAAGCTATTGGATGATGATAAGG

TTGCTGGCAAGATTGTTTATGGAGGTGAAAAGGATGAAAAGAAATTGTAAGCTCCCTTAATCGATCTGTTTTTAATTAAATTTAACTTTTAGCCAGCTTG

GATGGGAGATTTTATCATTAAAGAAATGCAATAGATCTTGACATTATTGGCTTTTTTACTGCCCCAAAATTTGCAGAAGGATTGCTCCCACTCTTTTATT

GGATGTTCCGCGAGATTCTCTGATAATGGGTGAGGAGATATTTGGTCCATTACTTCCTATTATCACGGTATGAACATTTACATATGTTTTAATTAGATAA

ATGCTTATAAAATAACGTGGAAACGTCTAGGTGTTTGTAATTTAAAAAGTTCTCGGTAATAAACTTTATTTGCTTCTTTTAATTTAAAGCACTGGAAGGT

TAACTATAATAAGTCTATAATACTTTTTTTCTTGCTTGTAACAGGTGAATAAAGTGGAAGAAAGTATTGATTTGATCAACTCAGGAACAAAGCCCCTGGC

TGCATATATATTTACAACCAACAAGAAGTTAAAGGAGCAGTTCGTAATGAATGTTCCTGCAGGGGGTTTGCTTGTCAATGACACTGTCTTACATGTAATA

TTTCTTATTTTAAAAATTTTCCATGAATGCATGAGTTTGCGTGTGCCTTTATATTATAGGATTTTATCGCTGAACAGGATTAAGGTCCTAATCAGCTTCA

AGGTTTGGGTTGCTGACAGTTTGTTGCCATGAAAGATGAATTCATTTTTTAAAAGATATATTTAAACTTAAATGGATAGATAATTTTTAGTTAATGTCAA

GAAAATAAATTTTAAATATATATATGTATTTCAGTATCTCTCTTGGGGGTGATAAAAAAAAGAGGTACCTTTTTAAATGTGTGTGATGCATGTATGTGTG

TGCAATAATCATGTCCTTCGATCTGTTGATTCGCATTGAATTTGATGAATTTCTTAGATGACTTGGTCATAGATTATGATATCTATAGATATGGATATAA

CATTGTTAATCAATATTTCTTATTTTTTGTTTTGAATCAATAATCATGTGTATTTTCCTTTACCTGGTGCAGCTTGTCGTTGATACTTTGCCATTTGGGG

GAGTTGGGGAGAGTGGAATGGGTGCATACCATGGGAAGTTCTCTTTTGATGCTTTTACACACAAAAAGGCAGTTCTTTATCGCAGTTTTGCTGGTGATTC

ATCATTAAGGTACCCACCATACACGGATACAAAGCTGAGATTGATGAAGGCTCTCATCGGTGGTCGTTTCCTCGGAATAATTCGTGCTCTTTTCGGATGG

TCCTAAGCTCAATTTGTTTTGAAATCTAGCAGTTGTTGATGCATCTTTCGTTCTTTTTTTTTTTTGTACTTTATCATGCTGTGTAGCTTTATTATTATTT

TGTGTTTTAGAAGAAAACAAATAAGCGAGAGTATTGTGCATGAAAAAAGCAAAATGATCAATGCTACTTTTCTTCGTGATCGTGTTAGATGTCTTGTGGT

TGTAAATTGTAATGTGGCTAATGCCTTTTAGAGGCTATGCAGGCTGTGATACTATATAATACTGTGTATATATGTACATATTAACATGCCTTTCACGGAA

AGACAATGCATGCTCTTATGTATGTCATAAATTCATAATCCAGATTTTTCTAGTGGTTTTGGTCTCTCATGGTGATGGCAGTGCTGTGAGTCTGAATATT

GAAGAGCACTGTATTATTAATATTTGAATTGAAAGCTGTGTTTGCGTT

>Glyma.13G030400 | Chr13:10019610..10026287 forward

AATCACGATCCATCATCACACCACTCAACTCTGCACGGCATAATCCTCTTTCTTTTTTTCTCTGTTTTCTTTACTTTCCTCTTCGCGTCGTTTTGTCCGC

GATAATGTCGTCGACGCCGCAGGACTCTGTCAAAACGACGGCGTCGGCGAAGAACACCGCGTTCGACGCGGAAGCGGCGTCGCGGCTCGTGAATGAGCTT

AGGCGAAACTTCGCTTCGAACAAAACGCGCAGTTACGAGTGGAGACTGTCGCAGCTCAACGCGCTCGAGAAACTCGTCGTCGTTCACGAGCAGGAGATCG

TCGACGCGCTCCGAAACGACCTCGGCAAGCCGCCACTCGAAACCGTTGCATACGAGGTCTCGTCGTTTACACTGTTACTCGTTCGCGTTTTGTTTATTTT

TTATATATATATTTTTGGTTTGACTTCGGTAGTGTAAAAGGAATATTCCGAAAAATTTTCGCAGGTGCATTTTGTGATAGAGATTTTCTGCGGTTCATTT

ATTACAGTGACAGAGTGTGTCATCTTCTCAGAAATTCTTTCTGAGCAAATAACAATCTTATGTTATGTTATGCTATGTGTTTGTGTGTATGCTCAGTGTT

GACGTGTTATGTTTTGCTTTTTTATTTTTTTTGGATACTATTATTTATTTATTTTTAATTTTAATTTTTTTGGGAAGGGAATTTCTGTTGAATTAAATTG

GTTGGTTATTTGTCTGTGTGGATTGCGGTTCGCGTTTCTTTGCCAATGGTGGTGGAAACGTTGCCGTTTGGGGGATTGGATTTGGGGCCTGGTGGTTTGG

TGTGAGCATTAATGGCGCGTTCTTTGCGTCCCAATTTTAGGGTGGGCCAGTGTTGTGGGCCTCTAAATTTGCCTTTGAATCTATGATATGTGGCTTTAAT

TCATGGATTTTGTTAACGTCAGCACTTATGGTTGATCCTAGATTGAGAAAAAGTAGTTATTACGTTAGAACGGTATAGTGAAATTTTAGTAAGTGTTGAG

TTTAATATGCATGCAGTCCTCTTTCGTGTAGACTGTGAATTAATCAGCAATTAAGTTTATTATGACTTAAAAAATATATAATAAAAATTAATAAATTTAT

CATGTCCGTTTAGTAATTGGGTGAGTACATGTTTATTCACAATTGTTCTGTTTCCTAATGATAACTTTTCATTTTTAGTTGGTTAAACTAACTGAATTTA

TTGCTAGTGCCACAAAATGTATAGCAGTCAAGGTGTTGACTTAAACTACTGTCACTGAATGAGTTGTTTCAGCAAACTTTGTCCTTGATCCCTTCTGGCT

TCTTTTTTTTTGAAACGTAATTTTTTAGTTTTTGTGATTTGTGTTGTTTGAAAAATGTTAAAAGGTACACTATTTTTTGGGTGTATGGATAGAAGGAAGA

GATAGATAGACAAAAAAATTGAGGGAAAAAAAGAACGAGAAACTGTTATATGTGAGAAATGCAGAGAGACTTGAGAAGTAAAATGGGTGAAAGTGAGTTA

TAAGAGAAGTTGAAAGTGGGATTTTCCTTTTTAGTTTATCGGAGACAATTCCATTCTTTCACGAGGTTGACAAATGTGATTTCAGTTTGGCTGTGAGATG

CCATTGGCTATTATAGTATATAACAGAATTTACTAAATGACACATGCCCACCTAATTTGGTACTTTTCAAGGACAATTCAATTCACACATTGTGTGCCTG

TTTAAACAGGAAAATATATGCCAATCAGGGTTAGCTAAACTGGTAGGAATGAAATGTAAAAATGAAAATGGTTTAAGTACCTATGTAAGCTGTCTACTGA

GACTTGTGGCATGCCTAGATTGTAAAATAGTGCACCCTCACTTCCCACATAAAACTGTTTGGTACCAAAAAATGAAAATCTTTGCATTTGGAGAAGATCC

AGGGGTCTTGCTGTTTACGACGTTGTGTTTTTTAGTAATGCCAAATGTATGGCAGGCAATTCATAATACAACTCTCAAAATTGCAAAACAATTCATTAGG

TTTATGCATGTTGAATTGGTTATTTCTAATTTCAAATATTTGGATTTTGGCATGTTAGGCTCCTCAGTCCTGCCTAACTTGTTTCTCCAAACTTCTCCAC

TTGAGTTTGAATGTTATTAAAGTTTATAAGATTTTGTTAGGTTGATATTTCTGTATGGATTGGAGATCTTTGATGATGTGCAACCAGTGGTCCTGTGCAC

TCAGTTATTAATATCCCTTGGATAGGAAAACACACTAAATCTACTTTATCCAAGATACTGAAAATTTCATTACAGATTAACGTAAGACGTAAAATTTGCT

ACTAAACAAACATTTTGAAGCCTTTAATAAGGACATGAATCTGAAAATGTGGAATAAAAACCCATTTGCCTGACTATTGCTTTTTGAAGGATTGGTATAA

TAATCAACAGTCAACACAATGGTTTTAAGTATGATATTTTCCATCTTCTTGTAACTGGGGTAGTTTGTATTGCAGATTGCTATGTTGAAAAACTCATGTA

GAATCGCACTCAAGGAATTGAAACATTGGATGACTCCTGAAAAGGTATACATTATGTGAATTTCATTGCTGACATTGTTTGGTACTTTTGATTTATCACA

TGAATTTCCCTTTTATTGCAGGTCAAAACTTCAATCGCAACTTTTCCTTCTTCAGCTGAAATAGTATCTGAACCACTGGGGGTTGTGTTAGTCATCTCTG

CATGGAACTACCCATTCTGTATGTTAATATTTAGTTGGTATTGTTTCACATACTTTTGAGCTGACATCTCACCTTTTTCTTTCCTCCAATCTCTGCAAAA

TCTATATTACATTCTCTGTCTATCTTTCCAGTGTTGTCACTTGATCCAGTCGTTGGAGCTATTGCAGCTGGTAATGCTGTTGTTTTAAAACCATCAGAAA

TTGCTCCTGCCACATCATCACTGCTGGCAAAGCTTATCGGAGACTACTTGGATAACTCATGTATTAGAGTTGTTGAGGGAGCAGTTGATGAAACATCTGC

ATTACTGCAGCAAAAGTGGGACAAAATTTTCTATACAGGTTCTTGTTTGTCACTCTTCTGTTTTCTTGGTGGTTCTGAATCCTGAAATGACATTGACAAT

TTTCCTTTCTTTTATATATAGTGGCATGTTAACAAGTACACTGTGTAACATACATTTTTTAACAGTCTTTATTATTTGGTGAAATTTCTGTGAGTTCCAC

TAAATGATGCAGGTCCCCCATGGATTTCACCCGATAATGAAAATGTTTTGAAATGAGAATTTTGATATTCCTTTTGGATAATTATATTCATATAAGTGCA

TTATTTCACCTGACAAAAATAAAAAGAAGTAAAAGTATAAAAATATTTTTCTCATTCAAGTTATATTCATAATTCCTAACTTAAGGATCACAATTAACCA

AGTTATGTCGGCAAACTTTTCTGTTAGGATACCTTATTCCATATGATTTTGATTTTGTTTTTGGTTGCTGAAGATGTCAGTTTAAGTATAACATTTGTTC

TTTAAATTCCTAAAAATTGACAGGCGTGCTACAAAAACTTGAATGTGGATTTTTTTCATTGTTAGCCGTTGGATGGTTTGCCACATGCTATTCTTAATGA

CATAATACATGTCCAATATTGCTTGTTTCTATAACCTTAATATGTCTGTTAAGCATGAATATCTATATTCTGTTTTAGTTTGGTGGATAACAAGGGAAGC

ATTTAACTTTTTAATCATTAATATAGGATTGTGCAGATTATGTTTATACTTTGTAGGCTTCATAGGCATCTAATCTTGGAATGCTTCTTTCTTTTCTATT

ATTTGGAATAGAAAATACTTCATTCTAAATAATGATTGGCAAGGTTGGTATGGATTGACGATTATAATACAGCTTGAATACATCTGGGATGTTACTGTTA

TTGGTCCTGGAAATAGTTCATGCAGATTACAATTTTGAGCAGTATCTCTTACTCATGAAGTATCTTTAGAGGCATATAGCCATATAGCTTGCTGAATCAC

ATGATTCACATCATTACTCAGGTTCAGGCATGATATTTACTTGGGAAAGCAGGATAGGAATTTGTTGAGCAACATAGGTGTCTCAGTGGATGATAAGATA

ATTTAAGTATAAAGCTTTTGAAAAGCTGTATCTAATTGATACGTGACAGGAAATTTGTATGAGATCATCGCTTATAGGTAGTATGGCACCTGACAGTGCT

CCAAACAATATATGCTGTAGGATAATTGATGATTAGGGATATGTTAGTCTTGAGTCTCTTGAGTCTTGGCCATTATTGTATTAAGTTTTAAGGAAGACAA

GGAATAAGGTTATTGTCTTAATCTATGTTCAATGGTTGTGAGTTGTGACCAAATGCCCTTGTACTTGTTTTGGACTTTCAGTTGTATTGACTGTACTTCT

ATATGTTCCATATTGTTAAGACTCTTAGTGTCAGTCTTATATAAGAAATCTAGTTTGTTATAAATATACAAGGATCTGATGCTGGATTTTCTTGAATGCT

TGTTTTATTACAATGATACCCAGGTCTGATAATATTCTTTACCTAAGTAACTGTTGGATTTTTAGGTAATGGACGAGTGGCACGCATTGTGATGGCTGCT

GCTTCAAAACACCTAACACCAGTTGTGCTGGAGCTTGGAGGAAAATCTCCAGTTGTTGTTGATTCAAATATCAATTTAAAGGTATACCTTTGTCCTGTTT

TATTTTCATCAATTACTTTCCATCACTGATCTCTTGCAATCTCACTTTTAAGATTAAATTAGTTTGAATTTGAATTGGCAAGGTAGCAACAAGACGAATA

ATTGCAGGAAAGTGGGGTTCTAATAATGGGCAAGCCTGCATTTCTCCAGATTATATTATAACAACTAAAGACTATGCTCCCAAGTTGGTAATAAATTTTT

TCCTAAGTGTTATTAGAATTCTAAATTTGTTGGTTTGTTCAGTGTTTGTTTCCTTCTATTTCTAGGTGGATGCGCTAAAGACTGAATTGGAGAAATTTTA

TGGAAAGAACCCATTGGAATCAAAAGATTTGTCCCGTGTTGTGAACTCCAACCACTTTAATCGGTTGACAAAGCTCTTGGATGATGATAAGGTTTCTGGT

AAGATTGTTTATGGAGGCCAAAAGGATGAAAACAAATTGTAAGTTCTGTATATGCTTTTGGGGGTACTTATTGATGAAATCTTGATTTAGTGAAATACAA

TAAGATCTTGACCCTAGTATTTCTTTTTCTCAATTTCAGGAAGATTAGCCCCACTGTTCTATTGGATGTCCCACGGGACTCTTTGATTATGAATGAGGAG

ATATTTGGTCCTTTACTTCCCATCCTCACGGTAGGAGTTAAGACACCATTATACCACAATAACTGTTTTTTGTGAGATTTTCTTCAAAATCTCATTGGTT

GCTTGCTGTTACAGTAATGAGTACTATATTGAAACAAGATATATATAACTTTTATGGTCATGCCCTGTGGGGAGAATTTCAATTATGTCACGTGCAAAAA

TCATCAAAATAAACAGCTGCTAATATTTTGTATACATTTGAAGATCATGAAGCCAATTGTTAAATCAACCTTTCTGGTGATTGTAAATTGTAAAATGTTT

ATCGATAAATTAAAGCCACATCAATCAAAATGTTTCATTTTTATTTTTATCTTTTTTCATTTGCTTGAAACAGGTTGACAAACTAGAAGAAAGCTTTGAC

GTGATCAATTCAGGACCCAAGCCTCTCGCTGCATATATATTTACAAATAACAAGAAGCTCAAGGAGCAATTTGTTATGACTATTTCAGCTGGTGGTTTGG

TTGTTAATGACACTACTTTACATGTAATTTCTAGTCTACTCTTGAATCTCTGAACCTGTGTTGTGTACCTCTGATTCTTATATGCAAACAAAACAACAGT

ATATATAGGTGATCACTAAAATATGCTTCAAGACTAAAATTAAATTTTGACTAAAGGCCATGTGATAAATTGTATTATTGTACAAGATTAAAATTAAAGA

AATCGTGAAAATAATTTCTTATTGTTTTTTCAGGTTTCATTTCTATAGTAAGGAGCATCTATTAAATAAAATGTGTTGTTTGCTCCCCATGCATTTGTTT

TTATTGGTTCATATTGAAACGAGTTTCTTCAGATTAAATGTAGTTTTTCTGTCTATAGATGCTCACAATTTTATTTAATGTTTTGAAATAATTTCAAGTG

TGAACACTATTCACCCGTGTTTCTATTTTCCATCTTTTTTGCCTGGTGCAGCTTGCAGTTCATACTTTACCATTTGGTGGAGTTGGTGAGAGTGGAGTGG

GTGCATACCATGGGAAATTCTCATTTGAAGCTTTTAGCCACAAAAAAGCTGTTCTCTATCGCAAATTTATTGGTGATGCTCCAGTAAGGTACCCACCATA

CACAAATACAAAGATGAGATTGCTGAAAGCTATCATAGGTGGTGGCATACATGGTATTGTTCGTGCCCTGTTTGGTTGGTGATAGTCTTTGATTTTAATT

ATATTGGAATCTTGATGCTGTAAATACGGCTTTTATTTTGGCTTCCTTGTATTTTAATCTCCAGTTATTATTATTATTTTTATGTAATACTCATCAACCT

TGACTTTCGGATGATAGTAAATAAGCATGTATTGTGTTGAAGATTAGATAAAAGATAGAGAAGAGCAGGTATATTTTT

>Glyma.14G152100 | Chr14:32978400..32985147 forward

AAATCTCAAATCACGTTAGCTTGCTAACTTGTTCTCACTTCTCAGTTTGTTTGTTTGTATTTGGCAAACTATAAAATAGGAAATATCTCTAATCTCAAGA

GGAATAACAACACGCCACAATCCATCGCATCACTCAACTCTGCACCGCAAAATCCTCTTTTATTTATTTATTTTTTTCTCTGCTTCGCGTCGTTTTGTCC

GCGATAATGTCGTCGTCGACGCCGGACTCAGACAAAACGACGACGTCGTCGAAGAAGAGCGCGTTCGACGCGTTAGCGGCGTCGCGGCTCGTGACGGAGC

TCAGGGGAAACTTCGCTTCGGGCAAAACGCGTAGTTATGAGTGGAGATTGTTGCAGCTCAACGCGATTGCGAAACTCGTCGTCGATCACGAGCAGGAGAT

CGTCGACGCGCTCCGAAACGACCTCGGCAAGCCGCCACTCGAAACCGTCGCATACGAGGTCTCGTCGTTTACACTGTTACTCGTTTGCGTTTTGTTTATA

TATATATATAGTTGGTTTGACTATGGTAGTGGAAAAGGAATATTCCAAAAAATTGAAAACAGGTGGATTTTGTGATAGAGATTTTCTGCGGTTCATTTAT

TATGGTAACAGAGTGTGTCATGGCCTCAGAAATTCTTTCTGTGCAAAATAGCAATGCTTATGTTATATGCTATGTGTTTGTGTGTGCCCAGTGTTGATGT

TAGGTTTTCCTTATTCTTTTTTTGGATACTGTCATTATTAATTTTAATTTTAATTTTTGTTGGAAGGGGAATTTCTGTTTAATTAGATTGGTTGGTGATT

TGTCTGCGTGGATTGCGGTTCGAGTTTCTCTTTGCCTACGGTGGAGGACACGTTGCCGTTTGGTGTGAGCATTAATGGCGCGCTCTTTGCGTCCCAATTT

TAGGGTGGGCCAATGTTGTGGGCCTCTAAATTTGCCTTTGAATCTATGATATGTGGCTTTAATTCATGGATTTTGTTAATATCACTTATGGAAATAAAAA

TGGATTGAGTAAGAAAGTAGTTATTACGTTAGAGATCCAGTATAGCGAAGTTTTAGTAAGTGTTGAGTTTAATATGCAGTGAATTAATCAACAATTATTT

TTATTATGACTTTTAAACAAATTATAATAAAAATTAACAAATTTATCATGTCTACTTGTGATTGGATGATTACATGTTTATTAACAATTGTTCATAACTG

AATTTGTTGCCAGTGTCACAAAATGTAGAGCTGTGGAAGTATTGACTTAAACTACTCTCACTGGCTGAGATGTGCAGCACACTTCGTCCTTTTTTTTTAT

CAACAAATGTTAATATTGTTAGTTTGTTAGTTTTTTGTTGGCGGGAAGATTCGATTCTGCAACACTTCGTCCTTGTCTGGATTTTAGTTTCTGATTTTTG

TTTTTTAAATTAAATTTTGGTGTTTGTGGGGTGTGTTGTTTGAAAAATGTTAAAAGGTACCAATATTTTTTTTGGGTGTATGTATGGAAGGAAGAGAGAG

ATAGACAGAAAAAAAATCAAGGAAAAAAAGAAAGAGAACCTGATAGAAATGAGAAATGAAGAGATTTTTTTTGAGAAAAAGAAATGAAGAGAGACTTGAG

AAGAAGAAAAATGGGTGAAAGTGGGTTATAAGAGAAGTTGAAAGTATGAGTATAATAACTCTTGTTTAGGTGTTGGTGTGGGATTTGCCTTTTTAGTTTA

GCAGAAGCACTTCTATTCATTCTACGAGGTTGACAAATATGATTTCAGTTTGGCTGTGAGAGGCCATTGGCTATTATAGTATATAACACAAGTTACTAAA

TGACACATGCCCACCTAATTTGGTACTTTTCAAGGACAATTCCATTCACACGTTGTGTGCCTGTTTAAACAGGAAAATATGTGCCAATCAGGGTTAGCTA

AACTGGTAGGATTGAAATGTAAAAATGCAAATGGTTCAAGTACCTATGTAAGCTGTCTACTGAGACTTGTGGCATGCCTTGATCGTAAAATAGTGCACCC

TCACCTCCCACAACTGTTTGGTACCAAAAAATGAAAATCTTTGCGTTTGGAGAAGATCCAGGGTTCTTGCTGTTTACTCTGTTATGTTTTTTAGTAATGC

CAAATGTATGGCAGGCAATTCATAATTGCAACTCTCAAAATTGCAAAAATAATTTTCATTACGTTTAAGCATGTTGAATTGGTTATTTCTAATTTCAAAT

ATTCAGATTTTGGTATGTTAGGCTGCTCAGTCCTGCCTTACTTGTTTCTCCAAACTTCTCCACTTGAGGTAGAATGTTGTTGAAGTTTATAAGATTTTGT

TATGTTGAAATTTCTGTATGGATCGGAGATTTCTGATGATGTGCAATCAGTGGTCCTGTGCACTCAGTTATAATACCCCTTGGATAGGAAAAGACACTAA

ATCTATTTTATCCAAGATACTGAAAATTTCATTACAGATTAACGTAAGACATAATATTTGCTGCTAAACAAACATTTTGAAGCCTTTAATAAGGACATGA

ATCTGAAAATGTGGAATGAAAACCCATTTTCCTGACTAGTGCTTTTTGAAAGGTTGGTATAATAATCAACAGTCAACACAATGATTTTAAGTATGATATT

TTCCATCTTCTTGTAACTTGGGTAGTTTGTATTGCAGATTGCTATGTTGAAAAACTCATGTAGAATCGCACTCAAGGAATTGAAACATTGGATGACTCCT

GAAAAGGTACACATTATGTGAATTTGTTGCTGACATTGTTTGGCATAACTCTTTTGATTTATCACATGAATTTACCTTTATTGCAGGTCAAAACTTCAAT

CGCAACTTTTCCTTCTTCAGCTGAAATAGTATCTGAACCACTGGGGGTTGTGTTAGTCATCTCTGCATGGAACTACCCATTCTGTATGTTAATATTTAGT

CAGCATTGTCTCACATGCTTTTGAGCTGACATCTCACCTTTTTCTTTCCTCCGAGCTCTGCAAAATCTATAAAGATGATAATATTACATTCTCTATCTAT

CTTTGCAGTGTTGTCACTTGATCCAGTCATTGGAGCTATTGCAGCTGGTAATGCTGTTGTTTTAAAACCATCAGAAATTGCTCCTGCCACATCATCACTG

CTGGCAAAACTGCTCGGAGACTACTTGGATAACTCATGTATTAAAGTTGTTGAGGGAGCAGTTGATGAAACATCTGCATTACTGCAGCAAAAGTGGGACA

AAATTTTCTATACAGGTTCTTGTTTGTCACTCTTCTGTTTTCTTGAGGTTTAGAATCCTGAAATGATTTTGTTTCTCATTCACAAAACTTTCCTTATGAT

TAGTCTTCTTTTAGGTATAGAGGCATGTTAGCAGTACACTGTGTGACATACATTTTTAACAGTCTTTATTATTTGGTGAAATCTATGTGAGTCCCACTAA

ATGATGCAGGTCCCCCATGGATTTCACCCGATAACAAGGGAAGCATTTATTTTTTTAATCATTAATATAGAATTGTGCAGATTATGTTTGTACTTTGTAG

GCTTCATAGGCATCTAATCTATATACTTTTAGAAGGCTGTTTCTTGGAATGCTTCTTTCTTTTCTACTATTTGGAATAGAAAATACTTCATTCTAAATAA

TGATTGGCATGGTATGGATTAACGATTGTAATACAGCTTCAATACATCTGGGATGGTACTGTTATTGGTCATGGAAATAGTTAATGACTTAATGCAGAGT

ACAATTTTGAGCAGTATCTCTTACTCGTGAAGTATCTTTTGAGGCATATAGCCATATAGCTCAGGTTCAGGTATGATATTTACTGGGGAAAACAGGATAG

GAATTTGTTGAGCAACGTAGGTGTCTCAGTGGATGATAAGATAAATTTAAGTATGAAGCTTTTGAAAAGCTGTATCTAATTGATACATGACAGGAAATTT

GTATGAGATTGTTGCATATAGGTAGTATGGCACTTGACAGTGCTCCAAACAATATATGCTGTAGGATAATTGATGATTAGAGATATGTTAGTCTTGAGTC

TCTTGACTCTTGAGAATTATTGTACTTTTATGGTTTAAGGAAGACAAGGAATAAGGTTATTGTCTTAATCCATGTTCAATGGTTGAGTTGTGACCAAATG

CCCTTGTACTTGTTTTGGACTTTCAGTTGCATTGACTGTGCTTGTATCTGTTCCATATTGTTAAGACTCTTAGTGTCAGTCTTATATAAGAAATCTAGTT

TGTTATAATATACAAGGATCTGATGCTGGATTTTCTCGAATGTTTGTTTTATTACAATGATACTCAGGTCTGATAATATTCTTTACCTAAGTAACGGTTG

GATTTTTAGGTAATGGACGAGTGGCACGCATTGTGATGGCTGCTGCTTCAAAACACCTTACACCAGTTGTGCTGGAGCTTGGAGGAAAATCTCCAGTTGT

TGTTGATTCAAATATCAATTTAAAGGTATACCTTTGTCCCACTTTATTTTTATCAATTACTTGCCATCACTGACCTCTTTGCATCTCACTTTTGACCTTA

AATTAATTTGAATTTGAATTGGCAAGGTAGCAACAAGACGAATAATTGCAGGAAAGTGGGGTTCTAATAATGGACAAGCCTGCATTTCTCCAGATTATAT

TATAACAACTAAAGACTATGCTCCCAAGTTGGTAATAAGTTTTTTCCAAGTGTTGTTAGAATCCTAAATTTGTTGGTTTGTTTAGTGTTTGGTTCCTTCT

ATTTCTAGGTGGATGCCCTAAAGACTGAATTGGAGAAATTTTATGGAAAGAACCCATTGGAATCAAAAGATTTGTCCCGTATTGTGAACTCCAACCACTT

TAATCGCTTGACAAAGCTCTTGGACGATGATAAGGTTTCTGGTAAGATTGTTTATGGAGGCGAAAAGGATGAAAGCAAATTGTAAGTTCTGTATATGCTT

TTTGGTGTACTTTATTGATGAAATCTGAATTTAATGAAATAAAGCAAATTGTAAGTTCTGTATATGCTTTTTGGCGTACTTTATTGATGAAATCTGGATT

TAGTGAAATACAATGAGATCTTGACCCTAGTATTTCTTTTTTCTCATTTGCAGGAAGATTAGCCCCACTGTTCTATTGGATGTCCCACGGGACTCTTTGA

TTATGAATGAGGAGATATTTGGTCCTTTACTTCCCATCCTCACGGTAGGAGTTAAGACACCATTACACCACAGTAACTGTTTTTTGTGAGATTTTCTTCA

AAATCTCATTGGTTGCTTGCTGTTACAGTAATTAGTAGTATAATGAAAGTAAGTGTGCACATAGTTTTTCAAATGGTTCAAGAACGATTGATATGTTCTC

TATGTGGAATAGGTGCCAACTTATCAATTTTCACATTTAATGTAAAATAACTACAAGTCTACAAGATATATATACCTTTTATGGTAAGGCCCTGGGGGGA

GAATTTCAATTATGTCACGTCCAAAAATCATCAAAATAAATAGCTGGTAATATTTTGTATACATTTGAAGAACATGAAGCCCAATTGTTAAATCAACCTT

ACTGGTGATAGTAAATTGTAAAATGTTTATCGATAAATTAAAGCCACAGCCGTTCAATCAAAATGTTTTATTTTTATTTTTATCTTTTTTTCATTTGCTT

GAAACAGGTTGATAAAATAGAAGAAAGCTTTGACGTAATCAATTCAGGATCAAAGCCTCTCGCTGCATATATATTTACAAATACCAAGAAGCTTAAGGAG

CAATTTGTTATGACTATTTCAGCTGGCGGTTTGGTTGTTAATGACACTACTTTACATGTAATTTCTATATTATTTTTACAGTCTATTCTTGAATCTGTGA

GCTTGTGTTGTGTGTGCCTCTAATTCTTATATGTAAACATAACAGCAGTATAGGTGATCACTAAAATCTGCTTCAAGACTAAAATTAAATTTGACTAAAG

GCCATGTGATAAATTGGATTATTGTACAAGATTAAAATTAAAGAAAATAACGTGAAAATAATTTCTTATTGTTTTTTCAGGTTTCTTTTCTATATAGTAA

GGGGCATTTATTGAATAAAATGTGTTGTTTGCTCGCCATGCATTTGTTTTTATTTGTTCATATTTAAACGAGTTTCTTTAATGTGGTTTTTCTAATAGAT

GCTCACAATTTATGTATTGTTTTGAAATAATTTCTATCTGAACACTATTCATCACTGTGTTTTTTGTTTTCGATTTGGCCTTTTTGCCTGGTGCAGCTTG

CAGTTCATACTTTACCATTTGGGGGAGTTGGTGAGAGTGGAGTGGGTGCATACCATGGGAAATTCACATTTGAAGCTTTTAGCCACAAAAAAGCAGTTCT

ATATCGCAGATTTATTGGTGATGCACCAGTAAGGTACCCACCATACACAAATACAAAGATGAGATTGCTGAAAGCTCTCATAGGTGGTGGCATACTTGGT

ATTATTCGCGCCCTGTTTGGTTGGTGATAGGCTTTAATTTTAATTATATTGGAATTGGAATCTTGATGCTATAGACGGCTTTTAATCTGGCTTCCTTGTA

TTTTAATCTCCAGTTAATTTTTTTATGTAATACTCATCAACCTTGACTTTTGGATGATAGTAAATAATCATGTATTGATATTCGTTTCTTTTGTCATATG

ATTGTTTTAAGATGCCCGGTGGACCTGTGTTTGTTCTGTCCTATCAGATGCCGTGCTAATACGGATATTACATGTGTTGTGTGTTGGCATGTTTCTAGCA

TGCCATTTAAGGAAATATGACTCATTGAGTAACACAATTGCAAATCCT

>Glyma.08G002700 | Chr08:195874..201005 reverse

CAAACTTGACTTGAGTGCACAAGATTTTTTTCTCTCATTGCTAAACTAAAAGTCTACAACGTTTCTTTTATTTTTGGCTATGTCCTTGTCAAATTACCCA

TGATTAATACCAAGTCTTTAAATGTGTTAAACATTATCTTCCTATTTTTCATGGCTGAGAAATCATTCCATTTCCGGCTTATATATATACACATAAATGC

AGCTTCTTTTATTTTTAATATTAGACATCACTACCAAAGATCTTTCTTTCAGCTTCTTGTTCTACAATGAAAAGCCTCTGCCTTGGGCCGTTTCTTGCTG

CTAGGTACGTATTTTTCTAACTTTTGAGCTTATTTTCTTCTACCTTTTGATTCATTAACCCAGTGATTGTTCATATATTATTTATTTTGTGATGATGAAA

ACTAAGATGATCAATGCAACTTGATGGGAAGCATTATCACCCAATTGAATAATAGAATTGCCCAAAATATGTTGAAAAAATAAAAACTACCATTTCATCA

TAATTGATGAGCCTCTTACTCCAGTTCTTCCTAATAACAGTGCCCCAGTTGGTAGAAGAGCCTATGGAGGCCACTTAAGCAGAAAGTGCTTTCAAAAGCA

ACTTCACTTCCACTCACGTTGTGTTGCTTTCTCTTCATTCATGTAAGCCCTCTCTCTCCCTCCACCTTTTGGTCATGGAGCGTTTTAATTGTGTATTCTT

AACTAAGATGTTATTTTCATGTGGTCTTCTTGCATGCATATGTATGGTTTACCTTGTGAAGATGTTCTGCAACTATATCAGTGATGCCAGAATTAGAAGA

GAAGCAAGTGTTTGATGGAGAGAAGGCTAATTTGCTAGTTAAAGATCTCAGAAAGAGCTTTGATTCAGGCATGACTAAGAGCTATGGATGGAGGGTTTCC

CAGTTGGAAGCCATAGCTAAGATGTTAGAAGAGAAAGAGAAGGAAATTACTGAAGCTCTATATAAGGACCTTGGAAAACCTCGACTTGAAGCATTTATAA

CCGAGGTCTTCAATTTCTTGCTTTTATAATTTTGTTTATATGCTTCATATGCCTTCTGTTAGCCATTAATTTATGGAATTCTTTCCTTTTTCTTTTTTTC

TCCATTCCCTTTTGTCATGATGGGATTTGATGTTTCCATTTGTCCAGTAATCTCCTCAGCCTCACCATCATCATCATGGTCACCAGATTTGATGTAAGTG

TTTCTTAATGCAGATTTCCCAGGCTAAATCCTCATGTAGTGAAGCACTTAAAGAGCTGAAAGAATGGATGAAGCCTGAGAAGGTAAATTGCCAAGAAAAC

ACAAGTTTCAACATGGCTTTATTAATTAACAACCAAGCTTCTTTCTTTTTTTTAACCCTTTTAGGTGAATACTTCAATCACAACATATCCATCATCAGCA

GAGATTGTGCCAGAACCACTGGGGGTTGTGTTGGTCATCTCAACATGGAACTTTCCTTTCTGTATGTAGAAGATGTTCCCTTTTCACTCTATTTATATAT

CTAAACCGTATTTGGTTATAATTGAGAACAGCTTTTCTTCATCTCAGCCAATTTCATCACTGTGGAGCTCTTGTAGTGGGATGTATTTGTCCTGTAAATA

AAACTCTTCGGCACTCTTTAAGTTTTCTTTGTCAAAATTCAAATTTCACCAGTTATTTCAAGCTCTAAACTTATTTGTGCTCTCTGTCTATGAATTTAGC

CAGAGGTTAATATTCCCTTGCTTAAACTTTCAATAAATTAGAGCCAAGTCTATTATACTTTTGTAACCTTCTCCATATCAACATAATCTTTTGATATTCA

CCAATCATGACTTGTCACTATCTAATGTGTTGACTTGGCATCTTCTCCTTGTCCTTTCCTTTCCATTGATTAGTTCTTATTTCCTATCTTCAGTGTTATC

AATGGATCCAGTCATTGGAGCCATTTCCGCAGGCAATGCTGTGGTCCTGAAACCATCAGAAATTTCTCCAGCAACATCATCACTGCTTGCAAATTTGATC

GAACAATATCTAGACAACTCTACCATAAGAGTTGTTGAAGGGGCTATTCCCGAAACATCTGCACTCCTGGATCAGAAGTGGGATAAGATACTTTATACAG

GTAATGTGTCTTCTAATTTCTATTGTATCAATATGACAATATCCTACAAGATCATACCATATCAAACAGATTCATCAGTCTCAAAGAATGTATGCAATGT

GAATAGTAAAATATCAGTGTTAATAGTATGGTCCAATTGTTTGATATTATTTTCTTTTGAAGCTGTATGAAATTTGGCTCTATTTTAAGCCATCCCATAT

ATATGTCTAATTCTAGTAATCACTTATCATAAGTTGCACCAAGAAATTTGAGTTGACACTGTTGAAGCTTTCACTTGATTATAAAGTCACACGTATTTAG

TTCATTTAACATGCTTACTTCATTACAAGGTTCCATGCATGATAGACCAGCTTTTTGCAGGTAGTGCTAGAGTAGGTCGCATTGTCATGGCTGCTGCTGC

AAAGCATCTTACTCCAGTGATTCTGGAACTTGGAGGAAAGTGCCCAGCTGTTGTTGAATCAGATGTCAACTTACAAGTAACATCATCAATGGATCTTGTT

ATTTACTCATCAATGGATCTTGTTATTTACGCATCAATGTAATTTCATGTATAACAATCACTGAATTTTCAGGTTACTGCAAGGAGGATAATAGCTGGCA

AGTGGGCATGCAATAGTGGACAAGCATGCATTTCTGTTGATTATATCATCACAAGAAAAGAGTTTGCCCCAAAGCTGGTAAGGTTGCCTCAATATTACAA

AATTCTGTGTGCTGCCTAATAAAATATATTACTCTCGATAATTTCTCACTCAACAGGTAGATGCCTTAAAAGAAGAATTGGAACAATTTTTTGGTAAAGA

TCCTATGGAGTCAAAAGACATGTCAAGAATTGTGTCTCCAAACCAGTTTGCGCGGCTTGTGAATCTCTTGGATGAGGATAAGGTATCTGACAAAATTGTT

TTGGGAGGTCAGAGGGATGAGAAGAAATTGTGAGTTGCATTCCCTTTTTAATTTTTAAGATTGCAAATATTACTTATACTATATCATAACACCAATTCTT

CCTTTATATTGCAGAAAAATTGCCCCAACTATCATATTGGGTGTTCCTGAAGATGCTATGATAATGCAAGAGGAGATATTTGGGCCAATAATGCCAATCG

TCACTGTGAGTACTAGATCACAATGAAAAAGTTAATTTAACATTTAAGACTTGTTGGCAAGGATTATGGATCTTTGTCAAGAAAATAATTCTGTTCTTGA

TGCAGGGAAAACATGTTTCATTTATTATAGGGAAAAATATGTTTTTGTTCCCTTCGGTCAAACTTGATTTTGGTCCTTATACTTTTAAATTGATGGATTT

AGTCCTTCAACTTTTGGAAAACATGTAAATTTAGTCCCTTTTCCAACTTTGTGGCAGTCAAAAACTGTCACTAAATGTTAAGTTTAAATGTGAGGAGGGA

CTCAATTCACATATTTTCAGTTGCAGGACTAAATTCATCAATTTAATACTATAGGGCCCCAAACCAAGTTTGGCCGAAAGTTCAAGCACCAAAAACATAT

TTTTTCCTTTATTTTTTTTATGCTCACATTTTCCCCTTTATTATAACTTAACCTTAATTTTCTTTTCCAGCTGGTAGTGGCCATTCTTTCGTTCCAACTC

CTTTGCAAAAACCATAACTGATTCCTGTTGACCAGTTTCATGTTCCCTGTTACAACTGACAGAGACCCTAAAACACCAGTTTTGAAACTTGCCTCTCTTT

TTCTTGTAGTATGCCACTGTGTGAATGAGAACATATTAAAAAGATGATTTATTCCTTCAAATGTTCAAGTGTATTGTCCAGATTCCAGAAAGATTTCACC

CTTCAAATATCAAGCAGTGTTAGCATGTGCTGTCTATCTTCCTTATTTCTCTTGTTCCCCTAGTTATTTCCAACCAAATTTACTCTTACAAAAATTATCT

CGGGAAGGACTTGATATGTTTGTTCATATTTAGCATAAAGTTGTTATATTTACCCTTACATTTTTGCAGGTAGACAATATAGAAGATTGCTACAGTATAA

TAAAATCAAAGCCAAAACCTCTTGCTGCATATCTCTTCACAAACAATGAGCAGCTGAAGAAGGATTATGTGGATAAGATATCTTCTGGAGGGATGCTCAT

CAATGACGCCGTCATCCATGTAAATATTCTCTTCCTACAATTTATACATGAAAAATTGTTGTATGCTAACATAATTTTCTTCAATTATGGTCATATTTTC

ATAAATCAATTGTTCTCTGATAGTGCCCCATCTGTAGAGGGAAAAAGAAAAGTTATGCTCTCTTTATTTGGCATCTTTCATTTGCATACATAAAATGATT

CAACTTAGCATGAGAGAAATTCTGATGGTTGAATGATATAAGTTGACTTTAAAAACAAAGTAAATAATAAAGCACTTGATGAACTTGGGCCTCTATCTCT

AGATGATTAAGACTTTGCAACCTAATTTTTAATAGCATGCAATGAAATGCAACCTGAAAATCCAGAGCACATATATTTATCAAATGAAACCAAAACTTAA

AAATTTAAGCTAGTATATACATGCATTTAAATGTGTTTATGTGTAGACACTTATCTATATGCATGCAGGTTGCAACTCGTGGGTTGCCTTTTGGAGGAGT

TGAAGAGAGTGGAATGGGTTGTTACCATGGAAAATTCTCTTTTGATAGTTTCAGCCACAGGAAGTCAGTTCTCTATAGAAGTTTTGATGCAGATTCTACC

ATTAGGTACCCTCCGTATACACCTCAGAAGGAAAAATTATTGAAGGCCCTTATTAGTGGCAATATTGTTCAAATTATTCTTTCTCTTTTGGGGTGGTCTT

AAGGATGATAAGGTCCCTTTAAAAAAAAAAAACGTTCATGAATTAGAAGTACTGTACTAAAACAATGTGCAAGGGCAGTGACTCGTGGCTTCCCACTTTG

GTTGAAGTGGCAAAAGATAATTACAATTTTCCCCTCGAGTTTTCAATCTCATGAATCCTAATAAAAAAAAAAAACTCATGAATCTGTTCTTTGATTGCTA

TTCCGCTCTAAATTAATGAATGAGATATCGAA

>Glyma.11G133300 | Chr11:10188790..10192565 reverse

GGACTCTAGCTTTTTCCTTTATGAAAGCCGAAACTAGTTTACAAATTGAAGTGAGTTAGTTGAACAAATATAAATACTGGTTTTGCTGTGAGGTTAAGCA

GCACCATATGGAGATCACTATGCAAACTTTGGAGAGAGACCTAAATGACACGAGGGGGTATTATGAGAGTGGAAAGACTAAGGAAGAGTCTTGGAGAGAA

TCGCAGCTCAAAGGGCTGCGTCGTTTTCTCTTGGAAAAACAAGTAGATATCATGAATGCCCTCATGCATGATTTAGGGAAACATCAACTTGAAGCTTTCA

GAGACGAGGTATCTTTTCACATATTTCTTCTGCTATTATATATTACAGTAAAAAAAGTCTTTTAATTTAGCATGGCTAAAGAGTTTCTTAAACACAAGTT

TTCTTTAAGTATTTTGGCTGTGCTATAATAATAAACTGTTTTCATATACTACAATTTAATTTCTTACTATTTGATCCATTATCGCAGATAGGAACGTTGA

TTAAGACCGTAAATTTGGCATTGAAGTCTTTGAAAGATTGGATGTCAGGAAAAAAGGTAAGATACATGCCTTTCCTTCACCTTGGAAATTGTTTGAAGAT

GACACTATGAGCATATATAGATACATATGTGAAGTAACTAGTTGCCAAATTAATTTTGATAGGCTGCGTTGCCACAATTAGCATTGCTCACCAGTGCAGA

AATTGTTCCGGAGCCACTTGGTCTGGTTCTCATTATTTCATCTTGGAATTTCCCCATTGGTGAGTTATACCCTTAGAAACAACATTTATATGTTCACACA

CACACACATATATAGGTTTGTGTAGCTAACTAATTAAGAGGTATTGATATATAAAATTGCAGGAATATCCTTGGAGCCACTGATAGGAGCAGTAGCTGCG

GGAAATGCAGCAGTCTTAAAGCCTTCAGAGTTGTCTCCGGCATGTTCTTCTCTACTTGCTTCTAGTCTCCCCACTTATTTGGACGATAAAGCCATTAAGG

TGATCCAAGGAGGGCCACAAGAGACCCAGCAACTTCTAGAGCAAAGATGGGACAAAATCTTCTTCACAGGTAAATGATAATTTTTTTCCCAATAAAATGT

GTTTTATAGGTGAGTGACTCCATATTATTTATCAAAATTAATGATAGTCTCTTACTTTAAAAATAATGATTTTAGTCTTGAATTTTTCATAATTGGTGAA

TTTCGCATCTCACCTTGTATTTTAAGTTTCCTAAAGAAAGATAAGATTTGCTAATTACTAAAAATATGAGAATCAAAATTATATTTTATAAACTAAAAGA

CTTAATCAATTGAGTCTAATTCAGTGGATTAAATTGAGTGTGTGAGTTGTTATAATTTTTATGGTGTCTCTCTTTAATTCTCACAGATAACAAAAATTAA

ACGAGTTAAACCTATTTTAATTAGTGAACTTAAAAACACACTATACTCTTTTTTTCCTTCTCTTTTTTAACTGAAGAAAATGTTGTTGTTCCTCCTCTTC

CATTGCTGTAAAAACTAATGGCAGGAAGTGCACGAGTGGGGCGGATTGTGATGTCTTCTGCTGTGAAGCATCTAACTCCTGTGACTTTGGAGTTGGGTGG

AAAATGCCCTGCAGTTGTTGATTCCCTTTCATCTTCTTGGGACAAAGAGGTCACTGTGAAGAGAATAATTGTGGGGAAATATGGGACTTGTGCTGGTCAA

GCATGCATAACAATTGATTATGTCCTTGTGGAAAAGGGTTATTGTTTAAAACTGGTACATACATACATGATACATACGCCCCTTGACATTTTTCACTGCT

CTTAATTTGTTTCTTCGTATGTCACACACACACATGTATATAGCTAGGGGCTTGTTGTGCTTATTCTTGTTTTCTTTTAGGTGGAATTGATGAAGGTCTG

GATCAAGAAAATGTTTGGACAGAACCCTCGAAAATCAAAAACTATTGCTAAGATAGTTAATAAACACCACTTCTCTAGATTGAAGAATCTTCTTGCGGAT

AAACAGGTCAAAGGTTCTGTGGTTTATGGTGGTTCAATGGACGAACAAAACTTGTAATTAACTGACTCTTTGCCCTTAATTTCTCCAGTAGATTCTCGTA

AAATAATCTTCCCTCTCCGTTAATGTCTATTTAGCATATTTTTAATGTATATCTATTTAGTATTAAGTAACATTATATTCTTTTACACATTTTTTTTTCT

CTCTCCGTTAACAGAATATTTAATACATTTTTTATAACGTAAAAGATAGATGTGTAAGAGAATATAATAAATAAAAAATAAATATTTAATTTTTAGAAAT

ATAAACACATTCATTACTTGTTATGCAATTCAATACTTATTAATTATCATATATAAAAAGGAAGTAAGAAGATATTATGGAAAGATTTAGAATTAGAAAA

GTTTTGGTAAACACTTTAATTAATATCTTATTGAATACACTTTTTTACTTTGACTAAAATTTATGATTAAAAAAATCTTAAATTAGTACTTGTCTCATTT

TTTTATTTAGTAAATTTCATTTGCAATATGATAATTTTTTAATAAATTTAACCGACAATAGTAGAAAAATATATTAGAAAAAATGTCTCAAAATGAATCA

TGCTTTACCACTTCTACAATTAGAATAATTCATTATATAGATTTATCGCATTAAATGCAATACGGTAATTCCTTAGAAAGGTCAGACAAATATGGTTGTA

CGTACAATTACTTGGCCGAAAAAACTTTATCTGAATTCGTGCAGATTTATTGAGCCAACGATCTTGGTGGATCCCCCACTGGAAGCAGCAATTATGTCGG

AAGAGATTTTTGGCCCATTGCTTCCAATTATTACCGTAAGTTTCATGCAACATGTGTCACAAAAAAAAAATATCAATATTGTTCTCAGTTATAGCACATG

TTTATACTATGTATTTCTCTATATATTTTCCAACTTGACCAATGAGTTCCATTTCTTTTTTCTTTTTCTTCCTATTAATAATTTGCAGGTGGAGAAGATT

GAGGACAGTATTAAATTCATAAATGCTAGACCTAAGCCTCTTGCCCTCTATGTTTTCACCAAAAACCATACACTCCAGAGAAGAATGATATCTGAAACAT

CATCTGGCAGTGTGACTATCAATGATGCAGTTCTACAAGTAATACCAATAACCTATATCTAACATCATTCTAATATTTATACATATTTTAATGTTCTCAC

TTAGGGCATAATATTTGTGTTAATTTGTGCAGTATGCAGCTGATACTATTCCATTTGGAGGAGTTGGGGAAAGTGGGTTTGGCATGTACCATGGGAAATT

CTCCTTTGACACATTTAGCCACCAGAAAGCAATTGTAAGAAGAAGTTTCCTCACTGACTTTTGGTATAGATATCCTCCATGGACACTAAATAAGTTACAA

CTACTTGAAGTCTCTTACAATTATGATTATCTGGGATTGCTCCTTGTCCTGTTGGGCTTAAAGAGACCATCAAAACGCTTAATCGCAGATCATGTTTAAT

GTTTAGCTAGCTAGCTCGTTACTTGGGTACTTTTATTTTATTTTATTTCTTTTTCAATAGCTGTTAAGATTTAGGAGCGTACCCAATAAAAATTGTTCTA

TGAACAATTAGTGAACGGTACTTATGGATAACAATATTTGCGTATTAAATTCTTATACAGCTTGATTAATCCATTATCGTGATAACAAGGTTCAATGTTA

TGGTTTTAGTAAGGTCGCTGTAGTACTGAATTGCAATTATTTGACATTAATCATATATCTTGTTCCATCAACCGGG

>Glyma.12G057400 | Chr12:4178669..4181863 reverse

CCGAAACTAGTTTACAAATTGAAGCGAGTTAGTTGAACAAATATAAATAGTGGTTTGCTGAGAGGCTAAGCAGCACCACAAAGCACGATGGAGATCATTA

TGCCAAGTTTGGAGAGAGACTTAAATGACACGAGGGGGTATTATGAGAGTGGAAAGACTAAGGAAGCGTCCTGGAGAGAATCGCAGCTCAAAGGGTTGCG

TCGTTTTCTCATTGAAAAACAAGAAGATATCATGAATGCCCTCATGCATGATTTAGGGAAACATCAACTTGAAGCTTTCAGAGACGAGGTATCTCTTCCC

ATATTAATTTCTTCTGCTATTATATTATATTACAATAAAAAGTGTTTTAGCATGGCTAATGAATTAAAAGAGTTTCTTACACAAAGTTCTCTTTTAAGTA

TTTTGGCTGTGCTATAATATGTGGGAATAATAAACCATTTTCATTTATAATTTCTTACTATTTGATCCATTCGCGCAGATAGGAACGTTGATTAAGACCT

TAAATCTGGCGTTGAAGTCTTTGAAACATTGGATGTCAGGCAAAAAGGTAAGATGTACGTGTTTATTCCTTCAAAATATTGTTACATAGTATATATAACT

TCACCTTTTGATAGGCTGCGTTGCCGCAATTAGCATTGCTAACCAGTGCAGAAATTGTTCCGGAGCCACTTGGTGTGGTCCTCATTATTTCATCTTGGAA

TTTCCCCTTTGGTGAGTTAGAAACAATATTCATATCACTGCATTAATTATATGTTCATCATATATAGGTTTGGTTAGCTAACTAATTAAGAGGGATTGAT

GCATAAAACTACAGGAATATCCTTGGAGCCACTGATAGGAGCAGTAGCTGCGGGAAATGCAGCAGTCTTAAAGCCTTCAGAGTTGTCTCCAGCATGTTCT

TCTCTACTTGCTTCTAATCTCTCCACTTATTTGGACAATAAAGCCATTAAGGTCATCCAAGGAGGGCCAAAAGAAACTCAGCAACTTCTAGAGCAAAGAT

GGGACAAAATATTCTTCACAGGTAAATTAAATGACTTTTTTTCTTCTTAAAAAAAATGTGTTTTAGGTGATAAGTGACTCCATTTTATTTATCAAAATTA

ATGATCGTTAACATTAATTTTCAAATTGTAATTTATAAATAAATATTACAAAATTAAATTCTTATTAAAATTAAATTTAAATAATTTATTTTCTCTTACT

TTAAAAATAATGATTTTAGTCTTGATTTTTTTTTATAATAGGTGAATTTTGCTTCTCAGTCTAGTATCTTAAACTTCATAATCAAGAGAGATAAAACTCA

CTAATTATTAAAAATACAAGAATCAAAATTATCATTTTTAAATTACAGGACTTAAACCTATTTTAACTAACAACTTAAAAACATAAACTTAAAACATACT

TTAGTCCTTACTCCTTAATTGTTTCTTAACAGAACAAAATGTTGTCTCTTCCATTGTTGTAAAAACTAATGGCAGGAAGTGCACATGTGGGGAAGATTGT

GATGTCTGCTGCTGTGAAGCATCTAACTCCTGTGACTTTGGAGTTGGGTGGAAAATGCCCTGCAGTTGTTGACTCCCTTTCATCTTCTTGGAACATAGAG

GTCGCTGTGAAGAGAATTATTGTGGGGAAATATGGGGCATGTGCTGGTCAAGCATGCATAGCAATTGATTATGTCCTTGTGGAAAAGGTTTACTGTTTTA

AACTGGTACATACATACATCCTTTTGACATTTTTCACTGTTCTTAATTTGTTTCTATATATATATATATATATATATATATATATATATATATATATATA

TAAATTTCTAGGGGCTGCTTTGTTGTGCTTATTCTTGTTCTTTTAATTAGGTGGAATTGATGAAGGTCTGGATCAAGAAAATGTGTGGAGAGAACCCTCA

ACAATCCAAAACTATTGCCAAGATAGTTAATAAACACCACTTCTCTAGATTGAAGAATCTTCTTGCTGATAAAAAGGTCAAAGAATCTGTGATTTATGGT

GGTTCAATGGACGAACAAAACTTGTAATTGACTGACTCTTTTTGCTCTTTCTATTATATTCTCGTACACATCTATCTACTATTTTAGAAAAACTGTATTA

AATAAAAAAAAATATTTAATCTTTAGAAATATAAAAAAGAGTATTGCTTATCTAACACTCCCGCAATTAGAATTATTCAATTATTAGTTGTTGATAGATT

TATCGCATTAAATGCAATACGGTAATTCCTTAGAAAGACCAGCTGACAGATTTGGTTGTACAATTACTCAGCCGAAAAAGCGTTATGTTATGTGAATTCG

TGCAGATTTATTGAGCCAACGATCTTGGTGGATCCCCCACTGGAAGCAGCAATTATGTCGGAAGAGATTTTTGGCCCATTACTTCCAATTATTACCGTAA

GCTTCATGCAACATTGTTCTCAGTTATACCACATGCTTATACTATATGTATTTCTCTATATATTTTCCAACTTGACCAATGAGTTCCATTTTTTTTCTTT

ATCTTCATATTAATAATTTGCAGGTGGAGAAGATTGAGGACAGTATTAAATTCATAAATTCTAGACCTAAACCTCTTGCCCTTTATGTTTTCACCAAAAA

CCAAACACTGCAGAGAAGAATGATATCTGAAACATCATCTGGCAGTGTGACTATCAACGACGCAATTCTACAAGTAATACCAATACCCTATCAAGCATTA

ATATTCCACTCTTAATCTAATCTTTTTGCATATTTTATTTATTAATTCCTCAGACTTAGTGCATAATATTTGTGTTAAATTTGTGCAGTATGCAGTTGAT

ACTGTTCCATTTGGAGGAGTGGGGGAAAGTGGGTTTGGCATGTACCATGGGAAATTCTCCTTTGACACATTTAGCCACCAGAAAGCAATTGTAAGAAGAA

GTTTCCTCACTGATTTTTGGTATAGATATCCTCCATGGACACTAAACAAGTTACAACTACTTGAAGTCTCTTACAATTATGATTATCTGGGATTGCTCCT

TGTCCTGTTGGGCTTAAAGAGACCATCAAAACGTTTAATCTCAGATCATGTTTAGCTAGCTAGCTAGCATATATGCACGTACGTGACTTCAGTACTAATT

AAATGTTACCTATAAGGGTATCCAATAAATATTGTTCTTTGTGCAAGTCATCCGTACTTGTGGATTATAACATTTGCGTATTAAATTCTTATGCA

>Glyma.13G340000 | Chr13:43201486..43205317 forward

AAATTAATGCTAAACCATAATTTTCTCCATAGAACGTGTCAGGTGAGGACAATTTATTACGGTTGTTAACCAAATAGACATTTGGTGGTGAAGATTATAT

ATACTACCTAGTCGGAGAGAATTGAAGTAGGGCTGTAGGGGTACATCAAATTCTTTATTTATGAGATGAAGTACACTGGGGAAGCTTTGGGAAGAGATCT

TGAGAACGTGAGGAAGTACTACGGGAGTGGAAAAACAAAGGAAGCATCATGGAGAGAATCACAGCTTAAAGGGTTGCACAATTTTCTCGTTGAAAAAGAG

GAAGAAATTTTGAGGGCCCTCAAGCATGATTTGGGGAAGCATTACGTGGAGGCTTTTAGAGACGAGGTGTGTGTTTGATATTATTTTATGTGAATCATGA

ATATGAATCATCCTAGACTAGTCCTCCTGTTGTTAAATTCTACTTGGGACTTTTGCTACTGTTAGCAAATTGTACACCTCTTTTAATTTTCGTTTCTAAA

TTAATCCATGATGATTAATTACTCGCAGGTTGGAACATTAATGAAGACCTTAAACTTGGCAAGCAAGTCTTTGAAAAATTGGATGGCAGGCAAAGAGGTG

AGGTAGCTCAAAGACCTTGTGAACAAGAATATTTTTTATTTTTTTATTTCCATGACTAACTTTGATGTTTGTCACAGGCAAAGCTGCCACGAATAGCACT

GCTTTCAAGTGCAGAGATAGTTCCTGAGCCACTTGGTCTTGTTCTCATAATCTCATCATGGAACTTTCCCTTTGGTGAGTTAATTCAAAATGAAATGTTT

AGGGTTCTTCTAAAAGTTGCTTTTAGGATCTCTTAAAAAGATCCACGTCGACGAGTGATATAGCTGAATTAAAATATTATAAATAAACAGAATCATTACC

ACCAGATCAAGTAAGTTAATGTTTGGTTTTATAAAACAGGACTTTCCTTGGAGCCACTGATAGGAGCAATAGCTGCTGGAAACAGTGTGGTTCTAAAGCC

TTCAGAGTTGTCCCCTACTTGCTCTTCTCTTTTAGCCACTTTCCTCCCAACTTACTTGGACAATAATGCCATTAAGGTCATCCAAGGTGGACCAGAAGTG

GGGGAGCTACTCTTGCAGCAAAGATGGGACAAGATTTTCTTCACAGGTTAACCTTTCTCAATCATATATAATCTTTTCCATCATCTTCTTATTTAACTCA

AAACCTTTGATGAGATTTTTTTTTAACGTATGACATCAATACTAGGAAGTGCAAGGGTAGGGCGGATTGTTATGTCTGCAGCAGCTGTGCATCTGACGCC

TGTGACTTTGGAGTTAGGTGGAAAATGCCCAGCCATAATTGATTCTCTTTCTTCTTCTTGGGACAAAGAGGTGGATACTCATTCAATCTTGTCACATCAC

ATGGCAAAAAAAGAAAAAATAAAGTTCTATTTTATTTTATTTTGTGTGATCGGGGCCCTTAAAATTAAGTCGATTACATATTTATATAATAATTATATGT

AAACTTGTGGCCCAAAAAAATTGCTAACTGGAAGTTTCTTTTGTTGAATGAACCACAAGGTGGCTGTGAAGCGAATTTTGGTGGCAAAATTTGGGGCTTG

TGGGGGACAGGCATGCATAGCAATCGATTATGTCCTTGTGGAGAAGAGCTTCAGTTCTACATTGGTACGAAGCTTCAAAGAATTTCGACCTTTTGTTATT

TTGATATTGAAGCATGGCTCTACTCTTTTTTTTTTTCTTTTTCTTTAGGTGACATTAATGAAGGAATGGATTAAGAAACTGTTTGGAGAAAACCCCAAGG

TGTCCAACACTATTGCAAGGATAGTTAATAAAAATCATTTCATGAGACTCAAGAATCTTCTCACTGAGCCAAGGGTCAAAGAATCGGTGGTTTATGGTGG

TTCAATGGATGAAAATGACTTGTGAGTATTACTCTCTTTCCTTTTTTTGCTACCAAGTACTCTCTTTTCTTAGTTGCTAGCTTCCAAGTTTTTCCATATG

TCGCATATGATCCTGTGCAGTTCACTTCCCACCTTAATGGGACAGACCAATTTTCTTTCACATTACTATCACCTTATAAATTTTGTACAGATTTATTGAA

CCGACAATATTGCTGGATCCGCCACTTGATTCAGCAATCATGGCTGAAGAAATCTTTGGCCCTGTACTTCCAATTATCACCGTAAGATGCAAAAAAAAAA

TATTTTACCTTTAGTTTCTCATTAATAGTCCGGGAATGTGGCAAGTGAAGTTTTATTTGTATTTTCCTGGCTTTTGCAGGTGGAGAAGATTGAAGAGAGT

GTTGAGTTCATAAGCTCAAGGCCTAAAGCTTTAGCTATTTATGCCTTCACGAAAAACCAGACTCTGCAGAGAAGATTGGTATCTGAAACATCATCCGGAA

GTCTTGTATTCAACGATGCAATTCTACAAGTATTAATCTGCTGCTTGTCTCTTTCTTCTTATTTAATAAGCTTGAGGCATTTTTTTAAAATCTCACATTG

ACTAATGCTAAAACAGAATATATAAGTGGAGTTGAGTTAGATTTTTTGGGTTTAAGTTAGGTCCAAATTTTATAATGATATATAGTAGATCATATTCTTG

TGATTGTTATTGTACTTAGTATATAAGTGGAGGAGGAGATATAAGGTGTGTTAGATGATCAAGGGAGAAGAAAAAATGGAAAAGATTGCGGTTTTAATAT

CATCTACTAATAAAACTTATATTCTAACAAATTAATATCATCTACTAATAAAACTTATATTCTAACAAATTAACATTTGTCGATAAAATATATAAAATAT

ATATATATATATATATATATATATATATATATTAGGAGCAAGAGGGGGAGGTTGAAATTTGGTTCTTTCCATAAGACAACTTTACGTTTGGTTCATTGTA

CCACTTTTCGGTCGCAATTAATCATAAGCACATGCATAGGCTGCTTGTGATCGAATGGTAACTACTAACTAGTCTGTATTTTGTGTAGTACGTAGCGGAT

ACCCTACCATTTGGAGGAGTTGGTGAATGTGGGTTTGGCAAGTACCATGGAAAGTTCTCCTTTGATGCGTTCAGTCACCATAAGGCTGTGGCTAGAAGAA

GTTACTTGACTGATTTTTGGTTCAGGTTCCCTCCATGGACTCTTAATAAGCTCCAACTACTTGAGGTGTCTTACAATTTAGACTACCTTGGAATACTTCT

AGTTCTGCTCGGCTTAAAGAAATCAAAACGGTCACTATTTCAGGCTTGTAATTGAGTTGCATTATGCTGCTTCACTTATCTATTTCAAGTGAGTCCCTAA

TAAGGATTGGTGATTCCAAGCTTGTGTGAATTCCTAGGTTTATGATGCTTAAATAAATGCATATCTGGTTTAGCTTTTGGCATTTGTGATCCACGTCCAA

CCTGAAGCATCTAAATGTTGCTTCTGCATCTTCTTAAACTTTTTATGCGGAAATGGGGAGGAACGCGCTGAAGCAAACACACTATATTTGATTATGTATG

TCTTATATGTCTAGTTAGACTTGGGTAAGGCAAGGTTTGGATGAAGGATGTTTGATTCTACTTCTATTACTATGTACTTTATACTGAAAAGATAATAAAA

GAAGTCAAATTAGTAAATGAATAAGGTGAAAGATTTTGTGGATCGGTGTATACCACTAGATTCAGAGAAGCGATGAGTCACAGAGGTGTGGAACACCAGA

AAAGCATATGACAGCACACAAGCTTCACTAACGAATTCCCTTTTCTTCAATTTGTAATGGTACAATGCAATTTATGCTACCCAACACTAGTACAGTTCAA

AATCAATACCATAGTACAAACTATTAACTATC

>Glyma.15G034400 | Chr15:2740960..2746344 reverse

TAGAATCTTGGTGGTGAAAGTATATATACTACTAGTCGCAGTGAAGTAGCTAGGGGCACATCAAATTCTTTATTTATAACATGGAGTACTCTGTGGAAAC

TTTGGAAAGAGATCTTAAGAACACGAGGAAATACTACGGGAGTGGAAAAACAAAGGAAGCACCATGGAGAGAATCACAGCTTAAAGGGTTGCACAATTTT

CTCGTGGAAAAAGAGGAAGAAATTGTGACGGCCCTCAAGCATGATTTGGGGAAGCATTACGTGGAGGCTTTTAGAGACGAGGTGTGTTTGATTTGATCAT

TTTTATGTGAATAATGAACGTGATGAAATCATCCAAGTCCTGTTGTTAAATTCTACGTGGGATTTAATTTTCGTTTCTAAATTAATCCATGATGATTACT

CGCAGCTTGGAACATTAATGAAGACCTTAAACTTGGCAACCAAGTCTTTGAAAAATTGGATGGCGGGGAAAGAGGTGAGGTTGCTCAAAGACCTTGTGAA

CAAGATTTTTTTAAAATTCCATTACTAACTTCGATGTTTGTCACAGGCAAAGCTGCCACGAATAGCACTGCTCTCAAGTGCAGAAATAGTTCCTGAGCCA

CTTGGTCTTGTTCTCATAATCTCATCATGGAACTTTCCCTTTGGTTAGTTGATTCAAAATGAAAGTTTAAGTTCAAAAGGTTCTTCTAAAAGTAGCATAT

AGGATCTCTTAAAAGATCCCCGTCCACAAGTGATATATATAGTCTAATAATTAAAATATTAAAGTATGTGTTATTAATGGTTTTATAAAACAGGACTTTC

CTTGGAGCCACTGATCGGAGCAGTAGCTGCTGGGAACAGTGTGGTTCTAAAGCCTTCAGAGTTGTCCCCTACTTGCTCTTCTCTTTTAGCCACTTTCCTC

CCAACTTACTTGGACAATAATGCCATTAAGGTCATCCAAGGTGGACCAGAAGTGGGGAAGCTACTCTTGCAGCAAAGATGGGACAAGATTTTCTTCACAG

GTTAACCTTAATTTCTCAATCATAATCCTGACTCTCGGTCATAACTCATAAATCAACCTTTGATGAGATTTTTTTTTAACGTATGATATGATCCAATACT

AGGAAGTGCAAGGGTGGGGCGGATTGTTATGTCTGCAGCAGCTGTGCATCTGACGCCTGTGACTTTGGAGTTAGGTGGAAAATGTCCAGCACTAATTGAC

TCTCTTTCTTCTTCTTGGGACAAAGAGGTAGATATTGTCATTAAAATTTTATTTTATTCACTTGATTATAAAATTTGTTTTCATTTTTTATTTATTTCAT

TAAAATGATTTTTTAATCAAAAAAATATTTCAATTTATTATATTTTTATAATCTTATATATTATCTTTTAAAATATAATATATAGGATTAATTAAAATAT

ACGTACTTATATAAGTTTTATTTTTATTTTACATATTATATTTTTATAATCTTACGTATTTTTATTGTATTATTCAAAACTTATTTTATACAATAAAAAT

ATAATATAAATTTTATCTTTAATGTATATTTTTTATACAAATTATAATTTTATAAAATGACAATATTTTATCATCGTTTAAAATATTTTTATATGTGATT

TAATGACAATATTTTTAACCTATATTAAGATATTCTTGTTATTTATTATAATTATTTTTTATTTATACTTAACTTTATTTAATCAAATATGTAAAATTTT

TAAAGAATTAAATTATATAGTAAAAATAGGTAACTAAATAAAATGATATTATTTTATATATTTTAAAATATTTTATGTATGTGATTTATTAATAGTACTT

TTTTTATCTATATTATAATATGTAACATGAATATTTTCATATAGATAAAAATATTTTCATTAAATCACAAGTATTATTTTAAAAGATAGATATAATATAT

AAAATAAAAATAAAACTTATATAAATATACATATTATAATTTATTTAAAAATAAAATTCAATCTTATATATTATATTTAAAAGAATAATAAATTAAAAAA

GAATAATATTTGAATTTTAAATTATTTTTACATAAAACTTATATTAATATTATATTTTACACTAAATATTCAAATAAATACTTACCATATTAATAAAGGA

ACGTGTTTCTCACACTTTATGATTCTAATCATTCCTTAAACATTTTTAAATTTCTTATTTTTTTTAAAAATATTGATCAAATTTTTAATTTAATCAACAT

TTAAAATTTAACCGTTAACTATCAAAATCAACACTAAGAACTTGGGTAATAAATTTTAAAATACAGCGGAATAAATTTTGTCATTAAGCCTATTTTATTT

TGTGTGGTGGTGGCGGCTTAAAATCAAGTTGATTACATATTTATATAATTATTATATTTAAACAGTAAACTTGTGGCACAAAAAGAATTGCTAACTGGAT

GTTTCTTTTATTGAATGAACCACAAGGTGGCTGTGAAGCGAATTTTGGTGGCAAAATTTGGGTCTTGTGCTGGGCAAGCATGCATTGCAATCGATTATGT

CCTTGTGGAGAAGAGTTTCAGTTCTACATTGGTACGAAGCTTGAAAGATTTTTTGAGCTTTTCTTATTTTGATATTGAAGCATGGCTCCACTCTTTTTAT

TTTATTTAGGTGACACTAATGAAGGAATGGATTAAGAAAATGTTTGGAGAAAACCCCAAAGCGTCCAACAGCATTGCAAGGATAGTTAATAAAAATCATT

TCATGAGACTCCAGAACCTTCTCACTGAGCCAAGGGTCAAAGAATCGGTGGTTTATGGTGGTTCAATGGACGAAAATGACTTGTGAGTAGTACTCTTTTT

CCTCTATTTTTTTGGCTACCAATACTCTCTTTTCTTAGTTGCTAGCTTCCAAGTTGTTTTTCCATTTTTAGCATAGGATCCTGTGAAGTTCACTTCCCAC

CTTAATGGGACAGACCAATTTTCTTTCACATTATTATCTTATCACCTAACAATTTTTGTACAGATTTATTGAACCGACAATATTGCTGGATCCGCCACTT

GATTCAGCAGTCATGGCTGAGGAAATCTTTGGCCCTGTACTTCCAATAATCACCGTAAGATGCATAATTTTATTTTACTTTTTGTTTCGCATTAATAGTC

CGGGAATGTGGCAATTGAAGTTTTATTTGTATTTTCCGGGCTTTTGCAGCTGGAGAAGATTGAAGACAGTGTTGAGTTCATAAGCTCGAGGCCTAAAGCT

TTAGCTATTTATGCCTTCACGAAAAACCAAACTCTGCAGAGAAGAATGGTATCTGAAACATCATCAGGAAGTCTTGTATTCAACGATGCAATTCTACAAG

TATTAATCTGCTGCTTGTCTCTTTCTTTATCTTATTTAATAAAGGCATTTTTTGAAACCTCACATTGACTAGTGATGCTAAAATAGAATATATGAGTGGA

TTTGAGTTAGGTCCAAAATTTATAGTAGTATTAGAGCTTATTCTAGGGATTGTTATTGTACCTAGTATATAAGTGGAGGCATGCTTATTAGAACAAGAAC

AGAAGGGACGGAAATCACATACACCAGATTTGCGGGNNNNNNNNNNNNNNNNNNNNNNNNNNNNNNNNNNNNNNNNNNNNNNNNNNNNNNNNNNNNNNNN

NNNNNNNNNNNNNNNNNNNNNNNNNNNNNNNNNNNNNNNNNNNNNNNNNNNNNNNNNNNNNNNNNNNNNNNNNNNNNNNNNNNNNNNNNNNNNNNNNNNN

NNNNNNNNNNNNNNNNNNNNNNNNNNNNNNNNNNNNNNNNNNNNNNNNNNNNNNNNNNNNNNNNNNNNNNNNNNNNNNNNNNNNNNNNNNNNNNNNNNNN

NNNNNNNNNNNNNNNNNNNNNNNNNNNNNNNNNNNNNNNNNNNNNNNNNNNNNNNNNNNNNNNNNNNNNNNNNNNNNNNNNNNNNNNNNNNNNNNNNNNN

NNNNNNNNNNNNNNNNNNNNNNNNNNNNNNNNNNNNNNNNNNNNNNNNNNNNNNNNNNNNNNNNNNNNNNNNNNNNNNNNNNNNNNNNNNNNNNNNNNNN

NNNNNNNNNNNNNNNNNNNNNNNNNNNNNNNNNNNNNNNNNNNNNNNNNNNNNNNNNNNNNNNNNNNNNNNNNNNNNNNNNNNNNNNNNNNNNNNNNNNN

NNNNNNNNNNNNNNNNNNNNNNNNNNNNNNNNNNNNNNNNNNNNNNNNNNNNNNNNNNNNNNNNNNNNNNNNNNNNNNNNNNNNNNNNNNNNNNNNNNNN

NNNNNNNNNNNNNNNNNNNNNNNNNNNNNNNNNNNNNNNNNNNNNNNNNNNNNNNNNNNNNNNNNNNNNNNNNNNNNNNNNNNNNNNNNNNNNNNNNNNN

NNNNNNNNNNNNNNNNNNNNNNNNNNNNNNNNNNNNNNNNNNNNNNNNNNNNNNNNNNNNNNNNNNNNNNNNNNNNNNNNNNNNNNNNNNNNNNNNNNNN

NNNNNNNNNNNNNNNNNNNNNNNNNNNNNNNNNNNNNNNNNNNNNNNNNNNNNNNNNNNNTGCATTGTACCATTTTTCAGTCGAATAATCATAAGCACAT

GCATTATGCATAGCCTGCTTGTGATTGAATGGTAACTAGTCTATATCTTTGTGTAGTACGTAGCGGATACCCTACCATTTGGAGGAGTTGGTGAATGTGG

GTTTGGCAAGTACCATGGAAAGTTCTCCTTTGATGCGTTCAGTCACCATAAGGCTGTGGCTAGAAGAAGTTACTTGACTGATTTTTGGTTTAGGTTCCCT

CCATGGACTCTTGATAAGCTCCAACTACTTGAGGTGTCTTACAATTTAGACTACCTTGGAATACTTCTAGTTCTGCTCGGCTTAAAGAAATCAAAACGGT

CACTATTTCAGGCTTGTAACTGATGTGCATTATGTTCCTTCACTTATCTATTTCAAGTGAGCCCCTAATAAGGATTGGTGATTCCAAGCTAGTGTTAATT

TCTAGGTTTATGATGCTTAAATAAGTGTGTTCTATTTGATTCTGTATGTGTTATATGCCTAGTTAGACTTGGGTAAGGCAAGGTTTGTATCAAGGATCTT

TGATTCTACTTCTATTACTATTACTTTATACTGAAAATACCACAAAGAAGTAAAATAAATAAAAAATAAATAAGGAGAAGGATTTTGTGGATCCGTATAT

ACCACTAGATTCAGAGAAGAAATCAGCCACAGAGACGTGTGGAACACCAGAAAAGGACATGACAGCACGCAAGCTTCACTAGCAAATGCCCTTTATCTTC

AATTTGTAATGGTACGATGCGATTTATGCTAAGGACTATTCCCAACAGCAATGCAGTTCAAAATCAATACAATAGCACAAGCTATTAACTATCCGGAATT

GCAGACATGTATTAGGTCCACTGTGTTTATTATCTCATGTGTCTAATGATAAGAAATAAATAACAAATAAATTAAATTTTCGATA

>Glyma.16G131700 | Chr16:28678260..28682437 forward

AACCCCAACCAACTTTGCATTTAATGAGACCACCGCACTATAAATATTCATGTCCACATGAGTGGGAAGGTAGCGTTGAGGAAAATAAAAAAGAGAAAGA

AAATAATCAATACGACAACTTTGCAATTTTGGTGGTAGACCAAGGTAGGTCAATTGCCATGGACATTGGTGGTGAAGTGGAAGAGACCGTGAGAGAGTTG

AGGCAATACTTCAAAACTGGAAAAACCAAGAGTGTGACATGGAGGAAAAACCAGCTCACAGCTCTCTTAGACCTTGTTCATGAGAATGAAGATGCTATAT

TTAAGGCCCTCCATCAAGATCTTGGGAAGCACCCTGTTGAGGCTTACCGTGATGAGGTTAATTATCATCAAACAGCACTAAAATTTTGTAGTTTTAATTA

GCCGAAACATAGTCACAAGTTGATTTGCTTTATATACACTTTACACCATATAGCTTGTTTGAATTTTTTGTTTTTATTTTACCCTTTAACTTGTATTGCT

TGATCCTTGACTAGTTGTTTGCTTTTCTTAGGTTGGAGGGGTAGAAAAATCCGCCAGCAATGCTTTGAGCTGCGTTGAGAAATGGATGGCCCCTAAAAAG

GCAGATTATTCTATTTCCCTTTTGTCTTATTAATTTCTCCCTTTCATTTTAAGTACTTTCCTTTTTTGTGTGTGCTTAATTTGACAATAACATATGATCT

GAAGTCTGAACATCACTTTTATCATCGAAAGCTCTAAATCAGAATTGTATGGTATTAAGGTATTAGAATTTTTTGTACTAGAAGTAAATATAATGTTGCA

TAAAGGATTATGGTCGTTATATATATATATATATATATATATATATATATATATATATATATGTTTGAATTGGTTGCTTATGTTTCATTACTTCACTAAT

GTATTTAGATCCTTTCATTTTGACTAACCCGTATAATGATTCATTAACCATAGTTCTTGTTCTATTCACTCTTTTTCAGCTGCTTCTTCAACAATTTTCT

TGTCATGATTTGTGTCTGCTGTTAACCTTTTTCTGTGTTGGGGCTATTATTGCAGAGTGATATCCCTTTTCTTTTCTTCCCAGCTAAAGGAGAAGTGTTG

TCAGAACCACTTGGTGTGGTTCTCATATTTTCTTCTTGGAACTTCCCAATCAGTGAGTCTAAAAATTTTCTATTCAACCCATGTTGTTATGTTTACTTGT

TAGTTACTGTTAGGTTTCAGAATTATTCATGAATCTGTTTCCTAGAGACTTTTTCATGTACATCTCTTCTTTTTTATTGCAAAATCTTGACAAATAATAT

TTTAGTTTTTTTTTTTAAGTCATTGTATGTTTATGGAATTAGCAGTGACCAAAGGTTAAGGTACTGTTATTTATCTCATAAGAATTTTGTGTAACACCAG

TACCAGACCGAATTTTGTCATACTTTGGGGTTTTAAAATTATCACAAAATGTTAATTTATTTACACTATTATGCAGTACTGACATTGGATCCAATAATTG

GGGCAATATCTGCTGGAAATGTTGTAGTCATAAAACCTTCAGAGCAATCTCCAGCAAGCTCTTCTTTTCTTGCCACTACCATTCCTCGCTACTTGGACTC

TAATGCCATCAAGGTGATTGAGGGAGGACCAGATGTCTGTGAACAACTACTGCTACAAAAATGGGACAAAATATTCTTCACTGGTATTATATTCTATACC

AAGCAATGTCATCTTGCAAAATTGTGGTATATATATAATGTATACATATATTTATTGTGTATATCAAAATTAATACATGTCTGGGGGAACATTTGCAAAC

TTGATCTTGGATGAACTTAGACTTGTCAAAATTGACTTTTGCTAAAAGTGAGTTTTAATTTAAGTGATTTTATATTCAGAATATTTTATCTCCAAAAGTT

TTCAGTCAAACCTAACCTGAATATTCAACTCAACCCAACATCAAAGTAAGTTATGCCTTTTTGTAATTTGTAGTGTTTGGACTTGTGTTTGAGACATTCA

AACCAAATCCAAAAATAACATTCATTCGATTAGCTAAACACCATTTCCTGTTACTTTGTGGTTTGTTTATCATCTCATTTAAGAATAAGATATAATCCTA

TAAAGACAACTACAAACTGAATTGTGGTTCTAGAATTATTTCTCCATGGTGTATGAAATTTTCAGGAAGTCCACGTGTGGCAAGCGTAGTCATGTCTGCT

GCTGCAAAGAATTTAACTCCCGTTACTCTAGAGTTGGGTGGAAAATGCCCTGCCATTCTTGATTCCCTTCCAAATCCTTTAGAGTTTAAGGTAGTTCACA

TGAGTTATTAATGTTATGCTTGCCACATTCAATTTCAAGGTCTAATTCAGCTTTCTTTTCTTTGTACATTTGTGGGCATCAAGTTGGCAGTAAAAAGAAT

TGTAGGAGGAAAGTGGGGACCTTGCAGTGGTCAAGCTTGCATAGCAATAGACTATTTGCTTGTTGAGAAGAAGTTCTCATATGCTCTGGTATTCATTACA

ATCACCAGCCGCTTTTTATGATAAAATTTTATTTTGAATAAATGATTATTTTGGTCCTGAATATGTAAACTAGTTATAATTTAGTTCCTGAAATATAATT

CTTTGCTTTAGTTACTGAATGTGAAAAAACTGTAACAATTACGTCCTATCGGTGAGAGTATTTTTTTATTAAGTTATAATACTCAAAGATCAATTTGTCA

TTTAGTTATAAACTTAAAGGATCAATTTGTCTGTCACACTTTTTACATATTCAAGACTAAATTACAATTTTCATTCTTTCATACTAAATAGTCATCCATT

TATACACATTTGAGAACCAAAATGTTCATTTACCCTTTTACTTTTCTAATAGTAAGTTGTTGGTTCTCTTCTAGATAGAATTACTAAAGAAGATCATTAG

AAGATTTTATGGTGAAAATCCAGTGGAGTCAAAGGTAATTTCAAGAATATTAAACAAGCAGCACTTTGAAAGATTATGCAATCTTCTCAAAGACCCTCTT

GTTGCGGCTTCCATCGTTCATGGTGGTTCAGTTGACGAAGAAAACTTGTAAGTTCTGCCTGCAGGGAAATAGAAGTGGTTAGTTAAGTTTATTTGCTTAA

CTCAGCCATATCTAACAATCTTTTCAGGTTCATTGAGCCAACAATTTTGTTAGATCCTCCGTTAGATTCTCAGATAATGTCAGAAGAGATATTTGGCCCA

CTGCTTCCTATAATCACTGTGAGAATCTACAATCCCATTTTATGTTTTGAATTCTCACCTATATTGTGTCATTGTCTCATTCAACTCTTGTTGCACTTGG

CATCTGTGCAGATGGATAAAATTCAGGAAAGTATTGAATTTATCAATGCAAAGCCAAAACCTCTTGCCATCTATGCCTTCACCAAAGATGAAACTTTCAA

GAGAAATATTCTATCAGAAACATCCTCAGGAAGTGTCGTATTTAACGACACAATGGTTCAAGTACTGCTTTTATCTTCCTCGTCTTCCCTAACTTAACTT

TCTTTTAATCAAGTATAATGTAAAGAAATATAGTAACACTTTCATTCTATGTGCAGTTTTTATGTGATACACTACCATTTGGAGGTGTTGGCCAGAGTGG

TTTTGGAAGGTACCATGGGAAGTACTCTTTTGACACTTTCAGCCATGAAAAAGCTGTAATGCATAGAAAGCTGTTCCTTGAAATTGAGCCAAGGTACCCT

CCTTGGAGTAAGTTCAAGCTCGAGTTTATCAGATTGGCATACAGGCTGAACTACTTTGGACTATTACTGCACATGCTGGGCTTGAAAAGATACAAGTAGA

TAAATACCTCAGCACAGTGTTCATCTATTCCTCAAATACACGTTGATTAAAAAAGAATTAAGCTTTATTGTGACTGAATATGAGTTGTAGGAAAATAGGA

GAAAGGGTCCTTATTTGCAGAAGTTGCACATGTGGTGAACTTTAACTTGGCGTTGTAATAAACGAACCTTTGTATCTCTTATTTTTCTTGCTAGTAATAG

GATGTTTTTACTTCTAATGTCCAACAGACCAGACAATGTCAAACATTTTTAACTTCACTTTCACCTGCATTTGTCCACCCGTGTGGCTGAACCATGAATA

AAGTGCCTACAGACGTAAATAATTAATTCAAATAGCACGAGCATGAAAAACGTGAATGTTGGCGGGATAAGGATGCCT

>Glyma.08G163700 | Chr08:12875239..12891802 reverse

AATATTATTTTAGCCGATTCACTTCATCAAGCACAAGTAAACAAAACCACTGGAATTTTTCGACCACCATTGCAAATGGCCGCACTGAACCTTTGCCGCA

TGGCACTTCGATCCTCGAAACTTCTCTATCGCCCTTATAATCTTCTCTCCGTTCAGTTGCAGATGCAGATGCAACCCTCTTCTCCTCCCCTCACTCGAAA

GGCATGCCCCTTTCTGTCTTTTCACTTTTTCACCGTTTTCATTTTCTGACATACCCATTTCCCCACTGCGTCTAAATGTTTGAAATTGAGTGCAAAGTTT

CGATCTTTTAGCCGATTCCCCACGACCCATTTGAGCTTTTTGGGTGTTTGGTGAAACTGTACATGTTTTTCACCCATTTGTTTAAGAGTTTAAGTTTGAT

GTAACACGAATTTTGCACTGTCGTTAGAAATCACCTCCTTCACATGAGTTTAAAGTAATCATTATAAAATTCAATAATTTCAACTATATCGTCCTTGTAA

ATTGCTCTTCACATGAATTTTAAAGTAATTATTGGAAAAGTCAACAATTTTTACTTATATGACAATCCATGATTGAGTGATACTATAGACAACCTTTGAT

TCTTTGTATAAACTGTGGACGACTTGAATGCGATTTCTTATTAGTATTGTTAATGGTTTGATGTAAAAGTTGACATTTGAATGACCAGTTTGATGTAGTT

ATCATTAGAGGACCAAACTAACGATACCTAAGCCTTTCAGGGGCTGCAAAATGGATGTTTATTATTATTAGAGAAATGCGAGATTTGGGATATTCTTGTT

ACTTGTGCATGAGTTGGGTTTGTTTGAATGGCAGATGAGCACGGATGCGCAGAGTATTGCTTCCCAGCTTAATAGTTCTGGATTGTTGAGGACTCAGGGC

CTTATTGCAGGGAAATGGAGTGATGCTTATGATGGTAAAACCATAAAGGTATACTTGCTGTTCTTTTTTCTCTTTTTCTTCTTCCAATGTTTTTATTTTA

GCAAAGTACAATAGAAGAGGGGCAGCATGCACTTGCTGCTTCATGGTCTATTGATGCTGCTGATGATCATAGTCTAAAGATGCAGCCTGTTTTATCACTC

GATGTTTCTTTGTTTTAACAGTGCTGTTGTGTAAGTATCTTATATGTTAAATGCCGTGGCAGGTTTATAATCCAGCAACTGGTGAATCTGTTGTAGATGT

TGCATGCATGGGTGGAAGAGAGACAAACGATGCAATTTCTGCAGCCTATGATGCATATGGATGTAAGAAGCTTCCGAGTTTGATACTGTATCCTATATTT

CAGAATGGAAGCAGAAGTATTGGAGAGGATTAAGATATATTACCATAAATGACTGAATATATATGTCTTGGTGTAAGATACTTAGGGGGTGTTTGGAAGA

GGCTATGAAGAGCTTATTTGAGCTCTTTATGGAACCTAGGGGGTGTATGAAACTTCCCAGTTTAATGCTATGTCTTATATTTTAGAGTAGAAGTAGAAGT

AGCGGAGAGGATTAAGATATATTTCCATAAATGACTGAATATAAGCCTTTGCCTAAGATACTTAGGGGGTGTTTGGAAGAGCCTATAAAGAGCTTATGAC

TCTTATAAGCTCTTTTTAGTTTATTTCAATAAGCTTCTTGGTGAAGTTTATCAAAATAAGCTCTTTTCAACTTATTCCAATAAGTTTTATGGTCAAATTT

ATAACATAAACTCTCATGTCATAAGAGCTTAATGAATATTAGAAAGCGAGGAATGGGCTTAACTCAACCCTACAAAAGTGACTTATAAGGTGAGGATTGC

TATCTACTTATATAGTTTATCTCAGCACTATCTTTAGTCGATGTGGAACTTCAACATGTTCCCTTTTGCCCATGGTTACCCACCATGTGGGACTTTTAAC

ACACCCCCTCTGTCTAGGGGTGACCACAATTTCGGTGGTTTTTTTCAACAATGGATAAGCACCTAATTAAGCTGATCACCCAAACACTCCCTTTAGTGTA

TAATTATTAAATAAGTTGGGTTCTGAAGTTTCTTTTATAACGTCTAGGATAAATTTGCATAAGATGTGATGGTTATGTTGAATAACAATAACAACTAACA

AGTTTGGTGTAAGAAATGAATTATCTCAGGTGACTGGTCATTTTTTTTTTGTTTTGTTTGCTAATGGGATTGCAGACCTGTCAAGAGTAACATCTATTTC

TTTAAAATAAAATATTAACTTCTGTTAAACCAAATTGGAGTAGCTTCCAATTGAAACTGGGTGTAGATGTTAAATAATTAAATGCACTAAGCATTAAAAG

AATTGCTACTTTATAGAGAGTATGGACAGACGACAGAGTTTTAGGTAATATTTGATGAAATGAAATATTAACCCCTCTATATTTTTATGATTATGTTTTA

GGATGACTTGCATAAAGTGAGGGATTAATATATTTCAATGTTCAGTCTGTGGTGGTTATTAATGTAGTTGATTTGTTCTATCGTATTAATCACACCCTTT

CACTCACAATTTCCCATTGCTGATGGATTAAAGTTCACGATTCCTTTTTGGTGCGAAAAATGAGTTAACTAAAAAATTCTGTTATTTGTATTGTTAGACT

ATTCACAATATTATAATGACTAAAATTTCCTGTGTGTAGCTTGGAGCAAAACCACTGCTGCTGAACGAAGCAAATTACTGAGGAAATGGTATAGTTGTTC

TCTGGTGATTGACCCACCTTTTTTGAAACACTTGACTTTTTTTTTTCTAACTTGCATTTGTTTTTAAGTTTTGGGACCGTTATGTTGACGTTAATGTAAT

TCAATTTATTTAGCCACAAATACTGCTTTATCTAGCATTGTTATTTATTAGGACCACTTTGTGTCAATTCAATTCAGAGTTTTTGTACTATATCACTAAA

CAGTAAATGGTTTTTGTTCATTTGATATGCAAACCTGTCATCTCCTTGTAATTGCAACTGTTGCTAGTGCTATGTGGAACTCCTATTACATTCATGTGTT

GACAACATTAGACCCTTGCAACTGCAAATTTTAGATGGAAAGGAAGATAATTAAATGCATTAGACAGGTTTAATATAATTGGATCTTTTAATTTATGAGG

AAATTTATGATGCTGAAAACAAAGGTAGATCAGAAATTTATAGTTATATCATGGAAGTTGCTTGAATTGTAATTTTCTCCTGATAATAGTTACATCCTAT

TAATTTTCTCTTCAATTTTCAAGGTATGATTTGCTAATGGTACATAAAGAAGAACTTGCACAGCTTATAACCTTGGAGCAGGGGAAACCTCTTAAAGAGT

CTGTGGGTGAGGTACACAATGGGTGAAGAGTTATTGTTTCTTTTCTTTCTTATGTATGTTCCCCAGTTGAGTTTCGTTATTGGTGCAGTATATATGTAAA

TGACTATTTTGCAGATAGTCTATGGGGCTGGCTTTATTGAGTTTGCAGCTGAGGAGGCAAAACGTATATATGGGGATATAGTTCCTGCACCTTTTTCTGA

TCGGCGATTATTTGTTCTAAAGCAGGTAAACTTGGTATCTTAAATTTTTATTTCAGCAGTTTTCTGGGATAACTTTATACCTAAAGTGAAGCAATTTTAT

GATGGGATGTCTGTACCTGATGTAATGTGCTAAATGAAGTGGAGTATGGTATTTTTGACATTAAAAATTATATCAAAGAATTAGGTTTGCGTTTAACATA

TCAAATAATTTTTTAAACTATAACTCTTTCTCATCTGACAACTTAAAGTATATTGCAACGGGTGTACATGACCTGCATTTCTGCAAGGTGATGTGAATGT

TTGTGATGTTAATTACTCTAATCATGCCTCGCAAGTATGGTGGTTGTTGATGTTTAATAACATTGGAAAATCAAACTCAAACTACTATAAGGGAAAGTAG

TGAATCAGAGGAGGCAAAAATAAAAGAATTGTTTGTTTGATTGGCATGCTATCTTGAATCATATTCTTTAATTTATATTCTTTTGGTGGTCTAAGGATGT

TAGTTTTTGATGATTTGAATTGGTTACAACAATGGTCAATATAGCTTTTCATGGAAGGTCAACACATTTTATATGGCATTGAAGTTTGGGATTGCTTTGT

TTGTTGCTAACTTGCTATTATTTACAATGATTTCGCATTTGCTTTCTTTATTACTGCAATTAGTCTAAATTATTAGTTTCTACTTTTGATTTGCCTTTTA

AATGCTCTACAGCAACTTGGAATATTTCATTTTTCTAGCTTCAGGGGCTTTGGCAGAACTAAGAAGGGTGAGGCTTATAGGATTATGGGATTCTGCTATT

TATGCCACTCTATGGTGTATTTGGCTGATGTGAAATGCCTATATCTTTAAGTATTCTTCTTTGCTTAAATCTGACATGGGACATAATCATTTTGATGGCC

TCTATTTGGTATTTGGGTCATGGTCTCTTTATGGGAGTGTCTCCTTAACTGATACATAATGGGATTGAGAGCTTTATAGTTTATTGAATTGCTATCTTTA

TTTGTTTCCCGGTTTATTTAATTGCTTAGCTCTCTTTCATCCCTCTATTTTCCGGAAAAGAAAAAAAAAACAGCTTGGTTAAATAGGAAATGGCACTCAT

CTGCAATTGAATTTTATTGATGTGAAATATGGCTACTAAATAAAGCCAAAATATGGCAATGTCCAAGTACTGCAGAATTTTTTATCTGAGGTCATATTAA

AAGAAATGAGATTAGTTTCTGATGAATTGAGCTAGATATTTATATCTTGAGTAATTGAAAACTATATTCTTGTGAATGTTGAGGAGGCACTTTCCTCAGC

AAATGAAAAGTAAATTTCTGTCTTGATATCCAATGATTTAGTCTGTGTGAATGCTGCTAGAAATGATGGTTTCAATTGCCTTCAATATTGGAAGTTCTAG

AATATGATTTTACTGATTTTAATATTCAATTTCTTCTTCATAAATCATAATCCTAATATTTATCTACATGGCTCGTATTTACCTCTTTGGGATTTGCAGC

CTGTAGGGGTTGTAGGTGCAATTACACCTTGGAACTTTCCCCTTGCTATGATTACTCGAAAGGTATCTTTAGTTTTGAATCTTCACTGTCCTAAATTTAC

CCATTTTCACTTTAAGTGTTTTTAGGAGTCAGGGAATAACAATCATTTTAAGAATTATTTGTTTTCTAATATATCAGGTTGGTCCAGCCCTTGCATGTGG

CTGTACAGTGGTCATAAAGCCCTCTGAACTTACACCTCTCACTGCTTTAGCTGCAGTGGAACTCTCCATTCAAGCTGGAATACCACCGGTAGTTTCTTGA

ACATACTTTTATCACTGCAATGAAAGTTCATTGCATATTTGGCTTACAGTTTCCATCATTTTCGGTTGTGATGTTTTGTAATTTAATAATTAAATTTATT

AAATATTGTCATTGTAGTATGGCAATTGTACATCAACAGCTTGGTTGTGATGTTTATAGAAAGTTCATTCTGATATTTTGGTATGTAATCTACTACGTGT

TTATGTCAAAAACTATGTTTGAACATATGCAGTTATTTTGGTTTCTTTGAATGTGTCTTTTAGAAACTTTAATGCTTTACTATAATATTTTGGAACTGTA

CAATGTACATATTATACATGTGAAGAGAAATACATATTGTCTTAATCATCATTAGTTTATTCCACAATATTGTGTAAATCTTACACCCACTATGAACCCA

AAGTCTGTAGTTTACCCATCTCTTATGTTGCTACATGTTTGCTGACCTTTTCTCTGCTTTTTCTTCTTTCATTCTTTTCCCATAAATGAAGCTTCCTTTT

CTTTTTTTCCCCCTTCCGGCCTTTCTTTTATGATTTTAATGGTTGTGATGTTTAATGACATAGGGTGTCGTGAACGTGGTAATGGGAAATGCTCCTGATA

TTGGGGACGCTTTACTTGCAAGTCCACAGGTTCTGTTACCATGTTATTTTACGAAGCAATTACCTTGTGAATGTAATTGAGCGCCTGAAAAAGAAATTAT

CAACCTTATTTAGGTAAGGAAGATCACATTCACAGGCTCGACAGCAGTTGGGAAGAAATTGATGGCTGGTTCAGCTGAAACAGTTAAAAAAGTATGCTTA

GATATTGTCATGAATTTCTTCAAATACAAGATTAGAAGGATTTTTTTTACCATTATATATATGCCTTTTGAACTTCCAGGTATCTCTTGAACTCGGTGGC

AATGCACCTTGCATAGTTTTTGATGATGCTGACCTGGATGTTGCAGTAAAAGGAACTGTAAGTTAGAAAGGACGAGAATGCATGGTGGCTTTAATGATGT

TTTTGTTTAAGATATTGCTTTATATGGATTTGCTGTGTCTGATTTGTTTGCAACTTAATGTATGCAGTGCCTAAGACCTTTTGATGATGCCTCTTAACAG

TAATGGATAATTCTTGTATTCTCCCTGTGGCCTCAAAAATGAATATTAGAAATGCAAGTAATTAGTGCTGTATTGTCATTTCTTTGTGTGGTGGGTGAGC

CCCAGATAGTTTGTTAGATTGAGAAAAAAATATGGGGTTTCTTCAGCTATGAGGAACTTAAATTGCACAGGGAAAATGCAACTTGGCAGTTGCCTGTGTA

CTTAGGTAAAAAATCGAGCTCTGAAATCAAAATAACTACTCCATATTTCATGGAGAAACAGAAACCTTGATTGCATGTGTCACCAAATTACGTATATGAT

TATTGGTCTTAGCTTGATGACTGGGGCTTGCTGGGCTAAGCAGTTGCAATTTTAGCAGTGGATTACTTGTTCCATGCATGCTAGTATTCTTTTAGATGTT

ACATATCACCTTCACTTAGATTTTTCTTTCTGACTGTTATTTACCGAAGTTTAGGTATATGGATATTTTTTGCTTCAGGTCTTATCTAGCTTGCTGAGGT

GGACTGGTGAAGTTATTTTATTAAACTATCTTTCAGTAGCCTATTGTGATTTTTTCTCCTTGATAAGTAATCTTAATTGATGATAATTTAATATTTATTA

ATTATATGTTTGAGATTTTAATATGTGCTCTCCCTCTCTTTCTCTCTCACTCACACACACACACACACACCAAACGCACAGAACTCCATCAACTTGGGGC

GGAAGCAACTTCCAGTTTAATTTCAGCAATTTTGGAGATTATGGTTTCTGTGCATGAATTCTTTTGTCAATTAGAATCAATGGGTCGATTTGTGGTTGGG

CAATTCGAGCATGGGGTGGTATGATAACAACTAACAAAGCTGCATAAAATGATAGAAGCTTCCAATTTTTGGGTTAATTGCTAAAATGGAAAGTTAAGGG

ACGTGCTGTGATTCTCCCTTCCCCTCTGTTTTGATCATATCATTAATCATAATATTTTATTAATTTGATTGCAGCTTGCAGCAAAGTTCCGTAACAGTGG

ACAAACATGTGTTTGTGCAAATAGAATTATTGTGCAAGAAGGTTAGATGGAAATGATAGTAAAATATCAATTTTACAATTACATTGCATTTATCTGATTT

GCCATCTCTTAAAAACTTTTCATTTGAATATAACATGTTTATGCCCTTCAAAAAAAAATATAACATGTTTATGGTTCTGAATTTATGAATACTTTTAGGA

GAATTTGATACTTTGTTGTTTTCTAATAGCTCCTTCTCCTAAATGGTATGAGTTTCTGGAATGTGTCACACTCACACACTAGCTGTTTCATGTAAAGTGC

TCTTGTATCAGTATTACTGATATAATCAAGATGACATTGTGAATCTTTGCAGGATGCTTACATAGCCTTTTGTAACCTTGTCATTAGGCTTATTGGTTGT

GACTACTGCTGTGAGCAGTTTCTTATTTAGTTTTGATCTCATTATATTATATGGAATTTGTAGTGTTTTGAAAAATAAAAAATTTCAGAATTGAAAACAG

AACACAAGATAACGAATAAAGGGCATTACAATGACATTCTTATGAGATTTTTCTCTGAAGTGTTTGTGTCCTATTGATTGTTTGGCTGTTTGAAGTTCAC

ACCAATGTGAAAATGTGCATTTAGATACATTATTTGTTTAATATTCATTGTTTTTTTTTACTTAACCAAGTTTCTTTGTGAAGGTATATATGAGAAGTTT

GCAAATGCTTTACGTGATGCTGTTCAGAATATGAAAGTTGGGGATGGTTTTAGTGAAGGTGTGTCACAGGTAGTTATTGGTCTTTTTTATTTAAAATAAT

TTATATTAGTTTCTGTTGAACCAACTGCCTGTGGATATTGCCTATCTTGCATCTCTGTTTTTCCAAGGTAATATTCATTCATATGGACTTCAGGTACTTT

TTGATAAATATTTCCTTTCCAATATTCCCGTTATAATATGTTTTTGAGTGTCATTACTCTTTAGAGTCCTCTCCTAAATTGATAGAAAATGAGTCATGGT

GTAACTCATTTCATATCCATCTCTAAACAGGGTCCTCTAATAAATGAAGCTGCTGTAAAAAAGGTTTGTGTTTCTCTTAGTATTTCATTTTCTTTTTGCA

TTGTTGCAGGATATCTTCCAACTAATAGGAACTTCTTGGTTTGCCTTGTGGTTTTTCCTTACAGGTTGAGTCCTTAATTCATGATGCTACATCGAAGGTT

TGTTGTCAATCTTTATTATGACATCCTATTCAATCTCTTGCATTCTAAATAAAGGATCTAAAGAAGTCAAATAAACAAAATTTTGATTATGCAAACTATT

TTGCCATCTATGATCCCTTTTGGGATTTGCTAATGTTTATTAATAGTGCAATTTTCTGATGTATTTTTTCTACTGCGTTGCATGCTTGAGCAACAGCAAG

CCTTATTTCACAGGATGGGGTTGGCTACATCGATCAAACTATTATGTTAACTTGATCAAAAACCAAATTTTCTGAAATTTCTTTTATATCAAGGTCTTTT

ATAATGGTTTCTTGACCCTTTCTTCATTCTTCTATTGCTGTGATATATATATACTTGAACAGAGTAACGTATATTTGAAACGTTTTTTTTTTCAAATGTA

CATTTTCCTTTATTTTTCTGTTTGCAAATATAGCAAGATGGATAAGTAGGTCTAGTAACAAATGGTAATAGTTCTGACTATTCAAATCCTTTTTATCTGA

AGGGAGCAAAAGTAATTCTTGGGGGTAAAAGACATAGCCTTGGACTTACTTTTTATGAACCCACAGTCATCAGTGATGTCAACAGTGATATGCACATATC

AAGGTAAACCTTTTACGACGTTTTCTTGTTTTCTGATTTGCGTCTCTCATTAGACTTTTAAGTTTTGACCAGTAACTTTGACATGATATTTTAATTAATT

TCTATACTAACTGATATTAACAACTATACAGTCTATACTTCACAGCTTGGTTATGTTATAGTATCTTGCGTTTTAAGTTGGAAATTGATGCTGGACATTA

AATTTGGTGCCCATTCACTATATTAGTGAATTTAAACTTCAAAATAAGATTAACATGATTGTTATTGAAAATTCAAAGGTTGTTTTTGAATATACCCAAC

TGCCCATATTGTTAGAAATCTAAGTGTATATATATATATATATATATACACATATATATATATATATAGAGAGAGAGAGAGAGAGAGAAAGTGTGTGTTC

TAATATTCTGGAATGAGAGAATACTTATTACATTGATTAACAAAAGTGAACAGTATTTATATGAGGGTAGGATGCTTGCATTGGAAACTCAGTTGTTTGA

TACAGCTGTCAACAAGCTTAACAGAATTCAAACAGAATCCTAATAAGCTCTAAAGCACTTAACTAACTACTCTAACATTAATGTTCTGTGCTGTGTTTCT

CTTTTTAAATAGTACTAAATGGAAAAAAGTTGCTAATAAGAGGTGATACCCATGCGAATTATCAGAATAGACGTATTGGAATGGTGGTAGTCAGGTCCAT

ATTATAATGGTGATGCTTAAAATTGGAGAAAAGGTTTTGTAAGATAATCAGAGATACAGAGAGTAAGAGAGACTATGTAAAGATTTCCCCCAGCTAGATG

CTTGTGAAATATTGACAGCATATTCTGTCACTGTCATGCTTGCATTCCTTAATCAAACTCCAGCCAGTTACCTAGAATCTCTTCCCAATTAATCACCCTT

GATTATGGGGTAAAAACTTCCTTAATTGTTTCCTAATTCTTGGCAGATTAATTATGGTTTCTCTGTCACTACAGATTAATATATGAACTGTCTTTTCTGC

TGTGCTTTTAAGATAATAAACTGGAGGGAAATTGGATTATGGATATGCTTTTATATACACTAGCAAAAAGATAGGCACTAATGTGCTTCTCTATTCACAT

TTTTTATTTTTTGGAATTTAATTTATTGTATATGTAATTAATACAGTTTTTTTTTTATCAATATAACTGATGTGAGAGTTGAGAATTGAAACAAAATGGG

CATAATTTGCAGTAACAGCTAAAGAGTAAACAAGTTTTGAAAAAGTGGAGACCAAACATCAGAGAGAGGATGTCAGGAGTTTGAATGGGAGAGTGGGGAG

TTACAAATGAAGCAGCTGAAGGATAAATTTGGTAATAAAATGTGTAAACCAAAAAGGGCGTATTGCCTTTATAATTTTTATAGATTATAGATGAGGCTAA

TATCTACAAAATACAAACTGCGATTGAGTTTGGTAGAAACACAACTGTGCAGAACTGTTGAATCTTTTCACTGCACTGTCATTATATCTTCTGTAAAGCT

AGTCTTCACCTCTCTTTGGTGATATGAGGATTCAAACCAATGTAGTTTATATAGTTTACAGTGCCTTGTTGCATGCTTCCTATATTCTATATGTGCAGAA

ATTTTGATATGGTTTATTTCACTTTGCCTTGATATCATCAAATGGAATTTTGTTGTTAACTTTCGCTTGCTGGACTTTTTTTTTTGTTAATTCACCTAAA

TGATTTGGCTTAAACCAGAGAGGAAGCATTTGGACCTGTTGCACCCCTTTTGAGATTCAAAACTGAAGAGGAAGCTATCAGAATTGCTAATGACACTAAC

GCAGGTAGTTCGTTTGGTTCTTCTTTATACAGCCTTGAAGAATGGTTTTTGCCCTCCCTCCCCCTCCTATACAGCCTAGGTTAATAGTTTTGTCATATTT

GGTCACTCTTGGTTTAATGTTGTGAAACTTTGGAACATGGAACTGCCTGTACATATGAAGGTTTGGGTTCATATGTATTTACAAACAGTATCCAACGATC

ATGGCGTGTTGCTGAGGCTCTTGAATATGGACTAGTGGGTGTTAATGAAGGAGTAATTTCAACTGAGGTGCATCTCATTCCTTATTAAATGATATCAAGT

GACAATGGAATTTTTGTTGATGGGTTTATCTACGTGCATGTGTTGATGTAGGTGGCTCCATTTGGTGGTTTTAAACAGTCTGGCCTTGGAAGAGAAGGCT

CCAAATATGGGATGGATGAATATTTAGAGGTAAGGACAAATCTAAAGTATTCATTTCTGTAATTTTCCTGTTACTTAATAAAAGACTGCTTCATAGTCCA

TAAGCATAGCCATTGATTATACTTGTGCAACTTTTGTCCTTTGAAGTTTGTAAAACTGAGTTTGGTTATGGTTTGAAGAATCTAGAAAAGAGGGCATATT

TGTGTAGAATTATGTGTGGTGGGTAGTTAGGGGATTGTGTTGAATTGTTAATAACACTTTTAGATGTTTTGGGTCAATGGGTATGGGCTGATACCTGGCA

TACAAAATAATGTTATTCTTCACTGGGTCTAAGATGTTTACCAAATAAAGTTTTCACAGTAAGATGATTCAGCTATTTCGTATGTAGTATTGGTGGAGGA

TCAGAAAGATTCCTTACCTTGCTTTGTTCAAATGTGAGGAAAAGAGGACAATTTTGTTTTCTAGCAGAAAGATACAAGGACATATATCACATATTATAAG

TTGAATGCTATTAATTATCTTTGAAAAATGTCTAAGAATGAAGGTTCATATGCTAAAATTTAAAATTTTTGCTAAAAATACTGTTTAGGTTTCAGTGTCA

GTATTGTGCAACATTGATATACTTTACATGCACGGTCAATTTGTCATTTCATTTTGATAAACTTTACATTAATGTGTGACATTGCTGAAGTTTGTCCCTA

TTACTTTTCCAGATCAAGTATGTGTGCTTCGGAAACATGAACAAAGAATGAAGTACGAGACAGTGCCAAGCAAAATAGGGAGGAGTCACCTTGCGTTTCT

TAGTTGAAGAATATGCATATAGCTGCACACGTGTTGTATACAAATAAGTGCTGGCTTGCAAAGGGCCTCCTCATTTTTCTGTTTGGTTCTTTTTGCTTTC

CTTTTTCATTCTCTTTATTTTTCACAGATTTTTAGAATATTAAAAAATGCACTGTGAAAGGTACGAGGTTAGTAAAAAAAAGAAAGAAAAGCAATTAGCC

TTTGACCCCTTTGGTGTAACGTTGTTTCATTGTGTTTTTCCAAATCAATGAACTCAAAAATCCAAATTGGATCGGTTTGAATAAAGGTGTTAGATTGTGT

CAAATTTTGAGTTTCATGGTCAAATGTAACTCAAAATAATGGTGTCAAATTTTTGAGCCCTTACATCTTTATTAATAAACTAATGTGCATAATGGTGTCT

ATGTGAATTCAAATCTTTTGAAGGCATCGGGGTTAGGCTTACGCCATCTTATGTTTGAGGATAAACTACTTGTACAATCTACGTTTTGAGTGAAAAAAAA

AATCTTTAAAATTTAAAACAACTATCACAATTAATTGCTTGCAAGCAAGTGTTGCTTAAATGTAAATAAAAATAAAAAAGCCTCTAGAACTAAAACGTGA

GAGTATCTCTAAAAATAAGTAAGCCAAGATAGGTTGCTCCAGGAAGTGATTGATTGCTTGGAAGTATGTTTTTCCCTACTTGAAACTTGAAAGAGATTTT

GAGAGCACATTTAATGAACTGTGATTATGCAAATCAATCTCAACTGGTTGGTGATAATAATAAAACCTAGATTTATATTTGGAATTATCATTTTGCCCAA

AAAGTACGGAAATTTGATGTATAAACTTTTAATAAATAATTACATAATAAATAATAAGTTTAACAAAAAATATTTATTATATTAATTTTATTTGTAAAAC

TACACATATGATCCATTACTAGAAGAAAATTAAACAAATATCAACTCAAAATCATATTTTTCTTATACATTTTAATGTAACATTAATAGGCCACAACATA

ATTTAAACACCAACTAGTCTCCATAAAACAACAAACATTATGATTGATATTTTAAACATCTCTTATCAAAATCTCATCATATTCTATTCATCATAACATA

AAATCTAAACTATAATCACCACATTTGACTCAAACAACATTCTCAACAACATATTCTACTCCATGGAGTTTTCTACATATTCTACATATTTCGCATACTT

TGTGCATCAACTGTACATCTATTGTATTCAAACGACGAAAAGAATATTTTGTGCATCAACTATGCACCCATCATATTTCAACAACAATTAGTGAAATATT

TTGTGTACCAACTGTACATCCATAGTATTCCAAGGATAAAAGAATATGTTGTGCACTAATGAAGTTGAGAGAAAAGAAGAGAGGAGAAAACTGAGATAGA

GAAATCTTGTATTATTCAACCCAATTGAAAAGTTGTTACAAGACAATTTATACAGTTATGCTAACTGTCAACTAACTCTAACAAACTTGAGTCAGTTAAC

ATACAATAGAAAGAGAACTCGCTACTAACTATAGTGATGATCCAGAATACAACAAAAAGAGTAAAAAAACAAAAGGAATAAAGTATACACTTATACTCCC

TCGCAAGCTCAAGAGGTGTTGGAAGAAGGTCTTTCAACCACTTTGAGTTTGGACCTAAAAGAAATGAAGGAAGAAGGAGACAAAGCTTTGGTCAGAACAT

CTGCAATCTGATCCATAGTAGGAACATGAACAACATCAAGCTTCTTGTTCAAAACTTTCTCTCGGACAAAAAACAAATCCAGTTCCATATGCTTGGTTCT

AGAGTGTAAAATAAGATTGTGAGCTAGTGAGACAGTGCTGGTGTTATCACAGAGAATGAGAGGAGTAGAGTGAGCAACTTGAAGTTCAGACAACAAGGAC

TGAATCTAAGTGACCTTTGCTACAATAAGAGTCATGCTTCTGTGTTCTGTCTCAATGGCTGGATCTGGCCACCACAGACTGTTTCTTGGACCACTAACAA

ATGAGATTAGGTCCAAGAAAGGACCTCCTGTAATCAGGATCAGAGGCCCAGTCAGAGCCACAGTAAGCATGAAGAGGAATGGGATGTTGTAGAGATGTTG

CCTGAAAATGTAATCCCCAAGAGATAGTACCCTTGAGGTACCTCAGAATACGCTTAATAGCCAAACAGTGCTGCTCAGAAGACTTGGACATGGAGTGACA

GACCTTGTTAACAGAGAAACAAATCTCAAGTCTAGTAATAGTAGCATATTGCAAAGCCCCAACTATTGACCTATATAAGGTAGAATTAGAGAAATTTGGA

GAGCCAAGCATGGTTAACTTGCAGCCTCCAACCATAGGAGAAGAAATAGGATTAGACTCATCCATTTTAGTCTTGGCCAAGAGATCTCTGATGTATTTTG

ATTGAGTCAGAATGAGGGATCAATCTGGTTGGTTTTGCACTTCTATTTCCAAAAAATAATCTAAATCACCAAGATCTTTGAAAGAGAAGACATAATTGAG

TTGAACAAGTGAGTGAATGAGTTTTGTGTGATGATAATGTCATCTACATAGACTAATATATAGACAACAAAAGCATTTTCTGAAAAAACAAAGAGTGATC

ACACTTGCTGGAATTAAAATTGAAGCTAAGAATAGTAGCTTGAAGTTTGTCAAACCAGGCACTAGATGCCTGCTTAAGACCATAAATAACTTTGTGTAAC

TAGCACACTTATGCTTGTTAGAAGACAAAAATCCTGGGGGTTGGGACATGTATACCTCCTCTTCAAGAATGCCATCGAGGAAGACATTGTTAACATCAAG

CTGCTGAAGGACCAGTTGTTAGAAATGGCAAGAGAGTACAAGTTTAACAATTACTTGCTTTATTACAGGGGAAAAAGTCTTCTTGTAATGAAAGCCTAAC

TGCCGGTGGAAGCCCTTGGCAACAAGCCTAGCTTTGTACATGTTGACTAAACCATCAGGATTCTCTCCTTCACTCTAAACACCCACTTACATCCAATAGC

AGTCTAGAGGGAGGGAGATCAACCAGAGGCCGAGTGGCATTCTTGACTAAAGTTGCATATTCAGTTTGAATGGCTGAGAGCCAGGAGGGATCATTCAAAG

CTGCTTTAGTGGACTTAGGTTCAAGATAGACAAGGAGAAAGGTAGGGTTAATTCTAGATTTTGCACGAGTGCACAGGGGATGGGTATTTGTGGGTCTGAC

TAAGGCAGGAACTATGTCAGGTGAGGGAAGGTTATCGGTTTGAGGTTGGTCAGTATTGGTAGTGAGGGACTGGGTATTAGGATTAATGAATGAAGTGGTT

GGTTGCGGGTTGGAAAAATCTGGAGAAATGAATGGTGAAGATGCAGTATGGCTAGTGACATTAGTGGAAAATGCCGGTTGGGTGGTAGACAAAACAGTGA

GGATTGAGGCAGGGTCAAGAACCTCAGAGGTAATTAAAGATGGTTCAGGAAAAAAAGGTCAGGGTAGGGAAAACTGTTTTCATTTCTGCAGGGTTCATGT

AACAGTTTGTATCTAATCTAGTTTGTTCTAGTTGGGGTAGCCAACTGCGGTTCCTCTCCTGACTCCATTTTAGTCAGTTTTGGAACCAATTCTGTGTTGG

GCATCCCAATATTTGTAATTAAGTACCTCTGGTACTAAATTAATATATATTATATATTTTTGCAGAGCAAAAAAAAAAATAAAAAAATAGAGATATAGAG

TCTACCATCAGCAGCAAGACATTAGTAACCTTTGTCTGAAGGAGAATATACTAAGAACACACATTCTTGAGATCTGAACTGTAGCTTATGCTTGTTGTAG

GGCCTTAGTAAAGGGAAACAAGAGCAACCAAATACCCTTAAAAAGTAGTAACGTGGTTGTTTGTTAAACAACTTTTGATAAGGAACCTCATTACCAATAG

TAGATGAAGGCAATTTGTTGATGAGATAGACACTGAAAATAAAAGCATGATCCTAATAGTGAAGAGGCAGCTTAGCTTGAGACATCAAAGTAAGGCCAAG

CTCTACTATGTGTTTGTGTTTGCGTTCCACTACTCCATTCTGATGATGAGTGTGGGGACAAATGAGTCTAGGTTGAATGCCATGTTCCTCCAAAAACCTA

GTAAAGGGTCTGAACTCCCCTCCCCAATTTTACTGAACAACCTTGATAGAAGCATTAAACTGATTTGAAACCATAGTTTTAGACAGGTTAAAAGTTTCAA

AGGTATCAAATTTATTTTTAAGCAAGTAAAACCAAGTGATGAATATGGTATTAACATCCACAAAAGTAACATAATACCTAAAAGCCAAAGGAGATGGAAA

TGGTGCTGGACCCCAAAGATCGGTAAAAATAAGATCAACAGGTTTTTCATAGACAATGAGAGAA

>Glyma.15G263500 | Chr15:49664672..49682418 forward

ATGCATCCAAATATTATTTATTAATTAAATTACATTATGACATATTGGCACATACACTATAGCTGATTCACTTCATCCAGCGCAAGTAAACAAAAACCAC

TGCAATTTTTCTATTAGGACGACCACCGCAAATGGCAGCATTGAACCTTTGCCGCATGGCACTTCGATCCTCGAAGCTTCTCTCTCGCCCTTATCATCGT

CTTTCTGTTCAGTTGCAGATGCAGATGCAGCCCTCTTCTCCTCCACTCACTCGAAAGGCATGTCCCTTTCTGTCTTCTGAATTTTGCACCGTTTTCATTT

TCTGACATACCCATTTCCCCACTGTGTCTAAATGTTTGAATTTTGATTGCAAAGTTTCAATCTTTTCTAATATTACCACGACCCATTTGAGCCTTTTGTG

TGTTTGGTGATAGTGTACATGTTTTTCACCCATTTGTTCAAGAGTTTAAGTTTGATGTCGCACGAATTTTGCACTGTCCTTAGAAATCACCCCCTTCACA

TGAGTTTAAAGTAATGATTTTAAAATTTAATAATTTTCAACTACATAGTCTCTTGGAATTGCTCTTCATATGACTTTTAGAGTAATTATTTTAAAAGTCA

ACAATTTTTAATTTTATGACAATCCATGATTGAGTGATACTATAAACAACCTTTAATTCTTAGTTAAAAGTGTGGACGACTTGAATGTGATTTCTTTTTA

GTATTGTTAATGGTTTGATGTCGAAAATTGACACCTGAATGACCAGTTTGATGTAGTGATCATTAGAGGACCAAGCGAATGACACTTAAGCCTTTCAGGG

ACTACAAAATGCATGTTTATTATTATTATTTGGGATATTCTTGTTACTTGTGCATGAGTCAGGGGTTTGTTTGAATGGCAGATGAGCATGGATGCACAGA

GTGTTGCTTCTCAGCTTAATAGTTCTGGATTGTTGAGGACTCAGGGCCTTATTGGAGGGAAATGGAGTGATGCTTATGATGGCAAAACCATAAAGGTATA

CATGTTCTTTTTTCTCCTTTTCTTCTTCCAATGATTTTTTAGCAAAGTACAATGGAAGAGGGGCAGCATGCACTTGCTGCTTCATGGTCATTGATGCTGC

TGATGATCATAGTCTAAAGATGCAGCTTGTTTTATAACTAGAAATTTCTTTGTTTTAATAATACTGTTGTCTAAGCATCTTATATATTCAATGCCGTGGC

AGGTTTATAATCCAGCAACTGGTGAATCTATTGTAGATGTTGCATGCATGGGTGGAAGAGAGACAAATGATGCAATTTCTGCAGCCTATGATGCATATGG

ATGTAAGAAACTTCCCAGTTTGATGCTGTATCTTATATTTCAGAATGGAAGCAGAAGTGTAGGAGAGGATTAAGATATAATACCATAAATGACTGTATAT

ATGTCTTTGCGTAAGATACTTAGGGGGTGTTTGGAAGAGCCCATAAAGAGCTTATTTCAGCTCTTTATGAAACTTAGGGGGTGTATGAAACTTCCCAGTT

TAATGCTATATCCTATATTTCAGAGTAGAAGTAGAAGTACCGGGGAGGATTAAGATATATCTCATAAATGACTGAATATATGCCTTTGCCTAAGATACTT

AGAGGGTGTTTGGAAGAGCCTATAAAGAGCTTATGACACTTATAAGCTCTTTTTAGTTTATTTCAATAAGCTTCTTGGTGAAGTTTATCAAAACAAGCTC

TTCTTAACTTATTCCAATAAGTTTTATGGTCAAATTTATAACTTAAACTCTCATGTGATAAGAGCTTAATGAATAAGTACTTAATTAAGTTGATCACCCA

AACACACCCTTTTGTGTATAATTATTAAATAAGTTGGGTACTGATGTTTCTTTTATAACGTCTAGGATAAATTTGCGTAAGATGCACACATAGTGTGATG

GTTATGTTGAATAACAATAACAACTAACAAGTTTGGTGTAAGAAATGAATTATCTCCAGTGACTGGTCATTCTTTTTGTTTTCTTTTCTAGTGGGCTTGC

AGACCTGTCAAGAGTGACATCTCTTGCTTCATAACAAAATATTAACTTCTGTTAAACAAAGTTGGGGTGGCTTCCAATTGAAACTGGGTGTAGATGTTAA

ATACACTATGCATTAAAAGAATTGCTACTTTATAGAGAGTATGGACAGACGACAGAGTTTTAGGTAACATTTGATGAAATTAAATATTAACCCCTCTTTT

TTTGTGTTATATGTTTTAGGATGACTTGCATAAAGTGAGGGATTAATATATTTCAATGTTCAGTCTATGCTCTGTGGTGGCTATAATGTAGTTGATTTGT

GCTATCGTTTTAATCACACCTTTTCACTCACAATTTCCCTTTGCTGATGGATTAATGTTCATGAGTCCTTTTTGATGCAAAAGATGAATTGACTAAAAAA

ATCTGTTATTTGTATCTTTGGACTATTCACAGTATTATAATAACTAAAATTTCCTGTGTGTAGCATGGAGCAAAACCACTGCCGCTGAACGAAGCAAATT

TCTGAGGAAATGGTATATTTGTTCTCTGGTGATTGACCCACCTTTTTTGAAATACTTGACTTCACTTCTAACATGCATTTGTTTTTAAGTTTTGGGACCA

TTATGTTGATGTTAATATAAATTTGATTTATTTTAGCCACAAATACAGCTTTATCTAGCATTGTTATTTATTAGTACCACTTTTATGGTGACTACTACTG

TAATCAGTACCTCTGGTACTTCTCTATTATATAAATATAATTTATCTTTGCAGATAAAAAAAAAACTTTGTGTCAATTCAATTCATAGAGTTTTTGTACT

GTATCACTAAACAGTTTTTGTTCATTTGATATGCAAACCTGTCATCTCCTTGTAATTGCAACTGTTGCTAGTGCTATGTGGAACTCCTATTACATTCATG

TGTTGCCAATATTGGACGCTTGCAACTGCAAATTTTAGATGGAAAGGAAGTTAATTAAAGGCGTTAGACAGGTTTAATATAATTGGATCTTTTAATTTAT

GATGAAATTTATGATGCTGGAAACAAAGGTAGATCAGAAATTTTATAGTTACATCATGGAAGTTGCCTGAATTGTAATTTTCTCATGATAATAGTTACAT

CCTGTTAATTTTCTCTTCAATTTCCAAGGTATGATTTGCTAATGGTACATAAAGAAGAACTTGCACAGCTTATAACCTTGGAGCAGGGGAAACCTCTTAA

AGAGTCTGTTGGTGAGGTACACAATGGGTGAAGAATTATTGTTTCTTTTCTTTCTTATGTATGTTCCCTAGTTGAATTTGGTTATTGGTGCAGTATATAT

GTAAATGACTATTTTGCAGATAAACTATGGGGCTGGCTTTATTGAGTTTGCAGCTGAGGAGGCAAAACGTATATATGGGGATATAATTCCTGCACCTCTA

TCTGATCGGCGATTATTTGTTCTAAAGCAGGTAAACTTGGTGTCTTAAATTTTTATTTCAGAAGTTTTCTGGGATAACTTTATACCTAAAGTGCAGGAAT

TTCATGATGGGATGTCTGTACCTAATGCAATGTACTAAAAGAAGTGGAGTATGGTATTTTTGGCATTAAAAATCATATCAAAGAATTAGTTTTGCATTTT

AACATTATTTTTTCCTGGTCTACTGTAATAATTTAAACTATGACTCTTTCCCATCTGATAACTTAAATATATAGCAATGGGTGTACATGAGCTGCATTTC

TGCAAGGTGATGAGAATGCCTGTGAAGTTAATTACTCTAATCATACCTCACAAGTATGGTAGTTGTTGATGTTTAATAAGATAGTGGAAACTCAACTCAA

ACTAATATAAGGGAAAAGAGTGAATCAGAGTAGGCAAAAATAAAAGAATGGTTTGTTTGATTGGCATGTTATCTTGAATCATATTCTTTAATTTATCTTC

TTTTGGTGGTCTAAGGATGTTAGTTTTTGATGATTTGAATTGGTTACAACCATGGTCAATATAGCTTTTCATGGAAGGTCAACACATTTTATATGCATTG

AAGTTTGGGGTTGCTTTCTTTGTTGCTAACTTGCTATTATTTACAATGACTTGGCAATTGCTTTCTTTGTTACTGCAATTAGTCTAAATTATTAGTTGCT

ACTTTTGATTTGCTTTTAAAATGCTGTACATCAACTTGGAATATTTTATTTTATTAGCTTCTGGGGCTTGGTAGAACTAAGAAGGGTGAGGCTTATTGGA

TAATGGGATTCTGCTATCTATGCCACTCTATGGTGTATTTGGCTGATGTGAAATGCCTATATCTTTAAGGATTCTTCTCAGCTTAAATCTGACATGGGAC

AGAATCATTTTTATGGCTTCTATTTGGTATTTGGGTTATGGTCTCTTTATGGGAGTCTCCTTAACAGATACATAATGGGATTGAGAGCTTTATTGAATTA

CTGTCTGTATTTATTTCCCTGTTTATTTATTTTCTGGAGAAGAAAAAAAGAACAGTTTGGTTAAGTAGGAAATGGCACTCAACCAGTGTTGTTAAATAGT

GGCCATGGCGCCACCATGGCAGAATGGTGTGGTGGTTTTTTTTTGCCAACCACCATATGCAATTTGTGAAGGGATGCCTGCCATGGTGGTGCCATAAGGC

ACACTGGCGTGTTTATATGGCGGAATTTTGGCCTTCCGTCATCCGCCATCCGCCATTGATATTACTGCACTCAATTGCAATTGAATTTTATTGATGTGAA

ACATGGCTACTAAATAAAGCCAAAATATTGCAATGTCCAAGTACTGCTGAATTTTTTTATCTGAGATCATATTAAAAGAAATAAGATTTGATTCTAATGA

ATTGTAGCTACATATTTATATCTTGAGTAATTGGAAAGAATATTCCTTTGAATGTAGAGGAGGCACTTTCCTCAGCAAATGAAAAGTAAATTCCTGTCTT

GATATCCAATGATTTAGTCTGTGTGAATGCTGCAAGAAATGATGGTTTCAATTGCCTTTAATATTGAAAGTTCTAGAATATGATTTTACTGATTTTAATA

TTCAATTTCTTCTTCATAATCCTAATATTTATATACATGGCTCGTATTTATCTCTTTGGGATTTGCAGCCTGTAGGGGTTGTAGGTGCAATTACACCTTG

GAACTTTCCCCTTGCTATGATTACTCGAAAGGTATCTTTAGTTTTGAATCTTCACTGTTCTAAATTTACCCATTTTCACTTTTTTAAGTGTTTTTAGGAG

TCGGGGAATAACAATCATTTTAGGAGTTATTTGTTTTCTAATATATCAGGTTGGTCCAGCCCTTGCATGTGGCTGTACAGTGGTCATAAAGCCCTCTGAA

CTTACGCCTCTCACCGCTTTAGCTGCAGCGGAACTCTCCATTCAAGCTGGAATACCACCGGTAGTTTCTTGAACATACTTTTATGACTGCAGCGAAAGTT

CATTGCATATTTTTCTGTCGTGATGTTTTGTAATTTAGTAATCAAATTTGTTGAAAATTGTCATTGTAGCATGGCAATTGTACATCAACAGCTTGATTAT

GATGTTTATAGAAAGTTCATTCTGATATTTTGGTATGTAATCTACTATGTGTTTATGTCAAAACTATATTTGAACATATGCAGTTATTTTGGTTTCTCTG

AATGTGTCTTTTAGTAACTTTAATGCTTTACTATAATATTTTGAAACTGTACAATGTACATATTGTACATGTGAAGTAGGTCTTCAGAAGAGAAATACTA

AGTTTGCTCCTAATCATCATTTGTTTCTTCCACAATGTTGTGTAAATATTTCAGCAACTATGAACCCAAAGTCTTTAGTTTACCCATCTCTTATGTTGCT

ACGTGTTTGCAGACCTTTTCTCTGCTTTTTCGTCTTTCATTCTTTTCCCATAAATGAAGCATCCTTTTTTTCCCCCTTCCGGCCCTTCTTTTATGATTTT

AATGGTTATGGTATTTAATGACATAGGGTGTCGTGAATGTGGTAATGGGAAATGCTCCTGATATTGGGGACGCTTTACTTGCAAGTCCACAGGTTCTGTG

ACCATATTATTTTACGAAGCAATTACCTTGTGAATGTAATTGAACATCTGAGAAAGAAATTATCAACCTTATTTAGGTAAGGAAGATCACATTCACAGGC

TCGACGGCTGTTGGGAAGAAATTGATGGCTGGTTCAGCTGAAACAGTTAAAAAAGTATGCTTAGATATTGTTGTGAATTTCTTCAAATACAGGATCTATA

CATCTTGATCTTATATATATATATGCCTTTTGAACTTCCAGGTATCTCTTGAACTCGGCGGCAATGCACCTTGCATAGTTTTTGATGATGCTGACCTGGA

TGTTGCAGTAAAAGGAACTGTAAGTTAGAAAGGACGAGAATGCATGGTGGCTGTAATGATGTTTTTGTTAAAGATATTGCTTTATATGGATTTCCTGTGT

CTGATTTGTTTACAACTTAATCTATGCAGTGCCTAAGACCTTTTGATTATGCCTCTTAACAGTAGTGGATAATTCTTGTATTCTCACTGTGGCCTCAAAA

TGAATATTAGAAATGCAAGTAATTAATGCTGTATTTTTGTTTCTGGTGTGGCGGGTGAGCCCCAGATAGTTTGTGTTAGATTGAGAAAAAGTTATGGGGT

TTCTTCAGCTATGTAGAACTTAAATTGCACAGGGAAAATGCAACTTGGCAGTTGCCCTTGTACTTGGGTAAAAAATAAGCTCTGAACAAAATAACTACTC

CATATTTCATAGAGAAACAGAAACCTTGATTGCATGTGTCACCAATTACCTATATGATTATTGGTCTTAACTTGATGACTGGGGATTGCACAGGGAAAAT

GCAACTTGGCAGTTGCCCTTGTACTTGGGTAAAAAATAAGCTCTGAACAAAATAACTACTCCATATTTCATAGAGAAACAGAAACCTTGATTGCATGTGT

CACCAATTACCTATATGATTATTGGTCTTAACTTGATGACTGGGGATTGCTGGGTATTAGAGTAATATACAGAGAGTTGCAGTATTCAACTGCACTCTCA

ACTAAAGTGAGTTGGTTAGAAGTTAGTTAGGAGTTAGTTAGTTGTTATAACTGACTTTTCTGGTCTATAAATACCAGATTGTACCACACTGTTCAGTGGC

TTAATATCATTTTCTTTTCTCTCTGTTAAATTCTCTCTCGCTCTCAAATATCTCTCTTATCTTCTTTATGATTCTGTTAGAATGCTAATATGGTATCAGA

GCTTGGTGAGATCTTCCATGGCGTCACAACAGAATGCGATCCTCACTTCTTCCTCTTTTCCTAACTCCATTTCTCACAAATTTGATGATTCAACCTTTAT

TCTCTGGCATCAACAAGTTGAGCCAGTTAATAAATCCCATCGCCTTCAGCATTTTGTCGTTAATCCACAAATTCCGCTACGATTTCTTTCTAAAGAAGAT

CGTGAAGCAGGGATTGAAAATCCTTAGTATGAAATGTGGGAGCAACAGGATCAGGTTCTTCTTACGTGGCTGCAATCGACACTTTATACATTGATTCTTT

CTTCAAGTCAAGCTTACTAAAACAGGTTCAGATTTGTTTTCTGATCCTACTTTGTATAGGTCAGTGGTGGGTGCTTTGCAGTACTTAACCATCACTAGAC

CAGAGATAAGCCTTGCTGTAAACAAAGTTTATCAATTTATCCATTTGGACACTCACTGGATGGCAGTAAAGAGGATTCTAAGGCATTTGAAAGGAATAGT

TCTTCATGGTCAACATCTAAAGCCTGCTGTTTTAGGACTTCCCTACTCCATTAGGGCTCTTTGTGATGCAGACTGGGCATTTGATGTCGATGATAGGATC

TACCTCTTGTGCAGTTGTATATTTTTGTCCTAATCTCATTTCTTGGTGGTCTTGCAAGCAGCAAGTTGTTGTCAGATCAAGTATATGTTAGATTTCTACT

TAAAACCAATTAGCACCAACTGAAGTTGTCCAACAAATATATAAGCTGCACCTCAAGAACTGAGGTAGGCGATGTGGAACTTCCTAACACCCCCCTCCAC

AGATGCTTGCCAATACAGGCGAGCCTACTAGAATAGGCAAGAACTAAGATGAGCTATAGGCTCTGATACCATGTTAGATTTCAACTTAAAACCAATTGGT

ACTAAGTGAAGTTGCCTAACAGATATATAAGCTGCACTCCAAGAACTGAGGCAGACGATGTGGAACTTCCTAACAGTATAGAGGCAGAGTATAGACGTCT

CGCTCAAACTGCTGCAGAAATATCCTGGATCCATACTTTATTGACTGAATTGAGAGTCTCTTTCAGAACTCCTATTGTCTTTTGTGACAATAAAAGTGCT

GTTGCCATTGCTCATAATCCAGTACTTCATGCAAGAACAAAGCATATGGAAATTTCTTTGTTCGTGAGAAAGTTCTGACCAAACAGCTAGTTGTTCATCA

TGTTCCTGCTCTTGATCAGTGGTAGATGCTCTCATCAAGCCTCTTTCTCCGAATATATTTGGTTTTCTAAGAGGCAAACTCAATGTAATTGAGACTCCAT

CAAAGTGTCAACTACCTTGAGTTTGAGGGGGGGTATTATAGTATTGTAAGAGTTGCAGTCATTCAACTGCCCTCTCTGTATTAGTTACACTAGTTAGTTC

TAGTGAGTTAGTTTACCAAGTTAGTGTTAGTTAGTGACAGCTGTTGACAGCTGTCATAACTAACTGTTTAGTCTATAAATACTAAACTATAACCTTGAGT

TCAGTGGCTAATAAAATTTCCCTTTCACTTTTCTACAGTTTTTTAGTTCTTTTAAATATCTTCTCTTTAGAATTCTATTTGGGTGGTATGGTAACAACTA

ACAGAACTGCATAAAATGATAAAAATGGAACAGAAGCTTCCAATTTTTGGGTTAATTGCTAAAATTGAAAGTTAAGGGGTGTGCTGTGATTCTCCCTTCC

CCTCTGTTATGATGATATCATTAATCATAACATTTCATTAATTTGGTTGCAGCTTGCAGCAAAGTTCCGTAACAGTGGACAAACATGTGTTTGTGCAAAT

AGAATTATTGTGCAAGAAGGTTAGAGATGAAAATGACAGTAAAATATCGATTTTATGATTACATTGCATTTATCTGATTTGCCATCTCTTAAAAACTTTA

TATTTGAATATAGCATGTTTATGGTTCCAAATTTAGGAATACTTTTAGGAGAATTTGATACTTTTGTTGTTTTCTAATAGCTCCTTCTCCTAAATGGACG

AGTTTCCGGAATGTGTCACACTCACATTCTAGCTGTTTCCTTAAAGTGCTTTTTTATCAGTATTGCTGATATAATCAAGATGACATTGTGAATCTGAGCA

GGATTTTTACATAGCCTTTTGTGACCTTGTCATTTGGCTTATTGGTTGTGACTACTGCAGTGAGTAGTTTCTTATTTAGTTTTGACCTTGTGATATGATA

TGGTATTTATAGTGTTTTGAAAAAAATATTTCAGAAATGAAAACAGGAGAACACGAGATAAAGAATAAAGAGCATTGAAGTGACATTCTTATATTAGATT

TTTCTCTGAAGAGTAGTGTTTGTGTTCGGCTGTTTGAAGTTCACACCAATGTGAAAATGTGCATTTAGATACATTCTTTGTTTAATATTAATTTTTTTTT

ACTTAACCAAGTTTCTTGGTGAAGGTATATATGAGAAGTTTGCAAATGCTTTACGTGATACTGTTCAGAATATGAAAGTTGGGGATGGTTTCAGTGAAGG

TGTGGCACAGGTAGTTATTGGTCTTTGTTTTTTTTTTTTTTTTTTTTAAATTTATGAGTTTCTGTTTTTCTATTGAACCAACTGCCTATGGATATTGTCT

ATCTTGCATCTCCGTTTTTCCAACTTAATGGGGGTAATATTCATTCATATAGACTTAAGGTATTTTTTGATAAATATTTCCTTTCCAATATTCCCGTTAT

AATATGTTGTGTGCCATTAGAGTCCTCTCCTAAATTGAGAGAAAATGAGTCATGGTGTAACTGATTTCATATCCATCTCTAAACAGGGTCCTCTAATCAA

TGAAGCTGCTGTCAAAAAGGTTTGTGTTTCTCTTAGTATTTCATTTTCTTTATGCATTTTTGCAGGGTGTGTTCCAACTAAAAGGAACTTCTTGGTTTGC

TTTGCGGTTTTTTCTTACAGGTTGAGTCCTTAATTCATGATGCTACATCAAAGGTTTGTTGCTAATCTCTATTATGACATCCTATTCAATGTCTTGCATT

CTAAATAAAGGATCTATGGGAATTGAATAAGCAAAATTTTGATTGTGCAAACTATTTTGCCATCTATGATCCCTTTTGGCATTGCTAATGTTTATTAATA

GTGCAATTTTCTGATGCATTTTTTCTACTGCATTGTATGCTTGAGCAACAGCGAGCCTTATTCCACAGGATGGGGTTGGCTACATGGACCAAACTATTCT

GTTAACTTTATCAAAACCAAAATTTCAGAAATTTCTTTTATATCAACATCTTTTGTAATGGTTTCTTGACCTTTTCTTCATTCTTCTATTGCTGTGATGT

ATATACTTGAACAGAGAAACTTACATTTGAAATGATTTTTTGTTTTTTATTCTCTAATGTACATTTTCCTTTATTTTTCTGTTTGGAAAATATAGCAAGA

TGAATAAGTATGTCTAGTAACAAATAGTAATAGTTCTGACTATTCAAATCCTTTTTATCTGAAGGGGGCAAAAGTAATTCTTGGGGGTAAAAGACATAGC

CTTGGATTTACTTTTTATGAACCCACGGTCATCAGTGATGTCAACAGTGATATGCGCATATCAAGGTAAACCTTTTACGATGTTATCTAGTTTTCTGATT

TGCGTCTCTCATAAGACTTTTAAGTTTTGACCTGTAACTTTGACATGATAGTTTAATTAATTTCTAGACTAACTGATATTAATAACTATACTGTCTATAC

TTCACAGCTTGGTTATGTTATATTGCGTTGTAAGTTGGAAGTTGATGCTGCACATTAAATTTGGTGTCCATGCATTATATTAGTGAATTCAAACTTCAAA

ATACGATTAATATGATTGTTATTGGAAATTCAAAGGTTGTTTTTGAATATGCCCAGCTGCCAATATTGTTATAAATCTAAGTGTAGATATATATATAGAG

TGAAAGTGTGTTCTAATATTCTGGAATGAGAGAATACTTTTCATTACATTGATTAACAGAAGTGAACACTATTTATATGAGAGTAGGATGCTTGCAGTGG

AAGCCCAGTTGTTTGATACAGCTGTCAACAAGCTTAACAGAATTCAAACAGAATCCTAATGAGCTCTAAAGCACTAAACTAACTACTCTAACATGGATGT

TGTGTGCTGTGTTTCTGTTTTTAAATAGTACTAAATGGAAAAGTTGCTAATAAGAGGTGACACCCATGCGAATTATCAGCATAGACATATTGGAATGGGG

GTTGTCAGGTCCATATTATAATGGCGATGCTGAAAATTGGAGAAAAGTTTTGTAAGATAATCAGAGATTCAGAATAAGAGAGACTATGTAAAAATTTCCC

CCAGCTAGATGCTTGTGAAATATTGACAGCATATTCTGTCACTGTCATTCTTGCATTCCTTAATCAAACTCCAGTCAGTCACCTAGAATCTCTTCCCAAT

TAACCACCCTTGATTTTGGGGTAATAAACACTTCCTTAATTGTTTTCTAATTCTTGGCAGATCAATTATGGTTTCTCTGTCACTACAGATTAATTTATAG

ACTGTCTTTTCTGTGTTTTTAAGATAATAAACTGGAGGCAAATTGGGTTATGCATTATTGGAGTGCTGTAAAGGATCTGCAGTTAGTCAACTGCAGTCCT

AGGTAGTTAGTTTACCCGAGTTAAGTTTGCTCAGTTAGTTAGAGTTAGTTAGCTGTCCTAACTAACTAGCCTATAAATATAGAACTGTAAGCAGTGAATC

TTGTGGTTTATGAAACTAGTTTTCTCCCCTTGATCATTTCTCTGTTATCTTTTCTATTATGGTATCCAGAGCCTAGTTCGATCGAAGTGATCAACAATGG

CAGAATCAAACAAATATTTTCCCTCCTCATCTGCATCCCTTTCTTCCTCTTCTGCTTTCCCCAATTCGATTGGTGAAAAGCTTGATGATTCCAATTTTCT

GTTATGGCGACAGCAGATTGAACCGGTGATCAAATCACACCGGTTTCAATGTTTTGTTGCAAATCCTGTGATCCCTCTGCGATTCCTCAAGGATGTCCAT

CGCAAAATTGGAAAAGAAAATCTGGCGTATGAGGTGTGGGAGCAATAGGATCAAGTTCTTCTCACATGACTGCAATCGACGCTCTCAAAGTCGATTTTGT

CGTGCTGCTTGGGGATCATACATTCATATCAAGTCTGGGAGAAGATTTGTGAATATTTCAACAAACAAACATGAGTTCAAGCAAGGCAACTGCGCACTGA

GTTGCGTTCTACGATGCTGAAGGATAAATCGGTGCGTGTGTTTCTTCTCAAAGTTCAAGAGATCGCTGATTCTCTTGCTTCAGTTGGAAGTCTGATATCT

CCTCAAGAGCATATGACTGCTTGAATGTCTTCCCCGCGGTTATAGTTCTGTCATTTCTGTAATTGAAAGCAAGTTTGAGCCGCTTCCAATCACAAAAGTT

GAGGCTCTTCTCTTAGCTCATGAAGCTCGACTTCACAAGTTTGATGAGGTTTCAGATTAGGGTTTGGTTGAAAATTCTGATCATACCAATAGTGTTAATG

AGAAGCAGTATGTCCATACTTGAAGCAAACTTGACACTGAAAGTTTGCAAAGCGACCACCGTGACCGCGTGCACCACTGTGATTGTATCTACCGCCATGG

CCAAAACCACCACGAGGTGCAAAATTTTCCGGTTCTGACTGCTGAAACTTATTTTAAGCAGAATGCGTATCAGCTCCGTACGTATGAGTCAGATTGACAG

ACGGTGGATCTGGCAACTCATCAAACTTGCGAAGCTGAGCTTCATGAGCTAAGAGAAGCGCCTCAACCTCTGCAATTGGAAGAGGTTCTAATTTACTCTC

ATTGATAGAGATGACCAAACTGTAACCTCAAGGAAGGCCTTCGATCAAAGCATCCACATTTTCTTGAGGAGAGATCGGACTTCCAACTGAAGCTAGAGTA

TTCAAGATCTCCTGAACTTTGAGAAGAAAAGCACGCATCGATCGATCCTTCAGCAAGGTGGATTGCAACTCAGAATGCAGTTGCCATGCTTGAGCTCTGG

TTTGTTTGTTGAAGTACTTGTGAATCTTGTCCCATACTTGATACGTTTGGATACTTACCAAACATCACAACAAAATCGACTTCGAGAGCGTCAATTGCAG

CCACGCGAGAAGGATTTGATCATGTTGCTCCCATATCTCATATGCCGGATTCTCATTTCCGATTTCACGATCAGCATCCGAGAGAAACTGCGGCGGAATC

ACAGGATTCACGACGAAACATTGAAGCCGGTGTGATTTGATCACCAGATCAATCTGCTGTCGCCACAGCAGAAAATTGGAGTCATCAAGTTTCTCACTAA

TCGAATTAGGGAATGTGGAAGAGGAGGTAAAGAGATTGTGTGATTCTGTCATTGCTGATCACTTGGATCGAACTAAGCTCTGATACCATAACAAAAAAGA

TAACAGAAAAATCAGTTTCATTAACCCAATGATTCAATGCTTACAACCCTGTATTTATAGACTAGTTAGTTAGGACCTCTAACTAACTGAGTAACTTACT

CTTAACTAACTAAGCAAACCTAACTCATGTTAGACTAACTACCCAGGACTGCAGTAGAGTAACTGCAGGTCCTTTAATATACTCCAATAAGTGGCTAAGG

GTTTCCATCAAGTTCAGGGTTTTGACTTCAATGAAACATTTTCTCCAGTCATAAAACCTGTCACTATTAGACTAATCATTACTCTAGCATTAACTCATCA

TTGGGATCTCTTTCAATTAGATGTCAGCAATGCTTTTCTCAATGGTACTCTTGAAGAATTTGTCTATATGTCACAGCCACCTGGTTTTGAAAACTCAATT

GCTCTCTGGTACATAACTAAAGAAGGCTCTTTATGAGCTCAAACAAGCCCCTAGACAGTGGTTTGACAAACTTCAGTCTATTCTTCTGCAGTACAGTTTC

ACAGCAAGCGTGATCCTTCCTTGTTTGTTTTTAAAGCTCAACATCAAACTGTTCATCTTCTGGTATATGTTGATGACATAATAATTACTGGTAGCTCTTC

TGTGCTGATTCAGAAACTCACTTCTAGACTCAATTCCAGCTTTGCTCTTAGCAACTTGGAAAATTGGACTATTTTTTGGGAATTGAGGTCAAAACTCTGC

CTGATAACTCTCTTGTTCTCACTCAGAGCAAGTATGTCATAGACCTTCTTCAGAAGACTAAAATGACAGAAGTTCAGCCTATTTCATCACCCATGACAAC

TACCTGCAAGTTGTCCAAGTCTGGTTCTGACTTATTTTCTGATCCTACCCTTTATAGGTCAATTGTGGGTGTCTTACAGTACACTACGATTACTCGACCA

GAAATAAGCTTTGCAGTTAACAAGGTCTGCCAATTCATGTCTAATCCTCTGGACACTCACTGGACAGCAGTCAAGAGAATTCTAAGATAGCTAAAAGGGA

CCCCCTCTCATGGACTTCAACTTAGACCAGCTATATCAGATCAACCCATTCCTCTTGGAGGTCTTTGAAATGCTGATTGGGCCTCAGATGTTGATGACAG

AAGGTCAACATCCGGAGCTGCAGTCTATTTAGGACCCAATCTTATTTCCTGGTGGTCTAGAAAACAACAAGTGGTAGCCAGGTCAAGTACTGAAGCTGAA

TACTGAAGTTTAGCACAAGTAACAGCAGAGATTTTATGGATTCAAACTATCTTGTCTGAGTTAGGTGTTTCTTTCAAAACACCAGTGGTGTTTTGTGATA

ACCAAAGTGCTGTAGCACTGGCTCACAATCCGGTGCTTCATACAAAGACAAAACACATGGAAATTGATGTTTTCTTTGTCAGAGAAAGAGTTCTAACTAA

GCAGTTGATTGTTCATCATATTCCTGGTCTTGATCAATGGGTTCATGCTTCAACCAAACCCCTCTCTCCAACCAGGTTTCAGTTTCTAAAAGGCAAACTC

AATGTGATTGATACTTCTCCCAAGTCTAACCACCTTGAGTTTAAAGGGGGGGTATTGGAGTGTTGTAAAGGATTTGCAGTTATTCAACTGCAGTCTTAGG

TAGTTAGTTTACCTGAGTTAAGTTTGCTTAGTTAGTTAGTGTTAGCTGCTCAGTTAGTTAAAGTTAGTTAGCTGTCCTAACTAACTAGTCTATAAATATA

GAACTGTAAGCATTGAATCTTTGGGTTAATGAAACTAATTTTCTCCCCTTGATCATTTCTCTGTTATCTTTTCTATTATGCATGTGCTTTTATATACAAT

AGCAAAAGGATAGGCACTAACATGCTTCTCTATTCACATTTTTTACTTTTTTAAATTTAATTTATTGCGTATGTAATTAATACAGTTTTTTTTGTTATCA

ATATTAACTGATGTGACAGTTGAGAATTGAAACAAAATAGAGACAATTTGCAGTAACAGTTAAAGGTTATAAAGGTTTTGAAAAAGTGGAGACCAAACAT

CATAGAGAGGGTGTCAGGAGCTTGAATGGGAGAGTGGGGAGTTGTAAATGAAGCAGTTGAAGGATAAAATTGGAAGTAAAATGTGTAAAACCAAAAAGGC

ATATTGGCTTTATAATTGTTATAGATGAGGCTAGTCTCTACAAAGTACAAACTGTGTTTGAGTTTGGTAGAAACACAACTATGCAAAACTGTTAAATCTT

TTCACCGCATTTTCATTATATCTTCTGTAAAGCTAGTCTTCAACTGTCTTTGCTGATATGAGTATTCAAACCAATGTAGCTTATATAGTTTATAGTGCTG

TGTTGCATCCTATATTCTATATTTGAAAAAAAATTGCTATGGATTTATTCCACTTTGCCTTGTTATCATCAAATGGAATTTTGTTGTTAATTTTCACTTG

CTAGACTTTTTTCTTTTCTTTCTTTCTTTCTTTCATTCTTGTTTTTTGTTAACTCACCTATATGATTTGTCTTAACTAGAGAGGAAGCATTTGGACCTGT

TGCACCCCTTTTGAGATTCAAAACTGAAGAGGATGCTATCAGAATTGCTAACGACACTAATGCAGGTAGTTCTTGTGGCTCTAGTTTCAAGTTTGATTCT

TCTTTATACAGCCTTGAAGAATAGTTTTTGCCCTCCCCCCTCCCCCTCCTATACAGCCTATGTTAATAGTTTTGTCATATCTAGTCACTCTTGGTTTAAC

GTTGTCAAACTTTGGACCATGGATCTACCTGTACATATGAAGGTTTGGGTTCATACATATTTACAAACAGTATCCAACGATCATGGCGTGTTGCTGAGGC

TCTAGAATATGGACTTGTGGGTGTTAATGAAGGAGTAATTTCAACTGAGGTACATCTCATTCCTTATTAAATGATATCAAATGACAATGGAATTTTTGTT

CATGGGTTTATCTACATGCATGCTTTGATGTAGGTGGCTCCATTCGGTGGTTTTAAACAGTCCGGCCTCGGAAGAGAAGGCTCCAAATATGGGATGGATG

AATATTTAGAGGTAAGGACAAATTTAAAGTCTCCAGTTCTGTAATTTTACTTTAGAAACACAAGATTGCTTCATAGTCTGTAGTCATTGGTTATACTTGT

GCAACTTTTATAAAACTCATTTTGGTTGTGGTTGAGAGGCTAGAAGAGAAAGGGCATATTTATGAAGAATTTTGAGCGTTGTGGACATGTTGGAGACAGT

TTTGAATTGTTGATAGCATTTTTAGGTGTTTTGGGTCAATGTAGATGGATTGATGCTTGGTATATGAAATGATATTATTGTCCTCTGGGTCTAGGATGTT

TACAAGAATGATGGTTGCCCAGTAAGATCATTCAACTTTTTCAAATGTAGTTTTGGTGGAGAATTAGAAAGATTCCTTAACTTCCTTTGTTCAATGTGTG

AGGAAAAGAGGACAACTTCCTTTTATAGCAGAATGAAATAGTGACATACATCACACATTATCAGCCGAATGCTACGTTTAAAAAATGTCTACAATAAAGT

TTCCTATGCTAAAATTTAATTTAATTTAACTTTCCTAATAATACTGTTCAGAGTCAGAGGTGTCTTTGTTATAGTTGGTGTCAGTATTATGTCAAACATG

GATGTGCCTTCTTATGGAGTTCTAAATACTTCATGTGACGTTCTACATTGAATAATGCATGCGCGGTCAATTTGTCATTTCATTTTTGATAAATTTTACA

TTAGAATGTGAACTTGTTGAAGTATATCCCTATTACTTTTTCAGATCAAGTATGTTTGCTTGGGAAACATGCACAAAGCTTGAACAAAGGTATACATGAG

GCAGTGGCTAGCTAGAGGGAAAAGTACTTACCTTCAGTGAGTTTGTTGAAGAATATGCATATAGCTGCACACGTGTTGTATACAAATAAGGGCAGGCTTG

CAAAGGACCTCCTCATTTTTCTGTTTTTCTTTTTCTTTTCATTTTCATTGCTTTCTTTTTATGGTCTTTTAGAATATTAAAAAAAATGCACTATAGAAGG

TACCGCACGAGGTTAACAAAAACTATGAAAATAGCCTTTGACCCTTCTTGGTTTAACGGTGTTTCGTTGGCTACTTAACGTGAAAATTACGTTGAAATGC

TACTTAACGTGATTTTAGATAAAATACTTACATGATTGATCTTACGC

>Glyma.07G183600 | Chr07:35126140..35132751 reverse

CTTCCTAATATTATATTGATACGCAACTGAAACGCAATATGCTATACGATCACTGACAGAGTCAGTGTTTCTGGTTGTTTGCACTTTAGATTCAAACCTG

ATAGGCGTCGTTGTAAAAGGAAAAAAAAAGTTCAGTGTTTTTGTGGTCTCGTATCAGAACTCAGAAGTTATCATCCTTAATATCTTCAAACAAGGTTTTT

CACAGCACATCGATACGAGGATGTTGCGGCTGTCGATTCAACGAGGTAAGTCGTAGTATTCAAGTTTTCTGGAATGCCCACATGCAAGGTATCTGATCAA

TACTTGGAATATTGATTCGGATGAAGAAATTGGAAATGTTTTGATTTTTTTTTTCTATTTTGTTGTTGCAGTGAGGAAGTTGAACTTTCTCAGTCCCCAG

ATCTCTGCTTTGGGAAGGTCCCATTTGTCAACTGCTGCTGAACCATCTTCCAGTAAGAGCAATCCACCGGTAAGCTCAAGCTTTGAGTATTTGCTTTCAT

GGGTATCTGTTAACTACCTCAGTGTTTGAAAAAATCGAAGCTTTATTGCCGAAGGCTTTTTTTTTTTTCCAGAGAAAACTGCTATAAAAAAAAATCTTTT

ATGGGTATCTGTTGACTACCTTCGTGTTTGACAAAATCGAAACTTTATTGCTGAAGGTCTTTTTCCTTCCCCAGAGAAACTGCTGTAAAAAAATTGTATA

TATTTCGTTTTTTTTTAGTTTGTTTCATTTTGCTTAAAGGTTGTTGATTTATAATGACTATTTTGTCAAATTGAATGAAAAGGGGGCATCTTTTTGGCTT

ACTTAATGTACACTGGTCTAAAGTTGAAGAGATTTTCTAGCTTTAGGTCCTACATGTAAATAAATATCATGTCATAAGAAATTTTAGAGTGGAACTTGAC

AACAACTCTGAATTGGATTTGACATAATTCAATATATGCTATCCTCGTTGACTTATGAGCATTAAGATAGTGCCTTTCCTGTTAATTAGTTTTTTTAGCC

TCTAATCCCCCCTTCTTTTTTTGTTGGATTCTATCAGAGGGTTCCAAATCTTATTGGAGGCAGTTTTGTTGACTCAAAAGCATCAACTGTCATTGATGTT

ATAAACCCAGTAAGTGTTTTTTTTATACTGCATTTACAGTTTTGTTTCTTTTTAGGGGGTTTTGAAAAATGCAGACTTATAATATTTCTATTATCTGATC

ATAGGCAACCCAAGAAGTTGTTTCACAAGTTCCTTTGTCTACTGATGAAGAGTTTAAGGAAGCTGTATCAGCAGCAAAGAAGGCATTTCCATCATGGCGT

AACACTCCAATTACAACACGCCAACGTGTTATGCTGAAGCTTCAAGAGCTTATACGCAGAGATATGGTAAGCATCTTAGAACATTCTATGACTGTCACAT

TCATTCCAAAACAGAGAAACATCCATACTGAAACACATGTTATTATTGCTTCACCACTTTGTAATTGTTTTAGGATAAACTTGCTCTGAATGTGACCACT

GAACAAGGAAAGACATTGAAGGATGCCCAAGGAGATGTATTTCGTGGTTTAGGTTGGTATTTCTATTATGTTGCTTGAGAAGATGTTGTAATGTGATAAT

AACTATGCTCATAATTTTTTATAATGCTATTCTCTGAATATTTATCTTTGTCATTTAGAGGTGGTGGAACATGCTTGTGGGATGGCAACTCTGCAAATGG

GGGAGTATGTTTCCAATGTATCACATGGAATTGATACTTATAGCATTAGAGAACCACTTGGTGTTTGTGCTGGTATTTGTCCTTTCAACTTTCCTGCAAT

GATTCCCTTGTGGGTAAGCAGTCTTGTATCTTTTGGATGGGTTTTTGGCTAACTAATTAATATTTCAAATTCAGCTCCATTACTTGGTTGGTCATCGTTT

GTGTCAGCATGCATGAGATAGAAAAAGTCTACTTTGTAGAATGATCCAGAAATAATTAGCCTGATTGATTTTCTTATTTCTCACTGCAATGTAATGTACA

TAATATGGATATGGATATGATAACAAATTCTGAATGTCTATCATCTGCAGATGTTTCCTATGGCTATTACCTGTGGTAATACCTTTGTTCTAAAACCATC

AGAGAAAGACCCAGGTAATGTTTTCTATTAATTTTGGTTGTCTATCAATACCCCCTTGAATTATCTCCTCAATCTGGAGCATAATATAACATTATAATGG

AAAAATAGATTCTACTATTGTTTGTCATTCAAAGCAAGCTTAAGGCATTTCAAATCTATCAGCCTAAATTGTACCATGAACAGCTTGAGCTGGAAACTTT

CTGAATAACATGATTGCCTCTAATATAACCTCTTCAGCAAGCGTAAATAATTTTGCGGTCACTAGGCCATTCATCTGTATTGCTATTGATAGCGTCCAAG

AGTTGCATATTTTGAAAATTATATTCAAGGCACAAAGTTGCAAGTTCCAAGAAAGTGTTATTGGCATTAATTTTTTTCACACCTCAGATTCTCCTTGCAT

TTAAATATATAGTTTTAGCTGTGATCTTCAGTTCTAAACTAAAGAATTTTTCAAAAACCTTTTTCTAGGAGCAATATTGTACACGAGTAATCTTTATTCA

ATAGTCATTTTTAATTTTGAATAATAAATGGTAACTCTCCATTTCTAATCTTTTTTAAGCCCTTCCTAAAATTTCCTATATCTTAGTTTCTTTTTTTTCC

AACATAGAATATTTCTGCCATATAATATTTTATTTCATATAATACATAGTGAACAAATAAAAAGAGAGGGGGTTGGGGAATTTGGTCTGTCCAGCCTACA

TTGTACTTGCAATTAATGAATTGCAATTTGTAATTATTTAAATTTAAACGAGGAAGCTGATGCTAATGTAGGAAGAAAAGGGTAAATAAGTTAACACCCA

AGTAACATTGGTGCCTTTTGAAAGAGGCTGTGATTCTGCTTCATGTTGGTCATATTATTTACTGTACTTTGTGTGTTGGGAGAAAATTGTCAGACTTTTA

CAGTTTGTCTGGAAATTCTAAATTAATTTATCATTCATCCACTAGTTTTTCTTTCCTATCCTTGTAATATTATATATGCAAGGTAATACTATATGGCACA

CAAATGTTGGTTCATCAGTTCACTTTTATGGTTCATTATCCTTGGTTTTTAATGACATCATGTAGAGAAATAATTTTCAATGTAGTTAGATGTGGTGATA

TCTCAGCTCCAGACCACCTACTAGGATAAGGCTTGGTTGTTATCTGAAATTCGTTTTTATTTTCCAGGTGCTTCTGTAATGCTTGCAGAATTGGCATTGG

AGGCTGGTTTGCCTGAGGGTGTCTTAAATATAGTTCATGGAACCCATGTATGTCATCACCTGGCTTTTTATTATACTTTTGTTTTTCACCTTTTTCTTGT

GATACTTATCAAAAAAGGTAATGATTGGCTCATTTGGCATTGGTGATTTCATCATAAATTCAAGTTTTAAATCTAAAATTTGCATATAATATTGCACTTG

TACCATACAAAGAATAGTCAATACTATCATAAATTTGATTTTATTCATAAAACTATTTTTCCAAGGGTGACAACTTGTCACATTATGACGTGTCGTGCAT

TAGTTGTTCTTTGTTTCTCCTACTGATTGATTTTCTTAATCATACAACTGGAGCAGGATATTGTGAATGCTATTTGTGATGATGACGACATCAAAGCCAT

ATCCTTTGTTGGTTCAAATGTTGTAAGTCATTTAATACTTGTTTGTTTTCTCCAATTCATGATTTCATGTTATTCTTCTTTTGTGACCTTAAACTAGCCA

AAGGCAATCCTTATACTTGAGAGCAAAATTGAATATTTTTTAGGTTTAACTGTGCTTTAAATGATAGATAGAAGAGGTCTAGTGATCAAATGCTTAGATT

TTGTAAATGATAGACCTCCTATATTACTATATATGCTTATTTGATGTTCTTATTTTTGTATCAAAGGCCGGAATGCACATATATTCAAGAGCAGCAGCTA

AAGGGAAACGCGTTCAGGTAGGTGTGCGTAGTTTTTTTCTCCCCTTGGTAAAATTGTGGGTTTATAACCACGCAAGGATATGAAGTTGGGCAACTAATAT

GCTATTGTTTGCAGTCTAACATGGGAGCCAAAAATCATGCGATTGTCATGGCAGATGCAAATGTTGATGCTACTCTTAATGCTTTAGTTGCTGCTGGTTT

TGGTGCTGCTGGACAAAGGTGTATGGCTCTCAGTACAGTTGTTTTTGTTGGAGGCTCAAAACCATGGTATCATATTATATTCTGTTTGAGTCTTTTGATT

TGATTCATATTTATTATGACTTCTGTAGTAGCATCTTACATTGCAGCAATTCAAAGCTCCTCCTGGTTGTTGTTCCAAAATGATCTGGAATTTTGAATGT

ATTTGATTGTGTGCCTAGTGGAATCATCACTCTTCCCTTACTGTATAATCACAAATTGTATATACTATATAGTTATTGTAATGCCAATATTAGTATGACC

AATTCATTATATTCATCCTTTTTAATAATTCTAAGAACATGGTTCCATGGGAAATACTATTCAATCTTGTACTATTTTTAGCTTCCTTTCCAATATTTGT

ACAGGGAGGATAAACTTTTAGAGCATGCCAAAGCTCTTAAAGTAAATGCCGGAACTGAACCTGATACAGATCTTGGTCCGGTGATCAGCAAGCAGGTTTG

TATTCCATATTTACTTTTTCCACATTGGATTTGAAGTAAAAGCCTGTATCATTCAAGAATCACTGAAAATGGTTATTCAGTGCCATACCATTTCCACTTG

AATTGTTGGGTATATTTTAGCTATTTGTATTTATGTTTCTTGAATATTCTGACTGGCTGCACAGGCAAAGGAGAGAATACACAGATTAGTTCAATCTGGG

GTTGAAAGTGGTGCCAGACTACTGCTTGATGGAAGAAATATAGTGGTAGCTTTTATGTCCTCTTAAAACACTCTGGTGGAATGTAATATTAGCACTCTGC

CTCATACTAATGAATTCATACATGCATGGCAAGTAGGTCCCAGGATATGAATCTGGCAATTTTATTGGTCCAACCATCTTATCAGATATCAATGCCAACA

TGGAGTGCTACAAGGTTACTCACTAGTATTCAATTTTAAATTGTCTTCTAAAAGAATTTCCACTACATAGAGACTGGAATTTTAGATGTTTAGTAGACTT

GACTGTCTCTAATATTGCACATGCAGGAGGAAATTTTTGGCCCAGTTCTTCTTTTCATGGAGGTGTGTCTGTTTGCTTCTTCCTTAGAATTATTATATTA

TTTTATAAGAGTCACAAAATATAATTCTTGGTAAAATGTTTCCTATCTAGGAACAATTAACCCTGGGAAGTGAGCAGCCAGTCACTTGAGGGGTTAAATT

ATTTCTAAATTGAGAATATTTCCACATCAGAGACTTAATTTTATGTTGAATTTTCATTTTGATTCAATAGACTGATATTCTGATACGGTCTTTTTCTTTT

GTCTTTTCAACTTCAGGCTGATAGCTTGGAAGAAGCCATAAACATTATCAACAGTAACAAGTATTTGCTAACAACTCGTTGTCATTGCCTTCTGTTAGAA

TTCTATCTATTCTTACTCTTGAGTTGACTTTTCCAACAGGTACGGAAATGGTGCTTCTATATTTACTACATCTGGTGTTGCTGCAAGGAAATTTCAGACT

GAGATAGAGGCTGGACAGGTAATGATACTTAAGTGAGATGAGTTCTGCATTTGTAAGAGCGTTAGCAACACACTTTTTGTATCTCTTTCCAATACACTAT

CATATTAGTTGAATTCCATGCAAGTTCTACTGGTTTGGAAATGATTAATGAGAGCGACAGACTTAATAAGACTAACATGAATTTCATCCATAAGGATCCA

AAAGAGTTTTAGAGAACATATTACTAGTATTTCTTTTAATAAATATATTTTGATAGTGTTACTCTTATGGATAATGACTTGTGACTGCATTTGAACCAGG

TTGGCATCAATGTCCCCATTCCAGTTCCTTTGCCCTTCTTCTCATTTACCGGCAACAAGGCATCGTTTGCCGGTGATCTCAACTTCTATGGTACAATGAT

TTCTACCAAACCTGGCTCTTCTGCACTTGTTTATCAATTACATTATTTAGGAGTTACATTTTGATTATGATATAGATACCGTCTGATTTGAATTTGATGT

GCTTTGTTGAATTACAGGCAAGGCAGGGGTTAACTTCTATACACAGATTAAGACAATAACACAACAATGGAAGGATTCAACTGGTGGTAGCAAGATTAAC

TTGGCAATGCCAACCTCTCAAAAATAATGTTGATAGCCAGAGCGTGTAATAAGGCTGTTTCTGAAGTCTTTGTTCTCTTGAAAAAAATGTCAAAGGTCAC

CTGCTATGCTTTGTTTTGAAACCTTTCTCTTTTCTTTTTCCTGATCTTATCTGTCTCTTGTGTCTCCACGTGGACAATACAATTCTTTTTGCTTAATTTT

CTAAAGTCAAGGTTTACGTGGAGCCTAATCCATTAATTCCATATCCATCCACATGAAATGGTTTTAATGGAGGAACTTAATTGACCCAAAAATAAGAGAA

TTAATTTTGCAT

>Glyma.08G066600 | Chr08:5106371..5113095 forward

ATTTTCTGCAAAATGAGTTTTACTCTACAAATTACCTTCATGTTCAATTTTTTTAAAAAAATGATCAGCTATGCATTTAAATTAAATACAATGACAGTTT

TATAGAAAGTTATTGAATTAAATGATATTTAATTTTAATAGTATGAGATTGAATCTGATTTGGCATGTCTATCGATTGATTTGTCCTTTCTAGTTCTTCC

CAATATTATATTGATACGCAATATGCTATACGATTACTGACAGAGTCAATGTTTCTGGTTGTGTGCACTTATTAGATTCAAACCTTATTGGCGTCGTAGC

AGAACTCAAAAGTTAACATCCTTAATCTCTTCAAACACGGTTGTTCATAGCATATGGAAACGAGGATGTTGCGGCTTTCGATTCAACGAGGTAAGTCGTA

GTATTACTCGTTTTTTGGAATGCCCACATGCAAGGTTTCTGATCAATACATGGAATGTGGATTCGGATGAAGCAATTAGAAATGTTTTGATTATTTTTTT

TTCTTTTTCTATTTTGGTGTTGCAGTGAGGAAGTTGAACTTTCTGAGGCCTCAGATCTCTGCTTTGGGAAGGTCCCATTTGTCAACTGCTGCTGAACCAT

CTTCCAGTAAGAGCAATCCACCTGTAAGCTCAAGCTTGGAGTATTTGCTTTCATGGATATCTGTTAACTACCTCAGTGTTTGAAAAAATCGAAGCTTTAT

TGCTGAAGGCTCTTTTTTTTTCCAGAGAAACTGATGTAAAAATAAATAAAATTGCTTTTATGGGTATTTGTTAACTACCTTAGTGCTTGAAAAAATCGAA

ACTTTATTCCTGAAGGCTCTTTTTTCCCCAGAGAAACTGCTGTGAAAAAATTATAAATTTTTTTTATTTATTTTGATTTTGTTTCATTTTGCTTAAAGAT

TGTTGACTTATTAAGACTATTTTGTCAAATTGACTGAAAAGGGGTATCCTTTTGGCTTACTTAATCTACAGTGGTCTAAAGTTTAAGAGATTTTCTAGCC

TTAGGTCCTACATTCAAATTTATATCATGTCATAATTCAGGGTGGAACTTGACAGTAAGTCTGAATTGGATTTGACATAATTCAATGTATGCTATTCTCG

TTGACTTATGACTTGTGAGCATTAATAAGTTTTTTTAACATCTAATTCCCCCTTCTTTTTTGTTGGATTCTATCAGAGGGTTCCAAATCTTATTGGGGGC

AGTTTTGTTGACTCAAAAGCATCAACTGTCATTGATGTTATAAACCCGGCAAGTGTTTCTTTTTTGTTTGTTTTATACTGTGTTTACAGTTGTATTTCTT

TTTTGTGGTTTTGAAAAATGCTGAATTATAATATTCCTATAATCTGATCATAGGCAACCCAAGAAGTTGTTTCACAAGTTCCTTTGTCGACGCATGAAGA

GTTTAAGGCAGCTGTATCTGCAGCAAAGGAAGCATTTCCATCATGGCGTAACACTCCAATTACAACACGCCAACGTGTTATGTTGAAGCTTCAAGAGCTT

ATACGCAGAGATATGGTAAGCATCTTAGGACATTCAATGGCTGTCACATTCATTCCAAAACAGAGAAACATCCATACTGGAACACATGTTATTATTGCAT

CACCACTTTGTAATTGTTTTAGGATAAACTTGCTTTGAATGTGACCACTGAACAAGGAAAGACATTGAAAGATGCACAAGGAGATGTATTTCGTGGTTTA

GGTTGGTATTTCTATTATGTTGCTTGAGAAGATACTGTAATGCGATAATAATTATGCTCATAATTTTTTATAATGCAATTTTTGTGAATATTTACCTTTG

TCATTTAGAGGTGGTGGAACATGCTTGTGGAATGGCTACTCTACAAATGGGGGAGTATGTTTCCAATGTATCACATGGAATTGATACTTACAGCATTAGA

GAACCACTTGGTGTTTGCGCTGGTATTTGTCCTTTCAACTTTCCTGCAATGATTCCCTTGTGGGTAAGCAGTCTTCTTCTATCTTTTGGATGCCTTTTTG

GCTACCTAATTAATATTTCACATTCACCTCCATTACTTGCATGGTCATCATTCGTATCAGCATGCATGAGATAGAAAAAGTATACTTTGTAGAATGATCC

AGAAATAATTAGCCTGAATAATTTTCTTATTTCTCATAGCAATGTAATGTACATAATAAGGATATGGATATGATAACAAATTCTGAATGTCTATCATTTA

CAGATGTTCCCTATGGCTGTTACCTGCGGTAATACCTTTGTTCTAAAACCATCAGAGAAAGACCCAGGTGATGTTTTCTGTTAATTTTGGTTGTCTATCA

ATACCCTTGAATTATATCGTCATTCTGGAGCATAATATAATGTTATAATGGAAAAATAGACTCTAATATTGTTTGTCATTCAAAGCAAGTTTAAGGCATT

TCAAATCTATCAACCTGACTTGTTCCGTGAACTGGAAATTTTCTCTTGAATTATCTCCTCAATCTGGAGCATAATATAATGTTATAATGGAAAAATAGAC

TCTAATATTGTTTGTCATTCAAAGCAAGCTTAAGGCATTTCTAATCTATCAACCTGAATTGTACCGTGAACACCGTGAGCTGGAAATTTTCTGAATAACA

TAATTGCCTCTAATATAACCTCTTCAGCTAGTGTAAATGATTTTGCAGTCACTAGGCCATTCATCTGTATTACTATTGACAGTGTCCAAGAGTTGCATAT

TTTGAAAATGATATGCAGGGCACAAAGTTGCAAATTCCAAGAAATAGTGTTATTTACATTTAATTTTTCCACACCTCAGTTTCTCATTACATTTAAACTA

AAGAATTTTTCAAAAACCTTTTTCTAGGAGCAATATTGTACACGGGCAATCTTTATTCAATAGTCATTTTTAATTTTGAATAATAAATGGTAACTCTCCA

CTTATGTCTGTCCAGCCTACACTGTACTTGAAATTAATGAATTTTACTTTGTAATTTATTTAAATTTATACAAGGAAGCTGATGACAATGTAGCAAGGAA

AGGGTAAATAAGTTAACACCCAAGTAACATTGGTGTCTTTGACAGAGGCTTTGATTCTGCTTCATGTTGGTCGTATTATTTACTGTAATTTGTGTGTTGG

GAGAAAATTGTCAGACTTTACAGTTTGTCTGGAAATTCTAAATTAAGTTATCATAGTAAGTTAGTAGCTATAGTGTCACTGTCCACCTAGTTATTGAATA

CTGTGTATTTATCATTCATTCATTAGCTTTTCTTTCCTATCCTTGTAATATTATGCAGGGTAGTCCTATATGGCAAACATATGTTGGTCCATCAGTCCAC

TTTTATGGTTCATCATCCTTGGTTTTTTGATGGCATCATGTAGAGAAATAAATTTCAATGTAGTTAGATGTTGTGATAAGGCTTGGTTGTTATCTGAATT

TCATTGTATTTTCCAGGTGCTTCTGTAATGCTTGCAGAATTGGCATTGGAGGCTGGTTTGCCTGAGGGTGTCTTAAATATAGTTCATGGAACCCATGTAT

GTCATCACCTGGTTTTTTTTATTATACTTTTGTTTTTCACCTTTGTGTTGTGATACTTTCCAAAAAAGGTACTCATTGGCTCATTTGGCATTTGGTGATT

TCATCATAAACTCAAGTTTTAAATCTAAAATTTGCTTATAATATTGCACTTGTACCATACAAAGAATGGTTGATACCATCATAAATTTGGTTTTATTCAT

AAAACCATTTTTTCAAGGGTGACAACTTGTCACATTATGATGATGAGTCATGCATTAGTTCTTTGTTTTTCCTACTGTTTGACTTTCTTAATCATATAAC

TGGAGCAGGATATTGTGAATGCTATTTGTGATGATGAAAACATCAAAGCCATATCTTTTGTTGGTTCAAATGTTGTAAGTCATTTAATACTTGTTTGTTT

TCTCCAATTCTTGATGTTATTCTTCTTTGCGACCTTAAACTAGCCAAAGACAATCCTGTTACTTGTCAGCAAAATTTAATATTTGTTAGATTAAACTGTG

CTTAAATGTAAAGATAGATTGAAGAGGTCTAGTGATCAAATGCTTAGATTTTTTAAATGATAGACCCCCTATATTACTATGCTTATTTTGATGTTCTTAT

TTTTGTATCAAAGGCTGGAATGCACATATATTCAAGAGCAGCAGCTAAAGGGAAACGTGTTCAGGTAGGTTGTGTAGTTTTTTTCTCCCCTTGGTAAAAT

TGTTGGTTTATAACCATACAAGGATATGAAGTTGGGCAACTAATATGCTACTGTTTGCAGTCTAACATGGGAGCCAAAAATCATGCGATTGTCATGCCAG

ATGCAAATGTTGATGCTACTCTTAATGCTTTAGTTGCTTCTGGTTTTGGTGCTGCTGGACAAAGGTGTATGGCTCTCAGCACAGTTGTTTTTGTTGGTGG

CTCAAAACCATGGTACCATATTATATTCTGTTTGAGTCTTTTGATTTAATTCATATTTATTATGACTCCTGTAGTAGCATCTTACACTTGGAGCAATTCA

AAGCTCCTCTAGTTGGTTGTTCCAAAATGATCTTGAATTCTGAATGTATTTGATTGTGTGCTAAGTGGAATCATCACTCTTCCCGTACTGTATAATCACA

AATTGTATATACTATATAGTTATTGTAATGCCAATATTAGTATGAGTGTATGACCAATTAATTATATTCATCCTTTTGGATAATTATGAGAACATGGTTC

CAAGGAAATACTATTCAATGTTGTACTATTTTTAGCATCCTTTGCGATATTTGTGCAGGGAGGATAAACTTTTAGAGCGTGCCAAAGCTCTAAAAGTAAA

TGCTGGAACTGAACCTGATACAGACCTTGGTCCGGTGATCAGCAAGCAGGTTTGTTTATGATTCCATATTTAATTTTTCCACATTGGATTTGAAGTAAAT

ACCTTAATATCATTCAAGAATTACTGAAAATGGCTATTTAATGCATCATTTCCACACAAATTGTTGGGTATATTTTAGCTATTCATATTTATGCTTCTTG

AATATTCTTACTGGCTGCACAGGCAAAGGAGAGAATACACAGATTAGTTCAATCTGGGGTTGAAAGTGGTGCCAGACTACTGCTTGATGGAAGAAATATA

GTGGTAGCTGTTATATCCTCTTAAAATTGCCAGACTCTGGTTAAATGTAATATAGCATTCTGCCTCATACTAATGTATTCATACGTGCATGGCTAGTAGG

TCCCAGGATATGAATCTGGCAATTTTATTGGTCCAACCATCTTATCAGATATCAATGCCAACATGGAGTGCTACAAGGTTACTCACTGCTCTCCAATCTT

AATGTGTCTTCTAAAAGAATTCCCACTACACAAGACTTGAACTTTAGATGTTTATTAGAGTTGACTGTCTCTAAAATTACATATGCAGGAGGAAATTTTT

GGTCCAGTTCTTCTTTTCATGGAGGTGTGTCTGTTTCTTCCTTAGAATTATATTATTTCATAAAATGAGTAAAAAATATAATTCTTGGTAAAATATTTCA

TATTTAGGAACAATTAACCCTTCAACTGATTAATGGGGTCCAAACACTGGCAATTGATTAACCAGTCACTTGACGGGTTAAATTATTTCTAAATTGAAAA

TATTTCCACGTCATAGACACAGCTATCTTGAATTTTTCATTCTAACTCAATAGATAGATATGCTGACAGTTTTTTTCTTTCGTCTTTTCAACTTCAGGCT

GATAGCTTGGAAGAAGCCATAAACATTATCAACAGTAACAAGTATTTGTTAACAACTCCTTGTTGTCATTTCCTTATGTTAAAATTCTATCTATTCTTCC

TCTTGAGTTTACTTTTCCAACAGGTACGGAAATGGTGCTTCTATATTTACTACATCCGGTGTTGCTGCGAGGAAATTTCAGACTGAGATAGAGGCTGGAC

AGGTAATGTTACTTAAGTGAGACGTTTCGTTCTTCATTAACAACACATTTGTAACTTTCTTTCCAATACAGTCTCATATTAGTTGAAATTCATGCAAGTC

CTACTAGTTTGGAGATGAGTCCCACATATTTAATGAGACTAACCTGAATTTCATCCGTTAGGAGAGAATGACTTACTAGTATTTCTTTTAATAAATATAT

TTTGATAGTGTTACTCTTATGGATAATAACTCTTCACTGCATTTGAACCAGGTTGGCATCAATGTACCCATTCCAGTTCCTTTGCCCTTCTTCTCATTTA

CCGGCAACAAGGCATCGTTTGCCGGTGATCTCAACTTCTATGGTATAATGATTTCTCCAAACCTGGCTCCTCTGCACTTGTTTATCACATAATTATTTAG

GAGTACATTTGCTCATAATATAATACTGCTTGATTTGAATTTGGCATGCTTTGTTGAATTACAGGCAAAGCAGGGGTTAACTTCTATACACAGATCAAGA

CAATAACACAGCAATGGAAGGATTCAACTGGTGGTAGCAGGATTAACTTGGCAATGCCAACCTCTCAAAAATAATGTTGATAGCCAGAGCGTGTAATATA

TATAAATGCTGTTTCTGAAACCTTTGCTCTCTTGAATAAAATGTCACAGGTCACCCTCCTGTTATGCTTTGTTCTGAAGCCTTTCCCTTTTCTTTACTGG

TTTTTATCTGTCTCGTGCCTCCACGTGGACTATACGATTCTTTTTCTTAGTTTTCTAATGTGAAGGTTTACGTGGAGCTTAATCCATTAATACCATATCC

ATCCACACGAAATGGTTTTAATGGA

>Glyma.15G058900 | Chr15:4545863..4553268 reverse

GTCCCTCTTACAAGTACAACAGCAAGAGTGGCTTTTTGGTGAATTGATTGGTGATCCCGAGTTGGTTGAGTTCACTGCGCATAGACATGTTCCTTAATTT

CCCCAAATATTAAAACAAGATTCAAAGCACGTCACTTACGTTCCCGCTTTCTGTTTAACAAGGTACCACTCGTTTAGTCGTTTATTTGTACAATGCCTCT

TCAATCATTTCAATTTGTTATATGATGAAGAAAAACCCAATTTTTATGTTGCTGCAGTGAAAAACTTGAACCTTTTGAGACCTCAGTTCTCTGCCATGGC

AAATTCTCATTTATCAACACCTTCTGAGCTATTTTCAAGACAACATAAGCCCCCTGTAAGCTCAAACTTGGTTTCAATTTCCACAAAATTTGACTTCATG

TTGCTAGTTATCTATTCATCTCACCTCCCATTTTTGTTTGGTTGTGTTGAATTAATCAGAGGGTTCCAAATCTTATTGGGGGCAGTTTTCTGGACTCAAA

ATCGTTAACTTTCATTGATGTTATCAACCCTGTAAGTGTTCTTTCTTCTTTTGGTTTGATATTTGGCTTATAATTGAGCTGAATCCCTTGATTTTGGTTT

TGAGAAAGGAAGAGTTCTAATATTGGTATCATCTTATAAGGCAACACAAGAGGTTGTTTCACAAGTTCCTTGCACCACAGATGAAGAGTTTAAAGCTGCG

GTATCTGCAGCAAAGAAGGCATTTCCATCATGGCGAAAAACTCCAATCACAAAACGCCAACGTGTTATGTTGAAGTTCCAGGAGCTTATACGCAGAGATA

TGGTAAACATTTTACAATGTTCTATGCCTCTGCCTCATATATTCACAGATTTAGGCCAAGACACCATACTGGCACATATGCCATTTAGCCATATCACTAC

TTTGTAACTGTTTCAGGATAAACTTGCCCTGAATGTGACCACTGAACAAGGAAAGACGTTAAAGGATGCACAAGGAGATGTTTTCCGTGGTTTAGGTTGG

TCTTTCTAGTATGTTGCTTGAGAAGTTTTTGAGATGAGATATTATTGGTTGAGCTTATAATTTGTGATGAACTTCTCTAAACATTTTGTCATTTAGAGGT

GGTTGAACATGCTTGTGGGATGGCAACTCTACAAATGGGAGAATATGTTTCTGATGTATCAAGTGGAATTGATACTTACAGCATTAGAGAACCACTTGGT

GTATGTGCTGGTATTTGTCCATTCAATTTTCCTGCAATGATTCCCTTGTGGGTAAGTTGTTTTCTACTTTTTTGGTGGTTATTTTGCTATGCAATTGTTT

TTCTCTTACAAGTTCTTGGGTACTCCTTTATTTCTTGTTTGCTTGGTTTTCTCGTCCATATCAGTATGCAAAGATGTATGATATTTTTGCAGATGTTCCC

AGTGGCAGTTACATGTGGCAACACCTTTATTCTAAAACCATCAGAGAAAGTTCCAGGTGATACTTTCTACTTAATTTGGTTGTTTATGAATCTCCTTGCA

TTAACTCCTCAGTTTGGATGGAGCTTTGCGTTATATATAATGGAAAAGGGAGCTCTGTAATTCAAAGCAAGCTTAAGCTTCTTTAATAAGCTATTGGCAT

TATGACTTCAGAACATGTTACCCTAAATATCTTGTATTGTGAATACCAGGATCAAGGATTTTTTTAAATAAAATGAATGCCTTCATTATTTTCTTCAGCA

ACTGATAAGGATTTTGCAGTTGCTAGCCTTGCATATTGTGTCTATATTGCCAATCTAGCTATTAATAAATTGCAAATTTTGAAAATGACAAATGAGAATA

AAATTCCAAAATTAATGTTCTTGGTATAAGTTTTGTTAAGCCTTCCTTTCTTGTTATATTCAAATACTTTATTGGTTGTGATCTTCACTAAGGGATTTTT

GAAAGAATATTTGTAGGAGCAACATCTTCACTAATATTCATTTTATAATTTAAAATAATAGATAACTACCCTCAACTGCTTTGAACCCTGTCTGCACTTG

CTCTTTTTGCAATCTAAATCCATTTAAACTAATTAAGGACGAGTTATTTATCAGATTAGGTTTGCATTTTGCAAATGATACTCTAGCAAGAAAAGGAAGC

TAGTGTCACCCAAGTAAAATATTGGTGCCTTGACTTTTGACTTTGTCCTTGTTTCTGCCTCACATGGATCATCTAATTAAATTTGATTTCTGAATAGATA

TATTAGGAAGAATGAAACAATGTCAGATCTTACAGTTTGAGAAGTCTATATTAATTTTTCTTTGTGATGGCTGATAAATGTGTTCTTATTTATCCATCTA

GTAATGGAATACTAAATATTATCGTCCACCCTTGAGTTTTTCTTCCCCATCCATGTAACGGTACATGCAGCATGCTTTTATGGTAATAGCACACTTAACT

GTCAGAACTCAGAAATATACAGTCTGCTGTCCACTTTCATGATTCATCATTTTTGGTTACTTGCAACTTATGGCAGAGAGAGTTGGATGTCATAACATTG

GCATTTTTCCAGGTGCCTCTGTAATGCTTGCAGAATTGGCAATGGAAGCTGGTTTGCCTGAGGGTGTCTTAAATATAGTTCATGGAACCCATGTATGCTA

TAACCTTTTCTAAACTTTTGTTCTTCACTCTTTCCTAGTAAATGAACTCATGGGCATATTTGGCATTTGGTACTTAATTGCATCATGTATTAAAGATTTA

AATCAAAGAGTTGCAACTTGCATATAACAACCTGTTGCATTGTCATACACCCACACCACTTTTTGTACATTGCTTTTCTTGTTTATTGATGTTCTTAATA

ACCCAATTGTTGACAGGATATTGTGAATGCTATTTGTGACGATGATGACATCAAAGCCATATCTTTTGTTGGTTCAAATGTTGTAAGTCATCTAATGCTT

TGTTTAGTTTTACTGCTTTTATGTTATGCCTTTTTTATTTAAACCAAAGGCAATGCTGTTTTTGTGTTGGCAAAATTGAATCATTTTACAATTTGACTGT

CCTTACATAAAAAGATGGAAGAGGACTAGTGATTCAAATGCTATTTTTTCTTAACTGATACTCTCCCCTTACATAAAAATTTGTTTTCAGGCTAATATTT

TTTTTTTGATGTTTTTATTTGTACAGGCTGGAATGCACATATATGCTAGAGCTGCAGCTAAAGGGAAACGTGTTCAGGTGGGTGCACTTAGCTTTGTCCT

CCCCTTAGTAAAATTGTTTATTACTGTTCATGGTAATGATCAGAAGTTTTGGACCTAATAAATTTTGTTTTCAGGCTAATATGGGGGCCAAAAATCATGC

AGTTGTAATGCCTGATGCTAGTGTTGATGCTACTGTAAATGCTTTAGTTGCTGCAGGTTTTGGTGCTGCTGGACAAAGGTGCATGGCTCTCAGCACTGTT

GTTTTTGTTGGAGACTCAAAACTGTGGTATCCTTCTCTACCATATTGTTATTCAATTTGATTTATTATTATTTTTGTGGTATCATCTTATGCTTGAAGCA

ATTTTATGCTCTTCCTCTGGTGCTTGTTCCAAAATGAACTGGATGTCTTTGGTGTGTACCAAACTGAATCACCACTCTTCCTTTACTAAGACAATTCAAC

ATCTCTAAAATATAATCACATTTTGTGAGCATTCTTACTGCTGTGAAAATTAGTAGGGATGATTAATTATTTCCATCCTTTTGATAATTCTGATGGGATT

GCCCCAAATAGACTGTTCCAAGTATTTGGATTTCTTTGTGAGCAGTCTTGGTGAGGAAATTGACTTTGACTTCTTCTTTGGCCTCATAAAACTTACTTTT

AACCATGCATTTATAATATTTTAAGTATAATAACTTCATCCTTTAGTCTTCACAATATTTTTCCAGGGAAAGTAAACTTGTGGAGCATGCCAAAGCTCTT

AAAGTAAATGTTGGAACTGAACCTGATGCAGACCTTGGTCCAGTCATTAGCAAACAGGTATGTTTATGGTTCTTGGACTAATAGAAAACTTCAGATTATT

GTTTAAGAATTACTATTCAGTGCATCACATTTCCAAATTGATATTGTATACATTGTAGTTATATGTTTCTTAAATATTCTCTCAACCTTACCCACTGCAC

AGGCAAAGGAGCGAATACACCGATTAATTCAATCTGGGGTTGAAAGTGGTGCCAGACTAGTTCTTGATGGAAGAAATATAGTGGTAGCTTTAATATTCTC

ATTCATTTTTATCATTTCATATTTTATTCATGGACATTTCCATACTTTTTCATGCATGATCAATTTACCTCTTCAATCTTTAGCATAGCAATTGACATGT

TTGGAAACTCAGAAGGCAGCAGAAAGATTTATGTATGCAAAGATTGCAACTAAAGTAATAATCTTCGATTATAGCACTCCTCTTCCTGAGGCCTTATAGA

AAACAAACAAAAAAGAAGAAAAACATATAGGAGATCATGAAACATTTATCTTTGTGTTGCATTTGATTTGGTTGATAACTAACAGTTCTTGTACAGTTGT

ACTGGTTAACCAATTAAAACCAGTTAAATACTACCGGTTAAAGAAATGTAACCTTCTATATTCTTGTATAATTCTTTTGGAACCTTGAAAAATGGTGCTT

GGTTCAGTTGTTTGGTGGTATGCATAAAGCCTGTCTCAGTGCAATAAAAATTGGTTTGGCTTAATTTTTAACTCCTGATTTTGTTACTTTGTGTTGGGTA

ATCAACTCTCCCTCAAATAAAATTATCCACACACACAAAGGATCGTGAAAATACAACAAAGATTACACCCTAAAAAAAAATAACAAACACAAAAATTTAA

CATGGTTCAACACCTCTTACTTACATCCACGAAATCGTCTCAAAAGTATTTCACTATCACAAAAATGATTACAAGATTGTAACTCATCCTTAATAGTGTA

TCACTCTACAAACCCGAGTACCTCACTCAATGGTTACAAGAAATGATAATATTCTCACACAAAGACACTTTTCTCTCAACAAAGTGACTTTGTTTCTCAA

TCTCTCTTCTCACACACTCTCTCTTCATATGTTTCTTCTCTACTAATTCTTTTTTCTATTTATAGTGAAGATTGTCACTAATTATAATAAATAAGTTCTC

TTGAAAGTTGAAAGAAAAACAACTTCCAATGCATCCATTCAAATAAGTTTCATCTAGTAAAATACAATTTTTCACTTTGCATTTGAATCACCTAAATCTT

AGTGATAAAAATTTAATTCCCTATATACATTAAATGCACTTTATCTTTTGACTTGGAACTCTACATTTTGTTCATTCTTGCTCCTTGGTTCTCCCTTCCC

TCCTTTGACACCCATCAAGCTGAGATGCAATAAGTTAAAATGAAGCACCCCAAATGGAATGTAAAGGACATACAGTTTTGTGCATATCATTATTCCACAT

CCTGAAGATCAGTCCTTGCATAGTCCTTTCTTTTTCCATATTGTCAATGGATTGTTTTCACCTTTTAATATCATTTTAAGACTGCTCAACAATTCTTTTG

TGAATAAGTAGGTCCCAGGATATGAATCTGGCAATTTTATTGGCCCAACCATCTTATCAGATGTCACCGCCAACATGGAGTGCTACAAGGTAATTTTCTC

ACTAATCTCCAATCTGTAAGTGTTTACTAAAGAATTTTGCAAGTGTTTACCAATCTGTAAGTGCTACAAGGTTTAGTCTTGAACTAAACTTGATTATCTC

TATCATGGCCTGTGCAGGAGGAGATCTTTGGACCAGTTCTTCTTCTCACGGAGGTGCATTTTATTTGTCTCATACTAAGCATCCACTAATTTGGAATTAT

TTTATTTGTCAATATTTGGTCATGTAGGAGGCTCATCTGCCTTGGATTTTTCATTTTAGCTAAATATAATGTTATGCTGACACAGTTTTTTAATTTTTTT

TTTATTTTTAACACTAGGCTGATAACTTGGAAGAGGCCATAAACATTATCAATGAAAACAAGTATATTTTAACAACTGCTACATTTTCATTTGTATTTGT

TAAACACTTAAACTTCCATTTATCTCTTTGACTTTTAAAACAGGTATGGAAACGGTGCTTCTATATTTACCACATCTGGTGTGGCTGCAAGGAAATTTCA

GACCGAGATTGAGGCGGGGCAGGTTTGTCATCTAACTATGATGTTTTCTAGTATCATGATTGGTCATGTGACTTTTTAAAAAACTTGAGTTATGTGTTTT

AATCTTGACCATTGATTAGATTTAACGCTCAATATCAATTTCTTTCACTCACTTAAAAAGGTACTTCTCACTATATATATTTGACACTGTTAATGTTATG

GATCATCATTTTGTGACTGCATTTGAACTAGGTGGGCATCAATGTTCCTATTCCAGTTCCTTTGCCCTTTTTTTCATTTACTGGCAACAAGGCATCATTT

GCTGGCGATCTTAATTTCTATGGTATTGTGATTTCTCCCAAAGCTAGCTCTTTTCAATTTTGCATGTGATATTGGTTATACCCTTGGATTTGAAATTGAT

ACAATTTTTTGAATGACAGGTAAGGCAGGGGTTAACTTTTATACACAGATCAAAACAGTAACCCAGCAGTGGAAGGATTCGGCCAGCGAAAGCAAGATTA

ATTTGGCTATGCCAACCTCTCAAAAATCTTGAGTCCGAAGTTTATATTGCCTCTGAAACCTTTGGACTCTTATAAATCCTGTTAAACGATTGTCTTATAT

CCTTAGCAAATTCTTGTGTAAATAATACACCCTGATCCACCTATTTTTATAAAGTATTGTACTATGTTATGTTTTATTGGATGTGTGCTACTGTATTGTT

CTCAGAATAAATGAATTTCTTGGGACTCGTAGGTAACGGATTTATTAATTTTCAACTTGAGAGTGGATGATAACTGCTAAATAATTGTATTTTGATAGTA

GAAAATTAGTCAAATATTGGCTTGAAATTAATTATTTAGCAGTTATTTGTGATTAAAAGTTAGAAAATTAATTAAATTGAATTTTTGGGTGCAGATATAA

AAATTGGAGGTGTAACAAGCAAAAAGGGCAGAAAAATTGAAGAAAAGAAGAAAATTTGAAGAAGGCCCAATCCAATACGCGCGCTCAGCGCGCGTCACGG

GCTAAGCGGGCCAGGAAGTACACGCGCTAAGCGCAGAGTGTCACGCTAAGCGCGCCTACGAAGGCCCAAAGCCCACTTCAGCAGCTATAAATAGAGAGTC

AGTCCAAGGGACAGAACACACACCACCACAGAACCCCCTCTCCTAGGGGTTTCATTTACTCTCTTTCTTTCTTTCACCTCCTTCTCATTCTCATTGTAAA

GCCCTCGTGACCATGAGTGGCTAACCCCCTAGCTAGGGCCTGGCAGGCCTAAAAAGCCAATGATGTATGGAACATTTTAAGAGTTGTCAATAAAAAGAGG

AATTCC

>Glyma.09G070300 | Chr09:7178794..7185018 forward

TTTTGTCCCACGTTATCTCAAAGTGCAAGCTTTGTTATTGTAAAAAAAAAACAAATACTGAATCCCCATCACTTTCTTCCCCATCGTATGTGTGTCCCTG

TCAGAGTATGATATGAGAAACTCTATAAATTAACTATTTTTTCTCCCACTTTCTGAATCAAACACAACACACACACGTAACTTCATCTGGGTTTCAGGAA

AAAAGAAAAAGGAAAACAATGGGTTCCGATAATCACCAGAACTTGGAGTTCTTGAAGGAGATCGGTTTGGGCTCTTCCAACATTGGCTCTTACATTAATG

GCCAGTGGAAAGCCACTGGTTCTTCTGTCACTTCTGTTAATCCTTCTAACAATCAGGTTCACCTTCTCATAATTAATTTTTTTCTTCAATTTTTGTTATT

TATTTCTTTATTTTGTGAGGCTTTCTTGTCTTTTTTGTTTTCATGTGTATGTGATTATTGGTTATTGGTGATTTGAAAATTAGAGTATAGCTCAAGTGAC

CGAAGCAACTTTGCAAGATTTTGAGGAGGGGTTGCGAGCTTGCAGTGAAGCAGCTAAGACATGGATGACTGTGAGTTCTTTTAAAAATACTTTTAATTTT

TTTTTTTTTTTTTTTTTGGGGGGGGGGGGGGGGGGGTGGGGGATGTGTGTGTGTTATAGTTGTGTTTTGGTTATATGTGGTAGAGTTGGTAGCTAGTAGC

CTTTTTTTTATGGACAAATGTTAGAATGTTAGTTTTTGTTAGCAAAAGGATCAAACATTCAACCCACCTTATATCTCCCTGGTAACCTAGTACTGAATCT

ACGTGCTATTGGAAACTAATTGAGGATGTGCTTGTAATGTTGTTGCAGATACCGGCACCGAAGAGAGGTGAGATTGTGAGACAGATTGGTGAAGCATTGA

GGGCCAAATTGGATCCTTTGGGTAGGCTGGTGTCTCTTGAGATGGGAAAAATTCTCCCAGAAGGAATTGGGGAAGTTCAGGTATCGCAATTCTCTTTGTT

CTGCCATATTATGTTACATGTCCTGACACCAAAGAAATCAGAGTTTAGATTGTGTATAAGGCTATAATACTTCTTTGATGGCAACTAAATCAAGATAAGG

TGGTGAGCTTTAGCTGTTTCTAATTAGAACAGTGTGATATATTCAGGGACATACAAATTTATGTCATTCTGATGAAATTTATGCCCTCTAGCTGATTATC

CGGTGATTTTCATTGCAAATGTTCTTAGAAATGTGAAGGGATTTGATGCTTTTTCATGGGGGTCTTCCTGAGCAACTGCTCATATCTAACTACATTTACA

TTTTATTTTCATCGATCGCATATGTTTTTCACCTAATCAAAAGGTTCTGTTCTCAGAAGAAAATGGCTGTTCTATTCAGTTTTTTTAGTAAATTGCGTAT

ATTTCAGAATTTTCTATATTCACTGCATCATCTTGGCTTAGGCCCCATGGTTCTCTGAGGATGGTTGCTTTTCAAAGTTACTTCATTCTTTCTATTGGGA

GCATTCCATATATGTTTGGTGTGTATATAGGCTAACTGCACTCTGAAAGCATTTTAGTGTTTTTTCTAATTATGCACAGAACTAAATTAATCACATACAC

CATGGGAAAGAGAAAATTATTAAAGATTTTGCACATAACATTTTTATATGATATAGTTTGTGATCTTTATCCATTACAGGAAATTATTGATATGTGTGAT

TATTGTGTTGGGCTAAGCAGACAATTGAATGGATCAATTATACCATCAGAACGTAAGATTGTTGTGTATCTATATTAATTGACCATCTTTTCTTTATAAT

CCTTCTGGTTACAAAGTTATTTAAAATCATATGTAGTTAGCGATAGTCTCTTGTTTTTATACTGTTTTCTCCAAGATATTCCAATTTTGAGTTTTTGTTT

TCTTCTACTTGTTTTTTAACAGGTCCAGATCATATGATGTTTGAGGTGCGTATTTGAAGTATAATTATTCACTGACTTTTGTACTGATTTATATGATATT

AACTGTAACCCATGCTTTTACACCACAGGTATGGAACCCACTAGGAATAGTTGGTGTAATCAGTGCTTTCAACTTTCCATGCGCTGTTCTAGGTATGTAT

TTTCTCATCCTGTCTTAATGCACATAGCAACAAATTCCTTTGGATCTTAGATTCAGTTCAAAGTTACTTATAATCAGATTGAAAATGTTAGTGCATTCAT

TCACTGTCATTTGGTCATCTAATTTAGGCATGTATTTTAAACTTTACCTGAAAATCTGTTGTGTCTAGGAGCCATCTTGATCTGTCATCAATCAGATTGT

GTGTGAACTGTTGTTCTGATAAAAGCTTGCAATATCATCAATTCATTAAACTTCTTGTTATCAAATTCATCTCCATGTTTAGATCATTTTTATTTGGTGA

TATGTTTTAGCTTTTTGTTACTTTTTTAGCTGCTCTTCAATCTTTCATATAATACAAAAGTAAGTCAGAATGGGGATTGTGACGTTGAATCTTTGTCAAT

TAAACATACTACTGGTTTCATTACTGACTCCTACTTTAAATAAATGGATGGGTGCCAACATATATGAGCAGATAGCTTTCTTGAAGTTTTTCCATCTAGT

TTGACTGCATTTTGCATCATACAGTCCACTTTCTATGATCTGATTTTTTTTATGTGTTGTTTTTTTATTGTAACTAAAACATGTTCATTTCTGTTGTGCT

TCAGGATGGAATGCTTGCATTGCTTTAGTCTGTGGTAACTGTGTTGTGTGGTAAGTCTATATATCTATCTCTGTATATTTTATGAGATTACTCAACTGGA

CATTTTTAAGTCAGATCATGATTGTTTTGTTAATATTTCATGACCTCTGCAATATTTCTTCTCTATTTTCATGTGACAATCAGCTTTGATCGAATATTTC

ATTTGCAAAATTGGTTGGAATAATGGTATATATGGACTTCGGATCAATTTTCATTGGTCTTATTGTATCCACCATTTTCTTTGACTTGAACTAGTGTAGG

AACCCGTACTTCAGTTAGACTCAACTCTATTTAACCAATACTTCAGTTCTTGATGCATTAGTGTTTAGTTGTATGTATTTTATCAGCACCAATTTGTATG

ATTGGTCAGAAACAGAGATATAGTGAATGTCTTCCTTGACTTTTTGGCTTCCTGCAACCACACATCTTGTTAGGGAAATTCACCTCTTCGATGGATCTCC

TATCCAATTTAAACGTATTAGGTGAATATATCTTAGATGACAAAATGTTGTTGCTAGAGAAGCTGAAAGATTTGGAGATAATATGCCCTCCAACTTCTCC

AATTGCACTTCCAGTTCCTTTGACTTATTTTCTTTTTACGAAGTGTCATAACCAATGACATCAATCCATGAAGCCTAACTCTCATAGTGGAATAAGGCTT

GGTGGTGGTGGTTTTTATTGTTGTTCATGAAATGGTAATGGTTAAAGCGCTATATATTTTTGTAGTTCAATCATCCTTGATTTCTGATTTATCTCCATAA

TGATTGCATACTTCATGCTTATCTCCATGTTGCACTTTGCCTACCCTTCAGCTCCCTCCCCCCCCCCAAAAAAGTTCCATATAATATACACAGTCATGAA

AGAAGGTCTATTAGAAAAGATGATAAAATTATCTTTGTTGGTGCCAATTATGTAGATCTGTTTGGATTTTTTATGTGATATATGTTCACTTGTCAGATTC

TTGAGTGTTTCAAATTTTCAATGGCAATCATGTTATCTTTTTAATATATGTTTTTTCTACGGCATCCATTAAAGGAAGGGTGCTCCAACAACTCCTTTGA

TAACTATTGCTGTGACAAAGCTAGTAGCTGAAGTTCTTGAGAGGAACAAATTACCTGGTGCAATATTCACCTCTTTCTGTGGAGGTGCTGACATTGGTCA

GGCAATAGCAAAAGACACTCGCATTCCCCTGGTTTCATTTACTGGAAGTTCAAAGGTATGTTATGTACTAAGTAGTTGGACATGAAATTTTAAACAGGAA

ACATAATTAAACTAAGTTTAATGCATATACACTGACGGTATAAAACTGTCTTACATTGTCTTCCAATCACATATCACCGTTTGAATTACTTTAAGATAAT

TATTTTAAAAGTCAACAAACTTACCATACATGGTTGGTTGTGATTGGATGGCTGTGTAAAACTTTTTACACTGTCAGTGCATAACTCTTTTTCTCTTAAA

ATAACTGAATAGGATTTATAAATTTATATTTTGTGTTAAAAGATGTTGATGTCACCTTAATGGTGCTCAGTTTAGTTGCCTGTGGTACTAGTAGTATCGG

TCTTATTGCAGTCACTGTCTAAAGATATGTGATATCAAACACACTAAAATATCACCCCTTTAGCGAAATTACTTCCATGTGTCATGTACTGGGTAATTGT

GTATGTTGCTCTTGTGCCCCACGTTAAAATAAAACTGTGCAAACATTTACTTATTTCATAATGATTTGGACAATACGATCTATGCTGTTTAGTTCATAAT

GATGTGTTCCCTGTTATATAGGTTGGCTTGATGGTCCAGCAAACAGTTAATGAGAGATTTGGCAAATGCTTGCTTGAGTTAAGTGGTAACAATGCAATAA

TTGTCATGGATGATGCAGACATCAAATTGGCTGTACGCTCTATTTTGTTTGCTGCTGTGGGTACTACTGGTCAGCGGTGTACAACTTGTCGTAGACTGGT

ATTAGAACCAATTTAAAAAATTGAATTTCATAATTTTAATGCAATCACGTGTGATTTTTTTAAGATATATTTAATTTTTAATTTAGCTTCCTTGTACTGC

AGTTTCTGCATGAAAGTATTTATACAGACGTACTAGACCAACTTGTTGAAGTCTACAAACAAGTCAAAATTGGGAACCCCTTGGAGAAGGGGACTCTAGT

TGGGCCCTTGCATACTCGTACTTCAGTAGAAAACTTTCAGAAGGGTATTTCAGTCATAAAATCTCAGGCATGGTTCATTTTACTCTTTTTCTAGTTAAGT

TAAATATTTAGGGGATGTAACCTTCCATTTCATGTCTGTAATTTTGCTCAGTAGGATATGCAAGCTTTTTCTCATACATATACCACGGATTGGATGCTTG

TCTTCTGTGTAGAGATAATTTACTTGATACTCTGATTATCAGGGAGGGAAAATCCTAACCGGTGGATCTGTATTAGAGTCGGGAGGAAATTTTGTACAAC

CAACAATTGTTGAGATTTCTCCAGATGCTCCTGTAGTTAAAGAAGAATTGTTTGGTCCAGTTCTGTATGTTATGAAATTTCAGGTAGCATATATTCTATA

TCTTCTTTCATCGGACTATTTCTTTTGGAGTTTAGTTTATGATCAAACTTCATGATTTTCTACTTAGACTCTAGAAGAAGCAATTGCCTTGAACAATTCT

GTACCTCAAGGATTAAGTAGTTCAATCTTTACCCAAAGACCTGGAACTATATTCAAATGGATCGGGTAAGTACTCACAAACTTATTTTTGTCCTATCCTT

TGGATCTCTGCTAATGAGTTTGGCCTACACACTTCAGTACTTGACTAACAATCCTATAAGATGTTTGTGGTGATTTATAGTTGCACAAATAGTATATTTT

ACTTGTTCATGGGTGTAAATATGGTGCGCAGGCCACGAGGTAGTGATTGTGGTATAGTGAATGCAAACATACCTACAAATGGAGCTGAAATTGGTGGTGC

CTTTGGTGGAGAAAAGGCGACAGGTGGTGGCCGTGAAGCGGGAAGTGACTCATGGAAGCAATACATGCGGCGTTCTACATGGTAAGTCTTCGTTGAATTT

ATGATTGTTACATTCCTTGGTTATAAGCTCTGTTAATGGTGAATATATCTTAATATTTTTTCTGTGCATGCTCGTAATGACATTATAATTGCTGTTCTTG

GCAGTACCATCAATTATGGAAGTGAACTACCATTAGCTCAGGGGATAAACTTTGGCTAGAAAGCTATCCTAGCACATGAGGCTGCACAAAATAATGCGAT

TTTGCAGTCTGAAGAGAGTTCTATATATACCGAGATGTTAGTCTGGCTGACATGGGTTCAAAGTGTTGTCTTAACAAGACATGCTTGCTGTAAGGATGGC

ACCTTGATATGACAGTTTCAATGTATAAGATATCTTAAATAAATGTTAAATGAATAAGCTGAGAATTCTTTTCCCAGTCAAAAAGTGTTTGATGTTTAAA

TTCGTGTTTTGTGATTTTGGCCATT

>Glyma.15G178400 | Chr15:17015128..17020833 forward

TCACTTTCCCCATCCCATGTGTGTCCCTGTCAGAGTATGATATGAGGAACTAACTCTATAAATAAAACTTTCTGAATCAAACACATCACACACACGTAAC

TTCATCTGGGTTGCAGGGAAAAAGAAAAATAAAATGGGTTCCGATAATACCAACTTGGAGTTCTTGAAGGAGATCGGTTTGGGCTCCTCCAACATTGGTT

CTTACATTAATGGCCAATGGAAAGCCACTGGTTCTTCTGTCACTTCTGTTAATCCTTCTAACAATCAGGTTCACCTTCTCATATTTTTTTTTTCAATTTC

TATTGTTTATTTTGTGAGGCTTTGTTGGTTTTTGTTTTCATGTGTATGTGATTATTGGTTATTGGTGATTTGAAATTAGAGCATAGCTCAAGTGACTGAA

GCGACTTTGCAAGATTATGAGGAGGGATTGCAAGCTTGCAGTGAAGCAGCCAAGACATGGATGACTGTGAGTTTTTTGTTTTTAAAAAAAGAACATTTTT

TTAATACTTTATTTGGGGGGGTTATTTTTTGTGTTTTAGTTACATGGTAGAGTTGGTAGATAGTACTGCATCTATGTGTTATTGGAAGCTAATTGAAACT

AATTGAGGATGTGATTGTAATGTTATTGCAGATTCCGGCACCGAAGAGAGGTGAGATTGTGAGACAGATTGGTGAAGCATTGAGGGCCAAGTTGGATCCT

TTGGGTAGACTGGTGTCTCTTGAGATGGGAAAAATTCTCCCAGAAGGAATTGGGGAAGTTCAGGTATCACAATTATAACTGTTCTGCAACTTTTTTTGAC

ATATTATCAGGTTTGACAGCATAGAAATCAGAGTTTAGATTGTGTATAATACTATAACACTTCTTTGATGGAAACTAAATCAAGATAATTGGTGGTGAAC

TTCAGGTATTTCTAATTAGAACAGTGTGATGTATTCAGGGACATCCAAATTTATGTCGTTTTGATGAAATTTTATGTCCTCTAGCTGATTATCTGGTGAT

TTTCATCTCTAATGTTCTTAGAAATGTGAAGGGATTTGATGTTTTTTCATGGGGGTCTTCCTGAGCAACTGCACATATCTAACTACATTTACATTTTTTT

TCATCGATCACTTATTTTTTCACCTAATCAAAAGATTCTGTTATCAGAAGATAATTCTAATTTTTATTCCAAAATAAAATGGCTGTTCTATTCAGTTTTT

TTTAAGTATATTGCTTATATTTCAGAATTTTCTATATTCACTGCATCATCTTGGCTTAGGCCATATATCTGGTTATCTGAGGATGGTTGCTTTTCAAAGT

TACTTCATTCTTTCTATTGGGAGCATTCCATAAATGTTTAGTGTGTATATAGGCTAACTGCACTCTGAAAGCATTTGAGTGTTTTTTCTAATTATGCACA

TAACTAAATTAACCACATACACCATGGGAAAGAGAAAATTATTGTGTGAAAGATTTTGCACATAACATTTTTATATGATATAGTTTGTGATCTTTCTATC

CATCGCAGGAAATTATTGATATGTGTGATTATTGTGTTGGGCTAAGCAGACAATTGAATGGATCAATTATACCATCAGAACGTAAGATTGTTGTGTATCT

ATATTAATTGTTCATCTTTTCTTTTTAATCCTTCTGGTTACATAGTTATTTAAAATCATATGTTGTTAGTGATAGTCTCTTGTTTTTTTTTTTTTTCTGA

TTTCTCCATGATATTCCAATTTTGAGTTATTGTGTTCTTCTACTTGCTTTTTAACAGGTCCAGATCATATGATGTTTGAGGTGCGTATTTAAAGTATAAC

TATTCACTGACTTTTGTACTGATTTATATGATATTAACTGTAACACATACTTTTACACCACAGGTATGGAACCCACTAGGAATAGTTGGTGTAATCACTG

CTTTCAACTTTCCATGCGCTGTTCTAGGTATGTATTTTCTCATCCTGTCTTAATGAACATAGCAACAAGTTCCTTTGGATCTTAGATTCAGTTCAACATT

ACTTATAATCAGATTGAAAATGTTAGTGCATTCATTCACTGCCATTTTGTCATCTAATTTTGGCATGTATTTTAAACTTTACCTGAAAATATGTTGTGTC

TAGGAGTCATCTTGATCTGTAAGCAACTAGATTGTGTGTGAACTGTTATTCTGATAAAAGCTTGCAATATCATCAATTCATTAAACTTCTTATTATCAAT

TTCATCTCCATGTTTGAATCATTTTCATTTGTTGATATGTTTTAGCTTTATGTTACTTTTAAGCTGCTCTTCAATCTTCATATAATACAAAAGTAAGTCA

GAATGGGGATTGTGACGTTGAATCTTTGTAAATTAAACATACTACTGGTTTCATTACTGACTCCTACTTTAAATAAATGGATGGGTGCCAACATATATGA

GCACATAGCTTTCTTGAAGTTTTTCCATCTAGTTTGACTGCATTTTGCATCATACAGTCCTACTTTAAATAACGTTTTAATGTTTTTATTATAACTAAAA

CATGTTCATTTCTGTTGTGCCTCAGGATGGAATGCTTGCATTGCTTTAGTCTGTGGTAACTGTGTTGTGTGGTATGTCTATATATCTATCTCTGTATATT

TTATGAGATTACTCAACTGGACATTTTTAAGTCAGATCATGATTGTTTTGTTAATATTTCATGACCTCTACAATATTTTGTTCTCTATTTTCATGTGACA

ATTGGCTTTGATCGAGATATTTCATTTACAAAACTGGTTGGAATAATGCTATATGTGAACTTCAGATCAATTTTCATTGGTCTTATTGTGTTCCACCATT

TTCTTTCACTTGAACTAGTGTAGGAACCCATGCTTCAGTTAGACTCTCAACTCTATTTAACCAATACTTCAGTTCTTGATGCACTAGTGTTTAGTTGTAT

GTATTCTATTAGCACCAATTTGTATGATTGGTCAGAAACAGAGATATAGTGAATGTCTTCCTTGACCTTTTGGCTTTCCTGCAACTACACATCTTGTTAG

GGAAATTCACCTCTTTGATGGATCTCTAATCCAATTTAAACGTATTAGTTGAATATATCTTGGATGACAAAATGTTGTTGCTAGACAAGCTGAAAGTTTC

GGAGATAATATGCCCTCCAACTTCTCCAATTGCACTTCCAGTTCCTTTGACTTATTTTCTTTTTCCGAAGTCCCAATGACATCAACCCATGAAGCCTAAC

TCTCATAGTGGAATAAGGCTTGGTGGTGGTGGTGGTTTTTATTGTTGTTCATGAAATGGTAACGATTAAAGCACTATATATTTTTGTAGTTCAATCATCC

TTGATTTCTAATTTATCTCCATAATGGTTGCATACTTCATGCTTATCTCCATGTTGGCATGTTGCTCTTTGCCTACCCTTCAGCCCCCTCCCCCCCAAAA

AGTTCATATAATATACACAGCCAAGAAAGAAGGCATGATGATAAAATTATCTTTGTTGGTGCAATTACATAGATCTGTTTGGATTTTTATGTGATATATG

TTCACTTGCCAGATTCTTGAGTGTTTCAAATTTTCAATGACAATCGTGTTATCTTTTTAATATATGTTTTTCTATGACATCCATTAAAGGAAGGGTGCTC

CAACAACTCCTTTGATAACTATTGCTGTGACAAAGCTAGTAGCTGAAGTTCTTGAGAGGAACAAATTACCTGGTGCAATATTCACCTCTTTCTGTGGAGG

TGCTGACATTGGTCAGGCAATAGCAAAAGACACCCGCATTCCCCTGGTTTCATTTACTGGAAGTTCAAAGGTATGTTATGTACTAAGTAGTTGGACATGA

TATTTTATACAGGAAACAATTCAACTAATTGAATAGGATGTATAAATTTATAGTGTTAAAAGATGTTGATGTCACCTTAATAGTGCTCAGTTTAGTTGCC

TATGGTACTAGTGGTATCAGTCTTATTGCAGTCACTGTCTAACACACTAAAATATCACCCTTTTAGTGAAATTACTTCCATGTGTCATGTACTGTGTAAT

CATGTATGTTGCTCTTGTGCCCCCTATTAAAACAAAACTATGCAAACATATCTATGCTGTTTAGTTCATAATGATGCGTTCCTGTTATATAGGTTGGCTT

GATGGTCCAGCAAACAGTTAATGAGAGATTTGGCAAATGCTTGCTTGAGTTAAGTGGTAACAATGCAATAATAGTCATGGATGATGCAGATATCAAATTG

GCTGTACGATCTATTTTGTTTGCAGCTGTGGGTACTGCTGGTCAGCGGTGTACAACTTGCCGTAGACTGGTATTAGAACCAATTTAAAAAATTGAATTTC

ATAGTTTTTAATGCAATCACATGTGATTTTTAGAAGGTATATTTAATTTTTCATTTAGCTTCCTTGTACTGCAGTTTCTGCATGAAAGTATTTACGCAGA

TGTACTAGACCAACTTATTGGAGTCTACAAACAAGTCAAAATTGGGAATCCCTTGGAGAAGGGGACTCTAGTTGGGCCCTTGCATACTCCTACTTCAGTG

GAAAACTTTCAGAAGGGTATTTCAGTCATAAAATCTCAGGTATGTTTCATTTCACTATTTTCTAGTTATGTTAAATATTTAGGGGATGTAACCTTCCATT

TCATATCTGTAATTTTGCTCAGTAAGATATGCAGGCTTTTGCTCATACATATACAATGGATTGGATGCTTGTATTCTGTGTAGAGATAATTTACTTGATA

CTCTGATTATCAGGGAGGGAAGATCCTAACAGGTGGATCTGTATTAGAGTCAGCAGGAAATTTTGTACAACCAACAATTGTTGAGATTTCTCCCGATGCT

CCTGTAGTTAAAGAAGAATTGTTTGGTCCAGTTCTGTATGTTATGAAATTTCAGGTAGCATATATTCTATATCTTCTTTCATTGGACTATTTATTTTGGA

GTTTAATTTATGATTGAACTTCATGATTTTCTACTTAGACTCTGGAAGAAGCAATTGCCTTGAACAATTCTGTACCTCAAGGATTAAGTAGTTCAATCTT

TACCCAAAGACCTGGAACTATATTCAAGTGGATTGGGTAAGTACTTACAAACTTATTTTTGTCCTATCCTTTCGATCTCTGCTGATGAGTTTGGCCTACA

CACTTCAGTACTTGACTAACACTCCTATAAGATGTTTGTGGTGATTTATAGTTGCATAAATAGTATATTTTACTTGTTCATGGGTGTGAATATGGTGCAG

GCCACGAGGTAGTGATTGTGGTATAGTGAATGCAAACATACCTACAAATGGAGCTGAGATTGGTGGTGCCTTTGGTGGAGAAAAGGCGACCGGTGGTGGC

CGTGAGGCGGGAAGTGACTCGTGGAAGCAATACATGCGGCGTTCTACATGGTAAGTCTTGTTGAATTTATGATGTTTCATTTCTTGGTTATAAGTTCTAT

TAATGACATTATAATTGCTGTCCTTGGCAGTACCATCAATTATGGAAGTGAACTACCATTAGCTCAGGGAATAAACTTTGGCTAGAAAGCTATCCTTGCA

AATGAAACTGCACAAATAATGCGATTTTGCAGCCCGAAAGAGAGATGTTAGTCTGGCTGACATGGGTTCAAAGTGTTGTATTAACAAGATCTGCCTGCTG

TAAGGATGGTACCTTCACATGACAGTTTCAATGTATAAGATATCTTAAATCTTAAATAAATTTAGCTGCAAAAGTTCTTATTTCCCAGTCAAAATGTTTG

ATGTTT

>Glyma.05G033500 | Chr05:2908499..2912457 reverse

ATGAGCATCCCAATTCCCCATCGGCAGTTATTCATAGACGGAGACTGGAAAGTCCCCGTCCTCAAGAATCGGATTCCCATCATCAACCCTTCCACCCAAC

ACATCATCGGTTCTCTTCTTTCCCATTCTCTGTCTCTCATCAGATCTCAATCTCAATCTCAATCTCCTTCCTTGTCTTCTCAATGCAGGGGATATCCCAG

CAGCTACTAAGGAAGACGTTGATCTCGCTGTCGCTGCCGCCAAAGCTGCCCTCTCCCGCAACAAGGGCGCCGATTGGGCCTCCGCTTCCGGCTCCGTTCG

GGCTCGCTACCTCCGCGCCATCGCTGCCAAGGTTCCTCTTATTTCACTTTTATTCTATTTTCGCTCTTTTCCTTATTTCCATTATTCTCCCTCCCTCCCT

CGCAGATCACCGAGAAAAAGCCTGAACTAGCAAAACTCGAAGCTATTGACTGTGGAAAACCGCTCGATGAAGCCGCCTGGGACATCGTAATCTCTCACAT

CCCTAAATTGCAAGCAAAGAAACACCGATTAAAGTTGTATCACTGTGTGTTCGGTTATTTGTTTCTGTAGGACGATGTTGCTGGTTGCTTTGAGTTCTAT

GCTGACCTTGCTGAAAAATTGGACGCACAGCAAAAGGCTCATGTGTCTCTTCCCATGGACACATTCAAGAGTTATGTTCTTAAGGAGCCGATTGGAGTCG

TTGCTTTAATAACTCCTTGGTACTACCTTCTCCTTCTCTCTCTCTCTCTCTCTCTGCTCTTTTTTAACTGAATCCGATGCTTTACTTTAATCATTGCATT

GCATTTGTGAATCATCATCCTCTGTTGATGGCGTTTTAGGAATTATCCTCTGTTGATGGCTACGTGGAAGGTTGCTCCTGCTCTGGCGGCCGGCTGTGCT

GCAATATTGAAGCCCTCTGAGTTGGCATCTGTGTATGTTTTTGTTCTCCATGACTGTCTGTTGCTGTCTTGCTTCTTTGTTTTGTTTTGTTCGTTGCTTG

GTTTCTTCTCATTCTATGTTGTCATGTGTTGTTGTGTGCAATTTTTAGGACATGTTTGGAGCTCGCTGAAATTTGCAAAGAAGTCGGGCTTCCTCCTGGC

GTGTTGAACATTCTCACTGGATTAGGACCTGAAGCGGGTGCTCCTTTAGCAGCTCATCCCGATGTAGACAAGGTTCAATTTTTAAAAGAATTGAAGCTGT

ATGGGGCTATATCTTGTTTTTATGTCTCACATTGTTGCACACAAGGTTTTAAATTGCAGTCACGGTCGCAATTTTGTCAATCCTTCAATTACAAACAGAT

GCATGTGGTCACAATTGTGGTCGCGGAAAGCCAAAAAAAACTTTATGTCCCAGCCAAAATTGCGGCCACGCGCTGTTTTTGAAAAACCTCGTTTGCGTGT

ATCATTACTGAGATTTCTTTGAGTTTGCAATGCTAGTTTGACATTGTGGCCGTAATTTTTCCAATAGATTGCCTTTACTGGAAGCTCTGCAACTGGGAGC

AAAATTATGACAGCTGCAGCTCAGCTGATCAAGGTATTTTTGAGCAGTTATCTGTAAGTTATGAACAGTTCGTGTTTCCATTTGTTTTGATAGAACAAAG

AACAAATAAAGGCATGCTCAGAAAGAGATAGGAACCCCTTTTTTTCCCCTTACATGTTTTAATTGTGTTTTCTTCATGAATCACTCTAACATAAAATGTG

TGGTTTCTTGGCAGCCTGTTTCACTAGAGCTTGGTGGGAAAAGCCCAATCATTGTTTTTGAGGATGTTGACCTTGACAAGGGTCAGTGACTTCGCAAGTA

TAGTGATTTAGTTATGGACAGCACTTGTCTACTTGCATCACAAAATCACATTTCCTTAAATACTGAGTGAGGATGAGTAATTTTTTTTCATCTATTTCCT

ATTGCATGATTTAGATCCTTTCTCCATGATTTAAACAGCTGCTGAATGGACCATATTTGGTTGCTTCTGGACAAATGGTCAGATATGCAGTGCAACTTCC

CGCCTTATTGTACATGCAAGTTTTGTCTTCTTTAACTTCAACTGTGGTTGAATTTTTTCAGCACCTTGATAGAAATTATGTTGGCATTTATCTTCAAAAA

CTAAGATACTCTCATTTTTCAGGAAAGTATAGCAACAGAATTTTTGAATAGGATTGTGAAATGGGTCAAAAACATCAAAATTTCTGATCCCTTGGAAGAA

GGTTGCAGACTAGGCCCTATTGTTAGTGAAGGACAGGTATATTTTCTGTTTCTCATTTCTGTATATCTTATGCATTTACGTCCTACTCTCTTTCTAAAGT

AAAGGGCAGGTATGCTTTGGTATGTTCAAATGCCCTTATTCAATATTCAAAATGAGAATGTGATTAATATGATATCATCATTAATTATGAATTATGATCA

AGCTTAATGTAGTATGGAATAATGCATAAGCTTCCTTAGAAGAATTATTGGTTGGCTGGCTATATATATATTATGAGAAGGAGTCAGAGAAGGAACCTTC

TCCTGGTTGTTGTATATGTTCCTATGATTTAGCTTTCTCTCTGTTCTTATAACTTAAAACATCTTATTGATATAAGCATATTTTTCTTGAACAAAGATTA

TCTAATATTTATATTATATGGTTCAGTATGAAAAGATATTGAAGTTTATCTCAAATGCTAAGAGTGAGGGTGCAACCATTTTGACTGGTGGGTCTCGCCC

AGAGGTATGATTATACATTTATAATTTCTTTGAAGGTGTGTTTTGGTTATGTGCTTCTGCAATTGTTGTGAGAATTGATTTTTTTTTTCTTTCTGAAATT

CAATGGCAGCATCTAAAGAAGGGATTCTTTGTTGACCAACTGTCATAACTGATGTAACTACCTCCATGCAAATTTGGAGAGAAGAAGTATTTGGACCAGT

TCTCTGTGTAAAAACATTTAGCACTGAGGAAGAAGCTATTGATCTAGCAAATGACACTGTGTGAGCTGATGTTTTCTGTGAACTGGCGACATTAACTTCC

TCTGTAAAAAAAAATTTCCCTTCATTTTTGGTTTGATATCTAAGTTATATACTTTAACCTTCCACAGATATGGCTTGGGTTCTGCTGTAATATCAAATGA

TCTAGAAAGATGTGAGCGCATTACTAAGGTGAGACTAATCCTAGGTTATCACAGAAAACAAAAAATGATTGGATTGTTTCTGCAGCTAAATAGTTCATCT

TGCTTCAATGCAGGCTTTTAAGGCTGGAATTGTGTGGATTAATTGCTCTCAACCATGCTTCACTCAAGCCCCATGGGGAGGCATTAAACGCAGTGGTTTT

GGTCGTGAATTAGGAGAATGGTATGGTCCTTATTTCCTAAATTAACAAATGCAAGTAACCTCAAATACCTTTCTTCTCTTTGCCTGGATATCATGTCTAA

CATGCAAAAGAAACTTACATTTATAAGTTGAAGTCCATGGATTGTTGAATGCTCCTGTACCACTCAACCGTTGACAGTTTGTTGACCATCAGATTTTGTA

CAACCAAATTTAATCAAAAGGTCGATAAAGCTGTGTAACATCGTGGCACTGGTGCAGCTTTGCACTTTTTAGTTGGAGTAATTTTAAATCATAGGGATTG

TGATGCATTGACACATTTAAATTAATAAACTAATAATAAAAAAGTCACCTACGATTTGAGTGAATTTATTCTAAGTTGTCACGTTCCTTTGGTTTGAAGC

TAGCATAAAAAATGAATAGGGTCACAGAGACAGTAGATTAAACTCACGCACAATTGCAAAAGAGAAAACATACCTTCACCTCGATCATTCTCAGATATGG

TGTGGAAAATAAAAACTTTTAAGCTTTTATTTGAGCTTCTGAGTAATTTTTCTTATTAACTTTTTCAGGGGACTTGATAATTACTTGAGTGTGAAGCAAG

TGACCCAATATATCTCTGATGAACCGTGGGGCTGGTACCAGTCTCCTTCAAGGCTGTGA

>Glyma.06G186300 | Chr06:16211217..16217224 forward

AAAAGATCTGTGATGACTCATTAGCAAGAACAAGACGTAGCCTCAATCTCATTATTTATATATATTATCATGTAGCTGCATTATTATTGATCCAATCAAG

AATACAATAACATAGCATAGAGTGAGAAGTGAGAAGAGAAAAATGGCAATCTCCATACCCAGTAGACAACTTTTCATAGATGGAGAGTGGAAAGTCCCTC

TTCTCAACAATCGGTTTCCGATCATCAACCCCGCTACTGAAGACATCATAGGTTCCCTCTCTAACTTTCTTTTCATTATTTTTCCACATGACTTCTTTGT

TCTATTGTCAAAACTCGTGTCTAAACTCATTTATTATGTTTATGATTATGATATGATTTTTGACACGTGCATAGTACTTGTAAGTAATAAGTAAAAAAAA

AAAAAAATACACATGTAATTTTAAGATTTTGACTTGATGTGATTGGTGTTCAGGGCATATTCCAGCCGCTACTAAGGAAGATGTTGACCTTGCGGTGGAT

GCTGCTAAAAGAGCCTTTTCCCATAACAAAGGCAAAGATTGGTCTTCAGCTCCTGGCTCTGTTCGTGCTCGCTATCTTCGAGCCATCGCTTCAAAGGTTC

TAACGTTTTTTTTCACTTGAATATGTTTTTATTGGCAGAGGGAAAAGATAAAATTATATATGAAGTTATTGAGTAAGTTGCATCAACTAGTATATTGTTT

AATAGCTTTCCATAGAATAACTTTATCTATTGGATTAAAAGTTCTTTATCATGTATACTATAACAAAATTTTATATTAACAAAAATTTGTTTAATATTCT

ATTAATTTATATCAAATTCATTGAATATATATATATATATATATATATATATATATATATATACTTTTTAACATATAAAAAATAAAATTATTTAATCATA

TATTTATAGATGCTTAATTTTGACTAAATTTAATACAAATAATCAAGATTGTTGTTTGAGGGTGAGTCTTGGCATAAGAGTAAAGTTGTGCCTTGGTGAT

TAGTTCGAATCCCAAAACAACATCTTTACATATTCAAGTGCATGGCTGCATATAATGACCCTCTTCTATACGATCTTCATAAGTTGTTTTTCTTTTTAAA

AAAAATCAAGATAGTTGTTCAACCCTTTAAATACATTTTGATGTGCATGTGTGGACACCTTACTGTGCTTTTCTTTATCACAGATAACAGAGAAAAAGGA

TGAACTAGGAAAGCTTGAAGCAATTGATTGTGGAAAACCACTAGATGAAGCACTGGCAGACCTGGTGAGTGGTGACATTTAACTTTGGGAAAACAACAAC

TGATGTTACCATGAGCTTTTTTTGCTTATTTTTATCTGTGACTTTATCATGTAGGATGATGTTATTGGTTGTTTTAACTACTATGCCGAGCTTGCAGAAG

GATTGGACGCAAAGCAAAATGCTCCTGTATCCCTTCCTATGGAAACCTTCAAGAGTTATGTTCTCAAGGAGCCCATCGGAGTTGTTGCATTAATTACTCC

ATGGTATTTGCTGTTTCTTCATTTTAACTGCTTATGCATTTCAATGTAATTTACTTCCATGAACAATCTTTATAAACTGAACATAGGTCCATAAGTGAAA

TATTATGCATTAATTAGCTTTTATCTAGTGCATACTGGTTATAGAGGCCAGGCTGCATTGACTTAGATTCTTATCAGTTTACTTGCATAGGAAATTGATG

CACTCACATTATCAACGATGTCAATTGCTGATTGAATGTGTTGAATAGCTAATGAAATTATATGATTTATGTTATGCATTAGGAACTATCCTCTATTGAT

GGCTACATGGAAAGTTGCTCCTGCTCTAGCTGCTGGTTGTACAGCAATATTGAAGCCATCTGAATTGGCATCTGTGTATGTTCTCATTAACTATTGTGCA

ACCTTGATTATTTGATATGTTGGTTTCTTATGCTGTCTTATCATTTACAATTTTAGGACCTGTTTGGAGCTGGCTGAAATATGCAGAGAAGTTGGCCTTC

CTCCAGGGGTATTAAACATTGTTACTGGATTAGGCAATGAAGCTGGTGCTCCTTTGTCATCTCATCCTGACGTAGACAAGGTTCAACCGATTTTACTTAT

TAGTAGAATCAAAATTATATGGAGATATATTTCATTCTATATATTGCAAGTATTGTTACTTTGCTATGATTTTTAAAAACTACTTAGCCATTGTGGCTGA

TGTGTAACTTTTCAACAGATTTCCTTTACTGGAAGCTCTGCAACTGGAAGCAGGATTATGACAGCTGCAGCACAGCTAACGAAGGTATTTTTAAGCAGTA

TTATCTGCAAATTATGTACAATTTATATTGTCATTCTGCTTTAAAAGATATAATAGAGGATACTAAGTAACAAGCATAAGATGAAATGATAGAAATCCTT

TTTATTTTTCCTTTCATATTTTCATTCTCTTTTCTTTGTGAGATCGTGTAATATCCAATTTGGGGTTTCTTGTTGGCAGCCTGTTTCTCTAGAGCTTGGT

GGAAAAAGCCCAATCATTGTTTTTGAGGATGTTGACCTTGATAAGAGTAAGTGCCTTTTATTTTGTTATGGACAACCTATGTTTTCTTGCATTGAGGAAT

CATATTTCCCATGAAACTGATTGAGTGGTTGTCGATGAGTAAAATTTGGGTACCTAGAACCTATTACTGAAACCAAAATCCAAATAGACAGTCATATACC

ATTTAACATTTATTTTGCTATTTTCCTTGATTTAAACAGCTGCTGAATGGACCATCTTTGGCTGCTTCTTTACAAATGGTCAGATATGCAGTGCAACATC

TCGCCTTATTGTGCATGTAAGTTTAACATATTGATTTTGATTCATCCATAACACATTTCCCTCTTCACAAACAAGTATTTGTAGCTTACTTTTCTTACTG

TGCTGTCTAAACAGGAAGGTTCTGATATACTCAGATATCATTTTGTCTGTAAACTTTGTAGCAATTGCATTGGCTGAGTTTTGAACATTGATAAAGTGAG

AGATGGTTTTACTGTTTCAGGAAAGTATAGCAACAGAATTTGTGAATAGACTTGTGCAATGGGCCAAAAACATCAAAATTTCAGATCCCTTTGAAGAAGG

TTGTAGGCTAGGTCCTATTGTTAGTGAAGGACAGGTATGTTTAATGTTTATCACTTTTGTATTTACTATATATTTGCTTAACTTACTCCCTCCTGCTTTT

ACATTGTAAAAGAATGCTTTTTTCATTTACTCCTTTATAGTATTAGGAAAAACCATAAAAAGTGCAAATGTTAAACACTTTGTCTGGCAATCAGTATTTG

TCTTTGGAATACAAAAAATTTAATTTATATTTGAAATATATCCGAATATAGGTCTAGCAGAAAAAATCTAACATATATCAGCAATTAGTTAGTACAGACA

CACTTGGAAGAGTTTAGTGATTTAGCAGGAAAGCAGTGCAAATATTCAGCATAGAAATGCCTTTAGAAGTCAAAAATATAAGCATATTTTGGATTATTTT

GAAGGAAAAATAGATGCTGTTTGCTTGAACATTTTCTGAAATCAAGGATTTCTTTGTGTGACCATTTCTCTCTCTAGAAGTAACTGGTCACATCTCCATA

AAAACTTAAGGTATATGCTTCAAGTCCAAACAGGGGAAACTTAAGGTAGTATTCCCTTGAACTTTTCCAATAAAAAAGAAAAAACTGCAGACTACAGAGA

GATATGCTTCATCTGAACATGGTTCATTCGTTGAATGCTTCATTATGTTAAAAATTGTCAAAGGAATGGATGCAATTATTACATATGTTACTTTTACTTT

ATGTTTTAATTCATAATTTAGAAGGAGAAACTGTCGGTTGTAATGATAATGTAATTTCATTGCTATTTTTACTTACTCTTTCTGATTAATGTACTTCTGC

CTTTAACGAAGATATGTCATGTTCTGGTTGTTACACCTTGTTTCATTTCTTCCTCTAATACGCTGTAGTTTTGGGTTTAAGATATTGTTTGAAGTAGATA

TGTAACACTGAAATCTACTGCTAACGTAGACTTCTGTATTTCTGCTTGTGTTTATTCAAATGCCTTCGCCATCATCCAAAATGGAAATGTATGTTAGTGT

GATGTCAGTGATTATGGTTGCATGTAGTATATATTAATCGAAGTTATATGAAAAAAAATCAAGTGGTTTGTTATATGAAGAAAATAAGTTTTTCCTGACA

TTGTTAATGCACTTACTTGTCTTGAACCAACATTTTATCATATCTAATGTTTGGATTATATGGTTCAGTATAAAAAAGTGTTGAATTGTATCTCAACTGC

TAAGAGTGAGGGTGCAACAATTTTGATTGGAGGGTCTCGCCCAGAGGTATGATTATACTCTCTAGTATGTGTGCCCGGTTATGTATTGCAAAACTTGTCA

TGAGAAATGATAGATTTGTCAAATTTGATGGCAGCATTTGAAAAAGGGGTACTTTGTTGAACCAACCATCATAACTGATGTGACTACCTCGATGCAAATT

TGGAGAGAAGAAGTTTTTGGACCTGTGCTATGTGTGAAAACATTCAGTACCGAAGAAGAAGCTATTGAACTAGCAAATGACACACAGTGAGTTAATTATT

TTTCTGGAGGTTTAAGTATATTTTTTATTCCTTAAATTTGAGTCATCGTTATTTTTTATCCCCTAAATTTTTTTTGTTTTCTAATTTTGAACAATGTTCT

TTTTAGTCTTTTTTGACTGATTTTTTAATGCAGAAGGGACTAAAGATTATTTTTCAAAGATTGAGAGACTAAAAAATAAATTTAAATTTTAAAGGACCAA

AAATTTAAAATAATAACCTCAATTTGAGGAACCAAAAACATTTTAAAGCCAAAACATATTTAAAACTGTAATACTTAACGCCTGTAACTACTTTTTATTC

ACTTGTGCATTGATGCTAATCAATGTTGTATATGATCTGTTATTTCACAGCTACGGCTTAGGGTCTGCTGTAATGTCAAAAGATCTAGAAAGATGCGAGC

GCATATCAAAGGTGAGACTGATCCTAGATTATCATAAAAACTGCAAAACAAAAGGGTTTTATGTAAATTGAATGGTTAATATTTATACTTGTATTGCTTT

TTGTGCAGGCTATTCAGGCTGGAATTGTATGGATAAATTGTGCTCAACCAAGCTTCATTCAAGCTCCATGGGGAGGCGTTAAACGTAGTGGTTTTGGTCG

TGAACTAGGAGAATGGTATGAATCTTATTTCCTATATCAGTGAATGAGGAAGTCAATTTTAATGCCTTTAATCCCTCATCTAATAGTTATTTGTGGAAAA

TAAAAATGTGAATTTAATTGGAACACATTAATGGTGTAAATGTTTTTTACTTTATCATCCAAGCGTGGATTAATATAAAATTCAAGCTTGTGAATTTTTA

TCAAGAGTTTCACACTGTCATATACACATCACTATTGGTGTAATTTTTAAGGTAATTGTTGTACATGACCAGTTGGTGATTGGATGACGGTAAGACTATT

TTACAGAGCGGAAAAAATTTAGTAGGCATGTTTAGGATTATTTTTAATTGACTCACAATGTAAAAAACTTTATGCTGTAAACTCTAAAAATATTGGTTAG

GCTATATCTTGATTCTTGATTGTTTTTCATCTGAGAATCTGAATCTGGGTCTGAGTTTTTGTTGTTTATTATTTCAGGGGACTCGAGAACTATTTAAGTG

TGAAGCAAGTGACCAAGTACATATCTGATGAACCGTGGGGCTGGTATCAGTCGCCTTCAAAGCTGTGATATGAATTGCTGTTCTAAAATCTGGCATGAAT

AAGTTGTGGGGGACATTAATTTGTGATGATGATCTTAATTCGAGAAAATAATGTGCATATGTATAACACTGAAATTGTTCCATGGCCTTGTTAATGACGG

GCTGAGTTGATTTTATCTTATTTGTGATGATGATCTTCACGTTAGAACTTTTTTTTTACAATGTTTACATGTGATTTCTTAAATGTTTTTGTTGTGTTTT

CTAGTTAA

>Glyma.02G202500 | Chr02:38754600..38759102 forward

AAGTTGTCACACAACACCAAACCATCACCCATAAAAATTAAACCAACCATACTACACAGACACTCCTCCTAACCTTAAATAATTCTCCTCTCACCCTTAT

AAAAACACTCATTTTAACTCCCACTTTTGCATTTCCACCTATCTATCTATCCATCTATCTTTTCTAACATGGCTGCTGGGACTGGACTCTTTGCAGAGAT

TTTGGATGGAGATGCGTACAAGTACTATGCTGATGGAGAGTGGAAGAAATCTGCTTCTGGGAAAAGTGTTTCCATCATCAACCCCACTACCAGAAAGACT

CAATACAAAGTTCAAGGTAAATTAACCGTTAATATGTCACATGTTAGCACTTGGCACATCATTAACCCTTTCGTTTGAATCCCTTTATTTCAGTTCCTTT

CTGTAGTTTTTAGAGTGCATGTTACTGGAAGCTTGAGAATTCTAGTTAGTATTTGCTGGATTTTCTATGCATGTAGATATTAATTAAAATTATTGGCAAA

ACAATTTATGTGTAATTTTTTTAAAAATTTCGTTACAAAATTTTTTGTAGCCACAGAATTAGTGAGTAAAAAGCAATTTTTGTCGTTAAATTTGGTTAAT

AATTCGTTGATTTTTAAAGCTCAAGTTCTTGTTTTGTGTAACCAAACTAGAACATGAAGCAAGATTCTCCTTTTACAATAACAGCAGATAAGTGTGTCTG

ATAAGCTCAAAAAATGATGTGCATGCAGCTTGTTCACAAGAGGAGGTTAATAAGGTCATGGACTTAGCAAAATCTGCACAAAAGTTATGGGCAAAGACCC

CACTATGGAAACGTGCTGAGCTTCTTCACAAGGCAGCTGCTATCCTTAAAGAGCACAAAACTCCAATTGCAGAGTGCCTGGTGAAAGAAATAGCAAAGCC

AGCCAAAGATGCTGTTATGGAGGTGCATATTGTGTCACCGGTTCTGTTAAACGAGCTAAATTATTGTTCAGGACTATTATTATTTTGAATGATGAAGATA

ACTAACTGTTTTTTTTTTTTTTCCTTGAAGGTTGTAAGATCTGGGGATCTGGTTTCTTACACTGCTGAAGAAGGTGTAAGGATTCTGGGAGAGGGAAAGT

TCTTGGTGTCGGATAGCTTTCCTGGAAATGAAAGGACAAAATATTGCCTCACATCCAAGGTTTATTCCATCACTGAGTGTTGCTTGACTTCACAAGTTAA

AATTCATTAATAACTGACTTGTCACAACTTTTTGTTATAAAATATACATTTTAACATGTAAAAGGTTTTCTTAGGTGTGAATTTCAGGACAATAAGTGGG

TAAAAGAATTCTCACATTTAGGGGGGGTGGGGTGATGGGGTAGGGACTTTATAAGAAAAAAGACCCATGAAGAACAGTTGCTTTTAAGAAATGGTATGGA

AGCAATAAATAGTAATATCAAAGAGAGAAGGAATGCTTTCCTATACTCTTGTATAGCATGTATTGGTAGAGAAAGTACCAAAAAATAATCAGGAAGTCAA

GGTTAAATACACTTTATCCATTAAAATGCACTAGGGAATCATATTCTTGTTCACCAGAGAAGTTCTCTGCTTCTAGTTCAAGATTTTCCTTAATGAAGGG

TTTTGCCTTTGACGCACATTTTGATTAACTGGTGTCTGGTGTCAACGTATAAGAATAATTTATAAAATAAACAAGGGAGATAAGTGTAATTTTTCAATTA

AGAAAGTTATAAGTATAACTAGTCCAAACATCAAGGGAGGTCAATGTAATTGGCTCTTATGACAAATAAAGCAGTCCCTGCTTTCTAGAGAGTGGACAGC

ATAGATTATAGTAATTCCAGAACAGATAGTCAGCTACATCTGGAAAATCTATATTTTATTAAGTTACAATTACAAACAAACACAATATCAAAACCCTGAG

TTCATAAAGTAGTGTAATCTCTCCATCACATTGTATTATCTTAGTAACTTCTAATTAGCATATTAAATGATCATATGATGAGGTGAAATGCCAAGTGTTA

ACATGTTTTTCAGATTTGGTTTTTGATGTGATCTAAGAAAGTTTCATTTGATTTTCAGATTCCACTAGGAGTGATTTTAGCCATTCCACCTTTTAACTAT

CCCGTCAATCTTGCTGTTTCCAAAATTGCTCCTGCCCTGATTGCTGGAAACTCAATTGTGCTCAAGCCTCCAACTCAGGTATTTATATCTTTGATGCGAA

ACTCTTCAAAATCAAAGACACTACACGTTAGATCCACACTCTCACAGTAGCAACTAGCTCAAATCATAGAAGTGCTAGAACACGTGTGTTGCAGTTAAAG

AAACCACAACAACAATAATTTATAATTTCATTTCAGGATTTTTATGTCCTACAATTTCCTCCCTTAGTTTCTTTCTTGTAAATGTTTGTTTAATGATGCA

TTTCAGGGAGCTGTTTCTGCTTTGCACATGGTTCACTGCTTTCACTTGGCTGGTTTTCCCAAAGGCCTAATCAACTGTGTCACTGGAAAAGGCTCAGAAA

TTGGTGATTTCCTTACAATGCATCCAGGAGTGAACTGTATAAGGTTCCTTTTTAAATGATGCTGTGATAGCATGGTACTGTATATCCCCAATTTCCCATG

TGTAAGTAATTTTTCCTCTACTGACTTATGACTATGCTGTTGGTCAGCTTTACTGGTGGTGATACTGGCATTTCAATTTCAAAGAAGGCAGGCATGATTC

CTCTGCAAATGGAGTTAGGAGGAAAAGATGCCTGCATTGTACTTGAGGATGCTGACCTGGATTTGGTTGCTGCAAACATTATAAAGGGAGGCTTTTCATA

CAGGTAATGGCACATCTGAATTAGTACTTCTGGGGCAAGGTTTCCTTTTCTGCATTGATTTGACAGAACTGCCAACCAATATTAAGGAAGACAATTAGAC

ATCCTACACACTTTGCATGCATCCTTGTTTTGCATCCTTCTGTGAAATAAACAGTTTGTGAGATATTGCTGCTAACATCAGATTATTTTACCATATAAGA

TGAATATTGCATGGTTAGAATTTATATAGATCTAGAAACTAAGCAAATATACTTCCATTTCAGCTTCATGTGCTTATTTTTTTATGTTATGGCTCTATGT

AAAACAGTAATCCAGATATAATTTTGCGGAGAACAAAATTCTCAAGGATTTTTCTTTGTATAATGGATACTTGAATTATAATTCTTTTTTCTTTTGCAAC

AAAAGTGGTCAGAGGTGCACTGCTGTTAAGGTTGTCCTGGTAATGGAGTCCGTTGCCGATGCTTTGGTTGAGAAGGTGAAGGCTAAGGTGGCAAAACTAA

CTGTTGGACCACCAGAGGATGACTGTGATATCACCCCGGTTGTGTCAGAATCATCTGCCAATTTCATTGAAGGCCTGGTCTTGGATGCTAAGGAGAAAGG

AGCAACATTCTGCCAGGAGTACAAAAGAGAAGGCAATCTTATTTGGCCTTTGCTACTGGACAATGTTAGGCCAGACATGAGAATCGCATGGGAAGAGCCA

TTTGGACCAGTTTTACCAGTAATTAGGATCAATTCAGTTGAAGAAGGAATCCATCATTGCAATGCTAGCAACTTTGGACTTCAGGTAAATAAAATTATAG

CCCTCAAACTTGAACATTTACTTACCCTTTAAAAAGTACACAATATTTTCTTATAATACAAGTAATTTGTATCAACACATTGAACTTGTGTAGGGATGTG

TCTTCACAAAGGATGTCAACAAAGCAATAATGATCAGTGATGCAATGGAAACGGGAACAGTTCAGATAAATTCCGCCCCGGCTCGTGGGCCAGATCATTT

TCCCTTTCAGGTGAACCTACCCTTCAACCAACTAAATTCTGCTATTTCCAAAACTTGGAAACGAACCAAAAGCATTTTTTTTCTTGTACAATGGTCGCAC

TGAGGTTCGAACTAAAAAAAAACATTTTTTTTCTATATCACCAATCTATTATTAACAAATACTGTTCTGACAGGGTATAAAGGACAGTGGCATTGGATCT

CAAGGGATTACCAACAGCATAAACATGATGACCAAGGTCAAAACGACTGTGATCAACTTACCCAGCCCTTCCTATACTATGGGCTAGTAGTGTAATAGTT

ATACACATACATGCATGGAGGAAAATCAATTTATATCATGTTCCATAAATCATAGTGAACGTTCAAAACGTATGAGTTCAACATTGCCTTCTCTTTTGCA

GCAGGGTATCATAGAGATGAATAACGAGTTTGATTGATCTGTCACGTAGCTACTTCACGTGACCACTTGTCGTACTCATATTTAGCATGATATGAATCTG

AATTTTTATCTTGTGCCTTATCATTAAGTAAGGAATATAATTGTGTTCCAGTTAAATTGAATGTCAAATCAATGAAAGGAGGGGGAAAACATTGTCTTCT

GTTTCCATTTTGTCCTAACATTTTTTTGCAATCCCTATTCCCTTGTATCCCTTTCTGTTAATTAAAGCACAAAAATATAAGGCACAACTGTCCTGAAATC

CTC

>Glyma.17G075300 | Chr17:5908293..5912735 reverse

AAAAATCAAAGAACACTTGGCAATCTCAGAAAACCGATGCGTGGCTTACCTTCTCCTTACATAAACGACGCATGTTGTCACACAACACCAAACCATCACC

CAGAAAAATTAAACCAACCATACTACACCAGACACTCCTCCTAACCTTCAATAATTCTCTTCTCACCCTTATAAAAAGAAACTCATTTTAACTCAGACTT

TGCATTTCACCTTATCTATCTATCTTTTCTAACATGGCTGCTGGGACTGGACTCTTTGCAGAGATTTTGGATGGAGATGTGTACAAGTACTATGCTGATG

GAGAATGGAAGAAATCTGCTTCTGGGAAAAGTGTTGCCATCATCAACCCCACTACCAGAAAGACTCAATACAAAGTTCAAGGTAAATTAACCGTTAAATA

TGTCAAATGTTGATTAGCACTTGGCACATCATTAATTTCCTTTTAATTTGAAGCCCTTTATTCCAGTTCCTTTCTATGTTTTTAGAGTGCATGTTACCGG

AAGCTTGAGAATTCTAGTTAGCATTTGCTGGATTTTCTATGCATGTAGATCGATATTAGTTAAAATTATTGACAAAACAATTTATATGTAATTTTCAAAA

AAATTGTTACTAAAATTTGTGACCTCAGAATTAGTGAGGAAAAAGCAATTTTTGTCACTAAATTTGGTCACTAATTCATGGAGTTTTAGAGCTTAAGTTA

TTGTTTTGTTTAACCAAAAACTAGAGCATGCAAGGTTGTCCTTTTTGTTTTGTTTACAATAACAGCAGATAAGTGTGTCCGATAAGCTCTAAAAATGATG

GGCATGCAGCTTGTTCACAAGAGGAGGTTAACAAGGTCATGGACTTGGCAAAATCTGCCCAAAAGTTATGGGCAAAGACCCCACTATGGAAACGTGCTGA

ACTTCTTCACAAGGCAGCTGCTATCCTTAAAGAGCACAAAGCACCAATTGCAGAGTGCCTGGTGAAAGAAATAGCAAAGCCAGCCAAAGATGCTGTTACA

GAGGTACATATTGTGTCGCGGGTTATGTGAAACAAGCTAAATTATTGTCCAGGACTATTATTTTGAATGATGAAGATAACTAACTTTTTTTTTTTTCCCT

TGAAGGTTGTAAGATCTGGGGATCTGGTTTCTTATACTGCTGAAGAAGGTGTAAGGATTCTGGGAGAGGGAAAGTTCTTGGTGTCGGATAGCTTTCCTGG

AAATGAAAGGACGAAATATTGCCTCACATCCAAGGTTTATTTCAACACTTGTTTACTATGAAAATTACTTAACTTCACAAGTTAAAATTCATTTATAACT

GGCTTGTCACATTTTTTTGTTGTAAAATATACATTTTAACATAAAACGTTTTGTTAAGCGTGAATTTCAGGACTGGAATTGGGTAAAAGAATTCTCACAT

TTAGGGGAAGGGGGGTGGGGGTTGATGGGGCCGAGACTTTAATTTATAAGCAAAAAGACCCATGAAGAAGGGGTGCTTCAAAGAAATGGTATGGAAGCAA

TAAATAGTAATATCAAAGATAGAAGGAATGCTTTCCTATAGTCTTGTATAGCATGTACTGGTGGAGAAAGGACAAAAATAATCAGTATTTCAAGGATAAA

TACACTTTATCCTTTAAAATGTACTAGAGGAATTAAATTCTTGTTCACCTACGAAGTTCTCTACTTCTAGTTCAAGATTTTCCTTAATGAAGGGTTTTGC

TTTTGGTGCACATTTTGATTAACTGGTGTCTGGTGTCAACGCTTAAGAATAATTTAAAATAAGGGAGATAAAGTTATAAGTATAACAAGTCCAAACATCA

AGGGAGGTCAATGTAGTTAGTTCTTATGACACAAATAAAACAGTCCCTTCTTGCTGGACGGTGGACACCATAGATCATAATAATTCTAGATATTCAGTCA

ATCCAGAAATTTATTATGGAAAAGTTCTTCAGTCATTCCAGAACAGACAGTCAGCTACATCTGGAAAATCAATATTTTATTAAGTCACAATTACAAACAG

ACACAATATCAAAACCCTGAGTTCATAAAGTAGTGTAATCTCTCCATCACATTCCATTATCTAAGTAACTTCTAATGACTAGCATATTAAATGATCACGA

TGAGGTAAGTTGACAGATGTTAACATGTTTTTTAGATTTGGGTTTTGATATGATCTAAGAAAGTTTCATTTGATTTTCAGATTCCACTTGGAGTGATTTT

AGCCATTCCACCTTTTAACTATCCCGTCAATCTTGCTGTTTCCAAAATTGCTCCTGCGCTGATTGCTGGAAACTCCATTGTGCTCAAGCCTCCAACTCAG

GTAATTATATCCTTCATTGTGGAACTCTTCAAAATCAAAGGCACTACACGTTAGATCCACACTCTTATAGTAACAATTAGCTCATGTCATAAAAGTGCTA

GCATATGTGTGCTGCAATTAAAGAAACCACAACAACAATTATTCCATTTCAGGAACTTTATGTCCTATAATTTCTTCCCTTCTTTTCTTTATTACTCTTT

CTAAATGTTGGTTTAATGATGCATTTCAGGGAGCTGTTTCTGCTCTTCATATGGTTCACTGCTTTCACTTGGCTGGTTTTCCCAAAGGCCTAATCAACTG

TGTCACTGGCAAAGGCTCAGAAATTGGTGACTTCCTTACAATGCATCCAGGAGTGAACTGTATAAGGTGACTATTTGTTCCTTCTAAATGATATGATAGG

ATGTTGCCACACATCCTCAATTTCCCATGTGCTAGTAATTTTTCCTCTACTGACTTGTCACAATGCTGTTGGTCAGCTTTACTGGTGGTGATACTGGCAT

TGCAATTTCAAAGAAGGCAGGCATGATTCCTCTGCAAATGGAGTTGGGAGGAAAAGATGCCTGCATTGTACTTGAAGATGCTGACCTGGATTTGGTTGCT

GCAAACATTATAAAGGGAGGTTTTTCATACAGGTAATGGCACATCAGAATTAGTACTTATGAGGCAAGGTTTCCTTTTCTGCATTGATTTGACAGAACTG

CCTACCAATATTAAGATAGACAATTAGACATCCTCCACACTTTGCATGCATTCTTGCTCTTTCTGTGAAATAAACAGTTTGTGAGATATTGCAACTCACA

TCAGATAATTTTACCATACAAGATGAATGTTGCTTGGTTAGAATTTATATAGATCTAGAAACTAAGTAAATATACTTCCATTTCAGCTTCATGTGCTTCT

TTACATAATGGATACTTACTTGAATTATAATTCTTATTTTTTTTTCAACAAAAGTGGTCAGAGATGCACTGCTGTTAAGGTTGTCCTGGTAATGGAGTCC

GCTGCTGATGCTTTGGTTGAGAAAGTGAAGGCTAAGGTGGCAAAACTAACTGTTGGACCACCAGAGGATGACTGTGATATCACCCCGGTTGTGTCAGAAT

CATCCGCCAATTTCATTGAAGGCCTGGTCTTGGATGCTAAGGAGAAAGGAGCAACATTCTGTCAGGAGTACAAAAGAGAAGGCAATCTCATTTGGCCTTT

GCTACTGGACAATGTTAGGCCAGACATGAGAATTGCATGGGAAGAGCCATTTGGACCGGTTTTACCAGTAATTAGGATCAATTCAGTTGAAGAAGGAATC

CATCATTGTAATGCTAGCAACTTTGGACTTCAGGTAAAATGAAATTATAGCCCTCAAGCTTGCACATTTACTTACCCTTTAAAAAAGTATACAATGCTTT

CTTATAATAGAAGTAATTTGTATCAACACACTGAACTTGTGTAGGGATGTGTCTTCACAAAGGATGTCAACAAAGCAATAATGATCAGTGATGCAATGGA

AACGGGAACAGTTCAGATAAATTCTGCCCCAGCTCGTGGGCCAGATCATTTTCCCTTTCAGGTGAACCTACCCTAAAACCAACGAAATTCTGCTATTTCC

AAGACTTGGAAACGAACCAAAACCATTTTTTTATGTACAATGGTCGCACTGAGGATCAAAGTAACAAAAAACATTTATTTCTATATCACCAATCTATTAT

TAACAAATACTTTGCTGACAGGGTATAAAGGACAGTGGCATTGGATCTCAAGGGATTACCAACAGCATAAACATGATGACCAAGGTCAAAACGACTGTGA

TCAACTTACCCAGCCCTTCCTATACTATGGGCTAGTAGTGTAATACACACATGCATGGGGGAACATCATTATATAGCATGTTCCATAAATCATAGTGAAC

GTACAACCGTGTGAGTTCAGCATTGCATTGTCTTTTGCAGCTGGGAATCACAGAGATGAATAACGAGTTTGATTGATATGTCGCATAGCTACTTCATATG

TGACTACTTGTTGTACTCGTATTTAGGATGATATGAAACTGAATTTTATCTTGTGCCTTATCATTAAGGAATATCATTGTGTTCCAATTAAATTGAATGT

CAAATCAATGAAATGAGGGGGAAAAACATTGGCTTCTGTTTCC

>Glyma.17G218200 | Chr17:36839844..36844952 forward

ATTTTTTTAGTTCCAGCTAAACAGCCTGAAAGCACACTGATCGGCCAAGGACTGAAATAGACTAAGCACTAAGTATTAGCAACATTTATAGCTGTTCCTT

GACCCTGTCTGAATCCACTTCAATAATCATGTTATACTAATAATAATCACCAACCACATAATCAACATCAATATATATATACTCCTAAGCCAAAGTACAA

ACCTCAAACCAAATCGAATCACTTGTTAGTTCAACATGGCTGGCAGTGGCACCTTTGCAGAGATCATAGATGGTGATGTGTTCAAGTACTATGCACAAGG

CCATTGGAACAAGTCCTCTTCTGGAAAATTCGTTCCCATCATCAATCCAACCACTAGGAAGACCCACTTCAAGGTCCAAGGTATTGTTAACACACCCTTT

TGTAAATATAACATGAAAACCATGTCCTTTTTTTTTGTCTTCACCATATCAATTATTGGTTTTTGATCCAATACTTTGATTGGAAGAGAATTAATAGCAC

ACCTATGTGATCATTTTGTTTTTCATGTCTTTGTTGTTGGAATTCATGGTTTTGAGGGGTGGTGACTTTAAGATACTAATCCGCTTTAATTGAATGTCAA

ACAGCTTGTACACAGAAAGAGGTTAATAGGGTCATGGAATCAGCAAAAACAGCTCAAAAATCATGGGCCAAGACTCCACTGTGGAAAAGAGCAGAGTTGC

TTCACAAGGCTGCAGCAATACTGAAGGAGCACAAAGCACCTATTGCTGAGTGTCTTGTAAAGGAGATTGCAAAGCCAGCTAAGGATGCTGTCACTGAGGT

ATGTGCATGTACGAGTAGAAATTTTGATTGGGCTAGTATTTATCAAATTCAAGAATTTTTTTGTCACCTTCCAATATGCACATAACATGTTAGGAACTCT

TGATGGAAAATTAAGGGCTTATGTTGCATGTTTAGAAGATTTGGCTATTTGGTTCTTGGCTTTCACTTTACTTTTTAAATTCTATGATTTTTATGTAGGT

GATCAGATCAGGGGATTTGGTGTCATATTGTGCTGAGGAAGGGGTGAGAATTCTTGGGGAAGGAAAATTTCTGGTCTCTGATAGTTTTCCGGGCAATGAA

AGAACCAAATACTGTCTCACTTCCAAGGTATTTTGACCTTTTCTTTTAGAGGTGATGGCTCTAATACTTGGCATTGACTATGCTGTTTTTTTCTGTCACA

AAATGTTTCATGTCTCAAATTTTAGTCTCTAATACCATCTTAGAATTTTAGCTTAGAACATAACTTAATCCTACAAAACCAATTTGTAAGATGAGAGGAC

TATTCAATCTGTATAAGGACTATCCAAACCTTATAAGGCCTATATTGACCATATCTCTAGTTGATATTGGATCTCAACAATATGCTAGACCCTATGCTTG

TGATTGTAAATAAGGCATTATTTTGAGATATTTTCTGTGTGCAGATTCCACTGGGAGTTGTTTTAGCCATTCCACCTTTCAACTATCCTGTTAATCTGGC

TGTTTCAAAGATTGCTCCAGCGCTTATTGCTGGAAACTCCATTGTTCTTAAACCCCCCACCCAGGTATGCAACTATTTATAGAGCATATACTTATTAGTT

ATCAATAGTTATATAGTCCTTCTATTATTTAATTTAGATAAAATCTCAAGGGTTGTGTATGTTTTGAATGCTTGAGTCAGAAAACTTGCTTAGCGAGTTA

GCTCTTTATCTTTACAGGGTGCTGTTGCTGCACTACATATGGTGCATTGCTTTCATTTGGCTGGTTTCCCCGAAGGCCTTATTAGCTGTGTGACAGGAAA

GGGTTCCGAGATTGGTGATTTTCTTACAATGCATCCGGGGGTGAACTGCATAAGGTAATATGCACTTCTTTGGCTATGTTGTTATTGTTATCCTCCTCTA

CAATGGCTGGTTTTATTGCTTTCTATACAATAACTCACGGGATTCATAATTTGCAAATGTAGTTTCAACTTCCAACAAAATGTTCAAGCAAGATTAGATA

CAATAAATCATGCCTCATCTTTTGTATGGTTACATGATATATCAGTCTTTAGTTTCAAAAGTAATTATAAGATAATATGAAAAACTTAAAGTAACTTTGT

ACCAAATTATAGAAGAGTGCCTGGGAATTTTTAAGTTATTAGAGAGATGTTTTGTCATTGCATTACATCTGGAAATTTTTCCACCTAATTTACCTTAATC

TTCAGTTTCACTGGTGGGGACACTGGAATAGCAATATCAAAGAAGGCCGGCATGGTTCCTCTTCAAATGGAATTGGGTGGAAAAGATGCCTGCATTGTTC

TTGAAGATGCTGACTTGGATTTGGCAGCTGCTAACATTGTAAAAGGAGGCTTTTCCTACAGGTAACAATAGTATATAGCACCTTCAGAACGAAATTATAT

TGACTTCAAAAAGCTTTTAAGGAGAAACATCAACATTGCTTCATATCGTTCGGGTACTACTTCGATCCTTGGCGGAAACAATTTTTTGTTAGGTTTTATT

TACCTCTTGGCCAAACTCCGGATTAGTTAGAGTTCATTTCCCCTCATGAATAGGAGGTTAAGACCAAAAAATCCTTTAACAGATTGTCTTAGATTTTCCT

AAAAGAATTGTTATTAATCCTTTGAATTTCATGATTAATGTGGAACTTTCACATTTTCCCAAGTTTAAAACTTCAATAAGGGAACTGATTTATACTTCAC

AATTTTCTCACTGTGGCTGCATTAACTCCTATCACAGCATAGTCCTAAATCCTAACACAAATGCTGTGATTTTTTTATGTCAGTGGTCAAAGATGCACAG

CTGTAAAGGTTGCTCTGGTCATGGAATCGGTTGCCAACACTCTTGTAAAGAGAATCAATGATAAAATTGCAAAGTTAACTGTTGGACCTCCTGAGATTGA

CAGTGATGTCACACCAGTTGTCACCGAATCCTCCGCTAACTTCATTGAAGGGTTGGTAATGGATGCTAAGGAGAAAGGAGCAACATTCTGCCAGGAATAC

GTAAGGGAGGGAAATCTCATTTGGCCATTGCTGTTGGATAATGTTCGTCCCGATATGAGGATAGCATGGGAGGAGCCATTTGGACCAGTTTTGCCAGTTA

TCAGGATAAATTCTGTTGAAGAAGGAATCCACCATTGTAATGCTAGCAATTTTGGCCTTCAGGTATAGCATCAGTTAATCAATGTGCATGCATACATGCA

AAGGACAAAAGGGTACCATCATTTAAAAAATGGTTACACTTTTTTTTTCCCCTAATTTTCTTTTTACATGCAAAAGAAAGTGTCAGTTATAATCAGTTTT

TACTTGATGAATTTGAAACGTTGAACATTTTTTTTCAAAAGTAGGATGGTCTCGTGAATCCTATGTACCCTTGAGAGTTAAGTGAGATTGTCCAATTTTT

TTTCAACATATTGATGAATGATGATCACTTTAGAGTGTCACAGCATAATCTTGAGTGTGTTCTCAGTCAAAAACGCCATTTATCCATTCCAAGGCGGAAA

AGTTTTCATTCCTTTGTTTAATCCTATGTATAACGCAGGGATGTGTCTTCACAAGAGACATTAACAAAGCAATGCTGATCAGTGATGCAATGGAGACAGG

AACAGTTCAAATTAATTCAGCACCAGCTCGTGGACCTGATCACTTTCCTTTTCAGGTGAAACATTTGTCCTCCATCTTGCTAAGTTGCCATAAAAATTTT

GTAGGTAGTAATACCAATTGTAAAATTCCTTTTTTATAAGAAACAAATTCAAAAGTTTTTACAAAAAAAAAAAAAAAAAAAACAGGAAAACATTCCTCCT

TTATTCCCACACAACATCATTAATTGATGAGTTTAACTTATATACCCTCACATCTAATCATTTTTTACAGAGATTGATGGGTCATTGAGCTTCGGGTGTT

GCTAGGTGCACCCAGCATTATTGACCTTCGGGTGTTGCTAGGTGTACCCAGCATTATTGCTGGTGCACCCAACATTTTTTAAAAATACCAAAATTGTTCA

TGTATTTTTTTTGCATTTGTGATAAGTGTGCGTGAGGGTGGTCCGTAAATCGTATGGATCAAGTTGATCCGTAAGATTGATACGAATCAAGTTGATTCGT

AAGTTTGATACAGATCAAGTTGATCCGTATGTTGATATTAAGCATACGGATCAACTTGATCCGTAAGCCTTTTACAGTACTACACGGATCAACTTCATCC

ATAAAAGCCTTACGCATCAACTTGATCCGTAAAAGACTTACGGATCAAGGACGGATCAAATTGATCCGTAAAAGACTTACGGATCAAGTTGATCCGCAAA

CCTTTTACGGATCAACTTAAAAGACTTACGGATCAAAGATATTTTCGTCATTTCATCTTAAATGTTGGGTGCACCAACAATAATGCTGGGTACACCTAAC

AACACCCTTGACCTTCACCCGTTTCCTAGCATAAACCCGTGAAAGATTGTTTGGACCCAATGGCCTATCACAGATATTCAATAAGATATGATCACATTAT

CATGCCATCTTATTCGTTAATATTATGTGGCAATAAGGTAACTCAACTGATGATATCATGAATTTAATGCTAAAAGGTGTATAAAAAATATTAAGCTCAA

TTGATATTACGAAATTTAAGAATAAATATAAAACCAAGCAGAGTCATAAATGTCATTTAGTAACATATTTTTCCGTCTAAACAAGCAAATATGATTCGTG

GCAGGGTCTGAAGGATAGTGGAATTGGTTCTCAAGGGATCACCAACAGTATTAACATGATGACAAAGGTTAAGACTACAATAATCAACTTGCCAGCACCT

TCTTACACTATGGGATGAGAATAATGAGATGTCCAAGAAACAGTTTAGTCACTCAAAGTAGTAGTAATCAGCATATAAAGTAGGCTTTTCCGATCTCCCA

CGTTACAGCTTAGCCCTATAGAGTTGAGTAAGGGACAGTCTTTGAGAGGGGGCTGCTTGCATACAAAAAATATTTGTTGCAGTGCATGTGGATTAACTGT

ACTTTATGTTTCTATGGAGAAAAGGGTAATCACTATTTGTACAAGTAGGATATGAATCTTGAATAAATTATGGTTCTCCAACTTTACTTGAATTCATGGG

TGTATGCAT

>Glyma.05G029100 | Chr05:2501803..2508504 forward

ATGTGTTTATTGCGTGTTTTGAGTGCTGAATTCATTTTTACGATATGTAGGTTTGCTCATTCATTACCATTTGCAACTGTACAAGCAGAAGAGATATCGG

ACTCTAGGCCTGCTGAAGTTTTGAACCTGGGTATGGCCCAAAACCTTTGATGTTTGTGTTCTTTGCAGAGGGGCATCAGTACTCTTATGAATTTCATAAT

ACTTTGGATAAATTTGGTGATTGAAGATTGAGAAGGGTTTCCATCATAAAATGTACTTAAATATTTTTCCATCTCTATTTTACATGATTCTGTCTGCTAG

GGGTGAAACCTGCTTTTCAAGTATTTATCAAATACAAAAGCAGTTCGTTAAATATTATGTTCATCAATGAAATTGAAATTTGATGAAAAAGAAAGGATTC

CTGAACATATTTCCCACAATTTTGTTTCCTTCTATACATCAGAATCTGAATGGTACTGATTGATATTGTAAATTTCTGAACCACATTATTGATAAATTCA

TTTTTAATTTAACTTATTGTGCTCTGCTTTGATGGCTATCACTCTGAATGTATTGTCTTCACTTTCAGTGCAAGGTAAATGGGCAGGATCTTCAAATTGG

AATACAGTTGTGGATCCTTTAAATGGAGATTCATTTATTAAAGTTGCTGAAGTTGATGAAACAGGCATTCAGGTGCATCCCTGTAATGAATTATTTGTCT

TGTTCGCTTAATATTTTATTTTGAAATATGTTTCTTTTGTTGCACGCATAAAGAACTGCTGCTTAGGGACAATTCAGCATGATGTGCTCAAGGCTGTATT

CTTTTATTAGTTCCTCACCCTTCATACTATGAAATATAAAGTAACTGAGTTATTATGTTATTACTTATTAGTAACCACCCACCGCACCTATTTTTTGAAT

AGGAGATTCAAGTTTGAAGCTTCAACCTCTGCCTGTGAGACAGAGTTCTCATCATTTTGTAAAAAATGAAAAGAAATAAATTTCAATTCGAAGGCTATAT

ATGAGTTGGTACTTGATTGGAATTCAACAGATGGTTTGAGACAGAGATCTTTACTAATTTTTTCCTTTTGTTTGACTTATCAGCCCTTTGTGGAAAGCTT

GTCCAGCTGTCCCAAACATGGTGTACACAATCCTTTTAAGGCACCAGAGAGGTATGACCAGATTATCCTTTGTATCTATTCATTTTCTTTATAGAAATAG

AAGCATATAATAATCTTTTTTCTTTGTTGGATTAATGTTTGTCATAAACGTCAAAGAACGAAAAAAAAAATGATAGGAACCTCATCCTCAACATAAACAT

TTATGCCTTATGTTGTCCATTATAGGAGACTGTTGCCCAAATGGGATTTTGCTGCGGATGTAAGCATAGGTGGATTATCTAGATTAACCCTAGAGGTTTC

AGCCTTATTTTTGTAAATAATACAGCAGTTTTCTGGATAGCAAATGACACGACAGTTTAGTTTTTCATTTTTCTTTTGAAACAGAACATTAGGTGATTTT

CAGATGATTAGTTTGCTAGGTGTGAATGGAACGGAAGTGCTTATCCTTTTCGGGTGTGTTTCAATAGTTCCTGACAAAGCAAAGTTTGGATTGTAGCATA

AACTTCCAACCTCGATGGTTTCAAAGTTCTGATAGGAGGAGTAACTAAAATATATGGGGCTCTGGTATTCTAAAGCTGGCTATGATAAGAGTTAGAGTAA

ATAAACTTCCTAATGTCACCCAGCGGTTATTTTCTCTTGAAGAAAGCAGCTGACTTAGGCATTTTAGTTACAAGTATTTTATGCTTTAGAGCATGATATC

AATTCTTATGTTCTTATTTTGGCTATCTATATGAAATTTAATCTGTTTTTTGCTATAAATCTTTTGCAGATATCTCATGTTTGGAGAGATATCTGCTAAG

GCAGCTCACATGTTATCACTTCCTAAGGTATTTCAAACTAAGGAAAGATTTTTTAATTTTATTATTAGTTTTTTACTATTACTTTATTATGTATTGTTAC

CTGTTGTATTCAAGATCAACTTTTTCTTCTTTAATTGGAAACATTAAATAATTTATACTGCATGACCCTCAGGTTTCAGATTTCTTTACGAGGTTAATAC

AAAGAGTTTCTCCAAAGAGTTACCAGCAGGCTTTTGGGGAAGTCTATGTGACACAAAAATTTCTAGAGAATTTTTGTGGAGATCAGGTATATCAAAATAA

GACATGTTAGTGCTAAATAAATGAAATTCCAAGTAAAAATATGCCATTCGCTGTAAGGAATGATAGAAGAATCAAAATTTCACATTGACTTGTAGATATT

GATCAATTTCGTTTCTATCATTGATACCTAGATTCTTTGTTAGTGGCAGAATTTAACACCGATACATGTACACATGGAAACACCCACACAAAACTAACAT

TATTTATTTATAATATATGGTTTCCCTATGATGTTACAAAGCTCATATAATTCCTTTAGTGATTTCTTCTTTGTTGTATTTAACAAAATATTGACAGGTT

CGTTTCCTGGCAAGGTCTTTTGGTGTACCTGGAAATCATCTTGGACAACAAAGCCATGGTTTTCGTTGGCCATATGGTCCTGTAAGGATTTTGTTTTGTT

TAGCTTTGTTATGTTTTTAATTGGTATACTATGTTCTAAAGGTAAAAAAATGAGTCTATGGCATTCTCTCACCAACAGTGTTTGTTTATTTGTGCCTAAA

TTATCAATTACCTTGTGGTCAAGCCAGTAAGTTTATGGAAGTAATGGGTTAAGTGTACTCTAAGAGGTGGAAGAAATGAAAAATAAAAGCAAGTTTTCCA

AAATAAAACTTAGTAGTATGTAATAATTGTGACTATGAGATTTATTATTAAGAAGGTGGATTGTGGTAATTATGCTAATTCAGATGCATCTATAGTTCTA

TACATCAAGCCAATGTACATTGATGACCTTGATTGGAGCGGCTCATTGCCCTTTGGTTCTATAGTAAAATAAAGATCACCTTTTATCCCAAAACAGAAAA

GAAAAGAAAAGAAAAAAGGAATATTGCCTGAGGTTTCAATTAGTTACATATGATAATAAGGCAAAACCCTGGACTTAGGTATTCTTTCTTGCAAGAAAAC

TCAAGTCATTTGTAAGGTAAATGGTATCTTTGATAAAAAAAAAGTTTTTTGTTATTTACAGAAAGGTTTATGTCCTACAGATGTCACTTCTACTCATGAA

AAGAGTTCACAAAGAATTAGATGTTTTCTGATTTTGAATTGAATTTTGTTTTTTGATTAGTGATGCCATGTGGAGAACTGGAGATGTTTTGCAGTTTTTT

CATGAGTGTCTGGTTTAGTAAGTGAATGCATAGATTTTTTCACACACCATAAACATTCACCTTCCCCTCCTGTCGTAATTTTAAAGCTGAAAGTATGTTC

TGTAGGTGGCAATTATTACTCCTTTTAATTTTCCCTTGGAGATTCCTGTCCTTCAATTGATGGGTGCACTCTATATGGGCAACAAGCCAGTCCTTAAAGT

TGATAGCAAGGTGAGTTCTTTTGGCTCTGATTTTGGCATACTTTATGACATTACATCGAATACAAAATTGATACAGGGCTAGCTTCTAGGCTGCATGAAA

TATTGATCACAATCAGTTGTGGTGAAAAATATTGTTATCTGATGTTTTTCCCCGTGTGTTTATGAGTGTGCTTTTGGGGGGAGGGGGGGGGCAGGGGAGG

GGTATTTTAGCAATATTTTTTTACTTGGTTGATACCTTCATCTAGTTAAAGAAACCATTGTATGATTCATATCTATTGCTCTTCTTATTTTACTCTTCAT

CTATTGGATTTTGGACATGTGAATTTTATTTGTTTTTAAATTCAAATCTTTCTACAGTTACAATGCTGAACCATCGACTCAAATTGTTGTAGGTGAGCAT

TGTTATGGACCAAATGTTGCGCCTGCTTCATAACTGTGGTTTACCTCTAGAAGATGTAGACTTCATAAATTCTGACGGGAAGACAATGAACAAACTGTTA

CTGGAGGTTGGTTGTTTGGTACCTTCTTTTTTTCATCCAATGCATTGATTCTACAGAGCATAATATTATTCCTGATAGATTGCCAAATTACTTTTATGAT

TCTATTTCTTGCTGACCTCAATGTTAACTCATTGACCTCAATGTTAACTCATATTGACCTTTTCTTGTAATTTTGTTGGATCTTATTGGCTTTTGACTCC

GTTATTTTGTGGTAATCATATATCAATATTTGTATATGATTACATGTTCCTTTTATAATATGATATTGGATTATGAAGTAGTATTGAATTTGCAGGCAAA

TCCACGGATGACGCTTTTTACTGGTAGTTCAAGAGTGGCAGAGAAGTTGGCTGTTGATTTGAAGGGCCGCGTTAAATTGGAAGATGCTGGATTTGACTGG

AAAATACTGGGCCCTGATGTCCTTCAGGTTTCTTACAATCTTTTGTGGATACCATAAAGGCACTAATTCAATTTTCAGTTCTATAATAGTTTCTCTATGG

TATCTTGGTTTCAGGAAGATTACATAGCCTGGGTCTGTGATCAGGATGCATATGCATGCAGTGGTCAGAAATGCTCGGCACAGTCATTGTTATTTATGCA

TGAGGTCTGTTACATTGGTTATTATGAGAACTTTGTTCATGATTAAATGTCCCCAAGAACTACGGGGAAGATGTACATAAGTACATTGGGTTTTTATTTT

TAATATGCATGTTACGAGTTTATGTATCGTGTCTTTACTAACTTCATGGTTTGGATTTCCCTTGTTCAGAACTGGTCTAAAACTTCCTTGCTATCTAAGT

TGAAAGATCTTGCTGACAGAAGAAAATTGGCAGACTTGACAGTCGGCCCAGTTCTCACAGTAAGTATTACATGTCTGTTGTTTCCCCCTTTTAGTAATTG

GTCGGATTTAACATTTAAAATTGCTTTGGTGGCCTGCTTGAAGTTTGTGGCAAATCTTCTCTTGAAGATAACAAAAGTTCAACCAGCTTTGTAATATTTT

ATAAATTTAATTATTGAACGGATTCATTCTTGCAAGTTGCAAGTTATATGCAAAATGTAATAGTTTCAAATTTCAATTATCACCCCATATTCCAAAATCC

CCTTTTCATTACATTTACAATATGGACAAGTTATCCATGCTTTTGTGTGATGGTTAAAGAAGGGAGGGTAAGGCTTTAAATATCAAACGATGCAGCTATG

CAATTGATTTGTCCTCAGCCATTTAGAGCAATCTTCAGTACATTATTGACAGACTAAGAAGTTTGGACACTGATTTGTGTTTATTCATTTTATAATATTT

AATTAATGATACTGTTGGGATGATGATTAAATTTAATGTTTATGCCTTAGCCGATACTTTCGATTTGATTTGGTAGACATTGAACCTTATATATCTAAAA

ATGAATAATTTGGTAAACTGTTTCTTGATGCATCCAGACAGGATGTTATGTTTAATTATTTAATTAGTAATTAATTTGCTGGTGCATCTTTGCTGGAATA

ATCATTTGCTTTATTCTATGCAATGCCAAGCTTAGTTTATTTGAAATTCATGATTTTTCATCTTATATGTTGCAGGTTACGACTGATTCAATGCTTGAAC

ACATCAATAAGTTGCTTGAGATACCAGGATCAAAGTTGCTATTTGGGGGTCAACCTTTAGAGGATCATTCAATTCCACCTATTTATGGTGCTATGAAACC

AACAGCTGTCTATGTTCCTCTTGAGGAAATTATGAAGGCTAAGAATTTTGAGCTTGTGACAAGAGAAATATTTGGACCATTTCAGGTATGAAAAGCGAGA

CTTGGAACATGAAAATAAAGTTTTCAGATGGATTTTCTAAAAGAATAATATCTGGTTGCTCTGTTGTGAGATAAATAATTTCTATATATATATATTTGCA

GATTGTTACGGATTACAAAAGCAGTCAACTATCAGTTGTATTGGATGCTTTGGAAAGAATGCATAACCATTTAACAGCTGCTGTAGTTTCCAATGATCCT

TTGTTTTTACAGGCAAGACTTTTATAACTTAAATTTATGACATGAACGATTTATCAATTTTTAAGCAAGTCTCAATTTAATATGACTCCTATCAACTTTT

CTCATTGTCCATTTTGGTCATAAAGATCATTAATTCCTTATGTTTCATATTTCATGCATGAAACAAAATTATGGCTATTTAGGGGATTGGACTCTATTTA

TACTGCACGTCTAAAAGTTTATTCTACATAATATGATATGATATCAATCGCCTGCAAGATTTTTTGTGGTAATTAGAGTTCAACTCCAAAATTGAGATGG

CTGACACTTATATTCAACATGCTACTTGTCTGCTGAGCACATAATAAGTGTGCTATCTTCTGATTTGATCATTGTGATTAAAACCTTATTTGCAGGAAGT

CATTGGCCAATCAGTAAATGGTACTGCTTATGCTGGTTTAAGAGCAAGGACAACCGGAGCTCCACAGAATCACTGGTAAATTGATATTTTTTTAAACTTG

TTTTACCTGTACATAACTAAGAAATGATTCTTTAATTTGTTGAAAATAATAATTTCATTGCAGGTTTGGACCCGCTGGTGATGCTAGAGGTGCAGGAATT

GGAACACCAGAGGCTATAAAACTTGTATGGTCTTGCCACAGAGAAATAATCTATGATTTTGGGCCTGTGCCAAAGAATTGGGAAGTTCCTCCTTCTACTT

GA

>Glyma.05G029200 | Chr05:2510682..2517754 forward

ATGTTCATGTTTTTGGTTAGCAGAGTAACTAAGGATTCAATTTCACGCAACCGCAATGCCTTTGCTTCTTTTGCTTTCTCTAGGTATCTCTTGTTTTTGT

TTGTCTCCTAAAATTGATTCACTGAGGATTTTAATTTATTCAATTTTCGTAATGCTGATCCTCCTTTAATTAGTGGAATTTAATAATTTTAATAGTTGAT

CACTTCACTGATCTATTTGTATCATAATTTGGCAGCAGTTTACTGGTTTTGGGAGGTCCTATTGAATTGCTACTTTGGTCTAATTGCATTTATCCAAACT

GATTGGGTTGGATCATTTTTCAGAACTCAAGTCAAGAGCAGCTGGGGCATTGTGTTGATTTTTATTTTTTTAGTTAAATAACTCACAGGAATGCTAGAAA

AACCATTTTGTAGTTTCATTCAATTCCCTTTTAAAAAAAACAAGATAAAAGTATTATATAAGCTTTAACTGGTGATTCTTTTTTATTCGGTTATATTTAT

TGTATATAATTCTAAAATATTAACGCTAAATTCATGTTTACAATATATAGCAGGTGTGCTCATTCATTATCATTTGCCACAGTAGAAGCAGAAGAGATAT

CAGGTTCTAGGCCTGCTGAAGTTTTGAACCTGGGTATGGCAAGCAACCTCTGATGTTATTGTTCTTTGCAGATATTATTAGTACTCTTAAGAATTTCATA

ATCGGATAAATTTGGTGATTTAAGATTGAAAAGGGTTACCATCATAAGATGTACCTGAATCTTTTTTCATCTCTATTTTCCATGATTCTGTCTATAAGTT

GTCCAACCTGCTTTTCAAGTATTTATCAAATCCAAAAGCATATTGTTAAACATTATGTTCATCAAAGAAATTGATATGTGATGGAAAAGGAGGGATTCCT

GAACATATTTCCCTTAATTTTGTTTACTCCTATGCATCAAAATCTGAATTGTACTTAGTTTAGTGTTATTGTAAATTTGTGCTACACCTATTTATCAACA

ATTCATCTTTAAACAAACTTATCGTGCTGTACTTCGATGACTATCCCTCTGTATTGTCTTCACTTTCAGTGCAAGGTAAATGGGTAGGATCTTCAAATTG

GAACACAATTGCAGATCCTTTAAATGGTGACTCATTTATTAAAGTTGCTGAAGTTGATGAAACAGGCATTCAGGTGCATCCCTGTAATGAATTATTTGTC

CTGTTAACTTAATATTTTATTTTGAAATATGTTCCTTTTGTGGCACTCACAAAAAACTGCTTCTTAGTGAGAATTCAGCTTGATATTATCAAGGTTGTAT

TCTTTTATTACTTCCTCACCCTTCAAACTTCAAACGATGAGATATGAATTGCCTGAGGCATTATCTTACTAGACACCACTTACCACGCCTGTTGTTTGAA

TAGGAGGTTCATGTTTGAAGCTTCAACCTGTGTGTGTGACACAGGGTTCATCATTTTGTGAAAACAAAAAAGAAATTAAATTTCAATTTGAAGACTTTAG

ATGAGTTGGTACTTGGTTGGAATTCAACATTTAGTTTGAGACAGAGATCTTGACTAAATTTTTTCTTGTGTTTGACTTATCAGCCTTTTATAAAAAGCTT

GTCCAGCTGTCCCAAACATGGTGTACACAATCCTTTTAAGGCACCAGAGAGGTAAGTGCAGATTGTTCTTTCTCTCTCTTCTTCATTTTCTTATAGAAAT

AGAAACATATAAAAATATCTTTCTTTTGGTTGAATTTCCATAGAGGTCAAAAAAGAAAACAATTATTGGAACCTCATGGTTGAATATAAACCTCTATGCC

CTGTATTCTCCATTATTGGAGACTGTTGCCCAAATGGGTTTTTGCTGAGGATGTACGCATAGGTGGATTATCTAGAATATTCCCAGAGGTTTCGGCCTTT

TTTTAAAACCTTTTGTAAATGATACATTATTTTTTTGGATAGCATATGACACGACAATTTAGTTATTTTTCTTTTGAAATGGAAACGTGAGATATGGTTG

GTTTGCAAAGTGTGAATGGAAGTACTAATCCTTTTTAGGCGTGCTTCCATTTTTCCTGACTAAACAAAGGTTGGATTATAGAATGAACTTCATCCCGGTT

GGTTTTAAATTTCTGCTATGAGGAGTAACTAAAATGCATGAGGCCCTGGTATTCTAAAGCTGCATATGATAAGAATGAGAATAAATAAACCGTCTACTGT

CACACACCTTTCATTTTCACTTTATGAAAGCAACCGACTTGAGCATTTGTTGCAAGTATTTTATGTTTCAGAGCATGATTTCAATGCTTATGCTCTTATT

GTGGCCATCTTTTATGAAATATAATGTGGTTTTCATTATAAATCTTTTGCAGATATCTTATGTATGGAGATATATCTACTAAGGCAGCTCATATGCTATC

ACTTCCTAAGGTATTTCAAACTAAAGATTTTTTTAAATTTTACTTTATTATGTATAGCTGTTACCTGTTGTATTCAAGATAAACTTTTTCTTTTTTAATT

GGAAAAATTAAATAATCTATACTGTGACCCTCAGGTTTCGGATTTCTTTACAAAGTTAATACAAAGAGTTTCTCCAAAGAGTTACCAGCAGGCTTTTGGG

GAAGTTTATGTGACACAAAAGTTTCTAGAGAATTTTTGCGGGGATCAGGTATATCAAAATAAGATATGTTAGTGCTAAATAAATGTAATCCCAAGTACAA

ATATGCCATTCTCTGTAATGGATTGATGGAAGAATCTAAATTGCTCATTGAATGGTATGTATTGATAAATTTCCTTTTGAATTCTATCATTGATACCTGG

ATTCTTCATTGGTGGGGGAATTCAGTCCTGGTACATGCGCGCACACCAACACCCACAAAACTAACATTGTCTTATGTGATTTCCCTAGGATGTTACAAAG

CTCATATAATTCCTTTAGTGATTTCTTCTCTGTTCTTGCTTCAATTTAACAAAATCTTTACAGGTTCGTTTCCTGGCAAGGTCTTTTGGTGTCCCTGGAA

ATCATCTTGGACAACAAAGTCATGGTTTTCGTTGGCCATATGGTCCCGTAAGGATTTTGTTTCATTTATTTTTGCTGTCTTTTAATTGGTATACTCTATT

CTATAGGCAAAAAATGAGTCTGTCATTCTCTTACCAATGGTGCTTGTTTATTGTGCCCAAATTATCAATTACCTTGTGGTCAAGCACGTTAGTCTATGGA

AGTAATTAGTTAAGTGTACTCTAAGAGATGGAAGAAATGGAAAAGGAAAGCAAGTTTTTCCAAAATAAAACCCAGTAATATGTAATAATTCTGTGGCTTT

GGTTTAGTAATAAGAAGGTGGAATGTGGTAGAACTGTTTAATATGCCAATCTAGTTACTTCTATATGTCAAGCCAATGTAAATTGATGACCTTGGCAGGA

GTGGCTAATTTCCCTTTGGTTATTAAGTAAAATAAAAATTGTCTTCTATCCCAAAAAAGAAAAGAAAAGATAAAGGAATACTTCCTGAGGCTTCAACTTT

TTACATACGAAGGGAAGTCAAAACCCTGGGCTCCAGTACTATAAGAATGCCTAAGTCTTTTATAAGTGCTATCTTTGATCAAAAATAGTTTTTTGTCTTC

AAAATAAAATTGTTCTATAGTGTAACTTCTACTCAAGAAAACAGATAAAAAATGGAATTGGATTTTTCTGATTTTGGATTTAGTTTTATTTAGATTAGTG

ATGATGTGCAGAGATGTGTTGCACTTTTTTCGTGATTGCCTGTTTAGTAAGCAAATCTTTAGATTCTTTTACACGCCAAAAATATTGACCTTCTCGTGAT

GTAATCTTAAAGCTGAAAGTGTATTCTGCAGGTGGCCATTATTACTCCTTTTAATTTTCCCTTGGAGATTCCTGTCCTTCAATTGATGGGTGCCCTTTAC

ATGGGCAACAAGCCAGTCCTTAAAGTTGACAGCAAGGCAAGTTCTTTTGGCTCTGTTTTTGGCATACTTTTATAACATTATACAGGGCAAGATTCTAGGC

TGCATGAAATATTGATCACAATCAATTGTGGATGAAAATAATCGCAATGCTTTTTCTCCCTGTAAGCGTTTGTGTGGGGATTATGTATTTTTCCTGCCAT

GCAATATTCTTTGATTCGGTTGGTATTCTCATTCAGTTAAAGAAATATATCTATTGCTCTTTCACATCCTTATTTTGATTTTCATCTATCAGATTTTGGA

AACATTAATTTTATTTGCCTTTAAAATTCAAATCTTTCAGCAATTATAATTCTGAATTATTGACTCAAATTAATGTAGGTGAGCATTGTTATGGAACAAA

TGTTGCGCCTGCTTCATACCTGTGGCTTACCTGCAGAAGATGTAGACTTCATAAATTCTGATGGGAAGACAATGAACAGGCTGTTGCTGGAGGTTGGTTG

TTTTGTTTGGTACCTTTGTTTTCACATCTAAGGCATTCATTTTGCAGAGAATAATGTTATTCCTGAAAGATTGCCAATTTACTTATATGCTTCTATTTCA

TGATGATCTCAATGTTAACTCATATTGACCTCTTTTTTTGAGTCTTGTTGAATTCTATTGCCTTTTAAGCTCCTTTATTTTGTGCTAATCATATATCAAT

ATTGATATGTGGTTACATGTTCCTTTTATAGTATGTCAATGACATTAGATCTTGAAATTATATTTACAGGCAAATCCACGAATGACCCTCTTTACTGGTA

GTTCAAGAGTGGCAGATAAATTGGCTGTTGATTTGAAAGGTCGCGTTAAATTAGAAGATGCTGGATTTGACTGGAAAATACTGGGCCCTGATGTCCATCA

GGTTTTTTACAACATTTTTTTGTGGATATCATAAAGGCACTGACTCAATTTTCAGTTCTATAACAGTTTTTCTATGGTTTCTTGGTTTCGGGATCAGGAA

GATTACATAGCGTGGGTCTGTGATCAGGATGCATATGCATGCAGTGGTCAGAAATGCTCAGCACAATCATTGTTATTTATGCATGAGGTCTGTCACATTG

GTTATTTTCAGAACTTGTTCATGTTTAAATGTCCTCAAGAACTAGGGTGAAGATGAAGATGTGCAATTGTTATTTATTTTGTAAGATGCACTTTACGCAT

TGCATCTTTACTAACTTCAGGATTTGAATTTCCCTTGTTTAGAACTGGTCTAAAACTTCCTTGCTATCTAAGTTGAAAGATCTTGCTGAGAGAAGAAAGC

TAGAAGACTTGACAATCGGCCCAGTCCTCACAGTAAGTGTTTTTTTTTTCTTAAAAGGAAGAAATATAAGAAAAAGAAAGTAAATGGAGACTCACAGTAT

TATATGCCAGTCATATCCCCCTATTAGTAAATTGAACTAGGATTTAACTTTTAGAACTGTTTTGGCGGTGCACCCAAAGTTTGTGGCATTCTTCTCTTTA

AGGTGATGAAAGTTCAAAGTGCTTTGTAATATTTTTTAACTTTAATTATATGACTGGATTCCAGTGTTCATTGTGATAACTTATATGCAAAATGCAATAG

TTGCAATTATCATCCCTTTTACATTACATTTACAATATGGAGAAGGTAGCTATACTTTTATGTAATGATTAAATGAGGGGGTTGGGGGAGGGGGGATGGG

AGGATATGCAACTGATTTGTCCTCAGTCAATTGATTTGTGTTATATTATTTGATTAATGATACTGCTGGGATGATGAGTCAAATTCAAAGTTTTTGCCTT

AGCTGATGGAGATTGTACATCATTGGTGGGCAGTGAACATATATATCCAAAAAATCAATAGCTTTTTGATAAACCATTTCTTCGTTCATCCAAACAGGAT

GTCATAATATTTATCTAGTTCTTCATTTGCTGATATGCATTTGGCAGAATAATTGTCTTCTTTCTTTCATGCAATGACAAGTTTCATTTGAAATTCTTAA

TTTTTCTTCTCATATGTTGCAGTGTACGACTGGTATGATGCTAGAACACAAGAATAAATTGCTTGAGATACCAGGATCAAAGCTGCTCTTTGGGGGTAGT

CCTCTAGAGAACCATTCAATTCCACCTATTTATGGTGCCATTAAACCAACAGCTGTCTATGTTCCTCTCGAGGAAATTATGAAGGATAAGAATTTTGATC

TTGTAACAAAAGAAATATTTGGACCCTTTCAGGTAAAGAAAGAGACACTGAGAACATGAAAATAAAGTTTTCATATGTCTGCTTTAAAAAATAATAATCT

ACTCTGGTCTTTTTGTGAGATAAATATTTTCTATTTATGTTTTCAGGTTATCACGGACTACAAAAACAGTCAACTATCAGTTGTATTGGATGCTGTGGAA

AGAATGCATAACCATTTAACGGCTGCTGTAGTTTCAAATGATCCTTTGTTTTTACAGGCAAGACTTTTATAACTCAAATTGTCAATTTCTACTCAGTTCT

CGATTTATCATGACTCCTATACATTTTCTTGTTGCCCATTTTGGTTATAAAATCATTAATTACTTTATTTTGGTAATTGTGTTTCATTCATGTAATAAAA

TTAGTGTTGTTTAGGGGATTGGACTCTATATTTATGCTGCACATCGAAAAATTTATTCTCCATTTTCATAACTTTGTAGTAAGTAACTACAGTCTAACTC

TTCAAAAATTGAGCTGACTCACATTTCTTTAATATCCAACATGCTACCTGTATGCTTATCGTATAGATGAGTTTGATATCTTCTGAAATATTTATCATGA

ATGTGCTTTATTTGTGTCAGGTTCTTTATCTGATGGGATTATTTATGATTAAACACATATTTGCAGGAAGTTGTTGGCAATTCAGTAAATGGTACTACTT

ATGCTGGTCTAAGAGCAAGGACAACCGGAGCTCCTCAGAATCATTGGTAACTCAATATTTTTTTGAGTTTGTTTTACTTGTACATGAGTAAGAAATGATG

TTTCGATTTGTTGAAAACAATAATTCTATTACAGGTTTGGTCCCGCTGGCGACGCTAGAGGTGCAGGAATTGGAACACCGGAGGCTATAAAACTTGTATG

GTCTTGCCACAGAGAAGTTATATATGATTTTGGACCTGTGCCAAAGGATTGGAAAACTCCTCAGTCCACTTGA

>Glyma.17G097800 | Chr17:7691840..7700173 reverse

TAACACAAGCAAAAATTATCAACCACCTCCCAGCAGGCCAGCACCACGGTAACTACTAACCAATATTTTCACTCAAGAGAAACACGATAAATCCCACGTG

TACTCAGCAAGATCATCACTTCATTTTGGACTAAACTAGAAAGTGATCGAACAGCATGTTCAAGCTTTTGGTTAGTAGAGCAGCTAGGGTTTCAACTCCA

CACAACCACAATGCCTTTGCTTCTTTCGCTTTCTCTAGGTATCTTCTTTTATCTTCTCTCGTCTAGTTAGTTGAATTTAGTAAGTTTAATCGTTAATCAA

TTCGATACCCAGCTTGTTCTGTGATTTAGATGAAAAATAGCCTCCAGTTATCTGCGAAACAGTTGAAATTGTGTTCATTGGAACTTTAATTCGTTCCACG

ATCAACTTCGGGTTATCTAATAAAAAATATTATATGTATTTAGGTTTATGATTAGGTCCGTGTTCTGCTTACAAACACTGATGTTTCTGAGAGTACTGTT

TGTTACAGAACATTTTAAGATGTTAGTTCTATATATAAAAAAAATGATTTGAGTAACTAAGGATTTGTAAAAAACAATTTAGCTAATGATTACCTGAGCT

GAGTACTGTTTAGAATATTAGCAAGGCTATAACTCTAAACCAGAATATATATTGACCGATTTATTTGCATCATAATAATGCTTTATTGGCTGCAATTTGC

TGTCGTATTGGAGGCCCAATTGATTTGCTATTTTTTGTCTTACTACATTTACGCAAACTGATTTGGTTAGATAATTTTTTCAGAATTGAAGTCAAGAGCA

GCTGGAGCATTATTGTATTATTTTTTTATTTCTTAGGTTAAATGACTCACAGGGATGCTAGATAAGTCATTTTGTAATTTCCTCCAATTCACGTTTTATA

CAAAAAAAATTCAAAGATAAAGTATTAGATTTAACTACTAACCAGTATTTTTTTTTATTTGGTCATGTTTGTTGTACATATTTCTAAAATAGTTTACTAT

TACTCGATTTCATTTCTATAGCTTGTGATACAAGTCTTATTGTGTATTTTGAGTGCTGAATTCATTTTTGTAGGTATGCTCATTCATTACCATTTGCAAC

TGTAGAAGCAGAAGAGATATCAGGCTCTAGGGCTGCTGAAGTTTTGAACCTGGGTATGCCCCAAAACCTTTGATGTTTTTGTTCTTTGCAGAAGGGGCAT

TAGTACTCTTATGAATTTCATAGTACTTTGGATAAATTTGGTGATTGAAGATTGAAAAGGGTTTCCATCATAAAATGTACTTAAATCTTTTTCTGTCTCT

ACTTTCCATGATTCTGTCTGCAAGGTGTCAAACCTACTTTTCAAGTATTTATCAAATCCAGAAGCAGTTTGTTAAATATTATGTTCATAAAGAAATTGAA

ATGTGATGAAAAAGAAGAAAGGATTCCTGAACATATTTCCCACTATTTTGTTTCCTTCTATACATCAGAATCTGAATGGTACTGAGTGTTATTGTAAATT

TCTGCACCACATATTTATCAATAAATTAATTTTTAATCAAACTTATTGTGCTCTGATTTGATGGCTATCACTCTGAATGTATGTTTTCACTTTCAGTGCA

AGGTAAATGGGTAGGATCTTCAAATTGGAATACAGTTGTAGATCCTTTAAATGGAGACTCATTTATTAAAGTTGCTGAAGTTGATGAAACAGGCATTCAG

GTGCATCCTTGTATTGAATTATTTGTCCGGTTAACTTAATATTTTATTTTGAAATATGTTTCTTTTGTTGCGCACATAAAGAACTGCTGCTTAGGGAGAA

TTCAGCTTGATAGGGTCAAGGCTGTATTCTTTTGTTAGTTCCTCACCCTTCAAACTATGAAATATAGAGTGACTGAGTTATTATGTTATCAGTCACCACC

CACCACGCCTATTTTTTGAATAGGAGGTTTAAGTTTGAAGCTTTAACCTCCGTCCATGAGACAGAGTTCTCGTCTTTTTGTGAAAAATGAAAAGAAATAA

ATTTCAATTTGAAGGCTATATATGAGTTGGTACTTGATTGGAATTCAATAGTTGGTTTGAGACAGAGATCTTTACTAACTTTTTCCTATTGTTTGACTTA

TCAGCCCTTTGTAGAAAGCTTGTCCAGCTGTCCCAAACATGGTGCACACAATCCTTTTAAGGCACCAGAGAGGTATGACCAGATTATCCTTTGTATCTAT

TTATTTTCTTTATAGAAATATAAACATATAAAAATCTTTTTTCTTTGTTGGATTGATGTTTGTCATATAGGTCAAAGAAAGAAAAAAAATGATGGGAACC

TCATCCTTCAATATAAACCTCCATGCCTTATATTGTCCATATAGGAGACTGTTGCCCAAATGGGATGTTACTGTGGATGTAAGCATAGGTGGATTATCTA

GATTAACCCGAGGTTTCTGCCTTTTTTTGTAAATAATACAGCAGTTTGCTGTATAGCAAATGACATGACAATTTAGTTTTTAATTTTCTTTTGAAGCAGA

ACATGATGTGATTTTCAAATGTTTAGTTTGCTAGGTGTGAATGGAATGGTAGTGCTTATTCTTTTCAGGTGCGTTTCAATAGTTCCTGACAAAATAAAGG

TTGGATTGTAGAATAAATTTCCAACCCCATTGCTTTCAAACTTCTGCTAAGAGGAGTAACTAAAATACCATGATCCTGGTATTCTAAAGCTGCCTATGAT

AAGAAGTTGAATAAATAAACTTTCTAATGTCACCCAGCTGCCATTTTCTCTCCAAAAAAGCAACTGACTTGGCTATTTTAGTTCCAAGTATTTTATGCTT

TAGAGCATGATTTCCTATGTTCTTATTTTGTCTACTTTATGAAATTTAATCTGGTTTTTTGCTATAAATCTTTTGCAGATATCTCATGTTTGGAGAGATA

TCTGCTAAGGCAGCTCACATGTTATCACTTCCTAAGGTATTTCAAACTAAAGAAAGTTTTTTTAATTTTATTATTATTTTTTTACTGTTACTTTATCATG

TATTATTACCTGTTGTATTCAAGATAAACTATTTCTTCCTTAATTGGAAACTTTAAATAATTTATACTGCATGACCCTCAGGTTTTGGATTTCTTTACAA

GGTTAATACAAAGAGTTTCTCCAAAGAGTTACCAGCAGGCTTTTGGTGAAGTCTATGTGACACAAAAGTTTCTAGAGAATTTTTGTGGAGACCAGGTATA

TCAAAATAAGACGCGTTAGTGCTAAATAAATGAAATTCCAAGTAAAAATATGCCATTCTCTGTAAGAGAATAATAGAAAAATCATAATTGCCCATTGACT

TGTAGATATTGATCAATTTTGTTTCTATCATTGATACCTAGATTCTTTGTTAGTGGCAGAATTTAACACTGATACATGCACACATGCAAACACACACAAA

ACTAACATTATTCTATATGGTTTCCCTAGGATGTTACAAAGCTCATATAATTCCTTTAGTGATTTCTTTGTTGTTTTATTTTAAACAAAATATTGGCAGG

TTCGTTTCCTGGCAAGGTCTTTTGCTGTACCTGGAAATCATCTTGGACAACAAAGTCATGGTTTTCGTTGGCCATATGGTCCGGTAAGGATTTTGTTTTG

TTTAGCTTTGATATGTTTTTAATTGGTATACTATGTTCTAAAGGAAAAAAAATGAGTCCATGTCATTCTCTCACCAACAGTGCTTTGCTTATTGTGCAAA

AATTATCAATTACCTTTTGGTCAAGCAAATGAGTTTGTGGAAGTAATGGGTTAATTCTAAGAGATGAAGAAATGAAAAACTAACCAAGTTTTCCAAAATA

AAACTTAGTAATTCGTAATAATTGTGAGTTGTGACTATGATTTGTTATCAAGGAGGTGGATTGTGGTATAACTGTTATTATGCTTATTCAGTTGCCTCTA

TACGTCAAGCCAATGTACATCGATGACCTTGGCTGGAGTGGCTGGTTACCCTTTGGTTCTTTAGTAGAATAAAAAATCATCTTTTATCCCAAAACAGAAA

AGATAAGAAAAAGAATATTGCCTGAGGTTTCAGTTAGTTACATATAATAATAAGGCAAAACCCTGGACTCAAGGTATTCTTTCCTACAAGAAAACTTAAG

TCTTTTGAAAGGTAAATGCTATCTTTGATAAAAAATAGTTTTGTCTTTTACAAAATAGTTTATGTCCTATAGATGTTACTTTTACTCAAGAAAAGAGATA

AAAAAGAATTAGACATTTTCTGATTTTGAATTGAATTTTTTAGATTAGTAATGCTATGTGGAGACATTTTGAAGTTTTTCATGAGTGTCTGGTTTAGTAA

GTGATTGCTTAGATTTTTTTCTCACACCATAAACATTCACCTTCCCCTCCTATTGTAATCTTAAAGCTGAAAGTGTGTTCTGTAGGTGGCAATTATTACT

CCTTTTAATTTTCCCTTAGAGATTCCTGTCCTTCAATTGATGGGTGCACTTTATATGGGCAACAAGCCAGTCCTTAAAGTTGATAGCAAGGTGAGTTCTT

TTGGCTCTGATTTTGGCATACTTTTTGACATTACATCTAATACAAAATTGATACAGGGCTTGCTTCTAGGCTGCATGAAATACTGATCGCAATCAGTTGT

GTCGAAAGTAATTGTTATCTGATGCTTTTTTCTGGGTTGTTTATGAGTGTGTGCAGGGACGGATCCAGGACTATATCAAAGGGGGAGCCCAAATTTTTTT

TACAAAATTATTTAAATATTTAACTTCTAATAAATAATAAGCTTTAAAAAATATTTATTATTAATTTGTGTAAAATTAGAGATATAACCCACTACTAGAA

CAAAATTAAGCAAATACTAATTCAAAAACTTATTTTTCTTATATATTTTCCTTAATTAGTATTTTTTTAATTTTATTTTTTCTTTTTTTGTCTCATATAT

ACATAAAGGGTCCTAGGGGAGGGGGGGCCAAGGCCCCTACTTGTGGATCCGTCCCTGAGTGTGTACTTGTGTTTGGTTGGGGGGGAGTGGGGGGTGTCCC

CTTCCAAGCAATCTCTTCTTACTTGGTTGGTACTTTCATTTAGTTAAAGAAACCATTGTATCTATTGCTCTTTCTTATTTTGTTCTTCATCTATTGGATT

TTGGAAATGTGACTTTTATTTGCTATTAAATTCAAATCTTTCTGCAGTGACAATGCTGAATTATTGACTCAAATTGTTGTAGGTGAGCATTGTTATGGAA

CAAATGTTGCGCCTGCTTCATACCTGTGGCTTACCTCTAGAAGATGTAGACTTCATAAATTCTGATGGGAAGACAATGAACAAGCTGTTGCTGGAGGTTG

GTTGTTTGGTACCTTTGTTTTCACATCCAAGGGATTGATTCTGCAGAGCATAATATTATTCCTGAAAGATTGCCAATTTACTTTTATGATTCTATTTCAT

GATGATCTCAATGTTAACTCATTTTGACCTCTTTCTATAATTTTGTTGGATCTTATTGCCTTTTGACTCTGTTATTTTTTGATAATCAAATATCAATACT

TTTATATGGTTACATGTTCCTTTTACAATATGATATTGGATTATGAAGTAGTATTAATTTACAGGGAAATCCACGAATGACTCTTTTTACTGGTAGTTCA

AGAGTGGCAGAGAAGTTGGCTGTTGATTTGAAGGGCCGTGTTAAATTGGAAGATGCTGGATTTGACTGGAAAATACTGGGCCCTGATGTGCATCAGGTTT

CTTACAATTTTTTGTGGATACCATAAAGGCACTAGTTCAATTTTCAGTTCTACAATAGTTTCTCTATGGTTTCTTGGTTTCAGGAAGATTACGTAGCATG

GGTCTGTGATCAGGATGCATACGCATGCAGTGGTCAGAAATGCTCAGCACAATCATTGTTATTTATGCATGAGGTTTGTTACATTGGTTATTATGAGTAC

TTTGTTCATGACTAAATGTCCCCAAGAACTACATTGGTTTTTTTATTTTGTATTATGCACTTTATGAGTTTATGTATTGTGTCATTACTAACTTCATGGT

TTGAATTTTCCCTTGTTCAGAACTGGTCTAAAACTTCCTTGCTATCTAAGTTGAAAGATCTTGCTGAGAGAAGAAAGTTGGCAGACTTGACAATCGGCCC

AGTTCTCACAGTAAGTATTGCATGCCTGTTATTTTCCCCTTCTAGTAATTGTTCCAGGATTTAACATTTAAAACTGTTTTGGTGGCCTGCTCGAAGTTTG

TGGCAAATCTTCTCTTGAAGTTTATGAAAGTTCAACCATCTTTGTAATATTTTGTAAATTTACTTATTGAACGGATTCATTCTTGTAAGTTATACGCAAA

ATGTATTAGTTTCAAATTTCAATTATCACCCCTCGATCCCTTTTTCATTACATTTACAATATGGACAAGTTATCCATGCTTTTGTGTGATGGTTAAGGGA

GGGAGGGGTGTGTGGAATGTGGGAGGATAACTTATAACTAAATAAGGCTTTAGATATCAAAGGTTGCCGGGTATGCAATTGATTTGTCCTCAGCTATTTA

GAGCAATCTTCAGTACATTGATTGACAGGCTAATAGAAGTTTGAATACTGATTTGTGTTTATTCATTTATAATATTTGATTAACGTTATTGTTGGGATGA

TGAGTCAAATTCAAAGTTTATGCCTTAATTGATATCTTCTATTTGATTTGGTAGTCTTTGAACCTTATATATCCAAAAACGAATAATTTATTGGTAAACT

GAAGAATTATTAATTAGCTGGTGCATCTTTGACGGAATAATTGTTTGCTTTATTCTAAGCAATGACAAGCTTAGTTTATTTGGAATTCTTGATTTTTCAT

CTCATATGTTGCAGGTTACGACTGATTCAATGCTTGAGCATGTGAATAAGTTGCTTGAGATACCAGGATCAAAGTTGCTATTCGGGGGTAGTCCTCTAGA

GAACCATTCAATTCCACCTATTTATGGTGCTATTAAACCAACAGCTGTCTATGTTCCTCTCGAAGAAATTATGAAGGATAAGAATTTTGAGCTTGTAACA

AAAGAAATATTTGGACCATTTCAGGTATGGAAAGGGACACTGAGAACATGAAAATAAAGTTTTTATATGCCCTTTTAAAAAAAAAATTCTTTTTTTTAGT

AAAAAATAATAATAATTCTACTCTATTCTTTTTGTGAGATAAATATTTATGTTTCCAGGTTATCACGGACTATCAAAACAGTCAGCTAGCAGTTGTATTG

GATGCTTTGGAAAGAATGCATAACCATTTAACAGCTGCTGTAGTTTCAAATGATCCTTTGTTTTTACAGGCAAGACTTTTATAACTGAAATTTATTTGTA

TGACATGAACAATTATCAATTTTTAATAATTGTGATTTTGGTCAATTTAGTATGACTCAATTTTAAGTTTCAATTTAGAATGACTCCTATCAACTTTTCT

CATTGCCCATTTTGGTCATAAAAATCATTATTTCCTTGTGTTTTCATAATTTCATTCATGAAACAAAATTATGGTTTAGGGGATTGGACTCTATTTATGC

CGCACGCTGAATTTTTTTTTTCTACTTATATCAATTGCCTGCAAGATATTTTGTAGTAATTAGAGTTCAACTCCAAAGTTATGGCTGACACTTATATCCA

ACATGTTACTTGTCTGCTGAGCACATAATTGTGTTATCTTCTGATGTGGTCATTGTGATTAAACACGTATTTGCAGGAAGTCATTGGCAAATCAGTAAAT

GGTACTACTTATGCTGGTTTAAGAGCAAGGACGACCGGAGCTCCACAGAATCACTGGTAAATTGATATTTTTTTCAACTTGTTTTACTTGTACATAACTA

AGAAATGATGCTTTAGTTAGTTGAAAATAATAATTTCATTGTAGGTTTGGACCCGCTGGTGATGCTAGAGGTGCAGGAATTGGAACACCAGAGGCTATAA

AACTTGTATGGTCTTGCCACAGAGAAATAATCTATGATTTTGGGCCTGTGCCAAAGAATTGGGAAGTTCCTCCTTCTACTTGAGAAAAGTCCTATTTATG

ATAGGAACAACGAAATTAGGAGGGTGTGTCCTATTTATGATAGAAGCGATGAGTAAGAATGTAAGAGGAAGCATATTTGCATGTAGGATATGCCACAGCC

CCTCCATGACATTTTTAGATTTTAGAATAAATTTATGAAAAAAAAATATTTTATTAGGCTTTTTTTTTATTGCTGAAATATAATTGGTGAGATCATCATC

GGTTGAGGTGCTGACATGATAAAAGGAATCAAATTGTATTGATGCCGAACAGTGTAGAATAAATAATTCATATCTAATGCTGTAAAAAAAAAAATCTTAT

CTAAATAATTTAAAGAATTGGCTTACAGGCTCTC

>Glyma.01G099800 | Chr01:33261108..33274284 forward

TTCACACCAGTGGCTTCTTCATTCCTTTATTCATTCATCTCACTTCTGAACACAAGAGAAACAATGGAACTTTTGCAAAATGGCCACAAAAATTTCGTCA

GCATCAAACCCTCCGAGTTACCCCTCACGAATGGGGCAGCGTTAACTCTCCTCAACTCGCTCTCCAAAACTCAGTACTTGGGCAACATAGATCCTTCCAG

AGTTTTTGTCACCAAAGTTAAGCGTATCATTGTAAAGGTACTGCTTTTAATTTATCACTACTTTTATTTTATTTGTAGAAATCAAACTCAAGTAGGTTGA

GTTTATAACAATTTATCATGGTTTCTCAAAACGATTTTTTTTTTTGTTTAATTGAGGAAATTATACAAAATGAGAGTACTAAGAGTTTGCTTACTTGGAT

TTTCATTTTTAATCTGTTTTTTATTTATAAAAGAAATAGTTTTTTTTCTGTATAAATATTTGAAAATAAGAAATAAATTGAAAATAAGATGATAATTTTG

TCCATTAAACATAAAAACAGACATAAATAGGCCTTAATTTTCAAAAGCAAAAATTAGAATTTCTTTCTCTCTTTAATTTAATTTTCAATGTTTTCCAAAT

TTCTAGTTTTCTCTTCTCTGCATCAAAATGAAATAAGGGAGACGAGGCCTTTCTGTGTTTCCTTTTTCAAACCTTTTTTCATATCCATATCGCGATTCTC

CTCAACAATCAAAATGTCATCATTTTTTTTTCAACACCTCTACTGGCATAAAGTAAACGGGTGTAGTAATTTTGTTTTACTCGATTTTCGAGTGAATTTC

CCTATTTATTTCTGAATTTCTTTTTTACGAACATGACAGTTTTTAGAAAGGTGAAGATTTAATTTTAGTACCAGGCATTTATAAATGTTTATTAAGTTAA

TTATTTTTGTGTGAGTGGTGATATGATTGTTTAATGTTTTTATATGTTTTTTTTAAAAACAAGGTATTATTAAAAAATATAACGAGTGTTATTACTAAAT

TTTTCGAATATGAGGCTGTAATTTTAGTTTATATTTATTTACTATTGTAATAACTTGTTTTTTTTTCTTAATTTCGTTTTATTGCCCTAAAAGCAAAAGT

TTGGGTTTCACATGTCTCTGTATATTTTTTAATGTTTTATTTCAATGAGTTGAAATTGAGAGGAAAAAAATAAAGAAAAAAGGAAAGGGACAAGAAAGAA

ATATAAGTAAAATTTAGATGAAATTTTAAGTTGTTTGGATGGATAAAATTAATAAAGAATAGTTCCTTTTCATTTATTTGGTTATGTTCAAAAATAAAGG

AAAACAATAAAAAAATTTGCAAAATCATATAATTTTATCACCAATGAAATAAAGCATGACATGATTCAAAAACTCAAACTCAAAATATTCATATAAAGGT

AGAATAGAAATATTAATATATTGACACAAGTGATTTGAGACTTAAATTTCTTAATTAAGATATGAGATTTGATTTTTAGGTAGAACATGAAATTATAAAT

AAATGTATCATTTTATTTTAAGATAAATCTATCCAATGAAAACTAATTAATTGAGTGTCAAATAAAATATTTCATATTTCGTAGTTTAAAAAAACATATT

GCTTAATAAAAAAAAAAGAAACACTTAACATATTTATGCTATCCAGCCAAAAGAAACATTAACATATCTATGCTTTGCAATTGCATCTTCCACTTTATTC

CTTTTGGCAAATCTTCTCTATATTTCATATCATCTAAAATGTGGAATAAATGCATTTCTACTCTTCTTTCATCCTTCCAATTTCATTCTTTTCCATTTCT

TCTCAAAGAAACAAAGACTAATAGTGCTATAAAGATATTATAAAGATATTAACAAGCTTATATTATTTTTATATTGATTGTAATTGTAATTAGTACTCAA

CAACTTTTTACTTGGACTAAAAAATTATTCTCTCTATTTAACACCAGTCCATCACAAAGAGTAAACATACATTTTTATCTAATTAAAAAAATGATTAAAT

ATGTTTTTTTATATCTAAAAGATATATTATTTTTAAGTTAATACATACTTTTGGTCAATTATTAATTATTAAATACCTGAAAAAAATTTATTTTAACATA

GGACCTGTATTACTCTTACTCTGTTATTCCGATGTTCCGTAGCTATTTCTTAATCATAATCGTTCATCCTCTTTGCAACCCAGCTTCTACCCTTTCGTTC

CTCTTTGCAATCCAGCTTCTACCCTTTCTTTGATACAGCGACCTCGTTGAAAACCTATATTACATGAGGCTATGAAGCACGGATACCCAAGTCTCTTGTC

GTGTTCGGTGTTCGACACTAGTTCGTGTCACTGTCCAACACCGACCCAACACCCGTGCTACGTTTTATATTTTAGACATTACAGATGTTTACGTGTTCGT

GTCGGTGCTTCATAGACTTGAGGATGCCACTGCCGCCGGATCCCCCTCTGTGCTACCGAAGGCCACCAAGGAGGCCCTCCTCCGGATCCCCAGCGTGATC

CTCAACCTCATCCTTGAAAAAAAAAAACCCTTTTATCTCTCGGACGTTCTATTGTTCCCAGAATCCCTTTTTTTTTCTTTCCCATCAACACGATGTCTTC

TTCGTTGCTCTATACATGGGAATCAAGCTCTGCTTCGACAAGAGCTCACCGTGGCAAACGATTTTGGCGAGTCTTCATAGGCTGATGCTTGTGCAGATCT

TTGTTTTCTCCATCGATCAGCGATGGCAGCAACAAAAACAAGTTGTATTTTCATGTGATTGGTTTTCATTTTATGGTTTGTTTGGAAAAAAAAAAACTCA

TGGATGTTGATTTTGAAAAACATGTTCGTCTTTGTGGTTGGATGCCATTTTGCGATATCCTCTTGCGATCTGGTTTCGAGATGTTTGCATCTAACAGAGA

AAGGTCAAATAATGTATTAGGAATTTTTATTATTTGTCTTTTTAAAATAAAACATAAAATAAAATTATATAATTATCCTTTAATTTATGTTGCTATAAAT

AAATGATAAAATGTTTTTTTTCTTCTCAAAAGTGAAAAGGTATAAAGTATTAATATACACCTTCTAAATTTTTAAGTGAGTCCCAAACTTCTTAATTAAT

ATATATATACTAACTCATAGTCATGGTTAAGATTTAAATAATTCAAACGAAGTTATACGTTTTTAAGTAAATCAAATTTGAATTTGACAGAATTAATTTC

TAACCGTAGTAAAGGTAATTAAAATGTATATTTAAATACATATTTACTAAAATTTTACATTAATATATATTTTTATTAAAAAACATGAAAATTAATATTC

AAATTTATTTTAAAAAGTAAAATATAATCAAGCAAACACACCTTTAAATTTTTTAATTCTTATTTTATAGAATTACTTAAAAAAACAAATAAGAACCGAG

TTTAGCAATTTGAAAAACTAAAAAAAAGTTTTAATATGATAAATATCTAATAATTCAAAAATAAATAGAAGATGTATTTGTTGCGAATGAATGGTTAAGG

ATTAAATAAAAATAAAACCTTATCTTCTAATACGAATGCGAATATTCTAAAGTCTTCTAAATTTTTTTGATAGACATTAATTACTCTCCACATAAACACT

TTGATAAAATTATTTGTGGTTTAGGTTTGTGCTGGACTTTTGTCAATCTTTTCAGCGTCCTATTAGCACCCCTACCCTGAATGATTAGATTTAGAGGTTC

CCTCCCCCTTTCATTCTTTGGATAAACGGGAACAAAAAAAGTGCAAGGAAATATATTTAATGAAGAGAATTAATTAATTTAAAAGTGAGAAGGGTACTTG

ACTCTCGTTTAAAATTTGAAAAACAATCAAAAAAAGAAAATTTTAATATTAATAAGCATTATCTGCATAAAGAAAATGAATTTTGAAATTAGTTTGAATT

AAGAAAAACTTAGTTTAGGTAGGTCATGTTTAACAAAGATAGCTAATAAGATAATTGAAAGTTGAAAGATGTAATATGAAAATTGAATAAAAAAGTTTTA

GTTAAAAATGACACGGAGAAATTTTTTTAAAATAAATAATTTAATTTCAAATAAAGTGATAAGTGATAAATTTTATTAATTTATTGAAGATAAAAATAAA

AATAATGATCTAAATTTTATGTTTAAAAACAAATCTTATTAGTTATATATAGAAAGAAACTTTATCATATTTATTGTTTTTTAATTAATTCCTCCTTCAA

GAAATATATATATATATATATATATATATATTAGATTAGTAAGATAATATATTTTTGAACGCATTAATAAGATAATATAAGAAGTAGGGTAAAATTATTA

TATTATATAATATAAAATTAAACAATTGATTACAACGTTATAGAATAATTAATTATCTTATTTTATCTTATTCATGAATCGCTAGGTTTAAATCTAAAAG

ATACTTTAAAAAAATAGAAAATAAAAGAAAAAAATCCAGGATAATGGAAATTTAGTCGAATAGAGAAGTGGTGTGATTGAGTGATGAAGCAACAGCAGTA

ACCGTGATTCGTAGGGCCCACAAAGCCATGTCTCTGAACTATGCCACCTATTAAAAGTATTTAGTACTTATTTCTTTTACTTGTACGCATTCTCACGCGC

TCGAGCCTCCTATTTAATTCGACTCGGTCCTATTGCATAGACTCCATGGATCCCACTCGAGCTTTCGTCAATTCTGTCAAGCGTGTTGTTGTCAAGGTCC

CACCTTGTTTTCATTGTTTCGTAATTCATAAGTACTATTATAATTATCCCGATTGTTATTTACAACATGCATGCATTCTCTTGTCTATTGGATAGAGATG

GTTCTTTATTTGTTATGGAAAAGATGTCACGTATTTTCTAGATGAAAAATTGGGCATGGTTTTTGTTCCTTATAGTTTGATGGTATTTTTTAATGTTGTG

TGATGTATGGGTGACTATATATATATATCAAGATGTTCTATCTCATCTAAGCTGATTAAATAGAATATGTGAATTATTCCTGTACCTATCTTTAATTTTA

TTGATAAAAAAATTATATGCATCATGTATGTATCGCTGCCATATTGCATTATTTTCTGATGTAACGTAATGATACAAAAAGGGAAAGAAAGTTTTTAATC

TAGACTCTAGAGGGTTTTAAATGGTACATTAAGTTCTAATTAGTTCACCCGAGTATTTTACTCGTTTTACTCTAACATTTACCATTCTTTGGGTTTCTTT

TTATTTTTGTGCATAGTTTACTTCTTTTGCGTTGTTGTCGCATTGATTATATGTTAGTGATATTTTTCTTTGGCTGAGTGATACATGCTTTATTTATAAT

TCTAATTCTACAATTAACACATTTGCTTGTTGGATCATCTTTAATGAAGGTTGGAACAGCTGTTGTTACTCGAAGTGATGGAAGATTAGCATTGGGCAGA

ATTGGAGCACTATGTGAGCAGGTATTAAAATAAAAGAAATTTCAGGATTATATATCTTCGGCAGATGCTAATAGGTGTTCTAAGGTCGCTGGTTAAACAA

CTAAGAATAAAAAGTTTTTTATTAGGATACGTAGAATTGCTCTTCCCATGATTTTATTTTTTCCGCTTCCTTATAATTTTCAGAGTAAAAATTTTCTCCT

TTTGATTCCTTAACCAGTACCCTAAGGGCATCAAAACCCTATATCTTGTATATTCCTTACTTTTCATGTATTTGTGTGTGTATATGTGACTAAAAGTTGT

GCTTCCTTTGAAAATTGTTGCAGCTTAAAGAACTAAGCAGTCAAGGGTATGAAGTCATATTGGTAACTTCAGGTGCTGTTGGTCTTGGCAGGCAAAGGCT

TAGATATCGCAAATTGGCCAATAGCAGGTTCTTATATCTTTTGCTATGCTAATTGTATATGTTTCCCAACGACATGAATCAAATTTCTTATCAATGATGG

CTTTCACACTTCGATACTTCAATTTGAAAAAATTGATAAAAGCTTCGGTTGTAGACTTAGAGGTACTTATGCAGAACTGTCTCAAGCTGAGCTGAAAGTA

CTTTAATGTTGTATACTCTACTAGCATGTTTCTCTTTAGAATTTTTAGTCCCAATTGTGTGATTAGGATTGAACCCATGTAGGATTGAAGGAGTTAATGC

AAATTTGTTTCTTTTCAATTAATTTGAGGTTGAATTTAGGGTGAGAATGACGAGCCTTAAAGACATTGTTTACTTGTTGTGATGCAGCTTTTCTGATCTT

CAAAAGCCACAAGAAGAGCTTGATGGAAAAGCATGTGCGGCTGTTGGGCAGAGTAGTCTCATGGCTCTCTATGATACCATGTTCAGCCAGGTTGGCACAA

AACTAAGTTGCTTATTTATTATCATCTCAATTCAATATAAGTGTTTTCTCTTCCTTTATTTACTACATTTGCATGTGAATTTGTTTTTTCCTCCATATTG

ATGTTGATACAAGATTTTTCGTGGTGCAGCTTGATGTGACTTCTTCCCAACTTCTTGTGAATGATGGGTTCTTCAGGGATTCTGGCTTCAGAAAACAACT

TTCAGACACAGTGAACTCATTGTTGGATTTAAGGGTTATCCCCATTTTCAATGAAAATGATGCTGTTAGTACTAGGAAGGCACCATATGAGGTAGGCTGG

TTTTTCTTCAACTATTTCATTTGAATTATCTCTACTTTTTTTTTTTGTTTTAAAATTGTTTAAATTTTGCTTTTGATTACTATCTTAGTTTGTTGAGATG

GTATGATCAAAATGTTTTAGTCAAAACTCTTTTATGTTACACCAACAATATGTTAATTTGATTAACATAAACAAAACAAGCCATGTCCCACTAGGTGGGT

TTGGCTACATGGTTGGTGACTTTGTAATATCTAATACGTGGCTATCTTAAATTTTAAGCACTGTGTGCAGGATTCTTCCGGTATTTTCTGGGACAATGAT

AGTTTGGCTGGTCTATTAGCCCTTGAACTTAAAGCTGACCTCCTTGTTTTATTGAGTGATGTTGAGGGGCTCTATAGTGGTCCTCCAAGTGACCCAAACT

CAAGGTTGATCCATACATATATAAAAGAGAAACATCAAGGGGAAATTACTTTTGGAGACAAGTCAAGATTGGGCAGAGGGGGTATGACTGCTAAAGTTAA

TGCTGCTGTTTGCGCTGCTCATGCTGGCATCCCGGTCATTATAACTAGGTATTTAACATTTTTGTGCTGTGCTAAAATGCTTGCACTGAAGATAACTATT

TTTAGTTTCACATTTTATACCATATATATATTGTTCTAATTGAGTCATTTTTGCATTGACTATTGGGGTAGTCACCCTCTCATGCTAAATTTTTAAAACC

ATGGACAAAACATAAAAAAAAGAGGGTTGAATTAGGTTCTCTTGTGAAATGGTTTCCAAAGGATTGTTCCTTTTTCTTGGTTGTGAAAGTGCATCAATGG

GTGTGTTCCTTCCCCACCCTCTTTTTGATACAACACATAGTTTATGGATATCAGACAAGGGAATGCAGATATTCCATTATGTCAGAGTATATATATCTTA

TGGCCTTAATCAGTTTGAAATAAATGTAATGTGCCATGCTATCTATGGTGGTTCGTGGATCATGTAATTCTAGGGTAAAGAAGAAGACAAAAAAAATTGA

CCTTTTCTTCTATTTGTAAGTTGCAACCATTGGTATATTATCTTTGAAATAGTTCGAGAGATAAAAAGGATTCTTATTTCTGCTGTGAGCAATAATTTGC

ATGTAGTGCATAATATCAAGTATACACTTGGATTTTGTACTTTACTGACTTCGTATTATATTTTAACCTCTGTTGAAGTGGCTATGCTACAAACAACATC

ATACGAGTGCTTCAAGGGGAAAGAATAGGTACTGTCTTTCATAAGGACGCTCATTTATGGACCAATATAAAGGAAGTAAGTGCACGTGAAATGGCAGTTG

CAGCACGTGAGGGTTCTAGACGGCTTCAGGTAAATACAGTGACATGTTGAGTGAATTACTAAATTAATCTTCCAAAGATAGCATGTCAGCATATTTGTCT

TCTAAAGATTTTTGTCCCCAAATGATTCCATCAAAGACTTAAATTGTTGCTATATCATCCTTATAAAACACTAAAGTGGGACGAACTTCACAACTATATT

GCATTTTTGAATTACTAACGTATGTGATGAAAAAAAATCTTTTGAGGAATAATGTGGAGAAATACTAATCTTTGAAGGACCAATTTAAGGCTTTACTTGT

TAAATAGTAAATATGTATTACTGTTATAAGTTTTCCTTAGTCCTTACTTTCATTAATATAATTTTTTTAAGTGGTGATTTATGTTATACTACTACTATTA

CTATTATTATTCTCCACACAATGAACTGAATTAGGGTAGTTATTAGAATTATATTGATGTTTTTGGTGTCAGATCCTCAAATCTGAAGAAAGGAGGAAAA

TATTGCTGGCAATTGCTGATGCATTGGAGACAAGTGAAAGTATGATAAGGCATGAGAATGAAGCTGATGTTGCTGATGCTGTGGCAACTGGATATGAAAA

ATCCTTGATGTCACGTTTAATCCTGAAGCAAGAGAAGGCAAGAACTTAATTGGTTGCATACTAGAGTTAAATACTGAAAGATAGCATACACCACTTTTTT

TGTTGTTACTGTATAACACTGATTTTTCATGTTCCAAAGTATGGTTTTTTTAAACTTTTTTTTTTATGAGAGCATGTTTTAGTCTGATCTTTATTGTTGT

ACTTTACAGATCTCTAGTCTTGCAAAGTCTGTGCGCATGCTGGCAGATATGGAAGAACCAATTGGTCAAATTTTAAAAAGGACTGAGGTAAAAAATCCCT

TTTCTTTTATCCTCCTAATTCTTTTGTAGTCACTAATTTCTTTGTCTTAATGAGTTTCTTCTTTTATTGAAAATAAAAGGCTTATAAGTAACTTCTGATA

CTGGTGATGCGACATTGAAATATTTAACATTTTCCTCTTTTAACATGTTTTAAGTATGTAATATGTTTCCAGTATTTGATAAAGTAACAAATTGTAGAGC

TGATCCTGTAACTTATGTTCACTAATTTTGGTTATAGTCACTGCTTTTACCAAAAAATGAATAATGTTATTAAACTATACATTTTATTGCTCAACACTCT

TTGTGACGGTTCTGATTGTTTTTCTCTTTGTCCAGCTAGTAGATAAACTCATCCTGGAGAAGATATCATGTCCTTTGGGTGTCCTTCTGGTTATATTTGA

GTCTCGACCTGATGCCCTTGTTCAGGTTAATTTAAATTCCCATTCCTTATCTGTCTTTCATCTTCAATAAACTGCTTAGAAGGACAAAGATTTGAGCATG

ATTACAAATTGTCCATTTAGTATAAGTTGATTGTCGTTTCAAATTTCAACATGTCCTATTGGTTTATGCAATCTTCTGACTCTACTTTTATTGATGAAGT

GTGTTGTTTGAAATTGTTTAGTTGTTTTGGATATTTATCTTCAAGTTGTTTGGTTAAACGGATTAACAGATAGCTGCATTGGCAATTCGAAGTGGGAATG

GTTTACTGCTTAAAGGTGGAAAGGAAGCCCGACGATCAAATGCAATCTTACACAAGGTTGGTACTATCTATTGATTTTAGTTGAGACTGGTGTTTTATGA

GATTAATCTGGATCAGATTTGACTGTTGTTTCTTTGCCATCAATTCAGTTATACATATGTTTCTATCTTTCATATTTCTTCTTTTATTGTAGGTCATTAC

TTCAGTGATGCCGGATACTGTTGGTGACAAACTTATTGGGCTTGTGACTTCAAGAGATGAGATTCTAGACCTACTTAAGGTTGGATATCACTAGTAAAAA

ATAAATCCTCATAATTATTGTTTTACAATGGTTTGTCACTTATTATACTCTGTCTCAGCTTGATGATGTAATAGATCTAGTGGTCCCTAGAGGCAGTAAT

AAGCTTGTTTCTCAAATCAAGGAATCAACAAAAATTCCTGTTCTTGGTCATGCTGGTAATGTTCTTCCATATAAATTTCATATTTTGTAGTTAGTATATA

GGCTTTATTTTTTGAATTTGTTTTTTTCATTATGCAGATGGAATTTGTCATGTATATGTTGACAAGTCTGCTAATATTGATATGGCAAAGCAGATTGTTA

GGGATGCAAAGACCGATTATCCTGCAGCTTGCAATGCGATGGTAATTAGCATTTACACATCCTTTGCAATGCACTAAATAAAACATGTTAATCTGTATCC

TCCTCTCCTTTTGGGTTTTACTGAATTGGTAGGTGTGATGTTATTGGAATTTATGGACTATGCTCATCAAAATATTTTTGTTTTTGTATTCTTAGGAAAC

TCTTCTCGTACACAAGGATCTGTCAAATAATGGTGGACTTCATGAGCTTGTCCTTGAACTCCAACGTGAAGGTACGACTTTACTTGCAAATGGATTGATA

AAAACAAACTTTAATTCAAATTGAAGTGTATCTTTTTTAATTTTTATTTTTTAACAATCCCCTTCATGAATGAAAAGAGATTTTGTTGTATTAGTTATGA

TATATATGGTGTTTTCTGTTTTCCCCCCATTTTATCATTAGATCACATCATGGTATTCTGTGTACAAGTAGTTCCCAACTTTTGAGCAATAATAAGTACA

ATGTGCAAGTTAAGGGAAGAAAAAGTGACCATAGTGTATAGAAGAATAAAAAAAAAGTTTGATCTTAAAACAAGCTTTTCAAAGGAAAGAAAAATCATCT

GTGTGAATAAAACTCACTCAAGTTATTCAAATTTCATATAGCAATGGCAAGAAAAAAAGTTGGACCACTTGCATAACAGAAAAGAATAAGTGGATTTGTT

TAATATGAAGCTATCATTTTGGAGTTCACCAAACTGAAATATAATTTCTTTTCTATTTATTTTTGTGTCATGTTTAATTCTCTCAATTTTCTTTTCTTAA

TGCATGGTGCAAGCAATATGCATCATATTTTCCTTCAAAATCAGAAAAGATAAATAAACAGGGATAAAATGGCTTGCAGGTGTTAAAATGTTTGGTGGAC

CAAGAGCCAGTGGCTTACTAAACATTGCAGAAACGAACACTTTTCATCATGAGTATAGTTCACTTGCTTGCACAGTTGAAATTGTAGAAGATGTATTTGC

TGCCATTGACCACATAAATCAACATGGAAGGCATGTTGGGTTCCTCTAAATAAATTTCATTTTCATTCTCTTAACTTCATCATTATTGAGTTTAAAATTC

TCTTACTTTGCTGGTTATGCAGTGCTCATACTGAATGCATTGTTACAGAAGATAGTGAAGTTGCTGAGACTTTCTTAAGTCAAGTTGACAGGTATGTCTA

GAAATTTGACTAAATGTTTGTCTTTTTTTGCCTTTATCTGTTAAATTTATTTTCACATTAGTAAGGATATTTTATTCCCACTCAGCCAACTAAGGGTAAA

TTTTCTTGCCCCAATTTTTTTAGTCTGGTGTCATTCTAACTTGATGACCCCACTTAGCAAGAAAAGGCTTGGTTGTTGTGTCACCATTACTCCATATCTT

GTGAGAAAAAGGCTTTTGTTGTTGTCAGTGTTAGTTACTACTTCATTCCTAAACCTCATCACCATAATAATATGTCCCTTTGCAACTAATATGTCTATAC

TCTTTCAGCTCATGAGATTGAGTTATCAATTGTGGTTTTTGCATTAGTTTGTCTTCAATGAATTGTTCCATTTCATAAACATTATTTTACCCTGTGGAAT

CTGTAAATTTTACAGAATCGTTGTTGTGCAATCCATCTATTTCTCTCTCCAGAAAATCTTTTGTCATTCACCCCATTCTCTCTCTTCCTATATAGTGCCT

CATGTCTAGTCCTAAATGAACAACATTTTGGGATTCAAAATGTTTGTTTCTTAAAATACAGTGAGGATACCAAGTACCAACCATCTTTTATTTGGACAAA

ATTCAATTTTCAACCTATTTTAGCATCCATTCTTTCATAACTTAGACTATGAATAACTCTTGTTGCTTCATGTCATTGACTGGAATATACGCAACTTTGC

CTTGTGTACAAACAGTGCTGCTGTATTCCACAATGCAAGTACACGGTTTTGTGATGGAGCACGCTTTGGGCTTGGCGCAGAGGTAATGCATCTTCCCGAA

ATCTTATTATCATTAGTAACATTGATTTTATTTCCTCATCTGATCTTGGTACTATTAATTGTTGAACAGGTTGGAATTAGTACAAGTCGAATTCATGCTC

GAGGTCCTGTAGGAGTTGAGGGGTTGTTGACAAACAGATGGTATGATATTCTGAGCTCATGTCAAGGCTTTTGTTTTATTTAATGAAGGAAATAATACTT

TCAACTAATATATGCACTTGTTGATACTTGTCATGTGGGTCTGTTCTGTGCTTCATTCTATTTTACTTCAACAATATTATTTGTGCAATTTTTTTTCATC

ATTCTCACAGCACTTCCCTTTTTCAATACCTTGGCAAATAAGGTACAAGAAATTGTGTGGAAAAATGTGCAAGAAACCTATTACTTTTTACCAATCTGTT

ATTCACATTAAAGGCTTACAGTTATATTATTCAGCACTATGTTTTCGCCAATTTTCACATTTTGTGGATATAATGCAGGATATTGAGAGGGAGTGGGCAT

GTGGTTGATGGAGATCAAGGGATCAATTACACTTACAAAGAGTTGCCACTAAAAGCATAACTTTGCATGCAGAACTTAGCTCACTTTCAAGCTATAACAT

GCTCACCTCAACAAAGCAGCATCACTTTTCTGTTCTGTTATGATATGCAGAAGTTGCTGAGATGCTGGCCATGTATAATCGTAACTATCACTTGACCCTT

TCCAGGGCCTTTTTCTTACCTGGAGCAAGCTCAAGGGGGGAGGAAATAATCGTAGGAATGCTACTGTTTGATTATACTTTTATAACATATTCCGAGTTTG

TAAAACAGCAATGCTGTGACAGTTACAAATATTATTCACTACGTACTAGTGAACTTGAATAATTACGGAGAAGTTATTATTATAGAGAATTCAATTTGTT

TTGTGCAGAATTGTTGTTAGTCAAGTGCTGATTCTGCTGCATGTGATTAATTCTGATATATCAGTTGTATTGTAGTTTATTTATTTACTTATTTGGAATG

ATTAGGTCTGTTAGTCAAACTGAATGTTTGTTTACTTGATCTAGTGTTTCTGCTTTAAATTCTTGATTTGATTGGCC

>Glyma.02G251100 | Chr02:43845202..43852326 reverse

TGATTAAACTACTCTTATAACTTTGCAGTATTTTTTTGTACATATATAATTTTGTAGTATTAAGGAGCGATTTGCCACGAAGGTGAAAGAAAGTAACGCG

GCAGGTTGTGATTCGGCGGCGCTATCCACTTTGATTCCATTTGCATTCGAAGGTCTTCTCTGCTCCGCTCCAGTATCAGCACGCAGCCATGGCGGATCCT

TCTCGGAGTTTCATGAAGGACGTGAAGCGCGTGATCATCAAGGTCGGCACCGCCGTGGTCACTCGCGAGGAGGGAAGGTTAGCTGTCGGAAGATTAGGCG

CTCTCTGCGAGCAGATTAAGCAACTCAACTCCCTCGGATACGACATTATTCTCGTCTCCTCCGGCGCCGTCGGCATCGGCCGCCAACGACTCCGCTACCG

CAAATTGATCAACAGCAGCTTCGCCGACCTCCAGAAACCCCAACACGAACTCGACGGCAAGGCCTGCGCCGCCGTCGGACAGAACAGTCTCATGGCTCTC

TACGATACTCTCTTCACTCAAGTAAGAAACCACTTTATAAATTGTTATTACTATTACATTCATGAACAAAATTTAGATTCAGTTTTTTAGATAATGTTTT

CGAATCGGAATTAGAGTTTTGCGTTGGTGATTTGCATTTCAATTCTAATGTCAGAATAACGCTAAAGTCTTCTAGGTTTTGTGCTATATTCATTTTCCTT

GAACTGCACGTGCTGTAATTTTAATTTTTCATTTCGCGGTTTCAGCTCGATGTGACGTCTGCTCAGCTTCTTGTGACGGATAACGATTTTCGAGATAAGG

ATTTCAGGAAGCAACTTACTGAGACCGTGAAATCGTTGTTATCGCTTAAGGTTATTCCGGTGTTCAATGAGAACGATGCTGTCAGCACTAGGAAGGCTCC

CTATGAGGTTTGTTACTCCACTTCCGTAATTTTGTTCGTGGAACCTACCAATGTGTTCTTTGTAGGTCACATGATATGATCCAAATGATGTTGATTCTGT

ATCCGAGGTTTTGTCTGCCTTTTTGCAACCGTAAAGCATATATTCCATCGGCCTCATTTTATTTTGACACTTTTTTGTTTGTCCCAAATTATTTGTTCAC

TCTCAATGAAATGATAAATATACTAGTAGATGTATTGGTGCAATTGTACAGTTTAGTTAACCATTTATGAAATAAGAGTATCTGTTAATCAGATAAGATT

ATTTTTAATCACGATAATAGGTATAATGAATTGCTTTTCTAGATGTGTGTATTACAAGAGAGGGAGTAGCAATTTCTGTAAGGAAAATAAGACTCATTTT

TTCTTCCATTCTTCAACGGATTTTATTTGGTTGTTTGGAGGTCAACATTTTTCATTTGAACTTTTTCTTTTCCAGATTGAAAGTTTAGTTTGAAGTTGCT

GCATTCTTTCCAGTTCTATATATTTAAGTACATCAAGTGTCATTAACGTTTTTTTTGTGCAGGATTCTTCTGGTATATTTTGGGATAATGATAGTTTATC

TGCTCTGTTGGCCTTGGAGTTAAAAGCCGATCTTCTTGTTTTGTTGAGTGATGTAGAAGGTCTTTATAGTGGCCCTCCAAGTGATCCACATTCAAAGCTT

ATTCATACGTATATTAAGGAGAAGCATCAGAATGAAATTACTTTTGGTGACAAATCTAGAGTGGGAAGAGGTGGAATGACTGCCAAAGTAAAAGCTGCTG

TTCATGCAGCTGATGCTGGCATTCCTGTTGTTATTACCAGGTGCACTTTTATACCATATTGCAGTGAAAGATTATTACTCTGCTGTGTTTAGGTTTTAAT

CAAAGCATTTCTTATCAGTGTTGTTTTCAATTAGAAATAGGTTAGTTAGGCCTTGACATCTAAACACTGTTTTGTTAAACCACAAGCAATTTTGGAAACT

TGAAAAATTGTTTAAATTGTTTGCACTTACCTGTTTCTTTATTCCTTGTTCTTCCAATTTCTTTTGTTTGCCTTTTTGTCCGCCACATGTTTCAAGTTCA

TAAATTTAAATGAAAATGTGTGTACATGTCCACATAGAGAATGAGAGAATCACTTGTGAAACCCCCTCCTTTTCTTTTTGATTTTTTTTTCTACCCCAAG

AACCCTATGAAATCAAGTGTGTTATTGGATGATCATCTTTACAAGTTGTTGGTTTATCCTTGTTTCTGTCTCATTTTTTTCTCCTTACAGTGGTTTTGCA

GCTGAGAATATTATCAATGTTCTCCAAGGACAACGCATAGGAACTCTCTTCCATAAAGATGCACATGAGTGGGTCCAAGTAAAAGAGGTTGATGCTCGTG

AGATGGCAGTTGCAGCCAGGGAATGTTCCAGAAGGCTCCAGGTATCTTTTCTATCTTCACATTTACAATATGTCTGACAAATCATAGATATATTACTGAG

ATGTTAGTGAAGCAATCCTAATATCTTAATTTTACTATGCAGGCCATATCTTCTGAAGAAAGGAATCAAATTTTACATAAAATAGCTGATGCCCTGGAAG

CAAATGAAAAAATAATCAGGACTGAAAATGAAGCTGATATTGCTGTGGCACAAGAAGCAGGATATGAAAAATCCTTGGTGGCAAGGCTAGCTATAAAACC

TGGGAAGGCAAGAAAGTTAACTTCTAGAAGAAATTGTCCATATTTTAACAAACTGAAGAGTTTATGATGCTAAACTATGATAACTGACAGATTGCAAGCC

TTGCAAACAACATGCGAATTATTGCCAACATGGAAGATCCAATTGGTCAAGTATTAAAAAGAACTGAGGTAATGGGAAGTTGATCTGGAAATATATATTT

TGGATCATGGTAGTTTCAAAACAACAGTTGCTGAGCCCACTTTCTGAGTATAATCAGCTTCCTTTCTGCATAGTATTAACATCATATTCCAATTTTACAT

CTTAATAGTAGATAAGATAGACTTGCATTTTTCTTGATCTTGTTGAAGGCAAATTAATTTAGTGTAACTTGCATTTTTTTTTTTGGGTGTTTTTAAGCAT

GAACAAATTTCTCGTTGACAGTCATAATTGCACCCAATTTGTTTTACAAAATATTGATCTGTAGTGTTTCTGACAGCTACCCTTTTATTTAGCTATCAGA

TGGGCTAATTTTAGAGAAGACATCATCTCCTTTGGGAGTGCTCCTTATTGTTTTTGAGTCACGCCCTGATGCTCTTGTACAGGTGAAAGCCTTTTTTCGT

ATTTTCTCTTTCTCTTTTCACCCTTTGTTTAAACTTTGTTGAGTACTGCAGAATTTTTTCCCCAAAGGGAAAACAAGCTCTCTTCGTTTAAATTTGTACT

CAGTGTTCAGCATTTATATTCATTTTATCCTTGTTTACTGCTGTTTGAAGTATCAACCTCTAAAGATGTTAGGGAAGATCTGTTTAAAGGCTACCAGATG

AGTGGGTTTTCAAATGAGCTAGATGATATCATGGGTATGGAAATTTAACTCTGATTTATATGGTCCTTAGCAGTAAAAGTAAGAGTTGAAAGAACCCTGT

GTTCCTCCACTTGGGCCATGCAAGCAAAAAAGTGGAATATTTTATTGTATAGACACATGAACTTTTTTGAATTTTGTTTTGCTTCCTATTAACCAGTTAA

TTTTTTTTCTGGCATGGAATTTTCCATAATCTTGAAAATGTTGTCTCATTGACTGTGCTGATTTTTTTTAACCTATTGAATATAATTTTTGATAGCCAAT

ACTTCCACGTCGCTGATGCTGTTGTTTTTCTATGCCAATGTATTGTGACATGGTGATATGCTGGTGTCATATATTTCTCATTTCATTTTGTTCACATCCT

TGAGGAGCATCATTTTTTTACTTATGTTTTCCTTTTCCTTTTCTAAATTTGTGCATGAACCTGTTAGAATGGTATGCATATGTTCGTAATTTATTAAGTG

AATATAAAGTTGTACTGCTAATCACTATTGATCTGTAAATGGATTAATGTTATGGTATGAATGAACTTTATGAGCTTAAAGCAGTTTGATTGGTTATCTG

TATCTACATTTTAAGTGGTAGCTTCTTATCATCTGAACAGATAGCTTCACTGGCAATCCGAAGTGGGAATGGACTTCTCTTGAAAGGTGGCAAAGAAGCT

AGGCGGTCAAATGCAATTTTGCACAAAGTTTGGTTTCAAATAAATTAATTTCTGAACTATTTCCTTTTTCAATTTTATTTTGTTTCATAGTCGCCATTAA

TAAAATATTTATGGGGGTGATTTTGTATCTCTGAGATGCAGGTAATTACTGAGGCCATACCAGATACTGTTGGTGGAAAACTTATAGGACTTGTGACCTC

AAGGGAAGAAATCCCTGAGCTACTTAAGGTGAACTAATCAGTGGAGCTTAATTTCAGTTTCATAACCCTGTTCACCTTAAGCATTGGCACCTTTCTATTT

AAATTTCAGCTGGATGATGTAATCGATCTGGTGATTCCAAGAGGCAGCAACAAACTTGTTTCTCAGATCAAGAGTTCCACTAAAATTCCTGTTTTAGGTC

ATGCTGGTAAGACTATTATGCTTAATGGCACTTTTACTTTGGACTGTGTGATTTTGGTCCCTCAAGTTTTTTCAAGTTGATTAAGTCCCTCTATTTTTGA

AATTAAGCAAATTTGGTCCTGCTGCTAAATTTTTTTTACCTAAGACCATCAAATTCTCATTTTCTACTTAAAATCTTCTCAAATTAAATCGGTTAAATTG

TTTTAAATCATCTTCTACTCAAAACTCATTATTCAAAGCATCAAATTTCATATAAAATAGAAAATTCTCAAAATTTTCAGTTCCAACCATAATCTTATAT

CCTATAAAATCCTTCAAATTATATCAATTTTTTTTATATAAATTTCTGACTTTTAAACTTTTAAAACTATCAACCTCAAATCCATCATAAATCCCTAATA

TTTGGATATAAAATAATAACTTGAAGGTTTGAGATGATTTTTTTTATGACAAGGCAATTTTATTTTTGCTTAATTTCAAAATCTGGACATAATTGGTTTG

AACAAAGTTTGACAGATCAAAATCGAAAACGTTCCAAACTAAATGGACCAAAAATGCAATTAAACCTTTAATATAATATAGAAGCCGCGCTTGATATTAC

TGTATTTCTTTTTTTTTGGATTAATAATCTGCGCTATTACTAATCCAGATGGAGTTTGTCATGTCTATGTTGATAAGTCTGCTAATGTGGAGATGGCAAG

GCGGATTGTACTTGATGCAAAAATAGATTATCCAGCAGCCTGCAACGCCATGGTAAACTAAATGTACTCAATGACCCTACAAGATTGGTAAAGCCAGCAA

TGTCTGATGTACATAACTTCTCTGTTTTCAAATTCCAACATTCAGGAAACACTTCTTGTCCACAAGGATTTGATAGAGAAAGGTTGGTTAAATGATATTG

TTGTTGACCTACGAACTGAAGGTATGATTTATAGCCATCATTTCCTCTTATTCTGTATTTTTTCAACTACATAGGCATTATCTTTTAAAGTTTTTTGGAA

TGACTAAATTATGCAGGCGTTAAATTATATGGAGGACCAAGGGCAAGTTCTCTGTTAAATATTCCACAAGCACAAACATTTCATCATGAGTACAGTTCGC

TGGCTTGCACTGTTGAAATTGTGGATGACGTGTATGCAGCTATTGATCATATAAATCTTTATGGAAGGCATGTCACTGTTTTCTTCTTGATAGGAAACAT

GTTGAAATATATTTTGGTTTCATTTGTCTTCTTTGCTCTTGTCCATTTCAATTGACATCTATGTTAGTGTAGTGAACTTAGATGGAAAAGTGTCATATAT

TCTCTTTCTTTTATGGCAGTGCACATACTGACTCCATCGTTGCAGAAGATAAAGAAGTTGCTAATGTGTTTCTACGCCAAGTAGACAGGTAATATTTGAA

ATATCATGCTTCTTTTTATTTAATGAAAAAAAAAATCCCGGGAAATAAAATAAAAAAGATGTTTCACTCAATTCTCTACTTTGTAGCGTCTCATTATTGT

GTGTAATTTAGGTGGTCTTTGACAAAGTTGCTAATTAGTAAACACAAAACAAACATTTCCTTTAAACTATTAACTGCTTTCCTAAAAGATATCAACGCAC

AAGTACAATGACATTTATGTAAATATTGTTCATCTTGTATTGGCAGTGCTGCTGTTTTTCACAATGCAAGCACCAGATTCAGTGATGGGGCAAGATTTGG

ACTAGGCGCAGAGGTATAATATTGTTTGCATTTTATTTGTAATCAATCTATTGCATTAAGGTTGTTGATTTATTATGTAATTCCCTTCTGGTTGAATAGG

TTGGAATTAGTACGAGCAGGATTCATGCTCGAGGTCCAGTAGGAGTTGAGGGATTGTTAACAACAAGATGGTATGAACTAATATGGTCATTATGCTGATA

AGTTACTAAATTAAATATTTCAGTCCTTCATTTGTCCGTTGTCACTGATAGTGTACACTGTACACCTTAAATATCAATAACTTGCTGCTACACAATCACG

GAAAGTAGCCAAACTAAGACACTGTTTTATTTTAATGGAACTATTTCGCAAGACTACTCTAGAGTCTACATCACTGTCCTAACCATTTATGATTTATAAT

ATAATCATATTGACATTTTATAGTAAAACCCCCGAGTTGTATGTGGTATTTCCTTTGTGTTTTATTGTCCCTTTTGCGTATGCAGGATACTAAAAGGAAG

TGGACAAGTAGTGGATGGTGATAAAGGGATTGTCTATACCCACAAAGACATTGCAACTTAATTTGAATGGTCTAATTGCTATACTTCCTTTTGTAGCCTT

TTCATTTATTTTGACAGTTGAGAACGGTATAAAATTGGGCAATTAAATAAAATAGGGTCATTTGTATCATCTGATGGTTCCTTTCCTTCAAGAATTTTGT

CAGCAATTACAGTTTGTTGTTGAATTTGTTATTTAACGCATCCGTTTCTGTTATGGCATACGAATAAAGCACCACCTCTAGGAAAACAGAGAATATGAAG

TCTCCGCAATGGAATCCATTTATTGCTTTGTTAACGACAATTGGATATTTATAGTTTCAGTTTTTGAGATGTTATTTTTCTTTATATACAACGTTGTATT

TTTCCCACAGAAAGGGAAGAGGTTG

>Glyma.03G069400 | Chr03:14507722..14520067 reverse

TCTCACACCAGTGGCTTCTATTTCATTCATTCATCTCTCATCTGAAACACAACAAGAGAAACAATGGAACTCTTGCAAAATGGCCACAAAAATTTGGTCA

GCATCAAACCCTCCGAGTTACCCCTCTTGAATGGGGCCGCGTTAACTCTCCTCAACTCGCTCTCCGAAACTCATGAGTACTATGGCAACATAGATCCTTC

CAGAGTTTTTGTCACCAAAGTGAAGCGTATCATTGTAAAGGTACTGCTTTTAATTTATCATTGTTTTATATTATTTATTTTATTCATAGAAATCAAACTC

AAACTCTAAGACAAGTTTATATCAATTTATATATCAGTACTATTTCTCCTTTTTTTAATCGAGGAAATTATACAAAATGAGGGTAAGAAGAGTTTGTTTA

CTTGGATTTTCATTTTTAATATCTTTGTTTTTAATGTGCTTTTTATTTTTAAATGGTATAGTTTTTTTTCTCTTTTTTACAGGTCAAAGAAAATATAGAT

GCATCACAATAAGTGTCCAATTAATTATGAGAACTTTCTCTCTTCATTTTATGTATAAATATTTGAAAGTAGAAAACAAATTGAAAATAAAATGAAAATT

TTGTCAATTAAAAATAAAAACAGAAATAAATAGGCCTTAATTTTCAAAAGAAAAATATATGATTTTTTTTCTTTAATTTAATTCTCAATCTTTTCTAAAT

TTCTCGTTTTGTTTTCTCTGCATCAAAATGAAGTAAGGGAGAAGAGGCCCTTTCTAGTTTCCTTTTACACACTTTTTTTTTTTTTTTTCATATCAATGTC

GCAGTTTCCTCAACAATCAAAACGTCATCAGTTTTTGATAGCACTGCATTGGCATAAAGTAAATGTGTGTAGTAATTTTGTTTTAATTACTGGATTTTTC

AGTGAATTTCCCTATTTATTTGTGAATTTCTTTTTTTTTACGAACATGACAAATTTTAGAAAGGCGAAAATTTAATGTTAGTACCTGCCACTTAAATGTT

AATTATTTGGTTGTGTAAGGTGTGATATGATTGTTTAATGTTTATATTTTCTTAAAAAACAAGGTATTATTAAGAAATGACGTGCGTAATTATAAATTTT

TTCAAAGAAGAATTTGAAGTTTTAGTTTATATTTATTTACTATTATAAATTATTTTTTTAATTTGTTTACTGCCCCAAAAGCAAAAGTTTCGGTACGTTG

CACATGTCTCTGACAGACGGGCTATATTTTTTAATGTTTTATTTGAATATGAGTTGAAATTGAGAGGAAAAAAAAAAAGAAACAAAGAAAAAAACAAGAA

AGAAATAAAAGTAAAATTTAGTTGAAAATTTATGTTGTTTGGATCGACAAAATTAATAGAGGAGAGTTCCTTTTCAATTATTTGGTTATATTGAAAATAA

AGGAAGAGAATGAAAAAACTTGTAAAATCATAATTGTATCACGGATGAGATAACTTGACGGAAATACATAAATAAATAAAGATGGAATAGAAACATTAAT

ATCTATTGTAAAAAACGAAACATTTTTTTTCTGAATAAAAAGAGACATTAATATATGAAAAAAGGATTATGTATTGAAAGTGTATAAATATTTCTTTTTG

CAAATCTTTTCTCTTTTTGCCTGGATTAAAAACAAAAAAAAGGAGTGCTATAAAATATGCAATGGATATATTTCTTCTCTTTCATCTCTTTCTTCTCTTT

CAATTTCATTCTTTTCCATTTCTCTACAAAGAAACAAAGGCTAAGAGTGCTAAAAATATTCACACGCTTACTCATGCATTGCTTCATAATTATGTTATAT

TTACATTGATTGTAATTAGTTTTATATTATTTTTCCTCACTTATAATATAGTTTTATCGTACATACTTTAAATATTAAGTAATATACTAACTCATAGTCA

TTGTTAAGATATAAATAATTCATTCGAAGTTATACGTTTTTAAAGTATATTAAAATTGAATTTGACAGAATTAATTTTAAACCGTAAAGTTAATTAAAAT

GTATATTTAAACAAATATATACTATAAATTTACATTAATATATTTTTATTAAAAGACGAAAATTAATATTCTAAAATTTCTTTTCAAAAGTAAAATATAA

TCAAGCAAGCACACATCTAAATTTTTTAATTCTTATTTTGTAGAATAATTTTTAAAAATAACAAACAAATAAGTTCCGAGCTTAGCAATTTAAAAAACCA

AAAAAAGAGTCTTAATGTGATTAATATCAAACAATTCAAAAATAAAAGATGAGTATAGAAGATGTATTTGTTGCGAATGATGGTTAAGGATTGAATAATA

AAAAAAACTTATCTTCTAATATGAATGCGATTTTGAATATTCCGAAGTCTTCTAAATTTTTTTGGTAGACAGTACTCTCCGCATAAACCATTTGATAAAA

TTATTTGTGGTTTAGGTTTGTGTTGGACTTTTGTCAATCTTTCCATCGTCCTTTTAGCACTCCTCCCCGGAATGATGAGATTTAGATAGATTCCCTCACC

CTTACATTATTTAGACTAACAGGAAAAAAAAAAAGAGAGCAAGGAAATGTATTGATAAAGATAATTAATTAATTTAAAAGTGAGGAGAGCACTTGATTCT

CGTTTAAAATTTGAAAAACAAGTTTCTAATTTGGTCAAAAAAACCAATTTTAATCTATAAATAAGCATAATATTTCTCATTTGCATAAAAAATGGATGTT

GAAATTAGTTTGAAGATATTTTGAATTAAGAAAAACTTAGCTTAGGTCATGTTTAAGAAAGATATCTTTTTAGTTATGATTGGAAACTGAAAAATATCTT

TTTAGTTAATAAAAAGAAATTTTATTAAAATAAATAATTTAATTTTAGATAAAAATGATAAATGATAAATTTTATTAATTTATTAAAAATAAATATAAAA

ATGATAATCGTATATTTAAATTATGTTTAAAAATAAATCTTACAAGTTAATAAGAAAAAATCATTTTTTACACACTTTAATTTGATCAAATAAATTTTTA

AATTTATCAAAAAATTAAAATTTAATTAAAATAACTTGATTAATATATATGTAATTTACTTTTATATAAATTTAAAAAAATTTATCTTATTTATTATTTT

AAAGATAATTTCTCATTCAAGATAATTAAGTTAATAATATTTTTTTTATTGATAAGAAAAAAAATACTATTTAAAATTTATAATAATTTACCTCTCAATC

TATTATATTTAATGAACCGAACTATATTAATAATAATAAGATAATATAATAAGTAAGATAAAATTATTATATTTTTACATAATAATAAACAATGAATTAT

AACTGTTATAGAATAATTATTTTATTTTATTTTATCTTATCCAAGTCACAAGAGACCCTTGTTTTGATCATATTCATGAAACGCTTGATTTAAATCTAAA

AGATACTTTTCTTTCAAAACAAAAAAGAAAATCTTTTTAATTTGTAACGAAAAAAAAATCTAAAAGATCTTTGAAAAATGAATTAGAAAAGAGAAAAAAA

AAATTATAACTGTTAAAGAATAATTATCTTATTTTATTTTATCTTATCCAAGTTACAAGAGACCTTGTTTTGATCATATTCTTGAAATGCTTGGTTTAAA

TCTAAAAGATATTTTTCTTTCAAAACAGAAAAGAAAATCTTTTTAATTTGTAACGAAAAAAATCTAAAAGATCAATGAAAAAAGAAATAGAAAAGAGAAA

AAAGATTCAAGATAATGAGAATTCAGTCGAATAGAAAGTGGTGTGAGTGGGTGAAGTAGCAGCAACAAGAACAGTACCCTCGATTCGTGGGGCCCACAAA

GCCAAGTATTACCCAGTCTCTGAAGCCACCTATAACTATTTATATTTCTTCTACGCATTTTCACGCACTCAGTCTTCTTCTATTTATTTCACACCCGTTT

CGACCAGGTCCTATTGCATTGGACTTCATGGATCCCAGTCGAGATTTCATCAATTCTGTCAAGCGTGTTGTTGTCAAGGTCCCACAACATTTTCATTATT

TTGTAATTATTAAGTACTATTATGATTACGATTGAACCAACAAGTAAACAATGGATCAAACAATTGCAAGCCATTTATATCCCTTAATTAAGTAGTTTAA

GATTTGAATTCTGATTCATCTTAATATAAAATAAATTATATTGGAAGGAAAATACATATTTTGTAGTTTCCGACGAAGATTAATCCACGGAGACTACTTC

GTACAAATACCTTATTTACAATTTTTATTATGATTATGATTGTTATTTACAACATGCATTCTCTTGTATATTGGACATAGATTATTTTTTATTTGTGATT

ATTGTTATGGAAAAGATGTCACCTATTTTTCTAGTTAAAAAATTTGCCTTTTATTTTTGTTTCTTATAATTTGATGACTTTCTTAATATTAATATGTGTG

ATGTATGATGTATCCACAAACATAAATATGTCTGTGTATATTAAGAGAGTCTAATTCAATTGATTGGATAAAATGTGAGTTGTTATAATTGTCTAGTACC

TATATTTGATTCCTACTAATAACAAAAATAAAATACGAATGTATCATGTATGTATCTATTGTTGGAATATATTGTATTATTTTCTGATGTAATGTAAAGA

TACAAAAAAGGAAAGAAAGTTTTTAATTTAGAGGGTTATAAATGGTATATTAAGTTCTAATTAATTCACCTGAGTATTTAATTCGTTTTGCTCTAACATG

TACCATTCTGCTGGTTTCTTTTTATTTTTGTGCATAGTTTACTTGTTTTGTGTTATGTCGCATTGATTATATGTTAGTAGTATTTTTCTTTGGCTGAGTG

ATACATGCTTTGTTTATAATTCTAATTCTATAACTAGTACATTTGTTTATTGGATCATCTTTAATGAAGGTTGGAACAGCTGTTGTTACTCGAAGTGATG

GAAGATTAGCATTGGGCAGAATTGGAGCTCTCTGTGAGCAGGTATAAAAAAAATTCAGAAATATATATCTTTTATATTCCTTATATATAATGTATTTGTG

TGTATGTGACTAATTTCCAATGAAATTGTTGCAGCTTAAAGAACTTAGCAGTCAAGGGTATGAAGTCATATTGGTAACTTCAGGTGCTGTTGGTCTTGGC

AGGCAAAGGCTTAGATACCGCAAATTGGCCAATAGCAGGTTCTTATAATCTATCTTTTGCTATGCTAATTGTATATCTTTTCCAATGACATGAATCAAAT

TTCTAATCAACAATGGCTTTCACATCTTTGATTACTTGAATTTGAAAGAAAAAAAATGATAAAAGCTTCACTTGTAGACTTGTAGGTGATGCTCCTAAGC

AAATATGAGATTTCGAATGCAATATGAATGGAGAAAAAGAACTGTCTTAAACACAACTGAAAGTACTTCAATGTTTTATACTCTACTAGCATGTTTCTCT

TTAGAACTTTTAGTCCAAATTGTGTGATTAGGGTTGGACCTATGAAGGATTGAGGGAGTTAATGCAAATTTGTTTCTTTCCGATTAGTTTGAGGTTCAAT

TCAGGGTGAGAATGACTAGCCTTAAAGACATTGTTTACTTGTGAATTGTGATGCAGCTTTTCTGATCTTCAAAAGCCACAAGGAGAGCTTGATGGAAAAG

CATGCGCGGCTGTTGGGCAGAGTAGTCTCATGGCTCTCTATGATACCATGTTCAGCCAGGTTGGCACAAAACTAATTAAGTTGCTTATTCATTATCATCA

CAATCCATTATAAGTGTTTTCTTTTCCTTAATTTGCAAGCGATTTTGTTGTTTCCTCCATATTGACGCTGATACCAGATTTTTTGTGGTGCAGCTTGATG

TGACTTCTTCCCAACTTCTTGTGAATGATGGGTTTTTCAGAGATTCTGGCTTTAGAAAACAACTTTCAGACACGGTGAACTCATTGTTAGATTTAAGGGT

TATCCCCATTTTCAATGAAAATGATGCTGTTAGTACTAGGAAGGCACCATATGAGGTAGGCTGGTTTTTCTTCAACTATTTCATTTGAATTATCTCTATT

TATTTATTTCAAAATTGTTTAATTTTTCTTTCGATAACTATCTTTCCTTTGTTTGTTGAGATGATATGATCAAAATGTCTTAGTCAAAACTCTTTATGTT

TCACCAACAATATGTTAATTTGATTAACATAAACAAAACAAGCCATGCCCCACTAGGTGGGGTTTGGTTACATGGTTGGTGACTTTGCAATATCTAATAT

GCGGTTATCTTAAATTGTAAGTGTTGTGTGCAGGATTCTTCGGGTATTTTCTGGGACAATGACAGTTTGGCTGGTCTATTAGCCCTTGAACTTAAAGCTG

ACCTCCTTGTTTTATTGAGTGATGTTGAGGGTCTTTATAGTGGTCCTCCAAGTGACCCAAACTCAAAGTTGATCCATACATATGTAAAAGAGAAGCATCA

AGGGGAAATTACTTTTGGAGACAAGTCAAGATTGGGGAGAGGGGGTATGACTGCTAAAGTTAATGCTGCTGTTTGCGCTGCTCATGCTGGCATCCCGGTC

ATTATAACTAGGTATTTAACATTTTGGGCTGTGCATTTAGTCATATAGGCACTGAAGATAACTATTTTTAGTTTCACATGTTATACCACATATATATTGT

TCTAATTGAGTCATTTTTGTGTTGACTATTGGGGGAGTCACCCTCATGCTAAATTTTTAAAACCACGGACAAAACATAAAAAAAAGGTTGAATTAGGTTC

TCTAGTGAAGTTGTTTTCAAAGTATTGTTCCTTTTTCTTGGTTGTGGTGCATCAATGGGTGTGTTCCTTCCCCACCCTCTTTTTGATACAACACTTAGAT

TATGGATATCAGACAAGGGAATGCAGATATTCTCCATGATGTCAGAGTATATATATCTTACGGCCTTGATCAGTTTGAAATAAATGTAATGTGTCACGCT

ATCTATAGTGGCTCGTGGATCATGTAATTCTAGAGTAAAGGAAAAGAGATTTTTTTTCCCTTTTTACTTGCAAGTTGAAACCTTTGGTATCTATTATCTT

GGAAATAGTTCAAGAGATAAAAAGGATTCTTATTGTTGTGACTTGTGAGCAATAATTTGCATGTAGTGCATAATATCACGTATACACTTGGATTTTGTAC

TTTACTGACTTTGTTAATATATTTCAACCTCTGTTGAAGTGGCTATGCTACAAACAACATCATACGAGTGCTTCAAGGGGAAAGAATAGGTACTGTATTT

CATAAAGATGCTCATTTATGGACCAATATAAAGGAAATGAGCGCACGTGAAATGGCAGTTGCAGCACGTGAGGGTTCTAGACAGCTTCAGGTAAATACAG

TTACACGTTGAGTGAATTTCTAAATTGATCTTCCAAATATAGCATGTCAGCATATTTGTCTTCTGAAGATTTTTGTCTCCAAATGAGTCAATCAAATACT

TAAATTGTTGCTATATTCATCCTTATAAAACAGTAAAGTGAACGAACTTCAAAACAACATTGCATTTTTAAATACTAACGTATGTGCTGAAAATAATCTT

TTGAGAAATAACGTGAAGACATACTAATCTTTGAAGGACCAATTTAAGGCTTTGCTTGTTAAATAATAAATATTAGTATTAGTGTTATTAGTTCGCCATA

GTCCTTCCTTTCATCAATATAAAATTTTAGGTGGTGACTTATATTATACTACTACTGTTACTGTTATTATTCTCAATGCAATGAACTGAATTAGGGTAGT

TATTGGAATTATATTGATGTTTTTGGTGTCAGATCCTCAAATCTGAAGATAGGAGGAAAATATTGCTGGCAATTGCTGATGCATTGGAGAAAAATGAAAG

TATGATAAGGCATGAGAATGAGGCTGATGTTGCTGATGCTGTGGTAGCTGGATATGAAAAATCCTTGATATCACGTTTAACCCTGAAGCAAGAGAAGGCA

AGAACTTAATTGGTTGCATACTAGAGTTAAATACTGAAAGATAGCATACACCACCTTCTTTGTTGTTACTGTATAACATTGATTTCTCCTGTTCCAAAGC

ATGGCATTTTCTAAGCTTTTTTTTGTGAGCATCTTTTAGTCTGACCTTTATTGTTGTACTTTACAGATCTCTAGTCTTGCAAAGTCTGTGCGCCTGCTGG

CAGATATGGAAGAACCAATTGGTCAAATTTTAAAAAGGACCGAGGTCAAAAATCCCTTTTCTTTTATCCCCCTAATTCTTTTGTAGTCACTGATTTCTTT

ATCTTAATGAGTATCTTCTTTTATTGAAAATAAATAGCTTATAAGTAACATCTGATATTGGTGATGTGACATTGAAATATTCAACATTTTCCTCTTTTAA

CATGTTTTAAGTATGTAATATGTTTCCAGTATTTGCTGAAGAAACAAATTGTAGAGCTGATCCTGTAACTTAACTTTGGTCTATAAGAACAAATTGATGT

CACTGCTTTGACCAAAAAATGAATAATGTTATTAAACTATACGTTCTATTGTTCAACACTCTTTGTGACAGTTCTGATTGTTGTTCTCTTTGTTCAGCTA

GTAGATAAACTCATCCTGGAGAAGACATCATGTCCTTTGGGTGTCCTTCTGGTTATATTTGAGTCTCGACCTGATGCCCTTGTTCAGGTTAATTTAAGAT

TCCTTTCCTCTTATTTGTTTTTTCAACTTCAATAAACTGCTTAGAAGGACAAAGATTTGAGCATGATTACAAATTATCCATTTAGTATAAGTTGATTGTC

ATTTCAATATATCATCAAATTTAAACATGTCCTATTGGTTTATGCAATCTTCTAAGTCTACTTTTATTGATAATGTATGTTGTTTGAAATTGTTTAGTTG

TTTTGGATATTTATCTTTAAGTTGTTTGGTTAAATTGATAAACAGATAGCTGCATTGGCAATTCGAAGTGGGAATGGTTTACTGCTTAAAGGTGGAAAGG

AAGCCCGACGATCAAATGCAATCTTACACAAGGTTAGTACTATCTAATATCTATTTATTTTAGTTGAGATTGTTGTTTTATGAAATTATTCTGGATCTGA

ATTGACTGTTGTTTCTTTGTCATTAATTCACTTATACATATTTTCTCTCATTTGTATTTCTTCTTTTATTGTAGGTCATTACTTCAGTGATGCCAGATAC

TGTTGGTGACAAACTTATTGGGCTTGTGACTTCAAGAGATGAGATTCCTGACCTACTTAAGGTTGGATATCACTAGTAAAAAATAAATCCTCAAAATTAT

TGTTTTACAATGATTTGTCACTAATTATACTCTGTCTCAGCTTGATGATGTAATAGATCTAGTGGTCCCTAGAGGCAGTAATAAGCTTGTTTCTCAAATC

AAGGAATCAACAAAAATTCCTGTTCTTGGTCATGCTGGTAATTTTCTTCCATATAAATTTCCTATTTTGTAGTTAGTAAATAGCCTCTCTTTTTTTACTG

AATTTGTTATTTTTGTTATGCAGATGGAATTTGTCATGTATATGTTGACAAGTCTGCTAATTTTGATATGGCAAAGCAGATTGTTAGGGATGCAAAGACT

GATTATCCTGCAGCTTGCAATGCAATGGTAATTAGCATTTACACATCCTTTGCAATGCAACAAGTAAAACATGTTCATCTGTATCCTCCTCTCCTTTTAG

GTTTTACTGAATTGGTATGTGTGATGTTATTGGAATTTATGGACTGTGCTCATCAAAATATTTTTGTTTTGGTATTCTTAGGAAACTCTTCTCATACACA

AGGATCTGTCAAATAATGGTGGACTTAATGAGCTTGTCCTTGAACTCCAACGAGAAGGTAGACTTTGCTTGCAAATGGATTTATAACAAAGAATGTTGGT

TCAAATTGATGTTTATCTTTTATTTTGTTTCGATTTTTAAACAATCCCCTTCATGGATGAGAAGAGATTTTGTTGTATTAGTTTTATGATATATATGTTG

TTTTCTGTTTCCCCCCATTTTATCATTAGATCACATCATGGTATTCTGTGTACAAGTAGTTCCCAACTTTTGAGCAATAAACAGAAGTACATTGTGCAAG

TTAAGGGAAGCAAAAGTGACCATAGTGTATAGAAGAATAAAGAAAAAGTTTTATCTTAAAACAAGCTTTTCAAAGGAAAGAAAAATCATCTGTGTAAATA

AAACTCAGTTAAGTTATTCAAATTTCATATAGCAATGGCAAGAAAAAAAGTTGGACCACTTGCATAACAAAAGTTGGAGTTCACCAAACTGAAATATAAT

TTCTTTTCTATTTATTGTGTCATGTTTAAATCTCTCAATTTTCTTTTCTTAATGAATGGTGCAAGCAATGTGCATCATATTTTCCTTCAAAATCAGAAAA

AACATGAATAAACAGGGATAAAATGGCTTGCAGGTGTTAAAATGTTTGGTGGACCAAGAGCCAGCGGCTTACTAAACATTGCTGAAACGAACACTTTTCA

TCATGAGTATAGTTCACTTGCTTGCACAGTTGAAATTGTAGAAGATGTATTTGCTGCCATTGACCACATAAATCAACATGGAAGGCATGTTGGGTTCCTC

TAAATAAATTTCATTTTCATTCTCTTATCTTTATCATTATTGTGTTTAAAATTCTCTTATCTTGCTGTGCAGTGCTCATACTGAATGCATTGTTACAGAA

GATAGTGAAGTTGCTGAGACTTTCTTAAGTCAAGTTGACAGGTATGTCTAGAAATTTGACTGCTTGCTTGTGTTTTTTGCCTTTATCTAATTGTTAGATT

TATTTTCACATTAGTATTGGTATTTTATTCTCACTCAGCCAACTAAGGGCTAATTTTCTTGCCCCAAAATTTTAAGTCTGGTGTCACTCTAACTTTGTTG

AGTCCACTTACCAAGAAAAGGCTTGGTTGTATCACCATGACACCATGTCTTGTGGGAAAAAGGCTTTTGTTGTTGTCATTGTTAGTTACTACTTCATTCC

TATACCTAATCACCTTTATAATATCTCTTTTGCAACTGATATGTCTATACTCTTTCAGCCCATAAGATTGAGTTATCAAGTGTGGTTTTGCATTAGTTTG

TCTTCAATGAATTATTCCATTTCATAAACATTACTTTACCATTCCCCTTAAAATCACTCCATAGATCTTAGTGTCCCGTGGAATCTGTAAATTTTACAGA

ATCATTGTTGTGCAATCCATCTATTTCTCTCTACAGAAAATCTTGTCATTCACCCCATTCTCTCTCTTCCTATATACTACCTCATGTCTAGTCCTTAATG

AACAACATTTTGGGATTCTGAAACTTTGTTTCTTAAAATACAGTGAGGATACCAAGTAACAACCATCTTTTATTTGGACATAATTCGATTTGCAACCTTA

AATAGAATTCATTCTCTCACAACTTAGACTATGAATAACTCATGTTGCTTCATGTCATTGACTTGAATATATGCGACTTTTCCTTGTGTACAAACAGTGC

TGCTGTATTCCACAATGCAAGTACGCGGTTTTGTGATGGAGCACGCTTTGGGCTTGGTGCAGAGGTAATGTATCTTCCCGAAATGCTATCATTATTAGTG

ACAGTGATTTCATTTTCTCATCTGGTCTTGGTTATATTAATTATTGAACAGGTTGGAATTAGTACAAGTCGAATTCATGCTCGAGGTCCTGTAGGAGTTG

AGGGGCTGTTGACAAACAGATGGTATGATATTTTTGAGCTCATCATATCAAGGGCTTTTGTTTTAACGAAGGAAATAACACTTTAAACTAAAAATATATG

CACTTGTTGATATTTGTCATGTGGATCTGTCCTTTGCTTCATTTTATTTTTACATTAACAATATTATTTGTACACATTTTTTTCCATTATTCTTACACAA

CTACCCTTTTTGGGTAATAAACAAAGGGAAACACACATTTATTTGCATTATTCAGTGCTATGTTTTGCCAATTTTCATATTTCGTGGATATAATGCAGGA

TTTTGAGAGGGAGTGGGCATGTGGTTGATGGTGATCAAGGGATCGATTACACTTACAAAGAGCTTCCACTAAAAGCATAGCTTTGCAAGCAGAGTCTTAG

TTCACTTTCAAGCTAAATATGCTCCACTTCAACAAAGCAGCATCACTTTTCTGTTCTGCTATGATATGCAGAAGTTGCTGAGATGCTGGCCAGCTATAAT

CGTGACTATCACTTAACCCTTTCCAGGGCCTTTTTCTTACCTGGAGCAAGTTCGAGTGGGGAGGAAATAATCATAGGAATGCTACTGTTTGATTATGTTG

CACTTTGGTGCTCTTTTATAAAATATTCCGAGTTTGTAAAACAGCAATGCAGTGACAGTTATAAATATTATTCACTACGTACTAGTGAACTTGAATAATT

ATGGAGAAGTTTTTATTATAGAGATTAAAAAATCTAATATGTGAAA

>Glyma.14G065600 | Chr14:5389804..5397475 forward

TGAAAGAGAGCAACGGGGCAGGTAATGATTCGGCGGCGCTATCCACTTTGATTCCATTCGCATTCGCAGGTCTACTGTAGAATCAGCACGCAGCCATGGC

GGACCGTTCTCGGAGCTTCATGAAGGACGTGAAGCGCGTGGTCATCAAGGTCGGCACCGCCGTGGTGACTCGCGAAGAGGGAAGGTTAGCGGTCGGAAGA

TTAGGCGCTCTCTGCGAGCAGATTAAGCAACTCAACTCTCTCGGATACGACATTATTCTTGTCTCCTCCGGCGCCGTCGGCATCGGCCGCCAACGACTCC

GCTACCGCAAATTGATCAACAGTAGCTTCGCCGACCTTCAAAAACCCCAACTCGAACTCGACGGCAAGGCATGCGCCGCCGTCGGGCAGAACAGTCTCAT

GGCTCTCTATGATATTCTCTTCACTCAGGTAAGAAACCACTTATAAATTGTAATTACTATTACATTCATGAAAATCAAAATTTAGATTCAGTTTTGTAGA

TAATGTTTTTGAATCGGAATTAGAGTTTTACATCGCTAATTCTAATATAGGAATAACGCTAACTAAAAGTCTTCTAAGTTTTGTGCTATATTCATTTTCC

TTGAACTGCACTTGCGGTAATTTTAATTTTTCGTTTCGCGGTTTCAGCTCGATGTGACGTCTGCTCAGCTTCTTGTGACGGATAACGATTTTCGAGATGA

GGATTTCAGGAAGCAACTTACTGAGACCGTGAAATCGTTGTTATCGCTTAAGGTTATTCCGGTGTTCAATGAGAACGATGCTGTCAGTACTAGGAAGGCT

CCCTATGAGGTTTGTTACTCCGCTTCCCTGATTTTGTTCGCGGAACCTACCAGTTTAAATGTGTTCTTTGTAGGTGACATGATATGATCCAAATGCTGTT

GATTCTGTATCCGAGGTTTTGTCTGCCTTATATTCCATCGGTCTCATTTTATTTGTCACTTTTTTGTTTGTCTCAAATTATTTGTTACTTTAAAATATCA

AGAAAGTATTGATTGATTACTTTTTTCCGATAACACTCTCAAGAAATAATAAATATACTAGTAGATGTATTGGTGCAATTCTACAGTTTAGTTAATCATT

TATGAAATAAGAGTATTTGTTAATCAGATAAGATTATTTTTATCATGAGAGTAGGCATAATGAATTGTTTTTCTAGATGTGTGTATTACAAGAGAGGGAT

TAGCAATTTCTGTAAGGAAAAATAAGACTCATTTTTTTCCTTCTTCAACGGATTTTATTTGTTTGTTTGGAGGTCAACATTTTTCACTTGAACTTTTTGT

TTTTCAGCTTGAATGTTTAGTTTGGAATTGCTACCTGGAATACTTGCTTTATTGTTTCCATTTCTATCTATTCAAGTACATCAAGTGTCATTAACGTATT

TTATGTGCAGGATTCTTCTGGTATATTTTGGGATAATGATAGTTTATCTGCTCTGTTGGCCTTGGAGTTAAAAGCCGATCTTCTTGTTTTGTTGAGTGAT

GTAGAAGGTCTTTATAGTGGCCCTCCAAGTGACCCACATTCAAAGCTTATTCATACATATATTAAGGAGAAGCATCAGAATGAAATTACTTTTGGTGACA

AGTCTAGAGTGGGAAGAGGTGGAATGACTGCCAAAGTAAAAGCTGCTGTTCATGCAGCTGATGCCGGCATTCCTGTTGTTATTACCAGGTGCACTATTAT

ACTATATTACCGAGAAAAATTATTACTCTACTGTATTTAGGTTTTAATAAACCATTTCTTATCAGTGCTGTTTTCAATTAGAAATAGGTTAGTTAGGCCT

CGACATCTTAACAGACTGTTTTGTTAAACCACAAGCAATTTTGGAAACTAAAAAAAAAATTGTTTAAATTGTTTGCACTTACCTGTTTCTTTACTCCTTA

TTCTTCCAATTTCCTCTTTTCTTGTCTTTTGTCCACCACATGTTTCAAGTTCATAAATTTAAATGAAAATGTGTGTACATGCCCACATAGAGAATGAGAG

AATCACTTGTGAAACCCCTCCTTTTCTTTTTCATTTGTTGTTCTACCCCAAGAACCCTATGAAATCAAGTGTGTTATAAGTACACTGACTATCATCTTTA

CAAGTTGGTTTATCCTTGCTTCTATCTCATTTTTTTCTCCTTACAGCGGTTTTGCAGCTGAGAATATTATTAATGTTCTCCAAGGACAACGTATAGGAAC

TCTCTTCCATAAAGATGCACATGAGTGGGTCCAAGTAAAAGAGGTCGATGCTCGTGAGATGGCAGTTGCAGCCAGGGAATGTTCCAGAAGGCTCCAGGTA

TCTTTTCTATCTTCACATTTACAAAATGTCTGACAGATCATAGATATATTACTGAGATGTTAGTGAAGCAATCCTAATATCTTAATTTTACTTTGCAGGC

CATATCTTCAGAAGAAAGGAAACAAATTTTACTTAAAATAGCTGATGATCTGGAAGCTAATGAAAAAATAATCAGGACTGAAAATGAAGCTGATGTTGCT

GTGGCACAACAAGCAGGATATGAAAATTCCTTGGTGGCTAGGCTAGCTTTAAAACCTGGGAAGGCAAGAAAGTTAACTTCTAGAAGAAATTGTCCATATT

TTAACAAACTGAAGAGTTTATGATGTTAAACTATGATAACTGACAGATTGCAAGCCTTGCAAACAACGTGCGAATTATCGCCAACATGGAAGATCCAATT

GGTCAAGTATTAAAACGAACTGAGGTAATGGGAAGTTGATCTGGAAATATATATTTTGGATCATGATAGTTGCAAAACAACAGTTGCCAAGGTCACTTTA

TGAGTTTAATCAGCTTCTTTTCTGCATAGTATTAACATCATATTCCAATTTTACATCTTAATAGTACATAAGATAGATTTGTGTTATTCATGATCTTGTG

GAAGGCAAATTAATTTAGTGTAACTGTTGCATTTTTCTGTGGTGTTTTTAATTATGAACAAATTTCTCATGGACACTCATAATTGCACCAAATCTTTTTT

TACAAAATATTGATCTGTAGTGTTTCTGACAGCTACCCTTTTATTTAGCTTTCAGATGGGCTAATTTTAGAGAAGACATCATCTCCTTTGGGAGTGCTCC

TTATTGTTTTTGAGTCACGCCCTGATGCTCTTGTACAGGTGAAAGCCTTTTTCAAATTTTCTCTTTCTCTTTTCACCCTTTGTTTAAACTTTGTTAAGTA

CTGCAGAATTTTTTCCCCAAAGGAAAAACATTCGCTCTTCATTTAATTTTGTACTTAGTGTTCAGCTTTTATATTCATTTGTTATCCTTGTTTACTGCGG

TTTGCAGTATTAACCTCTAAAGGCGTTAGGGAAAATCTGTTTAAAGGCTACCAGATGAGTGAGTTTTCAAATGAGCTAGATGATATCATGGGTATGAAAA

TTTAACTACGATTTATATGGTCCTTAGCAGTAAAAGTAAGAGTTGAAAGAACCCTGTGTGCCTCCACTTGGGCAATAGAAGCAAAAAAGTGGAATATTTT

ATTGTATAGACACATGAGCTTTTTGAATTTTGTTTCGCTTCCTATTTACCAGTTAAATTTTGTTCCTGGCATGCAATTTTCCATAATCTTGAAACTGTTG

TCTCATTGACTGTACTGATTCTTTTTAACCTGATGAATATAATTTTTGATAGCCAATACTTCCATGCCACTGATGATGTTGTTTTTCTATGCTAATGTAT

TGTGACATGGTGGTATGCTGCTGTCAGATATTTCTCATTTCATTTTGTTCCCATCCTTGAGGAGCATCATTTTTTTTTTACTTATGTTTTCCTTTCCTTT

TTCTTTTTTAAATTTATGCATGTTTCCTTTTAACCTGTAAGAATGGTATGCATATGTTTGTAATTTATTAAATGAATATAAAGTTGTACTGCTAATCACT

ATTGATCTGTAAATAGATTTATGTTATGGTATGAATGAACCTTATGAGCCTAAAGCTGTTTGATTGATTATCTGTATTATATGCTGTTTATCAACATTTT

AAGTGGTAGCTTCTTATCATCTGAACAGATAGCTTCGCTGGCAATCCGAAGTGGGAATGGGCTTCTCTTGAAAGGTGGCAAAGAAGCTAAGCGGTCAAAT

GCAATTTTGCACAAAGTTTGGTTTCAAATAAATTTATTTCTGAACTATTTCCAGTTTCAATTTTATTTTGTTTCACAGTTGCCATTAATAAAATATTTAT

GGGGATGATGCTGTATCTCTGAGATGCAGGTAATTACTGAGGCCATACCAGATACTGTTGGTGGAAAACTTATAGGACTTGTGACCTCAAGGGAAGAAAT

CCCTGAGCTACTTAAGGTGAACTAATCAGTGGAGCTTAATTTCAATTTCATAAGCCTGTTCACTTTAAGCATTGGCCACCTTTAAATACTTAAATTTCAG

CTGGATGACGTAATTGATCTGGTGATTCCAAGAGGCAGCAACAAACTTGTTTCTCAAATCAAGAGTTCCACTAAAATTCCTGTTTTAGGTCACGCTGGTA

AGACTATTATGCTTATGTTTAAATATAGGCATAAATTTTTACTCTATTTAATTTGGACTGTGTGATTTTGATTCCTCAAGTTTTTTTCAAGTCAATTAAG

TACCTTTATTTTTTAAATTAAGCAAATTTGGTCCTTCTGCTAATTTTTTTCAACCTAAGACCATAATCTTATATCCTATAAAACCCTTCAAATAATATCA

AATTTTTTAATAAATGTCGGAATTTTAAACTTTCAAAACTATCTACCTCAAATCCATCATAAATCCCTAAATATTTGGATATAAAATAATAATTTGAAGG

TTCTAGGAGAAGCCTTTTTTATGACAATGCAATTTTGTTTAATTTCAAAATTATGACACAATTGGTTTGAACAAAATTTAAGAGATCAAAATCACTCACG

TTCCAACCTAAATGGACCAAAAATGTAATTAAACCTTTAATATAATATATAATCTGTGCTTGATATTACTGTATTTCTTTTTTTGGACTAATAGTCTGCG

CTATTACTAATCCAGATGGAGTTTGCCATGTCTATGTTGATAAGTCTGCTAATGTGGAGATGGCGAGGGGGATTGTACTTGATGCAAAATTAGATTATCC

AGCAGCCTGCAATGCCATGGTAAACTAAATGTACTCAATGACTCTACAAGATTGGTAAAGCCAGCAATGTCTGATGTACATAACTTCTGTTTTTAAATTC

CAACTTTTAGGAAACACTTCTTATCCACAAGGATTTGATAGAGAAAGGTTGGCTAAATGATATTGTTGTTGACCTACGAACTGAAGGTATGATTTATAAC

CATCATTCCCTCTTATTCTGTATTTTTTTTCAACTACATAGACTGCATGTTCTTGTAACACATAACAAAAGTGAATTTTGTAAATTGAACAGATTAGGAA

AACTAACACTGGTTGAAGTTTATTAGGACTCACTAAATGATTTGGGTCTCACTTTCTATTTAATGAGTTTTACTTAATTTCATGATTTTCAATAAATGTC

AACCAAAATTAAAAATGTGTTTAAAAGAGTGTGTTAGAAAGTGTTTTGCTAGCACTACTCTTTCTTGAATTCTCTTTCGCAAGTTATGATGAGAAGATAA

ACATCAATGCAGTTTGATTTTCACAGTGCTAGTGATATCTTCCAGAGTCATTTATTACTTACAGTTATATTGCAGAGCTAGTTCTGAATCCAAGTTATTG

TGGACAAATCCTACTGTGAATATTATAAGCTTAAATATCTGCTGTTTAATGGTTTTGAGTTTGGCTACATATTATCTAACTTGTGAATATTTCTAAAATG

AGCATTTCCTGTTTAACACAAACTATCATGTTTCAAAAAGATATTAGTGACATCGAATTGTAAACATTGATTAAGACTTAATATCTAAACAGTGTTAGCT

ATTTTTATTTTTTTCGATTAAAAAATATTTAAGCAATTGAAACGATCTTTTAGTTAACTTGGAAATGAAAGGCAACTCTGAGGTTGTCAGTTGTCATTTT

ATGGACTTCAGTTTACATAATTGTCTGATTGGCATCATTTTTTTAACATAAAAACTTATGTAATGGGGAGTCGAATGAGGCACCATACCAATTTTAAAGC

ATTTTCTTTTATGGTTTTTGGAATGATTAAATTATGTAAATTTATGCAGGTGTTAAATTATATGGAGGACCAAGGGCAAGTTCTCTGTTAAATATTCCAC

AAGCACATTCATTTCATCATGAGTACAGTTCGCTGGCTTGCACTGTTGAAATTGTGGATGACGTGTATGCAGCTATTGAACATATAAATCTTTATGGAAG

GCATGTCACTGTTTTCTTCTCGATAGGAAACATGTTGAAATATATTTTGCTTTCATTTGTCTTCTTTGCTCTTCTCCATTTCAATTGACATCTATGTTAG

TGTAGTGAACTTAGGTGGAAAAGTGTCATATCTTCTCTTTTCTTTTATGGCAGTGCACACACTGATTCCATCATTGCAGAAGATAAAGAAGTTGCTAATG

TGTTTCTACGCCAAGTAGACAGGTAATATTTGAAATATCATGCTTCTTTTTATTTAATGAAAAAAAAATCAAGGGAAATAAAATAAAAAGATGTTTCACT

CAATTCTCTACTTTGTAGCGTCTCATTATTGTGTGTAAATTTAGTTGGTCTTTGACAAAGTTGCTAATTAGTAAACACTAAACAAACATTTCCTTTAAAC

TATTAACTGCTTTTCAAAAAGATATCAACACATAAGTACAATGGCATTTATATAAATATTGTTCCTCTTGTATTGGCAGTGCTGCTGTTTTTCACAATGC

AAGCACCAGATTTAGTGATGGGGCACGATTTGGACTGGGCGCAGAGGTATAATATTGGTTGCATTTTATTATCAATCTATTGCATTAAGGTTGTTGATTT

ATTATGTAATTCCCTTCTGGTTGAATAGGTTGGAATTAGTACGAGCAGGATTCATGCTCGAGGTCCAGTAGGTGTTGAGGGATTGTTAACAACAAGATGG

TATGAACTCATGGTCATTTTGCTGATATTAAATTTTCGGTCCTTCATTTGTCCGTTGTCACGGATAGTGTACACTGTACATCTTAAATATCAATAACTTG

CTGCTACACATCAAGGAAAGTAGCCAATCTATGACACTGCTTTATTTTAATGGAATTATTTCGTAAGACTGCTCTAGAGTCTACATCACAGTCCTAACCC

ACGTATGATTAATAATATAATCATAATGACATTTTATAATAAAACCCCCGAGTTGTATGTGGCATTTCCTTTGTGTTTTACTCATTGTCTTATGCCTTTT

GCAAATGCAGGATACTAAAAGGAAGTGGACAAGTAGTAGATGGTGATAAAGGGATTGTCTATACCCACAAAGACCTTGCAGCTTAATTTGAATGGTCTAA

CTGCTTTACTTCCTTTTGTAGCCTTTTTCATTTGTTTTGACAGTTGAGAACGGTATAAAATTGTACAATTTAATAAAATAGGGTCATTTGTATCATCTGA

TGGTTCCTTTCCTTCAAGAATTTTTTACGTCAACAATGACAGTTTGTGATTGAATTTGTTATTTAGCGCATCCGTTTACGAATAAAGCACCACCTCTAGG

AAAACACAAAATGTTAAGTGTCAGCAATGGAATCCATTTATTGCTTTGTTAACGACAATTGAATATTTATAG

>Glyma.18G034300 | Chr18:2647511..2653492 forward

ATAATTCCTCTAACCATAGGCTGTAATAATTAAGAATAAATAAAAGAAAATGTAAAAATATATGTGGTGATAGGATAGTGTAAAGTGTTAACCTTTTTAT

CAACACGTGACATTAAGATTAAGAAGTAAGAACTGTTCACGGCTACACTCTCTTTCTTCTCTGCCAACCACACTGAACAACAAACACAGTCTGTTTGGTC

AGAATATGGAGAACACAGATCCTTGTAGACATTTTCTCAAGGATGTTAAGCGCATCATAATCAAGGTTCTCTTCACTCTCCAAAAACTACTATCACTATG

CTGTTTCGTTTTTTTTTTTTTTTTTGTGTAACAAATGGTTGGCGTGCAATGCTTGAACCATAGGATTATGCAAATGGTTAAGGATGTTTTGTTTGGTTTT

TTAAGGTTGGGACTGCTGTGGTCACTCGCCAAGATGGAAGGTTAGCTGTTGGAAAATTAGGGGCACTCTGTGAGCAGGTTGCTTTTGTATATTTTTGTTT

TGAGTTTTTTTTTTTGTTTGTTCAAGGAATAAAAATTGGATTTTGAGTTTGGCATGTGTGACCCATTTGAAAATCAATGGCAGATTAAGGAGCTGAATTC

TCTTGGTTATGAGATTATTTTGGTGTCATCAGGTGCTGTTGGCCTTGGTCGCCAAAGGCTTAGATACAGAAAATTAATTAACAGCAGGTAATAAAGTTGT

AACACCTTTGTTTTTGTTTTGTTTTGTTTCTCTCAGCATGCACATGTGAGGTTGGTTGAAATTGTAATTTACTTGGGTCTTTCATGGGACATGTCACAGC

TTTGCTGACCTTCAGAAGCCGCAAGTTGAACTTGATGGCAAGGCTTGTGCTGCTGTTGGACAAAACAGCCTTATGGCTCTTTATGATGTTTTGTTTAGTC

AGGTAATTGTTGTTGATTGATGTTCATGACTGAACTATCATCTTATGCTTGCTCGCTCTAATTCCAAATGTCATCAAGATTTTGTTTTGTTTATCCTTAA

TGCCATTTCATTTTCCTTATAATTTGTTGCCTTATTTTAGGATAGCTGACAGTGATGTGTTAACTCCTTTCTCTACCTGTCTTGCAGCTGGATGTGACAT

CTGCTCAGCTTCTTGTGACGGACAATGATTTTAGAGACAAAGATTTCAGAATGCAGCTCAGCGAAACTATGAAATCATTGTTAGCATTAAAAGTTATTCC

CATATTCAACGAGAACGATGCAGTTAGTACTAGGAAAGCTCCATATGAGGTTTGTTATTCTACTTGTCTGGTTTGTTATCAGAGCAGAACTTAATCTTAG

AGCTGATTGCTTCAAGCTATGCACCTTTATAAAAAAAAAAACAGGTGACGTATGAGACTAGACTAGAGAATACTTAAAGTGTCACTAAATCATTTTATGC

AGGATTCTTCTGGCATATTTTGGGATAATGACAGTTTATCAGCTTTATTGGCATTGGAATTAAAAGCTGATCTTCTTATTTTGTTGAGTGATGTAGAGGG

TCTCTATAGTGGCCCTCCAAGTGATCCACGTTCAAAACTTATTCATACATACATTAAGGAAAAGCATCAAAGTGAAATTACTTTTGGAGACAAGTCTAGA

GTGGGAAGAGGTGGAATGACTGCTAAAGTAAAGGCTTCTATTCATGCAGCTGAAGCTGGCATTCCTGTTATCATTACTAGGTGCTCTATTATACGATGTT

ACCTATAAAAATTAAAACTGAAACTTGGTTTTTCATTTTTCAAAAGCACTGCTATTGACAGCATATTTGTATCCCTTCTTTAGGTTTTATTTAAATCATT

TTTAAGTGGCTCTGGTTTCAATTAGAAATATTTTATTTAGGACATGACTTGTGAAATCACTAATCACAACTTTCACTAAACCACAGGCTATTTCGAGAAA

TGGAAATAAAAAGGCTCTTATCTATTTAATATTGTATACAGTTATCTCTTGATTTGTTCTATTTGTTTTTCTCTTTGGATGACTTTTTGTTGACCACATG

TTTAGTCATTAAATTTAAATGAGACATGCGTGCATTGCATGCGCAAGGATGGGAAAGATCACTCTTGAAAATTTCCCCTTTCAATTTTTGATATATTAAA

ACAGAAATTTTAATTCCAAGAACACTATGAAATCCATTATTTATGCTACCCACTTTATCTGATTTTCATCTTGCAAATTGGTTTTTCCTTGGTTCTGCCA

CATTTTAGTGGTTATGCAGCTGAAAATATCATTAAAGTTCTCCAAGGACAACGGATTGGAACCCTCTTCCATAAAGATGCACATAAATGGGCCCCGGTAA

AAGAGGTTGATGCTCGTGAGATGGCAGTTGCAGCTAGGGACTGTTCCAGACGGCTTCAGGTCTTCCTTATGTCTTAACACTAAACAAATACTACTGAGCT

GCCAGTATACATTCCTACTTTAGCCTTCTGATCTCCTTATTCTCCATTACAGGCCTTATCTTCAGAAGAGAGGAAACAAATTTTGCTAAAAATAGCTGAT

GCCCTGGAAGCACATCAAAATGAGATCAGGATTGAAAATGAAGCTGATGTTGCTGATGCAAAAGAAGCAGGATATGAAAAATCCTTGGTTGCTAGACTGG

TTTTAAAGAATGAGAAGGCAAGAAAGTTCACCTAAAGAAATTGTTTAAATTTTACCTAACTGTACATAATGATTTAAATTTCAACAAACTGTAAAATTTG

TGATAATAAATCATGATAACTGACAGCTTGCAAGCCTTGCTAACAACATTCGAATTATTGCAAACATGGAAGATCCAATTGGTCGAGTGTTAAAACGAAC

TGAGGTAATAAAAACTTTGCTATAGCTATATATTACAGGTCATGGTAGTTTTACATTATGCTAAGTTGAATTGTTTTCCATTTTATTTATTGTATTTATT

ACATATTCTAGTTTTACATTACGCCTGTCAAAAACAACGAAATGAGTTGTGTTATTTAAATATTGTATAAGTTGTCAGAACTCCCCAGTATGGGACTGCA

ATTGTTGCTTAGTCATTGTTCATATAATACATAGTTTATGATGTTTTGAAGAAGCTTTTCAAGGACATTTGATTGTACTGAATTTTTAATTTTTAATAAT

AGTTATTGATCTATAATATTTCTGACAGCTACCTTTTTATTTAGCTTGCAGAGGGGCTGATTTTGGAGAAAACATCATCTTCTTTAGGAGTTCTTTTAAT

TGTTTTTGAGTCTCGCCCTGATGCACTAGTACAGGTAAATTAACATTTTCATTTTTCTTTTTCACCTTCTGTTAATACCTGGTTAATTATTCTCAATTTC

TCGCTTTTACCTTTCCTCTTGCCTTTATTTATGATTCTTTTTCAATTTGCTGTCTAGATGACATGCTTGGACAACTTATATTACCTACTATGCCGTGGAT

CAGGCCTTACTTTAAAATAATAATATGCATTCTTCTCATATCACATAAAAAGATTAATCTTTTTTTTTAGATTAAAAAAGATGTCTAAGTTGCAGTTCAG

AAAACTAAAAGTCTAGATTGAATATCTTTTAATTTTATGTTTATCTGCATCCTAAGCCAGTAGCTTCATGTAATCTGAACAGATAGCTTCTTTGGCAATC

CGAAGTGGGAATGGGCTTCTCTTGAAAGGAGGCAAGGAGGCTAAGCGATCAAATGCAATTTTGCACAAAGTATAGTTTCAAATTAAATGATAATTTGTGA

ACCTTTTTCTTTTTAAATTAGATTTGATTTTTGCAGGCTCATTAATAAACATTTTTCTGGGCAATTTGTCATTGCTGGAATATCAGGTAATTACTGAAGC

CATACCAGATATTGTTGGCTCAAAACTTATTGGACTTGTGACATCAAGAGCAGAAATTCCTGAGCTACTTAAGGTGAATACAAAGTAGATATTCATTTAT

GATTTATAGTCCTGCTCACTTTGAACATTGGTACCTTTAACAAAAGCTAGAGTTTGCATTCTACTTTTCTTTCAGTTGGATGATGTAATTGATCTAGTGA

TTCCAAGAGGCAGCAACAAACTTGTCACTCAGATCAAGAGTTCCACTAAAATTCCTGTCCTGGGTCATGCAGGTAAGACCTTTCTGCTTAACATGCAAGA

AATGCTTAATGTGATATGGTAAATCCTTTTGTCAAAAGAATTTGCCATAACAAGGCCACATTATTGTCCAACCAGATGGAATTTGTCATGTCTATGTTGA

TAAATCTGCTGATCTGGAGATGGCAAGGCGGATTGTACTAGATGCAAAAATAGACTATCCAGCAGGCTGCAATGCCATGGTAATTTCTATGTACTGGATG

GCAGTGCTTTGTTTGCTGGCTATTTTTTCTTACTCTTCAAATTTCAATTTTTAGGAAACTCTTCTTGTTCACAAGGACTTGGTAGAGAAAGGTTGGCTCA

ATAGTATTATTATTGACCTACGGACAGAAGGTATGACTTCTGGCTAACATTTCTATTAGACTGGTGAATTATCTGACTGACATCTTTTCCCTGATTGAAG

AATGGTTTCTTGAAGTCAAATACACTGTTAAGTGTAGTTTGTAAGGTTTCTGGAATGCTGAAATGGTTTAAAATTTATGCAGGTGTTACATTGTATGGAG

GACCCAAGGCAAGTCCTTTGTTAAATATTCCAATGGCTCGTATGTTACATCATGAGTACAATTCGTTGGCATGCACAGTTGAAATTGTGGATGATGTTTA

TGCAGCTATTGATCATATAAATCTTTATGGAAGGCATGCCACTCTTTATTTCTTGATAATGTGTTGGAAGCATATTTTCCTTTCATCCATCTAATTAGAT

CTGTAGCCGTGCTGTAGGCTTAGATTGAAATTTTTCACATTTTCTCCTTTGTGGCAGTGCACATACTGATTCCGTTGTTGCAGAAGATCATGAAGTAGCC

AATGTGTTTCTACGCCAAGTAGACAGGTAATATTTGAAATATAATGTCTTATTATCTTGTGTTACTAATCACATTATCTACTTTGCAGCATTTCATTATT

GTGTAATTTAATTGCCTTTTATGACTGAGTTATGTATTTAATAAACACAACAACCCTTTTTCTTGTAAGCAATCAACCCTTTTTGAGATATCAGCACATT

GTACAATGATAGCTATGCAAATAATGTTTGCTGTATTGGCAGTGCTGCTGTTTTCCACAATGCTAGTACAAGATTCAGTGATGGGGCACGATTTGGACTA

GGTGCAGAGGTATGATATTCTTTACATTCTTTTAATAATCATTCTCTTATGCTAAAGTCATTGATTTAATTCATAATTCTCATGTCATTGGCTTTTATAG

GTTGGAATAAGTACAAGCAGGATTCATGCTCGGGGTCCAGTAGGAGTTGATGGATTGTTAACAACCAGATGGTATGAGCTTGATGGTCATTAATTCATTA

TAATACTTTAGAAACAGTACAAACATTTTAGTTTCCTGTACAACTAGCCATTTCATTCTGTCTCTTATTTAGTTGACATGATCTATTAGAATCACACAAG

GTACAAAAGATATGTGAATAGTTATATCATATACAAACTAACATACCAATAGGCTTATAAAAGGATAATAACTGACACTACAACCATTCATTTCCTTGTG

TGCAGGATTCTCAAAGGAAGTGGACAAATAGTGGATGGTGACAAAGCAGTTAACTACACTCACAGAGACCTTTCTATATGATTCATGATTGTTGCAAGTA

AAAATTCATGGATTGGAGGGATGGGAGAAAAGGAGGGTTGCTAGGCTGTGCTTTATTTTCTCTAGAGTGGGGCTAAAGTTGTACAATTCAGTGGAGGAGG

GATATTTATGCTATCTGGTGCTGCTGCTTTTACGTTCAAGATGTTATACAAGGGAATTGCTATTCTCCCCAACGTTAGAAAATTGATGTTCATTGTGTGA

GTTTTTTTTTTGCTATAAATTTAAACGTCAGTATAAATATTTATTATAAAATCAGTATGAATGTTTTTTCTCCCCAACGTTA

>Glyma.07G240300 | Chr07:42101307..42113123 reverse

ATCGAGAGAGACAACAAGATTTGTGACTTGTGAGGGACTCAGCGGAGACTTGCTGTCTATTATATAAGCCCATCTACTAAAAACATTCAAAGATATTACT

ACCATTTCTTTTCCTTTCCATTTTTTTTTTGTGTGGATTTTAATCCCTCCTTTCTTTCACAATAAAAAAGAGAAAGAAGGTATAGAGAGAACATAGGAGT

CCCAGTAGAGAAGTAAAGTGCCATAAAAAACGACAAGACGCATCACGCTACGAATGGAATCTGTGTTTCCTTTTTCCTCATTTTCGTGTCACGGAGCATG

AATTTTCCGTTTCGTTTTTACCTTGCTGACTAAGATTGTTATTGTTAATATCAATGCCATCCTCATCAACACGAAGCTTAAGCTTACGCGTTCACTGAGT

GGGAGCATTTTCCTATAATACACTCCGCAGAAGCTTCGAGTTAAAGAGCATCTATCCAAATCGAAGCCCTAGCTGGTTTTCTTCCGATAGAAAGAGACGC

GCTCTGAATCTGCGCATTTCCAATGGCGTTTTGGTGGCCGTTGCTCGTTTTAGCATTCGCTTACGGTATTTGTAGGTTCCTTCTCATGCTCATTCCTCCT

AAAGTTCCTTCCATCGACGTTGACACTTCCGATGGTACTCTATCTCTCTCTACATATCTATCTATATATTGTACACACACTCAAGGAGGTTTGTCGTGAC

GGTGAAATTGTGATAATGACAGTGTTGGACGATGGAAACCAAGCGCAGGAGAACAGTTTCATATATGTGAGTTCTCTCTTTCTCTGCTTTGATTTACGAA

TCGCATATGAAAGAATCTTGCAAATCGTTGCATGATATGGTACTGTTGTGCGTTTAATTGCATTTTTTCTCAATCGTTATGATTTGTCATTGGTTCCAAT

CCAGGTACCTCCAAGGGGAACATCGCAGCAATCGGGCAAGATAGTTCAGTGCTACGAACCTGCAACTATGAAATATTTGGGATATGTTCCTGCATTGACG

CATGAGGAGGTCAGTGAGCTTCAGCCTGTGCTCTTGTAACAGTGGACTAATGGTTATGATGAGTAGTATTTGTATCTATTTGACCGTAGTTTATGATTGA

TGGTTTGATTAGGAACTATGAAGCACCAATACTTTAATTTGCTTCAAGTATCCGTATCGTATATGTATCCTGTATCGATACGTACATGTGAAGTATCCAA

ACCTTTAACAAAAGTTTATGAAAATCCTATACAACCACATATGAGACATAGGAATAAGATTGGAAATTATGGATGAGTTATCCTCTTTATGGATGATTTC

AAGAAAATAATAAGACCGATACTATTCCTATTTATAGACGGTAGGAATTGAATAATAATAATAATGATTTACAATTTTTATTTTGTTTTTAGTAATGTCC

TACAAATATGTTCTTAATTTGGATTTGCATAATTGCATTATATATTTATTATGTTTTATGAATTTTTATATATATCTAGATATGTATTGTGTTATCAGAT

ATGTATCGTATTGAATATACATATCGTATCTGTATATCATAGGCTAGGAAGCACAATTAGTTTTCAATTAAACTTATTTTGTGATTAGTGTTTGATGAGA

TGCATTCATGTGTGTTTGGATACCCAGTTTGCAACCAACGTAATGTCAATCCACCCTTAAGGTTGCTTCTACATTTGGTGCATTCAAATGTACAAGCTTC

CATTTAAAATGGCACAAACTGGTTTCAAAACATTAGAAATCTACAAGGACATCATTGCCATGCAAGAAATATGCTGGTTGTGCTGAATTAAATCTGACAT

GTCATAGCTTCTACCTTCTGGAAAAAATATTATACTAACTAGACCAGTGCCCAGTGTGCATGTGAAATAAAGGCAACACAATCCAAATAGAATATCACTT

TTACAATAATATTACCAGAATAACATCATTTAAACGGGAAGCGGAATTGTCAATGATTGAAATGTAGAACATTGTTGGACAGTTTTAATATAATTATTGA

TTATCCATTTTGTTGTCTATGTTGAAGTATAAGTTCTATGGAATTCCTTTGATTTCTATGTTTTTTGTTCTCACAATTTTTCTTGATGTTGACCATATTT

AGTTTTCATCCTTTTTGTTTATGAATGTCTGCACTTATTTTTAAAAATGAATTAATGTATAAGTATAGTACCTCTAGTACTGATTTATATATAATACTAC

ATTATTTTTGCTGTGCAAAAAAAAAAAATTAATGTATAAGTATAATACATCCCATTAGCATTCTTATTGATCTTTATACATCTGGAAACATTAATGACAA

AGCATCCAGACATACTAAGTGGAGAATATTGATGTAAAGTACCAGTTGTTGCAGAGCATGCATGATGAAATACTGGTTCCTAGTTTTTGTGTAAATTTTT

TACAAGCTTTGCTAGATTAACTATAACTGCTTTGTGTGTATGGATAGGTCAAAGATCGAGTGTCAAAAGTAAGGAAGGCACAAAAAATGTGGGCAAAGTC

CAGCTTCAAGCAAAGACGCTTATTTTTGCGTATACTTTTAAAGTATATAATTAAACATCAAGCGCTTATATGCGAGTAAGAACACTTTTCCATGGTTGTA

GAAGTTTATCATATTTTTTTACACTTCTTGGAAACGTTTGTGATACAGACTTGAACTTATAGTCACTTTTTTGACATTTTAGGAAATATGCAAATCTTTC

ATAGCTACCCACTATTTTTAGTTATCCTACCGTTTAATCCATCTGCTCTGAAGTAGATGTAAATTGTGATCATTGCATGATGAGAATGATGATAGCATCT

TGTAATTCATGTGCTTTAATTAGAATATCTTCACGTGATACTGGAAAGACAATGGTAGATGCCTCTTTAGGAGAAATAATGACAACATGTGAGAAGATCA

ATTGGCTATTGTCAGAGGGTGAGCAGTGGTTAAAGCCTGAATACCGGTACCTTCTTTTCCCAGTAGTTGACTATTTTATTTTCTTAAACTGATTATGATG

AATAATCTGTCTCATTTTATTTTCTGTTCTTTCCATACCTTCTTTTACACAAAAACTAGGCCTTATAACTTATAAGATACCTATTGTTTTATTAAAAAAA

AATAGTGTTATAACTTCTTCCTTGTACATGCATATAATATCACTATGTTGAAATTTCCAATTATCTAAAGTCACTTCTATGTTTACTTTATTTTTCCTTG

TGTGTGATGAATAAAAAATCTTTGTAGGTCTTCTGGAAGATCAATGCTTCATAAGAGAGCTAAAGTAGAATTTCATCCCCTTGGTGTTATTGGAGCCATT

GTTTCATGGAATTATCCCTTCCACAATATTTTTAATCCTATGCTGGCTGCAATTTTTTCTGGAAACGGTATTGTGATTAAGGTATAAAATATCTCACCAT

CTACCTATCACAATTGATCTTCATGCTGTGGGTGTATTGTGTTGATTTTAGGCATTGCTTACATTTGTGCTTAATGTCAATTAGGTTATATTCCTTGGTA

AAATTAAAAAATAAAAATCTCTTGCATGATGCAATACAAGTCCAGATCCTCTCCATTTGTTTGAGAAATGGACATCTCCATTTCTATGAAGGAAAAGTAG

ACACGTGGCATAAAAGAAAATCAAATGGCTGAGATTTAATTTGGTTCAATGCATTTTAAATTAAAAAAATTGAAAGCCAATGAGTGACAAAAGCAAGGCA

CGTGTTGTTTCATTGTGGGTTTTGGACGGCCTCAACTGGCAGAAAAGATTGAGTGCAACGACCCATTAGAGGCGGCACAGCTCCATGGTGGGTGTTATTC

TAAGGAAGATCCTTAGAGGCACCTCAGAATTCATTTCTTGGTTCATAATTTCCTGAACTATTGCCATTGAAAACTCATCAAAATGGACACTGGGTAAAGT

TTTGTTGGGTTGTGCTGGTAGATTGTTCTTCACTTATTCACCTCCTCGATCAATCTGAGTTTTGAAAACAACTGGACCAATTAATATAACACAGTCTTAG

TGTTCAACATTAAATATTTTTTTTTTATCTTGTGTTGTACAGTTTTTGAATCCTGCCTAGGTTAGCTTTGCTATTCTTAGCCGGTCCCAAGCCCGGATAA

AAGGAGAGGGTTGTGTTAGGCTTTCGACAGCCAACGTAAAACTTTGTCGAATCTCTATGACATGGATCAATTACGTAATAATGTGAATGCTAGGTCGTTG

CCCGGAAGCAACGCGCTGTATGGCTCGAGTACAGTGTCAAAAGAGCAAGGGCCGCTGCATCGCCGCCCGGATGTAGTGAAAAGTAAGCAAGGGTTCCTAC

ATTTTCGTGAATGGGTGTGGGTAAAGAAGCTAGTTCATGACAGGAGGATTCGCTTTGGTACATGGAATATAAGCACACTTACTGGAAAATCTATGGAAAT

AGTGGATGTTATGGTGAGGAGGAAGATCAATTTTATGTGCCTACAAGAAACTAAGTGGACAGGTGAAAAAGCGAAAGAATTAGACAACTCGGGATTTAAG

CTGTGGTATACGGGAAAAATCAGATCAAGAAATGGGGTAGGGATTATTGTGGACAAGGAGTGGAAGAAGGATGTCGTAGATGTAAGAAGAGTAGGAGATC

GTATCATAGCCTTAAAATTGGTAGTGGGACAGGACACCTTTAATGTTATTAGTGGGTACGCACCTCAGGTTGGGTTAGCAGAACACTTTGAGGTAAAATT

TTGGGAGGATCTAGAAGGGGTACTTCAGGATATACCCCAAGGAGAGAAAGTTTTCCTAGGAGGGGATCTCAATGGACATGTAGGTAGCGTGGATAGAGGT

TTTGAGGGGGTGCATGGGGGTTTTGGCCTAGGGGAGATGAATGGGGAGGGTAAATCCATCTTGGAGTTTTCGGAGGCTTTGGATCTTTCTATAGCCAATA

CATGGTTTCAGAAAAGAGAGGAACATCTTATCACTTACAAAAGTGGAGGGACATGTTCTCAGATAGATTTCTTCCTTATCAGGAAGTCTGATAGGAAGTA

TTGCTTGAACTGTAAAGTTATCCCGGGAGAGAGCTTGACTACCCAACATAGAGTTTTGGTTATGGATGTAAGAATTAGAGATAGGGCAAAGAGAAGAAGT

CCTATGGTAGCACCAAGGATCAAATGGTGGCACTTGAAGGGTGAGAAACAAGGAATCTTCCAACAAAAGATATGGGAGGGATGGTGTGGAACAAGACGTC

CCAAGAGATTATTAAAGTGGCTAAAGAGACGTTGGGTGAATCTAGAGGTTTTGGACCTAGGGGTAAAGAATCGTGGTGGTGGAATGAAAGTGTTCAGAGC

AAAGTTAGAGTAAAAAAGGAGTATTTCAAGGAGTGGTCTAGGTGTAGAAATTCTGAAACTTGGGATAAGTATAAGATAGCTAGAAATGAAACCAAAAAGG

CGGTGAGTGAGGCAAGAGCCCAAGCTTTTGACGGACTATACCAAGCTCTAGGAACCAGGGACGGAGAAAGATCTATATATAGGCTTGTTAAGGGTAGAGA

GAGGAAGACTAGAGATTTGGATCACGTAAAGTGTGTTAAGGATGAAGAAGGCAAAGTCTTAGTGCATGAAAAAGATATCAAGGAAAGGTGGAAGGTGTAT

TTCCACAACTTATTTAATGATGGATATGGATACGACTCTAGCAGTCTAGACACAAGAGAAGAGGACCGGAACTATAAGTATTATCGTCGGATTCAGAAAC

AGGAAGTAAAGGAAGCGTTGAAAAGAATGAGTAACAGTAAGGCGGTGGGGCCAGACAACATACCTATTGAAGTGTGGAAAACTCTTGGAAATAGAGGTCT

TGAGTGGCTCACCAAACTCTTTAATGAAATTATGAGATCAAAACGCATGCCGGAGGAATGGAGGAGAAGCACGTTAGTGCCAATCTATAAGAACAAGAGG

ATATATAAAATTGTGCAAATTATAGGGGAATCAAGCTCATGAGTCATACCATGAAATTATGGGAAAGAGTGATCGAACGGAGATTAAGAAAGGAGACTCA

AGTTACTGAGAATCAATTTGGTTTCATGCCAGGAAGGTCGACCATGGAAGCGATTTATTTATTACGGCGGGTGATGGAGCAATATCGCATGGACCAACAA

GACTTGCACTTGATTTTTATTGACTTGGAGAAAGCGTATGATAGAGTGCCTAGAGAGATTTTGTGGAAAGCTCTAGAGAAGAAAGGGGTTAGGGTTGCAT

ATATTCGAGCTATCCAAGATATGTATGATAGGGTATCGACTAGTGTTAGGACACAGGGTGGAGAGTCAGACGATTTTCCCATCACAATTGGTTTGCATCA

AGGGTCAACCCTTAGCCCCTACTTTTTTACCTTAATTCTGGATGTCCTCACGGAACAAATCCAAGAGATAGCGCCGAGATGCATGCTTTTTACAGATGAC

ATAGTCCTCCTTGGAGAGTCGAGGGAGGAGTTGAATGAGAGGTTGGAAACTTGGAGACGAGCTCTAGAAACACATGGCTTTTGCCTAAGCAGAAGCAAAT

CGGAGTATATGGAATGTAAGTTCAACAAAAGTAGGAGGGTATCTAACTCAGAGGTGAAAATAGGAGACCATATTATCCTTCAAGTCACACGGTTTAAATA

TCTTGGGTCTGTAATACAGGATGATGGAGAAATTGAAAGGGATGTGAATCATCGCATTCAAGCAGGATGGATGAAATGGAGAAAAGCATTGGGGGTGTTA

TGTGATGCAAAGGTACCGATCAAGCTAAAGGGAAAGTTTTATCGGACTGCGGTAAGATCGGCGATTTTGTACGGAACAGAATGTTGGGCGGTCAAGAGCC

AACATGAGACTAAAGTAGGTGTAGCGGAGATGAGGATGTTGCGGTGGATGTGTGGTAAGACTCGACAGGATAAAATTAGAAACTGAACTATTAAAGAGAG

GGTTGGAGTAGCGCCTATTGTAGAGAAGATGGTGGAAAATAGACTCAGGTGGTTTGGGCATGTAGAGAGAAGACCGGTAGACTCTGTAGTGAGGAGAGTA

GACCAGATGGAGAGAAGGCAAACAATTCGAGGCAGAGGAAGACCCAAAAAGACTATAAGAGAGGTTATCAAGAAGGATCTCGAACTTAATGATTTGGATA

GAAGTATGATACTTGATAGAACATTATGGCGGAAGTTGATCCATGTAGCCGACCCCACCTAGTGGGATAAGGCGTTGTTGTTGTTGTTGTTGTTGTTGTT

GTTGTACAGTTTTTGCTACACCTCAATATTGTTTACTACTTCAGGATTTTTCTTCCAAATTCATTGTAATAGGAAAGGGGGGAAATGAGCATTTTCTCAC

TTTCTAACTTTTCCCAGTACTGTCGTCGGATCGTCAAAATTCTGACGGGATGCCGGCGAGTTTGATCTGGGCAAATGGCAATGCTCCATTGCAAACAAAC

CACCCTGCCTCGAAAATTCCCCTTCGTCCCCGTAAGACTAGTGGTCAGAAACATAAGTTTATTGTTGCTTGTTCAATGTCATGCAAAGGGCGAAGGCAAA

GTTGAGATCGTAAATCATAGTGCAGACACGGAGAAACTCCCTATGCCACATGTATGACGGAAATGAAGTGAAATGGAAATGTCCATTTCTTAAGGAAATG

GAGAGGAACTGAACGCGTGCAATGCATACTCTTACTTTTTCCACCTAATAACCATTTAGAAGATTGTTATTTTTTGTCCACTAATGCAAGCCTCTAGGAC

CAGGTTTGATCCTTTCTTTCCACCCATTTCTTCATTATTAAGCTGAATTTCAGCACAAGTTAAAATTAGCCTGTGCACTATCAATGCGTTAAGCAACAAA

AAGATGCTATTGCTCAAGGATCATGTTAGAATTGGAGGCATAAAACCATTTTTGAGCTTCCACCTACCAGCTTAAGCTTTTAGGAGAATTATTATTTAAC

AGTATTAAATGCTTCTATGTTTTCCAATTTGAAAGAAAACCTTGTGCTTTTTATTGTAGATATCAGAACATGCAAGTTGGTCTGGATGCTTTTATTTCCG

GATCATCCAATCAGCCCTTGCTGCCATAGGAGCTCCAGAGGACCTCGTTGAGGTGATAACAGGGTACGGCCATTTGTCTTACCATTCCTTTTTCCTTTTT

GTTCTATGGTTGTGTTTATCATCTATGGCATTGATTGAGCCTTCTTCCAGGTTTGCTGAAACAGGAGAAGCATTGGTATCTTCTGTTGATAAAGTCATAT

TTGTTGGATCGCCTGGTGTTGGTAAGATGGTATGATTCTTCTAGCCAATTGAATGAGTTCACTTTGCTCTCACCTGGAATTGTATCATAATTCATTACTT

CCAATCATTGTCATTACTTCATTCATTTTTATGATGGCATCCTATTTCCCATGAATTGCGCCATGCTAATATGCTTCATCTTTTTATGCAGATAATGAAT

AATGCTTCTAACACACTTATACCAGTGACACTGGAGCTTGGTGGCAAAGATGCATTTATTGTTTGTGAAGACGTAGATCTGGACCATGTATGAATTCTTT

CTCCCTGTTCAGATTCCCCAAAGCTTGGATGTAACTATTATCCATAAAAGCTTAATGAGATCTCCATTCTTGAGTACCTTAGTTTTAACAGCCTTTGACC

TGCTGAATATAGGTTGCACAAATTGCTGTCAGGGCTGTGCTTCAGTCAAGTGGACAGAACTGTGCTGGGGCAGAGCGATTTTATGTCCATAGGGAAATCT

ATTCTTCTTTTGTTAGCAAAGTTACCAAAATTGTGAAGTCTGTTACAGCTGTAAGTGTTTTTAACAACATTTCACTAAGTTTAACTCTTTTGTAACTTTC

ATAATAGGCTTCTGATGCATAGAATCACGTATGCTTACACTTCATTGCCAGTTTGCCACAAGATTGCTTTTTCAGAAATCTATCATATTGTTTATACATC

TGTTTTAGCTCTAGGTCAAAAACTATGAGAAATAGACATCTATTTGATTCAGGATACCTTGCATTTTCGTCTATTTTAGTTGAACCTATGCATTTTTTTG

GTTTCTCTGATTTCTCCCTTTCAGTAACAGTGTGCCCCCAAGGAACCATTTTCTACATAAATCTTTGAAGAAAAATATTAGCATCAACTTATTACTGGCT

TTATATTCTAGTGTGTGTGTGTTCTATGTATTTGGAACTCTTCAAAATGAATAGTATCTGTGAATTGTTGTTGATGTCTAGCAAATAAATAGAATTTGGT

GATGAACAAATTGCATGGCATCACTGACATGCTCCAATTCTCTTCTGAGATGATATTTTCCTTTAAAACAAAAAGTGCTGAAATCTGGAATTCATGATTA

TACTGTTTGAGAGGAGGGACAAAAGGATCAGAAATTTATGTTCATTCACAGATGGCATTAACATTGAGGACTACTATAAACATGAACTCATTGCTTACTG

ATTATTGAAGAATGGACTTCTGTCCTAGGAAATATTGAGTATAAAGATGTCAATTTTTTATTTAATTTATTGGTTATTGATTTCTTGCAGGGCCCACCAC

TTGTTGGAAAGTATGATATGGGAGCTTTATGCATGCATGAGCATTCTGAAAAGCTTGAAGGCCTTGTAAATGATGCTTTAGACAAAGGAGCTGAAATTGT

TGCCCGTGGGAATCTTGGACATATAGGCGAAGACGCAGTTGATCAATATTTCCCCCCTACTGTGATTGTGAATGTGAACCACACAATGAGATTGATGCAA

GAAGAGGTAACGAAATCACTATCTTTTCTACTTTAAGATGCCTATTTTTTTTAATTATTATATTAGAAATATCCTACACACACTCATACATCACTTCACA

TTGCCATAGTTTTTATCATCTAATTACAGTTAGTTATTCCACGTTTTTTGTGCAATTAAACTTTGATTTTGCCGTATAAAAACTATATTAGTGAGTCAAT

GATGATGTGAGCCATGATGTGCGAGAAGTGTTACTACTATTGTTGTATTTAAGAACTAGTTTCACTGTTAGTAGGTTCGCATACATTTCATACCTAGACT

ACCATTTGAGATTTTTTTAATTTAGGTAATCATATCCCATGACAGTCATGCACCTCAATTGTGCATTTACATTCAAGAATTTCCCTTCTATATTCTATGC

GTCAGCAACTGCTTACTAAATTCATCTGAGCAATTAAATGCTTGACTAGGCATTTGGACCAATCATGCCAATAATGAAATTCAGCTCTGATGAGGAGGTT

GTCAGGCTTGCAAATGACTCAAAATATGGGCTCGGCTGTGCTGTTTTCTCAGGCAATCAGAGCCGTGCCAGAGAGATAGCTTCACAGATACATGCTGGGG

TAGCTGCAGTTAATGATTTTGCATCAACATACATGTGTCAGGTAAATTTATTAACTCTCTTGAAAGGGGATTCACTTAAAAAGATTTAATGGTAATGTAT

CTTGTCATTCTTGTATCTAACTGCTTGTTTAGTATGTGTTGATCTGTTGTGAATGTGCAACAAGTAGGCTGACTGCAGAATTTCCCTGATCAACAGAATT

TCCCAAAACTTCTATCTAGACTCTGCTTCTCTGCTGGGAAGTATTTGGTTTGCAAAGATGCATGATTGCATAAACATAATGAGATGTCTGGTATTAAAAA

TGTAAAGACTGATAAAATAATAAATTACTAATGATTAAAATTTCTGGTGGGTAGTTTGCTAAAACTATCGAGAAGTAATTTATCAGAGACCCAAAATTGG

AAGCGGGTAACCTGTTTAGCCCAGTACTTGTGCAGTTATTTTTTTCTTTTCAAACTGCCTGGAAAAGTTAACTCGGCCTTGGTTATCCTCCAATTTTCAC

TGAAGTAATTATGCATTATGATTTTGCAGTCCCTACCATTTGGGGGTGTGAAACATAGTGGATTTGGACGATTTGGTGGTGTAGAAGGTTTGCGAGCTTG

CTGTCTTGTTAAAGCAGTTGTTGAAGATCGATGGTGGCCATTTGTCAAAACCAAGATACCTAAGCCTATTCAGGTCAGTCAGGAAAACTTTTTGTCATAT

TTCCTTCCAATATCTTTAGATGTTTAAGGTTAATGCACATTAATTAAGAACTATTTTATAGATGCAAATTTGGACTAAAAAGCCGTTATTTAACATCCCA

TCAATAGTAATAAATTATATTACAAATTCCTAATTATCTCAACTGTAATAGCTATTAAATAAGGGTATAGTCGAGAAAAACAGATTAGATTTTGAAATTC

TAAAACATGAATTGTTTTGAGATAAAAGAAAGAAAGCTAAGACATGTAAAGATATGGAAAAATGTAGTATTTTTTTACTTCACCGTGAATAGAAACTTCA

ATGGGCTTCTTGTCTGCTACAGTATCCTGTTGCAGAGAATGGATTCGAATTTCAGGAGTCACTTGTTGAAGCGCTTTATGGTCTCGGCATATGGGACCGT

TTGCGAGCATTAGTAAACGTTTTAAAAATGCTTACTGAGCAAAATCCTGGTGGCAGTAGCAATAAGAGAAGAAATGACTGATTGTCAGGTCAGGTAACAT

TCTAAATGGCTAATATCGTCAGATACTCAAACCCTGCTGCTAGCAAGCTGTAAGAGAAGAAATGAATAATTGGCGGCAACTCAGGATAACACGCTAAATG

GTTAATATTGCCAAGTTAGGTGGGGGATGTCACTTTTGGTTGACGTATTTATCTAATCTCTATCTAGAAATCTCCTGTGACTATAATGTTAAGGGGATTT

AAACTCAAACTAGTTGGTGATTATATAAATTGCAGGAGATAGGATATTTTTGTGATTGTGGCCTTTGGATACCTATTCATTAGGGCATATACCAATTATC

AACTTAAGGCTATGGGT

>Glyma.09G036000 | Chr09:3014591..3025170 reverse

CAAGTACAACACTAACAACAGTATCAAACGCACAGTTTCTTCTTTCATCGCCTGAAGGTTCCCAGATTCTTTCACTGTCTTCACTCTTCAACCATTGTCC

TCAAAAGAACAATCGAAACTCCGCGGAAGCTTCTGGAAACCCTACCGAGGCAATTTTCTCGCTCACTCTGAATCTGCTGCGAACTTCTTCCAATGGCGTT

TTGGTGGCCATTGCTCGTTCTGGCACTCGCTTTCGCCATCTGCAAGTTCCTTCTCATTCTCATTCCTCCCAAGGTTCCTTCCATCGACGTCGACGCTTCG

GATGGTACTCTGCCTCACTCAATCTCTATCGCTCGTAAATTTGTTGCCACGCTGAAAATGTGATGTAGATTTTTTACTGTCACAGTGTTGGACGATGGCA

GCCAGGCGCAGGAGAACAGCTTCATATACGTGAGTGAGTGCTATGCACCTGCAATTTTGAATTTTATAAATCGCTGTGTAGTGATTAGTGACTTATTTTT

TTATCGGTTTTATGCTTTTGTGTTTTGCTTGATCGGTGTGATTTGTTTTTATATGTGGAATTTAAAATTCAAATACGGATTAATTGCAAATGCATGTGGT

TCGGAGTTGTTGTAGTTTAGGGGTTAACTTGTTCACGACATGGCACTTTGAATGCAATGATTTGGAGTTGGAGGTTTGTGATTTTTTTTTTCCTGTTCTG

GAGAGTATGGCTGATTCGGTCTCATTGATGATTGATTCGTAATCAGGTGCCTCCAAGGGGAACGGCGCAGCAATCGAGCGGGAAAGTTCAGTGCTATGAG

CCTGCTACCATGAAATATTTGGGATATGTTCCTGCATTGACGCCGGATGAGGTCTGTGAGCCTGTGCTTCTAAGGAAACTGATCGTTTGATGCATGCTTT

TAGTATCTAGTTTGAGCAACATTTGTTTTAAGATGCAATTTTTTTTTCTCTTGGTAATTAGTGTTTGGGCAACATCCCTCCCCCATACCTTCGCATAGCG

AAGAGCCTCTGGGCAATGGGGTACGAAGTTTTTTTAATTAGTGTTTGGAAATTTTGATATTTAAGGATACGATACTTGAAAATTTTTGTGGCTGAAGAAA

AATACCATAGTGTTTATAAGGTAGCATTGGTTGTCAGAAAATATGCTAGTTGTGCTGAAGTTGACATGTCATTAGCTTCTAGCTAAACTTTTATGTTAAA

GCTTCATTTTTGCATTTTACATACTTGTCTATGTTGAAATGCAAATTCAATGGAATTTTATTCAAGCTGTATGTTTTTTGATGTTAAATATGGAAATTCC

TTTTTTCCAATGTTGACTACCTTTTGGTTTTCATCCATTTATTATTTTTCGTCGTATGAATGTGTATTATGCCCGTGCCTAGCTTAATTAAAGTCCTCCT

GGCACTCTTAATGATCCATGTGGGGGTCCAAGTGACAGTTACGGGTGGAAGTAAGCCGAGTCGAGCAAAACCTAGCACAAACTCGGTGCAATGAATTCCT

TTTCTTGGTTCATTCCTTGCTCATTGGCTGCTTCAGTTTGATCCTTTACTTTATACTTAATAAATTTTTTCTTTAATTTTTATGGACTTCAGTCGAAAAA

AAAATTGAAATTGCTACATCCTCACACACAAACACACACGCACACAAGTTAGTCAGAATTGCACCAAACAACCCTATGACCCACCAATTTAATGCTACCT

TTTGATCCCTTGCACACCGAAGATCATTTATTTTGACGGGATTGCGTAGGATCACTGGGGAAACTTGTAAAAATACTGGAATTGGTGATTTTCCATGAGT

TAGGAACCTGGCGTGCTGACAGGTTAGCACTTAGCAACCTGTGTCATAAGTCGATGATTTTATACCAAATCTGAGTTCATTGTCCTCTCTCTGATTCTCT

CTCAACCGAACTCTTTCAATCTCCGTCCTCTCTCAGTCTGTCAGTCTCTGCTCTTCGTCATCTCCTACACCTGCAGTCTAAGCCTTTGTTCTTCGTCGTT

ATTTGTGAAACAGTGAAACTTGAGTGAAAAGCCACTCGGTTAGGATCTCATCTCTAGAAGCACTTACAAAGCCACAAAGAAGGATTATTTGATTTACAAG

AACAAGTTATTGCTAAGTGTTTAAAATATTGGTTGTCATGCCAGTTTGACTAGATTAACAAGTATGGCAAGAATTAACTAAAACTATGATGCATGCATGA

ATAGGCCAGGGAGCAAGTGGAAAAAGTACGGAAGGCACAAAAAATGTGGGCGAAGACAAGCTTCAAGAAAAGGCGCCAGTTTTTACGTATACTTTTGAAG

TATATAATTAAACATCAAGCACTTATATGCGAGTAAGAGCAGTTCTTCTCCGTGGTCATTGAAGGAAGATTTAAGTGTTGGTATTTTTTGTTAACTTACA

TAATGTTGCTCTGATGATATTCTGGGAAGAATGTTAACGACTTCTGTTTTTAGTTATCCTATTGTTCTTTTCATTAGCCTAAAATATATATGAATTATAC

TTATTGCATGAGAATATTTATAACTTTCTTGAGTATATTCTAATTTGTGTGCCTTTACCAGAATATCTTCACGCGATACTGGAAAGACAATGGTGGATGC

CTCTTTAGGAGAAATAATGACAACATGTGAGAAGATAAATTGGCTACTGTCGGAGGGTGAGCAGTGCCTAAAGCCTGAGTACCGGTATACACCTTAAACT

TTTTTTTTATAAATTTAGACCTAAAAACTAGTAGATGTAGATCTATACATATCTTTTTTCTTATAAGTGGGTACACCATAACCCCCCTCCGCCCAACCCC

AGTAATTGACACTTCCTCTCTGCTTAAAGGGACTGGTAAATCATCTGTCTCGGCTTATTTTATTTTTTAATATATTTTAGGTTCTTTCTCTGAAATTGTA

AAGTTTTAAATTTTTTGCATATATTCCACCAATTGTTTTACAAAAAAAAGAGGTGTTACCTTTCAAATATACTTAAATGACTTTTTATGGTAATTTTTTT

TTCTAAATGCTCATTCATGACTACTTTTATGTTTTGTGATAATTAAAAAAATTTACAGATCCTCTGGAAGAGCTATGCTTCATAAGAGAGCCAAAGTAGA

ATTTCACCCCCTTGGTGTTATCGGTGCCATTGTGTCGTGGAACTATCCTTTCCACAATATTTTTAACCCTATGTTGGCAGCAGTTTTTTCTGGAAATGGC

GTTGTGATTAAGGTATAAAATGACTTAATATCTGCTATCTACCTATCTAAAATGGTTCATTCCTTGAATGCATTACATCTTTTTAAGAATAAACATTGTT

GACTGATATGCTTTCACGGTGATTATATAACTTTCATTGACAAACTTATATTGAAACTGAGACTCTGCATAGGTCCACTTACAAGTTACTATAACATATA

TTATTTCAATTTGACAGAAATTCGTGTGATTCTGGTTGTAGATTTCGGAACATGCTAGTTGGTCTGGATGCTTTTACTTCAGAATCATCCAATCAGCCCT

TGCTGCCATAGGAGCTCCGGAGGACCTTGTTGAGGTGATAACAGGGTATAGCCTTATTTTGTGCCCTTCTTCCCCTGGTTTCCGTCACATCTGCATCTAT

AGCCTTAATTGGACTTTTCTTACCAGGTTTGCTGAAACAGGAGAAGCACTGGTATCTTCTGCTGACAAAGTCATTTTTGTTGGATCCCCTGGGGTTGGCA

AGATGGTATGATTCCTTTGGCCTATCAAATTGCTCCTTCTTGTCCTTCATTTCTAAATTGTATTCTCTAACAATAATAATTTTGTTTCCAATTCTGCAAA

ATATGGTTCTGAATGTGTTATGCATGGTTGATGTTTAATAAAGAAATTCTAGAGAGTACTCAGTCAATCTTTAACTGAGATGTATTTGAATCTTAAGCCT

GATCTGTAGCCAGAAAAAAAAAAAAGGAAAGAACAAGAAAGTAACCTCAAAGGTTGTTGTAGTAGAGTGATGCACTGGCAGATCTACATTGTGGCCAGGG

TGTGCAAGTGCACCCCCCTCATTTTAAAAAAATCACAAATAAAAGTTTTTTTTCACATAAAATTACAAGTAAAATCTTATATTTTTTAATTTGAACCCAT

TGATTTAATCTATTTAATCTATATTTATATATTTGAACCCATTGATTTTAGTTTTATAATTTGCCCCCACTAACTCTGGATCCTGAGTCTGCCACTAGAT

GTCTTGGAAGCCGAATGAATGCTGGGGATAGTATCCACCTTTTAATACAGTAAGATGTCTGTGTTCTAATGATATGCTGCATTCTTTTATACAGATAATG

AGCAATGCTGCCGAGACACTTATCCCAGTTACACTAGAGCTTGGTGGAAAAGATGCATTTATTGTTTGTGAAGATGTAGATGTGGATCTTGTATGAATCC

CTTCCAACATTTGGCTAACAAATGTTAACCTTGCAACTTAATGAAATACTCATTCTTGAAGCCCATAGTTTTAACTGGCTGATTTGCTGAATATAGGTTG

CTCAAATTGCTGTCAGGGCTGCTCTTCAGTCAAGTGGGCAGAACTGTGCTGGGGCTGAGAGGTTTTATGTCCACAGGAAAATTTATGCATCCTTTGTCAG

TAAAGTTACCAAAATTATAAAATCCATTACAGCTGTAAATGCCCCTTTAAAACATTTCATTGGTAAAAATTCTTAATCCTGCATCTTTCTTTTTAAGCCA

ACATGAGCTTTTTAATAACACGTGCTTGCACTTAATTGTTACAGCATTGCTATTTCAGTAATCTATCAGCTTGTCTTTACATCTGTTCTAGTCTTGTATT

CTGAGACCTTGATCAGAATAAGTATTTGTGACAAAAAAAATTAGCGATAAACCCCAATTGTGGAGATGAGAATGACCCACAAAACCAGAAGCAAATGATA

GAACCCTGCAGAACTTAGAGGGTCTGCACCTCCCTTCCCCGAAGCCCCAAAACTTACACAATGACTGCTTGCGTCCCTCCTATCCCCCGTTCCCTTTTAT

CTTTGTCTCAAATTGACTCCCCCACCTGCATACTTGCCATGTTGCCAAGGTTGTTACAACTCAAATCCTTGTTCTGCCCCTTACTGATATTTCCCACACG

GTGAGTACCCAACAGATGTTCATATGATATGATTCATGATATAACCTTGTCCTTTTATTTGTCGATTTTTTCCTTTCCTCTTTTCATTTTCCTCCCTTTT

AGCCCTAAGGAAGCATGAGCTATATAAATATTTTCAGAGTTAAATTATACACAGATAAATGTGCATTTGTATATCTTTAAAACTCTTTAAATTTTAACCA

AAAAAAAAAAAAAAAAACTCATTCTAGTCTATCAAAGCCCTTGGCCATCAAGTCTAAGAAAAAGTTAAATACCAGTCTATCAAAGCCCTTGGTCACCTTC

ATTGAAAAAGACAGCTTCTAAAACCGGTTCATCAAGAGATAAATGGGCTATTTCAAGGATTCCATTCTCATCAAGTGAAAACCTATCTCTTGCAATATCC

CACATTCTTGTTTGAGGAATTTCTTGAAGTGAGGCGAAGATTGCTATGAACATATACAAGATCCTCCGTTCTTTTATGTGTCATCTTATTTCTTTTCAAA

CTATGTATGAAATTTTAGGTGCTCCAAATCCTTTCACAACAAGAAGATGAAGATGGTTGCCCAAGTAGCTCACCATGAACAAGCCACCAAGATTTTGTAT

CCAATTCACCTTTGTCCCTTAAAGGAGTCAACATCATGAAAGCCTTCTCTTCCACCAAAAAAATTTGCAAACTCCAAATTGGCTTGTCTCCTTACATCCA

CATCATCAAAGTATCTTCTAAAGCATTTCTTCCTTTCATCAGTAAGTTCCACACCTTGATGTGAGGGAACTCTTGATGGATCTTCACTTAACCATTCATG

ACTGTAATATCTACAACATTAAAAAAAAAATATAAACATAAATTAATATAATCCACAAAGAAATGCATGTAAAAAAATATAATCATAAATTAATTTAGGA

TATAAAGTATTACCTTGGGTTTAAAGAATGTGCTAGGCAATGAAGAGGTGTGCTACTCTTAGTCCAATGGTCCATTAATATTTTGTGCACTACGTCATAA

AAGGATGATTGTTCATTTTCTGTCTTTCTTTCTTGTCGATATATGACTTGTCTCACCTTTTCAATCATTGAATCCCACATTTCATATACCAAGTGTAGAA

CAACCATATCAATATCTATCTTTCTAAGAACATAAATAGGAGCAGTGAAAGAAAGTATGTAATCAACCTTGTCCCACCAAATATCATCCAACAAAGTTTC

TTCCATTTTTTTTTTGCCTTTCCAACATCATTGTCGAGTAGACTATTCAGGACTAATAACCATCTTTTGGAGTTCTTCTTTCAAACTTCTAAACCTCTTG

AGCATCACAACAGTTGAAGCAAATCTTGTAGGAGCAACAGAAAGCAACTTCAATGAGTTGAAGTTGTTGTACAATGATAATCTCATAGAGTGTCCCATGA

TAAAAAATTTAATCAAATTAGCATCGCCTGCAATTTATGTGATCCAAGAACATTGTTCATAAGTAACATTGTTTTTCTTAGCATCCTTGGCTGCACAAAT

ATTTTTCAATGCAAGTTCAAGGTGTGCGCAGCACAAGGAGTCCAATAGATGACAGGAAACTCATTCTCTATGATCATACATGCTGCCTTACATACAACTG

CATTATTAGTCACAATTTGTACCACATTTGATGACCCAACCTCCATAATTGAATCTTTGATGTGTTGAGCAATAAAATCCTTGTCCTTTATATCACCAAA

GCAATTTACCGCCTTTAAGAACATTGGTCCACTCTCAGTGACAGCCATGAAGTTGATAAGAGGGCTTCTTTGGGGATCACTCCACCCATCATTAACAGTG

GTCACACCTTTCTGATTCCATGAATTTCTTATCGGTTGCAAAAGGTTCACCACATGTCTTCTCTTTGTTGTAAGAAAGGTAGTTCTCAACTTATTGTAAC

CTAGAGGCTTATATCCACTGAGATTAGGAGTATTGGCAGCATAAGACAATGCCTCCCTATAATAAGGATTCCTTGCTAAATGAAATGGTAGCCCTGAAGA

GAAAAACATCCTTGCAATTCTGGCATCAAGATCATCTTTGCCTTGCAAATTGAAACCTTTTTCTAAAGGACCTAGGACTTTTGGAGGAGCACCATCTTGT

TTCTGTTTTCCTTCCCCAGGTAGAGAGACACATTTTTTCTTTGAACTTTGTATCTTCAAAGTTGCTTCATTGTCCAGTTTTTTTAATTCGATAATTTTTG

GAGGAGTCACCTTTGAACAAGGTCGAACTGCTTCTCTCAAGATTTTCAGCAGGTGAGCCCTTACTCGGGTGTAAGACCCAATGAATGAGATTTCACACAA

ATTGCATTTGATCATGGCATTTCCCCCGCCAGGAGTCTTTCTTAACTTCGTGACATATCTCCATAGGGGTTTGATTTGATCCTCTTGTTCCATTGCTTCA

TTAGCACTACTCATCTCTACAATAAAAAAATGGATATAATAAGAATTAAGCAAAATAAAACATTGAACAAAACCTATATATGTATGAACGAGCACATGAC

CAGTTAGGGAAAAAAAGGGAAAACTTTGATTAAATAGAGACAAAATAGAAAGTCCATAGTCACTTTCTCTGTTTTATGATCAGCCAAACAAAGAATTTTA

TTTATAACAAACCCACAATGCAGCCAGAGAAACAGCCAAACAGGGAGTGTCAAACCCAGAAACCCAGAATTTTATATTATGAAACAAGAAAAATAAAACA

TAGAAAAGAGAAACAGGGGAGCAACAGAAAAATAAAACGTAGAAAAGAGAAACAACAGTGCAACAGAAAAATAAAACGTAGAAAAGAGAGAAATAGGGGA

GTTACCTCGTGTTCTTCGCACTGCAATGGAGGTCCAGCAAGGTCTGAGCGCAGCGATGGCGTGCTCTGCGTGCAGCGACAGAGGTCCGGTGTGGTCTGAG

CGCGGCGGTCTGCGTGTAGCAATGACATGCTATGCACGTGGCGCAGAGGTCCGACAATGCTGTTTGGTTTGCTTGCAGAGCAGGAAACGTGAAAGATAAG

AAGGTTCATTTCGTGCGTGAGAACTGAGGCAGTTCATTCTCTTTTGTAAATGTTTTTCTTTAATTTTTTTTAAAAAATAATAAAAAATCAGTGACATGTC

TGCGTCCGGATGGTGGGCCGTGTCCAATTGCGCGTGTCCAACGAACTCAGATGCACCTGACACCGCGTTGCACGCGAGTCGTGTCTGAATCTGTATCCGG

TGCGATTCCGATGAGGGGCACGCCCTTGCGCGGTCGCATCCGTGCTTCGTAGTTGATATTTAAGTAACTTGCTGGTCAACTAGCAAATTGATAGAACTTA

CTCAATTTATTCATTTAGATGTGAACAAATTACTATTGGCATCCTCCAATTTCATTCTGAAATAATAGTTTTTTTTTATAAAAAAAAGCACAGGGAATCT

GGATTCATGACTCATATAGATAGGGGAAGGAGTGAAAGGGTCATGAACTTGGATAAATCCCACACATTGCATTAACATTGAAGATTGGGAACTGCTATAC

TATCAACACTTTTTCACTGATTATTGGAGAACATGCATCTGCCTCAGAAATATTTAGTATTAAGCATTCCATTTTAAGCTTTCCAGTTAATGGTTTCTTT

CAGGGCCCACCTCTTGCTGGAAAGTATGATATGGGAGCTTTATGTATGCATGCACATTCTGAAATGCTTGAAGCCCTTATAAATGATGCTTTAGACAAAG

GAGCTGAAATTATTGCACGTGGAAGTTTTGGACCTATAGGTGAAGATGCAGTTGATCAATATTTTCCCCCTACAGTGATTGTGAATGTGAATCACTCAAT

GAGATTGATGCAAGAAGAGGTAGAAAAACTTGCTGTTTTCTCTGCCATAGGCATTGCCATTTTTTTTTTCCTTTTCCATCTGTCAAGCGTTGCACTGATC

AGCCATATTGTCCATATCTATGTAACATTGTAACTAAAATACATATCGTTTCATGTCTTGACTAAAAGCATTGGATATCTTTAAATACGGATAATTGTGT

CTCCTCATCTCATGTTATATATGTCCTGCAACTATGCATTTATATTCAAGAATATATTTCTATATATCTTTTGTGCTTCATAAACTGCTTACTTGATTCA

TTTGTATATTTTAATGACTTCTAGGCATTTGGACCAATCATGCCAATAATGAAATTTAGCTCTGATGAAGAGGTTGTCAGGCTTGCAAATGACTCAAAAT

ATGGGCTTGGGTGTAACGTTTTCTCAGGCAGTCAGAGCCGTGCTAGAGAGATAGCTTCCCAGATACATTGTGGGCTTGCTGCAGTTAATGATTTTGCATC

AACATACATGTGTCAGGTAAATTTATTTTACTATTTATCTTCATCTCTCTATCAACATACATGTGATATGAACTGTGCTATGTTGATCACATTCAGCTGC

TAGAGAGAGATTTTTGAAATATTAGTAGGTGCACCTGAGTAAGGAGTTTCTGATTAGAAAGTTCGTCAAAGAAAAAATTGAAATGGGGGTCTTTGCCAAT

TTGGCTATGGAACCAACTATTCGCTTATTCATGAAAAAACACAATTTCCTTAATCCTTTCTGTTTATAATTTACTATAACATCTACTTTGCCACAAAATA

ACGTATTTAGGAAAGATCTTCTGATTCAAGTGACAATATATGAATGTTATATGAGAAGAGTTCAATTATTTTGTTTTTTTCGGCTATTATAATTATGTAG

TTCTTTAGCCAAATATGGCCGGGATATGTTTACTACTCAACCTAGGTCACAATTTTAGTTTCCACTGAATTAAAGTTGCATTGTGAATTTTCCACTATTC

CAGTCCCTTCCATTTGGGGGCGTGAAAAACAGTGGATTTGGACGATTCGGTGGTGTTGAGGGTTTGCGAGCCTGCTGCCTTGTAAAATCTGTTGTTGAAG

ATAGATGGTGGCCATTCATCAAAACCGTGATACCGAAGCCTATTCAGGTTAGTCTGGAAAACCTTTCATAATTGTAAATCATTTTACTTCACTTCAAGTT

GAAAGATACTGAGATGTGTTTTTCCTCTGCCCTAGTATCCTGTAGCTGAAAATGGATTTGAGTTCCAGGAGTCGCTTGTTGAAGCACTATACGGGCTCAG

CGTGTGGGACCGTTTGCAAGCATTGGTTAACGTGCTGAAAATGCTTACTGAGCAGAACTCTACAAGTGGCAGCAGAAAAAAGAAAAATGACTAATCATGT

CAGGACTGGTAACATTCCATATGGTTCATATTCATATCATCATCATGTTCAAATAATTGAGGGCCTTTCGGTTTGTCGTTTATCTCCCAAGAAGACTCCA

ACTCTAATTTGCAGTAGCCATTATTAACTCCTGCCATTTTTGCTTTGCTTATCGATGACAATCCTGGGCTACTTGTCTGATGAACGTTCATATGGATTTT

ATATCTTTATGGTAATGAACTTACAGCTAGAAATTAGCAACTTACAACTCTTTTTTTCTTAATTAAAAAAATGAATATAA

>Glyma.15G140900 | Chr15:11538070..11545857 reverse

ATCAAGACAATTTCTTCTTTCATCGCCTAAAGCTTCCCCAGATTCTTTCACTGTCTTCAACCATTGTCCTCAAAACAACAATCGAAACTCCGCGGAAGCT

TCTCGAAACCCTACCGAGGCGATTTTCTCACTCACTCCGAATCTGCTGCCAAGTTCCAATGGCGTTTTGGTGGCCATTGCTCGTTCTGGCACTCGCTTTC

GCCATCTGCAAGTTCCTTCTCATTCTCATTCCTCCCAAGGTTCCTTCCATCGACGTCGACGCTTCCGATGGTACTCTTCCTCCATCACTCTCTATTGCTC

GTAAATTTGTTGCGACACTGAAATGTGATGTGGATTTTTTACTCTAACAGTGTTGGACGATGGGAGCCTGACGCAGGAGAACAGTTTCATATACGTGAGT

GCTATGCATCTGCAATTTGAATTTCATAAATCGCTGTGTAGTGAGTAGTGACTTTTTATTTTTATTTTTATTTTTATCGGTTTTATGCTTTTGTGTTTTG

CTTGATCGGTGTGATTTGCTTATATATGTGGAATTTAAAATTTGAATTCGGATTAATTGCAAATCTATGTCGTGCGGAGTTTATGTAGGCTAGGTATGAA

CTTGTTCACGACGTGACGCTTTGAATGCAATGATTTGGAATTGGAGCTTTTGAAATTTTTGTTTTTTCTGTTTTGAAGAATATGGCTGATAAGGTCTCAT

TCATGATTGATTCATAATCAGGTGCCTCCGAGGGGAACGGCACAGCAATCGAGCGGAAAAGTTCAGTGCTATGAGCCTGCTACCATGAAATATTTGGGAT

ATGTTCCTGCATTGACGCCGGATGAGGTATGTGAGCCTATGCTTCTAAGGGAACTAATTGTTGTGATGCATGCTTTTAGTGTCTAGTTTGAGCTACATTT

GTTTTTAGTATCCTTTTGTTGTGAGCCTATGCTTTTAGTATCCTTTTGTTGTTTTTTTTTTCCTCTGGGTAATTAGTATTTAGAAATTTTGATATTTAAG

GATACGATGATACTTAGGACTTTCTGTGGCTGAAGAAAAGTACCAGGCAAAAAGTAGTTTATATAAGGTAGCATTGATCATCAGAAATATGCTAGTTGTG

CTGAAGTTGACATGTCATTAGCTTCTAGCTAAACTTGTATGTTAAAGCTTCATTTTTGCATTTTGCATACTTGTCTATAGTGAAATGCAAATTCAATGGA

ATTTCATTCAAGCCGTATGTTTATTGATGTTAAATATGGAAATTCCTTTTTCCCAACGTTGACTACCTTTTGGTTTTCACTTTTCATTGATGTATTACTT

TTCGTGCGTATGAATGTGTATTAAGCCCGTGCCTAGTGGTTAATTAAAGTCCTCGTGGCACTCTTAACGATCCATGTGGGGGTACAAGTGACAGTTACGG

GTGAAAGTAAGCCGAGTTGAGCCAAACCTAGCACAAACTCGGTGCAATGAATTCCGTTTCTTGGCTCAACCCTTGCTCTTTGGATGCTTCAACTTGATCA

TGTACTTTATACTCAATAATTTTTTTCTTTAATTTTATGTACTTTAATTGAAAAAAAATTGAAATTGCTACATGCTCACACGCAAACACACATGCACACA

AGTTAGTCAGAATTGCACCGAACAACCCTATGACCTACAAATTTAATGCTACCTTTTGATACCTTGCACACTGAAGATCGTTTATTTTGATATTTTTGCG

TGGGATCAGTGGGGAAACTTGTAAAAATACTGGAATTGGTGATTTTCCATGAGTTAGGCGCCTGACATGCTGACAGGTTAGCACTTAGCAACCTGTGACA

TAAGCAAATGGTTTTATGCCAAATCTGAGTTCATTGTCCTCTCTCTCTCTCTCAACCAAACTCTCTCAATCTCAGTCCTCTCTCAGTCTGTCAATCTTTG

CTCTTCATCATCTCCTACACCTGCAGTCTAAGCCTTTGTTCTTCGTCGTTATTTGTGAAACTTAAATTTTGTGAGTGGTTCCCTTATTCTCAGTATCAAA

CTTCAGTTCCAACTTCCAAGAGCTCCACTTCCATTTTTCTGTTCCTCTGTTTTGCTCTTCACTTTTACTTCAGTACTTCTCTGTTTTTCTTTTTTGCTTT

TTAGTTTCCTATATGTTGTCTAGAACCTACATATTTTATATTGTAGTTGTTAGCTTGTAACTTTAGCTTAAGTTCTAACATGTGTTGAATTAAGAATCGG

ATGAAATAACTTGTGTGAAAAGCCACTCGGCTAGGATCTGATCTCTAGAAGCACTTACAAAGCCACATAGAAGGATTATTTGATTCACAAGAACAAGTTA

TTGCAAAGTGTTTTAAAATATTGGTTGCCACACCAGTTTGACTAGATTAACAAGTATAGCAAGAATTAACTAGAACTATGATGCATGCATGAATAGGTCA

AGGAGCAAGTGGAAAAAGTACGGAAGGCACAAAAAATGTGGGCGAAGACAAGCTTCAAGAAAAGGCGCCACTTTTTACGTATACTTTTGAAGTATATAAT

TAAACATCAAGCACTTATATGCGAGTAAGAGCAGTTCTTCTCCATGGTCATTGAAGGAAGTTTTAAGTGTTGGCATTTTTTGTTAACTTATATAACGTTG

CTCTCATGATATTCTGGGAAGAATGCTAATGTCTTTTGTTTTTAGTTATCCTATTGTTTTATTCATTAGCCTAAAATATATGTGAATTATACTTATAACA

TGAGTATATTAATAACTTTGTTGAGTATATTCTAATTTGTGTGCCTTTACCAGAATATCTTCTCGCGATACTGGAAAGACAATGGTAGATGCCTCTTTAG

GAGAAATAATGACAACATGTGAGAAGATAAATTGGCTACTATCAGAGGGTGAGCAGTGCCTAAAGCCTGAGTACCGGTATACACCTCGAACTTTTTTTTT

TCATTTAGACCTAAAAATTAGTACCGGTATCCACCTCAAACTAGTGTTTTTCTTGTAACACCATAGCACCCCCTCCCCCCAACCCTAGTAATTGACACTC

CCTCTCTGCTTAAAGGGACTGTGGTAAATCGTTTGTCTCAGTTTATTTTATTTTATAATATTTTTTAGGTTCTTTCTCTGAAATTGTAAAGTTTTAAATT

TTTTGCATATATTCCCCCAATTATTTTACAAAAAAAGAGGTATTACCTTTTTAATATACTTAAATGACTTTTTATGGTAAAATAAAATTACAAATTTTCT

AAATGCACATTCATGACTACTTTTATGTTTTGTGATAATTAAAAAAACTCACAGGTCCTCTGGAAGAGCTATGCTTCATAAGAGATCCAAAGTAGAATTT

CTCCCCCTTGGTGTTATCGGTGCCATTGTGTCGTGGAACTATCCTTTCCACAATATTTTTAACCCTATGTTGGCAGCAGTTTTTTCTGGAAATGGCATTG

TGATTAAGGTATAAAATGACTTAATATCTGCTATCTACCAATCTAAAATTGTTCATTTTGTGAATGCATCACGCCTTTTTAAGAATAAACATTGTTGACT

GATATGCTTTCATGGTGATTAAATTACATTCATTGACTAACTTATATTAAAACGGAGACTTCGCATGGGTTAATACCTCCACTTCCAAGTTACTGTAAAA

TTTATGATTTCAATTTGACAGAAATCCATGTGATTCTGGTTGTAGATTTCGGAACATGCAAGTTGGTCTGGATGCTTTTACTTCCGAATCATCCAATCAG

CACTTGCTGCCATAGGAGCTCCGGAGGAACTTGTTGAGGTGATAACAGGGTATAGTCTTATTTTGTACCCTTCATTCTTCCTTCTCGGTTTCAATCACAT

CTGCATCTATAGCCTTAATTGGACTTTTCTTACCAGGTTTGCTGAAACAGGAGAAGCACTGGTAGCTTCTGCTGACAAAGTCATTTTTGTTGGATCCCCT

GGGGTTGGCAAGATGGTATGATTCCTTTGGCCTATCAAATTGGTCCATCCTGTCCTTCATTTCTAAATTTTATTCTATAACAATAGTAACTTTGTTTCCA

ATTCTGCAAAATAAGCTTCTGAATGTGTTATGCATGGTAGATATTTAATAAAGAAATTCTAGTGAGTACTCAATCAATGTTTAACTTTGGATCTGCTTAA

GCCCGACCTGTAGCCAGAAAAAAAAGGAACGATAAAGAAAGTAACCTCAAAAGTTGTTGTGGTAGAGTGATGTCTTCACTGGCAGATCTACATTGTGTCC

AGGGTGTGCAAGTGCACCCTCTCATTTAAAAAAAAAATCGCAAATAAAAGTTTTTTTCACATAAAATTACAATTAAAATCTTATATTTTTATATTTTATT

TCATCTATATTTATATATTTGAACCACTGATATTAGTTTTTGTAATTTGCCACCACTGACTCAGGATCCTGAGTCCGCCGCTAGATGTCTTGGAAGCTGA

ATGAATGATGGGGATAGTATCCACCTTTTAATGTTGTAACATGTCTTTGTGTTCTGATGATATGCTGCATTCTTTTATACAGATAATGAGCAATGCTGCT

GAGACACTTATCCCAGTTACACTAGAGCTTGGTGGAAAAGATGTATTTATTGTTTGTGAAGATGCAGATGTGGATCATGTATGAATTCCTTCCAACATTT

GGCTGGCAAATGTTAACCTTGCAACTTAATGAAATACTCATTCTTGAATGCCATAGTTTTAACTGGCTGATTTACTGAATATAGGTTGCTCAAGTTGCTG

TCAGGGCTGCTCTGCAGTCAAGTGGGCAGAACTGTGCTGGGGCAGAGCGATTTTATGTCCACAGGAACATATATGCATCCTTTGTCAGTAAAGTTACCAA

AATTATAAAATCTGTTACAGCTGTAAGTGTCCCTTTTAAAACATTTCATTGGTAAAAAGTCTTTTTAAGTCAACATAGGCTTTTTAGTCACAGTGCTTGC

ACTTAATTGTTACAGCATTGCTATTTCAGCAATCTATCAGCTTGTCTTTACATCTGTTTTAGTCTTGTATTCTGAGACCTTGATAGGAATAAGTATTTGT

TACAGAAAAAACTATTGATAAACCCCAATTGTAGAGACAAGAATGACTCACAAAACCAGAAGCAAATGATAGAACCCTGCAGAACTTAAATTGTCTGCAC

CTCCCTTCCCTGAAGCCCCAAAACTTACTCAATGACTGCTTGCCTCCCACCTACCCCCCGCTTCCTTTTATCTTTGCCTCTATTTGACTCCCCACCTGCA

TGCTTGCCATGTTGGCAAGGTTGTTACAACTCAAATCCTTGTTCTGCCCCTTACTGATATTTCCCACACGGTGAGTATCCAACAGATGTTCATATGATTC

ATGATATGTTACCTTTTCCTTTTTTGTTGTTGAATTTTTCATTTCCTCTTCTTTTCATTTTCCTCCCTTTTGGCCTTGTGGAAGCATTAGCTTTGTAAAG

CTTTTCAGACTTAAATTATACACAGATTCATGTGAATTTCTGTCTCTTTAAAGTGATATGTACGTAACTTTGTCAGCTAGCAAATTGATAGAACTTGCTC

AATTTATTCATTTAGATGTGAACAAATTACTTTTGGTATCCTCCAATTTCATTCTGAATTAATAGTTTTTCTTAAAAATAAAGCACAGGGAATCTGGATT

CATGACCCATAGATAGGGGATGGGTAAAAGGGTCAGGAACTTGGATAGATCCCACACATTGCATTAACATTGAAGATTAGGAACTGCTATACTATCAAGA

ACCTTTTTTCGCTGATTATTGGAGAATATGCATCTCCCTCGGAAATATCTAGTATTAAGAACTCCTTTTTAATCTTTCTGCTAATGGTTTCTGCAGGGCC

CACCACTTGCTGGAAAGTATGATATGGGAGCTTTGTGTATGCATGCACATTCTGAAAAGCTTGAAGCCCTTATAAATGATGCTTTAGACAAAGGAGCTGA

AATTATTGCACGTGGAAGTTTTGGACATATAGGTGAAGATGCAGTTGATCAATATTTCCCCCCTACAGTGATTGTGAATGTGAATCACTCGATGAGATTA

ATGCAAGAAGAGGTAGAAAAACTCTGTTTTCTCTGGCATAGGCATTGCCATTTCTTTTCCATTTGTTGATGTCAAGTGTGACACTGATCAGCCATTTTGT

CCATATCAATGCAACATTGTAACCAAGAAAAATATTGTTCATATCTTGACTAGAAGCATTGGAGATCTTTAAATATGGATAATTGTGTCTCATGTCGTAT

ATGTACTGCAACTATGCATTTATATTCAAGAATATCTTTCTATATATCTTTTATGCTTCAAAAACTGCTTACTTGATTCATCCGTGTATTTTGATGACTT

CTAGGCGTTTGGACCAATCATGCCAATAATGAAATTTAGCTCTGATGAAGAGGTTGTCAGGCTTGCAAATGATTCAAAATATGGGCTTGGGTGTAATGTT

TTCTCAGGCAGTCAGAGCCGTGCTAGAGAGATAGCTTCCCAGATACATTGTGGGCTTGCTGCGGTTAATGATTTTGCTGCAACATACATGTGTCAGGTAA

ATTTATTTTACTATTTTATCTTTATTTCTCTCTCAAAAGGGTTTTCACCCTTAACCAACCTAGTAGCATTGATATGACCAATGGTGATCACATTCAGCTG

CTAGAGAGACTTTTGAAATATTAGCAGTTGCACCTGAGTAAGGAATTTCTGATTAGAAAGTTGGTCTACGAAAAGTTTGAAATGGGGATCTTTGCCAATT

TGGCTATGGAACCAACTATTGGTTTATTCACGAGAAAACACAATCTCTTTATTGTTTCTAATTTACTTTAATATCTACTTTGCCATAAAATACATACTTA

GGAAAACTTCTGATTCAAGTGACAATAGATGAATGCTATATGAGAAGAGTTTTATTATTTTATATGTTTTTCTGGCTTATTATAATTATGCAGTTCTTTT

AGCCAAATTGCCAGAATATGTTCCCAATTCAACCTAGGTTACCTCTGTAATTTTAATTTCCACTGAATTGAAGTTGCATTGTGAATTTTCCACTATTCCA

GTCCCTGCCATTTGGGGGTGTGAAAAACAGTGGATTTGGACGATTCGGTGGTGTTGAGGGTTTGCGAGCATGCTGCCTTGTAAAATCTGTTGTTGAAGAT

AGATGGTGGCCATTCATAAAAACTGTGATACCGAAGCCTATACAGGTTAGTCTGGAAAATCTTTCATAATTATATATCATTTTACTTCACTTCGAGTGGA

GAGATACTGAGATGGGCTTTTTCTCTGCCCTAGTATCCTGTTGCAGAAAATGGATTTGAGTTCCAGGAGTCGCTTGTTGAAGCACTTTACGGGCTCAGCG

TATGGGACCGTTTGCAAGCATTGGTGAACGTGTTGAAAATGCTTACTGAGCAGAACTCTACAAGTGGCAGCAGAAAAAAGAAAAATGACTAATCGTGTCA

GGACGGGTAACATTCCTAATGGTTCATATTCATATCATCATCATGTTCAAATAATTGAGGTCCTTTCGGTTTGTCGTTTATCTCCCAAGAAGACTCCAAC

TCTAATTTGTAGTAGCCATTATTAACTCCTGCCATTTTTGATGTGCTTGTTAGATGACAATCCTGGGCTACTTGTCTGATGAACGTTCATATGGATTTTA

TATCATTATGGTAATGAAATTACAGCTAGAAAATAAGTAACTACTATCATGCCAGAATCTTTTTTTTTTAATTAGAAAACTGAATATAATCTGTACTGTT

AAATTACCCTCATCATCCTCGGAGTAGCTCAAAAACACTCCCATGTTATGAAATTTGACATTTTACTAATGATTAGATTATCAGATTC

>Glyma.17G033100 | Chr17:2416414..2423912 forward

TGGGATCGAGAGATCGAGAGAGAGACAACGAGTTTTGTGACTTGTGAGGGACTCAGGGAAGATTGCTGTCTCTTTATATAAGCGGCTAAGCTCATCTACT

AAAAATATTCAAGATATTATCAACTATCAAGTACCAGTACCATTTCTTTCTTTACTATTTTTTCTTTGAGGATTTTAATCCCTCCTTACTTTCACATAAA

AAGAGAGAGAGAGAAAGAAGGCATAGACTAGAGAGAACATACGAGTCCAATAGAGAAGTAAAGTGCCATAAAAAACGACAGGACGCATCACGCTACGAAT

GGAATCTGCGTTTCCTTTTTTCCTTATTTTCGTGGCACGGAGTATGAATTTTCCGTTTCGTTTTTTTTTTCCGTTTCGTTTTTTCCTTGCTGACTATGAT

TGTTATTGTTAATATCAATGCCATGCTCATCTCCACGAGCTTAAGCTTACGCATTCACTGACTGGGAGCATTTTCCTTTAAAACACTCCGCAGAAGCTTC

GAGTTAAAGAGCATCAATCCAAATCAAAATCCCTAGCTGGTTTTCTTCCGATTCGAAGAGAACGCGCTCTGAAACAGCGCATTTCCAATGGCGTTTTGGT

GGCCGTTGCTCGTTTTAGCATTCGCTTACGGTATTTGTAGGTTCCTTCTCATGCTCATTCCTCCTAAGGTTCCTTCCATCGACGTTGACACTTCCGATGG

TACTCTCTCTCTCTCTCTCTCCACACGCACGCACGCACACACACTTACACAATGAGGTTTGTTGTGACGGTCAAATTTGTGACAATGACAGTGTTAGACG

ATGGAAACCAAGCGCAGGAGAACAGTTTCATATATGTGAGTTCTCTCTCTCTCTTTCTGCTTTGGTTAACGAATCGCATATGAAAGAATCTTGCAAATCG

TTGCATGATAAGGTACCGTACTGCATTTAATTGCATTTTTTCAGAATCGTTATGATCTGTCATTGGTTCCAATCCAGGTACCTCCGAGGGGAACATCGCA

GCAATCAGGCAAGATAGTTCAGTGCTACGAGCCTGCAACTATGAAATATTTGGGATATGTTCCTGCATTGACGCGTGATGAGGTCAGTGAGCCTCAGCCC

GTGCTCTTGTAACAGTGGGCTAATGGTTATGATGAGTAGTATTTGTATCTATTTAACCGAAGCTAATGATTGATGGTTTAATTAGGAACTACGAAGCACT

GATACTTTAATTTACTTCAAGTATCTGTATCCGATATGTATCTTGTACCGATACTCCTAGATACTTTATTGTTACGTACATCGGTACATGTGAAGTATCC

AAACCTTTAACAGAAATTTATGAAAATTCGATACAAATACATATGAGACTCAGGAATAAGATTGGAAATTATGGATGCGTTATCCTTTTATGGATGATTT

CAAGAAAGTAATAAGATTGATACTATTCCTATTTATAGACAATAGGAATTGATTAATGATAACAAGGATTTACAATTTTTATTTTGTTTTCTTTATTTGG

ATTTGCATAATTGCAAATATATATTTATTGTGTCTTATGAATTTTTATGCATATTTAGATATGTGTTGCGGTATCATATATGTATCGAGTTGAAAATAAG

TATCATATCTGTATATCATAGACTGGGAAGCACAATTAGTTTTCAATTAAACTTAGTTTGTGATTAGTGTTTGATGAAGTGTGATTTTAACTTTTTAAGA

ATTAATTTTCATAGCTAAAGAGATGGAGAGCGAATAAATAGAAGAGATGTGTTCGTGTGTGTTTGGAAACCTGGTTTGCAACTAACATAATGCCAATCCA

CCCTTAAGGTTGCTTCTACATTTGGTGCATTCAAATGTGTAAGCTTCCATTTAAAATGGCACAAACTGGTTTCCAAACACTCAAGAAATCTACAAGGACA

TCATTGCCATGCAAGAAATATGCTGGTTGTGCTGAATTAAATCTGACATGTCATAGCTTCTACCTTCTGGATAAAATTTTATACTAACTAGAGCAGTGCC

GGTGTGCATGTGCAACAACACGATCCAAATAGAATATCACTATTTTACAATAATATTACCAGAATAACACTATTTAAGCTGGAAGCGGACTTGACAATGA

TTGAAATGTAGAACATTGTTGGACAGTTTTAATATAATTATTGATTATTCATTTTGTTGTTTATAGTACAAGTTCTATGGAATTCCTTTGATTTTTATGT

TTGTTGTTCTCACAATTTTTCTTCATGTTGACCATATTTAGTTTCCATCCTTTTTTTCTTCTTGTTTATGAATGTCTGCACTTATTTGTAAAAATGAATT

AATGTATAATGTATCCCATTAGCATTCTTATTGATCTTTATACATCTGGAAACATTAATGACAAAGCATTCACAGATACTAAGAGGAGAATATTGATGTA

AAGTGCCAGTTGTTGCAGAGCATGCATGATAAAATACTGTTTCCTAGTTTTAGAGGAATTTTTTTACTAGCTTTGCTGGATTAACTATAACTGCTTTGCA

TCTATGGATAGGTCAAAGACCGAGTGGCAAAAGTAAGGAAGGCGCAAAAAATGTGGGCAAAGTCCAGCTTCAAGCAAAGACGCCTATTTTTGCGTATACT

TTTAAAGTATATAATTAAACATCAAGCGCTTATATGCGAGTAAGAACACTTTTCCATGTTTGTAAAAGTCTTCTCATTTTTTACACTTCTTGGAAACGTT

TGTGATACAGACTTGAACTTAGCAAATATGCAAAACTTTCATAACTATCCACTGTTTATAGTTATCCTACTGTTTAATTCATCTGGTCTGAAGTAGATGT

AAATTGTGATCATTGCATGAGAATGATGATAACATCTTGTAATTCATGTGCTTTAATTAGAATATCTTCGCGTGATACTGGAAAGACAATGGTAGATGCC

TCTTTAGGAGAAATAATGACAACATGTGAGAAGATCAATTGGCTACTGTCAGAGGGTGAGCAGTGGTTAAAGCCTGAATACCGGTACCTTCTTTTAGTAG

TTGACTATTTTCTTTTCTTAAACTGATTATGATGAATTATCTGTCTCTTTTTATTTTCTGTTCTTTATGTGCCTTTTTTTACACAAAAAACTAGGCCTTT

TAACTTATAAGATATGTATTGTTTTATTACAAAAAATCAAGTTATAACTTCTTCCTTATACATGCATTTAATATCACTATGGTGAAATTTCAAATTATCT

AAAGTCACTTCTATGTTCACTTTCTTTTTCCTTGTGTGTGATGAATAAAAATCTTTGCAGGTCTTCTGGAAGATCAATGCTTCATAAGAGAGCTAAAGTA

GAATTTCATCCCCTTGGTGTTATTGGAGCCATTGTTTCATGGAATTATCCCTTCCACAATATTTTTAATCCTATGCTGGCTGCAATTTTTTCTGGAAACG

GTATTGTGATTAAGGTATAAAATATCTTAATATCTACCTATCACAATTATTCTTCATGCTGCTGGTGTATTGTGTTGATTTTAGTTAGATTATTTTCCTT

GGTAAAATTAAAAAATAAAAATCTCTTGCATGGTGCGATACATACTCTTGTTTTAGAGATTTAAAACTATTGATGAACTTCCACCTAATAACCATTTAGA

AGATTGTTATGGTTTCCACTAACGCAACCAAGCTTGATCCTTTCTTTCCACCCATTTCTTCATTATTAAGCTGAATTTCAGCACAAGTTAAAATTGGCCT

ATGCACTATTGATGTATTAAGTGATAAAAAAGGCTATTGCTCAAGGATCATGTTAGAGTTGGAGGCATAAAACCATTTTTTAGCTTCCACCTACTGGCTT

AAGCTTTTAGGAGAATTGTTATTTAACCATATAAAATGCTTCTATGTTTTGCAATTTGAAAGAAAATCATGTGCTTTTTATTGTAGATATCAGAACATGC

AAGTTGGTCTGGATGCTTCTACTTCCGGATCATCCAATCAGCCCTTGCTGCCATAGGAGCTCCAGAGGACCTTGTTGAGGTGATAACAGGGTACGGCCAT

TTGTCTTACCATTCCTTTTTCCTTTTTGTTCTATGGTTGTGTTTATCACCTATGACATTGATTGGATCTTCTTCCAGGTTTGCTGAAACAGGAGAAGCAT

TAGTATCTTCTGTTGATAAAGTCATATTTGTTGGATCGCCTGGTGTTGGTAAGATGGTATGATTCTTCTGGCCAATTGAATGGGTTCACTCTGCTATCAC

CTGGAATTGTATCATAGTTCATTGCTTCCAATCATTGTCATATACTCCATTCATTTTTATGATGGCATCCTATTTCCCATGAATTGCACTATATGCTAAT

ATGCTTCATCTTTTTATGCAGATAATGAACAATGCTGCTAACACTCTTACACCAGTGACACTGGAGCTTGGTGGGAAAGATGCATTTATTGTTTGTGAAG

ACGTAGATCTGGACCATGTATGAATCCTTTCTCCCTGTTCAGATTCCCCAAAGCTTGGATTTAACTATTATTCACAAAAGCTTAATGAGATCTCCATTCT

TGAGTACCTTAGTTTTAACAGCCTTTGATCTGCTGAATATAGGTTGCACAAATTGCTGTCAGGGCTGTACTTCAGTCAAGTGGACAGAACTGTGCTGGGG

CAGAGCGATTTTATGTCCATAGGGAAATATATTCTTCTTTTGTTAGCTTAGTTACCAAAATTGTGAAGTCTGTTACAGCTGTAAGTGTTTTTAACAACAT

TCATTAAATCTAACTCTCTAACTCTATAACTTTCATAATAGGCTTCTGATGCATATAATCACATATGCTTGCACTTCATTTCTAGTTTGTCACAAGATTG

CTTTTTCAGAAATCTATCTTATTGTTTATACATCTGTTTTAGCTCTATGTCAAAAACTATGAGAAATAGACATTTGGTTCAGGATACATTGCATTGTCGT

CTATTTTAGTTGAACCTCTGCATGTTTTTTGTTTCTCCCATTTCTCCCTTTCTGTACCAGTGTGCCCCCAAGGAACCATTTTCTACATAAACCTTTAAAG

AAAAATATTAGCATCAACTTATTACTGGCTTTATATTCTAGTATGTGTGTGTGTGTGTTCTATGTATTTGGAACTCTTTAAAATGTATAGTATCTGTGAA

TTGTTGTTGATGTCTAGCAAACAAATAGAATTTAGAGATGAACAAATTGCATGGCATCACTGGGATGCTCCAATTCTCTTCTGAGATGATATTTTCCTTT

AAAACAAAAAGTACTGAAATCTGGAATTCATGATTATATACTGTTTGAGAGGAGGGACAAAAGTATCAGAAATTTATGTTCATTCACAGATGGCATTAAC

ATTGACGACTACAATAATCAAGAACTTGTTGCTTACTGATTATTGAAGAATGAACTTCTGTCCTAGGAGTATTGAGTATAGTCAATTTTTTTATTTAATT

TATTGGTTATTGATTTCTTGCAGGGCCCACCACTTGTTGGAAAGTATGATATGGGAGCTTTATGCATGCATGAGCATTCTGAAAAGCTTGAAGGCCTTGT

AAATGATGCTTTAGACAAAGGAGCTGAAATTGTTGCCCGTGGGAGTTTTGGACATATTGGTGAAGACGCAGTTGATCAATATTTCCCCCCTACTGTGATT

GTGAATGTGAACCACACAATGAGATTGATGCAAGAAGAGGTAGCAAAACTCACTATCTTTTCTACTTGAAGATGCCTTTATTTTCTTCTTTGATTATTAC

AAATATCCTACACACTCATACATCACTTCACATTGCCATAGTTTTCATCATCTAATTACAGTTAGTTGTTCCATGTTTTTTGCGCAATTACGCTTTGATT

TTACTGCACAAAACTATATTAGTGTGAATCAATGATAATGTGAGCCATGATGTGCAAGAAGCGTTATTACTATTGTTGTATTTAAGTTCATTGTTAGTAG

GTATGCATACATTCCATACCTACTTACCTAGACTGCAACCATTTGAGATTTTTTAAAATTTAGGTAATCATATCCCATGACAGTCATGCGCCTCAATTGT

GCATTTACATTCAAGAATTTCCCTTCTATATTCTATTATTCTATGCTTCAGCAACTGCTTACTAAATTCATCTGTGCAATTAAATACTTGACTAGGCATT

TGGACCAATCATGCCGATAATGAAATTCAGCTCTGATGAGGAGGTTGTCAGGCTTGCAAATGAGTCAAAATATGGGCTCGGCTGTGCTGTTTTCTCAGGC

AATCAGAGTCGTGCCAGAGAGATAGCTTCACAGATACATGCTGGGGTAGCTGCGGTTAATGATTTTGCATCAACATACATGTGTCAGGTAAATTTATTTC

ATCTTTAACTCTCTTGAAAGGATTTAATGGTAATTTTATCTTCTTGTCTATCTTGCATCTAAATGCTTGTTTAATATGTGTTGATCTGTTCTGAATGTGC

AACAAGTAGGCTGACTGCAGATTGTGCAGTTTTTTTCTTTTTCAAACTGCCTGGAAAAGTTAACTCAGCCTAGGTTATCCTCCATTTTCACTGAAGCAAT

TATGCATTATGATTTTGCAGTCCCTACCATTTGGGGGTGTGAAACATAGTGGATTTGGACGATTTGGTGGTGTAGAAGGTTTGCGAGCTTGCTGTCTTGT

TAAAGCAGTTGCTGAAGATCGATGGTGGCCATTTGTCAAAACCAAGATACCTAAGCCTATTCAGGTCAGTCAGGAAAACTGTCATTACTATTTCCTTCCG

ACATCAATTGTTTTGAGATAAAAGAAAGAAAGCTAATTCTCCAAAAAAAGAAAGAAAAAGAAAGCTAAAACATGCAAAGATATGGAAAAAAGGTACTAGT

ATTTTTTACTTCACCATGAATAGAAACTTTAATGGGCTTCTTGTCTGCTACAGTATCCTGTTGCAGAGAATGGATTCGAATTTCAGGAGTCACTTGTTGA

AGCGCTTTATGGTATTGGCATATGGGACCGTTTGCGAGCATTAGTAAACGTTTTAAAAATGCTTACTGAGCAGCATCCTGGTGGCGGCGGCAAGAGAAGA

AATGACTGATTGTCAGGTCAGGTAACATGCTAAATGGCTAATATTGTCATACTCAAACCCAGCTGCTAGGAAGCTGTAAGGGAAGAAATGAATAATTGGC

GGCAAGTCAAGAGAACACGCTAAATGGTTAATATTGCCAAGTTGGGTGGGGGGTGTCACTTTTGGTTGACGTATTTATATCTAACTACAATGCTTTTTTT

TTTGTACATGTTTCCTGTGCCATCATTTTTGTTGAGCTTTGGTATCACTTTAATATAATTTTTAAATGCTCTCCCCCCACCACCCACACACACCCAAAAT

CAAACGTTGCTATCTTTATCTGTTCTTGTCAAAGAAAACTCTCAGAAGTTATCATTGTTTTGTCTTTCTTTTCAAATGTAAGGGGATTTAAACTCAAACT

AGTTGACGATTATTTGGATTGCAGGAGATGGGATATTTTTGTGATTGTGGCCTTTGGTTTGATACCTATTCATTAGGGCATATAACAATGATCAACTTT

**The coding sequences of 53 soybean *ALDH* genes**

>Glyma.01G031500.1 CDS

ATGGCTTCATCAATGAGGATCTCAAGGCTGCTCTCTCGTTCTTTTCTTTCTGCTTCCACTACTCCTTTGTTCTCTAGAGGTGGAAGTGGTGCTCTTGGTG

CAGGGCTCAGTAAATTCAGCACTGCTGCTGCAATTGAAGAACCAATCAAACCACCAGTTAAAGTAGAACACACCCAGCTCTTAATCGATGGAAAATTCGT

TGATGCTGCTACTGGCAAAACTTTTCCAACGTTGGACCCCAGGACAGGAGATGTGATTTCTCATGTTGCTGAGGGTGACCATGAAGATGTTGATCGAGCA

GTTGCAGCTGCTCGCAAAGCCTTCGACCATGGACCTTGGCCAAAGATGACAGCCTATGAAAGACAAAGGATATTGCTGCGTGCTGCTGATCTGTTTGAAA

AGCACAATGATGAGCTTGCTGCTCTTGAGACTTGGGATAATGGGAAGCCATATGAACAATCTGCTCAAATCGAAATTCCAATGCTGGTTCGCCTATTTCG

ATACTATGCTGGTTGGGCAGATAAGATTCATGGTCTCACAGTTCCAGCTGATGGGCCATATCATGTGCAAACATTGCATGAACCCATTGGTGTTGCTGGT

CAGATCATTCCGTGGAACTTCCCTCTTGTCATGTTTGCCTGGAAGGTTGGGCCAGCATTGGCCTGTGGTAACACCATTGTTCTCAAAACAGCTGAGCAGA

CTCCATTATCTGCTTTGTATGCATCAAAGTTGCTTCATGAGGCTGGACTTCCTCCTGGCGTTCTGAATGTAATATCTGGCTTTGGTCCTACCGCTGGTGC

TGCCATTGCTAGTCACATGGACATAGATAAGCTTGCTTTCACTGGTTCAACCGAAACTGGAAAAGTTGTCCTTGAATTGGCAGCTAGAAGTAATCTTAAG

CCTGTTACTCTGGAGCTTGGTGGGAAATCTCCTTTTATAGTATGCGAAGATGCTGATGTAGATGAGGCTGTTGAGCTAGCACACTTTGCATTATTCTTTA

ATCAGGGACAATGCTGCTGTGCTGGGTCTAGAACATTTGTACACGAACGTGTGTACGATGAATTTATTGAGAAAGCAAAGGCACGTGCTTTGAAGCGTGC

TGTTGGTGATCCATTCAAGGGGGGAATAGAGCAAGGTCCTCAGATTGATTCAGAACAATTTCAGAAAATACTGAAGTATATTAGGTCCGGTGTTGAAAGT

GGAGCTACTCTTGAAACTGGAGGAGATAGATTTGGCAACTCAGGCTTCTATATTCAGCCTACTGTCTTCTCAAATGTTAAGGATGACATGCTGATTGCAA

AGGAAGAGATCTTTGGTCCAGTGCAAACCATATTAAAATTCAAGGACCTTGATGATGTGATTCAAAGAGCAAATAACACACACTATGGCCTTGCAGCAGG

GGTGTTCACAAAAAACATTAACACAGCTAACACTTTGACTAGAGCATTGCGAGTTGGAACAGTGTGGATTAACTGCTTTGATACATTTGATGCAGCAATT

CCCTTTGGAGGGTACAAAATGAGTGGCCAAGGAAGAGAAAAAGGAGAATACAGTCTCAAGAACTACTTGCAAGTGAAGGCTGTTGTTACATCCTTGAAGA

ACCCTGCTTGGCTTTGA

>Glyma.02G034000.1 CDS

ATGGCTTCTTCATTGAGGATCTCAAGGCTCCTCTCTCGTTCTTTTCTTTCTGCTTCCACTACTACTCCTTTGTTTTCTAGAGGCGGAAGTGGTGCTCTTG

GTGCAGGGCTCAGTAAATTCAGCACTGCTGCTGCCATTGAAGAACCAATTAAACCACCACTTAAAGTTGAACACACCCAGCTCTTAATTGATGGAAAATT

TGTTGATGCTGCTACTGGCAAAACTTTTCCAACGTTGGACCCCAGGACAGGAGATGTGATTTCTCATGTTGCTGAGGGTGACCATGAAGATGTTGATCGA

GCAGTTGCAGCTGCTCGCAAAGCCTTCGACCGCGGACCTTGGCCAAAGATGACAGCCTATGAAAGACAAAGGATATTGCTGCGCGCTGCTGATCTGTTTG

AAAAGCACAATGATGACCTTGCTGCACTCGAGACTTGGGATAATGGGAAGCCATATGAACAGTCTGCTCAAATCGAAATTCCAATGCTGGTTCGCCTATT

TCGATACTATGCTGGTTGGGCAGATAAGATTCATGGTCTCACAGTTCCAGCTGATGGGCCATATCATGTGCAAACATTGCATGAACCCATTGGTGTTGCC

GGTCAGATCATTCCGTGGAACTTCCCTCTTGTCATGTTTGCCTGGAAGGTTGGGCCAGCATTGGCCTGTGGTAACACCATTGTTCTCAAAACAGCTGAGC

AGACTCCATTATCTGCTTTGTATGCATCAAAGTTGCTTCATGAGGCTGGACTGCCTCCTGGTGTTCTGAATATAATATCTGGCTTTGGTCCAACTGCCGG

TGCTGCTATTGCTAGTCACATGGACATAGATAAGCTTGCTTTCACTGGTTCAACCGAAACTGGAAAAATTGTCCTTGAACTGGCGGCTAGAAGTAACCTT

AAGCCTGTAACTTTGGAGCTTGGTGGGAAATCCCCATTCATTGTATGCGAAGATGCTGATGTAGATGAGGCTGTTGAGTTAGCACACTTTGCATTATTCT

TTAATCAGGGACAATGCTGCTGTGCCGGGTCTAGAACATTTGTACACGAACGTGTGTACGATGAATTTATTGAGAAAGCAAAGGCACGCGCTTTGAAGCG

TGCTGTTGGTGATCCATTCAAGGGGGGAATAGAGCAAGGTCCTCAGATTGATTCAGAACAATTTCAGAAAATACTGAAGTATATTAGATCCGGTGTTGAA

AGTGGAGCTACTCTTGAAACCGGAGGAGATAGATTTGGCAACTCGGGCTTCTATATTCAGCCTACTGTCTTCTCAAATGTTAAGGATGACATGCTGATTG

CGAAGGAAGAGATCTTTGGCCCAGTGCAAAGCATATTAAAATTCAAGGACCTTGATGATGTGATTCAAAGAGCAAATAACACACACTATGGCCTTGCAGC

AGGGGTGTTCACAAAAAACATTAACACAGCTAATACTTTGACTAGAGCATTGAGAGCTGGAACAGTGTGGGTGAACTGCTTTGATACATTTGATGCAGCA

ATTCCCTTTGGAGGGTACAAAATGAGTGGTCAAGGAAGAGAAAAAGGAGAATACAGTCTCAAGAATTACTTGCAAGTGAAGGCTGTTGTTACATCCTTGA

AGAACCCAGCTTGGCTTTGA

>Glyma.06G183900.1 CDS

ATGGCAACTCGAAGACTTTCATTGCTCCTCTCTCGCTCTCTATCTTCCACTTCTTTTCAAGCTGCTTCTCTACTTCACTCACTAGGGAGAAACTCTGGCA

AGTGGGGAAACTTTAACAGATTCAGCACTGCAGCAGCAGTTGAGGATTTAATCACTCCACAAGTTCCAATAACATACACTAAGCATCTGATAAATGGACA

ATTTGTGGATGCTGCTTCAGGGAAAACTTTTCCAACATATGACCCACGCACAGGAGAAGTGATTGCTCAAGTTGCTGAAGGTGATGCAGAAGATATCAAC

CGTGCAGTATCGGCAGCTCGCAAGGCCTTTGATGAAGGACCTTGGCCAAAATTGACTGCTTATGAAAGATGCAAGATAATACTGCGCTTTGCTGATTTGG

TTGAGAAACACGGTGATGAACTTGCAGCTCTGGAGACATGGAACAATGGAAAGCCTTATGAACAGTCTGCCACTGCTGAATTACCAACGTTTGTGCGTTT

GTTTCGTTACTATGCTGGTTGGGCAGATAAAATCCATGGTCTGACAGTGCCTGCTGATGGAAATTATCATGTGGAGACATTGCATGAACCAATTGGTGTT

GCAGGACAAATCATACCTTGGAACTTTCCTCTCCTCATGTTTGCTTGGAAAGTTGGACCAGCTCTTGCATGTGGAAATACTGTCATCCTTAAGACTGCTG

AACAAACACCTTTAACAGCTCTCTATGTAGCAAAACTTTTTCATGAGGCTGGTCTTCCACCAGGTGTTTTGAATGTAGTTTCTGGCTATGGTCCAACTGC

TGGTGCAGCTCTAGCAAGTCATATGGATGTTGACAAGCTAGCATTCACTGGATCTACCGAGACTGGAAAAGTTGTGCTTGGATTGGCTGCGCAAAGCAAT

TTGAAGCCTGTGACATTGGAACTTGGAGGGAAATCACCTTTCATAGTGTGTGAGGATGCTGATGTTGACCAGGCTGTAGAACTTGCGCACTTTGCTTTGT

TCTTTAATCAGGGGCAATGTTGCTGTGCTGGGTCTCGTACCTTTGTACATGAGCATATCTATGATGAGTTCTTGGAGAAAGCAAAGGCTCGTGCTTTGAA

ACGTGTTGTTGGGGATCCCTTCAAAAAGGGTGTCGAACAAGGACCTCAGATTGATGTGGAACAATTCCAGAAGGTCCTTAGGTACATAAAGTCCGGTATT

GAAAGCAAGGCCACCCTTGAATGTGGGGGTGACCAAATTGGCTCAAAGGGCTTCTTTGTCCAGCCCACTGTCTTCTCAAATGTTCAGGATGACATGTTGA

TAGCAAAAGATGAAATATTTGGCCCTGTACAAACCATCTTGAAGTTCAAGGACATCGACGAAGTAATTCGAAGGTCAAACGCAACGCACTATGGTCTAGC

TGCAGGAGTTTTCACTAAGAATGTGCACACAGCCAACACTTTGATGCGAGCACTGAGGGTTGGAACAGTATGGATCAATTGCTTTGATGTTTTTGATGCT

GCAATTCCCTTTGGAGGGTACAAAATGAGCGGCATTGGCAGGGAGAAGGGTATCTACAGTCTCAACAATTACCTGCAAGTGAAAGCTGTGGTTTCACCAG

TGAAGAAACCTGCATGGTTGTGA

>Glyma.08G288000.1 CDS

ATGGCTTCTTCACTCAGAATCTCAAGACTTATCTCTCGCTCCTTTTCTTCTACTTCTTTCTTTTCACGAGGTGGGAATGGTTTCCTTGGTTCAAGACAAA

GTAAATTCAGCACTTCTGCTGCCATTGAGGAAGAACCCATTAAACCATCAATACAAGTAGAACACACCCAACTCTTAATTGATGGAAAATTTGTAGATGC

TGCTTCTGGTAAAACTTTTCAAACTTTAGATCCTAGGACAGGTGAAGTGATTGCTCATGTTGCTGAGGGTCACTCTGAAGATGTTGATCGAGCAGTTTCA

GCTGCACGAAAAGCATTTGATCATGGACCATGGCCTAAGATGACAGCTTATGAACGGCAAAGGATCTTGTTGCGTGTGGCCGATCTGATCGAGAAGCATA

ATGATGAGCTTGCAGCACTTGAGACTTGGGATAATGGAAAGCCTTATGAACAAGCTGCTAAGATTGAAGTTCCAATGCTTGTTCGCCTGATTCGATACTA

TGCTGGTTGGGCAGATAAGATTCATGGTCTAACAGTTCCAGCTGATGGACCTTATCATGTGCAAACATTGCACGAACCGATTGGTGTTGCCGGTCAGATC

ATTCCATGGAACTTCCCTCTTCTCATGTTTGCCTGGAAGGTTGGACCGGCATTGGCCTGTGGCAACACCATTGTTCTCAAAACAGCTGAGCAGACACCAT

TATCTGCTTTATATGCAGCAAAACTATTTCATGAGGCTGGACTTCCTGCTGGTGTTTTGAATGTAGTTTCCGGCTTTGGTCCAACTGCTGGTGCTGCTCT

TGCTAGTCACATGGAGGTTGATAAGCTTGCTTTTACCGGTTCCACTGATACTGGGAAAGTTGTCCTTGAACTGGCGGCTAAAAGCAATCTTAAGCCTGTT

ACTTTGGAGCTTGGTGGGAAATCTCCCTTTATAGTGTGTGAAGACGCTGATGTAGATCAGGCTGTTGAGCTCGCACACTTTGCCTTATTCTTTAATCAGG

GACAATGTTGCTGTGCTGGGTCTCGAACATTTGTACATGAAAATGTATACGAGGAGTTTGTTCAGAAAGCAAAGGCTCGTGCTTTGAGACGTGTTGTTGG

TGATCCATTCAAGGGGGGAATAGAGCAAGGTCCTCAGATTGATTCAGACCAATTTGAGAAAATACTGAGGTACATCAGATCCGGTGTTGAAAGCGGGGCA

ACCCTTGAAACAGGAGGAGACAAGCTTGGCAACAAGGGCTTCTACATTCAACCTACAGTCTTCTCAAATGTTAAGGATGGCATGCTGATTGCAAAGGATG

AGATCTTTGGTCCAGTCCAATCCATATTAAAATTCAAGGACCTTGGTGAGGTAGTTCAAAGAGCGAACAACACACGTTACGGGCTTGCGGCAGGAGTGTT

CACAAAGAACATGGACACTGCAAACACTTTGACGCGGGCACTGAGAGTTGGAACAGTTTGGATAAACTGCTTTGACACATTTGATGCTGCAATTCCCTTT

GGAGGGTACAAAATGAGTGGTCAAGGCAGAGAAAAAGGAGAGTATAGTCTCAAAAACTACTTGCAAGTGAAGGCTGTCGTTAATCCCTTGAAGAACCCAG

CATGGCTTTGA

>Glyma.13G170600.1 CDS

ATGCAGGTCATGGCCTCTAGAATTCTTTCAACTCTACATTATGTGTGTTCTTCTTCTGCCTCAGCCACAAAACGATGTTTAGGCCTATATAGTCATTGGC

AGAGAAGTATAAGTGGAATTGCTGCGTCTGTTGTTGCTGATGTGGAGCCTAGCATAGCACCAGTGCAAATTGATCAAAGTCAGCTTCTAATTGATGGGAA

ATTTGTTGATGCAGCTTCTGGAAAAACTTTTCCAACTTTTGATCCTAGGACTGGAGATGTAATTGCCAATGTTGCTGAAGGAGATGCTGAAGATGTAAAT

CGCGCTGTCCATGCTGCTCGCAAGGCTTTTGATGAGGGACCATGGCCAAAGATGACAGCTTATGAAAGATCACGTATAATTTTGCGCTTTGCTGATTTAT

TGGAGAAACACAATGATGAAGTTGCAGCCATAGAAACATGGGATAGTGGCAAGACTTATGAACAGGCTGCAAATGTTGAAATACCCATGGTAGTACGATT

GTTCCGATATTATGCAGGTTGGGCGGATAAAATTCACGGTCTAACAGTTCCAGCTGATGGACCATACCATGTCCAAACTTTGCATGAACCAATTGGTGTA

GCAGGGCAGATTGTTCCTTGGAATTTCCCACTTCTTATTTTTTCATGGAAGGTTGCACCTGCATTAGCTTGTGGTAATACAGTTGTAATGAAAACTGCAG

AGCAGACACCCCTTTCAGCCCTTTATGTGTCAAAGCTATTTCTTGAGGCAGGACTTCCTCCTGGAGTTCTGAATGTTATCTCTGGCTTTGGTCCTACTGC

TGGTGCAGCACTGTGTAGTCATATGGACGTTGACAAGCTTGCTTTCACAGGATCCACTAGCACTGGTAAACGTGTACTTGAACTGTCTGCACACAGCAAC

CTGAAGCCAGTAACTCTGGAGCTGGGTGGAAAATCTCCATTCATTGTGTGCAAGGATGCTGATGTTGATGCAGCCGTTGAGGCTTCACATTTTGCTTTAT

TTTTTAATCAGGGGCAATGTTGCTGTGCTGGTTCTCGCACATTTGTCCATGAAAGCATATATGGTGAATTTGTTGAAAAGGCTAAAGCCCGTGCTCTTAA

ACGAGTAGTTGGAGACCCTTTCAAAAATGGGGTTGAGCAAGGTCCTCAGATTGATTCTGTGCAATTCGAGAAGATCATGAAATACATAAGATCAGGCGTT

GAAAGTGGTGCTCAACTGGAATCTGGTGGTCAAAGAATTGGCTCCAAAGGCTACTACATCCAACCCACTGTTTTCTCAAATGTTCAGGACAACATGTTGA

TAGCAAAAGATGAGATATTTGGTCCAGTACAATCTATCTTGAAATTCAAGGATCTAGAGGAAGTGATAAGAAGAGCCAATGCAACTTCCTATGGCCTGGC

TGCTGGAGTTTTTACTAAAAATATGGACACTGCCAACACCTTGATGCGAGCATTGCAAGCTGGAACGGTATGGATCAACTGCTATGATGTCTTTGATGCA

GCAATACCTTTTGGTGGATATAAGATGAGTGGTCAAGGGAGAGTAAGGGGAATCTACAGCCTTAGAAGCTACCTTCAAGTTAAAGCTGTTGTCACTGCTT

TGAAGAATCCCGCATGGCTGTAG

>Glyma.17G091000.1 CDS

ATGTTGCTGAAGGGGATGTTGAAGATATCAACCCGTGCAGTATCAGCAGCTCGCAAGGCCTTTGATGAGGGACCTTGGCCTAAAATGACTGCATATGAAA

GAAGTCGGATATTGCTGCGCTTTGCTGATTTGGTTGAGAAACACAGCGATGAGCTTGCTGCTCTCGAGACATGGAATAACGGAAAGACTTATGAACAGGC

TGCCAAGACTGAATTACCAATGTTCGTGCGTTTATTTCACTATTATGCTGGTTGGGCAGACAAAATTCATGGGTTGACAGTGCCAGCTGATGGAGATTAT

CATGTGCAGACATTGCATGAACCAATTGGTGTAGCAGGACAAATTATACCTTGGAACTTTCCTCTAGTTATGTTTGCTTGGAAAGTTGGACCAGCTCTAG

CATGTGGTAATACCATTGTTCTGAAGACTGCTGAGCAAACTCCACTGACAGCTCTCTTTGTAGCAAAACTATTTCATGAGGCTGGTCTTCCTGATGGTGT

TCTGAATGTAGTTTCTGGGTACGGTCCAACTGCTGGCGCAGCTCTTGCAAGTCATATGGATGTGGACAAGCTAGCATTTACTGGCTCAACAGATACCGGA

AAAGTTGTACTCGAACTGGCTGCAAGAAGCAATCTTAAGCCAGTGACATTGGAGCTTGGAGGGAAATCGCCTTTCATAATATGTGAGGATGCTGATGTTG

ACAAGGCTGTTGAACTTGCACACTTTGCTCTGTTCTTTAATCAGGGGCAATGTTGCTGTGCCGGCTCACGTACCTTTGTACATGAGCGTGTCTATGATGA

GTTCTTGGAGAAATCAAAGAAACGGGCTTTGAGACGTGTTGTTGGTGATCCATTCAAGAAAGGTGTTGAACAAGGTCCTCAGATTGACGTGGAACAATTT

GAGAAAGTCCTTAGGTACATAAGGTCTGGAATTGAAAGCCATGCCACCCTTGAATGTGGGGGTGATAGATTGGGCTCAAAAGGCTTCTTTGTCCAGCCAA

CTGTCTTCTCAAATGTTCAGGATGACATGTTGATAGCACAAGACGAAATCTTTGGCCCAGTTCAGTCCATCTTGAAATTCAAGGACATTGATGAAGTAAT

ACGACGGGCAAATAAAACACGCTATGGTCTAGCAGCAGGTGTGTTTACTAAGAACGTGAGCACAGCCAACACATTGATGCGGGCACTGAGAGCTGGAACT

GTGTGGATTAATTGCTTTGATGTTTTTGATGCTGCAATTCCTTTTGGTGGATACAAGATGAGTGGTATTGGCAGGGAGAAAGGAATCTACAGTCTTCACA

ACTACCTGCAGGTGAAAGCTGTTGTCTCACCAGTGAAGAATCCTGCGTGGCTATAA

>Glyma.18G137300.1 CDS

ATGGCTTCTTCACTCAGAATTTCAAGACTCATCTCTCGCTCCTTTTCTTCTACCTCCTTCTTTTCACGAGGTGGGAATGGTTTCCTTGGTTCAAGACACT

GCAAATACAGCACTTCTTCTGCCATTGAAGAAGAACCCGTTAAACCATCAGTGCAAGTAGAACACACCCAACTCTTAATTGATGGAAAATTTGTAGATGC

TGCTTCTGGTAAAACTTTTCCAACTTTAGATCCTAGGACAGGTGAAGTAATTGCTCATGTTGCTGAGGGTCACTCTGAGGATGTTGATCGAGCAGTTGCA

GCTGCACGAAAAGCATTTGATCATGGACCATGGCCTAAGATGACGGCTTATGAACGGCAAAGGATCTTGTTGCGCGCAGCTGATCTGCTTGAGAAGCATA

ATGATGAGCTTGCAGCACTTGAGACTTGGGATAATGGAAAGCCTTATGAACAAGCTGCTAAGATTGAAGTTCCAATGCTTGTTCGCCTGATTCGATACTA

TGCTGGTTGGGCAGATAAGATCCATGGTCTAACAGTTCCAGCTGATGGACCATATCATGTGCAAACATTGCATGAACCAATTGGTGTTGCCGGTCAGATC

ATTCCTTGGAACTTCCCTCTTCTCATGTTTGCCTGGAAGGTTGGACCGGCATTGGCCTGTGGCAACACCATTGTTCTCAAAACAGCTGAGCAGACACCGT

TATCTGCTTTATATGCAGCAAAACTATTTCATGAGGCTGGACTTCCTGCTGGTGTTTTAAATGTAGTATCTGGCTTTGGTCCAACTGCTGGTGCTGCTCT

TGCTAGCCACATGGAAGTTGACAAGCTTGCTTTTACTGGTTCCACTGATACTGGGAAAGTTGTCCTTGAACTGGCGGCTAAAAGCAATCTTAAGCCTGTT

ACTTTGGAGCTTGGTGGGAAATCCCCCTTTATTGTGTGTGAAGATGCTGATGTAGATCAGGCTGTTGAGCTAGCCCATTTTGCTTTATTCTTTAATCAGG

GACAATGTTGCTGTGCTGGGTCTCGAACATTTGTACATGAAAGTGTATACGATGAGTTTGTTGAGAAAGCAAAGGCTCGTGCTTTGAAGCGTGTTGTTGG

TGATCCATTCAAGGGGGGAATAGAGCAAGGTCCTCAGATTGATTCAGATCAATTTGAGAAAATACTGAGGTACATCAGATCTGGTGTTGAAAGTGGGGCA

ACCCTTGAAACCGGAGGAGACAAGCTTGGCAACAAGGGCTTCTACATTCAACCTACAGTCTTCTCAAATGTTAAGGATGGCATGCTGATTGCAAGGGATG

AGATCTTTGGTCCAGTCCAATCCATATTAAAATTCAAGGATCTTGGTGAGGTAGTTCAAAGAGCAAATAACACACGTTACGGGCTTGCGGCCGGAGTGTT

CACAACGAACATGGACACTGCCTACACTTTGACGCGGGCACTGAGAGTTGGAACAGTGTGGATAAACTGCTTTGACACATTTGATGCTGCAATTCCCTTT

GGAGGGTACAAGATGAGTGGTCAAGGCAGAGAAAAAGGAGAGTATAGTCTCAAAAACTACTTGCAAGTGAAGGCTGTTGTTAATCCCCTGAAAAACCCAG

CATGGCTTTGA

>Glyma.19G010800.1 CDS

ATGACTAGTATTCGACAATGTGAAAGTGATGAATCAAGTTTAAAATCTGCATTTGAAGAAGTATCAACATTTAGCCTATGTTCTCACTGGCATAGAAGTA

TAAGTGGAATTGGTGCTTCTGCTGCTGCTGATGTGGAGCCTAGCATAGCACCAGTGCAAATTGATCATAGTCAGCTTCTAATTGATGGACAGTTTGTTGA

TGCAGCTTCTGGAAAAACTTTTCCAACCTTTGATCCTAGAACTGGAGATGTAATTGCCAATGTTGCTGAAGGTGACACTGAAGACGTAAATCGTGCTGTT

CGTGCTGCTCGCAAGGCTTTTGATGAGGGACCATGGCCAAAGATGACAGCTTATGAAAGATCGCGTATAATTTTGCGCTTTGCTGATTTATTGGAGAAAC

ACAATGATGAAGTTGCCGCAATTGAAACATGGGATAGTGGGAAGACTTATGAACAGGCTGCTAAGGTTGAAATACCTATGGTGGTACGCTTATTCCGATA

TTATGCAGGTTGGGTGGATAAAATTCATGGTCTAACAGTTCCGGCTGATGGACCATACCATGTCCAAACTTTGCATGAACCAATTGGTGTAGCAGGGCAG

ATTGTTCCTTGGAATTTCCCACTTCTTATTTTTTCATGGATGGCTGCACCTGCATTAGCTTGTGGTAATACAGTTGTAATAAAAACTTCAGAGCAGGCAC

CACTTTCTGCCCTTTATGTGTCAAAGCCATTTCTTGAGGCAGGACTTCCTCCTGGTGTTCTGAATGTTATCACTGGCTTTGGTGCTACTGCTGGTGCATC

GCTGTGTAGTCATATGGACGTTGATAAGCTTGCTTTCACAGGATCTACTAGCACTGGTAAACGTCAATCTGAAGTAACTCTGGAGCTGGGTGGAAAATCT

CCATTCATTGTGTGCGAGGATGCTGATGTTGATGCAGCTGTTGAGGCTGCACATTTTGCTTTATTTTTTAATCAGGGGCAATGTTGTTGTGCTGGTTCTC

GCACATTTGTCCATGAAAGCATATATGATGAATTTGTGGAAAAGGCTAAAGCTCGTGCTCTTAAACGTGTAGTTGGAGACCCTTTCAAAAATGGAGTTGA

GCAAGGTCCTCAGATTGATTCTGCGCAATTCGAGAAGATCATGAAGTACATAAGATCAGGCGTTGAAAATGGTGCTACACTGGAATCTGGTGGTCAAAGA

ATTGGCTCCAAAGGCTATTACATCCAACCCACTGTTTTCTCAAATGTTCAGGACAACATGTTGATAGCAAAAGATGAGATATTTGGTCCAGTACAATCTA

TCTTGAAATTCAAGGATCTAGAGGAAGTGATAAGAAGAGCCAATGCAACTTCCTATGGCCTGGCTTCTGGAGTTTTTACTCAAAATATGGACACTGCCAA

CACCTTGATGCGCGCATTGCGAGTTGGAACAGTATGGATCAACTGCTATGATGTCTTTGATGCAGCAATACCTTTTGGTGGATATAAGATGAGTGGACAA

GGGAGAGTAAGGGGAATCTACAGCCTTAGAAGCTACCTTCAAGTTAAAGCTGTTGTCACTGCTTTGAAGAATCCCGCATGGTTGTAG

>Glyma.05G231800.1 CDS

ATGACTTTCAATAATGGCGATGCAGCTGCTGCCTCCCTCAACAAGGTCCCCACGGTCAACTTCACCAAGCTCTTCATTGACGGACACTTCGTTCACTCTG

TGTCAGGAAAGACATTTGAGACAATAGATCCAAGAACAGGAGATGTTATAGCGAGGATCAGCGAGGGAGATAAAGAAGACATTGACATTGCTGTTAAAGC

AGCACGTCATGCATTTGACAATGGTCCATGGCCTCGCCTTCCCGGTTCTGAGAGAGGAAGAATTTTGCTGAAATGGGCAGAGCTAATAGAGGAAAATGCA

GAAGAACTTGCGGCACTAGATGCCATTGATGCGGGGAAGTTGTACCATATGTGTAGGAATTTGGAAGTTCCAGCAGCAGCAAACACTCTTCGTTACTATG

CAGGTGCTGCCGATAAGATTCATGGCGAGGTGTTGAAAATGTCCCGAGACTTCCATGCCTATACATTGCTTGAACCACTTGGTGTTGTGGGACACATTAC

TCCCTGGAATTTCCCCAATACCATGTTCTACATCAAGGTTGCTCCTTCTTTAGCTGCTGGCTGCACCATGGTCCTCAAGCCCGCCGAGCAAACACCCCTC

TCTGCTTTGTTTAATGCTCATCTTGCTAAATTGGCTGGAATCCCAGATGGAGTGATCAATGTAGTGCCTGGATTTGGCCCAACTGCTGGTGCTGCATTAA

GCTCACACATGGATGTTGATAAGGTTAGCTTTACTGGTTCAACACAAACAGGCCGTGAGATAATGCAGGCTGCAGCTAAGAGTAACTTGAAACAAGTTTC

ACTTGAATTAGGAGGCAAGTCACCCCTCATAATTTTTGATGATGCTGATATAGACAAAGCTGCTGAGCTAGCTCTACTAGGCATCCTATATAACAAGGGA

GAAGTTTGTGTTGCAAGTTCCCGTGTGCTTGTTCAAGAAGGGATCTATGATGAATTTGAGAAAAAACTGGTGGAGAAGGCAAAAGCTTGGGTCGTTGGGG

ATCCCTTTGATCCTAAAGTTCAACAAGGCCCTCAGGTTGACAAGGAACAATTTGAAAAGGTCCTTTCATATATTGAGCATGGAAAGAAAGAAGGAGCTAC

CCTTTTGACCGGGGGTAAAACAGTGGGAAACAAGGGCTACTTTATTGAGCCAACAATTTTCTCCAATATAAGGGAGGATATGCTTATAGCACAGGATGAA

ATATTTGGCCCAGTAATGGCACTGAAGAAGTTTAAGACCATTGAGGAAGCAATTAAGAGTGCTAACAATACCAAGTATGGCCTAGCAGCAGGCATAGTGA

CCAAGAATTTGGATACGGCAAACACAGTGTCAAGGTCCATTCGTGCAGGCACTATTTGGATCAACTGCTATTTTGCCTTTGGCGATGATGTTCCCTTTGG

AGGGTATAAGATGAGTGGATTTGGAAAAGATCATGGATTGGAAGCCCTTCACAAGTACCTACAAGTTAAATCTGTTGTTACTCCCCTTTACAATTCACCC

TGGCTTTGA

>Glyma.05G231900.1 CDS

ATGTTCTTGTCTTTGCATATAGTCCTAATTAATCATATTGCAACATTCCACTTACCGACACCATCTCTACGACAACCTCCTTTCTCTCTATCTCTAGCTC

GGATGAGTGCTCTCTCTAACTCCAGTAGTAGCCACGGCAATTCCTTCCTCAAGATGCCCGCCATCAAGTTTACCAAGCTCTTCATCAATGGAGATTTTGT

TGATTCCATATCAGGAAGGACATTTGAGACTATAGACCCCAGAAAAGAAGAGGTAATTGCAAGAGTTAGTGAGGGAGATAAAGAAGACATTGATATTGCT

GTTAAAGCAGCACGTCAGGCATTTGACTCGGGTCCATGGCCTCGCTTGCCAGGCTCTGAAAGGGCAAAAATTATGATGAAATGGGCAGACCTAGTTGATG

AAAATATAGAAGAACTAGCAGCATTAGATACCATTGATGCTGGAAAGCTATACTATATTAATAAGGTAGCGGAAATTCCTTCAGCTACAAATGCGTTACG

GTACTATGCAGGTGCTGCTGATAAAATTCACGGTGACGTATTAAAAATGAACGGGGATTTCCATGCATATACACTTTTGGAACCAATTGGTGTTGTGGGA

CACATAATTCCATGGAATGCCCCCAGCCTCTCATTTTTCATCAAGGTTAGCCCTTCCTTAGCTGCAGGCTGTACTATGGTCCTCAAACCTGCTGAACAAA

CACCCCTCTCTGCTTTGTTTTATGCTCATCTAGCTAAGTTGGCTGGAATCCCAGATGGTGTGCTTAATATAGTACCTGGATTTGGCCCAACTGCTGGCGC

AGCAATAAGCTCACACATGGACATAGATGCGGTCAGTTTTACTGGTTCAATTGAAGTAGGGCGTGAAGTGTTGCAGGCTGCAGCTTGGAGCAATTTAAAA

CCGGTTTCACTTGAATTAGGAGGCAAGTCTCCTCTCATTATTTTCAATGATGCGGATATAGACAAAGCTTCCGAGCTTGCTCTCTTTGGCATCATGTCTA

ACAAGGGAGAAATTTGTGTGGCAGGTTCTCGTGTGTTTGTCCAGGAAGAGATCTATGATGAATTTGAGAAGAAGTTGGTGGAGAAGGCAAAATCTTGGGT

CGTTGGGGATCCTTTTGATCCCAAATCGCTGCAAGGGCCTCAGGCTGACAGGAACCAATTGGAGAAAATACTTTCATATATTGAGCACGGAAAGAGAGAA

GGAGCCACCCTTTTGACAGGAGGTAATACAGTGGGCAACAAAGGTTACTACATAGAACCTACAATTTTCTCCAATGTAAAGGAGGACATGCTAATAGCAC

GGGATGAAATATTTGGCCCTGTACTAGCGCTGATGAAGTTTAAGACCATGGAGGAAGCAATTAAAAGTGCTAACAATACCAAGTATGGCCTAGCAGCAGG

AATTGTAACCAAGAATTTGGATACTGCAAACACTATGTCAAGGTCCATTCGTGCAGGCATTGTTTGGATCAACTGCTATTTTACCGTAGGGAGTGATGTT

CCTTTTGGAGGGTATAAGATGAGCGGATTTGGAAGAGATTTGGGATTACAGGCCCTTCACAAGTACTTACAAGTTAAATCTGTTGTAACACCTATTCATA

ATTCTCCTTGGCTTTGA

>Glyma.07G087400.1 CDS

ATGGAAAATCTCAGCAATGGTCACTTAGAATCCTTTGTCAAGATTCCAACAATAAAGTTTACCAAGCTTTTCATCAATGGAGAATTCCTAGATTCTGTTT

CAGGAAAAACGTTTGAGACCGTTGACCCAAGAACAGAGGAAGTGATTGCTGAAATAGCAGAGGCTAACAAAGAAGATGTTGATATTGCTGTGAAGGCCGC

ACGTGAAGCATTTGATTGTGGTCCATGGCCACGCATGCCCGGTGCTGAAAGAGCAAAGATTATGCTGAAATGGTCAGAGCTAATTGAACAGAACGCAGAA

GAAATAGCAGCATTGGACACCATTGATGGGGGAAAGCTATTCAGTTGGTGTAAGGCTGTGGATGTTCCTGAAGCATCCAACATCCTACGTTACTATGCCG

GCGCTGCTGATAAAATTCATGGAGATGTGTTCAAAACATCTCGTGACCTCCACTTGTATTCTCTGATGGAACCTGTTGGTGTTGTTGGACACATTATCCC

TTGGAATTTCCCTACCGTCATGTTCTTCGCCAAGGTTGCCCCGGCCTTGGCTGCTGGCTGCACCATGGTCATCAAGCCTGCTGAGCAAACACCTCTCTCA

TCACTCTTTTATGCTCATCTTGCTAGGCTGGCTGGTATCCCAGATGGAGTGCTGAATGTAGTACCCGGATTTGGCTCAATTGCAGGGGCTGCAATAAGTT

CACATATGGACATTGATGCGGTCAGTTTTACAGGTTCAACAGAAACAGGTCGTAAAATAATGCAGGCTGCGGCCTTGAGCAATTTGAAACCAGTTTCACT

CGAATTAGGAGGAAAGTCACCAGTTTTGATTTTTGATGATGCTGATGTAGACAAAGCTGTTGACCTTGCTCTCTTTGGCATCCTACATAACAAGGGAGAA

ATCTGTGTTGCATTCTCCAGAGTTTATGTCCAGGAAGGGATTTATGATGAATTTGAGAAGAAGGTAGTGGAGAAGGCTAAAACTTGGGTAGTGGGAGACC

CCTTTGATCCTAAAGTTCAGCAAGGACCCCAAACTAGTAAGGCTCAATATGATAAAATTATTTCCTATATTGAGCATGGAAAGAGTGAAGGAGCCACACT

GTTGACTGGGGGTAAGCCAGCGGGCAACAAGGGATACTACATTGAGCCTACCATTTTTGTCAATGTTAAGGAGGACATGCTAATTGCACAAGAGGAAATA

TTTGGACCTGTGATGACACTTTCCAAGTTCAAGACCATTGAGGATGCAATTAAGAAAGCCAACAATTCCAAATATGGCCTAGCAGCAGGGATTGTGACCA

AGAACTTGGATATTGCAAACACTGTATCAAGGTCCATCCGTGCAGGCATCATTTGGATCAATTGCTTCTTTGCCTTTGATATTGACTGCCCTTTTGGAGG

GTATAAGATGAGTGGATTTGGAAGAGATTATGGACTGGAAGCACTTCATAAGTTTCTCAAAGTTAAGTCTGTTGCAACTCCTATTTACGATTCTCCTTGG

CTTTGA

>Glyma.07G087500.1 CDS

ATGGCTGCTCTCTCCAACGGCCACGACGCTTCCTTCTTCAAGATGCCCTCCATCAAGTTCACCAAGCTCTTCATCAATGGAGAATTTGTTGATTCCCTTT

CAGGGAAGGAGTTTGAAACAATAGATCCCAGGACAGGAGAAGTGATTACAAGAATCGCAGAAGGAGCAAAAGAAGACATTGATGTTGCTGTCAAAGCGGC

ACGTGACGCTTTCGACTATGGTCCATGGCCCCGTATGCCCGGTGCTGAAAGAGCAAAAATCATGATGAAATGGGCAGACTTAATTGATCAGAACATCGAG

GAAATAGCAGCATTGGATGCCATTGATGCAGGGAAGTTGTACCATTGGTGCAAGGCTGTTGACATTCCTGCTGCAGCAAATACTATTCGTTACTATGCCG

GTGCTGCGGATAAAATTCATGGGGAGGTCTTAAAAGCGTCTAGGGAGTTCCATGCATATACTTTGTTAGAACCAATTGGTGTTGTAGGACACATTATTCC

GTGGAATTTCCCCAGCACCATGTTTGTTGCCAAGGTGAGTCCTTCCTTGGCTGCTGGTTGCACAATGGTCCTCAAGCCTGCTGAACAAACACCTCTCTCG

GCCTTGTTTTATGCTCATCTTGCTAAGCTGGCTGGAATTCCAGATGGAGTGCTTAATGTAGTACCCGGATTTGGCCAAACTGCAGGTGCTGCAATAAGCT

CACACATGGACATTGATAAGGTAAGCTTTACGGGTTCAACAGAAGTGGGGCGTGAAGTAATGCGTGCTGCAGCTAATAGTAATTTGAAACCAGTTTCACT

TGAGCTAGGAGGCAAGTCACCCGTCATAGTTTTTGATGATGCTGATGTAGATAAAGCTGCTGGACTTGCTCTCATGGGCATCCTATTTAATAAGGGAGAA

ATTTGTGTTGCAGGCTCCCGTGTGTTGGTTCAGGAAGGAATCTATGATGAATTTGAGAAGAAATTGGTGGAAAAAGCAAATGCTTGGGTGGTTGGTGATC

CTTTTGATCCTAAAGTTCAGCAAGGGCCTCAGGTTGACAAGAAGCAATTTGAAAAGATTCTTTCCTATATCGAGCATGGAAAGAAAGAAGGTGCAACCCT

TTTGACAGGGGGCAAAAGAGTGGGCAACAAGGGTTACTACATTGAGCCTACAATTTTCTCTAATGTTAAGGAGGACATGCTTATAGTACAAGATGAAATA

TTTGGCCCTGTGATGGCTTTGATGAAGTTTAAGACTATTGAAGATGCAATTAAGATTGCCAACAATACAAGGTATGGCCTAGCATCAGGCATTGTGACAA

AGAGTTTGGACACAGCCAACACTGTGTCAAGGTCCATTCGTGCAGGCATTGTTTGGATCAACTGTTATTTTGCCTTTGGGGATGACATTCCTTATGGGGG

GTACAAGATGAGTGGATTTGGAAGAGATTTTGGAATGGAAGCCCTACACAAGTATCTTCAAGTTAAATCTGTTGTAACTCCCATTTACAATTCTCCCTGG

CTTTGA

>Glyma.08G039200.1 CDS

ATGACTAGCCTTACTAATGGCGATGCTGGCTCCCTCAACAAGGTCCCCACGATCAAGTTCACCAAGCTCTTCATTAATGGAGACTTCGTTGACTCTCTGT

CAGGAAAGACATTTGAGACAATAGATCCAAGAACAGGAGATGTTATAGCGAGGATAAGCGAGGGAGATAAAGAAGACATTGACATTGCTGTTAAAGCAGC

ACGTCATGCATTTGACAACGGTCCATGGCCTCGCCTTCCCGGCTCTGAGAGAGCAAGAATATTGCTGAAATGGGCAGAAATAATTGAGGAAAACGCAGAA

GAACTTGCAGCACTAGATGCGATTGATGCGGGGAAGTTGTACCATATGTGTAGGAATGTGGAAGTTCCAGCAGCGGCAAACACTCTTCGTTACTATGCTG

GTGCAGCCGATAAGATTCATGGAGAGGTGTTGAAAATGTCTCGAGAGTTCCATGCCTATACATTACTTGAACCACTTGGTGTTGTGGGACACATTACTCC

CTGGAATTTCCCCAATACCATGTTCTACATCAAAGTCGCTCCTTCTTTAGCTGCTGGCTGCACCATGGTCCTCAAGCCTGCTGAGCAAACACCCCTCTCT

GCTTTGTTTTCTGCTCATCTTGCTAAATTGGCTGGAATCCCAGATGGAGTGATCAATGTAGTGCCTGGATTTGGCCCAACTGCTGGTGCTGCATTAAGCT

CACACATGGATGTTGATAAGGTCAGCTTTACCGGTTCAACACAAACAGGCCGTGTGATAATGCAGGCTGCAGCAAAGAGTAACTTGAAACAAGTTTCACT

TGAATTAGGAGGCAAGTCACCCCTCATAATTTTTGATGATGCAGATATAGACAAAGCTACTGAGCTAGCTCTACTAGGCATCCTATATAACAAGGGAGAA

GTTTGTGTTGCAAGTTCCCGTGTGTTTGTTCAAGAAGGGATCTATGATGAATTTGAGAAAAAACTGGTGGAGAAGGCAAAAGCTTGGGTCGTTGGGGATC

CCTTTGATCCTAAAGTTCAACAAGGCCCTCAGGTTGACAAGGAACAATTTGAAAAAGTTCTTTCATATATTGAGCATGGAAAGAAAGAAGGAGCTACCCT

TTTGACCGGGGGTAAAACAGTGGGGAACAAGGGCTACTTTATTGAGCCGACAATTTTCTCCAATATAAGAGAGGATATGCTTATAGCACAGGATGAAATA

TTTGGCCCAGTGATGGCACTGAAGAAGTTTAAGACCACTGAGGAAGCAATTAAGAGTGCTAACAATACCAAGTATGGCCTAGCAGCAGGCATAGTGACCA

AGAATTTGGATACAGCAAACACAGTGTCAAGGTCCATTCGGGCAGGCACTATATGGATCAACTGCTATTTTGCCTTTGGCGATGATGTTCCCTTTGGAGG

ATATAAGATGAGTGGATTTGGAAAAGATCATGGATTGGAAGCCCTTCACAAGTACCTACAAGTTAAATCTGTTGTTACTCCCCTTTACAATTCTCCCTGG

CTTTGA

>Glyma.08G039300.1 CDS

ATGAGTTCCCTCTCTAATAACTCTAGTAGTAGCCACGGCAATTCCTTCCTCCAGATGCCCCCCATCAAGTTTACCAAGCTCTTCATCAATGGAGATTTCG

TTGATTCCTTATCTGGAAGGACATTTGAGACTATAGACCCCAGAACAGAAGAGGTAATTGCAAGAGTTAGTGAGGGAGATAAAGAAGATATTGATATTGC

TGTTAAAGCAGCACGTCAGGCATTTGACTCAGGTCCATGGCCTCGCTTGCCCGCCTCTGAAAGGGCAAAAATTATGATGAAATGGGCAGACCTAATTGAT

GAAAACATAGAAGAACTAGCAGCATTAGATACCGTTGATGCTGGCAAGCTGAACTATATTAATAAGGTTGTGGAAATTCCTTCAGCTACAAATGCGTTAC

GATACTATGCAGGTGCGGCTGATAAAATTCATGGTGAAGTATTAAAAATGAATGGGGATTTTCATGCATATACACTTTTGGAACCAATTGGTGTTGTGGG

ACACATAATTCCATGGAATGCCCCCAGCCTCTCGTTTTTCATCAAGGTTAGCCCTTCCTTAGCTGCAGGCTGCACCATGGTCCTCAAACCTGCTGAACAA

ACACCCCTCTCTGCCTTGTTTTATGCTCATCTAGCTAAGTTGGCTGGAATCCCAGATGGTGTGCTTAATATAGTACCTGGATTTGGCCCAACCGCTGGTG

CAGCAATAAGCTCACACATGGACATAGATGTGGTCAGTTTTACTGGTTCAATTGAAGTAGGGCGTGAAGTGATGCAGGCTGCAGCTAGGAGCAATTTAAA

ACCAGTTTCACTTGAATTAGGAGGCAAGTCTCCTCTCATTATTTTCAATGACGCGGATATAGACAAAGCTGCCCAGCTTGCTCTCTTTGGCATCATGTCT

AACAAGGGAGAAATTTGTGTGGCAAGTTCTCGTGTGTTTGTCCAGGAAGAGATCTATGATGAATTTGAGAAGAAGTTGGTGGAGAAGGCAAAATCTTGGG

TCGTTGGGGATCCTTTTGATCCCAAATCCCTGCAAGGGCCTCAGGCTGACAGGAACCAATTGGAGAAAATACTCTCCTATATTGAACACGGAAAGAGAGA

AGGAGCTACCCTTTTGACCGGAGGTAATACAGTGGGCAACAAAGGTTACTACATAGAACCTACAATTTTCTGTAATGTAAAGGAGGACATGCTTATAGCA

CGAGATGAAATATTTGGCCCTGTACTAGCGCTGATGAAGTTTAAGACCATGGAGGAAGCAATTAAAAGTGCTAACAATACCAAGTATGGCCTAGCAGCAG

GAATTGTGACCAAGAATTTGGATACTGCAAACACTATGTCAAGGTCCATTCGTGCAGGCATTGTTTGGATCAACTGCTATTTAACCGTAGGGAGTGACGT

TCCTTTTGGAGGGTATAAGATGAGTGGATTTGGAAGAGATTTGGGATTGCAGGCCCTTCATAAGTACTTACAAGTTAAATCTGTTGTAACACCTATTCAC

AATTCTCCTTGGCTTTGA

>Glyma.09G189200.1 CDS

ATGAACTCCAATGGTTACCCTGCTTCCTCCTTCAAAATCCCCACCGTCAAGTTCACCAAGCTCTTCATCAATGGACATTTTGTTGATTCACTTTCAGGAG

GAGAATTTGAGACAATAGACCCAAGAACCGGAGAGGTGATTGCAAGGATTGCAGAGGGAACCAAAGAAGACATTGACCTTGCCGTCAAAGCGTCTCGTCT

GGCTTTCGACCATGGTCCATGGCCTCGTATGCCTGCCGTAGAAAGAGCAAGAATTATGATGAAATGGGCTGACCTAATTGACCAACACGTAGAAGAAATT

GCAGCACTGGATGCCATTGATGCTGGAAAGTTGTACCATATGCTTAAGGCTATTGAAATTCCTGCAACAGCAAATACTATACGTTATTATGCTGGTGCTG

CTGATAAAATTCATGGAGAGGTATTAAAGCCGGCACGGGAGTTCCATGCATATACTTTACTTGAACCAGTTGGTGTTGTGGGACATATCATTCCTTGGAA

TTTCCCTAGCATCATGTTTGTCTCCAAGGTTAGCCCTTGCTTGGCTGCTGGTTGCACAATGGTCCTCAAGCCTGCTGAACAAACACCTCTTTCAGCTTTG

TTTTATGCTCATCTAGCTAAACTGGCTGGAATTCCAGACGGAGTGCTCAATGTAGTCCCCGGATTTGGCGCAACTGCAGGTGCTGCAATATGCTCAGACA

TGGACATTGATAAGGTCAGTTTTACAGGTTCAACAGAAGTAGGGCGTGAAGTAATGCGTGCTGCAGCTAACAGTAATTTGAAACCAGTTTCACTTGAACT

AGGAGGCAAGTCACCATTCATAATTTTTGATGATGCAGATCTAGATAAAGCGGTTGAGCTTGCTCTCATGGCCGTAGTATATAACAAGCAACAACACATC

TTCATTAGTGACAACTATTTATTGTTGTTGTCTGGTTTTCAGGGAGAAGTTTGTGCTGCAGGCTCCCGCGTATTTGTTCAGGAAGGGATCTATGATGAAT

TTGAGAAGAGGTTGGTGGAGAAGGCAAAAGCTTGGGTTGTTGGGGATCCCTTTGATCCTAATGTTCAGCAAGGGCCTCAGGTTGACAAAAAGCAATTTGA

AAAAATTCTTTCCTACATCGAGCATGGAAAAAGAGAAGGGGCCACCCTTTTGACAGGGGGCAAAAGAGTGGGTAACAAGGGTTACTACATTGAGCCAACA

ATTTTCTCTAATGTTAAGGAGGACATGCTTATAGCACAAGATGAAATATTCGGTCCCGTGATAGCACTGATGAAGTTTAAGACAATTGAGGAAGCAATTA

AAAGTGCTAACAACAGCAGATATGGCCTAGTAGCAGGTGTTGTGACCAAGAGCTTGGACACAGCAAACACAATGTCAAGGTCAATTCGTGCAGGGGTTGT

TTGGATCAACTGTTATTTTGCCTTCGAAAATGACATTCCTTATGGAGGATGTAAGATGAGTGGATTTGGAAAAGATTCTGGATTGGAAGCCCTACACAAG

TACCTACATGTTAAATCCGTTGTAACACCCATTTACAATTCTCCTTGGCTTTGA

>Glyma.09G189300.1 CDS

ATGGCTGCTCTCTCCAACGGCCACGGCTCTTCCTTCTTCAAGATGCCCCCAATCAAGTTCACAAAGCTCTTCATCAATGGGGAATTTGTTGATTCCCTTT

CAGGGAGGGAGTTTGAAACAAGAGACCCTAGGACAGGGGAGGTGATTACAAGAATCGCAGAGGGAGCAAAAGAAGACGTTGATGTTGCTGTCAAAGCGGC

ACGTGCGGCTTTCGACTATGGTCCATGGCCCCGTATGCCCGGCGCTGAAAGAGCAAAAATTATGATGAAATGGGCAGACTTAGTTGATCAGAACATTGAG

GAAATAGCAGCATTGGATGCCATTGATGCTGGAAAGTTGTACCATTGGTGCAAGGCTGTTGATATTCCTGCTGCAGCAAGTACTATCCGTTACTATGCAG

GTGCTGCAGATAAAATTCATGGAGAGGTCTTAAAAGCTTCTAGGGAGTTCCATGCATATACTTTGCTAGAACCAATTGGTGTTGTGGGACACATTATTCC

TTGGAACTTCCCTAGCACCATGTTTGTTGCGAAGGTTAGTCCTTCCTTGGCTGCTGGTTGCACAATGGTCCTCAAGCCTGCTGAACAAACACCTCTCTCG

GCCTTGTTTTATGCTCATCTTGCTAAGCTGGCTGGAATTCCGGATGGAGTGCTTAATGTGGTACCTGGATTTGGCCAAACTGCAGGTGTTGCAATAAGCT

TACACATGGACATTGATAAGGTAAGCTTTACGGGTTCGACAGAAGTGGGGCGTGAAGTAATGCGTGCTGCAGCTAATAGTAATTTGAAACCAGTTTCACT

TGAACTAGGAGGCAAGTCACCCGTCATAGTTTTTGATGATGCTGATGTAGATAAAGCTGCTGAACTTGCTCTCCTGGGCATCCTATTTAATAAGGGAGAA

ATTTGTGTTGCGGGCTCCCGTGTGTTGGTTCAAGAAGGAATCTATGATGAATTTGAGAAGAAATTGGTGGAGAAAGCAAAAGCTTGGGTGGTTGGTGATC

CTTTTGATCCTAAAGTTCAACAAGGGCCTCAGGTTGACAAGAAGCAATTTGAAAAGATTCTTTCCTATATCGAACAAGGAAAGAAAGAAGGGGCAACTCT

TTTGACAGGGGGCAAAAGAGTGGGCAACAAGGGTTACTACATTGAGCCTACAATTTTCTCTAATGTTAAGGAGGACATGCTCATAGTACAAGATGAAATA

TTTGGCCCTGTGATGGCTTTGATGAAGTTTAAGACTATTGAGGATGCAATTAAGATTGCCAACAATACAAGATATGGCCTAGCATCAGGCATTGTGACAA

AGAGTTTGGACACAGCCAACACTGTGTCAAGGTCCATTCGTGCTGGCATTGTTTGGATCAACTGTTACTTTGCCTTTGGGAATGACATTCCTTATGGAGG

GTACAAGATGAGTGGATTTGGAAGAGATTTTGGAATGGAAGCCCTACACAAGTATCTTCAAGTTAAATCTGTTGTAACTCCCATTTACAATTCTCCCTGG

CTTTGA

>Glyma.09G189500.1 CDS

ATGGCAAATCTCAGCAATAGCCACTCAGAATCCTTTGTCAAGATTCCAACAGTAAAGTTTGCCAAGCTTTTCATCAATGGAGAATTCCTAGATTCTGTTT

CAGGAAAAACGTTTGAGACTGTTGACCCAAGAACAGAGGAAGTGATTGCTGAAATAGCAGAGGCTAACAAAGAAGATGTTGATATTGCTGTGAAGGCCGC

ACGTGAAGCATTTGATTTTGGTCCATGGCCACGCATCCCCGGTGCTGAAAGAGCAAAGATTATGCTGAAATGGTCACAGCTAATTGAACAGAACGCAGAA

GAAATAGCAGCATTGGACACCATTGATGGGGGAAAGCTTTTCAGTTGGTGTAAGGCTGTGGATGTTCCTGAAGCATCCAACATCCTACGTTACTATGCCG

GTGCTGCTGATAAAATTCATGGAGATGTGTTCAAAACATCTCGCAACCTCCACTTGTATTCTCTGATGGAACCTGTTGGTGTTGTTGGACACATTATCCC

TTGGAATTTCCCTACCGTCATGTTTTTCGCCAAGGTTGCCCCGGCCTTGGCTGCTGGCTGCACCGTGGTCATCAAGCCTTCTGAGCAAACACCTCTGTCA

TCACTCTTTTATGCTCATCTTTCTAAGTTGGCTGGTATCCCAGATGGAGTGCTCAATGTAGTGCCCGGATTTGGCTCAATTGCAGGGGCTGCAATAAGTT

CACATATGGACATTGACGCGGTCAGTTTTACAGGTTCAACAGAAACAGGTCGTAAAATAATGCAGGCTGCAGCCTTAAGCAATTTGAAACCTGTTTCACT

CGAATTAGGAGGAAAGTCACCACTTTTGATTTTTGATGATGCTGATGTAGACAAAGCTGTTGATCTTGCTCTCTTTGGCATCCTACACAACAAGGGAGAA

ATCTGTGTTGCATTCTCCAGAGTTTATGTCCAGAAAGGGATTTATGATGAATTTGAGAAGAAGGTAGTCGAGAAGGCTAAAACTTGGGTTGTGGGGGACC

CTTTTGATCCTAAAGTTCAGCAAGGACCCCAAACTAGTAAGGCACAATATGATAAAATTCTTTCCTATATTGAGCATGGAAAGAGTGAAGGAGCCACACT

GTTGACTGGGGGAAATCCAGCGGGCAACAAGGGATACTACATTGAGCCTACCATTTTTGCAAATGTTAAGGAGGACATGCTGATTGCACAAGAGGAAATA

TTTGGACCTGTGATGACACTTTCCAAGTTCAAGACCATTGAGGATGGAATTAAGAAAGCCAACAGCTCCAAATATGGCCTAGCAGCAGGGATTGTGACCA

AGAACTTGGATATTGCAAACACTGTATCAAGGTCCATCCGTGCAGGCATCATTTGGATCAATTGCTTCTTTGCCTTTGATATTGATTGCCCTTTTGGAGG

GTATAAGATGAGTGGATTTGGAAGAGATTATGGACTGGAAGCACTTCATAAGTTTCTCAAAGTTAAATCTGTTGCAACTCCTATTTACAATTCTCCTTGG

CTTTGA

>Glyma.04G181100.1 CDS

ATGGCTGTCTTCAATTCATTGCATAAAACTTCCACAAGATTTTTTGTTCCCACAGGGAGAAACTCTGGCAAGTGGGGAAACGTTAATAGATTCAGCACTG

CAGCAGCAGTTGAGGAATTAATCATTCCACAAGTTCCAATAACATACACTAAGCATTTGATAAATGGACAATTTGTGGATGCTGATGCTGCTTCAGGGAA

AACTTTTCCAACATATGACCCGCGCACAGGAGAAGTGATTGCTCGTGTTGCTGAAGGTGATGCAGAAGATATCAACCGTGCAGTATCAGCAGCTCGCAAA

GCCTTTGATGAAGGACCTTGGCCAAAAATGACTGCTTATGAAAGATGCCAGATAATACTGCGCTTTGCTGATTTGACATGGAACAATGGAAAGCCTTATG

AACAATGGGCCACTTCTGAATTACCAACGTTTGTGCGTTTGTTTCGTTACTATGCTGCTGATAAAATCCATGGTCTGACAGTGCCTGCTGATGGAAATTA

TCATGTGGAGACATTGCATGAACCAATTGGTGTTGCAGGACAAATCATACCTTGGAACTTTCCTCTCCTCATGTTTGCTTGGAAAGTTGGACCAGCTCTT

GCATGTGGAAATACTGTCATCCTTAAGACTGCTGAACAAACACCTTTAACAGCTCTCTATGTGGCAAAAGCCGGTCTTCCACCAGGTGTTTTGAATGTAG

TTTCTGGCTATGGTCCAACTGCTGGTGCAGCTCTAGCAAGTCATATGGATGTTGACAAGCTAGCATTCACCGGATCTACTGAGACTGGAAAAGTTGTACT

TGAATTGGCCGCAAGAAGCAATTTGAAGCCTGTGATATTGAACTTGGAGGGAAATCTCCTTTCATTGGGGCAATGTTGCTGTGCTGGGTCTCGTACCTTT

GTACATGAGCGTATCTATGATGAGTTCTTGGAGAAAGCAAAGGCTCGTGCTTTGAAACGTGTTGTTGGGGATCCCTTCATAAAGGGTGTCGAACAAGGTC

CTCAGGTGTGTTTTGCTAGTACTCTTAGGCAGAATATTATTGATTGTGTTCTTTCTTATCATTTTTGTTCCTTTTACAGTTACTACAAGGCCACCCTTGA

ATGTGGGGGTGACAGAATTGGCTCAAAGGGCTTCTTTGTCCAGCCTACTGTCTTCTCAAATGTTCAGAGAGTTGGAACTGTGTGGATCAATTGCTTTGAT

GTTTTTGATGCTGCAATTCCTTTTGGAGGGTACAAAATGAGCGGCATTAGCAGGGAGAAGGGTATCTACAGCCTCAACAATTACCTGCAAGTGAAAGCTG

TGGTTTCACCAGTGAAGAATCCTGCATGGCTGTGA

>Glyma.02G051500.1 CDS

ATGGATATTGGTGGTGGAGTGGAAGAGCCAGTGAGAGAGTTGAGGCAATACTTCAAAACTGGAAAAACCAAGAGTGTAACATGGAGGAAAAACCAGCTCA

CATCTCTCATAGACCTTGTTCATGAGAATGAAGATGCTATATTTAAGGCCCTCCATAAAGATCTTGGGAAGCACCCTGTTGAGGCTTACCGTGATGAGGT

TGGAGGGGTAGAAAAATCAGCCAGCAAAGCTTTGAGCTGCGTTGAGAAATGGATGGCCCCTAAAAAGAGTGATATCCCTTTTCTTTTCTTCCCAGCTAAA

GGAGAAGTGTTGTCAGAACCACTTGGTGTGGTTCTCATAATTTCTTCTTGGAACTTCCCAATCATACTGGCATTGGATCCAATAATTGGGGCAATATCTG

CTGGAAATGTTGTAGTCATTAAACCTTCAGAGCAAGCTCCAGCATGCTCTTCTTTTCTTGCCAATACCATTCCTCGCTACTTGGACTCTAATGCCATCAA

GGTGATTGAGGGTGGAGAAGATGTCTGTGAACAATTACTGCGACAAAAATGGGACAAAATATTCTTCACTGGAAGTCCACGTGTGGCAAGCGTAGTGATG

TCTGCTGCTGCAAAGAATTTAACTCCTGTTACTCTAGAGTTGGGTGGAAAATGCCCTGCCATACTGGATTCCCTTCCCAATCCTTCAGAGTTTGAGTTGG

CAGTGAAAAGAATTGTAGGAGGAAAGTGGGGACCTTGCAGTGGTCAAGCTTGCATAGGAATAGACTATTTGCTTGTTGAGGAGAAGTTCTCATCTGCTGT

GATAAAACTATTAAAGAAGTTCATAAGAAGATTTTATGGTGAAAACCCAGTGGAGTCAAAGGTAATTTCAAGAATAATAAACAAGCAGCACTTTGAGAGA

TTATGCAATCTTCTCAAAGACCCTCTTGTTGCGGCTTCCATCGTTCATGGTGGTTCAGTCGACGAAGAAAACTTGTTCATTGAGCCTACAATTTTGTTAG

ATCCTCCACTAGATTCTGAGATAATGGCAGAAGAGATATTCGGCCCACTGCTTCCTATAATCACATTGGATAAAATTCAGGAAAGTATTGAGTTTATCAA

TGCAAAGCCAAAACCTCTTGCCATTTATGCCTTCACCAAAGATGAAACTTTCAAGAGAAAGATTCTATCAGAAACATCCTCAGGAAGTGTCGTATTTAAT

GACACAATGGTTCAATTTTTATGTGATACGCTGCCATTTGGAGGAGTTGGCCAGAGTGGTTTGGGAAGGTACCATGGGAAGTACTCTTTTGACACTTTCA

GCCATGAAAAAGCTGTAATGCATAGAAAGCTGTTCCTTGAAATTGAGCCAAGGTACCCTCCTTGGAATAAGTTCAAGCTAGAGTTTATCAGATTAGCATA

CAGGCTGAACTACTTTGGACTAGTACTGCACATGCTGGGCTTGAAAAGATACAACTAG

>Glyma.04G248500.1 CDS

ATGTCGGGTGAGGAGACACAGAGGAATGTGTTTGGCGCGGAAACGGCGTCGTCCTTGGTGAAGGAGCTGAGGGACAACTTCGGTAAAGGAACCACGCGAA

GTTACGAATGGAGAGTGTCGCAAGTGAAGGCTCTATTGAAGGCGGTGGTTGAAAACGAGGACCAGATCGTCGGTGCTCTTTGTTCCGACCTAGCCAAGCC

TCCGCTCGAAACCGTCGTCTACGAGATTGGTATGTTTCAAAACTCATGTGAAGTCATACTGAAGGAATTGAAACATTGGATGACGCCAGAAAAGGTCAAA

ACGTCAATCAGAACTTTTCCTTCTTCAGCTGAAATAGTACCTGAACCGCTGGGTGTCGTGTTAGTCATCTCTGCATGGAACTATCCAATCTTGTTGTCCC

TTGATCCAGTAGTTGGAGCTATAGCAGCTGGTAATGCTGTGGTTCTAAAGCCATCCGAAATTGCTCCGGCCACTTCATCAGTACTAGCAAAGTTGATAGA

GAAGTATATGGATAACTCATTTGTAAGAGTTGTTGAGGGGGCAGTTGATGAAACAACTGCACTATTGCAGCAAAAGTGGAACAAGATTTTCTACACAGGT

AATGGAAGAGTGGGAAAAATAGTGATGACTGCGGCTGCAAAACACCTTACACCAGTTGTACTGGAGCTTGGAGGAAAATCTCCTGTTGTTGTTGATTCAA

ACAACAATTTGCTGGTAGCAGCTAGACGAATAATTGCTGGCAAGTGGGGATTGAACAATGGACAAGCCTGCATTTCTCCCGATTATGTTATAACAACAAA

AGACTATGCTCCCAAGTTGGTGGATACCCTAAAGACTGAATTGGAGTCATTTTATGGAAGGAACCCGTTGGAATCTGAAGATTTGTCTCGAATTGTGAGT

TCCAACCACTTTGCTCGCTTGTCAAAGCTCTTGAATGATGATAAGGTTTCTGGCAAGATTGTTTATGGAGGCGAAAAGGATGAAAAGAAATTAAGGATTG

CTCCCACTATTTTATTGGATGTTCCGCAAGATTCTTCGATAATGGGTGAGGAGATATTTGGTCCATTGCTTCCCATCATCACGGTCAATAAGCTCGAAGA

AAGTATAGATGTGATCAACTCAGGAGCAAAGCCCCTCGCTGCATATGTATTTACAACCGACAACAAGTTCAAGGAGCAGTTCGTAAAGAATGTTTCTGCC

GGGGGTTTGCTCGTCAATGACACTGCCTTACATCTTGTGGTTGATACCTTGCCATTTGGGGGAGTTGGGGAGAGTGGAATGGGTGCATACCATGGGAAGT

TCTCTTTTGATGCTTTTACACACAAAAAAGCAGTTCTTTATCGCAGTTTTGCTGGGGATTCAGCAATAAGGTACCCACCATACACGGATACAAAACTGAG

ATTGATGAAGGCTCTCGTCGGTGGTCGTATCCTCGGAATAATTCGTGCTCTTTTCGGATGGTCCTAA

>Glyma.06G114300.1 CDS

ATGTCGGTTGAGGAGATGCAGTCACAGAAGAGGAATGTGTTTGACGCAGAAACGGCGTCGTCCTTGGTGAAGGAGCTGAGGGACAACTTCGGTTCGGGAA

GGACTCGGAGTTATGAATGGAGAGTGTCACAAGTGAAGGCTCTGTTGAAGGCGGTGGTTGATAACGAGGAACAGATCGTCGACGCTCTTCGTTCCGACCT

AGCCAAGCCTCCACTCGAAACCATCGTCTACGAGGTTGGTATGTTTAAAAACTCATGTGAAGTCATACTGAAGGAATTGAAACAATGGATGAAGCCAGAA

AAGGTCAAAACATCAATCAGAACTTTTCCTTCGTCAGCTGAAATAGTACCTGAACCACTGGGTGTTGTGTTGGTCATCTCTGCATGGAACTATCCAATTT

TGTTGTCTCTTGATCCAGTCGTTGGAGCTATAGCTGCTGGGAATGCTGTGGTTCTAAAACCATCAGAAATTGCTCCTGCCTCATCATCATTATTATTAAA

ATTGATAGAGAAGTATTGTGATAACTCATTTATAAGAGTTGTGGAGGGGGCAGTTGATGAAACAACCGCACTATTGCAGCAAAAGTGGGACAAGATTTTC

TACACAGGCAATGGAAAAGTGGGAAGAATAGTGATGACTGCTGCTGCAAAACACCTTACACCAGTTGTGCTGGAGCTTGGAGGAAAATCTCCTGTTGTTG

TTGATTCCAATGTCGATTTGCAGATAGCAGCTAGGCGAATAATTTCTGGCAAGTGGGGATTGAATAATGGACAAGCCTGCATTTCTCCAGATTATGTTAT

AACAACAAAAGATTGTGCTCCCAAGTTGGTGGATGCCCTAAAGACTGAATTGGAGAAATGTTATGGAAAGAACCCGTTGGAATCAGAAGATTTGTCTCGG

ATTGTGACTTCCAACCACTTTGCTCGATTGTCAAAGCTATTGGATGATGATAAGGTTGCTGGCAAGATTGTTTATGGAGGTGAAAAGGATGAAAAGAAAT

TAAGGATTGCTCCCACTCTTTTATTGGATGTTCCGCGAGATTCTCTGATAATGGGTGAGGAGATATTTGGTCCATTACTTCCTATTATCACGGTGAATAA

AGTGGAAGAAAGTATTGATTTGATCAACTCAGGAACAAAGCCCCTGGCTGCATATATATTTACAACCAACAAGAAGTTAAAGGAGCAGTTCGTAATGAAT

GTTCCTGCAGGGGGTTTGCTTGTCAATGACACTGTCTTACATCTTGTCGTTGATACTTTGCCATTTGGGGGAGTTGGGGAGAGTGGAATGGGTGCATACC

ATGGGAAGTTCTCTTTTGATGCTTTTACACACAAAAAGGCAGTTCTTTATCGCAGTTTTGCTGGTGATTCATCATTAAGGTACCCACCATACACGGATAC

AAAGCTGAGATTGATGAAGGCTCTCATCGGTGGTCGTTTCCTCGGAATAATTCGTGCTCTTTTCGGATGGTCCTAA

>Glyma.13G030400.1 CDS

ATGTCGTCGACGCCGCAGGACTCTGTCAAAACGACGGCGTCGGCGAAGAACACCGCGTTCGACGCGGAAGCGGCGTCGCGGCTCGTGAATGAGCTTAGGC

GAAACTTCGCTTCGAACAAAACGCGCAGTTACGAGTGGAGACTGTCGCAGCTCAACGCGCTCGAGAAACTCGTCGTCGTTCACGAGCAGGAGATCGTCGA

CGCGCTCCGAAACGACCTCGGCAAGCCGCCACTCGAAACCGTTGCATACGAGATTGCTATGTTGAAAAACTCATGTAGAATCGCACTCAAGGAATTGAAA

CATTGGATGACTCCTGAAAAGGTCAAAACTTCAATCGCAACTTTTCCTTCTTCAGCTGAAATAGTATCTGAACCACTGGGGGTTGTGTTAGTCATCTCTG

CATGGAACTACCCATTCTTGTTGTCACTTGATCCAGTCGTTGGAGCTATTGCAGCTGGTAATGCTGTTGTTTTAAAACCATCAGAAATTGCTCCTGCCAC

ATCATCACTGCTGGCAAAGCTTATCGGAGACTACTTGGATAACTCATGTATTAGAGTTGTTGAGGGAGCAGTTGATGAAACATCTGCATTACTGCAGCAA

AAGTGGGACAAAATTTTCTATACAGGTAATGGACGAGTGGCACGCATTGTGATGGCTGCTGCTTCAAAACACCTAACACCAGTTGTGCTGGAGCTTGGAG

GAAAATCTCCAGTTGTTGTTGATTCAAATATCAATTTAAAGGTAGCAACAAGACGAATAATTGCAGGAAAGTGGGGTTCTAATAATGGGCAAGCCTGCAT

TTCTCCAGATTATATTATAACAACTAAAGACTATGCTCCCAAGTTGGTGGATGCGCTAAAGACTGAATTGGAGAAATTTTATGGAAAGAACCCATTGGAA

TCAAAAGATTTGTCCCGTGTTGTGAACTCCAACCACTTTAATCGGTTGACAAAGCTCTTGGATGATGATAAGGTTTCTGGTAAGATTGTTTATGGAGGCC

AAAAGGATGAAAACAAATTGAAGATTAGCCCCACTGTTCTATTGGATGTCCCACGGGACTCTTTGATTATGAATGAGGAGATATTTGGTCCTTTACTTCC

CATCCTCACGGTTGACAAACTAGAAGAAAGCTTTGACGTGATCAATTCAGGACCCAAGCCTCTCGCTGCATATATATTTACAAATAACAAGAAGCTCAAG

GAGCAATTTGTTATGACTATTTCAGCTGGTGGTTTGGTTGTTAATGACACTACTTTACATCTTGCAGTTCATACTTTACCATTTGGTGGAGTTGGTGAGA

GTGGAGTGGGTGCATACCATGGGAAATTCTCATTTGAAGCTTTTAGCCACAAAAAAGCTGTTCTCTATCGCAAATTTATTGGTGATGCTCCAGTAAGGTA

CCCACCATACACAAATACAAAGATGAGATTGCTGAAAGCTATCATAGGTGGTGGCATACATGGTATTGTTCGTGCCCTGTTTGGTTGGTGA

>Glyma.14G152100.1 CDS

ATGTCGTCGTCGACGCCGGACTCAGACAAAACGACGACGTCGTCGAAGAAGAGCGCGTTCGACGCGTTAGCGGCGTCGCGGCTCGTGACGGAGCTCAGGG

GAAACTTCGCTTCGGGCAAAACGCGTAGTTATGAGTGGAGATTGTTGCAGCTCAACGCGATTGCGAAACTCGTCGTCGATCACGAGCAGGAGATCGTCGA

CGCGCTCCGAAACGACCTCGGCAAGCCGCCACTCGAAACCGTCGCATACGAGATTGCTATGTTGAAAAACTCATGTAGAATCGCACTCAAGGAATTGAAA

CATTGGATGACTCCTGAAAAGGTCAAAACTTCAATCGCAACTTTTCCTTCTTCAGCTGAAATAGTATCTGAACCACTGGGGGTTGTGTTAGTCATCTCTG

CATGGAACTACCCATTCTTGTTGTCACTTGATCCAGTCATTGGAGCTATTGCAGCTGGTAATGCTGTTGTTTTAAAACCATCAGAAATTGCTCCTGCCAC

ATCATCACTGCTGGCAAAACTGCTCGGAGACTACTTGGATAACTCATGTATTAAAGTTGTTGAGGGAGCAGTTGATGAAACATCTGCATTACTGCAGCAA

AAGTGGGACAAAATTTTCTATACAGGTAATGGACGAGTGGCACGCATTGTGATGGCTGCTGCTTCAAAACACCTTACACCAGTTGTGCTGGAGCTTGGAG

GAAAATCTCCAGTTGTTGTTGATTCAAATATCAATTTAAAGGTAGCAACAAGACGAATAATTGCAGGAAAGTGGGGTTCTAATAATGGACAAGCCTGCAT

TTCTCCAGATTATATTATAACAACTAAAGACTATGCTCCCAAGTTGGTGGATGCCCTAAAGACTGAATTGGAGAAATTTTATGGAAAGAACCCATTGGAA

TCAAAAGATTTGTCCCGTATTGTGAACTCCAACCACTTTAATCGCTTGACAAAGCTCTTGGACGATGATAAGGTTTCTGGTAAGATTGTTTATGGAGGCG

AAAAGGATGAAAGCAAATTGAAGATTAGCCCCACTGTTCTATTGGATGTCCCACGGGACTCTTTGATTATGAATGAGGAGATATTTGGTCCTTTACTTCC

CATCCTCACGGTTGATAAAATAGAAGAAAGCTTTGACGTAATCAATTCAGGATCAAAGCCTCTCGCTGCATATATATTTACAAATACCAAGAAGCTTAAG

GAGCAATTTGTTATGACTATTTCAGCTGGCGGTTTGGTTGTTAATGACACTACTTTACATCTTGCAGTTCATACTTTACCATTTGGGGGAGTTGGTGAGA

GTGGAGTGGGTGCATACCATGGGAAATTCACATTTGAAGCTTTTAGCCACAAAAAAGCAGTTCTATATCGCAGATTTATTGGTGATGCACCAGTAAGGTA

CCCACCATACACAAATACAAAGATGAGATTGCTGAAAGCTCTCATAGGTGGTGGCATACTTGGTATTATTCGCGCCCTGTTTGGTTGGTGA

>Glyma.08G002700.1 CDS

ATGAAAAGCCTCTGCCTTGGGCCGTTTCTTGCTGCTAGTGCCCCAGTTGGTAGAAGAGCCTATGGAGGCCACTTAAGCAGAAAGTGCTTTCAAAAGCAAC

TTCACTTCCACTCACGTTGTGTTGCTTTCTCTTCATTCATATGTTCTGCAACTATATCAGTGATGCCAGAATTAGAAGAGAAGCAAGTGTTTGATGGAGA

GAAGGCTAATTTGCTAGTTAAAGATCTCAGAAAGAGCTTTGATTCAGGCATGACTAAGAGCTATGGATGGAGGGTTTCCCAGTTGGAAGCCATAGCTAAG

ATGTTAGAAGAGAAAGAGAAGGAAATTACTGAAGCTCTATATAAGGACCTTGGAAAACCTCGACTTGAAGCATTTATAACCGAGATTTCCCAGGCTAAAT

CCTCATGTAGTGAAGCACTTAAAGAGCTGAAAGAATGGATGAAGCCTGAGAAGGTGAATACTTCAATCACAACATATCCATCATCAGCAGAGATTGTGCC

AGAACCACTGGGGGTTGTGTTGGTCATCTCAACATGGAACTTTCCTTTCTTGTTATCAATGGATCCAGTCATTGGAGCCATTTCCGCAGGCAATGCTGTG

GTCCTGAAACCATCAGAAATTTCTCCAGCAACATCATCACTGCTTGCAAATTTGATCGAACAATATCTAGACAACTCTACCATAAGAGTTGTTGAAGGGG

CTATTCCCGAAACATCTGCACTCCTGGATCAGAAGTGGGATAAGATACTTTATACAGGTAGTGCTAGAGTAGGTCGCATTGTCATGGCTGCTGCTGCAAA

GCATCTTACTCCAGTGATTCTGGAACTTGGAGGAAAGTGCCCAGCTGTTGTTGAATCAGATGTCAACTTACAAGTTACTGCAAGGAGGATAATAGCTGGC

AAGTGGGCATGCAATAGTGGACAAGCATGCATTTCTGTTGATTATATCATCACAAGAAAAGAGTTTGCCCCAAAGCTGGTAGATGCCTTAAAAGAAGAAT

TGGAACAATTTTTTGGTAAAGATCCTATGGAGTCAAAAGACATGTCAAGAATTGTGTCTCCAAACCAGTTTGCGCGGCTTGTGAATCTCTTGGATGAGGA

TAAGGTATCTGACAAAATTGTTTTGGGAGGTCAGAGGGATGAGAAGAAATTAAAAATTGCCCCAACTATCATATTGGGTGTTCCTGAAGATGCTATGATA

ATGCAAGAGGAGATATTTGGGCCAATAATGCCAATCGTCACTGTAGACAATATAGAAGATTGCTACAGTATAATAAAATCAAAGCCAAAACCTCTTGCTG

CATATCTCTTCACAAACAATGAGCAGCTGAAGAAGGATTATGTGGATAAGATATCTTCTGGAGGGATGCTCATCAATGACGCCGTCATCCATGTTGCAAC

TCGTGGGTTGCCTTTTGGAGGAGTTGAAGAGAGTGGAATGGGTTGTTACCATGGAAAATTCTCTTTTGATAGTTTCAGCCACAGGAAGTCAGTTCTCTAT

AGAAGTTTTGATGCAGATTCTACCATTAGGTACCCTCCGTATACACCTCAGAAGGAAAAATTATTGAAGGCCCTTATTAGTGGCAATATTGTTCAAATTA

TTCTTTCTCTTTTGGGGTGGTCTTAA

>Glyma.11G133300.1 CDS

ATGGAGATCACTATGCAAACTTTGGAGAGAGACCTAAATGACACGAGGGGGTATTATGAGAGTGGAAAGACTAAGGAAGAGTCTTGGAGAGAATCGCAGC

TCAAAGGGCTGCGTCGTTTTCTCTTGGAAAAACAAGTAGATATCATGAATGCCCTCATGCATGATTTAGGGAAACATCAACTTGAAGCTTTCAGAGACGA

GATAGGAACGTTGATTAAGACCGTAAATTTGGCATTGAAGTCTTTGAAAGATTGGATGTCAGGAAAAAAGGCTGCGTTGCCACAATTAGCATTGCTCACC

AGTGCAGAAATTGTTCCGGAGCCACTTGGTCTGGTTCTCATTATTTCATCTTGGAATTTCCCCATTGGAATATCCTTGGAGCCACTGATAGGAGCAGTAG

CTGCGGGAAATGCAGCAGTCTTAAAGCCTTCAGAGTTGTCTCCGGCATGTTCTTCTCTACTTGCTTCTAGTCTCCCCACTTATTTGGACGATAAAGCCAT

TAAGGTGATCCAAGGAGGGCCACAAGAGACCCAGCAACTTCTAGAGCAAAGATGGGACAAAATCTTCTTCACAGGAAGTGCACGAGTGGGGCGGATTGTG

ATGTCTTCTGCTGTGAAGCATCTAACTCCTGTGACTTTGGAGTTGGGTGGAAAATGCCCTGCAGTTGTTGATTCCCTTTCATCTTCTTGGGACAAAGAGG

TCACTGTGAAGAGAATAATTGTGGGGAAATATGGGACTTGTGCTGGTCAAGCATGCATAACAATTGATTATGTCCTTGTGGAAAAGGGTTATTGTTTAAA

ACTGGTGGAATTGATGAAGGTCTGGATCAAGAAAATGTTTGGACAGAACCCTCGAAAATCAAAAACTATTGCTAAGATAGTTAATAAACACCACTTCTCT

AGATTGAAGAATCTTCTTGCGGATAAACAGGTCAAAGGTTCTGTGGTTTATGGTGGTTCAATGGACGAACAAAACTTATTTATTGAGCCAACGATCTTGG

TGGATCCCCCACTGGAAGCAGCAATTATGTCGGAAGAGATTTTTGGCCCATTGCTTCCAATTATTACCGTGGAGAAGATTGAGGACAGTATTAAATTCAT

AAATGCTAGACCTAAGCCTCTTGCCCTCTATGTTTTCACCAAAAACCATACACTCCAGAGAAGAATGATATCTGAAACATCATCTGGCAGTGTGACTATC

AATGATGCAGTTCTACAATATGCAGCTGATACTATTCCATTTGGAGGAGTTGGGGAAAGTGGGTTTGGCATGTACCATGGGAAATTCTCCTTTGACACAT

TTAGCCACCAGAAAGCAATTGTAAGAAGAAGTTTCCTCACTGACTTTTGGTATAGATATCCTCCATGGACACTAAATAAGTTACAACTACTTGAAGTCTC

TTACAATTATGATTATCTGGGATTGCTCCTTGTCCTGTTGGGCTTAAAGAGACCATCAAAACGCTTAATCGCAGATCATGTTTAA

>Glyma.12G057400.1 CDS
[truncated: 79,716 more chars]
