# Supplementary material for: Genome-wide characterization of the aldehyde dehydrogenase gene superfamily in soybean and its potential role in drought stress response
Source: BMC Genomics. 2017 Jul 7;18:518. doi: 10.1186/s12864-017-3908-y (PMC5501352; doi:10.1186/s12864-017-3908-y)
Supplement: Supplementary file 19 — The 1500 bp upstream sequences of the 53 GmALDH genes. (DOC 111 kb) [file 12864_2017_3908_MOESM19_ESM.doc]

**Additional file 19. The 1500 bp upstream sequences of the 53 *GmALDH* genes.**

**Note:** The 1500 bp upstream sequences of 13 up-regulated *GmALDH* genes are listed on pages 1-9, 14 down-regulated *GmALDH* genes are listed on pages 9-17, and 26 non-regulated *GmALDH* genes are listed on pages 17-32. Changes in expression were determined by the criteria of *P* value < 0.05 and │log_2_ fold change│≥ 2 comparing soybean plants under drought stress with control.

**The 1500 bp upstream sequences of 13 up-regulated *GmALDH* genes in response to drought stress.**

>GmALDH2B1

ACATAAAAGAAAACCAACCAATAAAAGTTAAATAACAAAAGAAAAAGGAAAATTGGATTAGTCATCACAACATGCTATGCCAAATCAGTCAGACACAATGCTGGCCAATCACAACCACCTATTTCCGGCGAAATTTGAATCACAGTCATAGTGACGTGTACAAAAGAAATACTATATCTTCTTAAAATATTAATAATATTTAAATAAAAAAAATATCAAATGAAAAAGAGAGAAAATGTTTCTGGTCTTAAAATATTAAGTACATTAAATTTAATATCTTCAAAATTTCAAACTGATTAATTAGGATGATTCAAAACTTATCTTTGAAAACCTGGATCCCATTCATTTACATTAAATGTTAATCAAGAAAGTAGTAAAAAAATAAATAAATAAATAAATAAAAGCTTTAAAGGATGATTGTATTAAATAACAGATAAAAAGGTTTCCCAATTAAAAAGGAATAAAAATGTGTGGCAGTATAAGGATAAGACATAAAAGCTGCGAACAGTTGAACAAGAAAAGGAACCTTGGAATATGGAAATCGAAGGAAACAGATAAAGGCTGAGGTTGCCACCGTAATAGTCTTTCCCTTTATTCAACGTTCAATCATAAAGGCTGGTCGACCAACGTGTGCTCATCCATGTCAAATTGAATTTGGATATTTTAAATTTTAATTTTATAAATCGAAAAATATAATTAAGAATAAATACTTTATTACAGAAAAATACATATTTTTGTACGAATATTAAGTATTGGAAAAGTTAATAAATTCTTGACATCGGTAACAAAATAATAAATAAAAGAATCGACACTGAATATTATTTAATATCCCACAAGAAAAAAAAGGACAAAATTATAAATGTAACATTAACACCTGATGATCAGAATAATAAGTATTAATAGAATATTTGATATAGAGAAACACATACTTAACAAGTAGCTGATATTATATTTGATTTTCTTTAGTAAAATATCTAATTTAAATCTTAATGAAAATAAAATTAATTAGAAAGATAAATTATTCACTAAAAATGATAAATCAGATATAAAGCTCATGACTATTATGTGGATTATCTTTCAAAACTATTAATAGACCAACATTATATTAGTCTAAATAAAACTGAGTACATGATGTGTCATTCAAGTCTTTAGATTAAAAAATATAAACAAAAGAAGAAAATCTATTAAAAATAATCAGTTACGTTTAAAAAAAAAATTAAAAATTAGTCTGAGTTCAATGATAACCTTATTGAAAAAATACCCATTATGGAAGGTGTGGTGAGCTGAGATGTCAACGTATTTTGGTGGCTCACGTGTTAGTGTCGCATTATTTCTGGGGTGATTGATAACGACATTTACGATTACGTTCACAATTGGTTAGAGGGTTGATTTCCCATTTGACCGTAACTATATAATCTTGGAAGAATTTCACAAAGTTCATAAAACTGGACTATAAACAGTAGTGGAGAGAGGGAGAGAAACCAAAGAAGAAGATTGACC

>GmALDH2B2

AAATCTTTTATACATAAAAAACAAAAATCAATCAATAAAAGAAGAAAAAGGAAAGGCAAAATTGGATTAGTCATCATAACATGGAATGCCAAATCAGTCGACACAATGCTGGCCAATCACAACAATATATTTCCGGCAAAATTTTGAATAATTGAATCACAGTCATAGTGACGTGTACAAAAGAAATACTATATCTTCCCAAAATATTAATAATATTTAAATAAAAAATATCAAATGAAAAAGAGAGAAAATGTTTCTGGTCTTAAAATATTAAATACATTAAATTTAATATTTTTTATTTTTCAAATTGATTAATTAGGATGATTCAAAACTTATCTTTGAAAACCTGGATCCCATTCATTTACATTAAATGTTAATCAAGAAAGTAAAAAATAAAAATATTAACCTTAAAAGGATGACTGTATTAGTTAAAACTTAAAAGGATGAAGATGAACCTTAAAATAAAAAGGTTACCCAATTTAACCGGAAAAAAACTGAGTGTCAGGATAAGGATAAGAATAAGACATAAAAGCTAGGAACAGATCAACAAGAAAAATAAAAGCTTGGAATATGGAAAAATAGAAGGAAACAGCCAAAAAGGCTGAGATTGCCGCCATAATAGTCTTTCCATTTATTCAACGCTCAAATTGGGTAATCATAAAGGCTGGTCGACCAACGTGTGCTCATCCCTATGGAATTAAATTTGGATATTTTGAATAAAATTTTAAGTTTAGATTTCATGAATGAAAATAAGTAATTAGGAAGAGAAATTTTATTAAGAAAACTAATCAAGATATTTAACATCAATCATAACATAGTAAAACAAAAAAGATTTGGTTGACACTGAATGTTATTTAATATTTAACAAGAGAAAAAAATATAAAAGTAACATGTAATGAAATACTGAGATAACAAAAAATATACAAAAATAATAAGTATTAGTATTAGTAGAATACTTGATACAAAGAGATGCATATGTATTATCATTCAAAACTTTTAATAATGTCTAAATGAAATGAGTTCATAATATCTTAAATTCAAGTTTTCAATAAAAAAATAATTCTGACATGATTAATAAATAATTTTATTCCTTAAGTTCATAACTATTAGTTATTGACTTTCAAGTTATCATTGGTTCGAAAAAAAGAAGAAGCAGAAAATCTATTAATAATAGCCAGTTAGCTTTCCTAACAAAAATTGAAAACTAGTAGTTCATAAAGTGACATATATTTTGTACCTATAACTTGATTGGAAAAAGAACACCTATTATGGAAGGTGTGCTGAGCAGAGATGTCAACGAATTATGGTTGCTCATATGTTAATGTCACATTATTTCTGGGGTGATTGATAACAACATTTAAGACTTCGTTCACAATGGGGTAAAGGGTCAGTTTCCCATTTGACTGCAACTATATAACACTCGTAACAATTTCACAAAGTTCATAAAACTTTACTATAAACAGTAGTGGAGAGAGGGAGAAAAACCAAAGAAGATTGACC

>GmALDH3H2

CATGTGCAATACTGGATCCTTTAGTTAGTCTTCAACACTTTCCTCCACCTATGTAAAAATAGAAATTTTAAATAAACAAATTTTAGGGGGTGAGAAGATGAAAAGAGGAGTGTAACTATCAGGCCCTGTTTTATTTTGTTATATATATGTTCGTTAACAATTAATTTACTTGACCCATGAAAAGGTCCGTTACTCAACCCACGCGCAAGGCCCAGCTACTCTGGACAATTCCCCCTTCCCTTGGCAGATCAGGGACATGGGCGCACGGCCTAGCTGCTACACAGAGTTTTCAGTGCAAATGTGTGTGTCTATATATATATATTTCTGGTTACCAATATTGCTAATTAATTTTTTAATGATTAATTATATTTTAAGTCTCTATAAATGTGATTTTGTCTGGTTCTAGTTTTTATAATTTTTTTTTAACTTTTAGATTTTAACATTGTTAATGTTTTTTTAGTTCTTTATATTAAATCTAGCTAGTTTAATATTGACATGAGTAATAAAACGAAGAATATAAGATAATTAAGTTAATGAATATTATAGTAAAAATTAATTAAATTTTTTATATATGGTACTATTAATTAAAATAATATTAATATAAAACATATTTTCGTACATAGTACTATTTCTATATTTATCAAATATTGCATAAAATTAACTGTAATATATTATATATGTGTTTATTAAATTTATTATGTATGACTTTTATATAAGTCTAGATATATCAATAATATAAATGTGTGGTTTTCAATATAAGTTTATGTGTATTAAAAATATATTATTAAATAAAATATAACAAGTATAGAATTTAATATATATGTATATATACATTAAAATATACTTATTTTATTGTTGTTTACAAAAGTGGTTTATAAAAATATTTTAAATTCATATTAATTACGTATTGACTCTACGTGACTTGTTCTTATTTTCTTTTTTCCAAAAATAGTAAAGTGGACTGTTTGGACGAAATTGGAATGGATGCGGCTCAGGGGCTAGAACTAACTAGTACCTTCTCTGTTTTTGTGTCCTTATGCAATTACGTGAAACATTTTATATCCAAAGCTGGAAACAAAATAAAACTATTCTATTCACGTTGTAAAAGTCCACACGCTCATTCCTACTCGATCGCCCATATGAACCCACGTTTTTGCGTCAAGCCTGATTCCTGAAGCCATCATATAGATGATGTTCCCTTCATATTTACGTTAATGATCGATTATTGCCACAATTAAGCTAGCTGTAATCCCCTCCCACACGCCATCCTCACGTCATTAATTACGACTAATCTCTCCACCTTTGCTATTATAATAATAATAATAATATAGTTTAATTTCCATCATCGAATTCATTATTCACACACATGTATATATGTATATATAGAGAGCTCTCCTCTCTTCCCTAATGTATGTGCTTTAACTTTGAAACCCAACCCAATTTCACCGTTGCTAGCTCTTGTTTCTTTTTTGTCGGAGAAAATAATAATAACAATATATA

>GmALDH7B1

TTTCTCTCTATAATTTTAAGGAATGGATATTCTCTTCCTTTCAAGTAATAGTGTATACTATGCAAATACAATACAATGAGTAAGAAATTACTGGATTATTAGGAAAAAGACTTCTAATTTCTTTTATCATTATCCGTCTGTCTTCTTTGTTTCGATGACTAAAAAAATCAAATACATTTAAGGGTAAATAGTCACTTTTATCTCTCAATATGTAATTCGTTGATGATAAATATATCTATGAATGATGAAAATACAAAATTTAGTCCTTTAAAGAGAGTAAAAAGTGCGATCAATATATCTGATCGTTAACTTCCGTCTGTCACCATTAATAAGATAGCCTATGTGACACGAAAGGATGAATTTGTCATTAAAATAATGATCAAAGTGGATTGACAACATATAAGCATATTTGTCATAATATTTTTTTTTGTTTTTTTTAAGTTAACTTCTATATTAGTTTGAGCATTATTGTATTTTTTATTTAAATTTATTTGAGTTTTTTATTTGTTAGTAATTGTTTTCATTTCATTCACATTATGTATAATAAGTGTTGTCTCGAGTGGAAGTACATAAGATTTATCTATGAGATTCTTTCATATTCGAGGTATATATATACATTTAATTGATTAAGGAAAAAGACTTATAATTTCACTTTAACTTTATCTAGTTTAAATTTTGTGAGACTTTTTTCTTTAAAAAAAAATAGTTGATTAATTTTTTTTTTAAAAATAAATAAATTTGGTCTCACTACGCGTCGTGTTTATCTACTCAAAACTCTCAAGACATTATTTTTTTAATCTAAATATGTACTTAAGTTGAAATTAGGATATGAAATAATTTTAAAGATTAAAAGAGAGAAATTTAATAAAAATTTGACAAAATATAAGATATAAGATAAAAAAAATTTAACAGCAAAGATTAAAGTTTGACAAGAAAATTAAAATAATAAAAATATATATATTAAAAGGCAATTTAAAAAATATATTACATAAGTACTCAATAAATAGGAATAATAAATTATAAACATATAATAATAATAAGTATCACACTTTTTATACTTTAGAAAATTAAATTTTATATTTTCAACTGAACACATTTATTAATGTGAAAAAACAAAAAGTAAAAATATTATAATAAATATGTCCTTATATGTCAATGACAAATTTGTCCCTCCCATGAACAACACATCTTTATATTTTCAATAATGCATCTTTCAATCAATTAGATCTAAGGAACACATAGAGGGACAAAAGTTACTAAATATCTTGAAATTAATTTTTATTTTTGTCCCACGTTATCTCAAAGTGCAAGCTTTGTTATTGTAAAAAAAAAACAAATACTGAATCCCCATCACTTTCTTCCCCATCGTATGTGTGTCCCTGTCAGAGTATGATATGAGAAACTCTATAAATTAACTATTTTTTCTCCCACTTTCTGAATCAAACACAACACACACACGTAACTTCATCTGGGTTTCAGGAAAAAAGAAAAAGGAAAACA

>GmALDH12A2

TGTGTCTTTATGCTTATGTAGGAGACGCTAAAAAGGAAAGCTTGTACCCGAATTTTTAAAAAAGGGGTTATTCTGTTTCATGCATCTCGGACAATTAAAGATAGTCATTTTACTTGCATTGACGAAGAACAGTCGCATAATGCAAATTCAAATCCCGGAGCAATTTTTTTTCTTTTAATTTGCGACAATCTTTTTTGCGTTGGTAGTTTAATTTCAGTATCTTTTCCATTACCTATCCATCAATTCAAAAATACCTCCGGATCTTCACTCCAAACATAGAGAATTCATAGGGGATTGAAGGAAAAAAAGAGAGAAGTGGAGATGAATTTGAACAGAGATGCAATGGGTGGTTTCTGGGGACTCGAGCAATGCTTCTGTAGAAACTAGAATCAAACGAATGCTAAGATTTTATTTTATTTTTATTGACAAATGTTATTTTGCTGATTTTATTAAAAATATTAGCATAGATTTGAACGTGCGATTTCTTTTCTCTTCCTTCTCCCTTTATTCTTTATGATTACTACTAGACTAATATTATATCGTACAGTAAAAACATCCTCTATTGCACTTATACTTTACTACAGGCAAATGACGCATTTCGTGCCTTACTTTTTACAAAGCAAAATAATTAAATATGTTGTTCACATTTTTTGAGAATGGTAGTTATCATTATAATAATTATAAATTATAGATTATAGTTTATAGATTATGCAGTTGCACTTGTTATTAACTAAAAATTACTTATTTAATAAATTTCATTCATAAAATTATAATTTTCAATTTTTTTAGTGAATACTAAAGAAAATATTAAAAAGTACAGTATATCCCTAACATCTTATATTAATAGCCTTGTCATAGTCACGTGTTAATATTTTTTTTTTGTCTGGATTGGAGATCATATAATATTTTTAATAATTTATTGGATTAACGATTAATTTCATTCATTATGAGGTTTATTAGGTGTGATTGTCATTAACACGAGATAATTTTGTACTCGGAGAAGAATCGAACAAGCTGGTATTTTGATATGATGCATCTTAAAAAATTTTATTTCTTGGTTAATAATTATTTTTTAGAATAAATTTTAATTTTTTATAACATTTTATACATATTTAAATCAGGAAATGCATTTAAATTTTTAACTAATACAGTATTTTTTATTAAAATAATAGAAAGATTTGTAGGAATACTTAAAAAAAGTAAAATCTTCCACTAATTGAGGCAGTCCAAATCTGTTACGGAAACCCATGCTATCTCCATGTGACATTTGTTACTAAGACCGGGTAATAGTATGGTGCGACTGACTCACTCGTACGTGTCCAACACGTGGGTTATAGAATCTAGAAGATTACGTTCAGAATCAAACTATCAAACTTGAAAGTGACACTTTTTTTTTTTTAATATTTATACGAATAAATTGGAGGAGAGTGTGTCACTTCATTTTGAACTCAACTGAAAGTGAGTGTGTGAAGGTAGGGTTTTAGAAACTGATCCTACGGC

>GmALDH18B3

CAAAAAATTTAGTTCAATATGAGGAAAAAATGACATTGATGATCACATGAGAAGACAATATTACATGTATTGCTCAGTTGAAAGAATGATATATCAAAGAAGATAAAACAAATCATATTTTACCAAAATTCTTTTTCACCAATGATCTAAAAAAGAATAGTGATAAAAATGTTCAACAAGTTCGTTCACGTGAAAATCTAGAAGATATCTTTAAAAGTCATTGTCAAGAAAGACTTTTGAGTAACAAGTTAACATGATTGGACTTTGTCACCTAAAAGATAATTATCTACACAAGAGAGATATAACACGTTCTACATTCTTTTTCCCTTAACCATGATTTTTCTAGTAAGATTTTAACGAGGTATATTCTCAGATAATGGACATCCAAGAGAGAGTGTTATGACTTATGTGGATGTCCATATATTCTTTTATCTTTATATGTGCTTATCTTTTGTAATTCTTCAGTTGTCTTTTATAACCTTTTGTATCTATAAATAAATTATTTCTCTTGAAATGAATACTCACAATTATCATTATATTTTTCTCTACTTTCTCTCTATACTATTCTCCTCCAAATTATATTATATTTAAAACATTTAAATATTAAAATAATAACTATTCATTTCTTTTTTAGAAAATAACTATTAATTTCTAAAAACCATATCTAGAACATAAAAGCAAACATACTTTAAAGGCTAAATTATAATTTTAGTCTTCTTAATTTTTCAAATTCATCATTTTGATCCCATGATTTTTAATTATAACACTTGATCCCCCTGATTTTATAAATTGATGATTTTGATCCCTTGATAGATTATCAACTAATAATTGTGATTAATAGAATTATTAAATTAATTATAAATAATTCATTATTAAAAAATTAATAATCTTTAATCACAATTATTAAAGTTTATTAAAGTCTTTAAGAATTCATAGATTTCATTAATTTTTTTAAAAATTAATAGTTTCAAATTAATTTATAATTAAGTTTATAAGTATATTAATTATAATTATCAGTTAATAATCTATCAAAAAGATTAAAATTATCAAATTAAAAAATTAGAGAGACCAAATATTACAATTAAAAATAATTAAAAATGAGGAAGAACAAAATGATAAATTTAAGAAATTAATATCAAAAGGTAATATATCCTACTTTAAATATGCATGCATGAACATCTCTTAAAATAACAATTAGCACCTTAGTTCGCCACCTGAGATAGCATCTTTTTTTCATTAGAACCGACCTCTTTATAAATATAAGTAAAAAACCCCTTTATCAGCCAAACTAGCACTGTACTCCAAACTCCAAAGTCAATGCAGAAGCCGTACTACTAAAAAATTCTGGTGACCACAAAACGCAGAAAAAAAAAAATTAAAAAAACCCCTAAAAATCCTGTGCATGCCTTTAAAAGCCCTAGTCATTTGAACAGAACCTCTCACACCAGTGGCTTCTATTTCATTCATTCATCTCTCATCTGAAACACAACAAGAGAAACA

>GmALDH2B6
CAACATGGGGAGTAAAACCCTTCAAACAATTTTACATAATCAATATCAAAATCAATTAATCAAAATCATAGGTAAAAAATGCGAAAACATCGAGAGCACTCAATTTTATCAACCAATTTGCATCAGGATATCAATTGGTCCGTCAAACACAACAATATCGTATTTATAATCATAAAGGAAAAATTATAATTCAATAAATATCTCAAAATAAATCTAATTTAATCCTTTAAGAATTCCTACACATGTTCATTCTAACCCCAATTGCGATAAACTCATCTCTTACCTTTAAACGAGCTCATGGGTGTAGTCAGATAGTAATAACAGCATCTCTAGCGGTTCTGTGAGAATCCTCAAATTTTTCCTTTGATTATTTTGTTAGGATTTCCAAGTGTCACAGAGAAGAAGAAAAAACTATAGCCTCCAATTCACTATCAACATGCAAGTCTAATTTCTCTTTGAAAGAACATTATTTCACAAATTCCAACGGTGTGTGTGTGAGAAAATGATTTCCAAAGGTTTTTTTTTTTTTCAAATTTCACGATGATCCAACGGTTAAAGAGTCCAAGATCATAGTTTTATTGGGATAGGTTTGGGTGTATGCACGAAAAGAGAAAGTTCAGTACGAGAGACATTTCTTCCACCAAGAATATTATCTTGAATATCCCAACGATGAGTGTGTTCGAATAAAGGTTTCGAAGTTGATGTTCATATTTCACGACGATCCAACGGTTAAAAAATATGAGATTGTCATTTTACTGAGACAGGTTTGAGTGTATGCGAAAAAGAGAGAGAGTTTTGGGAGGAGGAGAAGGGAAAACGAAGTTAAGAGCAAAAGAAAGCCTAAAGATGTATTGTGTGTTTGAAAACTGACATAATATATTATAGTTAGGGTACTTTAAACCTATTATTTACTCAATTTTTTATTATTTTATAAACAAAACCTCTATTTTATTTCCTATCAAATGAATAAATAGCATATCTTTTTATTTCCTTTCAAACCATTATTTTAATTATAAAAATCTCATTATTTTTCTAAAATTCTAATTATTTTTAAATAAAAAATATTTTTAATTTATTTTACAAAAATTGGGTATTACAGGGTGGATGATTATAATTTTCCTATCACATATAACTTGCAATTTGATGTTACCTATTATGGCGGCTATCATTATTATATTTTTAATTAGAATGTGACTCTTTTTAACTTGTTCATTGGCTCACTCTTAGAATAGTAATTTTGATCCATAAGCAAATTAGACATGATATGCTCATTTACGTTAATGATAACTGACTATTGCTAACTATGTTTTGCTGTCTACCTTCTCTTCCATTCGGGTGACACAGTACTGAGATGCTTACTGGGTAACACTTTATTAAGCTCTTAAAAGAAGGGTGGTAAGAAAATTAATTTCACGAAGATTTGTATAGTTCTAGAAATCTCTGAGGGTAGGATTACAAAGAAAGGGAAGAAAAAAAAATCCTCAACATTAAAGAATCTTC

>GmALDH2B9
ATTGTTGAATAATCTCTATTGAAAATTTTCTCTCTGATTATTCTAAAGTTTTTTTTTTTGTTTAAAATTAATTGTTTCAATAAAAAAAAATAAGAAAGACAATATTTTGTCTAATCATAATTTGGATCTATACAGATAGGAAACCAAACCCACTAAATTATACTATGTCTTTTTATTAATTAAATATGAGCTAATGACCTCAAATTCAGGATTATAATGTAATTCTGGCCACTCGTGCTTCCTGAAAAATGTAATCCTCCATAAATAAAATTCTCTTCCATATCAACTAAGCTAAGCCAGAAGGTATGATTAGATCCCTAATAATTTTTTATATAATGGATTCGGAATATCTCATTTCAAGTATTTTGAATATTAGGTGTCACGTCAAAACATAAATTATTTTGTTATTATCCAATAAAAAAATAAATATAAAGTATTTCTTTTTAGAAACCTTTCTTATTATAAATATTTAAAGTTGTGTATGCCTGGGATCTAACTTGCAAGGTCTCGTACACAATCTGCAAGATTCTCTGTTTCCCTTGAATGAGACTGTCTACCTCTTTCTTTGTTTTTTTATTTTTTTATTCCAAAAACGTTAAAGTGGAATTCACTGAATAAGATGGAAAAGCAAGATAAGTTATACATACATGGATGCAATCAAAGGATCTATTGTTCACACAATGTTATAAAATAAAAAAAAATTTAAAAAAAAACTGTAGAAAAAGAGTATTATATTATCTGTCTGAAACAAAGTAATTAACTTCTTTTTAATAATAAAATAGCAATATGTTATGCTACTCACACAATCCATGAATCGTTATACATAACTTTTGTTGGTTTCACTTTCATATAAAATGATTCTTTCACTTGAGACATTAAATCAGATTCTTATTTATAAAGCCATGATATAATTTTCGTTTGAAGATTTAACCTTTTTTCTTTTGTGTCTAACCAATTGACCTAAAGTAAAGGTAATTATATTCAAAAACTTATTTTTATATGGTTTTTTTAAATATTTAATAAGCATAAACTGATGAATTTAAATCTTCAAATAAATGATAACTAGTACTTTAGCCTACGTAATATACAAAATAAATGTTATGTAGTAATTAAAATTTATAATGAATAAATACTTTTAAACTTATAAATTATATTAAAATTAAAATTTATAATAAATAAATATTTAAAAATGTAAACTATATTTTAGATTTGTGGTAAACAATTTATATTATGGTACAATTTTTTAATTATTGATGTTTTCTTTTAAAAAATTATTGAAATTCAATTTTAAAATAAAGGTGAAGTTTTTGGTTACAAAAATAAATAAATAAAATAAAAGGTGAATAAAAAATTGAAAGAGATCAGTTAAAAAAACCCTAAAAATGATATTATTAAATAGTAATTCCTAAAAATGCTCCAGTTGAAGAATAGTCAAAATAATTATTCTAATTTATTTATATATAGAAGATAAGTATACTAACAGGTTGAATTTTAGTGGAA

>GmALDH2C3
CTTGCACATACGTCATACTATTTGTTGGGCGTTAATTATTCTCTAGTTTATACCAAATTTCAAGGTCAAATTTATTATTAAAAAATTAAGTCAAGATATATTTTAAAATTTTACCTAGTTAAGAAAAAGCCATTGTATAATAACAATATATTGTATTAAATTTTTATTACAATATTTTAACATTATATAGTTATTAGTAATTTAAGTGCCCATTGGCTTGCGTATAAAGTTAAGTTTATTAGCCTATTAGAGATAAATTTATAATCTTATTTAAATATCATATTATAAAGTAGGTGTTTAAATAAACTTTTAAGTTAGAATAGGCAATAAAGAAAACTTATATAGATAAATAGGTCCGAATATAATAGACCTTAAATTTAGATAACAAATTAAGATTAAGTCTTATAAAACTTAATTTGTATCTACTCCTATAATCTAGTGTTGGCAGCTAAACCATTTCATGTGTGACTTTTATATTAAAAGAAAAAGGTTTGACAAATCATATAAGATACACTAATACAAGGTGATACCGGAGCTAGCTATTTGTGATTTTGTGTCCAAAAGTAAGTTTGATCATTCTTTTTTTACTACTGACAATTTGTTTTAACAATAACGCTAGCTAATTGAAAATGAGGATTGTGTACTATACTTGATCTAATAACGACTACACAGACACAATTAATTTGTTTGTAAGTCTCAGTAAATTGTTTGATTCTAGGGAAGGAAAAGAAAAAAAAGGGTAACGTCAGAACTCAGAAGTAAACATATATATTGACTATAGTATTTTCTAAATATAATATATAAATATATACTCATAGTCAACATATATAGTCAGTTTTACTTAATCATAACATATGGACTATAGTATTTGTTAAATATAATATATAATATATACTAAAATTAAGCTACATAATTAAATACTATTTGAATGTTGTATTTACTAATCACAATAAATTTATTAAATTTTATTAAATATATAGTCAAAGTAAACATACAATTAAAAATTATTTTCGTTAAATATGAAATTTTAGTTAAATATAATAGTAAATTAAAAAAATTGAATAGAAATTATAATAGTTAATAAATATATAAAACTGAAATTGAAATTAAATTCTACATTTAATACTTTTATTTTCCTCTTTTCTTGGTTGTGCATCATTCTTTGTATTCCCACAAATTTTGACCATGTGGCATTTAACGTGGAAAATGCGAAAGGGAAAAAGGGTGAATTCCATGTATTAGGGGATGCCAATTGCCAACAAACTAAACTGTTTTGAGATTGATATCTGTCCACCTACCTACCAAATGATCAACCATCCTACCAATATATATATTATATAGCAGCAATAAGCTAGTGAGATAGAAATGCATGTGTTGTGTTGTGCTACATATAAATAACTCTTGTTCTCCCTCTAAACACTCTCTACCCTGCCTCCAAAAACTTGTTGTTTGGCACTTCTTCACACTCAGCTAGCAGATCAGTCACTGAAGTGGATAAGA

>GmALDH2C5

AAAATATTTGAAATTTGATTTTAGAAAAAATAAATTGATTGTTAAATATTTATTTTTAACTACTTATATATCAATAAATTTATTATAGCACATGTGTCTACAAAAATTGTTAAGAAAAAAATATTAAATCATGTAAAAAATTTAAAATAAAATTAGCTAGTAATAAAAATTCTAGGTCTTTTGATTAAATTATGCAAAAATTCTAAAAAATTTAAGTGACGGGGCAGGTCCGGGTTCGAGGTCGGGGTGAATGTAGTAATCTCATACTTGTACTCGTACCCGACTTTTGGTTATCAGAAAAAATCTGAACCCAAATCCATACCCGGTCAACTCGGGTATTACCCATCAAAGTCAGAACGGATTCAGACAGATACCCACGAGTACGGATTTTCTTGTCATGTCTAGTTCAATATTAAATTAAAAACTTGCAAAAATACATAAATATGATCGATGCTTATAAAAATAATTTTAAATTATCATTTAATTTAATAATGAATTGTCACTAAACTTTTTTTTTATAAAAAAAACTATATGAAAACATACCTAACCTAAGAATTCCTAAATAATTAATAATATAAAATTTATATAAACTCTTTGTTTTAATAATTAAAAAGGCCACGTGATCAACACGTTTCCCAGGGCTACCATACTACTTCCTCCTACAAACTTGCTGAGTTGACCCTGCCAAAGGGGACATATCAAATAAAAAAAAACTTGGTAATTACAAGAAACGAAAAAACTTATTAATGACCACGATTAAATTAAGAGCAAGGGATCAAATTAGTGTAAGTAATTAAAGGTTTCGCACAGTTTCAGCCTCATTCACCCTCCTAACCTGGTCCTAACTTGTTGGCGGTGTTGCCCCATAAATGATAAATATAATTAGGTTAGGTAGTGTACGATTATAGTTTTTAAAAACAGATTTTAGGATAATAAAAAACCCAAACCTTTACGATAACAAAACTGAAATAAAACATGCTTTAAGTATATTTATCATTATTTTAATAAAAAAAATCTTCTCATTTTATTTCCTCTTCTTTTTTTCTTTTTCATTTTTTTTGTCATGTCTCTCTTTTTTCTACTTTTGTAGTTTGTACAGTAGATAATTCGCTTTAAGTTTACTCTTTGCCTTTCCAAAATAGGTCTGTCCCTGGTAGTTCTAACTTCTAATTCAGAGGAGCAGATTTCATAATTGTCATTCTCCCAAATTTAAACATTATATCCAAAAGATTCTCTGTATATTATACCTATGATGATTTAAGTAATAAATTTACTTATAAATTATAAAACTTACTTTATAAGTATTATAGACATGGGATTTTACATAATTGCCATCTAATGTCGATTAATACGTGAGCTCCTTGTTTGTTTTATCCTTTGATGATATTTTATCGCATATATAACAGTGGTGCTAGCTTAGTCTGTTTGTATTACAATTAGTCTCAGTCCCCAACGACTGGTTCTGTTTTCTCTCTGCTGCTTCGATTCATTCACACAACC

>GmALDH6B2
AAACATGTATATTAATACAAAATAAATTTCAACTAAATTTATATCTTTATCCTATTTAATGACTCATTTTTCAAAATATCTTTTTATGAGAGTGTATTCCAAAATATTCTTTTTTTTTATATTTATGATCTCGAGTTTTAGTGAGAGATAATTTAACAAAATAAATTATTTTTAAAAAAATAAATGATATTAAGTAATTATTTTGATTTATATTCACATCTAAAAAATAGTATTTGCTTTAAAAAATAATAATAGAAATTTGTTATTTTCAGTTTTGTAAACATATTATATATACACACATGTATAGAAAACTGAAAATAGCGTGCATCATTTTCTGCAAAGTGAGTTTCACTCTACAAATTACCCTCATGCTCTATTTTTTTAAAAAAAGAGATCAGCTATGCATATAAAATAAAATACAGTGACAGTTTTATAGAGAGATAGTGAAATAAATGATATTNNNNNNNNNNNNNNNNNNNNNNNNNNNNNNNNNNNNNNNNNNNNNNNNNNNNNNNNNNNNNNNNNNNNNNNNNNNNNNNNNNNNNNNNNNNNNNNNNNNNTGAAAAACGTCCACAATCTATTGCGTATATTCACATGCCAACGAGTGTGTTACAATTATAAAGAGACAGAATAAAAACTTTAAAAACCTAAAACATATTATTGTGTGTATTAAAAAAAGTCACTTATTTTAAAATATATATATATATATATATATATATATATATATATATATATATATATATATATATATATATATATATATATATATATATATAACTAATAAGTTAATACTTCTCCTATCTTGAATATAAATAAAAAAACATGTATATTAATACAAAGTAAATTTCAACTAAATTTATATCTTTATCCTATTTAATGACTCAATTTTCAAAATACCTTTTTATGAGATTGTATTCCAAAATATTCTTTTTTTTTATATTTATGATCTCAAGTTTTAATGAATGATAATTTAACAAAATAAATTATTTTTAAAAAAATAAATGATATTAAGTAATTAATTTGATTTATATTCACATCTAAATAATAGTAATTGCTTTAAAAAATAATAATAGTAATTTGTTATTTTCAGTTTTGTAAACATATTATTTATACTCACATGTTTAGAAAACTGAAAATAGCATGCATCATTTTCTGCAAAATGAGTTTTACTCTACAAATTACCTTCATGTTCAATTTTTTTAAAAAAATGATCAGCTATGCATTTAAATTAAATACAATGACAGTTTTATAGAAAGTTATTGAATTAAATGATATTTAATTTTAATAGTATGAGATTGAATCTGATTTGGCATGTCTATCGATTGATTTGTCCTTTCTAGTTCTTCCCAATATTATATTGATACGCAATATGCTATACGATTACTGACAGAGTCAATGTTTCTGGTTGTGTGCACTTATTAGATTCAAACCTTATTGGCGTCGTAGCAGAACTCAAAAGTTAACATCCTTAATCTCTTCAAACACGGTTGTTCATAGCAT

>GmALDH10A2

TTGTGATTTAATGAATGTGTAAAACTGTTTACACCGTTGATGCATATTCCTTTTCTCTTAAAGTAATTATAATAAAAATTAATAAATTTATGATATATAGTAAGTTATAATTGAAGAGAAAAATGGTTATATATTAATAATATAAAATTTTTTATATAATTATTTAATTATAATTCATCATGTATAATAAACTTCTAAAATAATAATCTTAAAATAATTTAAAATATAATTTATAATTAGATAAAAATATAAAATTATTTTAAATAAAAATACACACGTTAAATTCGTAATTGAATGAAAATGTAAACTCATTTAGTAATAAATATTCGCCATATACCGTCCACGTGCTAAAAACAATCTTTTGTGGCATTGGTTTCCGTGCTTCCATTTTGAAAGACGCAGTTCAGAGTCTAGACGCGTGAACAAAAGATACTATAGTACTGCTATTATATAAAATTAAAAATATTATACACTCAGCCAGGATTTCTCGGCAACAAATAATGTGACAGGAGATATGGTTGGGGTCTCTGTATCACGTAATATGAGGCTATGTCCATTATTGTGGGCTAATCATCATTTTTTTAATCTCTAATAATTTATTTTATTACTTTGAATTTAATGATATGTTAAAATTTTATTTTAAGTGATATATTTACTGATGATGTGATATTGTCACTCTTCTAATATATATTAATTATTAGTTTAATAGCAAAAGTTTAAAATAAATTTTTTAATACGTAAGAGATTTAAAATAATACTTTTTTTATTAAAAAATTAAAATAAAATCAAATATATATGAACTTAAAAGTTTTTATTTTATAGAGCCAACATTTATGGCAGCAGTAGATCCAGAATTAGAATAGAAAATACTTATTAAAGTGGAATATATTTTTTTACAAAAAGTGGAATAATTTTGCCCCCAATTAATTTCCACACTTGTTAATAAAAAGAGATATAAATATAATAAGCTGTATAATATGATAAGAAAAGAAGTGAAAATAATTTTAAAAAATTTAAAAAGTGTTTAAACTTTAAACTTAACGAGTCTTCGTGTATAATGTGATTACTCTTTATTTTTTTTTTGAAGGTGTGATTACTCTTTTTTTGTAACAACTTATTTTATGTGTTATTTGAGTGAACTTTTCAGAGAGTAATTAACAATCTGTCATTTATAACTTGAGACATATATAACTGCTATAAGCGTAAAAATTTCAATCAGTCCACCAATCAGAACATGGACAGAATAACGTTACAAGCCAAAATCTTACCAAACCAAGAATCGAAGAAACTAGAAGAAAAAAAAAAGTCAACAAAAAAATTGGTACCGACCACCGACATAAAAAGCAAGAAAAAATAAAAAAAGATCTGTGATGACTCATTAGCAAGAACAAGACGTAGCCTCAATCTCATTATTTATATATATTATCATGTAGCTGCATTATTATTGATCCAATCAAGAATACAATAACATAGCATAGAGTGAGAAGTGAGAAGAGAAAA

>GmALDH18B1

TACAAGGTTTCACCAAGGTTTTTAAACTTTCATAGTATTATGTATAAAATTATTTTATGGTCCCCTCTTTTTTTTTCCTCTAAGTTCTCTTTAATTTTTTTTTTACTAACAATGTTTAATCTAAAAGTCAAAATTCTCTTCATTTTTTACTTTCTTCTATTTGTAAATTATATTTAATTTCTTGTATACTCTTTCGACTTCTTATATACTATTTTGGCTCTGGCAAGATCACAAGTTTTTTTCATTGTTGTTGTTCTTCCTACTTTGTGCTATGACTTTCTTTTACTACTAAAATTTTTCATCCTGTATTTTTTTTCCTTACATGTTATTTTTTTAATTGGGTCTTATAAATAAGAATGCAAGAACAAATGAAGTATTTAAAATAATTCTGTACCTTTTATAAAATTATTAACATATAGAAAAACTAACTTCGAACAAATAAAAAAATTGGTTTCTTTTAGTAATATTTTATTAGATTTATAGTTTCTATTTTTGTTAATGATACATCTATACTTAACTAATTTCTCTAAGCATTTATCATGTTTTATTCATTTAATCAAGTATTTTTTAAGAATTTTTCATGTATTACTCAAATATTTATTTTTGAAATATTTGAATAACATGCTTTGACTAATAAAATTTTCTTTTCACTTAAATGAAAAATTTATATTTTCTTCTTTTGCTATCAGTTGTTGAACGCTTATGTATTAGTTATACTTGTAGATTAATATCTCATGCTCAACACAATTTATATATTTTTTTTTGCTCATAAAAGTATTAGCTATATATTAATTTTTTGTAAATGTCATGTATGCACGCTTATTAAATTTGAATATTTTTTTAAGTCATGATCTTTTTATATTGGTTACCTTATCTAAAATTCCTACTCATATGTCTTTCGAAGACAATCTCTTATAAGTTACCATCAAACACTAACAACATATTTCAAAACACAGTAATTAAAAAAAATCACATTCAAAATGTAAAAATAATAATTATTAATTTCTAAAAATTATATATATTTTAATTTATAAAAATCAAAGACAATAAAAAATTTATAATATTTATACGACATTTTAATAAATTGAGGTAAACCCATTTAATTCCAAACACCACATCTAAAACATAAAACCAAACATACTTTAAATATACACGAACATCTCTCCAAATAAATTTTTCATTAGAGCCAACCACCTCAGTATCCTTTTTCATTAGAGCCAACCTCCTTAATATATATATATATATATATATATATATATATATATATATATATATATATTAATTAAGGACTGTGCTCAAACTCCAAAGTCATTGCAGATGCCGTACTACTAAAAAATTCTGGTGATTATTAAACGCAGAAAAAAAATTAAAAACTAAAAATAAAAACCCTCAAAACACTGTGCATGCCTTTAAAAGCCCTTGTCATTTGAACAGAACTTCACACCAGTGGCTTCTTCATTCCTTTATTCATTCATCTCACTTCTGAACACAAGAGAAACA

**The 1500 bp upstream sequences of 14 down-regulated *GmALDH* genes in response to drought stress.**

>GmALDH2B3
CGTGAATTAATTATCCACATAAAATTATTTTATGATCACTTGGTAAATTAGTCTTGAGTTGTAACAGCTTAAATTTTAAGAAGAATTTTATCATTTATAATAATCTCATTCGAATAAATAATTTGGTTTTAAAAAAGAAAAAAAAAACTTGTGTTAAAAATTAAAATGTTAACTTGTTGACTCATTTGCCTTGTAAAAAAAACTGTTTACTCATTCTCAAGAATCTAAACATATATCTTCAAAATCCAAAAGTTTGGGCAGTATCATCCACAAAATGATACGTCAAATGCTGGGCTCACTGTTAGTCCATATATTTGTTTCATAAAATTTTGGCAAAAACGACAGTTGACGGTTACATACAGAACTTAAAAGTCAAAACTAAAACATTGGTATTGTGTGAAGCTCCTAATCACCAAGTAGAAGAAGCAGAAAACACCCTTTTGGAGATATTTGCTTTGGCCATTGGTCATGGCAACTCGAAGACTTTCCTGGCTCCTCTCTCGCTCTCTATCTTCTTCCCCTTCTTTTCAAGATGCTTCTCTGCTTCACTCACTTGATAACCAACCTATTCTCTTTCCCTTTTTTTATTGGTAAATGTTAATTTGTTAGAAATATTAGTGGAAGGGATTCTAACTGGCGACCTATTCCCTTCCTTATATTCTTCATTTTGCAGCTTCCTCTTGCTTTTTGGTCACTATTACTTTATGGGATTTGTATGGATTTGGCATATTTAAAGTGGTGGATGTACTATAGTGGATAATCAGGATAGCCAAATGTTTCTCAGTTTGCATGTTTTCTGCTTCTCTGCCTTCATCAAAGTACAGGACACAAACTGTGTGACTATAGTACTGCTATGCTTAGCACATGCACATATAGTTAGGGTCCATTTTTAAAACCAACTAAAGTTTTTCTTTTGTATGTTTGGATAACAGCAAAGGAAACACTTAGAAACGTACTACTATTTTCAATTGTTTGGGTGGAGAGGAAGTATAAAAAAGAAGAAAAATTGACTTTTTAAAACAAAAATTCAATTTCTTTTCTCTTCTATTTTTCCATCATTTCAACTTTATTTCTTAATTTTTTGTCTAGCATACCAATCAAACCATTTATGTTGGTTAGGATATGGAAAGAAAAAGAGGGAGAAGAGAATGTTCTAAGTTGTTGTTTTTAATATTTCAAATATTTGTCTAGAAAATATTTGAATTAGCTAGAGAATGAGAAGGGAAAAAGTTCTAAGTTGTTGTTTTTAAAAATAATTTTTTCTCTCTATTTTCTTTTCACTTTTTTCCATCAAAACAAAAAAGATATATTTTTTATTCTATATATTCTGTCTTTCTTTCTATTCAAACATAGCTAGATATTTTCTTTCTATTCTGTCATTTTTTTATTAACTTATTACCACCTGCTTGTAATTTATTTTTTTTGCTTTTTAATTTTATAATTTATCTTAGTTTGATTTCTTATATAATGAGTGCTTTTCAGTGACTATTACTCATCAGA

>GmALDH2B4

GCCTACTGTTCAATTAATTAATTCCTTTTTAGTTTTTTTTCCCATTGTTTTTGTGTAAGTTGAAATCAACTGATTTATAATCCCAACTCCCAAGAGATTCTAGGAAGAATGAAAGGAAAATATAAATAAAGCATCCTTTTGCTGCTGCATCCAGCATCATTTCATGTTTGCTTCTCTACTGCCCATATCTTTTCGAGTTATGAAATTTACACACTTATTTAACTAATTATTGTTTGCATATGTATATGTACACAATAATAATAAACTAGTAAATGTTCTTGTACGATGCAGAGGACAAATTATTCATGGGATAATCTTTTTTATAATTTAAAAGAAAAATAAAACTTGAAAAAAAATAAAAATATATATAACTTTATATGGTGCGACAATATCAATTAATTAATTTTAGTTTATCATATTCAAACAACCTCTAAGTTATAATTTTATTGAAAACACTGACGAACTCTTAAATCTAGCATGTTCGAGAAAATATTTTTGTGAAATTTATTATTTATTAAAATTTAATATTTAAATAATTGTATATAAAAAAAATCAACCCCTTTTATTGCATTTCTAGATCCATTCCTGATTTAAATTATATGTTTTCCTTAATAAAAGGATAAAAAATGGCTTCTGAACAAAAAGTAATTTTTAGGCCTATGAAATGAAATAAGAGCATATTCTTGAACTGAAGAATTAACGATAATATAGTGTCACCAATGGATGTATATAATTATTCAACTCTTTTTGTGATGGGTTAATACCGTGAACGAGGAAAGATACATACTGTACATAAAAGAAAGTAGTGAAGACAGTCGAGAGGGTAATAACGGAACTTTTGAATTCCAAAAAACATCCTTTTTTATGTGATATTAAAATCAAGTAAATATTAATGAGTATTTTAAAAGTAATAGAGAAGAATTTAAAAATAAATAAAAGTTTGAATATATTTAACTTCAATATTAAATGCTATTGTTGAATAGCACTAAACATACATGTAAGTTAACTTCATGAAATTATTTCAATTTAATCTTAAATCAAATTTGATACTTGAGTTGCAACTACATTAAATATAATATTAAAAAGAGTTTTATAAAAAAATTTATCCGTGAAAATTAAAGATAACATTAGAGGTTAAAATAATTTATGCACCATATTTAATCAATTGAACTAAATATATAATAATCCTATCTGAGTAAAAGATTTGGTTTGGAAAAAAAAGAAATTAACCTGTGTTAAAATGTTAAATTGTTGACTCATTCTCAAGAATCTAAACATATATCTTCACAATCCAAAAGTTTGGGCAGTATCATCCACAAAATGAAACGTCAAATGTTGGGCTCACTGACAGTCCATTTATTTGTTTCGTAAAATTTTGGCAAAAAGGACAGTTGACGGTTACAGACAGAACTTGAAGCATTGTTATTGTGTGAAGCTCCTAATCACAAAGTAGAAGAAATAATAAACACCCTTTGGAGATATTTGCTTAGGCCATTGGTC

>GmALDH2B7
TTGTATATGCTTTCTTCTGCTTCACTTGTCTTATTCCCACTTCTGCTTCACCTTCACAAAATTTTTATTGCCCAACTGAAATATAAGCAATTCTTGTATGAGTTATCTTTAGACACAGTTTATTAAAAACTATGCTATCTGTAAACACCATGGCAACATGAAAAATTAACCGTGCAAATATAGTATCCCAACTCGACTCGGAAAGGCTAATGTATAGTCAAGCATTGCAAATTTCTATATCATGTCTTAAATCTTAATAATATTATCTCCAAATCAGCACTTTAATATCCTTTTCAGACCCCACAAGTATGGACAAAATGTCATCCATAAAATGACACTTTCTTGCATTGCTTTCTTTTTTACTACATAATATTGATTGATGGTGATAACGTTTAATGACACAAGGTACAACCATTTTTTATTTTGAAAAGACTCTTTTATCATTCATAAGAATAAGAACAAGGAGGAGAAGAAAAAGAAGCAGGGAAAAATATTTGACTTTGATGATGGCAGCTCGCAGTCTTTGCAGGCTACTCTCTCGCTCTCTCTCTTCTTCCAATTCTTCTGGAGCTGCTTCTCTGCTTCACTCACTAGGTGATCAACTTAGTTTCATTCTCATCTTTTATTTGTTAATGCTTAATTTGTTTCCTAACCCTTTATGAATGTTGATGATGGCCATGAGTTTCCCAGATTGCAAAGCATATGAATAATTTGACTAGCACTATTCACTTTTGTTTAGCACACCAAAACTATTGTGATATAATGAATCTTTTTCATTACTTTCAAGACCAAATTTTAAAGTATCTTCTGATAGCGGTTATTATCCACCACTTGTATTTCTTTATATATTCATTTTGGCAACTATATTATCAGAATACAATCTTCAATTTGTTGAATAAAACTGCCACATGATTTCTTTGTTTATCCCAGGAAGAAACCCTGGAGGGTGGAGAAACATTAACAGATTTAGCACGGCTGCAGCAGTTGAGGAACTAATCACTCCACAAGTTTCAATACGTTACACACAGAACCTGATAAATGGGCAATTTGTGGATGCTGCATCAGGTGTGTCTAGCTTGAACAAGCTTTGTTATAACATGTTTGACTTCTAGTTCGAACACCCCAAACCTCACTTTGTTATAACATGTTTGGATTAAAATTGGGAAAGTATTTTTGTGAATGCTAATGCACATTTGCAAATTTCAATACACAAAATGAAGAGGACTAATTTGTTGCTTTGGGAGTAAAAAATACTTTTGAACTCTCTGAACTGTAATGCAAACATGCACTAACTTGTTTAACAAAATCATGGTTAGGTTGTGTACTTGTGTTTGAATTTGAACATGAATGTTGCGTCTCAATTCATGGATTGAGTGGAACTGAAAAGAAGTTAGCTTTTCCCATTTGTAAAATCAATTGGTGGACTTTATCTACAATTTCTTTTGGTCTCAGGGAAAACATTTCCAACATATGATCCGCGCTCAGGAGAAGTGATTGCTA

>GmALDH2C1
CCCAAATAAATTGATTGGTTAAGGTATTTCTCATGATTTTTAATTTATTGTGATAAAAAATATTGTGTTAATTTTTTATATTTCAAATATATATTTTCCATATTGTAATATTTTTCTCATTATTATTTTTTGTATATTTATCGTTTCAAAATAAAATTATGAATAAATTATTTTTCTGAAGCAAAAATAAATAGTAAACTATAAAGATGTGAAATAATGAGACCCAATTTAAGTTCTCAATTAGTTCAATATTAGAGTAAAAAACATGTAAAAATACATAAATCTGATCAATGTGATAGTGTGAGACTCGAAGAAAATAAATTAAGAATTTAAGATTGAAATTTTCATTGCAGATACTTATAATAATTTTACATTATCATTTGATTATAAATTATTACTAAATTTATCATATTTTATATAAAAAAAACTATATGAAAACATACCTAACCTAATAATTCCTAAATAATTAATTAATAATGTAAAATTTTATATAAACTCTTAGTTTTAATAATTAAAAAAAGCCACTTAATTTAAACACGTTTCCTGCGTTGACCCTGCCAAAGGGGACATATCAAATAAAAAAACTTGGTAATTACAAGAAACGAAAAAACTTATTAATGACAACGATGAAATTAAGAGCAAGGGATCACATTAGTTTGCTACAATAATTAAAAAGGTGACATGATTACCAGGTTTCCCACTGTTACAATTCTCATTTCACCCTCCTAAGTCCTAACTTGTTGGCGGTGTTGCCCCAGCATATGGAAATGATAAATATAATTAGGGTGTGAATGATTATAGTTTTTTTATAAATATTTTTTTAAAAAAACACTTTTGACCTTTAGGACAATAAAACTGTAATCAAACATGCTTTAAGTATATTGATTTATCATAATTTTAATAAAAAAAAATTATCTCATTTTCTTTCCACTTCTTTCTTTCTATTTCATTTTTTTTTTCTACCTTTGTACAGTCCAGAATTGGCTTTAAGTTTACTCTTTCGCTTCCCAAAATAGTTCTGTCCCTGGTAGTTCTAATTCATCAGAGGAGCAGATTTCATAATTGGATCTTTCTCCCAAATTTAAACATTACCCAAAAGATTCTCTGTGTATTTTTTTGGTAATGATTCTGTGTTCTCTGTGTATTAAATTTAGATATCAAGACTTTTGTTAATATGTGATTTAAAAATAAGTATTTGTTTATATTTTAATTAATACTATATAACATGATAAGATAAAATGAAATACAGATAATAGAATATTAATTAATTTTAAATTATATGAAACTTGATAATTTAGTCTACCTATGACGATTTAAATCATAATTTACTTATGAAACTTAATTTATAATTATGATAGACATGGGATTTTACATAATTGTCATCTAATGTCGATTAATACGAGTGGTGCTAGTTCTGTTTGCATATTATTACAAGTCTCAGTCCCCAACGACTGGTTCTGCTTTCTCTCTGTTGCTTCGATTCATTCACACAACTCAACC

>GmALDH2C2
TAGTGTCAGTTAAGTTTAGCTTATTAAAACAAGAGGGATGATTCTCCAAAGAAAGAAAACTTCACATTTACATTTTTTTCCCTCGTTTAGTAATGTCAAAGTATGAAGGAAAAAGAAAAATCTATATGGATTCCATATAAAAAGAAATGTTTCTTTCCAAATCGGAAAAACAAAGGAAAAGAGAAGAGTGAAAGCTTTGTTAACGTAACAAACAAATACCTCGTTTACATCAAATTTCATTTTTTGTTTAGGGTATTTATTTTATTTTATACATATTTTTTATTTCTTTTCACTTTTTATATAATCAAACAAGAGGAAAGAAAATTTTTACCGTTCTCTTTTAATTTCTTTTTCCTTCTACCAGATAACTTAAAAAATTACTTCACTTTCTACTTTTTTTTTCTTTTTTTCTGCTTTCTTTTCACTTTCTTTCCTAAATCAAATATAACTTTTGTAAAAAAAAAATTAAATAAAACTTTAAGGTGAGTAATTGACAAAATGAACATTCCTCCATTGCATAATACCATCGAACCCTTAGAAGATCTCCATTGTTGCATAATGTCATCATACCCAAGAAGACCTCCAATGCAGAATATAATCATTCCATATTGTTGGAATTGTAAAGCACTATACTGATAGTGATAGGTAATGTTTGTCCTTATGAAGAACTTGTTCTGTTACAAACAGCCTTATCATTCGCGACATCCCATGCCAACGCATGTTGAGCCTTTTGGAGAAGCACCAAATGAATTCTGTAAATAAATAAAAGGCAAAATCACCATTTAAATTATTGATTTAAATATAATTTCCATACCTGTTGATTTTAATTTTATTATCTAAAGTTTACTTTTTATTTTATTAGGTAGAATACTTTTACCTTTAATTTTAGTATTTGTTATAAGATAAAATTTGTATAATAATGACGTGACATTTATGTGTCACATTATTACTTAGGTGGTGATATAACATCTTATTAGTCTGCACATTATAATTTAATAGATTTATACTTTGCTGATTAAAAAATAATTTAATCGATCTAACTAATGGATAAAAATTAAAATCAAACGTAAAAATATTTCATTAATGAAATTTGAATAATAAAATTAAAATAAATTATATTACAGGTTTAAAAAACATGTTTAATCCTAAATTCGTTTACAAAAATAGAAGTTATCCGGGGTTAGGTGGGAATGATTTAATAGTGCTTATTAATTTACGTGGTGTGCCTTAGCAAGAACGGTACGGATTTTCTCCACCTAAATCCTAAAGCTGCACCATGCATGTTCTTGTCTACGCACCTTTGCAGAAGCACTATATTAATTCAAATAAAAGGCAAAATCACTATTTAATTGTTTAAAAAATAGAAATTAAATGGGGTTAGGTGGGGAATGATTTAAAAACAATAATGATAGTATTATATAATTTATTTTCTCGTACGTTCTGTGCCTTGAAGAGCACTGATTTTGTCCACCAAAAGTCCTAAAGTTGCGCCAATAATTC

>GmALDH2C7
TATAAACAAAAACTTTATTTTATTTCCTATCAAATAAATAAATAAACTATACTTTTTATTTTCTTCAAATCATTATTTTAATAAAAGTTACTTTTATTTATTTAACTATAAAAACCTCATCATTTTTTAAAAATTCTATTTATTTTTAAATAGAAATATTTTTTAATTTATTTTAAAAAAATGAAATGTTACACATGAACTGTATATAGTTTTGGAGGAAAACTTATGTTGTTGCATAGGGTTAATGGTTAAAGGATAAATTACACTTGCACCCCTTATTTTTTTTATTATACACAATAACATTTTTTCCTATTCATGCATTAACCTCCTTAATGTTTGAAAACATTACATTAATATCATTTACACTAACTCCTTAATTTTTAAAAATATTAATTAATATTGTTCTCGAAAAAAGCTTCCTCGTAACGAAAACGACCTAATCTAAAACACTAAAATGAGGTGTAAAGTTTAACCTATCTTCCTAAGTCAGAAGGAAAATTATTTATCATCGTCAATTGGTTGACCTCATTCTTATATTCCGGGTTAATTCATTTATAACATATCAATATATTTCATTGTATTCTCCTTGATCTCCGAGTATAATTGGGTTTAAATTAGTTAAATTTTTGTAAGTGAGGTTGTTTTATTAGAATATTTCTTTTAATTCCATAATGTTCACCTCAGGTTGAATCAATATCGACATTATAGGATATGACTCGGATTATAGTCCCATGATTATGTCCACCCCCCATTAATGGGATATGACATGTAATAGGTATCAGGCTTCAATTCGATCTAGGTATGTGCACAAACCCCCTATGCACAAGGTGTGTAAAGGCTATAAGCGTTCTGACTTCTGATCATGGTCTAATCTGATATGTATAGATACTCGTTTATATATTAAACCAAGGCCACCATAAGAAAAGTTGCTGTAAATATTACACAATGAGATATATTGTGTAATTTGAGAAAATTTTAAAAAAATTAACGTAATTTGCCAAAAAAAAATAATGTGACAACTAAGAACTCATAATTATTATCATAAATTAAAATTATAAATTTCAATCAGACAATCATACTTGAATGGTTTTTTTTTATTTAAAAAAAGTCATGCATGAATGACCAAATTAAAAATTTCTGAAAATTTTTAAAATTGTATATTTACCTTTTAATTTTTACCATTAGTATATAATTCAAATTTATGATTTTTGCTTCTCACAATAACGATTTTTTTTTTTTGCTTCATATGCTTCCACGTTTTAGATCACAAATATGCTTTTATCATGGCATGAGTATAATTTTACTAACAAGTTTGTCTGAAATATAAAACTATTTCTTATTGGGAAAAAATATAAACTGTCTTCCCACGTGGTACAATAATAAAAACACAAAAGAAAATTACTATGATGTAAGCCCTCGTTTCTGCTCTGAAGACTGAAGTTATAACCGGTGAAGTTGGTTGTCCTCACGCCATTCATTATTGTACCAGGCTCTTCATCA

>GmALDH3I1
GAACTAAAGTGGAAGGTAGAGACGTAGATTGAATATTAAATATTTATTCAATTATAATAAAAATAGCACAATTAAACAAGTGTTTCTTCTTGATTGTGGGTTGCAATAAATGACGCATCCACACTTACTTGCTAGATGCACATATACTCCCTCACTTTTAAATATTTATTCAATTATATATTTTGTCGTCCTTAATTTTGTATATTTGAATCTTCTATCCCAACTTTTTTTTTAATTTGAGTAAATTATAATGAATTTAGGAATTATTAGACATGTATTCCTGATTACTTTTGCTTCCGTGACTGAGGGTGTCAACTTTTATTGAAATGGCGAAACAACCCTTATGACTTAAGCCCCCTCATTTGTTCTCACCTCTGATCTTACATACTTTGGGTATTTTTTTTGTTCTCATTCTTACATTTGTGTTTTCTTATCCCTTCCTTCAAGAAAGCTGATTTATTGAATTTGCACCGACATTTGTAACAACTACCGCTCTACCGCCACCGTCAATACCTTTCCCTAATTTATTATGCTTTTCATATTTTATTATATTTAATGCCGATAAAAAAAAGGCAATAAGAAACACAAAATCTTGTATCGATTTCGTGTATGAAGATTTTGAGTTATTTGAATTGATTGTTATTCTCTTCCTGTGTTACTCATTTTTCTTTTCTTTTTTCCATTACAAATGAGGAAGACAATGGTAGGTTGGTCCATGTATTTCATTAAACACGCATGGGCAAAGCACATGACCCCTTCTAGTTAATAGAAGCCAACGGTGTTAATGTGCAGGGTTGTCTGTGTAACAGAATGATAAAATTAGGGGAAGTTTACTTAGGAGTAAAATAACAAGGGAATGTAAGTGTTATAAGGCCTAAATATTGGAGTAGTCAGAGTAATTTGCCTCTCTTTTTTTATTTTTTACATAAATTGTCTCTCATAGATCCTAGATTTACCACTCAATTGGGACAATTTTCAAACGTGTCTATGTCTATTGTGTTGGGCAGGTAGACAAAACAAAGCCACTGTTCTGTATCGTGATTGTCACTTTACTTACACTTTTGTCTTTAACTAACATAGTACACTAATTATTGTATGTACTAATGTATTTTTACTGGCTTTTATCAATTGATCCAAGGGTTGTGACATATAAAAGATGCATGCATGGAACGGTTAGGATTTAAGAGACCCACGTGAGTTCGCGTACGACATAAATTCCCAGCCACTCCATTACCAAACTTGACTTGAGTGCACAAGATTTTTTTCTCTCATTGCTAAACTAAAAGTCTACAACGTTTCTTTTATTTTTGGCTATGTCCTTGTCAAATTACCCATGATTAATACCAAGTCTTTAAATGTGTTAAACATTATCTTCCTATTTTTCATGGCTGAGAAATCATTCCATTTCCGGCTTATATATATACACATAAATGCAGCTTCTTTTATTTTTAATATTAGACATCACTACCAAAGATCTTTCTTTCAGCTTCTTGTTCTACA

>GmALDH3J1

AAAACGACCAAACGTAAAGTGCAGCAAGTACAATCAGCTTGGACACGAAGCTATAATTTGTAAAAGCAAATTTCAGCAGCAAGAAGTCAATGCCCAAGTTGTTGAGCAGGAGGAAAAAGATTATATTTTTGCTTCAACATGCTATTCGATGAGGAGTAGTTGTAAATGTTAGTTGATTGATAGTGGTTGTACGAACCACATGACATATGATAAGACTCTATTCAAGGATTTGAAGCCAACTAATGTCTCAGAGGTCAGAATTGGGAATGGTGGCTATATTCCAGTAAAAGGAAAAGGAACTGTTGCAATTTCAACGTGTTCAGGTATCAAATTAATATGAGATGTTCTTTATGTACCTAACATTGACCAAAACTTACTAAGTGTAGGTCAGTTGATTAAAAAGGGATTTAAAGTGTCCTTTCAACATCAACATTGTTTTATCTATGACATTGTTGGCCGGGAAGTTCTAAGGGTTAAAATGAAAGGTAAAAGTTTTTCATTTGATCCAACAGAGGAAGAGCATACAACCTATTTCACTCAAGTCACACCCACAGAACTCTAGCACAAGAGACTTGGTCATTGCCATCTTGAAAGAATGCTAAACATGAAAAAAAAAGAAATATGCAAAAGAAAATTTGAAGAAGTTTCAAATGGAGGAATGCAAATTTGTTAGCACACCAATGAATCAAAAGGAGAAGTTCAGCAAGGAAGAAAGTGTTGATAACATTGATGAAGGATATTATGGGAGCTTGATTGGATGTCTAATGTATCTCACTACAACAAGGCCAAACATTTTATTTTCTCAAAAGAAAAAAACTGGAATTTTTGTTGACAATCAAGTAGTCATTGCTATTGCAAACAATTCCGTGTGTTATGGGAAGACTAAACATTTCAATATTAAAGTCTATTATTTGATAAAGATGCAACAAAGTGGAGAAGTGAACTTAATTTACTGTAAGTCTAAAGATCAACTGGCTGTCATGTTTACAAAGTCACTACCTATCAACAAACTTGAACTCAAAGAATTGGAATTTGCAGTTCCAAAAGTAAGGAGGAGTGTTGAAGATATACTTTTGTAACTGCATAATATTTTATTTTTTTAGTTTTTAAAATAGGATTTAGTAAATAAGTTGGTTTCCTATATTTTAGGAGTAAGTCAGTTTTAATGGATTAGCCTAGTCAATAAGTGATTTCTATCTTTAAGGAGTAAGTTAGTTTTAATATTCTGTTTTACTAATATCTTGTTATCTAATTAGTTTATCTTTTGTTATTTATTGTATTGCTGCGGAGAACAATTTGAATGAGAATAGAATAATTCCATTTCCTCCGCATACGTATTGTTTCTCTTATCTCCTCTGCTTTTTTATAATTTTCTTCACAAAACCAACACAAGGGACTCTAGCTTTTTCCTTTATGAAAGCCGAAACTAGTTTACAAATTGAAGTGAGTTAGTTGAACAAATATAAATACTGGTTTTGCTGTGAGGTTAAGCAGCACCAT

>GmALDH3J2
CTGCAAACTACCTTCAGAGCTTTAACTGCACACCATGTGCAATCAATCCATCCACTTAAGAGCCCCGGTTTTACCGACCAACTACACCTCTTTGGCTCTTTCCATTTTGACACTATGGCTCCTACATCTCCATATATATCTGTAGCATCCAACTTTTTTTTTCTTCAAAAAAAAGTTTAAGTGAGATAGATTATTGCAGCTCACTGTGACAAGTCTCAAAGTTGAATAAATCTTTATAAAGGTATTAATGTCCTCACTTCAATGGCACGAATCAAATTTGTGTATTTTTAGGATATGATTCTTATCAGCCAGAGAAATCTTTGTTGGTTGCATTTTAATATAAGGATCTATTTAATTTAGAGGATGAAAATAGTGATAACGAAAATAAAAGAAATTTTTTTGAACTAAAACAAAATATAAAAGGTGTAGATCACAAAATTTTAAAATTTCTCGTTGTTATTTTTAGGATAAATAACTATTTTCTTCTTTAAATATTTAGAATACTGATAAATTCGTTCCTGAGAGATAAAAATTTAAATTTTAGTTTTCGAAAGTGAAAAAAGTATAATAAATTCATTCACCCATTAACTTTTATCCGCAACGAAAGAGCCTACATAATATATTTATGGACAAATTTGTCTAAGAATTACACATCCAACAACAAAAATAATTATTTATCTAAAATATAATTTAATTTTTATCCTTTTCTCTTTTTTTAATCGTTACAAACATAACATTACACTAAATACTAAAAAAAAATAAAAATTAGAACAAACATAATAATAAACATCATTATTTATTTTTATTTTTATTTTAAAAACCAAACACAGCTCTGTTAACTATCAATAACCCCTTGATTATAATTGCACGTCCGTACCATCCCTTTTTTATACGTCCTACCGCCCCTGTCTTTCAAACTATAGAAAATCCTAACCCTATCATACAATTATTTCAATATATATATATATATATATATATATATACCTTATTTGTTTGGGTCAATATTTGTCAAAATTTAATAATAATAAAATCTAATTAAATAATTCTTATTTTTTATATTTTAAAGTATATATTAAGTTAATTATATTGTAAATAAATAAGATATTAAATAATTTTAGATTAATATAATTTTTTTCATATATGATTTGTGAATAAAAATTTCAATATAATATTACAGTAGGTTTTGAAATAAAAAATTGTCCCATTAAATTTTTTGAATAACTTAATATAGTATGTGCAAATTATATTAATGTAACTTATAAATATTGATTTTAACATATTTTTTTATAAAAAAAACTTATCCTAAAGATCATGTAAGTACCCCGACAAAAACATGTGAATGACAGAATGCAAAGAGCCCTGGCTTACCTTTATGAAAGCCTAAACTAGTTTTAAAAAAAAAAAATAATAAAGCCGAAACTAGTTTACAAATTGAAGCGAGTTAGTTGAACAAATATAAATAGTGGTTTGCTGAGAGGCTAAGCAGCACCACAAAGCACG

>GmALDH3J3
AGTGGGGCAACTTCTCCATGGCTTGGTATTAAATATAATAATAGATATTCCGTACGATGAGAATTCAGACCTTGCGTTACAATTGTGGGAGATTAATCATTTTTGCTGAACTTAATCCATACATGTAGATTTCAAGAAATATTTTTTAATGAGTAAATATCTAAAAGGAACTTTTAATCCCATTCATCTTTTAAAAGATATAAGTAATCACATAATTATTTAAATAAAAATATGAGTAATTTAATTGTTTTTTATTTTTAATACATAATATAATTAATCTTTAAGAAAATACATCAAATTGACCTCTTAAACATTATTAATAAATTAAAGAATTTAACTAGATAGAATGTATTTTTATTGTAAGTTTTTTTTAATGTTGTTGTTTAATTATGAATTATTATATATGATAAGATTATTATATTTTGTAATAATAATTTTAGAAGACACATGTTGTATGCTTTTTAAATAATTGATATTTTAATGTCCAAATAGCATGAAATAATGTCTTTTACATTCAATAATTAATAGAAAAATCATGAAATATTTTTTTATTATTTATAATTCAAGATTAACTCTAAAACATAATTTCAAACTCTAATAAATTAACACATTTAGTCAATATGAATGACTTAAGAATTTTTTTTCTTTTTTTATTCAATTATCATGGACAAGATCTTACTTATATTATAGAGCTCAAACTTATTACCAAAAGTTGATTGATTCTTTTTAAATTAATCATCAACTTGTGTTTTAAGGTATTAGGTGTCCGAAATTAAAATTATAATAAATAACTTGTTAATTATTATGATAATCTCATATAAAAAAATTATTATATTTCTTCTTGAGAACTTTTTATTCATCAATCAAAATAATATTAACATTAAAAATTCTGAATTGAATGAGTTCAATGACATCCATATGTACATCATTTATATATGTAATTTATAAGTGAAGTCTATTAATCTTTATTCAATAAAAGTCATTACATATATATTCATCCATTCGGATGTTCAATTTACATTAATCCTACAATCAAACATAATTTAGATTAAAATGATAAGGACTTATTTTTCATTATCAAAATTTTAATTAAGAACTTGTCAAATTAACAATTTAATAAAATAATAAATATAATAATAAAATAAAACATAATTCTTTATTAAAATTAATAACATGATATATTAGATTTTAGACATGTATATTTATTTGCAAACTTTCACACAAAATTAAAATGTGTTATGGATTGGACTACACCTATTGTGTAAAATGACTATTTATTCTTTCTATTTCTGTCACCTTCAAAACATTTTCCAGAAGATGATTACAGTGGTGCCCAAATTAATGCTAAACCATAATTTTCTCCATAGAACGTGTCAGGTGAGGACAATTTATTACGGTTGTTAACCAAATAGACATTTGGTGGTGAAGATTATATATACTACCTAGTCGGAGAGAATTGAAGTAGGGCTGTAGGGGTACATCAAATTCTTTATTTATGAG

>GmALDH3J4
CGTTTATTCTGCACGTTAATAGTGATTGGAGTTCAATTGAAGTTTGAAAGCACGGAATTAATTTTTTAGTACATATACAACTACAAAAGTGAATATTAAATATAGTAGTAATTATAGTAGTACGATTGAGTGATTGACAATTCAAAACTAATAAGAAGCAATCAATCATAGCGTTTGGAGGAAATCTTGGCAGTTCTTGATTGTTGTACACCATAATTTGAGAAGTCGGTTTTCATTGAACTATTTGATATCAGTACCGAAATAGAAAAATAGTTTCGGCTTAATAGAAAGATTTAGGAGCAGGGAATTCAAGGCAGAAAAACATGATCTGGACTCACACAACTTACTTGTGCGACCAGTCAAATGCAAAGTTCAAAGTTCTATTTTGCTCCACACGATCAATTCCTTGAAACTACTGGATTCTAGGAGTTCGAGTGTTTAATTCAGAGTCAGAAATACATGCATCCTCATATAATAAAATATAAGAAATATGAATCTCTCATTATAAAATGAAAATTTTCTCTTATATATTATTTTTTTTCCCTTTAAAACAAGTACATCCTCAAAGTGATTTGCATAAAATGTAAATTAAATATTATAAGCAACTGAAATCTAATTAAATAGAATAAAATCGGAAAGATCAAAATTAAGACAAAATTATAAGCTAGTTAATAAATGTAAAATAATAATAAAAAGACATATTATTTGATTATAAATATAAAAATTAAAATCATAATAATTTTTTTATTGAGACAAATTAAATTTTAAAATATTTCTAGGGACAAAAACACATATTTTTTATTTTCTTTTTTGTCAAACATATTATTTATTATATCTATCACTAGTAAAAAGTAATTTTTGTTCCTAAATTTGGTGAAAATATTTTCTTTCTTTGCCAAACATATTATTTATTATATCTATCACTAGTAAAAGGTAATTTTTGTCCCTAAATTTGGTGAGCATTGTGATATTAGTCTCTAAACCTAAAAAATATCAAATAAATTCTTGAAATGTGAAATAAATGATCAAACTTGATTATTTACTTTTATTTTAGTCTTTCAACATGTGTTAATATTATCATTTAAGCTATTTTTTTAATTTTGAACTAATGTGATTATCATTTTATATTTTTAAGGATTAATTAATATTTTCTTATAAGTTTCCATCCTACAGACTATGAGAATAATCTTCTATATGAACTTAATCATACTTAAAAAATTGTCATTTTTTAACCCACATTCTTTCTCACAAAAATGTATATTATGAACTGAATTATACTTATTGGATCTAAAAGGACTATTTATTCTTTTTATTTCTATCAAAACCTTTTGCACAAGATAGTTACAATGTTGACCACAATTTTTTTTTCTATAGAACGTGTCCGGTGAGAACAATTTATTGCGGCCGGTTGTTAACCAAATAGAATCTTGGTGGTGAAAGTATATATACTACTAGTCGCAGTGAAGTAGCTAGGGGCACATCAAATTCTTTATTTATAAC

>GmALDH11A1
TTTCCAATGGTACATTGGGTACCAGCTTCAGTTGCACGTTCTTCTGACTTTTCTTCCCCATCTTCGTAGTATATATTGTACATTAGTATCTTGAGTAGTGTTTAAGAATTATTTTTTATCGTAGCCAATTCTATACTGATCTTTCGGGCCAGACATCCTCAACTTCCTTTATCTGTGTATGAGAAAATAATGATATTTTTTCCATAATATATAAAGCATTGATTTTTTCCACTTTTTTTCTGTACTAACTTTACACCTTTATCTATTCCTCTCCTTCTCTCTCTCACTCGTCGTATGTTCGTCCTAATTTTGACAATTTAACCTATTTATAAGAGTGTAAATGGGGTATAAATCCCAAAGTGTTAACCATTGCTTATTCTAATATAAAATATAAGACGAATATTGTGACATTTTAACAAACTAGAACTAGCAGCTCCAATAACTCACCGTTTAATTAGCGCCACGTAAATTAAACAAAAGATAAACCCAAATATCAACCACACGTGCTTTTTCTTGCACAAATAGCTATCATTGACAAATCTTGATGCGAATTTACCGAACTCCAGTAGCATTTACTAATAATCTTAAAAAATCGTGAAATAAATTGACCAATTTTCATCAACATTTATCCTAATAATGAATTTTTATTATTTTGTTCGATTTTTGTTAGATAAATAATGTTGAGAAAATTACTAAAGAGGATACAAATTCTGATGTAAGAGGTTATATGGCATGAATATATGAAACATCATATAAACATTTTTCTGTATAGATTAAATTAGAACGAAGGCATTTAGCCTTTTCAAAGTGGTAAATGCATAGAACATATGAAAAAGAAAAGTGTCTCCACCATTAATTTGAATTAAAGTACTAATTCGTAAATTTAACTAACTGTGAATACATTCTTACTTTACACTTATACTTTTTAGAGGAAGCAAATATATGCAGAATATTCCTTCCTTTCTACGAATATTGTTGTCCGATTCTTTTTGCCGCAGTAACTCTTTTTTACCCTGATAAATCATAATCAAACCCACCCAACACAACTCTGTTAGTGATCAATCTCTCTCTTCCTCCAAGAAAAAAGGAGGAAAACTAAAATTTGATTTACTCGTAACATTTTTTCCATGACTACATGCCACGGCATGATGATATATCCTAATTACGATTACATTATGACAAAGTTTGAAGATAGAAAGGACAAGGTAATTCAGAAAAATCAAAGAACACTTGGCAATCTCAGAACCACCAAACGAATTCTGGCTTTATTGTCCCATGTCACAGGACGTACAAAACGATACGTGGCTTACCTTCTCCTTACATAAACGACGCAAGTTGTCACACAACACCAAACCATCACCCATAAAAATTAAACCAACCATACTACACAGACACTCCTCCTAACCTTAAATAATTCTCCTCTCACCCTTATAAAAACACTCATTTTAACTCCCACTTTTGCATTTCCACCTATCTATCTATCCATCTATCTTTTCTAAC

> GmALDH11A2
TAAACATTAATATATATAATTTATAAACGTTGACGGACAACTTACACATCACCAATATTTTTTTAAAATTATTTAATTACTAACAAACTTAACTTATTAAAAAACAAATTAAAAAAATATAGAATTAATAAATCTGAAATTGACCAAGAAAAATTTTAAAAATAAAAAATAAAATATTTCAATTAAAAATAAATAAACTAAAATTACGTATTTTAAAAAAATAAAAACAAAAATTACATTTTAATCTTTTTTTTTTATCATAGTCAATCCTATACTGATCTTCCGTGCAGACATCCTCAACTTCCTTTATCATCTGTTTGAGTCTTTGAGAAATTAATAATGATATTTTTTTTCATTATATATGCAGCATTGATTGTTTCCGCTTTTTTTTTTATATACTAACTTTACACCTTTATCTATTCCTCTCTTTCTTACTCGTCGTATGTTCGTCCTAATTTTGACTATTTGACCATTGCTTATATAAAACGAATATTGTGACATTTTAACTAGAAGCTCCAATAACTCGCCGTTTAATTAGCACCACGTAAACTAAACAAAAGATAAACTCAAATATCAACCACCCGTGCTTTTTCTTGCACAAATAGCTAGCATTCACAAATCTTGATGTGAATTTACCAAACTCCAGTAGCATTTACCAATCTTCACAAACGTTGATCAATAATTCAATATTCCAGTAGCTTTTTCATTAATACTTATCCTAATAATGATTTTTTATTTTATTTTATACGATTTTTGCTAGAGAAATAATGTACAGAGAATTACTACTAAAGAGGATCCAAATTCTGATGTAAGATGGTATGTGCCATGAATATATGAAACATTATATAAACATTTTTCTGTATAGATTAAATTAGATTGAAGGAATTTAAAGGGGTAAATGTATAGAACATATGAAAAAGAAAAGTGTCTCCACCATTAATTGAATTAAAGTATTTCGTAAATTGATCGTGAATACATTCTTACTTTACATTTTTACCTCTTTTTTATAATCAGAATAATATTCGATTGCTTTCTTCGAATATTATTATTGTCCGATTCTTTCTGCGGCAGTAACACTTATCTTACTCTCATAATCATACCCACCCATCACACCTCTGTTTCTTATCTGCGTCTCTCTCTTCCTCCAAGAAAAAAGGAGGATAACTAAAATTTGATTTTAACTTTTTTTCCATGACTACATGATGATATCCTAATTACGATTACATCATATGACAAGTTTGAAGATAGAAAGGACAAGGTAATTAAGAAAAATCAAAGAACACTTGGCAATCTCAGAAAACCGATGCGTGGCTTACCTTCTCCTTACATAAACGACGCATGTTGTCACACAACACCAAACCATCACCCAGAAAAATTAAACCAACCATACTACACCAGACACTCCTCCTAACCTTCAATAATTCTCTTCTCACCCTTATAAAAAGAAACTCATTTTAACTCAGACTTTGCATTTCACCTTATCTATCTATCTTTTCTAAC

>GmALDH18B5
TCTTAGGTTTATAATACGTGATTTATAATTTTATAATATGCGGTTTTTGTTGTTCTATAATAAATATTCAATTAATTTATAACGTTCTTTTGATATATAATTAAGATGTATAATTTACTCGTATTTGTTAAAAAAAAGTGTATCATCTGTTCCTAAATTAAATTCTTTCAAAAGCCTCTAAAAAAAACTATTTCAAAAGTATCCAATTTACACTGCATGCGGATGAGGAAGAAGGGCACCATTAAGAGACATGTACTTTGCACATATGGATTTGTTACTTTACTTTTTGACAGGGACAAGAGTAATAAATCGGAGTCATATGATATGATTTTTAATTGGATTTTTAATGTTATATTTTATATTTTTGAAATTTTAAATTTCTTATTTATACAATTTCTCTCTCTCTAAAATTTTCATTTCCGATGCATAAACTTTTAAAAAATTAATCTTGTACTTTGAATTTTTAAAATTTAAATTGTTTTATACATATATTAATCATTTTTAATATACAGTCACATATTCCTGTAAAAAAAATATATACAGTCACATTAAATAAGATATTCTTTAAAGCTGTAAAATATATGCTTGTTGGTTCAATCTGTCATAATATTTTTGGAAAAGAAATAGTAAAATATTATACCCACATTTAATATTTCTAGTGGCTTATATGACAGTTGGCTCAAAAACATTCAACCGTGTGGACTTAGGTTTAAAATGAGATTGCCTGCAATTCTCTGCAACCTTTTTCGTTAGGAAAAGAAGAGCAACAATAAATATCAATTAATGTGAGGCATTGGTAACACTAATTAACAATTACACTACTTCCGTTTTGGTTTTACTTTAGGCCAGTCTTATTCTTCATTTTTTTAGAGTGTATAATCATTTATTTTGTTTTTATATAGAAAGTTGTATTTATAAAATCAAAACGTACTCCACTGTAATCTATTTTCTGGACACAATTTTTTCTTATTATCAACTTGTAACTAAATAAATCCTTTAAGAATTTTCACTCTTTATATAACTAGATAAGTTTTAATGAGAATGCATGAGTTGTTATAATTTTTTATATCTGAATTTGATTTTTATAAATAAAAAATATTTTGACAATACAGAAAAAGCTGATAAAAAACGTGAATAATATATATAATAATCCAATTATAAAAATTTAAAACTAAGCGAGGGAAAATGATAGAAAAAGAATAAAAAACTACTCCATAAGACGATTGTCATTATTAAGACAAATTCAAGTATGATTCGAGACCAAAAGAGTATACAGTAATTAGGATTTATCTGGAATAATTCCTCTAACCATAGGCTGTAATAATTAAGAATAAATAAAAGAAAATGTAAAAATATATGTGGTGATAGGATAGTGTAAAGTGTTAACCTTTTTATCAACACGTGACATTAAGATTAAGAAGTAAGAACTGTTCACGGCTACACTCTCTTTCTTCTCTGCCAACCACACTGAACAACAAACACAGTCTGTTTGGTCAGAAT

**The 1500 bp upstream sequences of 26 non-regulated *GmALDH* genes in response to drought stress.**

>GmALDH22A2
TGTTTTTGTTTTCTGAATGTTTGATGGGACAGTGCACGAAGGTGGAGGACCAATACCGCAATTTGTGAGCTTGATAATGAAAGAGCGACCATTGCGTTCTGCATTATTGGTGGCTCAGACTATTCCACACATTTGCTCACTTTGAAACTGAGGTTCGCTAATCGCCACCCACCGTTGGATGGAAACAAAGAAACCAATCCTTCCTTTTTAAGCTTAGATGTGTTTCTTGTTTCTATAATACAAGATTTTTTTTTAATTGATTCACATTTAACAAAATTAGTTAATATGATTAATAACATTAAATTTATTAATAATTTATATTATTTTTTTTAAAATTATTCACAAGTTTATTGAGATTTATAATTTTTCTCTTTTCATTTAAATATTTTTATAATTAATTAATTAATGTGTTAGTATTTTTATTCCATTCTTATAAGAGAAATAATATATTTATTTTTTATAACATTTATTGTAAAATAGTAAACGATATTTGGGTTGAAAATAATTAATTTTTTTAGCCATTTTTATGAAATGAATGTCGAGATTTATATTGTTTTTATTGATAAAAGTGAGTTTTAACTTATAATTAAAGTAAATTATACTCATTCCTCCTTAAATTAGTGTTTATTATAGTTATATTCTTTCTTTAAAATTATATTTTTTTTCTCTCCTGGTTGATGAACAATCACTCCTTTCAATGCAAGTTTGCTATCGGTCCAAATTCACATATGAAAACACAGATAACTCGTTCCTTACCTTTCTCGTCAACTTCGTCACCTTTTCACCAATCACAATAACTTCATTGTCCTTGCCTCCTCTATCGTCCTCTATGGTCTCTTCTAAATATTGTGATGACTTCACCAATAACAAGTGATCTCCCTACATGTCACATGCAGTTATCAAGCTCAACAACCTGCTTCACCTCTAGGACAACGCATTGCAACTCAACCGTTGTTTCGTCAAATATAATAATATCACATTCTTTGAGGTCGAAGATGTGACAGCGACATCATTAGATCTTCACTTCTTTCTCCTTTTTCTGATTATTATTTATAATATTAGTGAATTAGGAGATCAAATCTTATGTATGATGAAAAAAAAAAGTAGATTGAATGCAACAAGTATTATGCTCCGTGTAAGTATAATTTGCTCTTATAATTAATTATACTTTATGAGTAAATGTTTGGGGACAAATCTTGTATGTACGAAAATATTTTTCCTATTACTTGTATGTACAAATCTTGTATCTTGTATGAACTAAATCAACTTGTAAGTTAATTAAAAGAGTACTTAGTGACGGAGTTAGTACAAGTACAACACTAACAACAGTATCAAACGCACAGTTTCTTCTTTCATCGCCTGAAGGTTCCCAGATTCTTTCACTGTCTTCACTCTTCAACCATTGTCCTCAAAAGAACAATCGAAACTCCGCGGAAGCTTCTGGAAACCCTACCGAGGCAATTTTCTCGCTCACTCTGAATCTGCTGCGAACTTCTTCCA

>GmALDH2B5
TTCCTTTAACGTACGACCTGCTGAAACCTGCTCATTTTGTTTTACAATGTGTACACTAGTCTTTTCTCTCTGGGTAATGGTATTTTTAATCTTTTTTCACTCCCCATATAATGTTTTGATAGGTTATCTTTATAATATTTTGATTGTAAAATCCTCTAATATGATGAGTAAAAAAAATAAAAACAATAAAAAAGAAACATAGGGTTTGGGGTATTTATATATTATATTTTTATATTGTATTCTTAATTTAATGATTATATTATTATTTTATTAATTTAACATTGTAAATTAAGACATTGATAGTGATAGATGATAGGTTTTTTTTTGTTATCATATTTTACATTCTTTTATGTTATAATATTTTTTTTAACCATTGAATCACTTATAATATTTAATTTATTGTTTCAACATTATATTTTGTTTATTTTTTAAAATAATTTTATATAAGATGATTTATCTTAATTATTATACTAATTAACATAATAAAAGTATAATGTTTACATAACTGTATTTTCTTATATTATTTTAAACTTGATTAAAAATATTTTAAGAAATAGTATAAGATTAATAATAAAATATTTAACATAAAATAGTAAAATATAAATATGTATTTATATTATTTTACATATTTTAACTATTTCAACAATTAGTTTTACCGAATACTTCTAATTTAATAATCTAATTTTGTTGAACATAACTTTCAGATAACATTTTAAAAAAGGCTAGAAACTACTAAAAAAATTTGTTTATGGAATAATTAAATTAACTTTTCAACAAAAAAAAAAGCAAAAAACTATCTAAAATATTTTAGCTAACATAATCATTATCTTTTATATATAATATAGATATAGATATAAGTTTTAATAATTTAGTTCTCTATTTTCAAAGGTATAAAATATGTTTATTTCCCTGTTCATTCCATCATAAAAATAATTAAACAATAACTGTATTTTATTATATTTTATCATGTTCTATTTTTAATCATTTTAAACATTCTAAAATCCTTGCATTGATAATGAAATTTGAACTTATGTCATTATAAAATAGTATAAGTCTAATCTTTATCAACTAGATTTTGTGATTAGCGTTTTTCATGTCAATTTGTAGTGATTTTTTTTAACCCAAACAACATTTATTTCAGTGAAAAATATTTTTTTAAACCATTAAAAACTCATTTAATAATTAAGATGGTATAACTAAAGGATACTTGCAATCCAGTACCTAGTATTAAAAAACATTAAAGACACTTTTGTTAATTCAGGTAATACAACTATAAGATATGCAATTTTGGATGAGGACAACCTACATGTGCAAGAGCCAAGACACATGCATAGATTAGCGGCATATAAACAATTCTTAAAAGTAAATAAATCAGCCTGATCGCGTGGTGTGACAAACAGTAACACATTGGGTTGATCATGCGTTTTCTTATATAATGGGGAACCAATAGCTATTTAATAGAACTTAATTAGTGAAGAGAGAGAGAAGACGAACACTATC

>GmALDH2B8
TTGTGATGACTAGAAGCAAGAGGATTCAAGAGGACTTCGAGGAGAAGGAAGGTCTTTTCCAAAAGCTACAGATGACATCCTTACTGAGAAGAACCAAACACTTAAGAAAGTTGTGGTTCCTACTTATAGGAATCCAGAGCCAAAGAAGGAAGGGGATGAAAAGAAGGAGATACTGACATCTCCGTAGGTAAGTCTACCCTTTCCTCATAGAGTTATTAATGACTAGCAAGATGTGCAATTTAGAAAATTTCTTGAGGTTATGAGTAAAGTGCATATCAACCTCCCTTTTGTTGATAGTCTTAAATTTGAGTTTTGTCAAGTTTGGAGGGAGAACTTTTTTTTTAGGAGGGGGGGAGGGGGAGTTTAATGTCACTTTTGGTCCATTATGTTTCATAGTTATTTTGCTTTGGTACATTAAGTTATAAAAATTATATTATGATCCTTTATGTTTTCGAAATGTATCATTTTGGTCCTTTTTTCAATTTTCCATTAGAAACGTCAGTATTCTGTTAACTTTTAAATTTTAAGATGATTTAATATCACTTTTGTTTCATTATGTTTCACAGTTGTTTTACTTTGGTACATTATGTTTTAAAAGTTTCATTTTGATCATTTATATTTTCGAAATGTATCATTTTTGTCCTTTTTTTATTGTTGCCTCACCTAACGACAACAATTGTCTCATCGTTGTTGCTGATAGCAGCATGTTGACGAATGCGAAGAAGAAGACTGACAACACTCAATTGTGGATGAGGCAACACTATGTAAAGTTAATTATTCTGTTTCATAGATGTTTCAACACTAAAGTTTGGTCCATTGTGTTTTGCATATGTTTCACTTTAATGCATTAAGTTTTAAAAGTTTTTTTTTCTGTTCCTTTGTGTTTTTTGAAAAATATCATTTTGGTCCTTTTTAAAATTTAAAAGTTAACCAACCCTAACGTTTTTAATGAAAAATTTTAAAAAAAGACCAAAATGATATATTTCGAAAATATAAAGGATCAAAATAAAAATTTTAAAACTTTACGTATCAAAACAAAATATCTACCAAACAAATGAACCAAAAATAACATTAAGTTTTTTTCTTTTTATAATAAAAAATTTAAGTAGTTGGATTCCAAATTCCAAGGACTATCATGGACAAAATAGACCGACTTTTCGAGCACTTATAATTTATAAATGATATTTACAAATTAACATCTTCACATTATATTAATCTTGTCATTTATATTAATAAGATTGTTTATAATAGTTAAAGTTTAATTTTGGACTTGATCACATCTACGGGATTAAATATAATTAGAGGATTAAATGTGTTAATAATTTTAAAGGAAATCAAAATTAAAATTAGAAATAAATAAAAAGACTAAAAATATAATTTTACCTAAAAAATAAATAAATCATTCTTATCGCGTGGGCTGACAAACAGTAACACATGTTTGATCATGTGTTTTTATAAAATGGAACTGAGTTAGGGAAGAGAGAGAAGAGGAACACTGAC

>GmALDH2C4

GATCCTTTGCTCATTGTATTTTGAATTATTAAATTAATTAAATTTTGAGATGATTTAAAGAGGGTTTTTTTTTTAATAATTAATGTTTTTAATTGAATTCTTATTAAATTAAATAGTATATTAATCAAATTAACGATTAAAAACTTCAATATTATATATATATTGTTACATAAGCAATTTAACGGTCAATGTTTTTCTTATTAATGATCAATGATCTAAAATTGATGTTTGAGCCAAAATCTGTAATTTTGAAAAAATAAAGGGACCGAATATTTTTTTATAAGAACATCAAAATTATAAATTAAATTAAATAAGAAAATCAAAATTATAATTGACAAAATATACTATATATTAATAATATAAAATTATTTTATACTGTCATTTAATCACATTAAAAAACTAAAATTAGAGAATAATTAGTTATGACTTAAAAAAATATCCAATTCAAATAATAAAAACTCAAGAAATCAAAATTCACAATTACAATAATAGTTAGGTATCAAGTGTAATTATATATTTTTTTTAAGACTTAATTATAAATTTGGTTTCCTTATTTATCCCAGCTGATAAATTTGATCTTTTTTGTAATTTAAGTTGAGTCCTTTTTTTTATAATATGATAATTTTAGTCTTTGATCCCGAATTTGGAAGTTGACCATTAATTGTTTATATTAATTGTCACGTAATATTTTTATTAGACATTAACCATTAATTATTTACGTTGATTATCACGTATTGTGTTTTGATTGGATATTAATCTTCACATGTGTTTTGATTTTTTATATCTTATTAACGTTCAATAAAGCACGACACGTGACGGTCAACATAAATAATTAATTATTAACATTTAATTTCAAAAATGAGGACTAAAATAACAGGATTAAATAAAAACTCAACTGATGAATTAAATTTAAAAAAAAAACTAAAATCGGGAGCTAAGATAAATAAGAAAATTAAATTTACAATTAAGTCTTAAAAAAAAGATGTAATGAGAAAAAAGTATTCAAAGTTTGTTACTTTGAAGACTTTGAAAAACAATGCTCATCATAAATTAATTTGATGGTTGGGAAACATGTGTCACACGGTACTAAAAAGAAATATATTTATACATTTTCATTAAAATAATGTAAACATAAGTAGAGAGATAAAAAGTTGAAAAACCGTGTGTTCCTTAAATGATTTAGAGTTTATATTTTAATTATGAAATAAATTATAATTAAAAAAAAAATATTCACTTTAAATATATTCAGAATCCTCCATAAAGATTTCGTCATGATAAAAAAGAATATAATTGACATAATAAACAAACAATTCTATTATAATTCTGGCAGGCTCGTGAGTTGTATATGACATGTTTCTCTGTGCACCCATTGTTCTTGTCTCAAAGTAAAAGTGTGTGTATATAAAAGAGGTCCCTCTTCTCAGTTCTTTGAAAACCTCAACAACTCAACACGTTGCCTCTACTCTCTGCAGCTTTTAATTTGTTTGCAGGCACACAACC

>GmALDH2C6

ATCTCAGCAATTTCTACTCAAGCGAGTAGAATCCAAAAATTGGGATTCATGTACTTTTTAAATGGAAAAGACTTTATAAATTTAACAAATGTTAGATTATAAGTTTTATAATTTTAAATTTTTCACAAGTATTTATATTTCAACAAGAGTTTGTAGTCGTGACTAATAACAAATTATTTATAAATATATAAAGGTATAGAAGTTTCATTGAGCTTTTTTAGCTACAACACCTATTTAAAACTAAGAGAGAGAATAATAGAAGATTATAAATGATTTTGTATTATAAATGTTAATAAAAATTAATCCATAAACATATAAATTATAGTTTTTCATGATATTGTTTTAACTTTGCACCATATTCTTTATTTTACTAATTTACTAAATAATTTAACATGTTTCAATTTTAGAAGGACAAAAAATAAATATAAAAATAAAAATTCATAGGGATGAAAACCAATTTTTTTAATTTTATAAAAACAAAAAAATATGTTTTAGTCTTTTAAACAAATTAACTTTATATTATTTTCTCATTCCTTTCTTTGTTAACTCAAATATAATTTTTACTAAGAAAGATTCATCGAACCGGTAAGGATCAGAGTGCATCCCACAAGAATCCGAGTTTAAGTCTCATTGATGTAAATATAGGGACCTTACTTATGGATAATTTATTAATATCTTTTAAGTGCTTAATAGATCTTAAAAATATTATCTCAAAATTCAATTCACCTAAATTTTTCTAAAAAAAATCAAATATAACTTTAAAGTGAGTTGGCATAATCACCATTCCTCCATTGCATAATACCATCGTATCCTAAGAAGACATCCATTGCATAATACCATCATTCCGGATTGTTGTAAAATTGAAACATGTTTAACTTTAAGAGTGCTAAATAATTTAACATGTTTCAATTTATAGTTTATCATTTTTGCCGTAACGAAGAACTAGTTATATTGCAAGCAGCCTTAAATTATCATTCGCGACATCCCATGGTATGTTGCGCTTTTGTTTTTTTATTCGGTGGAAAAATTTCAAAAGAAGAAACTATCCTGCACTGTCTCTAAACAGGAGTAGTGTGTGTTCATCGAAGGAGGGGGAGGCCGAATCCCAAATACAATATTAATTCTTTGCACCTTTCCAGAAGCACTATATTAATTCTTTAAATAAATAAAAGGCAAAATCACTATTTTAATTGTTTTTAAAAAATAAAAATTAAATGGGGTTAGGTGGGGAATGATTTAAATATTCTTAATGGAACTCTTATTAAACAATAATGATAGTATTATATTCTATTACCAGCAGGTAGCAGGTGTATCATTGGCGTTCCTTGCCTTTGCGAGAGCGCTGATTTCTCCACCTAAAGTCCTAAAGTTGCGCCAATTCATGTTCTTATCTTTGCATTGCATATATATATATGTTCTTCGTAATTAATCATATTGCAACATCCCGCGTACCGCCAGCGTCTCTACGACAATCTCCTTTCTCTCTAACTCATAACTCAG

>GmALDH2C8

ATGTTGGGCTAGTTGGTTATTTAAAATAAGTATTTATTTTTTTATTTTTTATGTATTTACTTTTTTTACTTAAAATAAATAGTTTTTTTTACCTTTTTAAATAAATTTTATCTTGTTCTTAAAAATACTTGTATAAAAAACCTTTTTTTTAAATTACTTTTTTAAGTTGAAATAGATTGACATATTTTTTTTTTTATGAAATCTGATAATGATTTAATCCAAGTCTAGCTTGGTTAGTTGAACAACGTATTTAGTTATAAATTCTTCAATATCTATTTTAGATTCCCATGGATAAAAAAATGACATAATTTTAGAATTGAAAATAATAATTGTAATTTATGACTTAAAGACTAATTTAAAAAATGTTTTCAGTATGAAATTTATTTAAAGGTGTAGTGTCTATAATGTCCCTAATTTGAGCGGAAAATTATTATAATTTCTGATATCATATTTTCTTACTTTTATATGTTAAGATACTCACTACTACTAACTAAACAACATTAATAATATAATACAAAAAAGTATAAAAAAATAATATTAATATAACACAAAAATTAATATTGCATTGATATATTTATTAACTTATTAATGAATTTAGTATCATTAATATTTTTTTAGGGGAGTATCATTAATATCCTAAAACATACAAATTTATATTTATTAATATTCATTTTAATATTTTATTTATATATTTACTAATTAATAAATAAAAATCAGTATGTTTTAGGATATTAATGAGATTAAATTAATTAATAAGTTAATAAATTTTTGTCAGAGAAAAAGTTTAGGTGACTAAAAGTTTAATTAATATGATTGGAGACAAATGTAATTGAGAAGAAAAAATCTTATTAGTTAAATTTTTCAACACATATTAGCTTTTGATAAAATTTATGAATATTTTATATTAATGATATCATAATAAAAAAAATATTTCAAACAATACATTAACATTATTAACATTATGTTTGATTAGAGAGAAAATAAAATATAATAGGAATAAAGATGTATTTTTTTAAAGAGAAAATAAAAAGAAAAAAGAGTCAACTCAAATGATTGTCATAACTTTGTAAGATTTGATCTATTGTGTTTATATAATTAAAAATGATTTATTTAGTGAGATGATATTTGTTTGATTTTTATTGTATATATTATGAATTATAAGTGATATTAGTTTTTAACCCAATAAATTAAGAGATAAGGCGTCATTAGGAAAAAAAGGTTATAAAATTCTATAATTGACATAATAAATTAACAATTAATTAATTGATTCTATAAAAGCGTTATACTATTAGTTTATTATAAAAATAATATAAGTCTGGCTGGCTCGTGAGTCGTATGTGAGATGTACCTTTTCTCTGTCGACTGGTTGTTCTTGTCTCGGAGTTAAGTGTGCGTAACTCTGTATATATAAAAGAGGTCCCATTCTCACTTTTTCGAAAAACACGTTTGCCTTTGATCTTCTTGTCTCTACTGCTTTTATTTGCAGACACCCAATTAACG

>GmALDH2C9

ATATACCTAGAAGCTTTACATTTTAGTTTTTATACGACCAATTTTCTTATGCTTTAATCCCTTGTTATTAGTTTTTGGCAGTATTAAAGTATCATAAAGTTGAAACATAAGGACTAAAATATAAAGAAATTGCGCGTAGAAGGATAAAAACATGGAAAAATTAGAAACATGAACTAATTAAAACGAAAAGTAGACACAAGTATAGAATAAAAAATAGTTAACTAAACCTTTTTTTATATTTAATTTTTTACTAATATCCTCACAAGAATGGTTGGGAAATGAATCTTTGAATTAAAATATAATATTTAAATAAAACTTTCATTCTAAACTAACAATTCTAAAATTAAAATTTTGATATTGTAAGTAAAATTTGCTTGCAAATACGTCATACTCGAAGGTCGTCAAATAAGTCTTATAAAACTTAATTTGTATCTACTTGATCCTATATTGTTGCAAGGCAGCTAATTAAACCATTTCATGTGTGACTTTTATATTAAAAGAAAAAGGTTTGAAAAATTATACACACTAATACCGGAGCTATTTTTCGTTTTTGTGAAGTAAGTTTGATCATTTTTTTTACTGCTTGATAATTTGTTTTAACAATACCGCGCTAGCTAATTGAAAATTGAGAACTGTGTACTATACTTGATCTAATAATAACGACTGCGCATCCATAATTAATTTGTTTCTAAGTCTCGGTAAATTGTTTGATTCTAGGGAAGGAAAGGAAAAAAAATGTGACGTCAGAAGTAAACATTTTTTTTTTTTTGATAATGATCAGAAGTAAACATATTGACTATAGTATTTGCTAAATATAATATATAAATACTCATAGCCAACATATATAGTCAGTTTTACTTATAACATATTGACTATAGTATTTGTTAAATATAATATATAATTATGATAAAATTAAATGTTGTATTTATTAAACACAATAAATTTATTAAATATAATATATATATCAAAGTAAACATACAATTAAAGGTTATTTACTTTAATTATGAAATTTTTATTAAATGAAATAGTAAATTAAAGAAATTGAATAGAAACGGTAATAGTTAATAAAGATATAAAACTGAAATTGAAATTAAATTCTACATTTAATACTTTTATTTTCCTTTTTTTTAAGGGACTTTTATTTTCCTCCTTTCTTGGTTTTGCATCATTCTTTGTATTCCCAGAAATTCTGACCATAATGTGGCATTTAACGTGGAAAATGCGAAAGTAAGAAAAAGGGTGAATTCCAAGTATTAAGGGGAGGCCAATTGCCAACAAACTAAACCGTTTTGAGATTGATATCTATCCACCTAGCTATGCCATCTACCTACCAAATGATCAACCAGCTAGCTACCAATATATACCATCATATTCATATAATGCATATGCAGTGTTGTGTCGTGCTACATATAAATAACTCTTGTGCTCCCTCTACAGTCTACACACACTCCCAGAAACTTGTTTGGTACTTGACACTCAGATTCACTCAAGTGGATAAGG

>GmALDH3F1

CAGCTTCCTAATAGAATCAATACTGCTGAAAGGAAAGAAATCCCGTGTCGTGCGCTAGTTGCTATCTGGTCCAGACATCCTTCTGATTCTAGCCGTAATTTCAATTGATTTAATGTTAGGTTATGAAGCATATGTTTTGGATTAAAATTTACAAACTTAAATTTGAAAAAATATCTTAATTTTTACGGTAATTTTTTTCTCTCAAACATATAAACTTATTTTTAGAACCTTCACACCAAAAATTCAAACAAATCCAATCCAATGAACTAATACTAACAACATATTCTTAACATTTTTGTAACAATTAAATTTGTTATTATTAAATAAATTATATGTAAATCTTATTAAATAAAGCGTAATTCCAACTTATTTAACAAAATCCACATAACATCTATGCAATATTAAAAAATGGGTTAAATTAAAAGTTCATCTATAATTAAAAACATTTTTCAGTACTCTATATGAGAGTCACTAATATTCATCGAAAGTATGTCTATCTAACGAAACATGATAAAGATATTGTTGAAAAACAAAAAGATAATATTTTTCAGATGAGATGGTAGTTGCTATTTACTCCCATAATAAATGGTTGGTTTAATTGGATAACTAAAAAAATATGAATAGATCTTTTTTTCTTTTTATCTCCGTGAATGAATCTTCCCAATTAAAAAAATCCTCCATATCAATAATACACATGTCAGTTTTGAATGGGGTAATTCACCTCTAAAACAGCTTATTTAATGTAGTTTATTCGCTTAGATATTTTGCTTCCCATGAAAAAAAAGTTTATGAATTAGTTCGTGTCATCATGTTGTCTAATCGTAACTTAAGTGCACCTTATTAATCTTATAAAGAGGCGAGGCCTTATAGTCAATTCAGATCTAAGCCAGGACACACAAAAGAATGATTTGACCTTTTAGATTTGTTAACATAGCCTAACAAAAATTAACATTATAATAAATTAATATTTATCTATAAAAAAACTAATTAACAATGAAAGCATAATCTTTCATCCCATTAAAGTTCCGCTTGTTTTAATTGAAATGATTGCAATTTAAAAAATTTGTTTAAGTAATTTATTTCAGAGATTAATTCTTACATAAATACGAACACATTAGACAACAAATAAATAAAGACAACACTCACACAATGAGGTGTAATTTTGTGATTAAGCGCGTCTAAAATTGTTACTCCTACGTTGAAGTGACTTGATAGGGTTGAGTTCTTACAATAATCACTTTTCTTCCGAAGCCAAAACTTATGTCTTCCATCCACCAATAGCTTTTCAGTCTCAACGTGAATTTATTACTACTAGACAACCAAGCAGTAGTTATGTTAGATAACCCCAACCAACTTTGCATTTAATGAGATCACCGCACTATAAATATCCATGTCCACATGTGTGGCAAGTAGAGTTGAGGAAAAATATTAAGAGAGAAAATAATCAATACTACAACTTTACAATTTTCGTGGTAGCTAGACCAAGGTAGTTCAATTGCC

>GmALDH3F2

AAAACTGTTTCAAAGCAGAATGCAAATGATATATGTTGTGTTGTGTGGTCTGATTTGAAAATTTTGCAGGTTAATTTTTGGGTTTATTGAAAAAACAGACAGTCTATTAAAGAAAATTCCTAATAACAATATCGATTTTTTTAAAATACCGATGTTAATATACACAAACAACATCAATTTTTGGAAAAACCGATGCTAACATTTTGGAAAAATCGATGTTGTTTGTGTATATTAACATCGGATTTCTAAAAAAATTGATGTTAACTTATATGCTAACATCAGTTTTTGTTAAAAACCGATGTTAACTTCCAAATACGTTAACATCGATTTTCTATAAAAATCGATGTTAACATTTGAAAGTTAACATCGGTTTTTTTTTTTTTAAAAAAAACTGATGTTAATGTATAAGTTAACATCAGTTTTACATTAAACCGATGTTAACATTGGCAGTTAACATCGGTTTTAAACTAAAACCGATGTTAACGGCCAACCCTGTTATGAACATTGATACCTTTTTTTGGTGTAATTCTCATTATATAACATCGGTTATTTAAATAACCGATGTTGTCAATTTTATGTTAACATCGATTTAAAAAAAATCGATGTTAATGTAATACTTTCAACATCTCTTAATAACCGATGTTGAAAGTCATAAATAATCGATATAAAAAACATATTTTCTAGTAGTGATTGAGAAATCTCTTGATGATATATATATTTCAGTTTGAAAATGTTTGGTGACAAAGTCATTGAATGAATAATGTTTGGTGACAAAGTCATTGAATGAATAAGTAATCGTCCTTAAAACGAACTATTTAATGTGCATTATCAGCCAATATATAATAAAATATTTTATATTTTTTCCTATATGAAAAAAGTATTTATGGATTGCTTCATATCATCATGTTGCCTAATTGTAACTTAACTGCAATCTTATAAATAGGTCAACTTTGGTCAAAGTCATTTAATATATATATAATATTTCCCTCAGTTAATACTTTATAGAAATTCTTCTTCACTTAGTGTCTAACCTTTTTCAATCAATCCTTACATCAACAGAAAAGAGCAGCACCACATCAAGAGACGAGGTCTAATAGTCAATTCAGATCTAAGCCAGGACACACCCAAAGGAATTATTTGACTTTTGTTGTTACCATAGACTCTCAACAATACAATTAAAGTTCCGCTTGTTTTAAATTCGTATGATTACAAATTTAAAATTTGTTAAACACGAACTCATCAACACATTAGACAAACAATAAATGAAGACAACACACACAATGAGGTGTAGAAATTTTGTGATCATTAAGCGCGTCTAATTGTGCGTTACCACGTCGAAGTGACTTGATAGGTTTGAGTACATACAATAATCACTCTTCTTCCGAAGCCAAAGCTTGTGTTTTCCACCCACCAATAGCTTTTCAGTTTCAACGTGAATTTATTACTACTAGACAACCAAGCAGTAGTTATGTTAGATAACCCCAACCAACTTTGCATTTA

>GmALDH3H1

CCCAACTCGACACTATACAACTCATTATTAGTTAGAAACATGTGATATCTAAAATAGAAATATTGATCTCCTCACATAGTATAATTTTATTGGACACCATGCATTATCATTCATGTAGAAACTTCATATCTTAAGATTGTATTATTATCAATTGCACCATTTGATTGTTTGCTTGCATGATTATATTTATGTTTTAGTTGTCCTATATGAAATTTGTTTGTTTAGTTGTTTCTAATTTTGCATGTAATAAAAATAAATTGATGATCATCCTATTCCAAGCCCCTTCACAGCCATCATACCCGCTAAGTTCGTACGAACAAACTTTATTATCGAGATAACACATTACCAAACTTTATCATCGAGATAACTTTATTTTAATTGTCCAAATTGTAAAGCTTCACTATCATTGAAGGAGAAGTTGATAAGGCAATGAAGAAAATATAGAGACAATATTTAATCAAGAATAGGCTATGTCTAAAGGGAAGTTCAGTCTTAATGATCGATTAGACAAGGCCTATACACGTCACCGACTAGACTTCATAGTTTTTAAATTTTTTAAAAAAGGACATATCGATCGACTTTTAAAAAAATAATTAAAATATTGATTTCTTCAAAAGAAAGAAAGCAAATAGATTAAGAGTCCTGATTTTTTATACGCACATATCTCACGTAAATCTTACAATCTTATTTATTTTCTATGTTTATCTTCCTCTTAAATCATAGGAAAATGTTTAAAAAACACTCAAAATGTTATCTAAAACTTAAAGAAAAAGAAAAAAATATATAAAAAAATAAATATAATATAATTTATGACGTGATAGAAAAAACAAAAAATATATTAATAGAGTGTTTAAAATTAAGAGATTGTAAGAATCACTAATGTGTTTTCATTTTTATTCAATAGATTAATAAAGTGAAGAGGGTATGGTCAACTTTGCTTTGAAATATCTCAACATAAAATATGAAAACAACAAAATTTCCAAGTTGTACGGAAAATTGATTTGAGAAATATCAATTATAGCAGGGTTTTTTCCATCAATTATTAAAAAAGTTAAGAGAGAAACGAAAAAGTCGTGATTGAGGATAATCGTGTTACATTGGGAACAAAAAAAGTTGTCTGGCCCATTCAGGAGCACCAACTTCTTCAAAACGACAAATATGAACCCTACTGAGCCACATTAGCGTTGCTCTCGCACCGCCAAGCCACTGCACAGATAATGTTCCCATGGTATGATTCAGAGAGTCACGTCCTTGATTATTGGAGCAAGCACGCACGCTATCCTTATCTATCTCTTCTCTTCTTATTGCTCATTATTACGCGTCTCTCTCGTGTAGTATATATATACGTATGGTACTGTTCTTTCAATCATTAAACTCCTTTATATTTACACACACATATATACCTCTCCTCCTAATTTATATCCCAAGTTTCCACCTATCCAATTTCACCGTTGCTAGCTCCTGTTTCTTTCTTTTTTGTCGGAGAAAATAGTGATAGTA

>GmALDH3H3

AAAATTTAAATATATAATTTAAAATGCATTAAAATAGAAATTAATAAAACCATAAATATGTAAATTGAATACATCTCATTGAAATAAATATATATTAAATGACCTAAATAAAATTTAATAACTATCTAGAAGAACATGAAGTATATTTGAATATAATAGTAATTGCCAACATAATGAGTGAACGTAATTTAAAAATAATTTTCTGTATTCATATGGTTTAATACCTGATTAATGCATGTAGTCAATATTTAGAAATATATAAAAACTAAAATAAATCTTGAAATCTGTCTTAATTAAGAGATCAAATAATAATTGTATTTTAAAACATTTAATAATCTGTCATGTTCTTATGCTAGCTCACATATTTTTTTTATGATGTGAATATGTATTTTTTTTTTGTCTTAAACACCTTTGGTTCATCATAAAAAATATATCATCGTTAATCTGAAATTTGATCGAAAAATAAATAAAATCTGATTAAAAATTATTTATGTCAAGGATCAATATCAGATAGAGTTAAAATAATTTAATCTTAATTTTAACACATTAATCACTTATATTTTATATTTCAGGTTACATGTATTGTATTGAATATTGATATTCTTATTATGAATGAAATACAATAATTCATTGTCTGAAAAAATGTATTTTAATTTTTTTTACTTTCACGCAATTTTTGTCATTATTTAGTTAATCAATAAAAAAAAATCACAGCAATGAAATAAGAAAGCATACCATTGAATCCTCAATATCACCCTGTCATCTGTTCAAAGCATGATAACATAGAATATTAAATTGCTTTAGAATAAAGAAAAGCATTCCAGTTTAACAAACATATAAAAATACATTTATTATTCTCATATTAAGATAAACGCACTAGTTTAATTTTATACAAGTATTTTATTCCGTGAGGACTCCTTGTGTCACATGTTAAAAATCATCTATACCAGAATAATTTGTATTTAATTTTTATTATATATAAAAAAATTTAAACCAACAACAGTCATATATCACAAACAAATATATGATTGAATGAACTAGAAACATCGGTTGGTTGTATGAAACCCAAGACCAAAATATTAGTGTTTTGTCATTGTACAGCAATTTTATTTTATTAACTATTATCATTAATTAATAATTGTCCTTTTTTGCACAAAGCCGTTCCCTTTGGCTCAACTCCAAAAACCAAATTATTCCTCATTTATATATTTTAATTTAATTTTATAAATTTTAACTCAACTCTAAAAATTTACTCAAAAGTTAAAAATTATCTTTAATTTATATATTTTAATTTAATTTTACTTTTAACCAATCTAGAACTTACCTTTTCTCAGTAGTATTTGGCAAGCTATAAAATCGGAAGTATTCCTACTTTCGAGAGTAGTAACACGCCACGAATCACGATCCATCATCACACCACTCAACTCTGCACGGCATAATCCTCTTTCTTTTTTTCTCTGTTTTCTTTACTTTCCTCTTCGCGTCGTTTTGTCCGCGATA

>GmALDH3H4

ATCTTTGTAATTTAGAGTTTTTTTTTTATACTTTACATCCTTTCAAAAATACTTTTCTGATTTTAGTTATTATAAATTATGTTTATTTAATTTTTAATTCTTAAAATATTTTAAATAACACTTTGAATAATAAAACAAATATTATCTAAATTACTATAAAGATAAAAAAAATACACATAATTTATCAGAACTAAAAATAAAAAATTATTTTTCAATACAAAAAATAAAAATATTAAATTATAAAGTTTAAAAAGATATTTAAGAAAAAAAATAAAAGAAAAACCTACAAGCAACCGGGGTAGGACTGTTGGAGCACCAAACTATTCCTTTCCAAACTCCCCAAAATAGGAAGAATGAACATTGAACAAGCAACTTTGGGCCATAATTACAAGCAACTTTCTTGTCCCGCCACGCGCTTCCATCATCGTGTGACCAACACCCTCCTCTCATAGCATATCAGCTTGATATTTTTATGAGTAGTATTTTATTCTATTTATTATATCTGATAAATATTTTTTATAATTAAAATTCATATTAAGCACATTGAATACATCTCATTAAAATAAATATATATTAAATGACCTAAATAAAATTTAATAACTGTCTAGAAGATCATTAAGTATATTTGAATATAATAATTGTTGACATAACGACTGAACGTAATTTAAAAATATTGATCTGTATTCATATGGTTTCAGACCTGAGCATGTAGTCAATATTTAGAAATATATAAAAACTAAAATAAAATATACTATTATTATTCATAATTATTATTGCCTTATACTCTTGAAATCTATCTTAATTAAGAGATTAAATAATAATAATTGTATTTTAAAACATTTAATGATTTGTCACGTCCTTATCCTAGCTCACATATCTTTGATAGGATAACATGTATTGATGTTACTATGAATCAAAAGCAATAAGTCTTTTTTTTTTTTTAAGAAAAAGTATTTTAAAACGTCTTTTTTAGTTTCACGCATTAAACCAAAAATATATTGTTGTCATTATTTAGTTAATCAATAAAATAATCACAGCAATAAAACAACAAAGCAATAAAACAACAAAGCATATCGTTGAATCCTTCATTGCTTTAGAATAAAGAAAAGCAATCCAGTTTAACAAACATATAAATACTTTTATTATCCTTTAATATTAAGACAAACATACGAATTAAACTTAATTTTATAGTGTTTTGTCAATGCACAAAAAATTTATTTTATTAATCATGATAATTAATTAATAATTGTCCTTTTGGCTTCCGAAATGGAAATCCAAACTAATCATAAAAGAAAAATCTCAAATCACGTTAGCTTGCTAACTTGTTCTCACTTCTCAGTTTGTTTGTTTGTATTTGGCAAACTATAAAATAGGAAATATCTCTAATCTCAAGAGGAATAACAACACGCCACAATCCATCGCATCACTCAACTCTGCACCGCAAAATCCTCTTTTATTTATTTATTTTTTTCTCTGCTTCGCGTCGTTTTGTCCGCGATA

>GmALDH5F1

TTTTTTGCATGATACTTAAGTAAATTTGAATTGTTAGATGTGCTGATTTTGAAATCAATGGTTAACATTTTTCTTTTTCTCCTTTTTTTTGTTTATCTTTCCCAATTTGTCGCTGACTACTCCGTTTCTCCGATCGCCTAATCTCCCTATCCTTTGTCATCCATTCTGTCTCCATTACAAACACCACTTTGCCACTAATAGAACCATTGCTTCTCCATGACCATCAATGGCTTTCAAGAAAATAACAAACAACAAGGATCCAACACAAATCAACAAGAAATCCATTCCCAAAGGAAAATGATGAACCATTGTAAAACCTTCCCTTGCATGTCAACACGAACCATCGTGCGGCATTGCTTCCGACCACCCCCTTCATCTCGTCTTGATGCCTCATTCCTACCATATCCAAGGGCGAATGAAACCATATCACCTTCCCGAACCACAAAAACTCAATGTGAAACCAGTAAATCCCATTGTGAATCGTGAAGGAGACCACAAATCATTACAAATCACATAACAAAAAAGGTGAAACTTCTACTAATTGCCCCGTGCCTCTACAGCCACCAAAGATCAATCGTTGGAACGAGGGAGTCTACTTAGATTTGTTTCAATCGAAGCCCAATATTGGAAAAAGAGATCTGTGATAGGGCTTATTTAGATCTACAAGATGATGGCCAACAGAGATGTGTGGGAGGGCGGTGTGTAGAAAGATGCAAGGTGGTTCGCCGCTAGGAAAAATGGAGATCAAAGTGGCTAGGTCTTTGAAGGGGATAGATAGAATGGGTTCTCTAAGGGACAAAATTGGGAAAGTGAAGAAAAAATAAAATTTTAAACCATAGATTTTACATTAAAAAATCTAACGATTATAAATAATTTGCGAACGATGCGTGAGGAGTTTAACTTACACACCGCAACCTTTGTTTGTCATTACCTCAAGAATCTCACATTTTTTTATACCTACCTTTATGAGTCTAGGACTTATGGGTTTTGTGGATGATTTTCCTTCTAGGTAATTCGGTCTTAATCACGGTTCCAATGTTTAATTGTTTCCCTAATAGTGACTAGGATGTCTTCATTATTTTGCTCTATTTAGTGAAAAAGGGGGGGCCTTAACCATTCAGCTAATGGGTAGTATATTCAACTAGCTGATTTGTGAGTTCTCAAAATAAACATGATATGCCACGCTCTTATACTTTTCGTTAAGTGTCTCGTCTTTTGAATAGTCAGAAACCCATTTGATATACATGCCTTTGGTACACTTACTTAATATTTCTCTTTCCTAATAATAAAATATTAAGAATAATATAAAAGATAAAATTAAAAAATCATTATTAATAAATTAATTAATATCTTATAAATAGAATCAAATAGTTTCATATGATTAAGGTACATTACAACACTCAATTAAAACATTACTAATTACTAAATATTATTTTAGCCGATTCACTTCATCAAGCACAAGTAAACAAAACCACTGGAATTTTTCGACCACCATTGCAA

>GmALDH5F2

CCAGAAATGTGGTAATTGATTCCAAGAAATGGTAATCGATTATCTCGTTTTAAACAGAGAACTTCTCATGCTTCTGAGTTTTCTGGAATAATCAATTATCTATATGGTAATTAATTATTTCAACCACATAGAGAATTCCTCAAGTTTTCAGACACATTTAATCAATTAGAATAAATTGTAATTGATTATCTCAAGCTACATAGACTTCCTTCTTCTAAAACTGACATATATATTTGATTACAAAACCAGTAATCGATTAATTCAATGCTTCTAACCAAATTTCAAGTAAAAGATGATTTTGTTACTTGTTCTAACACTTTGTAATGGATTAAAAAAACCTTGTAATTGATTACACAATGTTGAACTTAATGTTTCTAAGAAACTTAGAGATCAATCCACTTGTCTAACATATTTGAATCATGCTAAGCATGGATATAAGAAAACTACAACTATATCAAGCATCATGCATAGTCTAAAAACATAAAATACAAATGTCACATTTATTAAAATTTGTTTGGCATTGTAAAATCATAAAACCAAAACCTTAGGGATAATCTTCAACACTTCAAACTTGAATTAACAAACTCTCCCTTTTTAGTTTTGATCATGTTAAAAAAATGATGAGTATTGATGTTTTTCTTTGTCATTGCATCTGTGAAAGTCTATATATAACTTAGCACAAATAGATCTCCCGTAATAAGTGTGTTTGTGATGGGAAAATGTCTTGACTCTTTTCATGAATGCGCTCACGGTCCTTGATGAATATTAGTAACCGTTTCCAATGAGACAACTTCATACCAATACTATGGTTAGCCAAAAAGGGATGGGTGGGGGGGGGGGATCAACTATAGGGTAGGTATTTGATTAGCCAAATGCCAAGTCCTCAAAATAAGTATGATATGCTTAGCTCTCGTACCTCCTAATTCAGTATAGATGTCCCTTAGATTCCTTGAAGTAAGATCTCGTGTTCGAGTTTTATAGATGAAAAAACGTGATTACTAAATTTTTTTTACTAAAAATGATGAATCATATTTTTTGACGGAAATTAATTATCAATAAAATTAATGGATATTTTACACTAATATTGACCAAAAAAATATAAGGGGAGATTTTTAACTCTAACTCAATTAAGCAGCCTAAACTCATCTAAAATCTTGAGACATTTTTGTTGCATAAAAAAATCACCCAATACTCTTTTTATTCATAAGAGGATAATTTAAAATGTAAAACTTTATTTTGGATTATCTTTTTAAATTAGGAAATGGTGTTGGATGATTTTATTTGGGTAAGGAATCAGCCCAAATGGTCGTAGTGATCCGAATTAACCCCACATTTGTAGCGTTTTTCTCATGAGATTATGCCAATAATCATGCATCCAAATATTATTTATTAATTAAATTACATTATGACATATTGGCACATACACTATAGCTGATTCACTTCATCCAGCGCAAGTAAACAAAAACCACTGCAATTTTTCTATTAGGACGACCACCGCAA

>GmALDH6B1

TCATTAATAAAATATCCAGCGTATTGATCCTAAAAAAAAAAAAAACACTCTGTTGATAGCACAAACTACCATTACCTAGTGTTTGGTTTAATTCCACTCGGATATATTAGAGTAGAAAGTTTTAAAAATCGGTATAAATCTCATATTAATCTTTTACTTTGATTCAAATATTTAATCTCTAGTCATTTTTACACATCTTTGATTGCTTTAAATCAGACTCTCTCTAGAAGACACTCAACCAAATATCGCAACCTTTGGTTCATCCTCTTACAAAGCCTCCACCTTTGATTGACCTTATCCTCTTATTTCATGGCCATCACTTCTATAACCCCAATGCCACTTAAATTAATCTTCTTTAACTATTTAAAATTTTAATTATTTTAAAAATTATTATTATAATCGCAGAATTCAAGTTACCTAACATTACTTTTGAACTCAAATAAATATTAGATTTGTAAAATTGTGAGATATAAAACGTAAGTGTAATTACTTTTTAAATTCATATTATAAATAGGACAGAACACTTAAAACAATACAAAAACTGTCAAATACAAATGACTCTACAACTCAACTGGGAGGTAAAATTAGAATAAAAACTTTATCACTTCGTTAAAAACCTAAAACATATTATTGTGTGAGTTAAAAAGTCACTTATTTTTAAAAAAGAAAAGAAAATACATATAACTTAATAAGTTGACACTTCTCCTGTCTTGAATGTAAATAAATAAAAATCATGTATGTTAATCATATTCAATATAAGTAAATATCAACCAATTTTACATTTTTTTTATCCTATTCAATGAAACAATTTTCAAAATATCTTTTACCAGACTGTATTCCAAATTACTTTTATTTAATTATTTGCGATGTCAACTTTTAATGAATGTCAACTTAAGCAAATAAATTATTTTTAATAAAATTAATAAAATAAAATTATATTAATTTATTTATTTTATTTATATTTACTTCTAAATAGCAATTTGTTATTTTCAGTTTTCTAAACCTATTACATTATTTATATTCACATTATGTTTAGAAAGCTGAAAGTATTTTCAGCAAAATGTTAAAGAGTTTAATTTATTTTCATTCACTCTAAATATAAAGATTTTCACTCTACAAATTACCTTCATATTCAATTTTTAAATTCTCAGCTATGCATTTAAATTAAAATTCAATGACAGTTTTATAGAAAGTTATTGAATTAAATGAGTATTTAATTTTAATAGTATGAGAGTGAGTCTGATTTGGCATGTCTATCGATTGATTTGTCCTTTCTTGTTCTTCCTAATATTATATTGATACGCAACTGAAACGCAATATGCTATACGATCACTGACAGAGTCAGTGTTTCTGGTTGTTTGCACTTTAGATTCAAACCTGATAGGCGTCGTTGTAAAAGGAAAAAAAAAGTTCAGTGTTTTTGTGGTCTCGTATCAGAACTCAGAAGTTATCATCCTTAATATCTTCAAACAAGGTTTTTCACAGCACATCGATACGAGG

>GmALDH6B3

TGGAGTTCAGAGAAAAGTTGAAGTGTTTGGTGATAAAGACCCTTTGAGAGGAAGCTTTTGATCTGGTTGGAAGGGGAAGAACATGTGGGGGCCACTGTGGTCAGAAGGCGTCGGGTGAAGAAGGCCATGCAGGGCAGAGGATTCAGAAACAGTGTTCATTTCTTGGGCAAATCCAGGTGCTTTTTGTTTTCTGACGGAACTGAAGAATTTTAGGACTATGTTTAATGGCCATTTCAAAACAAGGGAGTGTCCCCCTCTTCCAATTTTCTTCCCCTGTATCAATTAATGCATCAATTCAAGAGAAATATATTAGTATACTTTTATAATAACTTTTTTTTTTTACTGTAACTTCAAATTTAAAAAAAATAAAAATATATATATATATATATATATATATGTTAATTTTAAATGTATTCCTTTAATATTCATGTATGAATGAAATATCAGATAAATTTAGATTAATATAAAAAGTTGCAGATATGATATACGAGGAAATTTTTTCGATCCAATAGAAAATTTTAAAATAAAATCATCAAATTAAATTTTATTAAATGATGTATTGGTTGAAATAAGTTGCATATGTATAATTTGATAAAAAAAAATATTTTTTTATAATAATCATTGCTTTAAAAATCAATAACTTTCGTAAGAAATTTAATTGATTAATAATATAAAAATCTCTATACTTAACTATTTCTTTCAACTCAAAATATTTATTATTATTTTTTAATAAATTATTCTAATCATTATTTTTTTAGGAAATAATTGAATCTTTTTAATTTGAACGGGTAAAATCTATTTTATTATAAATTTTTCATTCTTTTTGTTTTTTTTACCGTCACTCTCTCATGTGTTTGAATTTATCAAAATTGAAATGTAAAATAACCAATTTATCAAAAATTATATTTATAAATTGATTTAAAAACGATTTATTTTACTCACTAATGCACGAAGTTAATATTTATGCACTGTTTTATATTATTATTTAATTATAAATTATTGTTTAAAATTTTTAATATAATTATTTTAAAAATTAATAAATTTATTGAGATGGGAAGAATGTGAAATGAATGACGTGCCTACCTTTTTTTCATATATTATACATTTATACTTCTGTCATCAGAAATACCATTTATTAGTTTCTAATAATTAAAAAGGATAAGAATCTCAATCCACATGCACATATATATATATAAGCAACACTTGTCCCTCTTACAAGTACAACAGCAAGAGTGGCTTTTTGGTGAATTGATTGGTGATCCCGAGTTGGTTGAGTTCACTGCGCATAGACATGTTCCTTAATTTCCCCAAATATTAAAACAAGATTCAAAGCACGTCACTTACGTTCCCGCTTTCTGTTTAACAAGGTACCACTCGTTTAGTCGTTTATTTGTACAATGCCTCTTCAATCATTTCAATTTGTTATATGATGAAGAAAAACCCAATTTTTATGTTGCTGCAGTGAAAAACTTGAACCTTTTGAGACCTCAGTTCTCTGCC

>GmALDH7B2

TAGGGAGAAAATGATTTGATAAAGAAAAAATAATACAGAAAAGTAATTTGACAAAATAAATCCACAGAAAATTAAAATTGTTAGAAAGTACATGAAAACTGAAATTTAAAATGAGAAATTTAAAGGTGTAAAATTATAGATTGATAGAAAATAAAAGAGTGTGAAACGTACAATGCAAGAAAGGAAATAATGACAAGAAAGAGTGTTAATATTATTTTTTAATATTAGTAATAAAACATTTTAATGTTTTTGTTAAATAAATATGTAGTATATTTTAATATTTTTATTTAAATTAAGTCTTTTTAATTATTTTTTTAATTTATAAAATAAATAATAATTCTTAACATTAGTAAAAAATATTAAATAAAACTAACAAAGAAAACATACATTAATTCATTAAATAATTAAAAAAATAGTTATGATTTAAAAAATATTAAAAAAAACTATTTATGATTTAAAAAAAGATTATATAGTCATTTTCGTCCTTAAATGTGTAGAGCGTTGAAAACTTCATCCTCAAAGATAAAAATTTAAATTTTAGTCTTCGAAGGAAAAAGAAATGCGACAAATTCATCTATCCGTTACAAGCATAACATTACAATAAACACTAAAAAAATAAAAAATTGAACATAAGTTAGAACAAACATAACAATAAGCATAATATTGATTAGTAAACTTAATCTGTATGTTGCTTTGATATTTCTTTGATAACATTAGGTAATGACAAAATCAAGTTAAGTCCCATTTTTATAAGAATTAACTTAACGGCTGTACATTTTTATTTGAGTATCATATTTTGAACGGTCACATAAGAAACTTTAGACTAATAGACAAATTATACTTATTAATTAATATGATGCTTATTATTATATTTGTTCTAACTTATGCTTATTCTTAATTTTTTAAGTATTTATTGTAATGTTATGCTTGTAATAATTAAAAAAGAAAAAAATGAAGATCAAATTATATTTTGGATAAATAACAATTTTTTTCCTTAGATATATAAAATGCTATCAAATTCGTGTATGAATGTTCTTTCATTAATGATAACAAATGAAAATTAAGGAATGAATAAATTTGTCATAGTTTTTCCTAATGTCAAAAATTTAAATTTTTATTTTCCGAGGAAGAATTAATTAACAACATTATGGACGAAAATAAGCATTTATTCAAAAAAATATAAAAAATATAAAATGAAAATAAAAAAGAAAACATTAATTCATTAAATCCCAAATGATCGTTTGCCCCATCATCATGAGTCTTCTTCGTTCTTATCGGTAAAAAAAAATCTAATATATTTAATTTTTTTTAAGTCCCACGTTATCTCTAAGTGCAAGCTTTATTGTACAAAAAAATCTGAATCCCCATCACTTTCCCCATCCCATGTGTGTCCCTGTCAGAGTATGATATGAGGAACTAACTCTATAAATAAAACTTTCTGAATCAAACACATCACACACACGTAACTTCATCTGGGTTGCAGGGAAAAAGAAAAATAAA

>GmALDH10A1

TAGCATTAAACGAATTTCTTTGTTTTAAATATAATAGGTACCTGATCAAAAAATTAATAATCTGTTCTACAATAATTTTACATGTAACTTTTAAAATAGAACATGTTTATATTAGGAAAACACATTATTGAGAGCGTAATATATCATTGTAACCATTATTTTTGTTTTCATTATGGTGATTGTTATCATAATTGTGACTACTATTTCAGTGATTGCCACAATCAAACAAATATATCTTATTCAAATTTAAAGGATACAGAATTTTTAAAAAAAGTTTATTTTGAAAGTATAAATTAAAAGAAAAAAAAAATCAAAACTGATTTTTAAAAACTTAGAACTAAAAACTACTCCAGGCTCTTAGTGTATATAGTGTATTTGGAAAATATAAATTAATTTATTTTTCAACAAAGTTAATTTATTAAGAAAATAATTAAATTTATCCTTAAAACATGAGACTAGCAAGTGTAATCAAGTTGGTTTAGATTCGATACGTAAAAACTGTGACTCAACCCAGTTAAATTTAAATGGTTGATTTTAGTTGATTTTCTACGAAAAAATAAACTCAATCCAAACATTTGTAAATAGTTTAGATTGGGTTGAGTTAAGTCAACATATCAAAACATTTAATTTTGTCAATTTTTTATAAAATGTAATATTCTTTATAAGTTATATTTTTTATTAAATGAATCAAAAACAATTATTTCTTATTAGATTTTTTTATTAAAAAAATTGTGATACTGTCACACCCTATTTCACTCTACTTACTCATATCTATCGAGAGAAAAAATTAAACATATCATATTTAAGATAAAAAATTTCAATAAATGAGAATGAACACTTTTTTCTATCATAATTAATAAGACATTGTCATGCTTACTATTTATAAATAAACCTAAAATATTTTTAGAAACCTATCTGAAATGGCTTTAATCAATAAAATTCTTATCATTTTGATCTTGAAGATTAAAAATGCACTAATATTTACTCAATTCAAGTTAATAAGTCTATGTAATCAAATACTCAATATTATTTGATTTTTTAAAAATTTTAGAAAATATATTATAATTACATATAGATATATTAATAATAATAATAATAATTTAATACAAATTAGCAAGTCAACGAACTAAATCAGTGGGATTTAAATGTTAAAATCTGTAATCTAATCCAAAATTCAACGTATTTGATTAGTTTAGTCTAAATATGACATAATTTCAAAAATTATTCAATTCAAATTGTCTGGATTAATTCGAGTTCAGAGATGATTCATGCCTACTTACATCTCATGTGAGACATTGATAAATTAATCTTGAAAAATAAAAAAAAATTGAAAATTAATATTTTAAATATGAAAAAAATAAAAATTAAATTTGCAGACAAATTCATACTTTATCTTTGCAACTTAACGATAAACTATTCTATTTAGGATAAAGAAGGAGAGACTGGACTAGCTGAGTTAGGGTGAGTTGCAGGCAGTGTCATCTCTCCTTTGACGACAAG

>GmALDH11A3

AAAATTTTCTTTCAAACATATTTTTTTTGGTAACTATGAAATTTATATAAAATGTCCACTAGATTTGTCTGAAAAAAATCTAATTAATCACTTGTAATAAATTTTGTTGTCTAATCAATTATGTTTTTTTATCCACGTAACTCTGTCTCTCAAACCTTAAGTATTACTTAAGATCCCAGCTCAATTTCAGTCAAATCGACGACATGTTTTTAAAAATATTGTTTATAATTTATTAATGTTTTAATTGAAGTATGTAAAAATACTAGTTTATTAGATAAACGCCATTTCTATAAATATTGAATTAAAACCATATAATTCAATAGTATTATATAATATTTATTCAAATTTTAATAAATTATTATCATATTAATATTCATATTTAAAACTAAAAATATTAAAAGATATTTATATATTTTCTCAATCACCATCACAATACCATCACAATGATACGGAATATTTACTGCCTTCCAGGGACTGACCCAAAAGTCTATATTGAAGGGATGAAATCTTTTTTCTTCCTTTGTAATTTTTTAAATCTCTAACTTATAATAAATAATACTTTTTAAAATATTTACTTTTTTACGCATAAAATTTGAAATTATTTTTTATTTTAAATGATAATAATTTTAACTATTATCATAATAACAATAAACACAACTAGTTTTATACAATTTTACACTAATTATCACTTGAATCATTGTCATAATAATAACAAATATAATTTATTTTACACTATCTAACCTTAATCATCATTATTTTATACCATATAAAATTAGTTTTACACAAAATTTACATCAATCAACTTAAAAATAGATATGCATATAAAAAAAAAAAAAAAATACCAAGGGATGGGGAGCTAGCCCCTGCCTGCCCCTCTACATCCGTCACTGCTATGCCGCCTTGGGAGACTAATTTCATATCTATAGATTATAACTAAAAACATTAAATAAAGAATTATGGGTTCCTAGTTGGGAGGCCTTTTACTTGCAATTTCGTTTTTCTATTTTATTTTTTGTTTTCACTTTTTAATTATAAAACTGTCACTTTGTTTTCAGTGATTTGTTTGTATAAAAAGACACTGAAAACAAGTTGATAGTTTTGTAATTAAAAAGTGAAAACAAAAATAAAAACAAAAAAATAGAATTACAAGTAAACCACCCCTAGTTTTCCGATTAAACAAGCCACGTTTATAATATCTATCATCCACAAATTAATACGAGGCGTGTCCTATATTTCAGTACTAAATTATTAATTAAACTGTTAACATTTTTTTAGTTCCAGCTAAACAGCCTGAAAGCACACTGATCGGCCAAGGACTGAAATAGACTAAGCACTAAGTATTAGCAACATTTATAGCTGTTCCTTGACCCTGTCTGAATCCACTTCAATAATCATGTTATACTAATAATAATCACCAACCACATAATCAACATCAATATATATATACTCCTAAGCCAAAGTACAAACCTCAAACCAAATCGAATCACTTGTTAGTTCAAC

>GmALDH12A1

TTTCAATAAATCTAGTTGTTGTAAATCTAGTTGCAGGAAAACTAACTTATTTTAAGAAATTAGTTTGTAGAAAATAATTGTGATACTATTATAATAAATCTCTCGTTAAAGAACAATTTATTTTAATTTATATTAGTTTATTTTAGAATACTATGTTGATGATAATAAATGAATCAATTTTATTACTTTTTGACATTTGATAATATTATGTTAATTATATTAGATATGTGGATAAATCATGATATAAAATAATAGTTATAAGAAAATAAAAAAATTAAATTTAAATGGATAATTCGTTGGCCCGAATCGAACCATTTATAAATGAATTGAGTTAAAAAAATGTGAAAACCGAATGAAATCAAACCAATCAAATTTGATTGAGTTAGGTATTAAATTTGATCAAAATTGATCTAAACTGATGTGTGAATACCCCTAATATTATATTATGCTCCCCCAACAAATGAACAAACAAATAGTAGAAATACAACTGAAAAAAATATTTATTTAGAAGAAAATAGAAATTGAAAAGTTAAAAGGTATTCTTTTATTGGAAAAATAGGAAAGCCATCTTCTACCCTGTAATTCAGACTTCAGTCGTTCCAGACTTTAATCGTTAATCAATTCGATACCCAGCTTGTTCTGTGATTTAGTTGAAATAGCCGCCAGTTATCTGAGTTGAAATTATGCTCATTGGAACTTTGATTCGTTCGACGATCAACTTGGGGTTCTCCAATAAAAAAATATAATAGAAAAAGTATATTTTATATGTATTTAGGTTTCTTACATCAAACAATAGAAAAAATGATTTTGAATGTTTATGATTAGTTCCGTGTTCTGCTTACAAACACCGATATTTCCGAGACTACTTTTTGTTACAGAAACATTTTAAGATGTTAGTTCTATAAAAAAAAATGATTTTAGTAACTAAGGACTTGGGAATAAGCAATTTAGCTAATGATTACCTGAGCTGAGTACTGTTTAGAAATTAGCAAGGCTAAAACTCTAAACTAGAATGTATCTTCACTGATTTACTTGCATCATAATAATACTTTATTGGCTACAATTCGCTGTCGTTTTGAAGGCCCAATTGGTTTGCTATTGTTGGTCTTACTACATTTACGCAAATTGACCGGGTTAGATCATATTTCGGAATTTAAGTCGAGCCGCTGTAGCACTGTATTATTTTTTATTTCTTCTTTTAAATAACTCACGGGGATGCTAGAAAAGTCATTTTTAAATTTCTTTGACACAGAAATAATTCTTCATCAGAGTTTACTTCCTGATTAATCAGGATCCTTTTTTTTTTTTTTATGAACCGGAGGGTTGATACCAAAAAAGAAAAGTCATTTCGTAATTTCCTCCAAGTCACCTTTTTTATAAAATAAATTCAAAGATTAAAAGTATTAGCTTTAGTAATTCTTTTTTATTTGATCATATTTATTGTACATATTTCTAAAATAGTTTATTATTACTTGATTTCATTACTATAATTTGTGATAC

>GmALDH12A3

GAAAACAAAATTTGACTTAAATTAATAAAATGCATTTTGGTTAAAGTTAGATTTTAATGAGTTAACTTACGTATGATTTAGGCTAGTTCAAAATCAGCATGATAAAAATATTGACAAAAATACGACATGAGGTTCCTTTTACAAATTATTTACCTAAATGGGTTGGGTCTGTTTTGAAATTATTTATCTCGTGGGTCTCTTTTTATACTATAAGGCGCTTCTCTGTTGGGCGCGTTTCTTGAAATGACGACACGTGACATGGGTTAAGAAGTGGTGACTTGCCAGGCGTGACCACACGTGGTGGCCTGCCAGGCGCGATCACACATGTTGGTCTGTCAGACACTTCCTTCCACGCCTCTCTATAAAACCCCTCTTCCTCCTTCGTTCCATTTGCACTACGAAATCGTTGAAGTGTTTTACGCTCAACTGAGTCTCACTCCTTACTGTTGTATGTATTATTGTTGAATTGCTTTATACCGGTTATAAATTTTGCCCAAAACTAATAAATGTTGTTATCTTCGATATATTATAGCATTATTTAGTATTTATTTTGTTGATGATAATAATGATTATATTTTGTAGGTGCATAAACAAATTGACACGGACGATAAATTTATTGTAACTTTGTTTATATTTTTTGTGTGATAAATAGTAATTTATTGTAACTTAATAATTTAATATTTGGTTATAATTATGTTGTGTTTATATATGTTATATTTGTTTGTTGATGACAATAATTATTTATTGTAGTTGTTTTTATGTCCACCACATTATTATTATTATTAGTTAAGCTCATAATTATTGATGTTGTTGGTATATTTTTTTAATAACCAACATTTACATAGACGTTACAAATAAATGTCGATTCTTTTATGTATCATTCACATTATCGTTATTTATTTTTATTTATTTTAAAAGTATTGCGCAATAAGATTGAAACGTTAAAGTATTTGAAAGGAAGTGTGGTCACGCCTGACACGTGTGGTCACGCCTGACAGCAGGCCACCACGTGTGGTCGCGTCTGACAGACCACCACTTCCTAACCCATGTCACGTGTCACCATTTCGGGGGGTGCGTTCAACACGGAGAAGCGCCTTGTAGTATAAAAAGAGACTCACGAGGTAAATAATTTCAAAACAGACCCATTTAGATAAATAATTTGTAAAAAGAACCCCATTTGATATTTTTGCCTAAAATTATTCGCTAGTTGAAGATCAGCTTGAGTCAAAATCTATTTTACTACTTGAGCTTAACTTGATTAGTCACAATATAATAATCATTGACAAAAAGAGGTAATTAACAATTATATCCGTACAAAATAAAATGTGAATAAAAGAAGATAAACTAACACAAGCAAAAATTATCAACCACCTCCCAGCAGGCCAGCACCACGGTAACTACTAACCAATATTTTCACTCAAGAGAAACACGATAAATCCCACGTGTACTCAGCAAGATCATCACTTCATTTTGGACTAAACTAGAAAGTGATCGAACAGC

>GmALDH18B2

ATAATAATAATAATAATAGGAATTATTATATTTGACATATAAATTACTCCTTTTACATTTAGGAGTATAAGAATCGGTTTTCAATGTTGGTGATTATACTAAACTTTTTACAAATAGTTAAACTAAATTAACTTTTAGAAAAAAATGTATTTGTAATTTATCCAAGAACATGCAATCATGCAAGTCAACTCATCATACCCAGCTATAGACACAGGTGATAGGCTCATAAAAAATTAGGAATACAGGATAAACCAGCTGGAGTGTATATTTAATTAATTAATCATTTTTTAAAATTTTGTTGCCTTGAGTTTTCCAATTTTGGTTGCAGGACAAGACAACGTGAGGGCTACAAACTATTTAAATGGAGTTTAGGTTGTTTACAGCCACCCTAGCTTTTTATAAATATTGAATGGTGAATATTTTTGCTGACGACGTTAGACAGTTGCAGAAAATCCACATGCATTCAAATTTTCAAATGACGTATCGCGTGTTCCACAAGGTTTTTATTTGTTATGATTTACAAAATTAACAAAAACAATTTGTTATTTTTAAAATAATGTTATTTTAAGAGGAAATAGTTAATTTAAGTAAGTTTATTGATTCTTAAAACTATTATTTTAAAAGTTATACTAATAATATTTTCTTATTGAACCATAACATGTAAAAATTTATATTAATTTTTCTTGTGTACAATAGCATCAATAAGATTTGAAAAAAATATTATATAAATTATCTAAATTTTCTCAGCGCTCTAAGCTGACGCTAATGAGTAAGTTTTTTTTGGCAAGTTAAACTGTTGTTTTAAACACTATCATAGTGACATAAACTAAAATAATAACATTAAATTCTTCTAGGTTAAGTCTATGGTTAATAGAATGAAAATTTTCTTTTTACAATAATTCTTTCACATTGCTCAATAACTAAACCACACAATATTTAACCAATTGATCTAAATTCGATTGTTACAGGACAAAAATTATAGCTGCACACTTATGTGTATGCAATTTTCTCATTATTAAATAAATTCTACATGGTAAATGAAAACACAATCTAAATTTCATTAAAAAATATATATTATTATAATTATAATTTAATAAAAAAAAGTTATTCACTCATAATATAATTTTTTTTACATCATCATTTAATTATATAATTTATCATAATAAATTTGTTAATTTTATAATAATTATTTAAAAAAGATGAATTTGTGATTGAAGTATTTTATCCTATGAGTGTAAAAGTATTTAAAAAAGATGAATTTGTGATTGAAGTATTTTATCCTATGAGTGTAAAAGTATTTTATGGTGTCTGATGATTAAACTACTCTTATAACTTTGCAGTATTTTTTTGTACATATATAATTTTGTAGTATTAAGGAGCGATTTGCCACGAAGGTGAAAGAAAGTAACGCGGCAGGTTGTGATTCGGCGGCGCTATCCACTTTGATTCCATTTGCATTCGAAGGTCTTCTCTGCTCCGCTCCAGTATCAGCACGCAGCC

>GmALDH18B4

AGAAAAAAAAATACTTTGAAAATGTTTGAATAATTTTTAAAATAATATAGCAAATTTCATATTAGTTGGATTTCTTTTAATTTGAGTGCAACAATAATAATAATAATAATAGGAGTTATTATGTTTGACATATAAATGAAGTAAAAAAAATGTTTGACATATTAATTACTCCTTTTACAAATAGGAGTATAAAAAGTTTTGTGATTATACTAAACTTTTTACAAATAATGAAATTAAATTAACTTTAGAAAAAATGTATTAGTAATTTAACCAAGAACATGCAATGATGCAAGTCAACTGATACCTAACTCTAGACACAGACACAGTTAGGTGATAGGCTTATAAAAAATTAGGAATACAGCGATAAAACCAGCTGGAGCGTAAATTAATTAATTAATCATTTTTAAAATTTTGTTGATTTGAGTTTTCCAACTTTGGTTGCAGGACAACGTGAGGGTTACAAAGTACAAACTATTTAAATGGAGAATATTTTTGCCCAAGAGGGCTACACAGTTGCAGAAAATCCACATGCATTCAAAATTTCAATTGACGTATCACGTGTTCCACAAAGTTCTTATTCAAAAAATAATAGCTCATTTTTATTTGTCATGCAAAATTAACAAAAGCATATTATTATTTTTAAAATAATATTATTTTAAGAGGAAATGGTTAATTTAAGTAAATTTATTGATTTTTAAAATAATTATTTTAAAAATTATACTATCAATATGATTGAACCATATCATTCAAAATTTTATACTAATTTTATTGTGTACAATAAGGTCAACCTCAACAAAATTTAAACCTAAAATATTATATAAATTATCAAAAAAAAATTATTATTATAAGTCAACCTTGATGAGTAATTTTTTGTCATAAAAATCAATTATTGTTTTAAACACTAACATTGTGACATAAACTAAAATAATAATATTAAATTCTTAATTTAGTATATGACTATGACTAAGAGAAATTTATAATAATTCATCCATATTATTAAATTATTTGAATCACATAATATTCAACCAATTAAGTTAAATTTAATAAATGCATGACAAAAATTATAGTTATATATAGAGAGAAAAAAAAATCAAATTACATTGATAATTTTGATAAATATAATATCATTTTCATAATTTATATAAAATATAATTTGATTGATCTGACCGTTAAATTATATTTATATATTAATAAAATTATCTATATTAAATTTTGTTAAAATTGAACAACAAAATTAAGGAAGAAAAGGTGGTTTCCAACAAAAATTAATCATCAATAAGATTAATAAATACTCTATACCAATTATATAATGATAAAATAATTTACATATTTATAATTATAATTTTGTAGTATTTTTTTTACATGATTTCATAGTACTACATTAAGGAGCGATTTGCCACGAAGGTGAAAGAGAGCAACGGGGCAGGTAATGATTCGGCGGCGCTATCCACTTTGATTCCATTCGCATTCGCAGGTCTACTGTAGAATCAGCACGCAGCC

>GmALDH22A1

TGAATGTGTTTCCAAACATGATGGATGTTTCAGCACAGGATTCTGCCGAGCGATGTAAAGGAAAATCCATAGACTGGATTAAGGAACAAACTGAATATTGATAGATGAGTGAAGGCTTCCAAGGGGAAAGGACAGTGAAAGCTGTTATGATCATACTTAAGCCTTGAAACTCTCTTACCTAGTGAATTAGTCTTTTGGATTGGACTCTCTTCTTGTACTTAAGTCCTAATAGTTGGACAACGATCTCTGAATAGAAGAGAAATCTACATTTACCTGCAAGACACTCTGATGCCAAAACTAATTAGTGTAAGAGATGCATAATATGAGCTTATATTCATAATAGATGCATTATATTTATATATATAAGAGGACTAAGACGTAATTCTATCAAATTTCTTTGGTAATTTAAGGCTTGTTATTTCAGTATTTCAATCTAGTTCACTTGTACATTTTTTTTTTTTGAATGGGTCAATTTCACTGTCTTCTGTCTCAGAAATACTGGTTTGTATGGAATGCATAGCCGTTAAGGAACAGGATTACAGGTATATCTGTTGAATGCTGTTTTTTTTCTTCTTTTTTTGCATGCATTGGTTTGTTTCGGGCTTTGTTTTTAGTTTGAATTTATATTTAAGTGTTTTTTTCCAATGATAGTGTTTTTGTAATGAATATGATTGATTTGGTCAATATACATTGTTCAAAACACTTTCAATTATTTGTCATGGATATGTGGCAAACTTAAAGAAACCCATAATTTTATTAGTTTTTTTTACTGGACTTCATTAGTTAATAATATAATTTTTTCATACTGATATACAAATTAATTTATATATATATATATATATATATATATATATATATATATATATATATATATATATAAATTATATAAGCACATACAAGTATTTTACTTTGGCATAAAACACCAATACTAATCAGAGAGAAAAAAGACACTTTCAGAACAGCCAGGCGGGTCCTGGGATCGAGAGAGACAACAAGATTTGTGACTTGTGAGGGACTCAGCGGAGACTTGCTGTCTATTATATAAGCCCATCTACTAAAAACATTCAAAGATATTACTACCATTTCTTTTCCTTTCCATTTTTTTTTTGTGTGGATTTTAATCCCTCCTTTCTTTCACAATAAAAAAGAGAAAGAAGGTATAGAGAGAACATAGGAGTCCCAGTAGAGAAGTAAAGTGCCATAAAAAACGACAAGACGCATCACGCTACGAATGGAATCTGTGTTTCCTTTTTCCTCATTTTCGTGTCACGGAGCATGAATTTTCCGTTTCGTTTTTACCTTGCTGACTAAGATTGTTATTGTTAATATCAATGCCATCCTCATCAACACGAAGCTTAAGCTTACGCGTTCACTGAGTGGGAGCATTTTCCTATAATACACTCCGCAGAAGCTTCGAGTTAAAGAGCATCTATCCAAATCGAAGCCCTAGCTGGTTTTCTTCCGATAGAAAGAGACGCGCTCTGAATCTGCGCATTTCCA

>GmALDH22A3

TAGTTTCTTTCATTATTTATTGATGTTGTGTCAACAATCTATTAAAGAATAAATTTAACATTTTTTAAATACCAAGGACTATAACAAATATATTTTAATAAAGAGCCAAAAATCTTGTTTTGATTCTTTATGTTTTTGAAGTATTTTGTTTGGTGCATTTTAACAAAAATGTTAAATATATTAATAGAAAAGTGTGATGTGTTTGTTAATGTTAATTAAGATGATGATATGAAATGTTAATTGAAATAACATTGTTTCAAATAATATTATTTTGATCCTTTGATATTTTAAAATGATTTTAATTCACCTCATCAAGTTTTTAAATGGTTTCAATTTTTGATCATTTAAGTTTATGAAAAGTTTCATATTAGCAATACTAATTTCATACTCACATACTCAATATCTATCTCTAGTAGCATATGATCTTTTCCTCTTTCTCACAATAATCATATCTTTTTCGTCCTTTGATCTACTACTTCAAGTAGATGTTGATAAGTCTTCCCCTATCAATAAATTCAACAATACTCATTATCCATGACAACATATCCAATAACAAGCATCTTTGACCACCTCCTAATACAACTTAAAGAGTTTTGTATGGTTAGAATTCTTTTCCTCTACCTCATAATCAGGGCAAAGAGAGAGGTTGACCCGGGGATGCCATGGTCACCCCCAAAAATTTGCAACTTCCTTTAGAATTCATATATGAAAGCATATTATGGCTATCCTAAGGGGAAAAAATGAGATATTTGATCTTGCCCCCATGAAACCAAAAGTTGCCATTGCCACTACTCATAATAACCATGTATTCCAAGTGATAGATTAACATTGTCTTAAGAATCAAGAATAGGTTGTATTATGAAATATATCAATGTAATTTGATAATTAATTATTCAAATTAAGGTGACTTTGTCTCGATTATGAATGTACTAGTTTAGTATTCAATATTTTAATATTTTGTATTCTCGTGAATTATATAACCAATGTAAAAAAAGAGAAAATAAAAAGATAAACTGTCACCTACAAAACAAACAAAAAACATAAAAGACCAATTAAAATAACGTCTCATGTATTATAAATGACAAAAGATCATCTTGCATAAATTGTTTTTACCCATTTAGGTAAATTATTTTTTAACCCATCTTTTGTAAAAAGTCGAGATTTAGATTATAATTAACTATACTTTTTGAGTGAATGTTAAATGTACGAATATCTTTTTCCTATTGCCTGTATCTACAATCTTGTATCTTGTATCTTGTATGTACTATGTACTTACTGAATCAACTAGTGAGTTAATTAAAAGAGCACTTAGTGACGTAGTTAGTACAACACTAACAACAGTATCAAGACAATTTCTTCTTTCATCGCCTAAAGCTTCCCCAGATTCTTTCACTGTCTTCAACCATTGTCCTCAAAACAACAATCGAAACTCCGCGGAAGCTTCTCGAAACCCTACCGAGGCGATTTTCTCACTCACTCCGAATCTGCTGCCAAGTTCCA

>GmALDH22A4

TTGAGTGATAGACTGATAGTGGTGTGAGAATTGAGATGGAAGAGTTTAGAAGGACATGCAGAAAAATAAACTTGGACCCATCTTTTACCTTAATTTATTAGTGTTACTACTTACAATGATTCTCTAAACACATTGTTCGAATGCGTTTCCAAAGATGGTGAATGTTTCAGAAGAGGATTCTGGCGAGCAACAACGGCCTCTAGTTAGAAGTAAAATCTGTATTTACCCGTAGAAACACTCCAATGCTAAAGTAAAAAAATGTAAAAGATGCATAATATGAGCTTATATTCTTAATAGATGCATAGTATTTATTTAAGACTAAGACCTAATGTTATCGAATGTCTTTGGTAATCTAAAAGGCTTGCTATTTCAGTATGGTTCAGTCTGACCAAGTTAATTTCCACTTCTAGATGAGATGTTTTATAGTAGTAATTGACTGGTGAAATTTTTGTGAGTCATGGATAATGTGACAAGCCTAAAGAAGTCCATAGTTTTTATTAGTTTTTTTTACTGAACATTTATTAGTTAATAATATAATTTTTATATACTGATATACAAAGTAATTATATTTATAGCATAATATATAAGCACATATTATTTTGGTATATAAGATATTATTATTCTTATTAAAACAAAAAAAATAATTATTTTTCTTTAAAGAGTATAAATAATTTGAAAAAAAAATCTTGTAATTAAAGAATCAGAAATTACTTTATAATAATGTCAATGTTAATCAGAGAGAAAAAGACACTTTTTTGCACGCTGATAAGTTCAAACTTTACCTCAGAACAGCCATGCAGGTCCTGGGATCGAGAGATCGAGAGAGAGACAACGAGTTTTGTGACTTGTGAGGGACTCAGGGAAGATTGCTGTCTCTTTATATAAGCGGCTAAGCTCATCTACTAAAAATATTCAAGATATTATCAACTATCAAGTACCAGTACCATTTCTTTCTTTACTATTTTTTCTTTGAGGATTTTAATCCCTCCTTACTTTCACATAAAAAGAGAGAGAGAGAAAGAAGGCATAGACTAGAGAGAACATACGAGTCCAATAGAGAAGTAAAGTGCCATAAAAAACGACAGGACGCATCACGCTACGAATGGAATCTGCGTTTCCTTTTTTCCTTATTTTCGTGGCACGGAGTATGAATTTTCCGTTTCGTTTTTTTTTTCCGTTTCGTTTTTTCCTTGCTGACTATGATTGTTATTGTTAATATCAATGCCATGCTCATCTCCACGAGCTTAAGCTTACGCATTCACTGACTGGGAGCATTTTCCTTTAAAACACTCCGCAGAAGCTTCGAGTTAAAGAGCATCAATCCAAATCAAAATCCCTAGCTGGTTTTCTTCCGATTCGAAGAGAACGCGCTCTGAAACAGCGCATTTCCAATGGCGTTTTGGTGGCCGTTGCTCGTTTTAGCATTCGCTTACGGTATTTGTAGGTTCCTTCTCATGCTCATTCCTCCTAAGGTTCCTTCCATCGACGTTGACACTTCCG
